# Can plant DNA barcoding be implemented in species-rich tropical regions? A perspective from São Paulo State, Brazil

**DOI:** 10.1590/1678-4685-GMB-2017-0282

**Published:** 2018

**Authors:** Renato A. Ferreira de Lima, Alexandre Adalardo de Oliveira, Gabriel Dalla Colletta, Thiago Bevilacqua Flores, Rubens L. Gayoso Coelho, Pedro Dias, Gabriel Ponzoni Frey, Amaia Iribar, Ricardo Ribeiro Rodrigues, Vinícius Castro Souza, Jérôme Chave

**Affiliations:** ^1^ Departamento de Ecologia, Instituto de Biociências, Universidade de São Paulo (IB-USP), São Paulo, SP, Brazil; ^2^ Departamento de Ciências Biológicas, Escola Superior de Agricultura ‘Luiz de Queiroz’, Universidade de São Paulo (ESALQ-USP), Piracicaba, SP, Brazil; ^3^ Departamento de Biologia Vegetal, Instituto de Biologia, Universidade Estadual de Campinas (UNICAMP), Campinas, SP, Brazil; ^4^ Escola de Artes, Ciências e Humanidades, Universidade de São Paulo (USP), São Paulo, SP, Brazil; ^5^ Laboratoire Evolution et Diversité Biologique, UMR 5174 CNRS, Université Paul Sabatier, Toulouse, France

**Keywords:** Biodiversity assessment, plant barcoding, species conservation, tree species

## Abstract

DNA barcoding helps to identify species, especially when identification is based on parts of organisms or life stages such as seeds, pollen, wood, roots or juveniles. However, the implementation of this approach strongly depends on the existence of complete reference libraries of DNA sequences. If such a library is incomplete, DNA-based identification will be inefficient. Here, we assess if DNA barcoding can already be implemented in species-rich tropical regions. We focus on the tree flora of São Paulo state, Brazil, which contains more than 2000 tree species. Using new DNA sequence data and carefully assembled GenBank accessions, we assembled 12,113 sequences from ten different regions. The ITS, *rbc*L, *psb*A-*trn*H, *mat*K and *trn*L regions were better represented within the available sequences for São Paulo tree flora. Currently, only 58% of the São Paulo tree flora currently have at least one barcoding sequence available. However, these species represent on average 89% of the trees in São Paulo state forests. Therefore, conservation-oriented and ecological studies can already benefit from DNA barcoding to obtain more accurate species identifications. We present which taxa remain underrepresented for the São Paulo tree flora and discuss the implications of this result for other species-rich tropical regions.

## Introduction

DNA barcoding consists in telling apart organisms based on specific regions of their DNA. Initially devised as a tool to identify microorganisms, such as bacteria (Woese *et al.*, 1990), its use can be extended to all life forms, as long as DNA can be extracted from living, dead or fossil material. This approach developed rapidly for animals, for which one gene fragment, the mitochondrial cytochrome oxidase I (COI), was shown to be a reliable barcode in important lineages (Hebert *et al.*, 2003). For land plants, however, the use of COI is inappropriate (Adams and Palmer, 2003) and the search for a substitute barcode has been challenging (Chase *et al.*, 2007; Hollingsworth *et al.*, 2011; Dong *et al.*, 2015). Today, the consensus view is that different genomic regions need to be jointly sequenced to properly identify a plant species (Chase *et al.*, 2007). The chloroplastic *rbc*L *and mat*K markers were the first proposals of barcodes for land plants (CBOL Plant Working Group, 2009), with further suggestions for the inclusion of ITS region and *psb*A*-trn*H as core barcodes (Gonzalez *et al.*, 2009; Hollingsworth, 2011; Li *et al.*, 2011).

DNA barcoding is a powerful tool for a range of applications. It can be used to provide objective identification of allergenic, illegal-trade species or any species relevant to conservation, such as threatened or invasive species (Armstrong and Ball, 2005; Lahaye *et al.*, 2008; Pryer *et al.*, 2010; Krishnamurthy and Francis, 2012). It can also be helpful to identify species in ecological studies (Joly *et al.*, 2014), especially in species-rich tropical forests and/or when identification relies on sterile materials or on cryptic life stages, such as seedlings and seeds (Gonzalez *et al.*, 2009; Gomes *et al.*, 2013). For instance, in the Brazilian Atlantic Forest, tree community surveys often result in 5-10% of trees as unidentified or identified at family level, even in regions where the local flora is well studied (e.g., Grombone-Guaratini *et al.*, 1990; Ruschel *et al.*, 2009). Where the tree flora is less known this fraction can reach up to 20% (e.g., Thomas *et al.*, 2008). Although it has been claimed that DNA barcodes have limited use for taxonomic studies (Rubinoff and Holland, 2005; Seberg and Petersen, 2009), they have been used for reconstructing phylogenetic relationships within communities (Kress *et al.*, 2009; Oliveira *et al.*, 2014), thus providing a significant contribution to the field of ecophylogenetics (Mouquet *et al.*, 2012). With the advent of high-throughput sequencing, the application of DNA barcodes to environmental samples (e.g., soil samples, feces or stomach contents) has been successfully used in biodiversity surveys (Taberlet *et al.*, 2012; Yoccoz *et al.*, 2012; Ji *et al.*, 2013; Kress *et al.*, 2015) and also to unravel species interaction networks (Yu *et al.*, 2012; García-Robledo *et al.*, 2013; Lima-Mendez *et al.*, 2015; Pornon *et al.*, 2016).

These applications, however, depend on the existence of complete reference libraries of DNA sequences to which new sequences can be compared with to perform species identification. If a reference library has a poor coverage in terms of species with sequences already available, the implementation of DNA-based identification techniques will remain inefficient. This is a basic issue of the implementation of DNA barcoding, but the limits related to the availability of comprehensive reference libraries have seldom been evaluated empirically. Reference libraries of DNA barcodes are expected to be far from complete in most of the tropics. However, we hypothesize here that in spite of the incompleteness of the reference libraries, DNA barcoding can be implemented if most of the individuals in a given region have reference sequences in the existing reference libraries. This critically depends on the relative abundances of the species under consideration and whether they have a large or restricted geographical distribution.

Here we perform such an assessment for the tree flora in one species-rich tropical region, the State of São Paulo, Brazil. This area has high plant diversity and endemism, but also has high levels of land-use change and forest fragmentation. It is the Brazilian State with the largest level of research funds, which would in theory allow for a thorough survey of its flora. Here, we combine sequences from a large sequencing effort carried out locally with sequences retrieved from GenBank to answer the following questions: (i) How many barcodes are available for the São Paulo tree flora, has this number increased through time, and which taxa still remain underrepresented? (ii) Which DNA markers are better represented among São Paulo tree species? (iii) What proportion of trees in São Paulo forests already has barcode sequences available? (iv) Do species with larger geographical distribution tend to have more information on DNA barcodes? In the light of our results, we finally propose directions to future sequencing efforts to achieve a more complete DNA reference library for São Paulo, which could be applied to other tropical areas.

## Materials and Methods

### Study site

São Paulo State in south-eastern Brazil (19º47’-25º19’S latitude and 44º10’-53º06’W longitude) has a total area of 248 thousand km^2^, representing ca. 3% of Brazil. Altitude varies from sea level to nearly 2800 m asl. Climate is mainly characterized by a marked rainy summer and dry winter (Dufek and Ambrizzi, 2008). However, Köppen’s climate classification ranges from Aw in the west of the state, Cfa and Cwa in its center, Cfb and Cwb in the mountains to Am and Af near the ocean (Alvares *et al.*, 2013). Mean annual rainfall and temperature for the 645 counties of São Paulo ranges widely from 1226 to 3150 mm and from 17.1 to 25.4 ^o^C (http://www.ciiagro.sp.gov.br). São Paulo is one of the Brazilian states with the highest degree of knowledge in terms of its flora. It shelters 7528 seed plants, mainly within the Atlantic Forest and Cerrado domains, two important biodiversity hotspots of plant and animal endemism (Myers *et al.*, 2000; The Brazil Flora Group, 2015).

### Species list, abundances, distribution and conservation status

We compiled a list of all native and naturalized woody species occurring in forests of the state of São Paulo, including trees, tall shrubs and arboreal species of palms, ferns, cactus and bamboos. We did not include woody lianas, but we did include woody hemi-epiphytes species of *Ficus*, *Coussapoa*, *Spirotheca* and *Oreopanax*. Hereafter, we will refer to this checklist as the SP tree flora. This list is based largely on the Brazilian Flora Checklist project (The Brazil Flora Group, 2015) which provided 91% of species records for the São Paulo tree list. The remaining records came from the NeoTropTree database (Oliveira-Filho, 2014), which contains an extensive list of trees and tall shrubs frequently sampled in forest surveys. We manually checked all records in the SP tree flora to ensure that the final list did not contain nomenclatural problems or records for other life forms such as lianas and small shrubs. Here, we considered as doubtful the occurrence *Acanthosyris spinescens* Griseb. (Santalaceae) in São Paulo, for which we found vouchers only for the state of Rio Grande do Sul in the speciesLink network (http://splink.cria.org.br).

The abundance of tree species in São Paulo forests was obtained from 135 published tree community surveys (Table S1). These surveys were compiled by Lima *et al.* (2015) for the Atlantic Forest, and were complemented by additional surveys for Cerrado forests (i.e., *Cerradão*), obtained using the same search methods. Most of surveys (53%) had total sampling effort ranging from 1 to 10.24 ha and a cut-off criterion of diameter at breast height ≥ 4.8-5.0 cm. Overall, the 135 surveys contained abundance records from around 174 ha of natural forests and 269,000 individuals. These surveys were used to calculate the proportion of individuals in each community belonging to species with DNA barcodes currently available. The distributions of these proportions across sites (and their descriptive statistics) were taken here as a measure of the extent to which DNA barcoding can be used to assist the identification of tree individuals occurring in São Paulo forests. This assessment was carried at species, genus and family levels. Records determined as a*ffinis* were considered only at genus level, but species determined as *confers* were attributed to the suggested species.

The geographical distribution of species was also obtained from the Brazilian Flora Checklist and NeoTropTree projects (Oliveira-Filho, 2014; The Brazil Flora Group, 2015), which provide the occurrence of species at country level and at state level for Brazilian records. We classified species geographical distributions as follows: non-endemic, Eastern South-American endemics (Atlantic Forest, Cerrado and/or Pantanal domains), regional endemic and local endemic. Regional endemics are those species restricted to South and South-eastern Brazil, North-east Argentina and Paraguay. Local endemics are species restricted to the State of São Paulo or to São Paulo and one more neighbor state, e.g., São Paulo and Rio de Janeiro. We obtained the threat status of species at state level based on the São Paulo official red list (Mamede *et al.*, 2007). A species was considered as being under threat of extinction if its current status was vulnerable, endangered, critically endangered, extinct in the wild or extinct, following the IUCN threatened classes.

### DNA sequences library

The library of DNA sequences for the SP tree flora was assembled from two sources: local sequencing efforts and sequences retrieved from GenBank. Local sequencing was carried out by collecting up to three samples of all tree and shrub species inside four 10.24-ha (320×320 m) forest plots of the ‘São Paulo Permanent Forest Plots’ project (Rodrigues, 2005). Together, these plots contained > 60,000 of trees from > 600 species in the four of the major forest types of São Paulo: the savannah forest (locally known as ‘*Cerradão*‘), the semi-deciduous seasonal forest, white-sand forest (locally known as ‘*Restinga*‘ forest), and the rain forest over clayey soils. In addition to the tagged trees inside each permanent plot, we also sampled the most abundant shrubs that usually did not reach the 4.8 cm diameter at breast height (mainly *Piper* spp., *Psychotria* spp., *Geonoma* spp. and *Leandra* spp.). In a second moment, additional sampling campaigns were carried out aiming to collect samples of species from orders and families that were not present in the four 40.96-ha plots. We performed rapid surveys in 40 forest fragments in 38 counties of São Paulo, collecting fertile specimens in the forest border and interior. Species identification was validated by consulting several specialists and taxonomic revisions. Vouchers for all species sampled were deposited at ESA and RB herbaria (acronyms follow Thiers, 2015). Finally, for the orders and families that were eventually not found in the field campaigns, we sampled leaf fragments from herbarium specimens deposited at ESA. In total, we obtained more than 2000 leaf samples from the permanent plots, forest fragments and herbarium specimens.

We assembled DNA sequences for *rbc*L and *mat*K for all families and the ITS region specifically for Myrtaceae and Melastomataceae, two species-rich families in the SP tree flora for which we had low amplification success for both plastid barcodes. Our choice of markers was related to an ongoing initiative funded by the state government to produce a complete reference library of plant DNA barcodes for the entire state of São Paulo, starting with the two core plastid barcodes. The DNA sequences were obtained using classic procedures of DNA extraction (Doyle and Doyle, 1987), for all leaf samples collected. For the DNA extracts that could not be easily amplified, we repeated PCRs using slightly different protocols or using purified DNA extracts. These cases generally corresponded to materials sampled at the ESA herbarium, and from latex-containing species (e.g. Euphorbiaceae, Moraceae and Clusiaceae) and those with secondary leaf compounds that may act as PCR inhibitors (Varma *et al.*, 2007), including Myrtaceae and Lauraceae. Details on DNA extraction and primers choice are provided in Table S2. Scientific permissions to access the studied sites and to sample and transport plant tissues were provided by ‘Instituto Florestal de São Paulo’ (COTEC processed 260108 - 002.446/2011 and 002.089/2014) and the ‘Instituto Brasileiro do Meio Ambiente e dos Recursos Naturais Renováveis’ (IBAMA - License 14BR013854).

We complemented this sequencing effort by compiling all accessions from GenBank (www.ncbi.nlm.nih.gov/genbank) for the valid binomials and synonyms of the SP tree flora recognized by the Brazilian Flora Checklist project. Our search included only accessions deposited until 31 December 2015. It was conducted using the ‘organism’ search field and was restricted to vascular plants. Only accessions verified by GenBank were considered. For each verified accession retrieved, we extracted the information on the date of submission, molecule type, the origin of the specimen, and the gene to which the accession referred to. We then classified genes as barcoding and non-barcoding plant DNA regions. The majority of studies on plant DNA barcodes concentrate on *rbc*L, *mat*K, the ITS region (i.e. 18S|ITS1|5.8S|ITS2|26S) and *psb*A*-trn*H (CBOL Plant Working Group, 2009; Gonzalez *et al.*, 2009; Hollingsworth, 2011; Li *et al.*, 2011). However, for the sake of completeness, we also included other five regions suggested as potential barcodes by Hollingsworth *et al.* (2011), namely *atp*F-*atp*H, *psb*K-*psb*I, *rpo*B, *rpo*C1 and *trn*L, and finally *ycf*1, as recently suggested by Dong *et al.* (2015).

### Data analysis

We evaluated how fast DNA barcoding data have accumulated for the São Paulo tree flora. To this end, we plotted the cumulative number of sequenced species, genera and families against time. The accumulation curves were fitted using the logistic function [Accumulated sequences = *a*/(1 + *c*exp(*b*time)); see Fattorini *et al.* (2012) and citations therein]. Because we know the total number of taxa for the SP tree flora, we fixed the saturation parameter *a*, so that we could estimate the date when the accumulation curves saturate according to the best logistic fit to data. We fitted functions using non-linear least squares regression (Pinheiro and Bates, 2000). GenBank accessions were restricted to dates until the end of 2015, so we arbitrarily set the date of 16 Jan 2016 for the sequences produced as part of this study. GenBank accessions are not published continuously throughout the year, so accessions were grouped by trimester prior to fit functions to the cumulative number of taxa.

Within each family and genus, we calculated the difference between the overall proportion of species in the SP tree flora and the proportion of species with DNA barcodes. We fitted a Cauchy distribution to the values of difference between these proportions using maximum likelihood and calculated the 95% and 90% confidence intervals to determine which groups had an under or overrepresentation in the DNA barcode library of the SP tree flora. The Cauchy distribution, which is a special case of the Student’s *t*-distribution, is also symmetrical but has a heavier tail than the Normal distribution, being more flexible to assess the distribution of values centered on zero. We also performed a priority assessment to indicate which families should be prioritized in future barcode sequencing efforts of the SP tree flora. This assessment took into account: (i) the proportion of barcodes not available, (ii) the proportion of threatened species without barcodes, and (iii) the proportion of endemic species without barcodes (see details in Table S3). We summed these three proportions to calculate a priority index varying between 0 and 3. The higher the index, the greater the priority for future sequencing efforts. To visualize this result, the priority index was mapped upon the phylogeny for the SP tree flora. This phylogeny was obtained at family-level using the stored megatree R20120829 available from the phylomatic website (http://phylodiversity.net/phylomatic)

To assess the representativeness of each barcode in respect to the São Paulo tree flora at different taxonomic levels, we implemented the following procedure. We calculated the abundance of sequences at order, family, genus and species taxonomic levels and then computed the Pielou index (*J*):

$$J=\frac{\left[-\sum pi\times \ln\left(pi\right)\right]}{\ln\left(S\right)}$$

where *p*_*i*_ is the relative abundance of sequences for taxon *i* and *S* is the total number of taxa for a given taxonomic level. This index accounts not only the number of taxa for which a given barcode has sequences available, but also how concentrated these sequences are across taxa. Venn diagrams with different combinations of barcodes were used to represent the overlap among barcodes in terms of species sequenced.

To assess whether the number of barcoding sequences depended on species geographical distributions, we performed an analysis of variance using a negative binomial error distribution (i.e., negative binomial regression). The response variable was the count of barcoding sequences available per species, which varies from 0 (no barcode at all) to 10 (sequences for all barcodes), and presented a skewed distribution with much more small than large counts per species. This variable was regressed on species geographical distribution, namely non-endemic, Eastern South-American endemics, regional endemics and local endemics. The significance of this regression model was tested using a Likelihood ratio test. All analyses conducted were performed using R (R Core Team, 2017).

## Results

The SP tree flora contains 2097 species (including 55 naturalized species) from 36 orders, 107 families and 508 genera (Table S4). It represents around 85%, 68% and 48% of the families, genera and species of native trees recorded for the Atlantic Forest and Cerrado domains together (Oliveira-Filho, 2014). Besides trees, tall shrubs comprised 13% of species while palms, ferns, cacti and bamboos summed 4%. We found 227 species threatened of extinction at state level (11%), including six species that are probably already extinct in São Paulo.

### The DNA barcode library for the SP tree flora

The first DNA barcode accession for SP trees was an *rbc*L sequence submitted to GenBank in December 1993 for *Trema micrantha*, a widespread Neotropical pioneer. But it was only in this century that the number of accessions increased in an exponential fashion, almost doubling at every three years in the past ten years (accumulated sequences = 61.3exp(0.063*time*)). By the end of 2015 GenBank contained nearly 30,000 accessions for the SP tree flora, of which 39% corresponded to sequences of the ten DNA markers considered here (Table S5). Few studies made great contributions to this increase (Kress *et al.*, 2009, 2010; Lucas *et al.*, 2007, 2011; Bolson *et al.*, 2015; and the iBOL Data Release in early 2012), representing 99-791 sequences of 38-86 species. We added 1611 new sequences (902 *rbc*L, 587 *mat*K and 122 ITS region) for 609 species (GenBank accessions: MG707972-MG708099, MG718033-MG719104, MG833417-MG833723, KF981191-KF981364), among which more than 80 are new species entries in the NCBI taxonomy database. This is by far the largest sequencing effort ever carried out for SP tree species. Today, there are currently ca. 12 thousand DNA barcodes available for the SP tree flora (Table 1).

**Table 1:** Number of sequences and taxa by source of information for the São Paulo tree flora, in respect to the ten DNA barcodes considered in this study. In parentheses, the proportion of each taxon with sequences available for any of these ten barcodes.

Source of sequences	Accessions for SP trees	Families	Genera	Species
		(n=107)	(n= 508)	(n= 2097)
GenBank	10,493	92	401	1,059
This study	1,611	89	302	609
Total	12,104	106 (99%)	456 (90%)	1,214 (58%)

**Figure 1:**
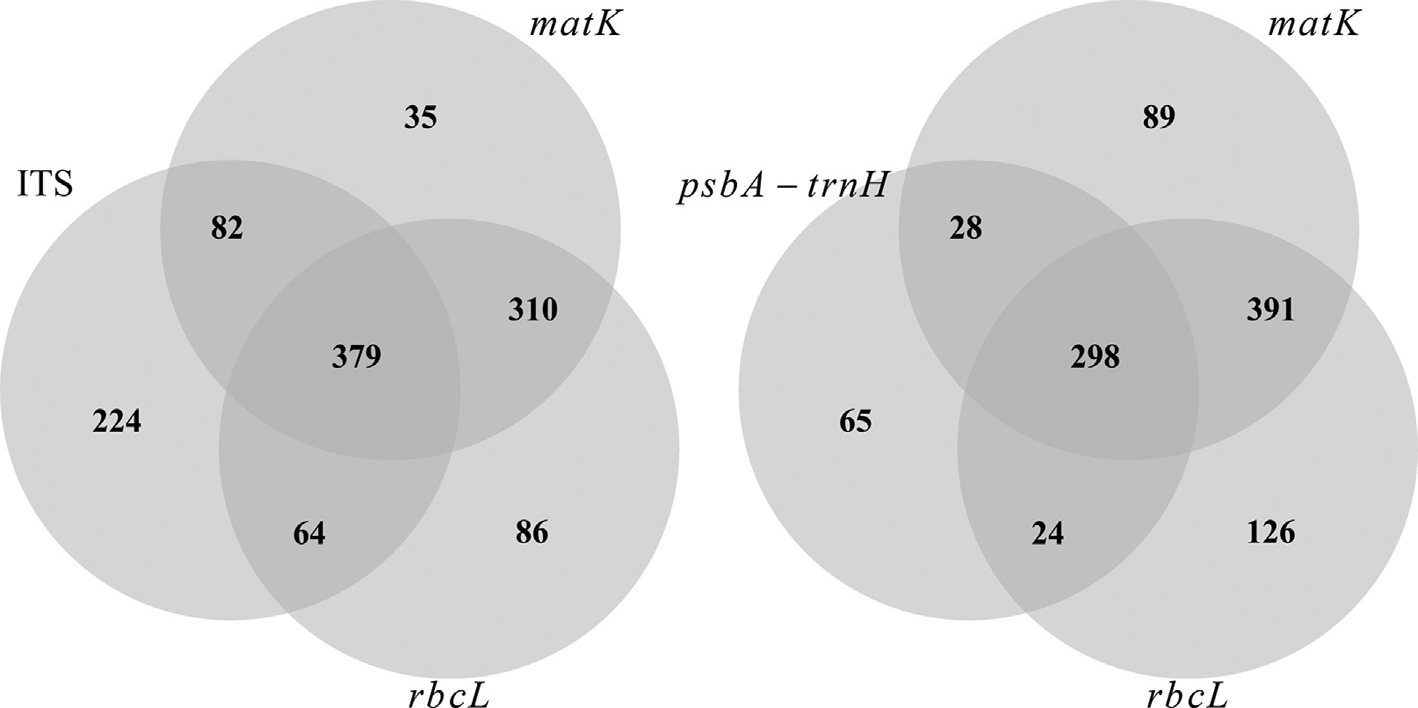
Venn diagrams of the number of species with sequences for the combination of (a) *rbc*L + *mat*K + ITS region and (b) *rbc*L + *mat*K + *psb*A-*trn*H.

The top five DNA barcodes were ITS, *rbc*L, *psb*A-*trn*H, *mat*K and *trn*L, these being the only barcodes well represented for the São Paulo tree flora. Accessions for the other five markers (i.e., *atp*F-*atp*H, *psb*K-*psb*I, *rpo*B, *rpo*C1 and *ycf*1) were so scarce (< 4% of all barcode accessions) that these markers will not be discussed hereafter. We found that *mat*K and *rbc*L had the highest evenness across taxonomic levels. In terms of evenness, *trn*L also outperformed the ITS region. None of the barcodes had sequences for more than 40% of SP tree species (Table 2) and only 8% species from the SP tree flora already have sequences for all of the top five barcodes. These proportions are higher if we consider combinations of two barcodes, such as *rbc*L+*mat*K (33%), *rbc*L+ITS (21%), *rbc*L+*psb*A-*trn*H (15%) and *mat*K+*psb*A-*trn*H (15%), but they are slightly higher for three-barcodes combinations, such as *rbc*L+*mat*K+ITS (18%) or *rbc*L+*mat*K+*psb*A-*trn*H (14%, Figure 1).

**Table 2:** Number of sequences, species and the evenness index of the ten DNA markers considered in this study regarding the São Paulo tree flora. In parentheses, the proportion of species sequenced with respect to the total of tree species occurring in São Paulo.

DNA marker	Sequences	Species (%)	Evenness (Pielou *J*)		
			Family	Genus	Species
*atp*F*-atp*H	74	35 (1.7%)	0.270	0.247	0.414
ITS region	3,380	805 (35.5%)	0.658	0.683	0.673
*mat*K	2,047	855 (37.1%)	0.779	0.867	0.820
*psb*A*-trn*H	2,290	448 (19.7%)	0.605	0.598	0.545
*psb*K*-psb*I	67	42 (2.0%)	0.283	0.326	0.435
*rbc*L	2,403	886 (39.9%)	0.820	0.871	0.818
*rpo*B	123	76 (3.6%)	0.586	0.551	0.523
*rpo*C1	171	89 (4.1%)	0.572	0.531	0.528
*trn*L	1,544	560 (24.9%)	0.698	0.763	0.694
*ycf*1	24	17 (0.8%)	0.463	0.359	0.352

**Figure 2:**
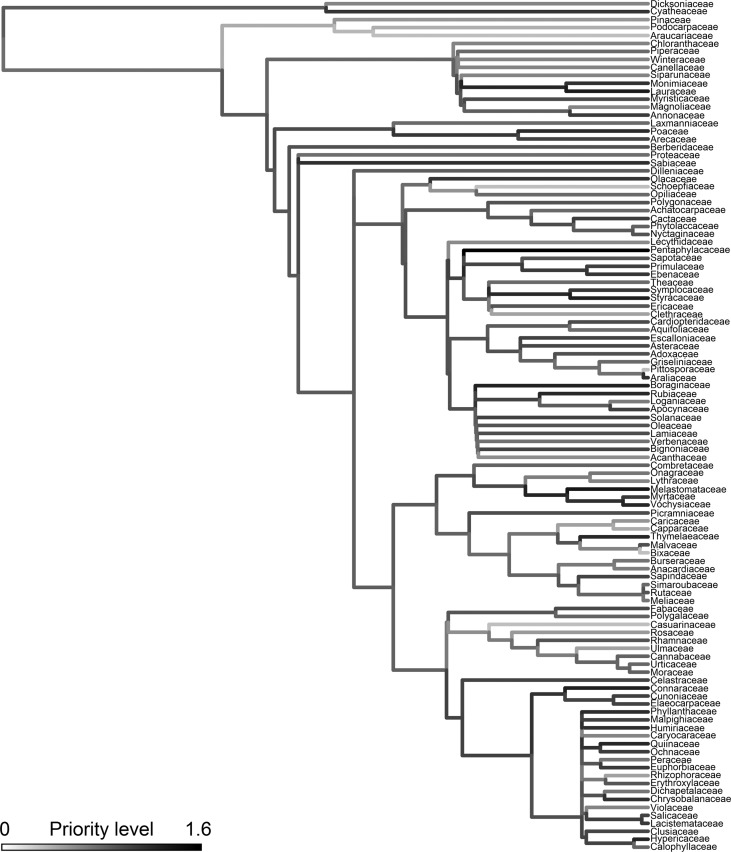
The sampling priority level in future sequencing for each family of the SP tree flora, plotted against its phylogenetic hypothesis. The priority level was defined based on the representativeness of DNA barcodes within the family and the proportion of threatened and endemic species without barcodes.

Genera that have proportionally fewer species with DNA barcodes than average were *Ocotea*, *Erythroxylum*, *Psidium*, *Mollinedia*, *Vochysia*, *Solanum*, *Merostachys*, *Calyptranthes*, *Baccharis* and *Eugenia* (Table S6). The only family lacking barcode information is Schoepfiaceae, represented by *Schoepfia brasiliensis*, for which we did not manage to amplify neither *rbc*L nor *mat*K. Regarding the threat and endemism status of species without barcodes, other groups come out as priorities for future sequencing efforts: Dichapetalaceae, Escalloniaceae, Hypericaceae and Pentaphylacaceae (Figure 2, Table S3). They are all rare in SP, have high proportions of threatened and endemic species, but lack DNA barcode sequences.

### Can DNA barcoding be implemented for the SP tree flora?

**Figure 3:**
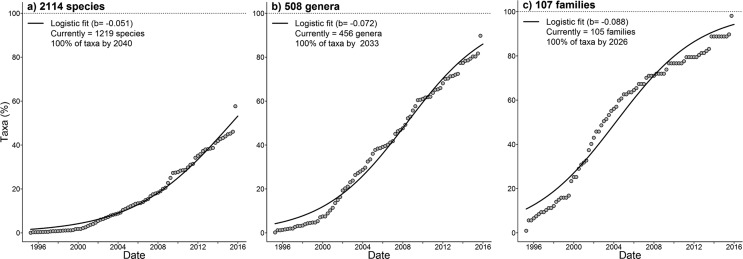
Increase of the number of (a) species, (b) genera and (c) families across time with respect to the ten DNA markers considered in this study. Bold lines are the best fit of the logistic function to the accumulated number of sequences or taxa. Dashed lines are the total of taxa in the SP tree flora. Dates are aggregated in trimesters and the fit of functions to the accumulated number of taxa was carried out only from mid 1995 on.

Although the number of accessions has increased in an exponential fashion, the accumulated number of taxa with barcodes has increased much more slowly. Currently, over half (58%) of SP tree species have DNA barcode information. If current accumulation rates remain the same, 100% of its families, genera and species will have at least one barcoding sequence available by 2026, 2033 and 2040, respectively (Figure 3). Few species were overrepresented with more than 500 sequences each (e.g., *Lychnophora ericoide*, *Handroanthus impetiginosus* and *Tabebuia aurea*).

Taking into account the abundance of the species with at least one DNA barcode currently available, they correspond on average to 88.9% of the tree individuals (median = 91.0%; 95% confidence intervals = 87.3-90.5%; range = 62-100%) across 135 surveys of São Paulo state forests. Because these sites had on average 1.5% (median = 0.6%; range = 0-11%) of their individual unidentified at species level, this proportion may be underestimated. When considered at genus and family levels, this proportion reaches 99.0% (median = 99.7%; range = 90.1-100%) and 99.5% (median = 99.9%; range = 93.1-100%), respectively.

### Geographical range and DNA barcode availability

The SP tree species list contains 237 local endemics (11%) and 493 regional endemics (24%). The availability of DNA barcoding data was strongly related to species geographical distribution (Likelihood ratio test: 409.5, d.f. = 2093, *p* < 0.0001). Widespread species had on average more barcodes available than Eastern South-American, regional and local endemic species (in decreasing order of number of barcoding sequences per species). Regional and local endemics also presented significantly less *rbc*L and *mat*K sequences (12.9%) than other species (43.5% - Likelihood ratio test: 227.19, d.f. = 2093, *p* < 0.0001). The same outcome was found when analysis was conducted considering only the top five DNA barcodes (Likelihood ratio test: 419.9, d.f. = 2093, *p* < 0.0001).

## Discussion

We investigated the completeness of the DNA barcode reference library for a tropical region, the São Paulo State, Brazil, and found that the current library encompasses only 58% of the tree species. Although there are thousands of barcoding sequences already available, a complete library of DNA barcodes for the São Paulo tree flora is still far from complete. In this study, we made an important contribution in this direction by directed sequencing of more rare genera and families. However, our results suggest that at least two more decades will be necessary to achieve 100% of species with DNA barcodes available.

In species-rich forests, the identification of all individuals in a given community is often a difficult and subjective task (Gonzalez *et al.*, 2009; Gomes *et al.*, 2013). Approaches that can help ecologists obtaining more accurate and consistent identifications would be very handy. In this study, we found that 89% of the individuals in a typical tree survey belong to species that are already available in the DNA barcoding reference library. Therefore, many conservation-oriented and ecological studies can already benefit from DNA barcoding. Because many species occurring in São Paulo are not restricted to this state, community studies conducted in forests of the Atlantic Forest and Cerrado domains will also benefit from this reference DNA library. Preliminary data from other 580 forest surveys outside São Paulo borders (http://labtrop.ib.usp.br/doku.php?id=projetos:treeco:star) suggest that 71-88% of the individuals from Atlantic and Cerrado forests in other regions of Brazil, Paraguay and Argentina already have sequences in the current DNA barcoding library. The exception is Northeast Brazil where this percentage is only 41%.

Defining which taxa should be prioritized in future sequencing efforts is not easy. Here, we defined a family-level priority scale based on the proportion of species without barcodes per family and on how many of them were threatened or endemic. This definition was first thought in the context of using DNA barcoding in assessments of conservation status in biodiversity surveys (Feest, 2006). However, one could argue that priority should be given to the taxa that are harder to identify by morphological characters. In São Paulo state, these taxa would certainly include several genera within the Myrtaceae, Lauraceae, Rubiaceae, Fabaceae and Melastomataceae (Caiafa and Martins, 2007). It would be hard to find an objective criterion based on the difficulty of species identification. These families are all species-rich and include several threatened and endemic tree species, so it would be important to prioritize them in future DNA barcoding efforts. Therefore, directed sampling aimed at the families listed here as high priority (i.e., *P* > 1 in Table S3) would therefore provide many sequences for taxa with high conservation relevance. As shown here, trees endemic to SP were indeed the ones with fewer barcoding sequences.

Having barcodes for all species does not necessarily guarantee a precise identification at species level. Within some genera, species cannot be resolved using only core DNA barcodes (i.e. *rbc*L and *mat*K), and many taxa will need the combination of two or more barcodes to guarantee accurate identifications (Chase *et al.*, 2007; Gonzalez *et al.*, 2009; Kress *et al.*, 2009). Currently, there is no published assessment of barcode efficiency for the SP tree flora. Published assessments of barcode efficiency and phylogenetic studies have suggested the ITS region and *psb*A*-trn*H as promising barcodes for Atlantic Forest species of Sapotaceae, Lauraceae, the tribe Myrteae, *Senna* and *Casearia* (Lucas *et al.*, 2007; Vivas *et al.*, 2014; Bolson *et al.*, 2015; Januario BB, 2014, MSc. Thesis Universidade Estadual Paulista, São Paulo). These two markers are already among the top five barcodes for the SP tree flora, along with *rbc*L and *mat*K. An interesting strategy would be to have *rbc*L and *mat*K sequenced for 100% of families and genera of the SP tree flora, which would require sequencing another 100 genera (see Table S5). Complementarily, the ITS and *psb*A*-trn*H regions could be amplified in selected clades that present low discrimination rates using only *rbc*L and *mat*K (Lucas *et al.*, 2007; Vivas *et al.*, 2014; Bolson *et al.*, 2015). The *trn*L intron would be another important marker to increase focus on because a short region on this marker (the P6 loop) is often recommended for plant diversity surveys based on degraded DNA from environmental samples (Yoccoz *et al.*, 2012).

New sequencing technologies are becoming rapidly available and these have greatly facilitated the sequencing of complete plastid genomes in plants (Taberlet *et al.*, 2012; Coissac *et al.*, 2016). Full plastid genomes are now technically within reach and will represent a considerable advance in the field of DNA-based species identification (Kane *et al.*, 2012; Li *et al.*, 2015). Plastid genome libraries may take time to be completed in developing, species-rich countries, but they would certainly offer a much larger base of knowledge than the marker-based efforts, such as the one described here (Kress *et al.*, 2015; Coissac *et al.*, 2016). Another promising approach relies on near infrared spectroscopy (Rodríguez-Fernández *et al.*, 2011), a technique that could be more thoroughly explored for the identification of land plants, although it cannot be used to assess the phylogenetic relationship among species.

In the past five years, it seems that DNA barcoding has fallen out of interest, probably due to the exposition of its limitations (Krishnamurthy and Francis, 2012; Taylor and Harris, 2012), or to the rise of new approaches that rely on next-generation sequencing (Taberlet *et al.*, 2012; Li *et al.*, 2015). Today, it is well known that classic DNA barcoding will not accurately discriminate 100% of species, be it within a specific taxonomic group (Seberg and Petersen, 2009; Federici *et al.*, 2013), or in communities with many closely-related taxa (Pei *et al.*, 2015). However, DNA barcoding is still valid to identify target species (e.g., endangered species), or to provide more accurate species lists in the study and monitoring of ecological communities (Yu *et al.*, 2012; Joly *et al.*, 2014; Fahner *et al.*, 2016), especially when species identification is based on seeds, pollen, wood, roots and juvenile individuals. So far, we are unaware of conservation initiatives or ecological studies carried out in São Paulo State that are based on DNA-barcoding. Bridging this gap will critically rely on coordinate efforts to train conservationists and ecologists to popularize DNA sequencing. Although representing a challenge in itself, we have shown here that building a useful reference library of DNA barcodes in species-rich tropical regions is possible. We hope that this study will encourage colleagues from other tropical countries to engage in this effort.

## Supplementary Material

The following online material is available for this article:

Table S1 - Tree community surveys used to obtain species abundances for the São Paulo tree flora.

Table S2 - Primers and PCR protocols used to obtain the DNA sequences.

Table S3 - The priority index for future sequencing efforts for tree species in the state of São Paulo, Brazil.

Table S4 - The full list of the São Paulo tree flora, including their life form, geographical distribution, threat status at state-level and the source of the record.

Table S5 - List of GenBank accessions found for the São Paulo tree flora.

Table S6 - Differences between the proportion of taxa in the SP tree flora and the proportion of taxa with barcoding sequences with DNA barcodes per family and genus.
