# Supplementary material for: Can plant DNA barcoding be implemented in species-rich tropical regions? A perspective from São Paulo State, Brazil
Source: Genet Mol Biol. 2018 Jul-Sep;41(3):661–70. doi: 10.1590/1678-4685-GMB-2017-0282 (PMC6136365; doi:10.1590/1678-4685-GMB-2017-0282)
Supplement: Supplementary file 1 [file 1415-4757-GMB-41-03-2017-0282-20180723-suppl1.pdf]

Supplementary Material to “Can plant DNA barcoding be implemented in species-rich tropical regions? A perspective from São Paulo State, Brazil”

Table S1 - Tree community surveys used to obtain species abundances for the São Paulo tree flora. For each survey, we provide the county and locality where the survey was conducted, along with the geographical coordinates (in decimal degrees), the sampling method (i.e. plots or point-centered quarter method - PCQ) and the diameter at breast height (dbh) cutoff criteria adopted. For dbh, the symbol '*' means that surveys were originally conducted using a different cutoff criteria, but data was filtered for the marked cutoff. The two columns related to the study effort are Area (total area sampled) and *N* (i.e. number of individuals). Finally, RefID is the identification number of the study related to each given survey, for which full references are provided after the table.

County, Locality	Latitude	Longitude	Method	dbh (cm)	Area (ha)	*N*	RefID
Agudos, Faz. Santa Rita	-22.4865	-49.058617	Plots	5.0	0.88	1200	12
Angatuba, EEx de Angatuba	-23.395906	-48.362369	Plots	5.0	0.50	1057	31
Anhembi, Faz. Barreiro Rico	-22.657802	-48.158864	PCQ	5.0*	0.60	729	20
Araras, Usina Santa Lúcia	-22.298718	-47.421032	Plots	3.0	0.50	728	83
Assis, EEc de Assis	-22.595556	-50.378056	Plots	5.0	10.24	21928	77
Assis, EEc de Assis	-22.602533	-50.383412	Plots	5.0	5.76	10564	59
Assis, EEc de Assis	-22.594748	-50.381627	Plots	5.0	1.00	1779	69
Assis, EEc de Assis	-22.593736	-50.36336	Plots	5.0	0.30	638	27
Atibaia, PM da Grota Funda	-23.179415	-46.531134	Plots	5.0	0.42	1026	39

County, Locality	Latitude	Longitude	Method	dbh (cm)	Area (ha)	*N*	RefID
Bananal, EEc Bananal	-22.806676	-44.36611	Plots	5.0	0.86	1702	74
Barra do Chapéu, Faz. Sra. Pedrina Demetrio	-24.46513	-49.018285	Plots	5.0	1.00	1881	85
Barra do Turvo, PE da Caverna do Diabo/Quilombos	-24.643302	-48.401438	Plots	5.0	0.80	966	15
Barretos, Faz. Floresta	-20.640253	-48.753521	Plots	5.0	1.00	571	73
Barretos, Faz. Vista Bonita	-20.485	-48.8225	Plots	5.0	1.00	907	73
Bauru, EEc Sebastião Aleixo da Silva	-22.231548	-49.081931	Plots	5.0	3.20	3838	96
Bauru, Campus UNESP	-22.344064	-49.021869	Plots	5.0	0.27	1568	14
Bauru, Parque Ecológico de Bauru	-22.337873	-48.998166	Plots	5.0*	1.00	3267	18
Bebedouro, Faz. Córrego dos Bois	-20.885000	-48.540560	Plots	5.0	1.00	699	73
Bofete, Morro Grande	-23.038889	-48.277222	Plots	5.0	0.64	687	60
Bofete, Faz. Santa Terezinha	-23.047222	-48.191389	PCQ	5.0	0.43	738	100
Botucatu, Faz. Experimental Edgardia	-22.814682	-48.394047	Plots	5.0	1.00	1104	32
Botucatu, Faz. Goldfarm	-22.737222	-48.306667	Plots	5.0*	0.50	900	11
Brotas, Horto Santa Fé	-22.264880	-48.038045	Plots	3.0*	1.00	3787	36
Brotas, Margem do Rio Jacaré-Pepira	-22.256841	-48.088728	Plots	5.0	0.36	735	56
Brotas, Faz. Santa Maria da Fábrica	-22.160899	-47.91976	Plots	5.0	0.20	498	22
Brotas, EEc de São Carlos	-22.081921	-48.026126	Plots	5.0	0.44	663	30
Caconde, PM Prainha	-21.567100	-46.608100	Plots	5.0	0.78	926	26
Campinas, RM Santa Genebra	-22.821541	-47.109603	Plots	5.0	1.00	1081	29
Campinas, Centro Experimental de Campinas (IAC)	-22.862924	-47.068666	Plots	5.0	0.50	422	68

County, Locality	Latitude	Longitude	Method	dbh (cm)	Area (ha)	*N*	RefID
Campinas, Margem do Ribeirão Cachoeira	-22.833722	-46.923818	Plots	5.0	1.00	1080	21
Campinas, Faz. São Vicente	-22.825058	-46.986549	Plots	5.0	0.42	596	9
Campinas, RM Santa Genebra	-22.824917	-47.111731	Plots	5.0	1.05	1067	35
Campos do Jordão, PE Campos do Jordão	-22.691958	-45.46331	Plots	5.0	1.00	1760	86
Campos do Jordão, PE Campos do Jordão	-22.701311	-45.478116	Plots	5.0	1.50	2702	51
Cananéia, Faz. Folha Larga	-24.887337	-47.933022	Plots	5.0	1.20	2311	98
Cananéia, parcelas D e E	-24.906754	-47.915292	Plots	5.0	0.40	577	98
Cananéia, parcela F	-24.913879	-47.902968	Plots	5.0	0.20	526	98
Cananéia, PE da Ilha do Cardoso	-25.076861	-47.934047	Plots	5.0*	10.24	15276	64
Cananéia, PE da Ilha do Cardoso	-25.106607	-47.933817	Plots	2.5	1.00	2510	61
Caraguatatuba, PE Serra do Mar (Núcleo Caraguatatuba)	-23.634877	-45.53154	Plots	5.0	1.00	2165	87
Caraguatatuba, UTGCA Petrobras	-23.640942	-45.450098	Plots	5.0	1.60	2599	67
Caraguatatuba, PE Serra do Mar (Núcleo Caraguatatuba)	-23.588799	-45.545569	Plots	5.0	1.00	1735	87
Cotia, RF do Morro Grande	-23.68697	-46.963813	PCQ	5.0	1.80	2394	17
Cristais Paulista, Faz. Tamboril	-20.42300	-47.43400	Plots	3.0	0.60	2036	92
Cruzeiro, NA	-22.64205	-44.976387	Plots	5.0	0.60	864	58
Gália, EEc de Caetetus	-22.411236	-49.701147	Plots	5.0	10.24	12105	77
Gália, EEc de Caetetus	-22.418195	-49.70147	Plots	5.0	5.76	7839	75
Gália, EEc de Caetetus	-22.394397	-49.708334	Plots	5.0	5.76	8884	23
Gália, EEc de Caetetus	-22.381833	-49.683361	Plots	5.0	1.00	1218	33

County, Locality	Latitude	Longitude	Method	dbh (cm)	Area (ha)	*N*	RefID
Gália, EEc de Caetetus	-22.408783	-49.688616	Plots	5.0	1.00	1111	33
Gália, EEc de Caetetus	-22.413951	-49.685434	Plots	5.0	0.60	644	28
Guaiçara, Margem do Córrego Canjarana	-21.493611	-49.683333	Plots	3.0	0.54	698	55
Iguape, EEc de Juréia-Itatins	-24.549215	-47.237847	Plots	5.0	1.00	1826	62
Iguape, EEc Chauás	-24.75030	-47.67440	Plots	5.0	0.24	640	99
Iperó, FLONA de Ipanema	-23.432599	-47.623588	Plots	5.0	1.12	1659	4
Ipeúna, Margem do Rio Passa Cinco	-22.415345	-47.721869	Plots	5.0	0.79	1417	10
Itatiba, Sítio Jatobá	-23.02193	-46.735042	PCQ	5.0	0.76	1470	19
Itatinga, Faz. Santa Irene	-23.29502	-48.648143	Plots	5.0	1.00	1242	44
Itatinga, Faz. Santa Irene	-23.277858	-48.557744	Plots	5.0	0.42	845	45
Itatinga, Faz. Santa Irene	-23.316667	-48.616667	PCQ	5.0	0.28	530	42
Itirapina, EEc de Itirapina	-22.24038	-47.87355	Plots	3.0	0.60	5124	93
Jaboticabal, campus UNESP	-21.247891	-48.268177	Plots	5.0	1.28	1694	70
Jaú, Faz. Santo Antônio	-22.317187	-48.509824	PCQ	5.0*	0.57	808	63
Joanópolis, Serra do Lopo	-22.90315	-46.339192	Plots	5.0	0.30	494	101
Luiz Antônio, EEc Jataí	-21.596752	-47.813902	Plots	5.0	1.00	1413	97
Macaubal, Faz. Pauã	-20.74300	-49.92917	Plots	5.0	1.00	1689	73
Mairiporã, PE da Cantareira (Núcleo Águas Claras)	-23.417031	-46.637861	Plots	5.0	1.68	2384	7
Matão, Faz. Cambuhy	-21.602923	-48.514606	Plots	5.0	1.62	2045	78
Matão, Faz. Cambuhy	-21.620556	-48.537222	Plots	5.0	1.00	1175	73

County, Locality	Latitude	Longitude	Method	dbh (cm)	Area (ha)	*N*	RefID
Mogi das Cruzes, PM da Serra do Itapety	-23.498413	-46.196521	PCQ	5.0	0.99	1440	95
Mogi Guaçu, Margem do Rio Mogi Guaçu	-22.374362	-46.900437	Plots	5.0	1.00	1470	48
Nova Granada, Faz. São João	-20.553108	-49.248754	Plots	5.0	1.00	1035	73
Novo Horizonte, Faz. Serrinha	-21.52100	-49.29472	Plots	5.0	1.00	782	73
Palestina, Faz. Boa Vista	-20.292905	-49.506943	Plots	5.0	1.00	1679	73
Palestina, Faz. Boa Vista	-20.323194	-49.508136	Plots	5.0	1.00	704	73
Pariquera-Açu, Antonio Povinske	-24.676036	-47.880773	Plots	5.0	1.21	1956	43
Pariquera-Açu, PE da Campina do Encantado	-24.641688	-47.783123	Plots	5.0	0.54	768	88
Pariquera-Açu, PE da Campina do Encantado	-24.640794	-47.786224	Plots	5.0	0.30	519	88
Pariquera-Açu, PE da Campina do Encantado	-24.63858	-47.780572	Plots	5.0	0.20	546	88
Paulo de Faria, EEc de Paulo de Faria	-19.95730	-49.535873	Plots	5.0*	1.00	1278	94
Peruíbe, EEc de Juréia-Itatins	-24.345705	-47.009199	Plots	5.0*	0.25	247	65
Pindamonhangaba, REc do Trabiju	-22.8000	-45.533333	Plots	5.0	0.25	517	37
Pindorama, RB Pindorama	-21.213705	-48.888526	Plots	5.0	0.65	572	1
Pindorama, Estação Experimental	-21.227046	-48.931437	Plots	5.0	1.00	769	73
Piracaia, Reservatório Cachoeira	-23.0075	-46.266944	Plots	5.0	0.48	875	5
Piracicaba, Bairro dos Godinhos	-22.6558	-47.6534	Plots	5.0	0.43	894	41
Piracicaba, Campus ESALQ (Mata da Pedreira)	-22.703762	-47.629498	Plots	5.0	0.40	532	16
Piracicaba, Campus ESALQ (Mata do Pomar)	-22.712438	-47.62426	Plots	5.0	0.60	535	89
Planalto, Faz. Taperão	-20.999442	-49.980144	Plots	5.0	1.00	2209	73

County, Locality	Latitude	Longitude	Method	dbh (cm)	Area (ha)	*N*	RefID
Ribeirão Preto, EEc Ribeirão Preto	-21.2222	-47.8481	PCQ	5.0	0.96	1392	90
Ribeirão Preto, EEc Ribeirão Preto	-21.224153	-47.850095	Plots	5.0*	0.52	503	3
Rio Claro, Serra do Itaqueri	-22.386866	-47.843863	Plots	5.0	1.29	873	47
Rio Claro, Faz. São José	-22.357523	-47.47442	Plots	5.0	0.45	1651	91
Rio Claro, Faz. São José	-22.359169	-47.480177	Plots	5.0*	0.43	783	52
Sales, Faz. Águas Claras	-21.403651	-49.493986	Plots	5.0	1.00	877	73
Sta. Rita do Passa Quatro, PE de Vassununga	-21.721158	-47.596855	PCQ	5.0	0.39	926	57
Sto. André, PNM Nascentes de Paranapiacaba	-23.7689	-46.28580	Plots	5.0	1.00	1991	50
Sto. Ant. do Aracanguá, Faz. Rancho Alegre	-20.9260	-50.34861	Plots	5.0	1.00	1415	73
São Carlos, Faz. Canchim	-21.964232	-47.843599	Plots	5.0	1.00	1216	82
São João de Iracema, Faz. São Francisco	-20.50964	-50.230988	Plots	5.0	1.00	795	73
São José do Barreiro, Frag Atibaia	-22.647706	-44.658458	PCQ	5.0	0.37	527	34
São José do Barreiro, Frag Mascote	-22.63517	-44.657175	PCQ	5.0	0.34	523	34
São José do Barreiro, Faz. São Domingos	-22.646805	-44.72343	PCQ	5.0	0.31	517	34
São José dos Campos, RF Professor Augusto Ruschi	-23.065702	-45.933914	Plots	5.0	0.70	1415	81
São Luiz do Paraitinga, PE Serra do Mar (Núcleo Sta. Virgínia)	-23.325278	-45.068611	Plots	5.0	1.00	1852	66
São Luiz do Paraitinga, PE Serra do Mar (Núcleo Sta. Virgínia)	-23.343333	-45.072778	Plots	5.0	1.00	1431	66
São Miguel Arcanjo, PE Carlos Botelho	-24.068723	-47.971617	Plots	5.0	5.76	10184	2
São Paulo, PNM Cratera de Colônia	-23.881151	-46.70529	Plots	3.0	1.20	1577	54
São Paulo, PE das Fontes do Ipiranga	-23.644382	-46.620095	PCQ	5.0	0.31	500	24

County, Locality	Latitude	Longitude	Method	dbh (cm)	Area (ha)	*N*	RefID
São Paulo, PE das Fontes do Ipiranga	-23.639913	-46.616247	PCQ	5.0	0.34	508	24
São Paulo, SESC Interlagos	-23.729205	-46.679032	Plots	5.0	0.50	830	53
São Roque, PM de São Roque	-23.526126	-47.104079	Plots	5.0	0.95	1324	49
Sete Barras, PE Carlos Botelho	-24.181361	-47.92500	Plots	5.0	10.24	11529	77
Sete Barras, PE Carlos Botelho	-24.166173	-47.972686	Plots	5.0	5.76	10352	25
Sete Barras, PE de Intervales	-24.233333	-48.066667	Plots	5.0	1.98	3078	40
Sete Barras, PE Intervales Sete Barras (Saibadela)	-24.232154	-48.082059	PCQ	5.0	0.52	781	6
Taquaritinga, Faz. Santa Lúcia	-21.404528	-48.689824	Plots	5.0	1.00	566	73
Teodoro Sampaio, PE do Morro do Diabo	-22.587019	-52.338032	PCQ	5.0	1.08	1143	80
Turmalina, Faz. São João	-20.00400	-50.43389	Plots	5.0	1.00	963	73
Ubatuba, PE Serra do Mar (Núcleo Picinguaba)	-23.330584	-44.828931	Plots	5.0	0.40	629	79
Ubatuba, PE Serra do Mar (Núcleo Picinguaba)	-23.358326	-44.85182	Plots	5.0	1.00	1892	79
Ubatuba, PE Serra do Mar (Núcleo Picinguaba)	-23.329033	-44.830086	Plots	5.0	0.40	563	79
Ubatuba, PE Serra do Mar (Núcleo Picinguaba)	-23.356232	-44.851572	Plots	5.0	1.00	1670	8
Ubatuba, PE Serra do Mar (Núcleo Picinguaba)	-23.336944	-44.835556	Plots	5.0	1.00	1076	8
Ubatuba, PE Serra do Mar (Núcleo Picinguaba)	-23.336182	-44.83494	Plots	5.0	1.00	1119	46
Ubatuba, PE Serra do Mar (Núcleo Picinguaba)	-23.335833	-44.833333	Plots	5.0	1.00	1281	71
Ubatuba, PE Serra do Mar (Núcleo Picinguaba)	-23.334167	-44.832222	Plots	5.0	1.00	1240	13
Ubatuba, Faz. Capricornio	-23.381389	-45.078889	Plots	5.0	1.00	1379	72
Ubatuba, PE Serra do Mar (Núcleo Picinguaba)	-23.373889	-45.080833	Plots	5.0	1.00	1496	38

County, Locality	Latitude	Longitude	Method	dbh (cm)	Area (ha)	*N*	RefID
Ubatuba, Faz. Capricornio	-23.381111	-45.078611	Plots	5.0	1.00	1528	72
Ubatuba, PE Serra do Mar (Núcleo Picinguaba)	-23.366944	-45.083611	Plots	5.0	1.00	1995	38
Ubatuba, Faz. Capricornio	-23.366389	-45.083889	Plots	5.0	1.00	1816	76
Ubatuba, PE Serra do Mar (Núcleo Santa Virgínia)	-23.360028	-45.109778	Plots	5.0	1.00	1728	84
União Paulista, Faz. Boa Vista	-20.921111	-49.926111	Plots	5.0	1.00	981	73
Votuporanga, Faz. Primavera	-20.513167	-50.090186	Plots	5.0	1.00	1532	73

References (RefID)

1. Abdo MTV (2009) Caracterização da vegetação arbórea e atributos do solo da Reserva Biológica de Pindorama, SP. D.Sc. Thesis, Universidade Estadual Paulista, Jaboticabal, 64 p.
2. Aguiar OT (2003) Comparação entre os métodos de quadrantes e parcelas na caracterização da composição florística e fitossociológica de um trecho de floresta ombrófila densa no Parque Estadual “Carlos Botelho” – São Miguel Arcanjo, São Paulo. M.Sc. Thesis, Universidade de São Paulo, Piracicaba, 140 p.
3. Aita L (1997) Florística e fitossociologia de uma floresta mesófila semi-caducifólia, Estação Ecológica de Ribeirão Preto - Mata Santa Teresa, SP. Etapa II. B.Sc. Thesis, Universidade de São Paulo, Ribeirão Preto, 65 p.
4. Albuquerque GB and Rodrigues RR (2000) A vegetação do Morro de Araçoiaba, Floresta Nacional de Ipanema, Iperó (SP). Sci For 58:145-159.
5. Almeida A, Padovezi A and Lima RAF (2011) Levantamento e avaliação de informações de referência para o Projeto 'Restauração de 350 ha do entorno do reservatório do Rio Cachoeira - Piracaia - SP. The Nature Conservancy do Brasil, Brasília, 108 p.
6. Almeida-Scabbia RJ (1996) Fitossociologia de um trecho de floresta atlântica no Parque Estadual Intervales, SP. M.Sc. Thesis, Universidade Estadual Paulista, Rio Claro, 121 p.
7. Arzolla FARDP (2002) Florística e fitossociologia de trecho da Serra da Cantareira, Núcleo Águas Claras, Parque Estadual da Cantareira, Mairiporã, SP. M.Sc. Thesis, Universidade Estadual de Campinas, Campinas, 184 p.
8. Assis MA, Prata EMB, Pedroni F, Sanchez M, Eisenlohr PV, Martins FR, Santos FAM, Tamashiro JY, Alves LF, Vieira AS, *et. al.* (2011) Florestas de restinga e de terras baixas na planície costeira do sudeste do Brasil: vegetação e heterogeneidade ambiental. Biota Neotropica 11:83-101.
9. Bernacci LC (1992) Estudo florístico e fitossociológico de uma floresta no município de Campinas, com ênfase nos componentes herbáceo e arbustivo. M.Sc. Thesis, Universidade Estadual de Campinas, Campinas, 146 p.
10. Bertani DF, Rodrigues RR, Batista JLF and Shepherd GJ (2001) Análise temporal da heterogeneidade florística e estrutural em uma floresta ribeirinha. Rev Bras Bot 24:11-23.
11. Bicudo LRH (1995) Florística, fitossociologia e ciclagem de nutrientes em um cerradão no município de Botucatu - SP. D.Sc. Thesis, Instituto de Biociências, Universidade Estadual Paulista Júlio de Mesquita Filho, Rio Claro, 167 p.
12. Camargo PFA (1999) Composição florística e estrutural fitossociológica de um remanescente de floresta estacional semidecidual submontana na Fazenda Santa Rita, no Município de Agudos – SP. M.Sc. Thesis, Instituto de Biociências, Universidade Estadual Paulista Júlio de Mesquita Filho, Botucatu, 118 p.
13. Campos MCR, Tamashiro JY, Assis MA and Joly CA (2011) Florística e fitossociologia do componente arbóreo da transição Floresta Ombrófila Densa das Terras Baixas - Floresta Ombrófila Densa Submontana do Núcleo Picinguaba/PESM, Ubatuba, sudeste do Brasil. Biota Neotropica 11:301-312.
14. Carboni M (2007) Composição, estrutura e diversidade vegetal de uma floresta estacional semidecídua ribeirinha com influência fluvial permanente (mata de brejo) em Bauru – SP. M.Sc. Thesis, Universidade Estadual Paulista, Botucatu, 116 p.
15. Cardoso-Leite E, Podadera DS, Peres JC and Castello ACD (2013) Analysis of floristic composition and structure as an aid to monitoring protected areas of dense rain forest in southeastern Brazil. Acta Bot Brasilica 27:180-194
16. Catharino ELM (1989) Estudo fisionômico-florístico e fitossociológico em matas residuais secundárias no município de Piracicaba, SP. M.Sc. Thesis, Universidade Estadual de Campinas, Campinas, 189 p.
17. Catharino ELM, Bernacci LC, Franco GADC, Durigan G and Metzger JP (2006) Aspectos da composição e diversidade do componente arbóreo das florestas da Reserva Florestal do Morro Grande, Cotia, SP. Biota Neotropica 6:1-28.
18. Cavassan O (1990) Florística e fitossociologia da vegetação lenhosa de um hectare de cerrado no Parque Ecológico Municipal de Bauru - SP. D.Sc. Thesis, Universidade Estadual de Campinas, Campinas, 206 p.

19. Cerqueira RM (2005) Florística e estrutura de um fragmento de floresta estacional semidecídua montana no município de Itatiba, SP. M.Sc. Thesis, Universidade de Campinas, Campinas, 106 p.
20. César O and Leitão-Filho HF (1990b) Estudo fitossociológico de mata mesófila semidecídua na Fazenda Barreiro Rico, município de Anhembi, SP. Rev Bras Biol 50:443-452.
21. Cielo-Filho R, Gneri M and Martins F (2011) Sampling precision and variability of tree species abundance ranks in a semideciduous Atlantic forest fragment. Community Ecol 12:188-195.
22. Costa FRC, Schlittler FHM, César O and Monteiro R (1997) Aspectos florísticos e fitossociológicos de um remanescente de brejo no Município de Brotas, SP. Braz Arch Biol Technol 40:263-270.
23. Couto HTZ (2005) Métodos de Inventário da Biodiversidade de Espécies Arbóreas. Escola Superior de Agricultura Luiz de Queiroz, Universidade de São Paulo, Piracicaba, 115 p
24. De Vuono YS (1985) Fitossociologia do estrato arbóreo da floresta da Reserva Biológica do Instituto de Botânica (São Paulo, SP). D.Sc. Thesis, Universidade de São Paulo, São Paulo, 213 p.
25. Dias AC (2005) Composição florística, fitossociológica, diversidade de espécies arbóreas e comparação de métodos de amostragem na floresta ombrófila densa do Parque Estadual de Carlos Botelho/SP, Brasil. D.Sc. Thesis, Universidade de São Paulo, Piracicaba, 203 p.
26. Dias G (2010) Florística e Fitossociologia das espécies arbóreas de ocorrência em mata ciliar no Alto Rio Pardo, na Estância Climática de Caconde, SP. M.Sc. Thesis, Universidade Estadual Paulista Júlio de Mesquita Filho, Rio Claro, 71 p.
27. Durigan G and Leitão-Filho HF (1995) Floristica e fitossociologia de matas ciliares do oeste paulista. Revista do Instituto Florestal 7:197-239.
28. Durigan G, Franco GADC, Saito M and Baitello JB (2000) Estrutura e diversidade do componente arbóreo da floresta na Estação Ecológica dos Caetetus, Gália, SP. Rev Bras Bot 23:371-383.
29. Farah FT (2009) Vinte anos de dinâmica em um hectare de Floresta Estacional Semidecidual. D.Sc. Thesis, Universidade Estadual de Campinas, Campinas, 130 p.
30. Feliciano ALP (1999) Caracterização ambiental, florística e fitossociológica de uma Unidade de Conservação. Estação Ecológica de São Carlos, Brotas, 160 p.
31. Figueiredo N (1993) Estudo fitossociologico em uma floresta mesofila semidecidua secundaria na Estação Experimental de Angatuba, municipio de Angatuba, SP. M.Sc. Thesis, Universidade Estadual de Campinas, Campinas, 160 p.
32. Fonseca RCB and Rodrigues RR (2000) Análise estrutural e aspectos do mosaico sucessional de uma floresta semidecídua em Botucatu, SP. Sci For 57:27-43.
33. Franco GADC (2002) Florística e fitossociologia de duas unidades do mosaico florestal da Estação Ecológica dos Caetetus - Floresta Estacional Semidecidual, Gália - SP. M.Sc. Thesis, Escola Superior de Agricultura Luiz de Queiroz, Universidade de São Paulo, Piracicaba, 95 p.
34. Freitas HS (2010) Caracterização florística e estrutural de três fragmentos de floresta estacional semidecidual da região leste do Vale do Paraíba – SP. D.Sc. Thesis, Universidade de São Paulo, São Paulo, 324 p.
35. Gandolfi S (2000) História Natural de uma floresta estacional semidecidual no município de Campinas (São Paulo, Brasil). D.Sc. Thesis, Universidade de Campinas, Campinas, 520 p.
36. Gomes BZ, Martins FR and Tamashiro JY (2004) Estrutura do cerradão e da transição entre cerradão e floresta paludícola num fragmento a International Paper do Brasil Ltda., em Brotas, SP. Rev Bras Bot 27:249-262.
37. Gomes EPC, Fisch STV and Mantovani W (2005) Estrutura e composição do componente arbóreo na Reserva Ecológica do Trabiju, Pindamonhangaba, SP, Brasil. Acta Bot Brasilica 19:451-464.
38. Gomes JAMA, Bernacci LC and Joly CA (2011) Diferenças florísticas e estruturais entre duas cotas altiduninais da Floresta Ombrófila Densa Submontana Atlântica, do Parque Estadual da Serra do Mar, município de Ubatuba/SP, Brasil. Biota Neotropica 11:123-137.
39. Grombone-Guaratini MT, Bernacci LC, Meira-Neto JA, Tamashiro JY and Leitão-Filho HF (1990) Estrutura fitossociológica da floresta semidecídua de altitude do Parque Municipal da Grota Funda (Atibaia – estado de São Paulo). Acta Bot Brasilica 4:47-64.
40. Guilherme FAG, Morellato LP and Assis MA (2004) Horizontal and vertical tree community structure in a lowland Atlantic Rain Forest, Southeastern Brazil. Ver Bras Bot 27:725-737.

41. Ivanauskas NM and Rodrigues RR (2000) Florística e fitossociologia de remanescentes de Floresta Estacional Decidual em Piracicaba, São Paulo, Brasil. Rev Bras Bot 23:291-304.
42. Ivanauskas NM and Rodrigues RR (2002) Fitossociologia de um remanescente de Floresta Estacional Semidecidual em Itatinga, SP, para fins de restauração de áreas degradadas. Revista Árvore 26:43-57.
43. Ivanauskas NM (1997) Caracterização florística e fisionomia da Floresta Atlântica sobre a formação Pariquera-Açu, na Zona da Morraria Costeira do estado de São Paulo. M.Sc. Thesis, Universidade Estadual de Campinas, Campinas, 231 p.
44. Ivanauskas NM, Rodrigues RR and Nave AG (1997) Aspectos ecológicos de um trecho de floresta de brejo em Itatinga, SP: florística, fitossociologia e seletividade de espécies. Rev Bras Bot 20:139-153.
45. Ivanauskas NM, Rodrigues RR and Nave AG (1999) Fitossociologia de um trecho de Floresta Estacional Semidecidual em Itatinga, São Paulo, Brasil. Sci For 56: 83-99.
46. Kamimura VA (2011) Aspectos estruturais e ecológicos de uma comunidade arbórea do Parque estadual da Serra do Mar. B.Sc. Thesis, Universidade Estadual Paulista Júlio de Mesquita Filho, Rio Claro, 45 p.
47. Kotchetkoff-Henriques O and JOLY CA (1994) Estudo florístico e fitossociológico em uma mata mesófila na Serra do Itaqueri, Itirapina, estado de São Paulo. Rev Brasa Biol 54:477-487.
48. Leitão-Filho HF, Azevedo DB, Santin DA, Gardolinski PCFC and Rodrigues RR (1994) Estudos de ecologia da mata ciliar dos rios Mogi Guaçu e Peixe-UHE, Mogi Guaçu - SP. Universidade de Campinas, Campinas, 95 p.
49. Leite EC and Rodrigues RR 2008. Fitossociologia e caracterização sucessional de um fragmento de floresta estacional no Sudeste do Brasil. Revista Árvore 32:583-595.
50. Lima MEL, Cordeiro I and Moreno PRH (2011) Estrutura do componente arbóreo em Floresta Ombrófila Densa Montana no Parque Natural Municipal Nascentes de Paranapiacaba (PNMNP), Santo André, SP, Brasil. Hoehnea 38:73-96.
51. Los MM (2004) Florística, estrutura e diversidade em floresta com Araucária em áreas de diferentes tamanhos. M.Sc. Thesis, Universidade de São Paulo, São Paulo, 79 p.
52. Manzatto AG (2005) Dinâmica da comunidade arbustivo-arbórea em um fragmento de floresta estacional semidecidual localizada no município de Rio Claro, SP, durante o período de 1989-2003. D.Sc. Thesis, Universidade Estadual Paulista, Rio Claro, 114 p.
53. Manzatto AG, Furlan A, César O and Pagano SN (1999) Vegetação lenhosa do SESC Interlagos, São Paulo, SP. Instituto de Biociências, Universidade Estadual Paulista Júlio de Mesquita Filho, Rio Claro, 74 p.
54. Marçon SL (2009) Composição florística e estrutura do componente arbustivo-arbóreo do Parque Natural Municipal da Cratera da Colônia, São Paulo, SP. M.Sc. Thesis, Universidade de São Paulo, Ribeirão Preto, 120 p.
55. Mardegan CM and Cavassan O (2009) Considerações sobre as alterações de um fragmento florestal às margens de uma represa no município de Guaiçara, SP, a partir da análise florística e fitossociológica. Revista do Instituto Florestal de São Paulo 21:139-150.
56. Marques MCM, Silva SM and Salino A (2003) Florística e estrutura do componente arbustivo-arbóreo de uma floresta higrófila da bacia do rio Jacaré-Pepira, SP, Brasil. Acta Bot Brasilica 17:495-506.
57. Martins FR (1991) Estrutura de uma floresta mesófila. Universidade Estadual de Campinas, Campinas, 214 p.
58. Martins SV, Coutinho MP and Marangon LC (2002) Composição florística e estrutura de uma floresta secundária no município de Cruzeiro-SP. Revista Árvore 26:35-41.
59. Medeiros DA (2004) Métodos de amostragem no levantamento da diversidade do cerradão da Estação Ecológica de Assis. M.Sc. Thesis, Escola Superior de Agricultura Luiz de Queiroz, Universidade de São Paulo, Piracicaba, 85 p.
60. Mello CE (2008) Estrutura da vegetação arbórea em gradiente altitudinal de um morro testemunho em uma Floresta Estacional Semidecidual na região centro - sul do estado de São Paulo, sudeste do Brasil. M.Sc. Thesis, Universidade Estadual Paulista Júlio de Mesquita Filho, Botucatu, 61 p.
61. Melo MMRF and Mantovani W (1994) Composição florística e estrutura de trecho de Mata Atlântica de encosta, na Ilha do Cardoso (Cananéia, SP, Brasil). Boletim do Instituto de Botânica de São Paulo 9:107-158.

62. Melo MMRF, Oliveira RJ, Rossi L, Mamede MCH and Cordeiro I (2000) Estrutura de trecho de floresta atlântica de planície na Estação Ecológica de Juréia-Itatins, Iguape, SP, Brasil. Hoehnea 27:299-322.
63. Nicolini-Gabriel EM and Pagano SN (1993) Phytosociological Study on the Tree Stratum of A Semideciduous Mesophytic Forest in the Municipality of Jahu, Sp, Brazil. Arquivos de Biologia e Tecnologia de São Paulo 26:165-184.
64. Oliveira AA, Vicentini A, Chave J, Castanho CT, Davies SJ, Martini AMZ, Lima RAF, Iribar A and Souza VC (2014) Habitat specialization and phylogenetic structure of tree species in a coastal Brazilian white-sand forest. Journal of Plant Ecology 7:134-144.
65. Oliveira RJ, Mantovani W and Melo MMRF (2001) Estrutura do componente arbustivo-arbóreo da floresta atlântica de encosta, Peruíbe, SP. Acta Bot Brasilica 15:391-412.
66. Padgurschi MCG, Pereira LP, Tamashiro JY and Joly CA (2011) Composição e similaridade florística entre duas áreas de Floresta Atlântica Montana, São Paulo, Brasil. Biota Neotropica 11:139-152.
67. Pansonato, M, Unpublished data.
68. Penha AS (1998) Propagação vegetativa de espécies arbóreas a partir de raízes gemíferas: representatividade na estrutura fitossociológica e descrição dos padrões de rebrota de uma comunidade florestal, Campinas, São Paulo. M.Sc. Thesis, Universidade de Campinas, Campinas, 114 p.
69. Pinheiro ES and Durigan G (2012) Diferenças florísticas e estruturais entre fitofisionomias do cerrado em Assis, SP, Brasil. Revista Árvore 36:181-193.
70. Pinto MM (1989) Levantamento fitossociológico de uma mata residual: campus de Jaboticabal da UNESP. M.Sc. Thesis, Universidade Estadual Paulista Júlio de Mesquita Filho, Jaboticabal, 67 p.
71. Prata EMB, Assis MA and Joly CA (2011) Composição florística e estrutura da comunidade arbórea na transição da Floresta Ombrófila Densa das Terras Baixas - Floresta Ombrófila Densa Submontana do Núcleo Picinguaba/PESM, Ubatuba, sudeste do Brasil. Biota Neotropica 11:285-299.
72. Ramos E, Torres RB, Veiga RFA and Joly CA (2011) Study of the arboreal component in two areas of the Submontane Rainforest in Ubatuba, São Paulo State. Biota Neotropica 11:313-335.
73. Ranga NT, Rezende AA, Cavassan O, Toniato MTZ, Cielo-Filho R and Stranghetti V (2011) Caracterização florística de remanescentes de vegetação nativa da região noroeste do estado de São Paulo. In: Necchi Jr O (ed) Fauna e flora de fragmentos florestais remanescentes da regiao noroeste do estado de São Paulo. FAPESP, São Paulo, pp. 105-136.
74. Ribeiro TM, Ivanauskas NM, Martins SV, Polisel RT and Santos RLR (2013) Fitossociologia de uma Floresta Secundária com Araucaria angustifolia (Bertol.) O. Kuntze na Estação Ecológica de Bananal, Bananal-SP. Floresta e Ambiente 20:159-172.
75. Rocha FT (2003) Levantamento florestal na Estação Ecológica dos Caetetus como subsídio para laudos de desapropriação ambiental. M.Sc. Thesis, Escola Superior de Agricultura Luiz de Queiroz, Universidade de São Paulo, Piracicaba, 156 p.
76. Rochelle ALC, Cielo-Filho R and Martins FR (2011) Tree community structure in an Atlantic forest fragment at Serra do Mar State Park, southeastern Brazil. Biota Neotropica 11:337-346.
77. Rodrigues RR, Gandolfi S and Souza VC (2006) Diversidade, dinâmica e conservação em florestas do estado de São Paulo: 40,96ha de parcelas permanentes. Universidade de São Paulo, Piracicaba, 68 p.
78. Rozza AF (1997) Florística, Fitossociologia e Caracterização Sucessional Em Uma Floresta Estacional Semidecidual: Mata da Virgínia, Matão, SP. M.Sc. Thesis, Universidade Estadual de Campinas, Campinas, 177 p.
79. Sanchez M (2001) Composição florística e estrutura da comunidade arbórea num gradiente altitudinal da Mata Atlântica. D.Sc. Thesis, Universidade Estadual de Campinas, Campinas, 147 p.
80. Schlittler FHM, Demarinis G and Cesar O (1995) Phytosociological Studies in the Morro do Diabo Forest (Pontal do Paranapanema, Sp), Brazil. Arquivos de Biologia e Tecnologia de São Paulo 38:217-234.

81. Silva AF (1989) Composição florística e estrutura fitossociológica do estrato arbóreo da Reserva Florestal Professor Augusto Ruschi, São José dos Campos, SP. D.Sc. Thesis, Universidade Estadual de Campinas, Campinas, 148 p.
82. Silva LA and Soares JJ (2002) Levantamento fitossociológico em um fragmento de Floresta Estacional Semidecídua, no município de São Carlos, SP. Acta Bot Brasilica 16:205-216.
83. Soares MS, Soares JJ, Lima MIS (2005) Florística e fitossociologia de mata estacional semidecidual denominada "Serra d'Águá" em Araras - SP. In: Anais do 56º Congresso Nacional de Botânica, Curitiba, p. 247.
84. Souza ACO (2013) Fatores abióticos influenciando a vegetação em Floresta Ombrófila Densa Montana, Parque Estadual da Serra do Mar (Ubatuba - SP, Brasil). M.Sc. Thesis, Instituto Agronômico de Campinas, Campinas, 92 p.
85. Souza RPM (2008) Estrutura da comunidade arbórea de trechos de florestas de Araucaria no estado de São Paulo, Brasil. M.Sc. Thesis, Escola Superior de Agricultura Luiz de Queiroz, Universidade de São Paulo, Piracicaba, 101 p.
86. Souza RPM, Souza VC, Polisel RT and Ivanauskas NM (2012) Estrutura e aspectos da regeneração natural de Floresta Ombrófila Mista no Parque Estadual de Campos do Jordão, SP, Brasil. Hoehnea 39:387-407.
87. Stefani EJF (2013) Estrutura, composição florística e similaridade em áreas de floresta ombrófila densa submontana e montana do Parque Estadual da Serra do Mar, Litoral Norte/SP. M.Sc. Thesis, Universidade Estadual de Campinas, Campinas, 93 p.
88. Sztutman M and Rodrigues RR (2002) O mosaico vegetational numa área de floresta contínua da planície litorânea, Parque Estadual da Campina do Encantado, Pariquera-Açu, SP. Rev Bras Bot 25:161-176.
89. Tabanez AAJ, Viana VM, Dias AS (1997) Conseqüências da fragmentação e do efeito de borda sobre a estrutura, diversidade e sustentabilidade de um fragmento de floresta de Planalto de Piracicaba, SP. Rev Bras Biol 57:47-60.
90. Tanaka GK (2009) Estrutura e florística do estrato arbóreo de um fragmento de floresta estacional semidecidual: Estação Ecológica de Ribeirão Preto, SP. M.Sc. Thesis, Universidade de São Paulo, Ribeirão Preto, 69 p.
91. Teixeira AP and Assis MA (2005) Caracterização florística e fitossociológica do componente arbustivo-arbóreo de uma floresta paludosa no município de Rio Claro (SP), Brasil. Rev Bras Bot 28:467-476.
92. Teixeira AP and Assis MA (2009) Relação entre heterogeneidade ambiental e distribuição de espécies em uma floresta paludosa no Município de Cristais Paulista, SP, Brasil. Acta Botanica Brasilica 23:843-853.
93. Teixeira AP, Assis MA and Luize BG (2011) Vegetation and environment relationships in a Neotropical swamp forest in southeastern Brazil (Itirapina, SP). Aquatic Botany 94:17-23.
94. Tomasetto, F (2003) Composição florística e estrutura do componente arbóreo de um trecho de floresta estacional semidecidual na Estação Ecológica de Paulo de Faria – SP. M.Sc. Thesis, Universidade Estadual Paulista, Rio Claro, 133 p.
95. Tomasulo PLB and Cordeiro I (2000) Composição florística do Parque Municipal da Serra do Itapety, Mogi das Cruzes, SP. Boletim do Instituto de Botânica 14:139-161.
96. Toniato MTZ and Oliveira-Filho AT (2004) Variations in tree community composition and structure in a fragment of tropical semideciduous forest in southeastern Brazil related to different human disturbance histories. Forest Ecology and Management 198: 319-339.
97. Toppa RH (2004) Estrutura e diversidade florística das diferentes fisionomias de cerrado e suas correlações com o solo na Estação Ecológica de Jataí, Luiz Antônio, SP. D.Sc. Thesis, Universidade Federal de São Carlos, São Carlos, SP, 127 p.
98. Urbanetz C (2010) Fatores abióticos e variação espacial na estrutura da Floresta Ombrófila Densa Atlântica. D.Sc. Thesis, Universidade de Campinas, Campinas, 114 p.
99. Vanini A (1999) Estudo comparativo de dois métodos de amostragem fitossociológica em Caxetais (floresta ombrófila densa permanentemente alagada). M.Sc. Thesis, Escola Superior de Agricultura Luiz de Queiroz, Universidade de São Paulo, Piracicaba, 120 p.

100. Viani RAG (2005) O uso da regeneração natural (Floresta Estacional Semidecidual e talhões de Eucalyptus) como estratégia de produção de mudas e resgate da diversidade vegetal na restauração florestal. M.Sc. Thesis, Universidade Estadual de Campinas, Campinas, 188 p.
101. Yamamoto LF (2009) Florística e fitossociologia de espécies arbóreas ao longo de um gradiente altitudinal no extremo sul da Mantiqueira (Serra do Lopo) - MG/SP. D.Sc. Thesis, Universidade de Campinas, Campinas, 169 p.
