# Supplementary material for: Can plant DNA barcoding be implemented in species-rich tropical regions? A perspective from São Paulo State, Brazil
Source: Genet Mol Biol. 2018 Jul-Sep;41(3):661–70. doi: 10.1590/1678-4685-GMB-2017-0282 (PMC6136365; doi:10.1590/1678-4685-GMB-2017-0282)
Supplement: Supplementary file 2 [file 1415-4757-GMB-41-03-2017-0282-20180723-suppl2.pdf]

Supplementary Material to “Can plant DNA barcoding be implemented in species-rich tropical regions? A perspective from São Paulo State, Brazil”

Table S2 - Primers and protocol used to obtain the DNA sequences and their respective sources.

Region	Primer	Sequence 5'-3'	Source
*rbc*La	724R	TCGCATATGTACCTGCAGTAGC	1
	1F	ATGTCACCACAAACAGAAAC	1
*mat*K	3F_Kim	CGTACAGTACTTTTGTGTTTACGAG	2
	1R_Kim	ACCCAGTCCATCTGGAAATCTTGGTTC	2
	5R	GTTCTAGCACAAGAAAGTCG	3
	XF	TAATTTACGATCAATTCATTC	3
	390F	CGATCTATTCATTCAATATTTC	4
	1326R	TCTAGCACACGAAAGTCGAAGT	4
	3F1	CGCTGTRATAATGAGAAAGRTTTC	5
	1R1	CATTTATTRCGATTCTTTCTYYACG	5
	Fi-Mora	ATTTTCTAGCATTTGACTYCGTAC	6
	Fi-Lau	GAATTCTCTAGCATTTGAYTCCTT	6
	Ri-Mora	CATGCATTATGTTAGRTATCAAGG	6
	Ri-Lau	ATGCATTATGTCCGATATCAAGG	6
ITS region	ITS-p5 (Fwd)	CCTTATCAYTTAGAGGAAGGAG	7
	ITS-p3 (Fwd)	YGACTCTCGGCAACGGATA	7
	ITS-p4 (Rev)	CCGCTTAKTGATATGCTTAAA	7

1- Kress WJ and Erickson DL (2007) A two-locus global DNA barcode for land plants: the coding gene complements the non-coding spacer region. PLoS One 2:e508.

2- Ki-Joong Kim unpublished *apud* Dunning LT and Savolainen V (2010) Broad-scale amplification of matK for DNA barcoding plants, a technical note. Bot J Linn Soc 164:1-9.

3- Ford CS, Ayres KL, Toomey N, Haider N, Stahl JVA, Kelly LJ, et al. (2009) Selection of candidate coding DNA barcoding regions for use on land plants. Bot J Linn Soc 159: 1–11.

4- Chatrou et al. unpublished *apud* Sun H, McLewin W and Fay MF (2001) Molecular phylogeny of *Helleborus* (Ranunculaceae), with an emphasis on the East Asia–Mediterranean disjunction. Taxon 50:1001–1018.

5- New adapted primers designed Guillaume Besnard (CNRS)

6- New internal primers designed Guillaume Besnard (CNRS)

7- Cheng T, Xu C, Lei L, Li C, Zhang Y and Zhou S (2016) Barcoding the kingdom Plantae: new PCR primers for ITS regions of plants with improved universality and specificity. Mol Ecol Resour 16:138-149.

Protocol

Leaf samples were stored in paper filter and dried in silica-gel. Genomic DNA was obtained using a 2% CTAB procedure on disrupted leaf fragments (ca. 1 cm^2), that were previously frozen at -80ºC. The CTAB buffer was incubated (65 ºC for 1 h, vortexing it at every 20 minutes) and then centrigugated (10 min at 14,000 rpm). Following centrifugation, the supernatant was submited to a BioSprint 15 workstation (Qiagen®, USA) using the manufactures's protocol for plant DNA extraction. For those DNA extracts that could not be amplified, we reruned PCRs either using a larger quantity of DNA, dilluted extracts or using prufied DNA extracts using the comercial kit Dneasy PlantMini (Qiagen®, USA), according to the manufacturer's protocol as well.

To amplify *rbc*L, *mat*K and ITS regions, we used the standard PCR procedures. Reactions were performed in 25 µl reaction mix composed by: 1 µl of DNA extract (10 ng/µl), 17.4 µl of RNAase free water, 5 µl of 5x Reaction Buffer, 0.5 µl of dNTP Mix (10 mM - both Promega), 0.5 µl of each forward and reverse primer (20 µM) and 0.1 µl of GoTaq® DNA polymerase (Promega). For *rbc*L we used 1F/724R and for the ITS region we used the plant specific p-5 or p-3/p-4 primers, while for *mat*K we used 3F_Kim/1R_Kim primers; whenever DNA amplification were not attained for *mat*K, it was used 390F/1326R or XR/5F primers (Table S2). For genus or families with no *mat*K accessions available, we also used internal primers kindly designed by Guillaume Besnard (CNRS, France) specifically for Magnoliales and Rosids (Table S2).

The PCR profile used for the amplification of *rbc*L and ITS region was: 95 °C for 1 min, 35 cycles of 95 °C for 1 min, 50 °C for 30 s, 72 °C for 2 min, with a final extension of 72 °C for 10 min. For *mat*K, the profile used was (Dunning & Savolainen 2010): 94 °C for 5 min, 38 cycles of 94 °C, 48 °C, 72 °C for 40 s each, with a final extension of 72 °C for 7 min. Whenever PCRs did not result in good PCR products, slightly different PCR profiles were used instead, generally varying the temperature and time spent at low and high parts of cycles. DNA amplification was inspected using electrophoresis in agarose gel containing ethidium bromide. Succesful PCRs were sent to GenoScreen (http://www.genoscreen.fr), where they were purified and sequenced using Sanger technology. Forward and reverse readings were trimmed and assembled into contigs using Geneious version 9.0.5 (http://www.geneious.com) and possible contaminations were verified using Blast (http://blast.ncbi.nlm.nih.gov).

We found a higher success to sequence *rbc*L (83%) than *mat*K (76%), even after trying all the combinations of primers and PCR profiles mentioned above for *mat*K. A relative poor success for both plastid barcodes (average <75%) was found for the Ericales, Malpighiales, Arecales, Aquifoliales and Myrtales. In addition, our success to sequence *mat*K was much lower than that of *rbc*L for Cyatheales, Piperales and Sapindales. For the Cyatheales, in particular, we were not able to amplify *mat*K at all. On the other hand, we had a higher success to sequence *mat*K for the Apiales and Magnoliales.
