# Supplementary material for: Can plant DNA barcoding be implemented in species-rich tropical regions? A perspective from São Paulo State, Brazil
Source: Genet Mol Biol. 2018 Jul-Sep;41(3):661–70. doi: 10.1590/1678-4685-GMB-2017-0282 (PMC6136365; doi:10.1590/1678-4685-GMB-2017-0282)
Supplement: Supplementary file 3 [file 1415-4757-GMB-41-03-2017-0282-20180723-suppl3.pdf]

Supplementary Material to "Can plant DNA barcoding be implemented in species-rich tropical regions? A perspective from São Paulo State, Brazil"

Table S3. Results per family used to calculate the sampling priority index *P* presented in the main text. The legend to all symbols is given in the text above. The protocol is presented below.

Family	S	S_{with}	N_b	Z_t	Z_e	P_b	P_t	P_e	P
Acanthaceae	3	3	10	0	0	0.333	0.000	0.000	0.333
Achatocarpaceae	1	1	2	0	0	0.600	0.000	0.000	0.600
Adoxaceae	1	1	1	0	0	0.800	0.000	0.000	0.800
Anacardiaceae	14	9	38	0	3	0.156	0.000	0.300	0.456
Annonaceae	35	24	61	2	9	0.492	0.091	0.409	0.992
Apocynaceae	29	17	41	3.5	6.5	0.518	0.146	0.271	0.934
Aquifoliaceae	13	11	37	0	1	0.327	0.000	0.250	0.577
Araliaceae	16	11	31	0	6.5	0.436	0.000	0.650	1.086
Araucariaceae	1	1	5	0	0	0.000	0.000	0.000	0.000
Arecaceae	22	13	34	0.5	7.5	0.477	0.028	0.417	0.921
Asteraceae	63	29	74	0.5	26	0.490	0.007	0.382	0.879
Berberidaceae	1	1	1	0	0	0.800	0.000	0.000	0.800
Bignoniaceae	31	21	60	0.5	9	0.429	0.025	0.450	0.904
Bixaceae	2	1	5	0	0	0.000	0.000	0.000	0.000
Boraginaceae	13	10	30	0	5	0.400	0.000	0.833	1.233
Burseraceae	7	7	17	0	0	0.514	0.000	0.000	0.514
Cactaceae	10	8	27	0	2.5	0.325	0.000	0.625	0.950
Calophyllaceae	9	3	10	0	3.5	0.333	0.000	0.292	0.625
Canellaceae	2	2	6	0	0	0.400	0.000	0.000	0.400
Cannabaceae	6	5	15	0	0.5	0.400	0.000	0.250	0.650
Capparaceae	3	3	13	0	0	0.133	0.000	0.000	0.133
Cardiopteridaceae	2	1	2	0	0.5	0.600	0.000	0.250	0.850
Caricaceae	5	5	18	0	0	0.280	0.000	0.000	0.280
Caryocaraceae	1	1	3	0	0	0.400	0.000	0.000	0.400
Casuarinaceae	1	1	5	0	0	0.000	0.000	0.000	0.000
Celastraceae	29	16	49	1.5	12	0.388	0.058	0.462	0.907
Chloranthaceae	1	1	3	0	0	0.400	0.000	0.000	0.400
Chrysobalanaceae	24	11	31	5	11.5	0.436	0.192	0.442	1.071
Clethraceae	1	1	4	0	0	0.200	0.000	0.000	0.200
Clusiaceae	7	6	18	0	1	0.400	0.000	0.500	0.900
Combretaceae	15	6	17	0	4	0.433	0.000	0.222	0.656
Connaraceae	6	4	5	1	1	0.750	0.250	0.250	1.250

Family	S	S_{with}	N_b	Z_t	Z_e	P_b	P_t	P_e	P
Cunoniaceae	9	2	7	2	7	0.300	0.143	0.500	0.943
Cyatheaceae	17	6	8	0.5	6	0.733	0.023	0.273	1.029
Dichapetalaceae	4	0	0	0	7	0.500	0.500	0.500	1.500
Dicksoniaceae	1	1	3	0	0	0.400	0.000	0.000	0.400
Dilleniaceae	3	2	3	0	0	0.700	0.000	0.000	0.700
Ebenaceae	3	2	3	0	2	0.700	0.000	0.333	1.033
Elaeocarpaceae	7	4	10	0	2.5	0.500	0.000	0.417	0.917
Ericaceae	9	2	8	2.5	5.5	0.200	0.179	0.393	0.771
Erythroxylaceae	21	7	17	1	5.5	0.514	0.036	0.196	0.746
Escalloniaceae	4	3	6	0	2	0.600	0.000	0.333	0.933
Euphorbiaceae	64	40	98	1	22	0.510	0.021	0.458	0.989
Fabaceae	211	152	469	4	51.5	0.383	0.034	0.436	0.853
Griseliniaceae	1	1	2	0	0	0.600	0.000	0.000	0.600
Humiriaceae	4	2	4	0	2	0.600	0.000	0.500	1.100
Hypericaceae	3	1	1	2	1.5	0.333	0.500	0.375	1.208
Lacistemataceae	5	2	5	0	3	0.500	0.000	0.500	1.000
Lamiaceae	18	7	15	0	5.5	0.571	0.000	0.250	0.821
Lauraceae	105	55	136	21	59	0.505	0.210	0.590	1.305
Laxmanniaceae	1	1	2	0	0	0.600	0.000	0.000	0.600
Lecythidaceae	4	4	13	0	0	0.350	0.000	0.000	0.350
Loganiaceae	3	3	8	0	0	0.467	0.000	0.000	0.467
Lythraceae	3	2	6	0	0	0.400	0.000	0.000	0.400
Magnoliaceae	1	1	3	0	0	0.400	0.000	0.000	0.400
Malpighiaceae	21	11	23	0	7	0.582	0.000	0.350	0.932
Malvaceae	37	17	48	3	12	0.435	0.075	0.300	0.810
Melastomataceae	121	83	122	4	45.5	0.706	0.053	0.599	1.357
Meliaceae	20	16	54	0	1.5	0.325	0.000	0.188	0.513
Monimiaceae	26	9	23	2.5	17.5	0.489	0.074	0.515	1.077
Moraceae	40	30	77	0	3.5	0.487	0.000	0.175	0.662
Myristicaceae	3	2	4	0	0.5	0.600	0.000	0.250	0.850
Myrtaceae	324	154	511	23	202.5	0.336	0.068	0.596	1.000
Nyctaginaceae	16	10	24	1	2	0.520	0.083	0.167	0.770
Ochnaceae	12	6	12	0	4.5	0.600	0.000	0.375	0.975
Olacaceae	4	3	8	2	0.5	0.467	0.333	0.250	1.050
Oleaceae	5	2	9	1	3	0.100	0.167	0.500	0.767
Onagraceae	3	2	5	0	0	0.500	0.000	0.000	0.500
Opiliaceae	2	1	2	0	0	0.600	0.000	0.000	0.600
Pentaphylacaceae	2	1	2	2	1	0.600	0.500	0.500	1.600
Peraceae	3	2	5	0	0	0.500	0.000	0.000	0.500
Phyllanthaceae	11	8	22	0	4	0.450	0.000	0.667	1.117
Phytolaccaceae	4	3	9	0	0.5	0.400	0.000	0.250	0.650
Picramniaceae	6	3	5	0	1	0.667	0.000	0.167	0.833
Pinaceae	3	3	11	0	0	0.267	0.000	0.000	0.267
Piperaceae	18	11	36	1.5	2	0.345	0.107	0.143	0.595
Pittosporaceae	1	1	5	0	0	0.000	0.000	0.000	0.000

Family	S	S_{with}	N_b	Z_t	Z_e	P_b	P_t	P_e	P
Poaceae	45	17	43	6	30	0.494	0.107	0.536	1.137
Podocarpaceae	2	2	9	0	0	0.100	0.000	0.000	0.100
Polygalaceae	5	3	9	0	1	0.400	0.000	0.250	0.650
Polygonaceae	14	6	15	0	4.5	0.500	0.000	0.281	0.781
Primulaceae	33	12	28	0	17.5	0.533	0.000	0.417	0.950
Proteaceae	12	2	10	1.5	10	0.000	0.075	0.500	0.575
Quiinaceae	2	1	2	0.5	2	0.600	0.250	0.333	1.183
Rhamnaceae	8	5	16	1.5	1	0.360	0.250	0.167	0.777
Rhizophoraceae	1	1	4	0	0	0.200	0.000	0.000	0.200
Rosaceae	3	2	10	0	0.5	0.000	0.000	0.250	0.250
Rubiaceae	126	62	163	7.5	80.5	0.474	0.059	0.629	1.162
Rutaceae	32	25	77	2	3.5	0.384	0.143	0.250	0.777
Sabiaceae	3	2	4	0	1	0.600	0.000	0.500	1.100
Salicaceae	25	13	29	0	8.5	0.554	0.000	0.354	0.908
Sapindaceae	33	16	49	2	15.5	0.388	0.059	0.456	0.902
Sapotaceae	31	27	91	0	3.5	0.326	0.000	0.438	0.763
Schoepfiaceae	1	0	0	0	0	1.000	0.000	0.000	1.000
Simaroubaceae	2	2	4	0	0	0.600	0.000	0.000	0.600
Siparunaceae	4	4	12	0	0	0.400	0.000	0.000	0.400
Solanaceae	77	40	95	0	29.5	0.525	0.000	0.399	0.924
Styracaceae	10	8	14	0	2.5	0.650	0.000	0.625	1.275
Symplocaceae	25	19	56	0	7.5	0.411	0.000	0.625	1.036
Theaceae	1	1	2	0	0	0.600	0.000	0.000	0.600
Thymelaeaceae	8	3	5	0	6.5	0.667	0.000	0.500	1.167
Ulmaceae	1	1	4	0	0	0.200	0.000	0.000	0.200
Urticaceae	11	8	22	0	1	0.450	0.000	0.167	0.617
Verbenaceae	9	5	18	0	2	0.280	0.000	0.250	0.530
Violaceae	3	3	9	0	0	0.400	0.000	0.000	0.400
Vochysiaceae	29	7	10	0	20.5	0.714	0.000	0.466	1.180
Winteraceae	1	1	3	0	0	0.400	0.000	0.000	0.400

The priority index for future sequencing efforts for tree species in the state of São Paulo, Brazil.

We calculated a sampling priority index (P) in order to orient future sequencing efforts of the SP tree flora. This index was based on three different components. The first component was the proportion of sequences of the top five barcodes (i.e. ITS, *rbc*L, *mat*K, *psb*A-*trn*H and *trn*L) that are still not available for species of each family (P_b), defined as:

$$P_b = 1 - N_b/(S \times 5),$$

where N_b is the number of barcodes available for species of the family and S is the total richness of the family. The denominator is actually the maximum number of barcodes a family can have for the five barcodes considered here. If all species of a given family have all five barcodes already available for the SP tree flora, P_b is zero.

Next, we calculated the proportion of threatened (P_t) and endemic species without barcodes (P_e) per family. To do so, we first substitute the different levels of extinction threaten and endemism by scores, aiming to give higher sampling priority levels to species that are most critical to conservation. We assigned 0.5, 1 and 2 for species Vulnerable, Endangered and Critically Endangered or Extinct in the Wild, respectively. Similarly, we assigned 0.5, 1 and 2 for species restricted to Eastern South-America and for Regional and Local endemics, respectively. We assigned 0 to all other categories of extinction threaten and geographical distribution. Then, we summed these scores for all

species without sequences for the top five DNA markers, a sum defined here as Zt and Z_e. Thus, P_t and P_e was calculated as follows:

$$P_t = Zt /[(S - S_{with}) \times 2] \text{ and}$$

$$P_e = Ze /[(S - S_{with}) \times 2],$$

where S_{with} is the number of species already with sequences available for any of the top five barcodes. Again, the denominator is the maximum score a family can have, in the case that all species without barcode have score equals two. Therefore, these two proportions summarize the "conservation score" of the SP tree species that still have no barcode available. Finally, the sampling priority index is obtained by summing these three proportions:

$$P = P_b + P_t + P_{e.}$$

This priority index varies from 0 to 3. The higher the index, the greater the priority of the family for future sequencing efforts. We provide this index and the individual proportion for each of the 107 families of the SP tree flora (Table S3), which were used to produce Figure 2 in the main text. In practice, the value o P varied from 0 to 1.6 for SP tree flora.
