# Supplementary material for: Can plant DNA barcoding be implemented in species-rich tropical regions? A perspective from São Paulo State, Brazil
Source: Genet Mol Biol. 2018 Jul-Sep;41(3):661–70. doi: 10.1590/1678-4685-GMB-2017-0282 (PMC6136365; doi:10.1590/1678-4685-GMB-2017-0282)
Supplement: Supplementary file 4 [file 1415-4757-GMB-41-03-2017-0282-20180723-suppl4.pdf]

Supplementary Material to "Can plant DNA barcoding be implemented in species-rich tropical regions? A perspective from São Paulo State, Brazil"

Table S4 - The full list of the São Paulo tree flora, including their life form, geographical distribution, threat status at state-level and the source of the record. In the first column, species followed by '(*)' are exotic species naturalized in the state of São Paulo. Life form followed by '(H)' or '(L)' means hemi-epiphytic or lianescent, respectively. Geographical distribution is Pantropical, Neotropical, South-American (SA), Eastern South-American (ESA), Regional and Local endemics. The threat statuses are vulnerable (VU), endangered (EN), critically endangered (CR) and probably extinct (EX). The source are the Brazilian Flora Checklist project (Reflora − Zappi *et al*., 2015) and the NeoTropTree database (NeoTrop − Oliveira-Filho, 2014).

Family (Order) and species	Life form	Geog. distribut.	Threat status	Source
ACANTHACEAE (Lamiales)				
Avicennia germinans (L.) L.	Tree	Pantrop.		Reflora
Avicennia schaueriana Stapf & Leechm. ex Moldenke	Tree	Neotrop.		Reflora
Ruellia brevifolia (Pohl) C.Ezcurra	Shrub	SA		NeoTrop
ACHATOCARPACEAE (Caryophyllales)				
Achatocarpus praecox Griseb.	Tree	SA		Reflora
ADOXACEAE (Dipsacales)				
Sambucus australis Cham. & Schltdl.	Tree	SA		Reflora
ANACARDIACEAE (Sapindales)				
Anacardium humile A.St.-Hil.	Tree	SA		Reflora
Anacardium occidentale L. (*)	Tree	SA		Reflora
Astronium graveolens Jacq.	Tree	SA		Reflora
Lithrea brasiliensis Marchand	Tree	ESA		Reflora
Lithrea molleoides (Vell.) Engl.	Tree	ESA		Reflora
Mangifera indica L. (*)	Tree	Neotrop.		Reflora
Myracrodruon urundeuva Allemão	Tree	ESA	VU	Reflora
Schinus engleri F.A.Barkley	Tree	Regional		Reflora
Schinus ramboi F.A.Barkley	Tree	Regional		Reflora
Schinus terebinthifolius Raddi	Tree	Neotrop.		Reflora

Family (Order) and species	Life form	Geog. distribut.	Threat status	Source
Spondias mombin L.	Tree	ESA		Reflora
Tapirira guianensis Aubl.	Tree	Neotrop.		Reflora
Tapirira obtusa (Benth.) J.D.Mitch.	Tree	SA		Reflora
ANNONACEAE (Magnoliales)				
Anaxagorea dolichocarpa Sprague & Sandwith	Tree	Neotrop.		NeoTrop
Annona cacans Warm.	Tree	ESA		Reflora
Annona coriacea Mart.	Tree	SA		Reflora
Annona cornifolia A.St.-Hil.	Tree	ESA		Reflora
Annona crassiflora Mart.	Tree	ESA		Reflora
Annona crotonifolia Mart.	Tree	ESA		Reflora
Annona dolabripetala Raddi	Tree	ESA		Reflora
Annona emarginata (Schltdl.) H.Rainer	Tree	SA		Reflora
Annona glabra L.	Tree	Neotrop.		Reflora
Annona malmeana R.E.Fr.	Shrub	ESA		Reflora
Annona montana Macfad.	Tree	Neotrop.		Reflora
Annona mucosa Jacq.	Tree	Neotrop.		NeoTrop
Annona neosericea H.Rainer	Tree	Regional		Reflora
Annona nutans (R.E.Fr.) R.E.Fr.	Shrub	ESA		NeoTrop
Annona parviflora (A.St.-Hil.) H.Rainer	Tree	Local	EN	NeoTrop
Annona rugulosa (Schltdl.) H.Rainer	Tree	Regional		Reflora
Annona sylvatica A.St.-Hil.	Tree	ESA		Reflora
Annona ubatubensis (Maas & Westra) H.Rainer	Tree	Local	EN	Reflora
Annona xylopiifolia A.St.-Hil. & Tul.	Shrub	Regional		Reflora
Duguetia furfuracea (A.St.-Hil.) Saff.	Shrub	SA		Reflora
Duguetia lanceolata A.St.-Hil.	Tree	ESA		Reflora
Duguetia salicifolia R.E.Fr.	Tree	Local	VU	Reflora
Guatteria australis A.St.-Hil.	Tree	ESA		Reflora
Guatteria latifolia R.E.Fr.	Tree	Regional		NeoTrop
Guatteria pohliana Schltdl.	Tree	Regional		Reflora
Guatteria sellowiana Schltdl.	Tree	ESA		NeoTrop

Family (Order) and species	Life form	Geog. distribut.	Threat status	Source
Porcelia macrocarpa (Warm.) R.E.Fr.	Tree	ESA		Reflora
Trigynaea oblongifolia Schltdl.	Shrub	Regional		Reflora
Unonopsis guatterioides (A.DC.) R.E.Fr.	Tree	SA		Reflora
Xylopia aromatica (Lam.) Mart.	Tree	Neotrop.		Reflora
Xylopia brasiliensis Spreng.	Tree	SA		Reflora
Xylopia emarginata Mart.	Tree	SA		Reflora
Xylopia langsdorfiana A.St.-Hil. & Tul.	Tree	Regional		Reflora
Xylopia sericea A.St.-Hil.	Tree	Neotrop.		NeoTrop
APOCYNACEAE (Gentianales)				
Aspidosperma australe Müll.Arg.	Tree	ESA		Reflora
Aspidosperma compactinervium Kuhlm.	Tree	Regional		NeoTrop
Aspidosperma cuspa (Kunth) S.F.Blake ex Pittier	Tree	Neotrop.		Reflora
Aspidosperma cylindrocarpon Müll.Arg.	Tree	SA		Reflora
Aspidosperma discolor A.DC.	Tree	SA		Reflora
Aspidosperma macrocarpon Mart.	Tree	SA	VU	Reflora
Aspidosperma multiflorum A.DC.	Tree	SA		Reflora
Aspidosperma nobile Müll.Arg.	Tree	SA	CR	Reflora
Aspidosperma olivaceum Müll.Arg.	Tree	Regional		Reflora
Aspidosperma parvifolium A.DC.	Tree	SA		Reflora
Aspidosperma polyneuron Müll.Arg.	Tree	ESA		Reflora
Aspidosperma pyricollum Müll.Arg.	Tree	ESA		Reflora
Aspidosperma ramiflorum Müll.Arg.	Tree	ESA		Reflora
Aspidosperma riedelii Müll.Arg.	Tree	ESA	EN	Reflora
Aspidosperma spruceanum Benth. ex Müll.Arg.	Tree	ESA	CR	Reflora
Aspidosperma subincanum Mart.	Tree	ESA		Reflora
Aspidosperma tomentosum Mart.	Tree	SA	EN	Reflora
Aspidosperma warmingii Müll.Arg.	Tree	Regional		Reflora
Calotropis procera (Aiton) W.T.Aiton	Tree	Pantrop.		Reflora
Hancornia speciosa Gomes	Tree	SA		Reflora
Himatanthus lancifolius (Müll.Arg.) Woodson	Tree	Regional		NeoTrop

Family (Order) and species	Life form	Geog. distribut.	Threat status	Source
Himatanthus obovatus (Müll.Arg.) Woodson	Tree	SA		Reflora
Malouetia cestroides (Nees ex Mart.) Müll.Arg.	Tree	SA		Reflora
Rauvolfia sellowii Müll.Arg.	Tree	ESA		Reflora
Tabernaemontana catharinensis A.DC.	Tree	ESA		Reflora
Tabernaemontana hystrix Steud.	Tree	ESA		Reflora
Tabernaemontana laeta Mart.	Tree	ESA		Reflora
Thevetia peruviana (Pers.) K.Schum.	Tree	SA		Reflora
AQUIFOLIACEAE (Aquifoliales)				
Ilex affinis Gardner	Tree	ESA		Reflora
Ilex brasiliensis (Spreng.) Loes.	Tree	ESA		Reflora
Ilex brevicuspis Reissek	Tree	ESA		Reflora
Ilex cerasifolia Reissek	Tree	ESA		Reflora
Ilex chamaedryfolia Reissek	Shrub	Regional		Reflora
Ilex dumosa Reissek	Tree	ESA		Reflora
Ilex floribunda Reissek ex Maxim.	Tree	ESA		Reflora
Ilex integerrima (Vell.) Reissek	Tree	ESA		Reflora
Ilex microdonta Reissek	Tree	ESA		Reflora
Ilex paraguariensis A.St.-Hil.	Tree	ESA		Reflora
Ilex pseudobuxus Reissek	Tree	ESA		Reflora
Ilex taubertiana Loes.	Tree	ESA		Reflora
Ilex theezans Mart. ex Reissek	Tree	ESA		Reflora
ARALIACEAE (Apiales)				
Aralia warmingiana (Marchal) J.Wen	Tree	ESA		Reflora
Dendropanax australis Fiaschi & Jung-Mend.	Shrub	Local		Reflora
Dendropanax cuneatus (DC.) Decne. & Planch.	Tree	SA		Reflora
Dendropanax denticulatus Fiaschi	Tree	Local		Reflora
Dendropanax exilis (Toledo) S.L.Jung	Shrub	Local		Reflora
Dendropanax monogynus (Vell.) Seem.	Tree	Local		Reflora
Dendropanax nebulosus Fiaschi & Jung-Mend.	Tree	Local		Reflora
Oreopanax capitatus (Jacq.) Decne. & Planch.	Tree (H)	Neotrop.		Reflora

Family (Order) and species	Life form	Geog. distribut.	Threat status	Source
Oreopanax fulvus Marchal	Tree	ESA		Reflora
Schefflera actinophylla (Endl.) Harms (*)	Tree	exotic		Reflora
Schefflera angustissima (Marchal) Frodin	Tree	ESA		Reflora
Schefflera calva (Cham.) Frodin & Fiaschi	Tree	ESA		Reflora
Schefflera macrocarpa (Cham. & Schltdl.) Frodin	Tree	ESA		Reflora
Schefflera malmei (Harms) Frodin	Tree	SA		Reflora
Schefflera morototoni (Aubl.) Maguire et al.	Tree	Neotrop.		Reflora
Schefflera vinosa (Cham. & Schltdl.) Frodin & Fiaschi	Tree	ESA		Reflora
ARAUCARIACEAE (Pinales)				
Araucaria angustifolia (Bertol.) Kuntze	Tree	ESA	VU	Reflora
ARECACEAE (Arecales)				
Acrocomia aculeata (Jacq.) Lodd. ex Mart.	Palm	Neotrop.		NeoTrop
Archontophoenix cunninghamiana (*)	Palm	Pantrop.		NeoTrop
Astrocaryum aculeatissimum (Schott) Burret	Palm	ESA		NeoTrop
Attalea compta Mart.	Palm	Regional		NeoTrop
Attalea dubia (Mart.) Burret	Palm	Neotrop.		NeoTrop
Attalea oleifera Barb.Rodr.	Palm	ESA	VU	NeoTrop
Attalea phalerata Mart. ex Spreng.	Palm	SA	EN	NeoTrop
Bactris hatschbachii Noblick ex A.J.Hend.	Palm	Regional		Reflora
Bactris setosa Mart.	Palm	ESA		Reflora
Bactris vulgaris Barb.Rodr.	Palm	Regional		Reflora
Butia eriospatha (Mart. ex Drude) Becc.	Palm	Regional		Reflora
Butia paraguayensis (Barb.Rodr.) Bailey	Palm	Regional		Reflora
Euterpe edulis Mart.	Palm	ESA	VU	Reflora
Geonoma elegans Mart.	Palm	ESA		NeoTrop
Geonoma pohliana Mart.	Palm	ESA		Reflora
Geonoma schottiana Mart.	Palm	ESA		Reflora
Lytocaryum hoehnei (Burret) Toledo	Palm	Local		Reflora
Mauritia flexuosa L.f.	Palm	Neotrop.		Reflora

Family (Order) and species	Life form	Geog. distribut.	Threat status	Source
Syagrus flexuosa (Mart.) Becc.	Palm	ESA		Reflora
Syagrus oleracea (Mart.) Becc.	Palm	ESA		Reflora
Syagrus pseudococos (Raddi) Glassman	Palm	Regional		Reflora
Syagrus romanzoffiana (Cham.) Glassman	Palm	ESA		Reflora
ASTERACEAE (Asterales)				
Austrocritonia angulicaulis (Sch.Bip. ex Baker) R.M.King & H.Rob.	Tree	Regional	VU	Reflora
Austrocritonia velutina (Gardner) R.M.King & H.Rob.	Tree	Regional		Reflora
Austroeupatorium inulaefolium (Kunth) R.M.King & H.Rob.	Shrub	Neotrop.		Reflora
Baccharis calvescens DC.	Tree	ESA		Reflora
Baccharis caprariifolia DC.	Shrub	ESA		Reflora
Baccharis dentata (Vell.) G.M.Barroso	Tree	ESA		Reflora
Baccharis dracunculifolia DC.	Tree	ESA		Reflora
Baccharis glaziovii Baker	Shrub	ESA		Reflora
Baccharis grandimucronata Malag.	Shrub	Regional		Reflora
Baccharis intermixta Gardner	Shrub	ESA		Reflora
Baccharis lateralis Baker	Tree	ESA		NeoTrop
Baccharis leucocephala Dusén	Shrub	Regional		NeoTrop
Baccharis microdonta DC.	Shrub	ESA		Reflora
Baccharis montana DC.	Tree	ESA		Reflora
Baccharis oblongifolia (Ruiz & Pav.) Pers.	Shrub	SA		Reflora
Baccharis oreophila Malme	Tree	Regional		Reflora
Baccharis regnellii Sch.Bip. ex Baker	Shrub	Regional		Reflora
Baccharis retusa DC.	Tree	ESA		Reflora
Baccharis semiserrata DC.	Tree	ESA		Reflora
Baccharis serrulata (Lam.) Pers.	Tree	SA		Reflora
Baccharis singularis (Vell.) G.M.Barroso	Tree	ESA		Reflora
Critoniopsis quinqueflora (Less.) H.Rob.	Tree	Regional		Reflora
Critoniopsis stellata (Spreng.) H.Rob.	Tree	Local		Reflora

Family (Order) and species	Life form	Geog. distribut.	Threat status	Source
Dasyphyllum brasiliense (Spreng.) Cabrera	Tree (L)	ESA		Reflora
Dasyphyllum spinescens (Less.) Cabrera	Tree (L)	ESA		Reflora
Dendrophorbium glaziovii (Baker) C.Jeffrey	Tree	Regional		Reflora
Eremanthus elaeagnus (Mart. ex DC.) Sch.Bip.	Tree	Regional		Reflora
Eremanthus erythropappus (DC.) MacLeish	Tree	ESA		Reflora
Eremanthus mattogrossensis Kuntze	Shrub	ESA		Reflora
Gochnatia barrosoae Cabrera	Shrub	ESA		NeoTrop
Gochnatia floribunda Cabrera	Tree	ESA		Reflora
Gochnatia paniculata (Less.) Cabrera	Tree	ESA		Reflora
Gochnatia polymorpha (Less.) Cabrera	Tree	ESA		Reflora
Gochnatia pulchra Cabrera	Shrub	ESA		Reflora
Gochnatia sordida (Less.) Cabrera	Shrub	Regional		Reflora
Gochnatia velutina (Bong.) Cabrera	Tree	Local		Reflora
Idiothamnus pseudorgyalis R.M.King & H.Rob.	Shrub	Regional	VU	Reflora
Kaunia rufescens (Lund ex DC.) R.M. King & H. Rob.	Shrub	ESA		Reflora
Lychnophora ericoides Mart.	Shrub	Regional		Reflora
Piptocarpha angustifolia Dusén ex Malme	Tree	ESA		Reflora
Piptocarpha axillaris (Less.) Baker	Tree	Regional		Reflora
Piptocarpha densifolia Dusén ex G. Lom. Sm.	Tree	Regional		Reflora
Piptocarpha macropoda (DC.) Baker	Tree	ESA		Reflora
Piptocarpha oblonga (Gardner) Baker	Tree (L)	ESA		NeoTrop
Piptocarpha organensis Cabrera	Tree	Local		Reflora
Piptocarpha regnellii (Sch.Bip.) Cabrera	Tree	Regional		Reflora
Piptocarpha rotundifolia (Less.) Baker	Tree	SA		Reflora
Piptocarpha sellowii (Sch.Bip.) Baker	Tree	Regional		NeoTrop
Raulinoreitzia leptophlebia (B.L.Rob.) R.M.King & H.Rob.	Shrub	Local	VU	Reflora
Stifftia chrysantha J.C.Mikan	Tree (L)	Regional		Reflora
Stifftia fruticosa (Vell.) D.J.N.Hind & Semir	Tree	Local	VU	Reflora
Stifftia parviflora (Leandro) D.Don	Tree	Regional		Reflora

Family (Order) and species	Life form	Geog. distribut.	Threat status	Source
Symphyopappus itatiayensis (Hieron.) R.M.King & H.Rob.	Tree	ESA		Reflora
Symphyopappus lymansmithii B.L.Rob.	Shrub	ESA		Reflora
Trixis praestans (Vell.) Cabrera	Shrub	ESA		NeoTrop
Verbesina glabrata Hook. & Arn.	Shrub	ESA		Reflora
Vernonanthura beyrichii (Less.) H.Rob.	Shrub	Regional		Reflora
Vernonanthura discolor (Spreng.) H.Rob.	Tree	ESA		Reflora
Vernonanthura divaricata (Spreng.) H.Rob.	Tree	ESA		Reflora
Vernonanthura petiolaris (DC.) H.Rob.	Tree	ESA		Reflora
Vernonanthura phosphorica (Vell.) H.Rob.	Shrub	ESA		Reflora
Vernonanthura puberula (Less.) H.Rob.	Tree	ESA		NeoTrop
Wunderlichia mirabilis Riedel ex Baker	Tree	ESA		Reflora
BERBERIDACEAE (Ranunculales)				
Berberis laurina Billb.	Shrub	ESA		Reflora
BIGNONIACEAE (Lamiales)				
Cybistax antisyphilitica (Mart.) Mart.	Tree	SA		Reflora
Handroanthus albus (Cham.) Mattos	Tree	ESA		Reflora
Handroanthus botelhensis (A.H.Gentry) S.Grose	Tree	Neotrop.		Reflora
Handroanthus bureavii (Sandwith) S.Grose	Tree	Regional		Reflora
Handroanthus catarinensis (A.H.Gentry) S.Grose	Shrub	Regional		NeoTrop
Handroanthus chrysotrichus (Mart. ex DC.) Mattos	Tree	ESA		Reflora
Handroanthus heptaphyllus (Vell.) Mattos	Tree	ESA		Reflora
Handroanthus impetiginosus (Mart. ex DC.) Mattos	Tree	Neotrop.		Reflora
Handroanthus ochraceus (Cham.) Mattos	Tree	Neotrop.		Reflora
Handroanthus pulcherrimus (Sandwith) Mattos	Tree	Regional		Reflora
Handroanthus serratifolius (A.H.Gentry) S.Grose	Tree	SA		Reflora
Handroanthus umbellatus (Sond.) Mattos	Tree	ESA		Reflora
Handroanthus vellosoi (Toledo) Mattos	Tree	Regional		Reflora
Jacaranda caroba (Vell.) DC.	Shrub	ESA		Reflora

Family (Order) and species	Life form	Geog. distribut.	Threat status	Source
Jacaranda cuspidifolia Mart.	Tree	ESA		Reflora
Jacaranda macrantha Cham.	Tree	SA		Reflora
Jacaranda micrantha Cham.	Tree	ESA		Reflora
Jacaranda montana Morawetz	Tree	Local		Reflora
Jacaranda puberula Cham.	Tree	ESA		Reflora
Jacaranda pulcherrima Morawetz	Shrub	Regional		Reflora
Jacaranda subalpina Morawetz	Tree	Local	VU	Reflora
Sparattosperma leucanthum (Vell.) K.Schum.	Tree	SA		Reflora
Spathodea campanulata P. Beauv. (*)	Tree	exotic		Reflora
Tabebuia aurea (Silva Manso) Benth. & Hook.f. ex S.Moore	Tree	SA		Reflora
Tabebuia cassinoides (Lam.) DC.	Tree	Regional		Reflora
Tabebuia insignis (Miq.) Sandwith	Tree	SA		Reflora
Tabebuia obtusifolia (Cham.) Bureau	Tree	ESA		Reflora
Tabebuia roseoalba (Ridl.) Sandwith	Tree	SA		Reflora
Tecoma stans (L.) Juss. ex Kunth (*)	Tree	exotic		NeoTrop
Zeyheria montana Mart.	Shrub	ESA		Reflora
Zeyheria tuberculosa (Vell.) Bureau ex Verl.	Tree	ESA		Reflora
BIXACEAE (Malvales)				
Bixa arborea Huber	Tree	SA		Reflora
Bixa orellana L.	Tree	Neotrop.		Reflora
BORAGINACEAE (Boraginales)				
Cordia americana (L.) Gottschling & J.S.Mill.	Tree	Regional		Reflora
Cordia ecalyculata Vell.	Tree	ESA		Reflora
Cordia glabrata (Mart.) A.DC.	Tree	ESA		Reflora
Cordia magnoliifolia Cham.	Tree	Regional		Reflora
Cordia ochnacea DC.	Tree	Local		Reflora
Cordia sellowiana Cham.	Tree	SA		Reflora
Cordia silvestris Fresen.	Tree	ESA	VU	Reflora
Cordia superba Cham.	Tree	ESA		Reflora

Family (Order) and species	Life form	Geog. distribut.	Threat status	Source
Cordia taguahyensis Vell.	Tree	ESA		Reflora
Cordia tarodae M.Stapf	Tree	Local		Reflora
Cordia trichoclada DC.	Tree	Regional	VU	Reflora
Cordia trichotoma (Vell.) Arráb. ex Steud.	Tree	ESA		Reflora
Tournefortia bicolor Sw.	Tree	SA		Reflora
BURSERACEAE (Sapindales)				
Protium brasiliense (Spreng.) Engl.	Tree	ESA		Reflora
Protium heptaphyllum (Aubl.) Marchand	Tree	SA		Reflora
Protium kleinii Cuatrec.	Tree	Regional	VU	Reflora
Protium ovatum Engl.	Tree	ESA		Reflora
Protium spruceanum (Benth.) Engl.	Tree	SA		Reflora
Protium warmingianum Marchand	Tree	ESA		NeoTrop
Protium widgrenii Engl.	Tree	Regional		Reflora
CACTACEAE (Caryophyllales)				
Arthrocereus melanurus (K.Schum.) Diers et al.	Cactus	Local		NeoTrop
Brasiliopuntia brasiliensis (Willd.) A.Berger	Cactus	SA		Reflora
Cereus bicolor Rizzini & A.Mattos	Cactus	ESA		NeoTrop
Cereus fernambucensis Lem.	Cactus	ESA		Reflora
Cereus hildmannianus K.Schum.	Cactus	ESA		Reflora
Coleocephalocereus fluminensis (Miq.) Backeb.	Cactus	Regional		Reflora
Opuntia monacantha Haw.	Cactus	ESA		Reflora
Pereskia aculeata Mill.	Tree (L)	Neotrop.		Reflora
Pereskia grandifolia Haw.	Tree (L)	ESA		Reflora
Pilosocereus machrisii (E.Y.Dawson) Backeb.	Cactus	ESA	EN	Reflora
CALOPHYLLACEAE (Malpighiales)				
Calophyllum brasiliense Cambess.	Tree	Neotrop.		Reflora
Kielmeyera coriacea Mart. & Zucc.	Tree	SA		Reflora
Kielmeyera decipiens Saddi	Tree	Local		Reflora
Kielmeyera grandiflora (Wawra) Saddi	Tree	SA		Reflora
Kielmeyera lathrophyton Saddi	Tree	ESA		Reflora

Family (Order) and species	Life form	Geog. distribut.	Threat status	Source
Kielmeyera membranacea Casar.	Tree	Regional		Reflora
Kielmeyera rosea Mart. & Zucc.	Tree	ESA		NeoTrop
Kielmeyera rubriflora Cambess.	Tree	SA		Reflora
Kielmeyera variabilis Mart. & Zucc.	Tree	ESA		Reflora
CANELLACEAE (Canellales)				
Cinnamodendron dinisii Schwacke	Tree	Regional		Reflora
Cinnamodendron occhionianum F.Barros & J.Salazar	Tree	Local		Reflora
CANNABACEAE (Rosales)				
Celtis ehrenbergiana (Klotzsch) Liebm.	Tree	ESA		Reflora
Celtis fluminensis Carauta	Tree	Regional		Reflora
Celtis iguanaea (Jacq.) Sarg.	Tree	Neotrop.		Reflora
Celtis pubescens (Kunth) Spreng.	Tree (L)	SA		Reflora
Celtis spinosa Spreng.	Tree	ESA		Reflora
Trema micrantha (L.) Blume	Tree	Neotrop.		Reflora
CAPPARACEAE (Brassicales)				
Capparidastrum frondosum (Jacq.) Cornejo & Iltis	Tree	Neotrop.		Reflora
Crateva tapia L.	Tree	Neotrop.	VU	Reflora
Cynophalla flexuosa (L.) J.Presl	Tree	Neotrop.		NeoTrop
CARDIOPTERIDACEAE (Aquifoliales)				
Citronella gongonha (Mart.) R.A.Howard	Tree	ESA		Reflora
Citronella paniculata (Mart.) R.A.Howard	Tree	SA		Reflora
CARICACEAE (Brassicales)				
Carica papaya L. (*)	Shrub	Neotrop.		Reflora
Jacaratia heptaphylla (Vell.) A.DC.	Tree	ESA		Reflora
Jacaratia spinosa (Aubl.) A.DC.	Tree	Neotrop.		Reflora
Vasconcellea monoica (Desf.) A.DC.	Tree	Regional		Reflora
Vasconcellea quercifolia A.St.-Hil.	Tree	SA		Reflora
CARYOCARACEAE (Malpighiales)				
Caryocar brasiliense Cambess.	Tree	ESA		Reflora

Family (Order) and species	Life form	Geog. distribut.	Threat status	Source
CASUARINACEAE (Fagales)				
Casuarina equisetifolia L. (*)	Tree	exotic		NeoTrop
CELASTRACEAE (Celastrales)				
Cheiloclinium cognatum (Miers) A.C.Sm.	Tree	Neotrop.		Reflora
Maytenus aquifolia Mart.	Tree	ESA		Reflora
Maytenus ardisiaefolia Reissek	Tree	Local		Reflora
Maytenus boaria Molina	Tree	SA		NeoTrop
Maytenus brasiliensis Mart.	Tree	ESA	EN	Reflora
Maytenus cestrifolia Reissek	Tree	Local		Reflora
Maytenus communis Reissek	Tree	Local		NeoTrop
Maytenus dasyclada Mart.	Tree	ESA		Reflora
Maytenus evonymoides Reissek	Tree	ESA		Reflora
Maytenus floribunda Reissek	Tree	SA	CR	Reflora
Maytenus glaucescens Reissek	Tree	Regional		Reflora
Maytenus gonoclada Mart.	Tree	ESA		Reflora
Maytenus ilicifolia Mart. ex Reissek	Tree	ESA	EN	Reflora
Maytenus littoralis Carv.-Okano	Tree	Regional		Reflora
Maytenus obtusifolia Mart.	Tree	ESA		Reflora
Maytenus patens Reissek	Tree	ESA		NeoTrop
Maytenus schumanniana Loes.	Tree	Regional		Reflora
Maytenus subalata Reissek	Shrub	Regional		Reflora
Peritassa flaviflora A.C.Sm.	Tree	Regional		Reflora
Plenckia populnea Reissek	Tree	SA		Reflora
Salacia arborea (Schrank) Peyr.	Tree	ESA	EN	Reflora
Salacia crassifolia (Mart. ex Schult.) G.Don	Tree	SA		Reflora
Salacia elliptica (Mart. ex Schult.) G.Don	Tree	Neotrop.		Reflora
Salacia grandifolia (Mart. ex Schult.) G.Don	Tree (L)	Regional		Reflora
Tontelea leptophylla A.C.Sm.	Tree	Regional	VU	Reflora
Tontelea micrantha (Mart. ex Schult.) A.C.Sm.	Tree	SA		Reflora
Tontelea miersii (Peyr.) A.C.Sm.	Tree	ESA		Reflora

Family (Order) and species	Life form	Geog. distribut.	Threat status	Source
Tontelea tenuicula (Miers) A.C.Sm.	Tree	ESA		Reflora
CHLORANTHACEAE (Chloranthales)				
Hedyosmum brasiliense Mart. ex Miq.	Tree	ESA		Reflora
CHRYSOBALANACEAE (Malpighiales)				
Chrysobalanus icaco L.	Tree	Pantrop.		Reflora
Couepia grandiflora (Mart. & Zucc.) Benth.	Tree	SA		Reflora
Couepia leitaofilhoi Prance	Tree	Local	EN	Reflora
Couepia meridionalis Prance	Tree	Local	EX	Reflora
Couepia monteclarensis Prance	Tree	Regional		Reflora
Couepia uiti (Mart. & Zucc.) Benth. ex Hook.f.	Tree	SA		Reflora
Couepia venosa Prance	Tree	Regional		Reflora
Hirtella angustifolia Schott ex Spreng.	Tree	Regional		Reflora
Hirtella glandulosa Spreng.	Tree	SA		NeoTrop
Hirtella glaziovii Taub.	Tree	Local		Reflora
Hirtella gracilipes (Hook.f.) Prance	Tree	SA		Reflora
Hirtella hebeclada Moric. ex DC.	Tree	ESA		Reflora
Hirtella racemosa Lam.	Shrub	Neotrop.	EN	NeoTrop
Licania gardneri (Hook.f.) Fritsch	Tree	SA	EN	Reflora
Licania hoehnei Pilg.	Tree	ESA		Reflora
Licania humilis Cham. & Schltdl.	Tree	SA		Reflora
Licania indurata Pilg.	Tree	Local	EN	Reflora
Licania kunthiana Hook.f.	Tree	SA		Reflora
Licania nitida Hook.f.	Tree	ESA		Reflora
Licania octandra (Hoffmanns. ex Roem. & Schult.) Kuntze	Tree	SA		Reflora
Licania rigida Benth.	Tree	Neotrop.		Reflora
Licania tomentosa (Benth.) Fritsch	Tree	ESA		Reflora
Parinari excelsa Sabine	Tree	SA		NeoTrop
Parinari obtusifolia Hook.f.	Shrub	ESA		Reflora
CLETHRACEAE (Ericales)				

Family (Order) and species	Life form	Geog. distribut.	Threat status	Source
Clethra scabra Pers.	Tree	ESA		Reflora
CLUSIACEAE (Malpighiales)				
Clusia criuva Cambess.	Tree	ESA		Reflora
Clusia grandiflora Splitg.	Tree	SA		Reflora
Clusia lanceolata Cambess.	Tree	Local		Reflora
Clusia organensis Planch. & Triana	Tree	Regional		Reflora
Garcinia gardneriana (Planch. & Triana) Zappi	Tree	SA		Reflora
Tovomitopsis paniculata (Spreng.) Planch. & Triana	Tree	Regional		Reflora
Tovomitopsis saldanhae Engl.	Tree	Regional		Reflora
COMBRETACEAE (Myrtales)				
Buchenavia hoehneana N.F.Mattos	Tree	Regional		Reflora
Buchenavia kleinii Exell	Tree	ESA		Reflora
Buchenavia parvifolia Ducke	Tree	SA		Reflora
Buchenavia tomentosa Eichler	Tree	SA		Reflora
Combretum duarteanum Cambess.	Tree (L)	ESA		Reflora
Combretum laxum Jacq.	Shrub (L)	SA		Reflora
Conocarpus erectus L.	Tree	Pantrop.		Reflora
Laguncularia racemosa (L.) C.F.Gaertn.	Tree	Pantrop.		Reflora
Terminalia argentea Mart.	Tree	ESA		Reflora
Terminalia catappa L. (*)	Tree	Pantrop.		Reflora
Terminalia fagifolia Mart.	Tree	ESA		NeoTrop
Terminalia glabrescens Mart.	Tree	ESA		Reflora
Terminalia januariensis DC.	Tree	ESA		Reflora
Terminalia phaeocarpa Eichler	Tree	ESA		Reflora
Terminalia triflora (Griseb.) Lillo	Tree	ESA		Reflora
CONNARACEAE (Oxalidales)				
Bernardinia fluminensis (Gardner) Planch.	Tree (L)	Regional		Reflora
Connarus regnellii G.Schellenb.	Tree	Regional		Reflora
Connarus rostratus (Vell.) L.B.Sm.	Tree	Regional		Reflora

Family (Order) and species	Life form	Geog. distribut.	Threat status	Source
Connarus suberosus Planch.	Tree	ESA		Reflora
Rourea induta Planch.	Shrub	ESA		Reflora
Rourea psammophila Forero	Tree	ESA	EN	Reflora
CUNONIACEAE (Oxalidales)				
Lamanonia chabertii (Pamp.) L.B.Sm.	Tree	Local	VU	Reflora
Lamanonia cuneata (Cambess.) Kuntze	Shrub	Regional	EN	Reflora
Lamanonia grandistipularis (Taub.) Taub.	Tree	Regional		NeoTrop
Lamanonia ternata Vell.	Tree	ESA		Reflora
Weinmannia discolor Gardner	Tree	ESA		Reflora
Weinmannia humilis Engl.	Tree	ESA		Reflora
Weinmannia organensis Gardner	Shrub	Local	VU	Reflora
Weinmannia paulliniifolia Pohl ex Ser.	Tree	ESA		Reflora
Weinmannia pinnata L.	Tree	Neotrop.		Reflora
CYATHEACEAE (Cyatheales)				
Alsophila capensis (L.f.) J.Sm.	Fern	ESA	EN	Reflora
Alsophila setosa Kaulf.	Fern	ESA		Reflora
Alsophila sternbergii (Sternb.) D.S.Conant	Fern	ESA		Reflora
Cyathea atrovirens (Langsd. & Fisch.) Domin	Fern	ESA		Reflora
Cyathea corcovadensis (Raddi) Domin	Fern	ESA		Reflora
Cyathea delgadii Sternb.	Fern	Neotrop.		Reflora
Cyathea dichromatolepis (Fée) Domin	Fern	Regional		Reflora
Cyathea gardneri Hook.	Fern	ESA		Reflora
Cyathea glaziovii (Fée) Domin	Fern	ESA	VU	Reflora
Cyathea hirsuta C.Presl	Fern	Regional		Reflora
Cyathea leucofolis Domin	Fern	ESA		Reflora
Cyathea microdonta (Desv.) Domin	Fern	Neotrop.		Reflora
Cyathea phalerata Mart.	Fern	ESA		Reflora
Cyathea praecincta (Kunze) Domin	Fern	ESA		Reflora
Cyathea pungens (Willd.) Domin	Fern	Neotrop.	VU	Reflora
Cyathea rufa (Fée) Lellinger	Fern	Regional		Reflora

Family (Order) and species	Life form	Geog. distribut.	Threat status	Source
Cyathea villosa Willd.	Fern	Neotrop.		Reflora
DICHAPETALACEAE (Malpighiales)				
Stephanopodium blanchetianum Baill.	Tree	Regional		NeoTrop
Stephanopodium estrellense Baill.	Tree	Local		NeoTrop
Stephanopodium organense (Rizzini) Prance	Tree	Local		NeoTrop
Stephanopodium sessile Rizzini	Tree	Local		NeoTrop
DICKSONIACEAE (Cyatheales)				
Dicksonia sellowiana Hook.	Fern	Neotrop.	VU	Reflora
DILLENIACEAE (Dilleniales)				
Curatella americana L.	Tree	Neotrop.		NeoTrop
Davilla elliptica A.St.-Hil.	Shrub (L)	SA		Reflora
Davilla grandiflora A.St.-Hil.	Shrub (L)	SA		Reflora
EBENACEAE (Ericales)				
Diospyros brasiliensis Mart. ex Miq.	Tree	Local		Reflora
Diospyros hispida A.DC.	Tree	SA		Reflora
Diospyros inconstans Jacq.	Tree	Neotrop.		Reflora
ELAEOCARPACEAE (Oxalidales)				
Sloanea garckeana K.Schum.	Tree	SA		Reflora
Sloanea guianensis (Aubl.) Benth.	Tree	Neotrop.		Reflora
Sloanea hirsuta (Schott) Planch. ex Benth.	Tree	ESA		Reflora
Sloanea lasiocoma K.Schum.	Tree	ESA		Reflora
Sloanea obtusifolia (Moric.) Schum.	Tree	Neotrop.		Reflora
Sloanea petalata D.Sampaio e V.C.Souza	Tree	Local		Reflora
Sloanea terniflora (DC.) Standl.	Tree	Neotrop.		Reflora
ERICACEAE (Ericales)				
Agarista coriifolia (Thunb.) Hook. ex Nied.	Shrub	Regional		Reflora
Agarista eucalyptoides (Cham. & Schltdl.) G.Don	Tree	ESA		Reflora
Agarista niederleinii (Sleumer) Judd	Tree	Regional	EN	Reflora
Agarista oleifolia (Cham.) G.Don	Tree	ESA		Reflora

Family (Order) and species	Life form	Geog. distribut.	Threat status	Source
Agarista pulchra (Cham. & Schltdl.) G.Don	Shrub	Regional	EN	Reflora
Gaultheria eriophylla (Pers.) Sleumer ex Burtt	Shrub	SA		Reflora
Gaylussacia brasiliensis (Spreng.) Meisn.	Shrub	ESA		Reflora
Gaylussacia pseudogaultheria Cham. & Schltdl.	Tree	ESA		Reflora
Gaylussacia rhododendron Cham. & Schltdl.	Shrub	Regional	VU	Reflora
ERYTHROXYLACEAE (Malpighiales)				
Erythroxylum ambiguum Peyr.	Shrub	ESA		Reflora
Erythroxylum amplifolium (Mart.) O.E.Schulz	Tree	ESA		Reflora
Erythroxylum anguifugum Mart.	Tree	SA		Reflora
Erythroxylum argentinum O.E.Schulz	Tree	ESA		Reflora
Erythroxylum buxus Peyr.	Shrub	ESA		Reflora
Erythroxylum campestre A.St.-Hil.	Shrub	ESA		Reflora
Erythroxylum citrifolium A.St.-Hil.	Tree	Neotrop.		Reflora
Erythroxylum coelophlebium Mart.	Tree	Regional	VU	Reflora
Erythroxylum cuneifolium (Mart.) O.E.Schulz	Tree	ESA		Reflora
Erythroxylum cuspidifolium Mart.	Tree	ESA		Reflora
Erythroxylum daphnites Mart.	Tree	SA		Reflora
Erythroxylum deciduum A.St.-Hil.	Tree	SA		Reflora
Erythroxylum frangulifolium A.St.-Hil.	Shrub	Regional		Reflora
Erythroxylum gonocladum (Mart.) O.E.Schulz	Shrub	ESA		NeoTrop
Erythroxylum myrsinites Mart.	Shrub	ESA	VU	Reflora
Erythroxylum pelleterianum A.St.-Hil.	Tree	ESA		Reflora
Erythroxylum pulchrum A.St.-Hil.	Tree	ESA		Reflora
Erythroxylum suberosum A.St.-Hil.	Shrub	SA		Reflora
Erythroxylum subracemosum Turcz.	Shrub	SA		Reflora
Erythroxylum tortuosum Mart.	Shrub	SA		Reflora
Erythroxylum vacciniifolium Mart.	Tree	ESA		Reflora
ESCALLONIACEAE (Escalloniales)				
Escallonia bifida Link & Otto	Tree	ESA		Reflora
Escallonia chlorophylla Cham. & Schltdl.	Shrub	Regional	EN	Reflora

Family (Order) and species	Life form	Geog. distribut.	Threat status	Source
Escallonia hispida (Vell.) Sleumer	Shrub	Local		Reflora
Escallonia megapotamica Spreng.	Shrub	ESA		Reflora
EUPHORBIACEAE (Malpighiales)				
Acalypha macrostachya Jacq.	Tree	SA		Reflora
Acalypha villosa Jacq.	Tree	ESA		Reflora
Actinostemon concepcionis (Chodat & Hassl.) Hochr.	Tree	ESA		NeoTrop
Actinostemon concolor (Spreng.) Müll.Arg.	Tree	Neotrop.		Reflora
Actinostemon klotzschii (Didr.) Pax	Tree	ESA		Reflora
Actinostemon lasiocarpus (Müll.Arg.) Baill.	Tree	ESA		NeoTrop
Actinostemon leptopus (Müll.Arg.) Pax	Tree	Local		Reflora
Actinostemon verticillatus (Klotzsch) Baill.	Tree	ESA		Reflora
Adelia membranifolia (Müll.Arg.) Chodat & Hassl.	Tree	ESA		Reflora
Alchornea glandulosa Poepp. & Endl.	Tree	Neotrop.		Reflora
Alchornea sidifolia Müll.Arg.	Tree	Regional		Reflora
Alchornea triplinervia (Spreng.) Müll.Arg.	Tree	Neotrop.		Reflora
Algernonia brasiliensis Baill.	Tree	Local		Reflora
Algernonia leandrii (Baill.) G.L.Webster	Tree	Local		Reflora
Algernonia riedelii (Müll.Arg.) G.L.Webster	Tree	Local		Reflora
Aparisthmium cordatum (A.Juss.) Baill.	Tree	Neotrop.		Reflora
Bernardia pulchella (Baill.) Müll.Arg.	Shrub	ESA		Reflora
Caryodendron janeirense Müll.Arg.	Tree	ESA		Reflora
Croton alchorneicarpus Croizat	Tree	Regional		Reflora
Croton campanulatus Caruzo & Cordeiro	Tree	Regional		NeoTrop
Croton celtidifolius Baill.	Tree	ESA		Reflora
Croton compressus Lam.	Shrub	Local	EN	Reflora
Croton floribundus Spreng.	Tree	ESA		Reflora
Croton gracilipes Baill.	Tree	Regional		Reflora
Croton hemiargyreus Müll.Arg.	Tree	ESA		Reflora
Croton macrobothrys Baill.	Tree	ESA		Reflora

Family (Order) and species	Life form	Geog. distribut.	Threat status	Source
Croton organensis Baill.	Tree	Regional		Reflora
Croton piptocalyx Müll.Arg.	Tree	Neotrop.		Reflora
Croton priscus Croizat	Tree	Local		Reflora
Croton rottlerifolius Baill.	Tree	Regional		Reflora
Croton salutaris Casar.	Tree	Regional		Reflora
Croton sphaerogynus Baill.	Shrub	Regional	EN	Reflora
Croton urucurana Baill.	Tree	SA		Reflora
Croton vulnerarius Baill.	Tree	Regional		Reflora
Euphorbia comosa Vell.	Tree	Regional		Reflora
Gymnanthes gaudichaudii Müll.Arg.	Tree	Regional		Reflora
Gymnanthes klotzschiana Müll.Arg.	Tree	ESA		Reflora
Gymnanthes schottiana Müll.Arg.	Shrub	ESA		Reflora
Jatropha curcas L. (*)	Tree	Neotrop.		Reflora
Joannesia princeps Vell.	Tree	ESA		Reflora
Mabea fistulifera Mart.	Tree	SA		Reflora
Mabea piriri Aubl.	Tree	Neotrop.		Reflora
Manihot anomala Pohl	Shrub (L)	ESA		Reflora
Manihot caerulescens Pohl	Tree	ESA		Reflora
Manihot grahamii Hook.	Tree	ESA		Reflora
Manihot pilosa Pohl	Tree	Regional		Reflora
Manihot tripartita (Spreng.) Müll.Arg.	Shrub	SA		Reflora
Maprounea brasiliensis A.St.-Hil.	Shrub	ESA		Reflora
Maprounea guianensis Aubl.	Tree	Neotrop.		Reflora
Micrandra elata (Didr.) Müll.Arg.	Tree	SA		Reflora
Ophthalmoblapton crassipes Müll.Arg.	Tree	Local		Reflora
Pachystroma longifolium (Nees) I.M.Johnst.	Tree	ESA		Reflora
Pausandra morisiana (Casar.) Radlk.	Tree	ESA		Reflora
Philyra brasiliensis Klotzsch	Tree	Regional		Reflora
Pleradenophora membranifolia (Müll.Arg.) Esser & A. L. Melo	Tree	SA		Reflora

Family (Order) and species	Life form	Geog. distribut.	Threat status	Source
Sapium glandulosum (L.) Morong	Tree	Neotrop.		Reflora
Sapium haematospermum Müll.Arg.	Tree	ESA		NeoTrop
Sapium sellowianum (Müll.Arg.) Klotzsch ex Baill.	Tree	Regional		Reflora
Sebastiania brasiliensis Spreng.	Tree	ESA		Reflora
Senefeldera verticillata (Vell.) Croizat	Tree	ESA		Reflora
Stillingia bodenbenderi (Kuntze) D.J.Rogers	Shrub	Regional		Reflora
Stillingia oppositifolia Baill. ex Müll.Arg.	Shrub	Regional		Reflora
Tetrorchidium dusenii Pax & K.Hoffm.	Tree	SA		Reflora
Tetrorchidium rubrivenium Poepp.	Tree	ESA		Reflora
FABACEAE (Fabales)				
Abarema brachystachya (DC.) Barneby & J.W.Grimes	Tree	ESA		Reflora
Abarema cochliacarpos (Gomes) Barneby & J.W.Grimes	Tree	ESA		Reflora
Abarema langsdorffii (Benth.) Barneby & J.W.Grimes	Tree	SA		Reflora
Acacia mearnsii De Wild. (*)	Tree	Neotrop.		NeoTrop
Albizia edwallii (Hoehne) Barneby & J.W.Grimes	Tree	SA		Reflora
Albizia inundata (Mart.) Barneby & J.W.Grimes	Tree	SA		Reflora
Albizia niopoides (Spruce ex Benth.) Burkart	Tree	Neotrop.		Reflora
Albizia pedicellaris (DC.) L.Rico	Tree	SA		Reflora
Albizia polycephala (Benth.) Killip ex Record	Tree	ESA		Reflora
Anadenanthera colubrina (Vell.) Brenan	Tree	SA		Reflora
Anadenanthera peregrina (L.) Speg.	Tree	Neotrop.		Reflora
Andira anthelmia (Vell.) Benth.	Tree	ESA		Reflora
Andira fraxinifolia Benth.	Tree	ESA		Reflora
Andira humilis Mart. ex Benth.	Tree	SA		Reflora
Andira inermis (W.Wright) DC.	Tree	Pantrop.		NeoTrop
Andira ormosioides Benth.	Tree	Regional		Reflora
Andira vermifuga (Mart.) Benth.	Tree	SA	EN	Reflora

Family (Order) and species	Life form	Geog. distribut.	Threat status	Source
Apuleia leiocarpa (Vogel) J.F.Macbr.	Tree	SA	EN	Reflora
Ateleia glazioveana Baill.	Tree	Regional		Reflora
Bauhinia brevipes Vogel	Tree	SA		Reflora
Bauhinia cheilantha (Bong.) Steud.	Tree	ESA		Reflora
Bauhinia forficata Link	Tree	ESA		Reflora
Bauhinia holophylla (Bong.) Steud.	Shrub	SA		Reflora
Bauhinia longifolia (Bong.) Steud.	Tree	SA		Reflora
Bauhinia pentandra (Bong.) D.Dietr.	Tree	ESA		Reflora
Bauhinia rufa (Bong.) Steud.	Tree	ESA		NeoTrop
Bauhinia ungulata L.	Tree	Neotrop.		Reflora
Bauhinia uruguayensis Benth.	Tree	Regional		NeoTrop
Bowdichia virgilioides Kunth	Tree	SA	VU	Reflora
Caesalpinia pulcherrima (L.) Sw. (*)	Tree	exotic		Reflora
Calliandra brevipes Benth.	Shrub	ESA		NeoTrop
Calliandra foliolosa Benth.	Tree	ESA		Reflora
Calliandra tweedii Benth.	Tree	ESA		Reflora
Cassia ferruginea (Schrad.) Schrad. ex DC.	Tree	ESA		Reflora
Cassia leptophylla Vogel	Tree	Regional		Reflora
Centrolobium robustum (Vell.) Mart. ex Benth.	Tree	Regional		Reflora
Centrolobium tomentosum Guillem. ex Benth.	Tree	ESA		Reflora
Chamaecrista apoucouita (Aubl.) H.S.Irwin & Barneby	Tree	SA		Reflora
Chamaecrista atroglandulosa (Taub. ex Harms) H.S.Irwin & Barneby	Tree	Local	EX	Reflora
Chamaecrista ensiformis (Vell.) H.S.Irwin & Barneby	Tree	SA		Reflora
Chloroleucon tenuiflorum (Benth.) Barneby & J.W.Grimes	Tree	SA		Reflora
Chloroleucon tortum (Mart.) Pittier	Tree	ESA		Reflora
Clitoria fairchildiana R.A.Howard (*)	Tree	exotic		Reflora
Copaifera langsdorffii Desf.	Tree	SA		Reflora

Family (Order) and species	Life form	Geog. distribut.	Threat status	Source
Copaifera lucens Dwyer	Tree	Regional		Reflora
Copaifera trapezifolia Hayne	Tree	Neotrop.		Reflora
Cyclolobium brasiliense Benth.	Tree	SA		Reflora
Dahlstedtia pentaphylla (Taub.) Burkart	Tree	Regional		Reflora
Dahlstedtia pinnata (Benth.) Malme	Tree	Regional		Reflora
Dalbergia brasiliensis Vogel	Tree	ESA		Reflora
Dalbergia ernest-ulei Hoehne	Tree	Regional		Reflora
Dalbergia foliolosa Benth.	Tree	ESA		Reflora
Dalbergia frutescens (Vell.) Britton	Tree (L)	SA		Reflora
Dalbergia glaziovii Harms	Tree	Local		NeoTrop
Dalbergia miscolobium Benth.	Tree	ESA		Reflora
Dalbergia nigra (Vell.) Allemão ex Benth.	Tree	ESA	EN	Reflora
Dalbergia villosa (Benth.) Benth.	Tree	ESA		Reflora
Dimorphandra exaltata Schott	Tree	Regional		Reflora
Dimorphandra mollis Benth.	Tree	SA		Reflora
Dipteryx alata Vogel	Tree	SA		Reflora
Diptychandra aurantiaca Tul.	Tree	SA		Reflora
Enterolobium contortisiliquum (Vell.) Morong	Tree	ESA		Reflora
Enterolobium gummiferum (Mart.) J.F.Macbr.	Tree	ESA		Reflora
Enterolobium timbouva Mart.	Tree	ESA		Reflora
Erythrina crista-galli L.	Tree	Neotrop.		Reflora
Erythrina dominguezii Hassl.	Tree	ESA		Reflora
Erythrina falcata Benth.	Tree	SA		Reflora
Erythrina speciosa Andrews	Tree	ESA		Reflora
Erythrina verna Vell.	Tree	ESA		Reflora
Exostyles godoyensis Soares-Silva & Mansano	Tree	Local		Reflora
Exostyles venusta Schott	Tree	ESA		Reflora
Holocalyx balansae Micheli	Tree	ESA		Reflora
Hymenaea courbaril L.	Tree	Neotrop.		Reflora
Hymenaea martiana Hayne	Tree	ESA	EN	Reflora

Family (Order) and species	Life form	Geog. distribut.	Threat status	Source
Hymenaea stigonocarpa Mart. ex Hayne	Tree	SA		Reflora
Hymenolobium janeirense Kuhlm.	Tree	ESA		Reflora
Inga barbata Benth.	Tree	SA		Reflora
Inga bullata Benth.	Tree	Local		Reflora
Inga capitata Desv.	Tree	Neotrop.		Reflora
Inga congesta T.D.Penn.	Shrub	Regional		NeoTrop
Inga cordistipula Mart.	Tree	Regional		Reflora
Inga edulis Mart.	Tree	SA		Reflora
Inga edwallii (Harms) T.D.Penn.	Tree	Regional		Reflora
Inga ingoides (Rich.) Willd.	Tree	Neotrop.		Reflora
Inga lanceifolia Benth.	Tree	Local		Reflora
Inga laurina (Sw.) Willd.	Tree	Neotrop.		Reflora
Inga lenticellata Benth.	Tree	Regional		Reflora
Inga marginata Willd.	Tree	Neotrop.		Reflora
Inga mendoncaei Harms	Tree	Local	VU	NeoTrop
Inga praegnans T.D.Penn.	Tree	Local	VU	Reflora
Inga schinifolia Benth.	Tree	Regional		Reflora
Inga sellowiana Benth.	Tree	Regional		Reflora
Inga sessilis (Vell.) Mart.	Tree	ESA		Reflora
Inga striata Benth.	Tree	SA		Reflora
Inga subnuda Salzm. ex Benth.	Tree	ESA		Reflora
Inga tenuis (Vell.) Mart.	Tree	Regional		NeoTrop
Inga thibaudiana DC.	Tree	Neotrop.		Reflora
Inga vera Willd.	Tree	Neotrop.		Reflora
Inga virescens Benth.	Tree	Regional		Reflora
Inga vulpina Mart. ex Benth.	Tree	ESA		Reflora
Leptolobium dasycarpum Vogel	Tree	SA		Reflora
Leptolobium elegans Vogel	Tree	ESA		Reflora
Leucaena leucocephala (Lam.) de Wit (*)	Tree	exotic		NeoTrop

Family (Order) and species	Life form	Geog. distribut.	Threat status	Source
Leucochloron incuriale (Vell.) Barneby & J.W.Grimes	Tree	Regional		Reflora
Lonchocarpus campestris Mart. ex Benth.	Tree	ESA		Reflora
Lonchocarpus cultratus (Vell.) A.M.G.Azevedo & H.C.Lima	Tree	SA		Reflora
Lonchocarpus filipes Benth.	Tree	Local		Reflora
Lonchocarpus latifolius (Willd.) DC.	Tree	Regional		Reflora
Lonchocarpus muehlbergianus Hassl.	Tree	ESA		Reflora
Lonchocarpus subglaucescens Mart. ex Benth.	Tree	Regional		Reflora
Luetzelburgia guaissara Toledo	Tree	Regional	VU	Reflora
Machaerium acutifolium Vogel	Tree	SA		Reflora
Machaerium brasiliense Vogel	Tree	ESA		Reflora
Machaerium cantarellianum Hoehne	Tree (L)	Regional		Reflora
Machaerium hatschbachii Rudd	Tree	Regional		Reflora
Machaerium hirtum (Vell.) Stellfeld	Tree	SA		Reflora
Machaerium incorruptibile (Vell.) Benth.	Tree	Regional		Reflora
Machaerium lanceolatum (Vell.) J.F.Macbr.	Tree (L)	SA		Reflora
Machaerium nyctitans (Vell.) Benth.	Tree	ESA		Reflora
Machaerium paraguariense Hassl.	Tree	ESA		Reflora
Machaerium scleroxylon Tul.	Tree	ESA		Reflora
Machaerium stipitatum Vogel	Tree	ESA		Reflora
Machaerium villosum Vogel	Tree	ESA		Reflora
Melanoxylon brauna Schott	Tree	ESA		Reflora
Mimosa bimucronata (DC.) Kuntze	Tree	SA		Reflora
Mimosa caesalpiniifolia Benth. (*)	Tree	SA		Reflora
Mimosa cubatanensis Hoehne	Tree	Regional		Reflora
Mimosa laticifera Rizzini & A.Mattos	Tree	ESA		Reflora
Mimosa micropteris Benth.	Shrub	Regional		Reflora
Mimosa myuros Barneby	Tree	Regional		Reflora
Mimosa pilulifera Benth.	Shrub	ESA		Reflora
Mimosa regnellii Benth.	Shrub	Regional		Reflora

Family (Order) and species	Life form	Geog. distribut.	Threat status	Source
Mimosa scabrella Benth.	Tree	ESA		Reflora
Mimosa tenuiflora (Willd.) Poir.	Tree	Neotrop.		NeoTrop
Myrocarpus frondosus Allemão	Tree	ESA		Reflora
Myrocarpus leprosus Pickel	Tree	Local		Reflora
Myroxylon peruiferum L.f.	Tree	SA	VU	Reflora
Ormosia arborea (Vell.) Harms	Tree	ESA		Reflora
Ormosia fastigiata Tul.	Tree	ESA		Reflora
Ormosia minor Vogel	Tree	Local		Reflora
Parapiptadenia rigida (Benth.) Brenan	Tree	ESA		Reflora
Parkinsonia aculeata L. (*)	Tree	Neotrop.		Reflora
Peltogyne confertiflora (Mart. ex Hayne) Benth.	Tree	ESA	EN	Reflora
Peltophorum dubium (Spreng.) Taub.	Tree	Neotrop.		Reflora
Piptadenia gonoacantha (Mart.) J.F.Macbr.	Tree	ESA		Reflora
Piptadenia loefgreniana Hoehne	Tree	Local		Reflora
Piptadenia paniculata Benth.	Tree	SA		Reflora
Pithecellobium dulce (Roxb.) Benth. (*)	Tree	Neotrop.		Reflora
Plathymenia reticulata Benth.	Tree	ESA		Reflora
Platycyamus regnellii Benth.	Tree	ESA		Reflora
Platymiscium floribundum Vogel	Tree	ESA		Reflora
Platypodium elegans Vogel	Tree	Neotrop.		Reflora
Poecilanthe parviflora Benth.	Tree	ESA		Reflora
Pseudopiptadenia contorta (DC.) G.P.Lewis & M.P.Lima	Tree	SA		Reflora
Pseudopiptadenia leptostachya (Benth.) Rauschert	Tree	Regional		Reflora
Pseudopiptadenia warmingii (Benth.) G.P.Lewis & M.P.Lima	Tree	ESA		Reflora
Pterocarpus rohrii Vahl	Tree	ESA		Reflora
Pterodon emarginatus Vogel	Tree	SA		Reflora
Pterodon pubescens (Benth.) Benth.	Tree	ESA		Reflora
Pterogyne nitens Tul.	Tree	SA		Reflora
Riedeliella graciliflora Harms	Shrub	ESA		Reflora

Family (Order) and species	Life form	Geog. distribut.	Threat status	Source
Schizolobium parahyba (Vell.) Blake	Tree	Neotrop.		Reflora
Senegalia hatschbachii Seigler,Ebinger & P. G. Ribeiro	Tree	Regional		Reflora
Senegalia martii (Benth.) Seigler & Ebinger	Tree	Regional		Reflora
Senegalia polyphylla (DC.) Britton & Rose	Tree	Neotrop.		Reflora
Senegalia tenuifolia (L.) Britton & Rose	Tree	Neotrop.		Reflora
Senna alata (L.) Roxb.	Shrub	SA		Reflora
Senna cana (Nees & Mart.) H.S.Irwin & Barneby	Tree	ESA		Reflora
Senna corymbosa (Lam.) H.S.Irwin & Barneby	Tree	ESA		Reflora
Senna itatiaiae H.S.Irwin & Barneby	Shrub	Regional		NeoTrop
Senna macranthera (DC. ex Collad.) H.S.Irwin & Barneby	Tree	SA		Reflora
Senna multijuga (Rich.) H.S.Irwin & Barneby	Tree	Neotrop.		Reflora
Senna oblongifolia (Vogel) H.S.Irwin & Barneby	Tree	ESA		NeoTrop
Senna organensis (Glaz. ex Harms) H.S.Irwin & Barneby	Shrub	ESA		NeoTrop
Senna pendula (Humb.& Bonpl.ex Willd.) H.S.Irwin & Barneby	Tree	Neotrop.		Reflora
Senna polyphylla (Jacq.) H.S.Irwin & Barneby	Tree	ESA		Reflora
Senna rugosa (G.Don) H.S.Irwin & Barneby	Shrub	SA		Reflora
Senna siamea (Lam.) H.S.Irwin & Barneby (*)	Tree	exotic		Reflora
Senna silvestris (Vell.) H.S.Irwin & Barneby	Tree	SA		Reflora
Senna spectabilis (DC.) H.S.Irwin & Barneby	Tree	Neotrop.		NeoTrop
Senna splendida (Vogel) H.S.Irwin & Barneby	Shrub (L)	ESA		Reflora
Senna trachypus (Benth.) H.S.Irwin & Barneby	Shrub	ESA		Reflora
Senna velutina (Vogel) H.S.Irwin & Barneby	Shrub	ESA		Reflora
Sesbania sesban (L.) Merr. (*)	Shrub	exotic		Reflora
Sophora tomentosa L.	Shrub	ESA		Reflora
Stryphnodendron adstringens (Mart.) Coville	Tree	ESA		Reflora
Stryphnodendron polyphyllum Mart.	Tree	ESA		NeoTrop
Stryphnodendron rotundifolium Mart.	Tree	SA		Reflora

Family (Order) and species	Life form	Geog. distribut.	Threat status	Source
Swartzia flaemingii Raddi	Tree	ESA	EN	Reflora
Swartzia langsdorffii Raddi	Tree	Regional		Reflora
Swartzia myrtifolia Sm.	Tree	Neotrop.		NeoTrop
Swartzia oblata R.S.Cowan	Tree	ESA		NeoTrop
Swartzia simplex (Sw.) Spreng.	Tree	Neotrop.	VU	Reflora
Swartzia submarginata (Benth.) Mansano	Tree	Regional		Reflora
Sweetia fruticosa Spreng.	Tree	ESA		Reflora
Tachigali aurea Tul.	Tree	ESA		Reflora
Tachigali denudata (Vogel) Oliveira-Filho	Tree	ESA		Reflora
Tachigali duckei (Dwyer) Oliveira-Filho	Tree	Local		NeoTrop
Tachigali friburgensis (Harms) L.G.Silva & H.C.Lima	Tree	Regional		Reflora
Tachigali paratyensis (Vell.) H.C.Lima	Tree	ESA		Reflora
Tachigali pilgeriana (Harms) Oliveira-Filho	Tree	Regional		Reflora
Tachigali rubiginosa (Mart. ex Tul.) Oliveira-Filho	Tree	ESA		NeoTrop
Tachigali rugosa (Mart. ex Benth.) Zarucchi & Pipoly	Tree	Neotrop.		Reflora
Tachigali vulgaris L.G.Silva & H.C.Lima	Tree	SA		Reflora
Tipuana tipu (Benth.) Kuntze (*)	Tree	SA		Reflora
Vatairea heteroptera (Allemão) Ducke	Tree	ESA		Reflora
Vatairea macrocarpa (Benth.) Ducke	Tree	SA		Reflora
Zollernia glabra (Spreng.) Yakovlev	Tree	Local	EN	Reflora
Zollernia ilicifolia (Brongn.) Vogel	Tree	SA		Reflora
Zygia latifolia (L.) Fawc. & Rendle	Tree	Neotrop.		Reflora
Zygia selloi (Benth.) L.Rico	Tree	SA		Reflora
GRISELINIACEAE (Apiales)				
Griselinia ruscifolia (Clos) Taub.	Shrub (H)	SA		Reflora
HUMIRIACEAE (Malpighiales)				
Humiriastrum dentatum (Casar.) Cuatrec.	Tree	Regional		Reflora

Family (Order) and species	Life form	Geog. distribut.	Threat status	Source
Humiriastrum glaziovii (Urb.) Cuatrec.	Tree	Local		Reflora
Sacoglottis mattogrossensis Malme	Tree	SA		Reflora
Vantanea compacta (Schnizl.) Cuatrec.	Tree	ESA		NeoTrop
HYPERICACEAE (Malpighiales)				
Vismia brasiliensis Choisy	Tree	Local		Reflora
Vismia martiana Mart.	Tree	ESA	CR	Reflora
Vismia micrantha A.St.-Hil.	Tree	Regional		Reflora
LACISTEMATACEAE (Malpighiales)				
Lacistema aggregatum (P.J.Bergius) Rusby	Tree	Neotrop.		Reflora
Lacistema hasslerianum Chodat	Tree	ESA		Reflora
Lacistema lucidum Schnizl.	Tree	Local		Reflora
Lacistema pubescens Mart.	Tree	SA		NeoTrop
Lacistema serrulatum Mart.	Shrub	Regional		Reflora
LAMIACEAE (Lamiales)				
Aegiphila brachiata Vell.	Tree	ESA		Reflora
Aegiphila fluminensis Vell.	Tree	ESA		Reflora
Aegiphila graveolens Mart. & Schauer	Tree	ESA		Reflora
Aegiphila integrifolia (Jacq.) Moldenke	Tree	SA		Reflora
Aegiphila luschnathii Schauer	Tree	ESA		Reflora
Aegiphila mediterranea Vell.	Tree	ESA		Reflora
Aegiphila obducta Vell.	Tree	ESA		Reflora
Aegiphila verticillata Vell.	Shrub	SA		Reflora
Aegiphila vitelliniflora Walp.	Shrub	SA		Reflora
Cyanocephalus lippioides (Pohl ex Benth.) Harley & J.F.B.Pastore	Tree	Local		Reflora
Hyptidendron canum (Pohl ex Benth.) Harley	Tree	ESA		Reflora
Vitex agnus-castus L. (*)	Tree	exotic		Reflora
Vitex cymosa Bertero ex Spreng.	Tree	Neotrop.		Reflora
Vitex megapotamica (Spreng.) Moldenke	Tree	ESA		Reflora
Vitex mexiae Moldenke	Tree	Regional		Reflora

Family (Order) and species	Life form	Geog. distribut.	Threat status	Source
Vitex polygama Cham.	Tree	SA		Reflora
Vitex sellowiana Cham.	Tree	SA		Reflora
Vitex vauthieri DC. ex Schauer	Tree	SA		Reflora
LAURACEAE (Laurales)				
Aiouea acarodomatifera Kosterm.	Tree	Regional	VU	Reflora
Aiouea bracteata Kosterm.	Tree	Local	VU	Reflora
Aiouea piauhyensis (Meisn.) Mez	Tree	ESA	EN	Reflora
Aiouea saligna Meisn.	Tree	ESA		Reflora
Aiouea trinervis Meisn.	Tree	ESA	EN	Reflora
Aniba firmula (Nees & Mart.) Mez	Tree	ESA		Reflora
Aniba heringeri Vattimo-Gil	Tree	ESA	CR	Reflora
Aniba viridis Mez	Tree	ESA		Reflora
Beilschmiedia emarginata (Meisn.) Kosterm.	Tree	Regional		Reflora
Beilschmiedia fluminensis Kosterm.	Tree	Local		Reflora
Cinnamomum hatschbachii Vattimo-Gil	Tree	Regional		NeoTrop
Cinnamomum hirsutum Lorea-Hern.	Tree	Local		NeoTrop
Cinnamomum pseudoglaziovii Lorea-Hern.	Tree	Regional		NeoTrop
Cinnamomum sellowianum (Nees & Mart.) Kosterm.	Tree	Regional		Reflora
Cinnamomum stenophyllum (Meisn.) Vattimo-Gil	Tree	Regional		Reflora
Cinnamomum triplinerve (Ruiz & Pav.) Kosterm.	Tree	Neotrop.		Reflora
Cryptocarya aschersoniana Mez	Tree	Neotrop.		Reflora
Cryptocarya botelhensis P.L.R.Moraes	Tree	Local		Reflora
Cryptocarya mandioccana Meisn.	Tree	ESA		Reflora
Cryptocarya micrantha Meisn.	Tree	Regional		Reflora
Cryptocarya moschata Nees & Mart.	Tree	ESA		Reflora
Cryptocarya saligna Mez	Tree	Regional		Reflora
Cryptocarya subcorymbosa Mez	Tree	Local		Reflora
Endlicheria paniculata (Spreng.) J.F.Macbr.	Tree	Neotrop.		Reflora
Licaria armeniaca (Nees) Kosterm.	Tree	Neotrop.		Reflora

Family (Order) and species	Life form	Geog. distribut.	Threat status	Source
Nectandra angustifolia (Schrad.) Nees	Tree	ESA	EN	Reflora
Nectandra barbellata Coe-Teix.	Tree	Local		Reflora
Nectandra cissiflora Nees	Tree	Neotrop.	EN	Reflora
Nectandra cuspidata Nees	Tree	SA		Reflora
Nectandra debilis Mez	Tree	Regional	EN	Reflora
Nectandra grandiflora Nees	Tree	ESA		Reflora
Nectandra hihua (Ruiz & Pav.) Rohwer	Tree	Neotrop.	EN	Reflora
Nectandra lanceolata Nees	Tree	ESA		Reflora
Nectandra leucantha Nees	Tree	ESA		Reflora
Nectandra megapotamica (Spreng.) Mez	Tree	ESA		Reflora
Nectandra membranacea (Sw.) Griseb.	Tree	Neotrop.		Reflora
Nectandra nitidula Nees	Tree	ESA		Reflora
Nectandra oppositifolia Nees	Tree	Neotrop.		Reflora
Nectandra paranaensis Coe-Teix.	Tree	Local	EN	Reflora
Nectandra psammophila Nees	Tree	ESA		Reflora
Nectandra puberula (Schott) Nees	Tree	ESA		Reflora
Nectandra reticulata (Ruiz & Pav.) Mez	Tree	SA		Reflora
Ocotea aciphylla (Nees & Mart.) Mez	Tree	SA		Reflora
Ocotea basicordatifolia Vattimo-Gil	Tree	Local	EN	Reflora
Ocotea beulahiae J.B. Baitello	Tree	Local	EN	Reflora
Ocotea beyrichii (Nees) Mez	Tree	Regional	CR	Reflora
Ocotea bicolor Vattimo-Gil	Tree	Regional		Reflora
Ocotea brachybotrya (Meisn.) Mez	Tree	ESA		Reflora
Ocotea bragai Coe-Teix.	Tree	Local	EN	Reflora
Ocotea cantareirae Vattimo-Gil	Tree	Local		Reflora
Ocotea catharinensis Mez	Tree	SA		Reflora
Ocotea corymbosa (Meisn.) Mez	Tree	ESA		Reflora
Ocotea curucutuensis J.B. Baitello	Tree	Local	VU	Reflora
Ocotea daphnifolia (Meisn.) Mez	Tree	ESA	EN	Reflora
Ocotea diospyrifolia (Meisn.) Mez	Tree	ESA		Reflora

Family (Order) and species	Life form	Geog. distribut.	Threat status	Source
Ocotea dispersa (Nees & Mart.) Mez	Tree	ESA		Reflora
Ocotea divaricata (Nees) Mez	Tree	Regional		Reflora
Ocotea elegans Mez	Tree	ESA		Reflora
Ocotea estrellensis (Meisn.) P.L.R.Moraes	Tree	Regional		Reflora
Ocotea felix Coe-Teix.	Tree	Local	EN	Reflora
Ocotea frondosa (Meisn.) Mez	Tree	Regional	VU	Reflora
Ocotea glauca (Nees & Mart.) Mez	Tree	ESA		Reflora
Ocotea glaziovii Mez	Tree	ESA		Reflora
Ocotea indecora (Schott) Mez	Tree	ESA		Reflora
Ocotea inhauba Coe-Teixeira	Tree	Local	EX	Reflora
Ocotea insignis Mez	Tree	Regional		NeoTrop
Ocotea lanata (Nees & Mart.) Mez	Tree	Regional		Reflora
Ocotea lancifolia (Schott) Mez	Tree	ESA		Reflora
Ocotea laxa (Nees) Mez	Tree	ESA		Reflora
Ocotea lobbii (Meisn.) Rohwer	Tree	ESA		Reflora
Ocotea mandioccana A.Quinet	Tree	ESA		NeoTrop
Ocotea minarum (Nees & Mart.) Mez	Tree	ESA		Reflora
Ocotea mosenii Mez	Tree	Local	CR	Reflora
Ocotea nectandrifolia Mez	Tree	ESA	VU	Reflora
Ocotea notata (Nees & Mart.) Mez	Tree	ESA		NeoTrop
Ocotea nunesiana (Vattimo-Gil) J.B. Baitello	Tree	Regional	VU	Reflora
Ocotea nutans (Nees) Mez	Tree	ESA		Reflora
Ocotea odorifera (Vell.) Rohwer	Tree	ESA		Reflora
Ocotea paranapiacabensis Coe-Teixeira	Tree	Local		Reflora
Ocotea porosa (Nees & Mart.) Barroso	Tree	Regional		Reflora
Ocotea prolifera (Nees & Mart.) Mez	Tree	ESA		Reflora
Ocotea puberula (Rich.) Nees	Tree	SA		Reflora
Ocotea pulchella (Nees & Mart.) Mez	Tree	ESA		Reflora
Ocotea pulchra Vattimo-Gil	Tree	ESA		Reflora
Ocotea serrana Coe-Teix.	Tree	Local	EN	Reflora

Family (Order) and species	Life form	Geog. distribut.	Threat status	Source
Ocotea silvestris Vattimo-Gil	Tree	ESA		Reflora
Ocotea tabacifolia (Meisn.) Rohwer	Tree	SA	VU	Reflora
Ocotea teleiandra (Meisn.) Mez	Tree	ESA		Reflora
Ocotea tristis (Nees & Mart.) Mez	Tree	ESA		Reflora
Ocotea vaccinioides (Meisn.) Mez	Tree	ESA	EN	Reflora
Ocotea velloziana (Meisn.) Mez	Tree	ESA		Reflora
Ocotea velutina (Nees) Rohwer	Tree	ESA		Reflora
Ocotea venulosa (Nees) Baitello	Tree	Local		Reflora
Ocotea virgultosa (Nees) Mart.	Tree	Local		Reflora
Persea alba Nees & Mart.	Tree	ESA		Reflora
Persea americana Mill. (*)	Tree	exotic		Reflora
Persea fulva L.E.Kopp	Tree	ESA		NeoTrop
Persea major (Meisn.) L.E.Kopp	Tree	ESA		Reflora
Persea obovata Nees & Mart.	Tree	Local	VU	Reflora
Persea punctata Meisn.	Tree	Regional	CR	Reflora
Persea rigida Nees & Mart.	Tree	Local	EN	Reflora
Persea venosa Nees & Mart.	Tree	Regional		Reflora
Persea willdenovii Kosterm.	Tree	ESA		Reflora
Rhodostemonodaphne macrocalyx (Meisn.) Rohwer ex Madriñán	Tree	ESA		Reflora
Urbanodendron bahiense (Meisn.) Rohwer	Tree	Regional	VU	Reflora
LAXMANNIACEAE (Asparagales)				
Cordyline spectabilis Kunth & Bouché	Tree	ESA		Reflora
LECYTHIDACEAE (Ericales)				
Cariniana estrellensis (Raddi) Kuntze	Tree	SA		Reflora
Cariniana legalis (Mart.) Kuntze	Tree	ESA		Reflora
Lecythis lanceolata Poir.	Tree	ESA		Reflora
Lecythis pisonis Cambess.	Tree	SA		Reflora
LOGANIACEAE (Gentianales)				
Strychnos bicolor Progel	Shrub	Regional	CR	Reflora

Family (Order) and species	Life form	Geog. distribut.	Threat status	Source
Strychnos brasiliensis Mart.	Tree	ESA		Reflora
Strychnos pseudoquina A.St.-Hil.	Tree	ESA		Reflora
LYTHRACEAE (Myrtales)				
Diplusodon virgatus Pohl	Tree	ESA		Reflora
Lafoensia pacari A.St.-Hil.	Tree	ESA		Reflora
Lafoensia vandelliana Cham. & Schltdl.	Tree	SA		Reflora
MAGNOLIACEAE (Magnoliales)				
Magnolia ovata (A.St.-Hil.) Spreng.	Tree	ESA		Reflora
MALPIGHIACEAE (Malpighiales)				
Banisteriopsis latifolia (A.Juss.) B.Gates	Shrub	ESA		NeoTrop
Barnebya dispar (Griseb.) W.R.Anderson & B.Gates	Tree	Regional	VU	Reflora
Bunchosia maritima (Vell.) J.F.Macbr.	Tree	Regional		Reflora
Bunchosia pallescens Skottsb.	Tree	Regional	EN	Reflora
Byrsonima affinis W.R.Anderson	Tree	Regional		Reflora
Byrsonima basiloba A.Juss.	Tree	Regional		NeoTrop
Byrsonima coccolobifolia Kunth	Tree	SA		Reflora
Byrsonima crassifolia (L.) Kunth	Tree	Neotrop.		Reflora
Byrsonima cydoniifolia A.Juss.	Tree	ESA		Reflora
Byrsonima intermedia A.Juss.	Shrub	ESA		Reflora
Byrsonima laxiflora Griseb.	Tree	ESA		Reflora
Byrsonima ligustrifolia A.Juss.	Tree	ESA		Reflora
Byrsonima myricifolia Griseb.	Tree	Regional		Reflora
Byrsonima niedenzuiana Skottsb.	Shrub	Regional		Reflora
Byrsonima pachyphylla A.Juss.	Shrub	ESA		Reflora
Byrsonima perseifolia Griseb.	Tree	Regional		Reflora
Byrsonima salzmanniana A.Juss.	Tree	Regional		Reflora
Byrsonima sericea DC.	Tree	SA		Reflora
Byrsonima variabilis A.Juss.	Tree	ESA		Reflora
Byrsonima verbascifolia (L.) DC.	Tree	Neotrop.		Reflora

Family (Order) and species	Life form	Geog. distribut.	Threat status	Source
Heteropterys byrsonimifolia A.Juss.	Tree	SA		Reflora
MALVACEAE (Malvales)				
Akrosida macrophylla (Ulbr.) Fryxell & Fuertes	Tree	Regional	EN	Reflora
Apeiba tibourbou Aubl.	Tree	Neotrop.		Reflora
Bastardiopsis densiflora (Hook. & Arn.) Hassl.	Tree	SA		Reflora
Callianthe latipelata (G.L. Esteves & Krapov.) Donnell	Tree	Local		Reflora
Ceiba pubiflora (A.St.-Hil.) K.Schum.	Tree	ESA		Reflora
Ceiba speciosa (A.St.-Hil.) Ravenna	Tree	SA		Reflora
Christiana macrodon Toledo	Tree	Local	VU	Reflora
Eriotheca candolleana (K.Schum.) A.Robyns	Tree	ESA		Reflora
Eriotheca gracilipes (K.Schum.) A.Robyns	Tree	SA		Reflora
Eriotheca pentaphylla (Vell. & K.Schum.) A.Robyns	Tree	Local		Reflora
Eriotheca pubescens (Mart. & Zucc.) Schott & Endl.	Tree	ESA	EN	Reflora
Guazuma crinita Mart.	Tree	SA		Reflora
Guazuma ulmifolia Lam.	Tree	Neotrop.		Reflora
Helicteres brevispira A.St.-Hil.	Tree	SA		Reflora
Helicteres corylifolia Nees & Mart.	Shrub	SA		Reflora
Helicteres lhotzkyana (Schott & Endl.) K.Schum.	Tree	ESA		Reflora
Helicteres ovata Lam.	Tree	ESA		Reflora
Heliocarpus popayanensis Kunth	Tree	Neotrop.		Reflora
Luehea candicans Mart. & Zucc.	Tree	SA		Reflora
Luehea conwentzii K.Schum.	Tree	Regional	EN	Reflora
Luehea divaricata Mart. & Zucc.	Tree	ESA		Reflora
Luehea grandiflora Mart. & Zucc.	Tree	Neotrop.		Reflora
Luehea paniculata Mart. & Zucc.	Tree	SA		Reflora
Pachira calophylla (K.Schum.) Fern. Alonso	Tree	Local		Reflora
Pachira glabra Pasq. (*)	Tree	exotic		Reflora
Pavonia malacophylla (Link & Otto) Garcke	Shrub	Neotrop.		Reflora

Family (Order) and species	Life form	Geog. distribut.	Threat status	Source
Pseudabutilon aristulosum (K.Schum.) Krapov.	Tree	ESA		NeoTrop
Pseudobombax grandiflorum (Cav.) A.Robyns	Tree	ESA		Reflora
Pseudobombax longiflorum (Mart. & Zucc.) A.Robyns	Tree	ESA		Reflora
Pseudobombax marginatum (A.St.-Hil.) A. Robyns	Tree	SA	EN	Reflora
Pseudobombax tomentosum (Mart. & Zucc.) A.Robyns	Tree	ESA	VU	Reflora
Quararibea turbinata (Sw.) Poir.	Tree	Neotrop.		Reflora
Spirotheca rivieri (Decne.) Ulbr.	Tree (H)	SA	EN	Reflora
Sterculia curiosa (Vell.) Taroda	Tree	ESA		Reflora
Sterculia striata A.St.-Hil. & Naudin	Tree	ESA		Reflora
Talipariti pernambucense (Arruda) Bovini	Tree	Neotrop.		Reflora
Urena lobata L. (*)	Shrub	exotic		NeoTrop
MELASTOMATACEAE (Myrtales)				
Behuria insignis Cham.	Shrub	Local		Reflora
Graffenrieda weddellii Naudin	Shrub	SA		Reflora
Henriettea glabra (Vell.) Penneys, F.A. Michelangeli, Judd et Almeda	Tree	Regional		Reflora
Henriettea saldanhaei Cogn.	Tree	Regional		NeoTrop
Huberia laurina DC.	Tree	Local	EN	Reflora
Huberia nettoana Brade	Tree	Regional	VU	Reflora
Huberia ovalifolia DC.	Tree	Regional		Reflora
Huberia semiserrata DC.	Tree	Regional		Reflora
Leandra acutiflora (Naudin) Cogn.	Shrub	ESA		Reflora
Leandra amplexicaulis DC.	Shrub	ESA		Reflora
Leandra aurea (Cham.) Cogn.	Shrub	ESA		Reflora
Leandra barbinervis (Cham. ex Triana) Cogn.	Tree	ESA		Reflora
Leandra brackenridgei (A.Gray) Cogn.	Shrub	Local		Reflora
Leandra carassana (DC.) Cogn.	Tree	SA		Reflora
Leandra clidemioides (Naudin) Wurdack	Tree	SA		Reflora

Family (Order) and species	Life form	Geog. distribut.	Threat status	Source
Leandra fallax (Cham.) Cogn.	Shrub	ESA		Reflora
Leandra fragilis Cogn.	Shrub	ESA		Reflora
Leandra lancifolia Cogn.	Shrub	Regional		Reflora
Leandra melastomoides Raddi	Tree	ESA		Reflora
Leandra multiplinervis (Naudin) Cogn.	Shrub	Regional		Reflora
Leandra purpureovillosa Hoehne	Shrub	ESA		Reflora
Leandra quinquedentata (DC.) Cogn.	Shrub	ESA		Reflora
Leandra quinquenodis Cogn.	Tree	Regional		Reflora
Leandra regnellii (Triana) Cogn.	Shrub	ESA		Reflora
Leandra tetraquetra Cogn.	Tree	Regional		Reflora
Leandra tristis Cogn.	Tree	Regional		Reflora
Leandra truncata Baumgratz & D`El Rei Souza	Tree	Local		Reflora
Leandra umbellata DC.	Tree	Regional		Reflora
Leandra variabilis Raddi	Shrub	ESA		Reflora
Leandra vesiculosa Cogn.	Tree	Regional		Reflora
Macairea radula (Bonpl.) DC.	Shrub	SA		Reflora
Meriania calyptrata (Naudin) Triana	Tree	Local		Reflora
Meriania claussenii (Naudin) Triana	Tree	Regional		Reflora
Meriania paniculata (DC.) Triana	Tree	Local		Reflora
Miconia affinis DC.	Tree	Neotrop.		Reflora
Miconia albicans (Sw.) Triana	Shrub	Neotrop.		Reflora
Miconia atlantica Caddah & R.Goldenb.	Tree	Regional		Reflora
Miconia baumgratziana R.Goldenb. & C.V.Martin	Tree	Local		Reflora
Miconia brasiliensis (Spreng.) Triana	Tree	Regional		Reflora
Miconia brunnea DC.	Tree	ESA		Reflora
Miconia budlejoides Triana	Tree	ESA		Reflora
Miconia cabucu Hoehne	Tree	Regional		Reflora
Miconia calvescens DC.	Tree	Neotrop.		Reflora
Miconia castaneiflora Naudin	Tree	Regional		Reflora

Family (Order) and species	Life form	Geog. distribut.	Threat status	Source
Miconia chamissois Naudin	Shrub	Neotrop.		Reflora
Miconia chartacea Triana	Tree	ESA		Reflora
Miconia cinerascens Miq.	Tree	ESA		Reflora
Miconia cinnamomifolia (DC.) Naudin	Tree	ESA		Reflora
Miconia collatata Wurdack	Tree	SA		Reflora
Miconia corallina Spring	Tree	Local		Reflora
Miconia cubatanensis Hoehne	Tree	ESA		Reflora
Miconia discolor DC.	Tree	ESA		Reflora
Miconia dodecandra Cogn.	Tree	Neotrop.		Reflora
Miconia elegans Cogn.	Tree	SA		Reflora
Miconia fasciculata Gardner	Tree	ESA		Reflora
Miconia ferruginata DC.	Shrub	ESA		Reflora
Miconia holosericea (L.) DC.	Tree	Neotrop.		Reflora
Miconia hyemalis A.St.-Hil. & Naudin	Shrub	ESA		Reflora
Miconia ibaguensis (Bonpl.) Triana	Tree	SA		Reflora
Miconia inconspicua Miq.	Tree	Regional		Reflora
Miconia jucunda (DC.) Triana	Shrub	ESA		Reflora
Miconia latecrenata (DC.) Naudin	Tree	ESA		Reflora
Miconia lepidota DC.	Tree	SA		Reflora
Miconia leucocarpa DC.	Tree	ESA		Reflora
Miconia ligustroides (DC.) Naudin	Tree	ESA		Reflora
Miconia longicuspis Cogn.	Shrub	Regional		Reflora
Miconia lymanii Wurdack	Shrub	Regional		Reflora
Miconia macrothyrsa Benth.	Shrub	SA		Reflora
Miconia mendoncae Cogn.	Shrub	Local	VU	NeoTrop
Miconia minutiflora (Bonpl.) DC.	Tree	Neotrop.		Reflora
Miconia nervosa (Sm.) Triana	Tree	SA		Reflora
Miconia paniculata (DC.) Naudin	Tree	ESA		Reflora
Miconia paucidens DC.	Shrub	Regional		Reflora
Miconia pepericarpa DC.	Shrub	Regional		Reflora

Family (Order) and species	Life form	Geog. distribut.	Threat status	Source
Miconia petropolitana Cogn.	Tree	ESA		Reflora
Miconia picinguabensis R.Goldenb. & A.B.Martins	Tree	Local	EN	Reflora
Miconia polyandra Gardner	Shrub	Regional	VU	Reflora
Miconia prasina (Sw.) DC.	Tree	Neotrop.		Reflora
Miconia pusilliflora (DC.) Naudin	Tree	ESA		Reflora
Miconia racemifera (DC.) Triana	Tree	Regional		Reflora
Miconia robustissima Cogn.	Tree	Regional	VU	Reflora
Miconia rubiginosa (Bonpl.) DC.	Tree	Neotrop.		Reflora
Miconia sclerophylla Triana	Shrub	ESA		Reflora
Miconia sellowiana Naudin	Tree	ESA		Reflora
Miconia serrulata (DC.) Naudin	Tree	Neotrop.		Reflora
Miconia setosociliata Cogn.	Shrub	Regional		Reflora
Miconia shepherdii R.Goldenb. & Reginato	Tree	Regional		Reflora
Miconia speciosa (A.St.-Hil. & Naudin) Naudin	Tree	Regional		Reflora
Miconia stenostachya DC.	Shrub	Neotrop.		Reflora
Miconia tentaculifera Naudin	Tree	Local		Reflora
Miconia theizans (Bonpl.) Cogn.	Tree	Neotrop.		Reflora
Miconia trianae Cogn.	Tree	Regional		Reflora
Miconia tristis Spring	Tree	ESA		Reflora
Miconia urophylla DC.	Tree	Regional		Reflora
Miconia valtheri Naudin	Tree	ESA		Reflora
Miconia willdenowii Klotzsch ex Naudin	Tree	Regional	EN	Reflora
Mouriri chamissoana Cogn.	Tree	ESA		Reflora
Mouriri glazioviana Cogn.	Tree	ESA		Reflora
Mouriri myrtilloides (Sw.) Poir.	Shrub	Neotrop.		Reflora
Ossaea angustifolia (DC.) Triana	Tree	ESA		Reflora
Ossaea marginata (Desr.) Triana	Shrub	ESA		Reflora
Pleroma caissara F. S. Mey.	Tree	Local		Reflora
Tibouchina arborea (Gardner) Cogn.	Tree	Regional		Reflora

Family (Order) and species	Life form	Geog. distribut.	Threat status	Source
Tibouchina boraceiensis Brade	Shrub	Local		Reflora
Tibouchina candolleana (Mart. ex DC.) Cogn.	Tree	Regional	VU	NeoTrop
Tibouchina canescens (D.Don) Cogn.	Tree	Regional		Reflora
Tibouchina clavata (Pers.) Wurdack	Shrub	Regional		Reflora
Tibouchina estrellensis (Raddi) Cogn.	Tree	Regional		Reflora
Tibouchina fothergillae (Schrank & Mart. ex DC.) Cogn.	Tree	Regional		Reflora
Tibouchina granulosa (Desr.) Cogn.	Tree	Local		NeoTrop
Tibouchina kuhlmannii Brade	Shrub	Local		Reflora
Tibouchina mutabilis (Vell.) Cogn.	Tree	Regional		Reflora
Tibouchina pulchra Cogn.	Tree	Regional		Reflora
Tibouchina regnellii Cogn.	Tree	Regional		Reflora
Tibouchina reitzii Brade	Shrub	Regional		Reflora
Tibouchina schenckii Cogn.	Tree	Local	VU	Reflora
Tibouchina sellowiana Cogn.	Tree	ESA		Reflora
Tibouchina stenocarpa (Schrank & Mart. ex DC.) Cogn.	Tree	SA		Reflora
Tibouchina trichopoda (DC.) Baill.	Tree	ESA		Reflora
Tococa guianensis Aubl.	Shrub	Neotrop.		Reflora
Trembleya parviflora (D.Don) Cogn.	Shrub	ESA		Reflora
MELIACEAE (Sapindales)				
Cabralea canjerana (Vell.) Mart.	Tree	Neotrop.		Reflora
Cedrela fissilis Vell.	Tree	SA		Reflora
Cedrela odorata L.	Tree	SA		Reflora
Guarea guidonia (L.) Sleumer	Tree	Neotrop.		Reflora
Guarea kunthiana A.Juss.	Tree	Neotrop.		Reflora
Guarea macrophylla Vahl	Tree	Neotrop.		Reflora
Melia azedarach L. (*)	Tree	exotic		Reflora
Trichilia casaretti C.DC.	Tree	ESA		Reflora
Trichilia catigua A.Juss.	Tree	SA		Reflora
Trichilia clausseni C.DC.	Tree	ESA		Reflora

Family (Order) and species	Life form	Geog. distribut.	Threat status	Source
Trichilia elegans A.Juss.	Tree	SA		Reflora
Trichilia emarginata (Turcz.) C.DC.	Tree	ESA		Reflora
Trichilia hirta L.	Tree	Neotrop.	VU	Reflora
Trichilia lepidota Mart.	Tree	ESA		Reflora
Trichilia martiana C.DC.	Tree	Neotrop.		Reflora
Trichilia pallens C.DC.	Tree	ESA		Reflora
Trichilia pallida Sw.	Tree	Neotrop.		Reflora
Trichilia pseudostipularis (A.Juss.) C.DC.	Tree	ESA		Reflora
Trichilia quadrijuga Kunth	Tree	Neotrop.		Reflora
Trichilia silvatica C.DC.	Tree	ESA		Reflora
MONIMIACEAE (Laurales)				
Hennecartia omphalandra J.Poiss.	Tree	ESA		Reflora
Macropeplus dentatus (Perkins) I.Santos & Peixoto	Tree	Regional		Reflora
Macropeplus ligustrinus (Tul.) Perkins	Tree	ESA		Reflora
Macrotorus utriculatus (Mart.) Perkins	Tree	Regional	CR	Reflora
Mollinedia acutissima Perkins	Tree	Local		NeoTrop
Mollinedia argyrogyna Perkins	Tree	ESA		Reflora
Mollinedia blumenaviana Perkins	Tree	Regional	VU	Reflora
Mollinedia boracensis Peixoto	Tree	Local	VU	Reflora
Mollinedia clavigera Tul.	Tree	Regional		Reflora
Mollinedia elegans Tul.	Tree	Regional		Reflora
Mollinedia engleriana Perkins	Tree	Regional		Reflora
Mollinedia fruticulosa Perkins	Shrub	ESA		Reflora
Mollinedia gilgiana Perkins	Tree	Regional	VU	Reflora
Mollinedia glabra (Spreng.) Perkins	Tree	Regional		Reflora
Mollinedia lamprophylla Perkins	Tree	Regional		NeoTrop
Mollinedia longifolia Perkins	Tree	Local		NeoTrop
Mollinedia luizae Peixoto	Tree	Local	VU	Reflora
Mollinedia micrantha Perkins	Shrub	Local		Reflora

Family (Order) and species	Life form	Geog. distribut.	Threat status	Source
Mollinedia oligantha Perkins	Tree	ESA		Reflora
Mollinedia ovata Ruiz & Pav.	Tree	SA	VU	Reflora
Mollinedia pachysandra Perkins	Tree	Regional	VU	Reflora
Mollinedia salicifolia Perkins	Tree	Regional	EN	Reflora
Mollinedia schottiana (Spreng.) Perkins	Tree	ESA		Reflora
Mollinedia triflora (Spreng.) Tul.	Tree	ESA		Reflora
Mollinedia uleana Perkins	Tree	Regional		Reflora
Mollinedia widgrenii A.DC.	Tree	ESA		Reflora
MORACEAE (Rosales)				
Artocarpus altilis (Parkinson) Fosberg (*)	Tree	Pantrop.		Reflora
Artocarpus heterophyllus Lam. (*)	Tree	Pantrop.		Reflora
Brosimum gaudichaudii Trécul	Tree	SA		Reflora
Brosimum glaziovii Taub.	Tree	ESA	VU	Reflora
Brosimum guianense (Aubl.) Huber	Tree	Neotrop.		Reflora
Brosimum lactescens (S.Moore) C.C.Berg	Tree	Neotrop.		Reflora
Clarisia ilicifolia (Spreng.) Lanj. & Rossberg	Tree	SA		Reflora
Ficus adhatodifolia Schott ex Spreng.	Tree	ESA		Reflora
Ficus arpazusa Casar.	Tree	SA		Reflora
Ficus catappifolia Kunth & C.D.Bouché	Tree	SA		Reflora
Ficus cestrifolia Schott ex Spreng.	Tree	ESA		Reflora
Ficus citrifolia Mill.	Tree (H)	Neotrop.		Reflora
Ficus clusiifolia Schott	Tree	Neotrop.		Reflora
Ficus cyclophylla (Miq.) Miq.	Tree	ESA		Reflora
Ficus enormis Mart. ex Miq.	Tree	ESA		Reflora
Ficus ernanii Carauta , Pederneir. , P.P.Souza , A.F.P.Machado , M.D.M.Vianna & Romaniuc	Tree	ESA		Reflora
Ficus eximia Schott	Tree (H)	Neotrop.		Reflora
Ficus gomelleira Kunth	Tree	SA		Reflora
Ficus guaranitica Chodat	Tree	ESA		Reflora
Ficus hirsuta Schott	Tree	ESA		Reflora

Family (Order) and species	Life form	Geog. distribut.	Threat status	Source
Ficus lagoensis C.C.Berg & Carauta	Tree	Regional		Reflora
Ficus luschnathiana (Miq.) Miq.	Tree	ESA		Reflora
Ficus obtusifolia Kunth	Tree (H)	Neotrop.		Reflora
Ficus obtusiuscula (Miq.) Miq.	Tree	SA		Reflora
Ficus organensis (Miq.) Miq.	Tree	SA		Reflora
Ficus pertusa L.f.	Tree (H)	Neotrop.		Reflora
Ficus pulchella Schott	Tree	SA		Reflora
Ficus tomentella (Miq.) Miq.	Tree	SA		Reflora
Ficus trigona L.f.	Tree (H)	SA		Reflora
Ficus trigonata L.	Tree	SA		Reflora
Maclura tinctoria (L.) D.Don ex Steud.	Tree	Neotrop.		Reflora
Morus alba L. (*)	Tree	exotic		NeoTrop
Morus nigra L. (*)	Tree	exotic		NeoTrop
Pseudolmedia hirtula Kuhlm.	Tree	Regional		Reflora
Pseudolmedia laevigata Trécul	Tree	Neotrop.		Reflora
Sorocea bonplandii (Baill.) W.C.Burger et al.	Tree	ESA		Reflora
Sorocea guilleminiana Gaudich.	Tree	SA		Reflora
Sorocea hilarii Gaudich.	Tree	ESA		Reflora
Sorocea jureiana Romaniuc	Tree	Local		Reflora
Sorocea racemosa Gaudich.	Tree	Regional		Reflora
MYRISTICACEAE (Magnoliales)				
Virola bicuhyba (Schott ex Spreng.) Warb.	Tree	ESA		Reflora
Virola gardneri (A.DC.) Warb.	Tree	ESA		Reflora
Virola sebifera Aubl.	Tree	Neotrop.		Reflora
MYRTACEAE (Myrtales)				
Acca sellowiana (O.Berg) Burret	Tree	Regional		Reflora
Blepharocalyx salicifolius (Kunth) O.Berg	Tree	SA		Reflora
Calyptranthes brasiliensis Spreng.	Tree	ESA		Reflora
Calyptranthes caudata Gardner	Tree	Local		NeoTrop
Calyptranthes clusiifolia O.Berg	Tree	SA		Reflora

Family (Order) and species	Life form	Geog. distribut.	Threat status	Source
Calyptranthes concinna DC.	Tree	ESA		Reflora
Calyptranthes curta Sobral & O.Aguiar	Tree	Local		Reflora
Calyptranthes dichotoma Casar.	Tree	Local		Reflora
Calyptranthes dryadica M.L.Kawas.	Shrub	Local	VU	Reflora
Calyptranthes fusiformis M.L.Kawas.	Shrub	Local	VU	Reflora
Calyptranthes grammica (Spreng.) D.Legrand	Tree	Regional		Reflora
Calyptranthes grandifolia O.Berg	Tree	ESA		Reflora
Calyptranthes lanceolata O.Berg	Tree	ESA		Reflora
Calyptranthes lucida Mart. ex DC.	Tree	Neotrop.		Reflora
Calyptranthes maritima Sobral & Bertoncello	Tree	Local		Reflora
Calyptranthes obovata Kiaersk.	Tree	Regional		Reflora
Calyptranthes obversa O.Berg	Tree	Regional		Reflora
Calyptranthes pileata D.Legrand	Tree	Regional		NeoTrop
Calyptranthes platyphylla O.Berg	Tree	Local		Reflora
Calyptranthes rubella (O.Berg) D.Legrand	Tree	Regional		Reflora
Calyptranthes rufa O.Berg	Shrub	Regional		NeoTrop
Calyptranthes serrana A.R.Lourenço	Tree	Local		Reflora
Calyptranthes strigipes O.Berg	Tree	Neotrop.		Reflora
Calyptranthes ubatubana Sobral & Rochelle	Tree	Local		Reflora
Calyptranthes widgreniana O.Berg	Tree	ESA		Reflora
Campomanesia adamantium (Cambess.) O.Berg	Shrub	ESA		NeoTrop
Campomanesia eugenioides (Cambess.) D.Legrand ex Landrum	Tree	ESA		Reflora
Campomanesia guaviroba (DC.) Kiaersk.	Tree	ESA		Reflora
Campomanesia guazumifolia (Cambess.) O.Berg	Tree	ESA		Reflora
Campomanesia laurifolia Gardner	Tree	Regional		NeoTrop
Campomanesia neriiflora (O.Berg) Nied.	Tree	Local		Reflora
Campomanesia phaea (O.Berg) Landrum	Tree	Regional		Reflora
Campomanesia pubescens (Mart. ex DC.) O.Berg	Tree	ESA		Reflora
Campomanesia reitziana D.Legrand	Tree	Regional		Reflora

Family (Order) and species	Life form	Geog. distribut.	Threat status	Source
Campomanesia schlechtendaliana (O.Berg) Nied.	Tree	ESA	VU	Reflora
Campomanesia sessiliflora (O.Berg) Mattos	Tree	ESA	CR	Reflora
Campomanesia simulans M.L.Kawas.	Tree	Local		Reflora
Campomanesia velutina (Cambess.) O.Berg	Tree	ESA		Reflora
Campomanesia xanthocarpa (Mart.) O.Berg	Tree	ESA		Reflora
Eugenia acutata Miq.	Tree	Regional		Reflora
Eugenia arenaria Cambess.	Shrub	ESA		NeoTrop
Eugenia astringens Cambess.	Tree	ESA		Reflora
Eugenia aurata O.Berg	Tree	ESA		Reflora
Eugenia bacopari D.Legrand	Shrub	Regional		NeoTrop
Eugenia bahiensis DC.	Tree	Regional	VU	NeoTrop
Eugenia batingabranca Sobral	Tree	Regional		Reflora
Eugenia beaurepairiana (Kiaersk.) D.Legrand	Tree	ESA		Reflora
Eugenia bocainensis Mattos	Tree	Regional	VU	Reflora
Eugenia brasiliensis Lam.	Tree	ESA	VU	Reflora
Eugenia brevistyla D.Legrand	Tree	Regional		Reflora
Eugenia brunneopubescens Mazine	Tree	Local		NeoTrop
Eugenia brunoi Mattos	Tree	Local	EN	Reflora
Eugenia bunchosiifolia Nied.	Tree	Regional	VU	Reflora
Eugenia burkartiana (D.Legrand) D.Legrand	Tree	Regional	VU	Reflora
Eugenia candolleana DC.	Tree	ESA		Reflora
Eugenia capitulifera O.Berg	Tree	Regional		NeoTrop
Eugenia catharinae O.Berg	Tree	Regional		NeoTrop
Eugenia catharinensis D.Legrand	Tree	Regional		Reflora
Eugenia cerasiflora Miq.	Tree	ESA		Reflora
Eugenia cereja D.Legrand	Tree	Regional		Reflora
Eugenia chlorophylla O.Berg	Tree	Regional		Reflora
Eugenia conchalensis D.Legrand & Mattos	Tree	Local		Reflora
Eugenia concolor Mattos	Tree	Local		Reflora
Eugenia copacabanensis Kiaersk.	Tree	Regional	VU	Reflora

Family (Order) and species	Life form	Geog. distribut.	Threat status	Source
Eugenia cuprea (O.Berg) Nied.	Tree	Regional		Reflora
Eugenia disperma Vell.	Tree	Local	VU	Reflora
Eugenia dodonaeifolia Cambess.	Tree	Regional		Reflora
Eugenia dulcis O.Berg	Tree	Local		Reflora
Eugenia dysenterica (Mart.) DC.	Tree	ESA		Reflora
Eugenia egensis DC.	Tree	Neotrop.		Reflora
Eugenia elongata Nied.	Tree	Local		Reflora
Eugenia excelsa O.Berg	Tree	SA		Reflora
Eugenia flamingensis O.Berg	Tree	Neotrop.		NeoTrop
Eugenia flavescens DC.	Shrub	SA		Reflora
Eugenia florida DC.	Tree	Neotrop.		Reflora
Eugenia francavilleana O.Berg	Tree	ESA		Reflora
Eugenia fusca O.Berg	Tree	ESA		Reflora
Eugenia gracillima Kiaersk.	Shrub	ESA		Reflora
Eugenia handroana D.Legrand	Tree	Regional		Reflora
Eugenia handroi (Mattos) Mattos	Tree	ESA		Reflora
Eugenia hermesiana Mattos	Tree	Local	VU	Reflora
Eugenia hiemalis Cambess.	Tree	ESA		Reflora
Eugenia impunctata O.Berg	Tree	Local		Reflora
Eugenia involucrata DC.	Tree	ESA		Reflora
Eugenia ischnosceles O.Berg	Tree	Local		Reflora
Eugenia kleinii D.Legrand	Tree	Regional		Reflora
Eugenia klotzschiana O.Berg	Shrub	ESA	VU	Reflora
Eugenia leptoclada O.Berg	Tree	Regional		Reflora
Eugenia ligustrina (Sw.) Willd.	Tree	Neotrop.		Reflora
Eugenia livida O.Berg	Shrub	ESA		Reflora
Eugenia longibracteata Mazine	Shrub	Local		Reflora
Eugenia longipedunculata Nied.	Tree	Regional		Reflora
Eugenia longipetiolata Mattos	Tree	Local		Reflora

Family (Order) and species	Life form	Geog. distribut.	Threat status	Source
Eugenia luschnathiana (O.Berg) Klotzsch ex B.D.Jacks.	Tree	ESA		Reflora
Eugenia macahensis O.Berg	Tree	Local		Reflora
Eugenia macedoi Mattos & D.Legrand	Shrub	Local		NeoTrop
Eugenia macrobracteolata Mattos	Tree	Regional		Reflora
Eugenia magnibracteolata Mattos & D.Legrand	Tree	Regional		Reflora
Eugenia malacantha D.Legrand	Tree	Regional		Reflora
Eugenia mansoi O.Berg	Tree	Regional		Reflora
Eugenia melanogyna (D.Legrand) Sobral	Tree	Regional		Reflora
Eugenia membranifolia Nied.	Tree	Local		Reflora
Eugenia modesta DC.	Tree	ESA		Reflora
Eugenia monosperma Vell.	Tree	Regional		Reflora
Eugenia mosenii (Kausel) Sobral	Tree	Regional		Reflora
Eugenia multicostata D.Legrand	Tree	Regional		Reflora
Eugenia myrcianthes Nied.	Tree	ESA		Reflora
Eugenia neoglomerata Sobral	Tree	ESA		Reflora
Eugenia neomyrtifolia Sobral	Tree	ESA		Reflora
Eugenia neosilvestris Sobral	Tree	Regional		Reflora
Eugenia neoverrucosa Sobral	Tree	ESA		Reflora
Eugenia nutans O.Berg	Tree	ESA		Reflora
Eugenia oblongata O.Berg	Tree	ESA		Reflora
Eugenia oeidocarpa O.Berg	Shrub	Regional		NeoTrop
Eugenia ophthalmantha Kiaersk.	Shrub	Regional		Reflora
Eugenia pantagensis O.Berg	Tree	Regional		Reflora
Eugenia paracatuana O.Berg	Tree	ESA		Reflora
Eugenia paranapiacabensis Mattos	Tree	Local		Reflora
Eugenia peruibensis Mattos	Tree	Local	VU	Reflora
Eugenia piloensis Cambess.	Tree	Regional		Reflora
Eugenia pisiformis Cambess.	Tree	ESA		Reflora
Eugenia platysema O.Berg	Tree	Regional		NeoTrop

Family (Order) and species	Life form	Geog. distribut.	Threat status	Source
Eugenia plicata Nied.	Tree	Regional		Reflora
Eugenia pluriflora DC.	Tree	ESA		Reflora
Eugenia prasina O.Berg	Tree	ESA		Reflora
Eugenia pruinosa D.Legrand	Tree	Regional		Reflora
Eugenia pruniformis Cambess.	Tree	Regional		NeoTrop
Eugenia pseudomalacantha D.Legrand	Tree	Regional		NeoTrop
Eugenia punicifolia (Kunth) DC.	Tree	Neotrop.		Reflora
Eugenia pyriformis Cambess.	Tree	ESA		Reflora
Eugenia ramboi D.Legrand	Tree	Regional		Reflora
Eugenia regia Bünger & Sobral	Tree	Local		Reflora
Eugenia repanda O.Berg	Tree	ESA		Reflora
Eugenia sonderiana O.Berg	Tree	ESA		Reflora
Eugenia speciosa Cambess.	Tree	ESA		Reflora
Eugenia sphenoides O.Berg	Tree	Local		Reflora
Eugenia sphenophylla O.Berg	Tree	Local		Reflora
Eugenia sprengelii DC.	Tree	Local		NeoTrop
Eugenia squamiflora Mattos	Tree	Local		Reflora
Eugenia stigmatosa DC.	Tree	ESA		Reflora
Eugenia subamplexicaulis DC.	Tree	Local		Reflora
Eugenia subavenia O.Berg	Tree	Regional		Reflora
Eugenia suberosa Cambess.	Shrub	ESA		Reflora
Eugenia subterminalis DC.	Tree	SA		Reflora
Eugenia subundulata Kiaersk.	Shrub	Regional		Reflora
Eugenia sulcata Spring ex Mart.	Tree	Regional		Reflora
Eugenia supraaxillaris Spring	Tree	Regional		Reflora
Eugenia tenuipedunculata Kiaersk.	Tree	Local		NeoTrop
Eugenia uniflora L.	Tree	ESA		Reflora
Eugenia uruguayensis Cambess.	Tree	Regional		NeoTrop
Eugenia vattimoana Mattos	Tree	Regional		NeoTrop
Eugenia verticillata (Vell.) Angely	Tree	ESA		Reflora

Family (Order) and species	Life form	Geog. distribut.	Threat status	Source
Eugenia viridiflora Cambess.	Shrub	Regional		Reflora
Eugenia xiriricana Mattos	Tree	Local		Reflora
Marlierea angustifolia (O.Berg) Mattos	Tree	Regional		Reflora
Marlierea antonia (O.Berg) D.Legrand	Tree	Local		Reflora
Marlierea clausseniana (O.Berg) Kiaersk.	Tree	ESA		Reflora
Marlierea eugeniopsoides (D.Legrand & Kausel) D.Legrand	Tree	Regional		Reflora
Marlierea excoriata Mart.	Tree	ESA		Reflora
Marlierea gaudichaudiana (O.Berg) Loefgr. & Everett	Tree	Regional		NeoTrop
Marlierea glazioviana Kiaersk.	Tree	Local		Reflora
Marlierea involucrata (O.Berg) Nied.	Tree	Local		NeoTrop
Marlierea laevigata (DC.) Kiaersk.	Tree	ESA		Reflora
Marlierea obscura O.Berg	Tree	Regional		NeoTrop
Marlierea polygama (O.Berg) D.Legrand	Tree	Regional		NeoTrop
Marlierea racemosa (Vell.) Kiaersk.	Tree	ESA		Reflora
Marlierea regeliana O.Berg	Tree	ESA	VU	NeoTrop
Marlierea reitzii D.Legrand	Tree	Local		Reflora
Marlierea riedeliana (O.Berg) D.Legrand	Shrub	ESA		Reflora
Marlierea silvatica (O.Berg) Kiaersk.	Tree	ESA		Reflora
Marlierea skortzoviana Mattos	Tree	Local	VU	Reflora
Marlierea suaveolens Cambess.	Tree	Regional	VU	Reflora
Marlierea tomentosa Cambess.	Tree	ESA		Reflora
Marlierea villas-boasii Mattos	Tree	Local		Reflora
Myrceugenia acutata D.Legrand	Shrub	ESA		Reflora
Myrceugenia acutiflora (Kiaersk.) D.Legrand & Kausel	Tree	ESA		NeoTrop
Myrceugenia alpigena (DC.) Landrum	Tree	ESA		Reflora
Myrceugenia bracteosa (DC.) D.Legrand & Kausel	Tree	Regional	VU	Reflora
Myrceugenia brevipedicellata (Burret) D.Legrand & Kausel	Shrub	Local	EN	Reflora

Family (Order) and species	Life form	Geog. distribut.	Threat status	Source
Myrceugenia campestris (DC.) D.Legrand & Kausel	Tree	ESA		Reflora
Myrceugenia cucullata D.Legrand	Tree	Regional		Reflora
Myrceugenia decussata Mattos	Shrub	Local		Reflora
Myrceugenia euosma (O.Berg) D.Legrand	Tree	Regional		Reflora
Myrceugenia franciscensis (O.Berg) Landrum	Tree	Regional		Reflora
Myrceugenia gertii Landrum	Tree	Local	VU	Reflora
Myrceugenia glaucescens (Cambess.) D.Legrand & Kausel	Tree	ESA		Reflora
Myrceugenia hoehnei (Burret) D.Legrand & Kausel	Shrub	Regional	VU	Reflora
Myrceugenia kleinii D.Legrand & Kausel	Tree	Regional	VU	Reflora
Myrceugenia miersiana (Gardner) D.Legrand & Kausel	Tree	ESA		Reflora
Myrceugenia myrcioides (Cambess.) O.Berg	Tree	ESA		Reflora
Myrceugenia ovalifolia (O.Berg) Landrum	Tree	ESA		Reflora
Myrceugenia ovata (Hook. & Arn.) O.Berg	Shrub	SA		NeoTrop
Myrceugenia oxysepala (Burret) D.Legrand & Kausel	Tree	Regional	EN	Reflora
Myrceugenia pilotantha (Kiaersk.) Landrum	Tree	ESA		Reflora
Myrceugenia reitzii D.Legrand	Tree	Regional		Reflora
Myrceugenia rufescens (DC.) D.Legrand & Kausel	Tree	Regional		Reflora
Myrceugenia scutellata D.Legrand	Tree	ESA		Reflora
Myrceugenia seriatoramosa (Kiaersk.) D.Legrand & Kausel	Tree	Regional		Reflora
Myrceugenia venosa D.Legrand	Tree	Local	VU	Reflora
Myrcia aethusa (O.Berg) N.Silveira	Tree	ESA		Reflora
Myrcia albotomentosa DC.	Shrub	ESA		NeoTrop
Myrcia amazonica DC.	Tree	Neotrop.		Reflora
Myrcia anacardiifolia Gardner	Tree	ESA		Reflora
Myrcia anceps (Spreng.) O.Berg	Tree	Regional		Reflora

Family (Order) and species	Life form	Geog. distribut.	Threat status	Source
Myrcia apiocarpa (O.Berg) N.Silveira	Tree	Local		Reflora
Myrcia bella Cambess.	Tree	ESA		Reflora
Myrcia bicolor Kiaersk.	Tree	Regional		Reflora
Myrcia brasiliensis Kiaersk.	Tree	ESA		Reflora
Myrcia calyptranthoides (O.Berg) Mattos	Tree	Regional		NeoTrop
Myrcia coelosepala Kiaersk.	Tree	Regional		Reflora
Myrcia colpodes Kiaersk.	Tree	Local		Reflora
Myrcia cordiifolia DC.	Tree	ESA	EN	NeoTrop
Myrcia crocea Kiaersk.	Tree	ESA	CR	NeoTrop
Myrcia diaphana (O.Berg) N.Silveira	Tree	ESA	VU	Reflora
Myrcia dichrophylla D.Legrand	Tree	Regional	VU	Reflora
Myrcia eriocalyx DC.	Tree	Regional		Reflora
Myrcia eriopus DC.	Tree	ESA	VU	Reflora
Myrcia eumecephylla (O.Berg) Nied.	Tree	Regional		Reflora
Myrcia fenzliana O.Berg	Tree	Neotrop.		Reflora
Myrcia flagellaris (D.Legrand) Sobral	Tree	Regional	VU	Reflora
Myrcia freyreissiana (O.Berg) Kiaersk.	Tree	Regional		Reflora
Myrcia glabra (O.Berg) D.Legrand	Tree	Regional		Reflora
Myrcia grandifolia Cambess.	Tree	ESA		Reflora
Myrcia guianensis (Aubl.) DC.	Tree	Neotrop.		Reflora
Myrcia hartwegiana (O.Berg) Kiaersk.	Tree	ESA		Reflora
Myrcia hatschbachii D.Legrand	Tree	Regional		NeoTrop
Myrcia hebepetala DC.	Tree	ESA		Reflora
Myrcia heringii D.Legrand	Shrub	Regional		Reflora
Myrcia hexasticha Kiaersk.	Tree	Regional	VU	Reflora
Myrcia ilheosensis Kiaersk.	Tree	SA		Reflora
Myrcia insularis Gardner	Tree	ESA	VU	Reflora
Myrcia isaiana G.M.Barroso & Peixoto	Tree	ESA		Reflora
Myrcia laruotteana Cambess.	Tree	ESA		Reflora
Myrcia laxiflora Cambess.	Tree	ESA		Reflora

Family (Order) and species	Life form	Geog. distribut.	Threat status	Source
Myrcia macrocarpa DC.	Tree	Regional		Reflora
Myrcia montana Cambess.	Tree	Regional		NeoTrop
Myrcia multiflora (Lam.) DC.	Tree	SA		Reflora
Myrcia neoblanchetiana E.Lucas & Sobral	Tree	Regional		NeoTrop
Myrcia oblongata DC.	Tree	ESA	VU	Reflora
Myrcia obovata (O.Berg) Nied.	Tree	Regional	EN	Reflora
Myrcia oligantha O.Berg	Tree	ESA		Reflora
Myrcia ovata Cambess.	Tree	Regional	VU	Reflora
Myrcia palustris DC.	Tree	ESA		Reflora
Myrcia plusiantha Kiaersk.	Tree	ESA		Reflora
Myrcia pubescens DC.	Tree	ESA		NeoTrop
Myrcia pubiflora DC.	Tree	ESA		Reflora
Myrcia pubipetala Miq.	Tree	ESA		Reflora
Myrcia pulchra (O.Berg) Kiaersk.	Tree	ESA	VU	Reflora
Myrcia racemosa (O.Berg) Kiaersk.	Tree	ESA		Reflora
Myrcia retorta Cambess.	Tree	ESA		Reflora
Myrcia rufipes DC.	Tree	ESA		Reflora
Myrcia rupicola D.Legrand	Tree	Regional		Reflora
Myrcia selloi (Spreng.) N.Silveira	Shrub	ESA		Reflora
Myrcia spectabilis DC.	Tree	ESA		Reflora
Myrcia splendens (Sw.) DC.	Tree	Neotrop.		Reflora
Myrcia springiana (O.Berg) Kiaersk.	Tree	Regional		Reflora
Myrcia squamata (Mattos & D.Legrand) Mattos	Tree	Regional		Reflora
Myrcia stictophylla (O.Berg) N.Silveira	Tree	Local	VU	Reflora
Myrcia tenuivenosa Kiaersk.	Tree	ESA		Reflora
Myrcia tijucensis Kiaersk.	Tree	ESA	VU	Reflora
Myrcia tomentosa (Aubl.) DC.	Tree	Neotrop.		Reflora
Myrcia uberavensis O.Berg	Shrub	ESA		Reflora
Myrcia undulata O.Berg	Tree	Regional		NeoTrop
Myrcia variabilis DC.	Tree	ESA	CR	Reflora

Family (Order) and species	Life form	Geog. distribut.	Threat status	Source
Myrcia venulosa DC.	Tree	ESA		Reflora
Myrcia vestita DC.	Tree	ESA		Reflora
Myrcia vittoriana Kiaersk.	Tree	ESA		NeoTrop
Myrcianthes cionei Mattos	Tree	Local		Reflora
Myrcianthes gigantea (D.Legrand) D.Legrand	Tree	ESA		NeoTrop
Myrcianthes pungens (O.Berg) D.Legrand	Tree	ESA		Reflora
Myrciaria cuspidata O.Berg	Tree	ESA		Reflora
Myrciaria delicatula (DC.) O.Berg	Tree	ESA		Reflora
Myrciaria floribunda (H.West ex Willd.) O.Berg	Tree	Neotrop.		Reflora
Myrciaria pallida O.Berg	Tree	Local		NeoTrop
Myrciaria tenella (DC.) O.Berg	Tree	Neotrop.		Reflora
Myrrhinium atropurpureum Schott	Tree	SA		Reflora
Neomitranthes amblymitra (Burret) Mattos	Tree	Local	CR	Reflora
Neomitranthes capivariensis (Mattos) Mattos	Tree	Local	EN	Reflora
Neomitranthes glomerata (D.Legrand) D.Legrand	Tree	ESA		Reflora
Neomitranthes gracilis (Burret) N.Silveira	Tree	Local	EX	Reflora
Neomitranthes obscura (DC.) N.Silveira	Tree	ESA		Reflora
Neomitranthes pedicellata (Burret) Mattos	Tree	Local	EN	Reflora
Neomitranthes regeliana (O.Berg) M.Souza	Tree	Regional		Reflora
Neomitranthes warmingiana (Kiaersk.) Mattos	Tree	Regional		Reflora
Pimenta pseudocaryophyllus (Gomes) Landrum	Tree	ESA		Reflora
Plinia cauliflora (Mart.) Kausel	Tree	ESA		Reflora
Plinia complanata M.L.Kawas. & B.Holst	Tree	Local	VU	Reflora
Plinia coronata (Mattos) Mattos	Tree	Local		Reflora
Plinia edulis (Vell.) Sobral	Tree	ESA	VU	Reflora
Plinia oblongata (Mattos) Mattos	Tree	Local		Reflora
Plinia peruviana (Poir.) Govaerts	Tree	SA		Reflora
Plinia phitrantha (Kiaersk.) Sobral	Tree	Regional		Reflora
Plinia pseudodichasiantha (Kiaersk.) G.M.Barroso ex Sobral	Tree	ESA		Reflora

Family (Order) and species	Life form	Geog. distribut.	Threat status	Source
Plinia rivularis (Cambess.) Rotman	Tree	SA		Reflora
Plinia rogersiana Mattos	Tree	Local		Reflora
Psidium araucanum Soares-Silva & Proença	Tree	Local		Reflora
Psidium cattleianum Sabine	Tree	ESA		Reflora
Psidium giganteum Mattos	Tree	Regional	VU	Reflora
Psidium grandifolium Mart. ex DC.	Shrub	ESA		NeoTrop
Psidium guajava L. (*)	Tree	Neotrop.		Reflora
Psidium guineense Sw.	Shrub	Neotrop.		Reflora
Psidium inaequilaterum O.Berg	Tree	Local		Reflora
Psidium itanareense O.Berg	Tree	Local		Reflora
Psidium laruotteanum Cambess.	Shrub	ESA		Reflora
Psidium longipetiolatum D.Legrand	Tree	Regional		Reflora
Psidium myrtoides O.Berg	Tree	ESA		Reflora
Psidium ovale (Spreng.) Burret	Tree	ESA		Reflora
Psidium robustum O.Berg	Shrub	Local		NeoTrop
Psidium rufum Mart. ex DC.	Tree	ESA		Reflora
Psidium salutare (Kunth) O.Berg	Shrub	Neotrop.		Reflora
Psidium sartorianum (O.Berg) Nied.	Tree	Neotrop.	VU	Reflora
Psidium subrostrifolium Mattos	Tree	Local		Reflora
Psidium ubatubense Mattos	Tree	Local		Reflora
Siphoneugena crassifolia (DC.) Proença & Sobral	Tree	Regional		Reflora
Siphoneugena densiflora O.Berg	Tree	ESA		Reflora
Siphoneugena guilfoyleiana Proença	Tree	Local		Reflora
Siphoneugena kiaerskoviana (Burret) Kausel	Tree	Regional		NeoTrop
Siphoneugena kuhlmannii Mattos	Tree	Local	VU	Reflora
Siphoneugena reitzii D.Legrand	Tree	ESA	VU	Reflora
Syzygium cumini (L.) Skeels (*)	Tree	exotic		Reflora
Syzygium jambos (L.) Alston (*)	Tree	Pantrop.		Reflora
NYCTAGINACEAE (Caryophyllales)				
Bougainvillea glabra Choisy	Tree (L)	ESA		Reflora

Family (Order) and species	Life form	Geog. distribut.	Threat status	Source
Bougainvillea spectabilis Willd.	Tree (L)	ESA		NeoTrop
Guapira areolata (Heimerl) Lundell	Tree	ESA		Reflora
Guapira graciliflora (Mart. ex Schmidt) Lundell	Tree	SA		Reflora
Guapira hirsuta (Choisy) Lundell	Tree	SA	EN	Reflora
Guapira nitida (Mart. ex J.A.Schmidt) Lundell	Tree	SA		Reflora
Guapira noxia (Netto) Lundell	Tree	ESA		Reflora
Guapira obtusata (Jacq.) Little	Tree	SA	VU	Reflora
Guapira opposita (Vell.) Reitz	Tree	SA		Reflora
Guapira pernambucensis (Casar.) Lundell	Tree	ESA		Reflora
Neea hermaphrodita S.Moore	Tree	SA		Reflora
Neea pendulina Heimerl	Tree	Regional		Reflora
Neea theifera Oerst.	Shrub	SA		Reflora
Neea verticillata Ruiz & Pav.	Tree	SA	EN	Reflora
Pisonia aculeata L.	Shrub	ESA		Reflora
Pisonia ambigua Heimerl	Tree	ESA		Reflora
OCHNACEAE (Malpighiales)				
Ouratea castaneifolia (DC.) Engl.	Tree	SA		NeoTrop
Ouratea cuspidata (A.St.-Hil.) Engl.	Shrub	ESA		Reflora
Ouratea floribunda (A.St.-Hil.) Engl.	Shrub	ESA		Reflora
Ouratea hexasperma (A.St.-Hil.) Baill.	Tree	ESA		Reflora
Ouratea multiflora (Pohl) Engl.	Tree	Regional		Reflora
Ouratea ovalis (Pohl) Engl.	Tree	Regional		Reflora
Ouratea parviflora (A.DC.) Baill.	Tree	ESA		Reflora
Ouratea salicifolia (A.St.-Hil. & Tul.) Engl.	Tree	ESA		Reflora
Ouratea sellowii (Planch.) Engl.	Shrub	Regional		Reflora
Ouratea semiserrata (Mart. & Nees) Engl.	Tree	Regional		Reflora
Ouratea spectabilis (Mart.) Engl.	Tree	ESA		Reflora
Ouratea vaccinioides (A.St.-Hil. & Tul.) Engl.	Tree	Regional		Reflora
OLACACEAE (Santalales)				
Heisteria perianthomega (Vell.) Sleumer	Tree	ESA	CR	Reflora

Family (Order) and species	Life form	Geog. distribut.	Threat status	Source
Heisteria silvianii Schwacke	Tree	ESA		Reflora
Tetrastylidium grandifolium (Baill.) Sleumer	Tree	ESA		Reflora
Ximenia americana L.	Tree	Pantrop.		Reflora
OLEACEAE (Lamiales)				
Chionanthus crassifolius (Mart.) P.S.Green	Tree	ESA		Reflora
Chionanthus filiformis (Vell.) P.S.Green	Tree	Regional		Reflora
Chionanthus fluminensis (Miers) P.S.Green	Tree	Local	EN	NeoTrop
Chionanthus trichotomus (Vell.) P.S.Green	Tree	ESA		Reflora
Ligustrum vulgare L. (*)	Tree	exotic		Reflora
ONAGRACEAE (Myrtales)				
Fuchsia regia (Vell.) Munz	Tree	Regional		Reflora
Ludwigia nervosa (Poir.) H.Hara	Shrub	Neotrop.		Reflora
OPILIACEAE (Santalales)				
Agonandra brasiliensis Miers ex Benth. & Hook.f.	Tree	Neotrop.		Reflora
Agonandra excelsa Griseb.	Tree	SA		Reflora
PENTAPHYLACACEAE (Ericales)				
Ternstroemia brasiliensis Cambess.	Tree	ESA		Reflora
Ternstroemia cuneifolia Gardner	Shrub	Regional	CR	Reflora
PERACEAE (Malpighiales)				
Chaetocarpus echinocarpus (Baill.) Ducke	Tree	SA		Reflora
Pera glabrata (Schott) Poepp. ex Baill.	Tree	SA		Reflora
Pera heteranthera (Schrank) I.M.Johnst.	Tree	ESA		Reflora
PHYLLANTHACEAE (Malpighiales)				
Gonatogyne brasiliensis (Baill.) Müll.Arg.	Tree	Regional		Reflora
Hyeronima alchorneoides Allemão	Tree	Neotrop.		Reflora
Hyeronima oblonga (Tul.) Müll.Arg.	Tree	SA		NeoTrop
Margaritaria nobilis L.f.	Tree	Neotrop.		Reflora
Phyllanthus acuminatus Vahl	Tree	Neotrop.		Reflora
Phyllanthus cladotrichus Müll.Arg.	Tree	Regional		Reflora

Family (Order) and species	Life form	Geog. distribut.	Threat status	Source
Phyllanthus juglandifolius Willd.	Tree	Neotrop.		Reflora
Phyllanthus riedelianus Müll.Arg.	Tree	Regional		Reflora
Phyllanthus umbratus Müll.Arg.	Tree	Local		Reflora
Richeria grandis Vahl	Tree	Neotrop.		Reflora
Savia dictyocarpa Müll.Arg.	Tree	ESA		Reflora
PHYTOLACCACEAE (Caryophyllales)				
Gallesia integrifolia (Spreng.) Harms	Tree	SA		Reflora
Phytolacca dioica L.	Tree	Neotrop.		Reflora
Seguieria americana L.	Tree (L)	Neotrop.		Reflora
Seguieria langsdorffii Moq.	Tree	ESA		Reflora
PICRAMNIACEAE (Picramniales)				
Picramnia ciliata Mart.	Tree	ESA		Reflora
Picramnia gardneri Planch.	Tree	ESA		Reflora
Picramnia glazioviana Engl.	Tree	ESA		Reflora
Picramnia parvifolia Engl.	Tree	Regional		Reflora
Picramnia ramiflora Planch.	Tree	ESA		Reflora
Picramnia sellowii Planch.	Tree	SA		Reflora
PINACEAE (Pinales)				
Pinus caribaea Morelet (*)	Tree	exotic		Reflora
Pinus elliottii L. (*)	Tree	exotic		Reflora
Pinus taeda L. (*)	Tree	exotic		Reflora
PIPERACEAE (Piperales)				
Piper aduncum L.	Tree	Neotrop.		Reflora
Piper amalago L.	Tree	Neotrop.		Reflora
Piper arboreum Aubl.	Tree	Neotrop.		Reflora
Piper caldense C.DC.	Shrub	ESA		Reflora
Piper cernuum Vell.	Tree	SA		Reflora
Piper corcovadensis (Miq.) C.DC.	Shrub	ESA		NeoTrop
Piper crassinervium Kunth	Shrub	Neotrop.		Reflora
Piper gaudichaudianum Kunth	Shrub	ESA		Reflora

Family (Order) and species	Life form	Geog. distribut.	Threat status	Source
Piper hispidum Sw.	Tree	Neotrop.		Reflora
Piper macedoi Yunck.	Shrub	ESA		Reflora
Piper mollicomum Kunth	Shrub	Neotrop.		Reflora
Piper obliquum Ruiz & Pav.	Tree	Neotrop.	EN	Reflora
Piper richardiifolium Kunth	Shrub	ESA		Reflora
Piper rivinoides Kunth	Shrub	SA		Reflora
Piper solmsianum C.DC.	Shrub	ESA		Reflora
Piper tectoniifolium Kunth	Tree	ESA	EN	Reflora
Piper tuberculatum Jacq.	Shrub	Neotrop.		NeoTrop
Piper xylosteoides (Kunth) Steud.	Shrub	ESA	VU	NeoTrop
PITTOSPORACEAE (Apiales)				
Pittosporum undulatum Vent. (*)	Tree	exotic		Reflora
POACEAE (Poales)				
Actinocladum verticillatum (Nees) McClure ex Soderstr.	Bamboo	SA		Reflora
Apoclada simplex McClure & L.B.Sm.	Bamboo	Regional	VU	Reflora
Aulonemia amplissima (Nees) McClure	Bamboo	Regional		Reflora
Aulonemia aristulata (Döll) McClure	Bamboo	ESA	VU	Reflora
Aulonemia radiata (Rupr.) McClure & L.B.Sm.	Bamboo	Regional		Reflora
Bambusa dissemulator McClure (*)	Bamboo	exotic		Reflora
Bambusa multiplex (Lour.) Raeusch. (*)	Bamboo	exotic		Reflora
Bambusa tulda Roxb. (*)	Bamboo	exotic		Reflora
Bambusa tuldoides Munro (*)	Bamboo	exotic		Reflora
Bambusa ventricosa McClure (*)	Bamboo	exotic		Reflora
Bambusa vulgaris Schrad. ex J.C.Wendl. (*)	Bamboo	exotic		Reflora
Chusquea anelythra Nees	Bamboo	Regional		Reflora
Chusquea anelytroides Rupr. ex Döll	Bamboo	Regional		Reflora
Chusquea capitata Nees	Bamboo	ESA		Reflora
Chusquea capituliflora Trin.	Bamboo	ESA		Reflora
Chusquea juergensii Hack.	Bamboo	Regional		Reflora

Family (Order) and species	Life form	Geog. distribut.	Threat status	Source
Chusquea meyeriana Rupr. ex Döll	Bamboo	ESA		Reflora
Chusquea urelytra Hack.	Bamboo	Regional		Reflora
Eremocaulon setosum Londono & L.G.Clark	Bamboo	Local	VU	Reflora
Guadua chacoensis (Rojas) Londoño & P.M.Peterson	Bamboo	ESA		Reflora
Guadua paniculata Munro	Bamboo	Neotrop.		Reflora
Guadua paraguayana Döll	Bamboo	ESA		Reflora
Guadua tagoara (Nees) Kunth	Bamboo	ESA		Reflora
Guadua trinii (Nees) Nees ex Rupr.	Bamboo	Regional		Reflora
Guadua velutina Londoño & L.G.Clark (*)	Bamboo	exotic		Reflora
Merostachys argyronema Lindm.	Bamboo	Local		Reflora
Merostachys brevigluma Send.	Bamboo	Local		Reflora
Merostachys burmanii Send.	Bamboo	Regional	EX	Reflora
Merostachys caucaiana Send.	Bamboo	Regional	EN	Reflora
Merostachys fistulosa Döll	Bamboo	Regional		Reflora
Merostachys magellanica Send.	Bamboo	Local		Reflora
Merostachys multiramea Hack.	Bamboo	Regional		Reflora
Merostachys neesii Rupr.	Bamboo	Regional	EN	Reflora
Merostachys petiolata Döll	Bamboo	Regional		Reflora
Merostachys pluriflora Munro ex E.G.Camus	Bamboo	Regional		Reflora
Merostachys polyantha McClure	Bamboo	Local		Reflora
Merostachys riedeliana Rupr. ex Döll	Bamboo	Local		Reflora
Merostachys scandens Send.	Bamboo	Local	VU	Reflora
Merostachys skvortzovii Send.	Bamboo	Regional	VU	Reflora
Merostachys speciosa Spreng.	Bamboo	Regional		Reflora
Merostachys ternata Nees	Bamboo	ESA		Reflora
Phyllostachys edulis (Carriére) J. Houz. (*)	Bamboo	exotic		Reflora
Phyllostachys heterocycla (Carriére) S.Matsum. (*)	Bamboo	exotic		Reflora
Phyllostachys nigra (Lodd. ex Lindl.) Munro (*)	Bamboo	exotic		Reflora
PODOCARPACEAE (Pinales)				

Family (Order) and species	Life form	Geog. distribut.	Threat status	Source
Podocarpus lambertii Klotzsch ex Endl.	Tree	ESA		Reflora
Podocarpus sellowii Klotzsch ex Endl.	Tree	SA		Reflora
POLYGALACEAE (Fabales)				
Acanthocladus brasiliensis (Klotzsch ex A.St.-Hil. & Moq.) Hassk.	Shrub	Regional		Reflora
Asemeia acuminata (Willd.) J.F.B.Pastore & J.R.Abbott	Shrub	SA		Reflora
Bredemeyera floribunda Willd.	Tree (L)	SA		Reflora
Bredemeyera hebeclada (DC.) J.F.B.Pastore	Tree (L)	ESA		Reflora
Bredemeyera laurifolia (A.St.-Hil. & Moq.) Klotzsch ex A.W.Benn.	Tree (L)	ESA		Reflora
POLYGONACEAE (Caryophyllales)				
Coccoloba arborescens (Vell.) R.A.Howard	Tree (L)	SA		NeoTrop
Coccoloba cordata Cham.	Tree	Regional		Reflora
Coccoloba cujabensis Wedd.	Tree	ESA		Reflora
Coccoloba declinata (Vell.) Mart.	Tree	SA		Reflora
Coccoloba fastigiata Meisn.	Tree	Local		Reflora
Coccoloba glaziovii Lindau	Tree	ESA		Reflora
Coccoloba mollis Casar.	Tree	Neotrop.		Reflora
Coccoloba persicaria Wedd.	Tree	ESA		Reflora
Coccoloba striata Benth.	Tree	SA		Reflora
Coccoloba warmingii Meisn.	Tree	ESA		Reflora
Ruprechtia laurifolia (Cham. & Schltdl.) A.C.Meyer	Tree	Regional		Reflora
Ruprechtia laxiflora Meisn.	Tree	SA		Reflora
Triplaris americana L.	Tree	Neotrop.		Reflora
Triplaris gardneriana Wedd.	Tree	ESA		NeoTrop
PRIMULACEAE (Ericales)				
Ardisia guianensis (Aubl.) Mez	Tree	Neotrop.		NeoTrop
Clavija nutans (Vell.) B.Ståhl	Shrub	ESA		Reflora
Clavija spinosa (Vell.) Mez	Shrub	Regional	EN	NeoTrop

Family (Order) and species	Life form	Geog. distribut.	Threat status	Source
Cybianthus alpestris (Warm.) Mez	Shrub	Local		NeoTrop
Cybianthus brasiliensis (Mez) G.Agostini	Shrub	ESA		NeoTrop
Cybianthus cuneifolius Mart.	Shrub	Regional		NeoTrop
Cybianthus fuscus Mart.	Tree	SA		NeoTrop
Cybianthus goyazensis Mez	Shrub	Regional		Reflora
Cybianthus membranaceus Jung-Mend., Bernacci & M.F.Freitas	Tree	Local		Reflora
Geissanthus ambiguus (Mart.) G.Agostini	Tree	SA		Reflora
Myrsine altomontana M.F.Freitas & Kin.-Gouv.	Tree	Regional		NeoTrop
Myrsine balansae (Mez) Otegui	Tree	ESA		Reflora
Myrsine coriacea (Sw.) R.Br. ex Roem. & Schult.	Tree	Neotrop.		Reflora
Myrsine emarginella Miq.	Shrub	Regional		NeoTrop
Myrsine gardneriana A.DC.	Tree	ESA		Reflora
Myrsine guianensis (Aubl.) Kuntze	Tree	SA		Reflora
Myrsine hermogenesii (Jung-Mend. & Bernacci) M.F.Freitas & Kin.-Gouv.	Tree	ESA		Reflora
Myrsine lancifolia Mart.	Tree	ESA		Reflora
Myrsine leuconeura Mart.	Tree	ESA		Reflora
Myrsine lineata (Mez) Imkhan.	Tree	ESA		Reflora
Myrsine loefgrenii (Mez) Imkhan.	Tree	Regional		Reflora
Myrsine monticola Mart.	Tree	ESA		Reflora
Myrsine parvifolia A.DC.	Tree	ESA		Reflora
Myrsine parvula (Mez) Otegui	Tree	ESA		Reflora
Myrsine rubra M.F.Freitas & Kin.-Gouv.	Tree	Regional		Reflora
Myrsine squarrosa (Mez) M.F.Freitas & Kin.-Gouv.	Shrub	ESA		Reflora
Myrsine umbellata Mart.	Tree	SA		Reflora
Myrsine venosa A.DC.	Tree	ESA		Reflora
Myrsine villosissima Mart.	Shrub	Regional		Reflora
Stylogyne leptantha (Miq.) Mez	Tree	Local		Reflora
Stylogyne lhotzkyana (A.DC.) Mez	Tree	Regional		Reflora

Family (Order) and species	Life form	Geog. distribut.	Threat status	Source
Stylogyne pauciflora Mez	Shrub	Regional		Reflora
Stylogyne warmingii Mez	Tree	Regional		Reflora
PROTEACEAE (Proteales)				
Euplassa cantareirae Sleumer	Tree	Regional	VU	Reflora
Euplassa hoehnei Sleumer	Tree	Local		Reflora
Euplassa itatiaiae Sleumer	Tree	Local		Reflora
Euplassa legalis (Vell.) I.M.Johnst.	Tree	Regional		NeoTrop
Euplassa nebularis Rambo & Sleumer	Tree	Regional		NeoTrop
Euplassa rufa (Loes.) Sleumer	Tree	Regional		NeoTrop
Grevillea robusta A. Cunn. ex R. Br. (*)	Tree	exotic		NeoTrop
Panopsis multiflora (Schott) Ducke	Tree	Regional	VU	Reflora
Roupala consimilis Mez ex Taub.	Tree	ESA		Reflora
Roupala montana Aubl.	Tree	Neotrop.		Reflora
Roupala paulensis Sleumer	Tree	ESA		Reflora
Roupala sculpta Sleumer	Tree	SA	VU	Reflora
QUIINACEAE (Malpighiales)				
Quiina glazovii Engl.	Tree	ESA		Reflora
Quiina magallano-gomesii Schwacke	Tree	Local	VU	Reflora
RHAMNACEAE (Rosales)				
Colubrina glandulosa Perkins	Tree	Neotrop.		Reflora
Colubrina retusa (Pittier) Cowan	Tree	SA	EN	Reflora
Condalia buxifolia Reissek	Tree	ESA		NeoTrop
Hovenia dulcis Thunb. (*)	Tree	exotic		Reflora
Rhamnidium elaeocarpum Reissek	Tree	SA		Reflora
Rhamnidium glabrum Reissek	Tree	ESA		Reflora
Rhamnus sphaerosperma Sw.	Tree	Neotrop.		Reflora
Scutia arenicola (Casar.) Reissek	Shrub	ESA	VU	Reflora
RHIZOPHORACEAE (Malpighiales)				
Rhizophora mangle L.	Tree	Neotrop.		Reflora
ROSACEAE (Rosales)				

Family (Order) and species	Life form	Geog. distribut.	Threat status	Source
Eriobotrya japonica (Thunb.) Lindl. (*)	Tree	exotic		Reflora
Prunus brasiliensis (Cham. & Schltdl.) D.Dietr.	Tree	ESA		Reflora
Prunus myrtifolia (L.) Urb.	Tree	Neotrop.		Reflora
RUBIACEAE (Gentianales)				
Alibertia edulis (Rich.) A.Rich.	Tree	Neotrop.		Reflora
Alseis floribunda Schott	Tree	ESA		Reflora
Alseis latifolia Gleason	Tree	ESA		Reflora
Amaioua guianensis Aubl.	Tree	SA		Reflora
Amaioua intermedia Mart. ex Schult. & Schult.f.	Tree	SA		Reflora
Bathysa australis (A.St.-Hil.) K.Schum.	Tree	ESA		Reflora
Bathysa gymnocarpa K.Schum.	Tree	Local		Reflora
Bathysa mendoncaei K.Schum.	Tree	Regional		Reflora
Bathysa stipulata (Vell.) C.Presl	Tree	Regional		Reflora
Chomelia bella (Standl.) Steyerm.	Tree	Local		Reflora
Chomelia brasiliana A.Rich.	Shrub	Regional		Reflora
Chomelia intercedens Müll.Arg.	Tree	Regional		Reflora
Chomelia obtusa Cham. & Schltdl.	Tree	ESA		Reflora
Chomelia parvifolia (Standl.) Govaerts	Tree	Regional		Reflora
Chomelia pedunculosa Benth.	Tree	ESA		Reflora
Chomelia pohliana Müll.Arg.	Tree	ESA		Reflora
Chomelia ribesioides Benth. ex A.Gray	Tree	ESA		NeoTrop
Coffea liberica Hiern (*)	Tree	exotic		Reflora
Cordiera concolor (Cham.) Kuntze	Tree	ESA		Reflora
Cordiera elliptica (Cham.) Kuntze	Tree	ESA		Reflora
Cordiera longiflora (K.Schum.) Kuntze	Tree	Local		NeoTrop
Cordiera macrophylla (K.Schum.) Kuntze	Tree	SA		Reflora
Cordiera myrciifolia (K.Schum.) C.H.Perss. & Delprete	Tree	Neotrop.		Reflora
Cordiera sessilis (Vell.) Kuntze	Tree	ESA		Reflora
Coussarea accedens Müll.Arg.	Tree	Local		Reflora

Family (Order) and species	Life form	Geog. distribut.	Threat status	Source
Coussarea bocainae M.Gomes	Tree	Local	VU	Reflora
Coussarea contracta (Walp.) Müll.Arg.	Tree	ESA		Reflora
Coussarea graciliflora (Mart.) Müll.Arg.	Tree	Regional		NeoTrop
Coussarea hydrangeifolia (Benth.) Müll.Arg.	Tree	SA	EN	Reflora
Coussarea meridionalis (Vell.) Müll.Arg.	Tree	Local		Reflora
Coussarea nodosa (Benth.) Müll.Arg.	Shrub	ESA	EN	Reflora
Coussarea platyphylla Müll.Arg.	Tree	SA		Reflora
Coutarea hexandra (Jacq.) K.Schum.	Tree	Neotrop.		Reflora
Faramea hyacinthina Mart.	Tree	ESA		NeoTrop
Faramea hymenocalyx M.Gomes	Tree	Local		Reflora
Faramea latifolia (Cham. & Schltdl.) DC.	Tree	ESA		Reflora
Faramea martiana Müll.Arg.	Tree	ESA		Reflora
Faramea montevidensis (Cham. & Schltdl.) DC.	Tree	ESA		Reflora
Faramea multiflora A.Rich. ex DC.	Tree	SA		Reflora
Faramea pachyantha Müll.Arg.	Tree	Regional		Reflora
Faramea paratiensis M.Gomes	Tree	Local	VU	Reflora
Faramea picinguabae M.Gomes	Shrub	Local		Reflora
Faramea stipulacea (Cham. & Schltdl.) DC.	Tree	Regional		Reflora
Faramea tetragona Müll.Arg.	Tree	Local		Reflora
Faramea truncata (Vell.) Müll.Arg.	Tree	Regional		Reflora
Genipa americana L.	Tree	Neotrop.		Reflora
Genipa infundibuliformis Zappi & Semir	Tree	Regional		Reflora
Guettarda platyphylla Müll.Arg.	Tree	Regional	EN	Reflora
Guettarda pohliana Müll.Arg.	Tree	ESA		Reflora
Guettarda uruguensis Cham. & Schltdl.	Tree	ESA		Reflora
Guettarda viburnoides Cham. & Schltdl.	Tree	ESA		Reflora
Hamelia patens Jacq.	Shrub	Neotrop.		Reflora
Ixora bracteolaris Müll.Arg.	Shrub	Local		NeoTrop
Ixora brevifolia Benth.	Tree	ESA		Reflora
Ixora burchelliana Müll.Arg.	Tree	Local		Reflora

Family (Order) and species	Life form	Geog. distribut.	Threat status	Source
Ixora gardneriana Benth.	Tree	Regional		Reflora
Ixora schottiana Müll.Arg.	Tree	Regional		Reflora
Ixora venulosa Benth.	Tree	ESA		Reflora
Ladenbergia hexandra (Pohl) Klotzsch	Tree	Regional	VU	Reflora
Machaonia brasiliensis (Hoffmanns. ex Humb.) Cham. & Schltdl.	Tree	SA		Reflora
Margaritopsis astrellantha (Wernham) L.Andersson	Shrub	SA		NeoTrop
Margaritopsis cephalantha (Müll.Arg.) C.M.Taylor	Tree	SA		NeoTrop
Margaritopsis chaenotricha (DC.) C.M.Taylor	Shrub	Regional		Reflora
Margaritopsis cymuligera (Müll.Arg.) C.M.Taylor	Shrub	Regional		NeoTrop
Palicourea macrobotrys (Ruiz & Pav.) Schult.	Tree	SA		Reflora
Palicourea marcgravii A.St.-Hil.	Shrub	ESA		Reflora
Palicourea rigida Kunth	Tree	SA		Reflora
Posoqueria acutifolia Mart.	Tree	Regional		Reflora
Posoqueria latifolia (Rudge) Schult.	Tree	Neotrop.		Reflora
Posoqueria palustris Mart.	Tree	Regional	VU	Reflora
Psychotria anceps Kunth	Tree	SA		Reflora
Psychotria appendiculata Müll.Arg.	Shrub	Regional		NeoTrop
Psychotria ararum C.M.Taylor	Tree	Local		Reflora
Psychotria beyrichiana Müll.Arg.	Tree	Local		Reflora
Psychotria brachypoda (Müll.Arg.) Britton	Tree	Regional		Reflora
Psychotria capitata Ruiz & Pav.	Shrub	SA	EN	NeoTrop
Psychotria carthagenensis Jacq.	Tree	Neotrop.		Reflora
Psychotria cupularis (Müll.Arg.) Standl.	Shrub	SA	VU	Reflora
Psychotria deflexa DC.	Shrub	Neotrop.		Reflora
Psychotria fluminensis Vell.	Shrub	Local		Reflora
Psychotria formosa Cham. & Schltdl.	Tree	Regional		Reflora
Psychotria forsteronioides Müll.Arg.	Shrub	Regional		Reflora

Family (Order) and species	Life form	Geog. distribut.	Threat status	Source
Psychotria glaziovii Müll.Arg.	Tree	Local		Reflora
Psychotria hastisepala Müll.Arg.	Shrub	Regional		Reflora
Psychotria hoffmannseggiana (Willd. ex Schult.) Müll.Arg.	Shrub	SA		Reflora
Psychotria laciniata Vell.	Shrub	Regional		Reflora
Psychotria leiocarpa Cham. & Schltdl.	Shrub	ESA		Reflora
Psychotria leitana C.M.Taylor	Shrub	Local		NeoTrop
Psychotria mapourioides DC.	Tree	SA		Reflora
Psychotria mima Standl.	Shrub	Local		Reflora
Psychotria myriantha Müll.Arg.	Shrub	ESA		Reflora
Psychotria nemorosa Gardner	Tree	ESA		Reflora
Psychotria nuda (Cham. & Schltdl.) Wawra	Tree	ESA		Reflora
Psychotria pubigera Schltdl.	Shrub	ESA		Reflora
Psychotria subspathulata (Müll.Arg.) C.M.Taylor	Shrub	Regional		Reflora
Psychotria suterella Müll.Arg.	Tree	ESA		Reflora
Psychotria vellosiana Benth.	Tree	SA		Reflora
Randia armata (Sw.) DC.	Tree	Neotrop.		Reflora
Randia calycina Cham.	Shrub	SA		Reflora
Randia ferox (Cham. & Schltdl.) DC.	Tree	Regional		Reflora
Rudgea coriacea (Spreng.) K.Schum.	Tree	Regional		Reflora
Rudgea coronata (Vell.) Müll.Arg.	Shrub	Regional		Reflora
Rudgea gardenioides (Cham.) Müll.Arg.	Tree	Regional		Reflora
Rudgea jasminoides (Cham.) Müll.Arg.	Tree	ESA		Reflora
Rudgea minor (Cham.) Standl.	Shrub	Local		Reflora
Rudgea nobilis Müll.Arg.	Tree	Local	VU	Reflora
Rudgea nodosa (Cham.) Benth.	Shrub	Regional		Reflora
Rudgea pachyphylla Müll.Arg.	Tree	Local	EX	Reflora
Rudgea parquioides (Cham.) Müll.Arg.	Shrub	Regional		NeoTrop
Rudgea recurva Müll.Arg.	Tree	ESA		Reflora
Rudgea sessilis (Vell.) Müll.Arg.	Tree	Regional	EN	Reflora

Family (Order) and species	Life form	Geog. distribut.	Threat status	Source
Rudgea triflora Benth.	Shrub	Local	VU	Reflora
Rudgea vellerea Müll.Arg.	Tree	Local		Reflora
Rudgea viburnoides (Cham.) Benth.	Tree	SA		Reflora
Rustia angustifolia K.Schum.	Tree	Local	VU	Reflora
Rustia formosa (Cham. & Schltdl.) Klotzsch	Tree	ESA		Reflora
Schizocalyx cuspidatus (A.St.-Hil.) Kainul. & B. Bremer	Tree	ESA	VU	Reflora
Simira corumbensis (Standl.) Steyerm.	Tree	SA		Reflora
Simira pikia (K.Schum.) Steyerm.	Tree	Local		Reflora
Simira rubra (Mart.) Steyerm.	Tree	Local		NeoTrop
Simira sampaioana (Standl.) Steyerm.	Tree	ESA		Reflora
Simira viridiflora (Allemão & Saldanha) Steyerm.	Tree	ESA	CR	Reflora
Tocoyena brasiliensis Mart.	Tree	ESA		Reflora
Tocoyena bullata (Vell.) Mart.	Tree	ESA		Reflora
Tocoyena formosa (Cham. & Schltdl.) K.Schum.	Tree	SA		Reflora
RUTACEAE (Sapindales)				
Almeidea lilacina A.St.-Hil.	Tree	Regional		Reflora
Balfourodendron riedelianum (Engl.) Engl.	Tree	ESA		Reflora
Citrus reticulata Blanco (*)	Tree	exotic		Reflora
Citrus X *limon* (L.) Osbeck (*)	Tree	exotic		Reflora
Conchocarpus fontanesianus (A. St.-Hil.) Kallunki & Pirani	Shrub	Local		Reflora
Conchocarpus pentandrus (A. St.-Hil.) Kallunki & Pirani	Tree	ESA		Reflora
Dictyoloma vandellianum A.Juss.	Tree	SA		Reflora
Esenbeckia febrifuga (A.St.-Hil.) A. Juss. ex Mart.	Tree	ESA		Reflora
Esenbeckia grandiflora Mart.	Tree	SA		Reflora
Esenbeckia hieronymi Engl.	Tree	Regional		Reflora
Esenbeckia leiocarpa Engl.	Tree	ESA		Reflora
Esenbeckia pilocarpoides Kunth	Tree	Neotrop.	EN	Reflora
Galipea jasminiflora (A.St.-Hil.) Engl.	Tree	ESA		Reflora

Family (Order) and species	Life form	Geog. distribut.	Threat status	Source
Helietta apiculata Benth.	Tree	ESA		Reflora
Hortia brasiliana Vand. ex DC.	Tree	ESA		Reflora
Metrodorea nigra A.St.-Hil.	Tree	ESA		Reflora
Metrodorea stipularis Mart.	Tree	ESA		Reflora
Murraya paniculata (L.) Jack (*)	Tree	exotic		Reflora
Neoraputia magnifica (Engl.) Emmerich ex Kallunki	Tree	ESA		Reflora
Pilocarpus giganteus Engl.	Tree	Regional	VU	Reflora
Pilocarpus pauciflorus A.St.-Hil.	Tree	ESA		Reflora
Pilocarpus pennatifolius Lem.	Tree	ESA		Reflora
Pilocarpus spicatus A.St.-Hil.	Tree	ESA		Reflora
Zanthoxylum acuminatum (Sw.) Sw.	Tree	Neotrop.		Reflora
Zanthoxylum caribaeum Lam.	Tree	SA		Reflora
Zanthoxylum fagara (L.) Sarg.	Tree	Neotrop.		Reflora
Zanthoxylum monogynum A.St.-Hil.	Tree	ESA		Reflora
Zanthoxylum petiolare A.St.-Hil. & Tul.	Tree	ESA	VU	Reflora
Zanthoxylum rhoifolium Lam.	Tree	Neotrop.		Reflora
Zanthoxylum riedelianum Engl.	Tree	Neotrop.		Reflora
Zanthoxylum rigidum Humb. & Bonpl. ex Willd.	Tree	SA		NeoTrop
Zanthoxylum tingoassuiba A.St.-Hil.	Tree	ESA		Reflora
SABIACEAE (Proteales)				
Meliosma chartacea Lombardi	Tree	Regional		Reflora
Meliosma itatiaiae Urb.	Tree	Regional		Reflora
Meliosma sellowii Urb.	Tree	ESA		Reflora
SALICACEAE (Malpighiales)				
Azara uruguayensis (Speg.) Sleumer	Tree	ESA		Reflora
Banara parviflora (A.Gray) Benth.	Tree	Regional		Reflora
Banara serrata (Vell.) Warb.	Tree	Regional		Reflora
Banara tomentosa Clos	Tree	ESA		Reflora
Casearia aculeata Jacq.	Tree	Neotrop.		Reflora

Family (Order) and species	Life form	Geog. distribut.	Threat status	Source
Casearia arborea (Rich.) Urb.	Tree	Neotrop.		Reflora
Casearia decandra Jacq.	Tree	Neotrop.		Reflora
Casearia gossypiosperma Briq.	Tree	SA		Reflora
Casearia grandiflora Cambess.	Tree	Neotrop.		Reflora
Casearia lasiophylla Eichler	Tree	ESA		Reflora
Casearia mariquitensis Kunth	Tree	SA		Reflora
Casearia melliodora Eichler	Tree	Regional		NeoTrop
Casearia obliqua Spreng.	Tree	ESA		Reflora
Casearia paranaensis Sleumer	Tree	Local		Reflora
Casearia rupestris Eichler	Tree	ESA		Reflora
Casearia selloana Eichler	Shrub	ESA		Reflora
Casearia sylvestris Sw.	Tree	SA		Reflora
Prockia crucis P.Browne ex L.	Tree	Neotrop.		Reflora
Salix humboldtiana Willd.	Tree	Neotrop.		Reflora
Xylosma ciliatifolia (Clos) Eichler	Tree	SA		Reflora
Xylosma glaberrimum Sleumer	Tree	Regional		Reflora
Xylosma prockia (Turcz.) Turcz.	Tree	ESA		Reflora
Xylosma pseudosalzmanii Sleumer	Tree	Regional		Reflora
Xylosma tweediana (Clos) Eichler	Tree	Regional		Reflora
Xylosma venosa N.E.Br.	Tree	ESA		Reflora
SAPINDACEAE (Sapindales)				
Allophylus edulis (A.St.-Hil. et al.) Hieron. ex Niederl.	Tree	SA		Reflora
Allophylus melanophloeus Radlk.	Tree	Regional		Reflora
Allophylus membranifolius Radlk.	Tree	Local		NeoTrop
Allophylus petiolulatus Radlk.	Tree	ESA		Reflora
Allophylus puberulus (Cambess.) Radlk.	Tree	ESA		Reflora
Allophylus racemosus Sw.	Tree	Neotrop.		Reflora
Allophylus semidentatus (Miq.) Radlk.	Tree	SA	VU	Reflora
Cupania bracteosa Radlk.	Tree	Regional		Reflora

Family (Order) and species	Life form	Geog. distribut.	Threat status	Source
Cupania concolor Radlk.	Tree	Regional	VU	Reflora
Cupania emarginata Cambess.	Tree	ESA		Reflora
Cupania furfuracea Radlk.	Tree	Local	EN	Reflora
Cupania ludowigii Somner & Ferrucci	Tree	Regional		Reflora
Cupania oblongifolia Mart.	Tree	Neotrop.		Reflora
Cupania tenuivalvis Radlk.	Tree	Regional		Reflora
Cupania vernalis Cambess.	Tree	SA		Reflora
Cupania zanthoxyloides Radlk.	Tree	Local		Reflora
Diatenopteryx sorbifolia Radlk.	Tree	SA		Reflora
Dilodendron bipinnatum Radlk.	Tree	SA	VU	Reflora
Dodonaea viscosa Jacq.	Tree	Pantrop.		Reflora
Magonia pubescens A.St.-Hil.	Tree	SA	EN	Reflora
Matayba cristae Reitz	Tree	Regional		Reflora
Matayba elaeagnoides Radlk.	Tree	ESA		Reflora
Matayba guianensis Aubl.	Tree	SA		Reflora
Matayba intermedia Radlk.	Tree	Regional		Reflora
Matayba juglandifolia (Cambess.) Radlk.	Tree	ESA		Reflora
Matayba marginata Radlk.	Tree	ESA		Reflora
Matayba obovata R. Coelho, Souza & Ferrucci	Tree	Regional		Reflora
Sapindus saponaria L.	Tree	Neotrop.		Reflora
Talisia angustifolia Radlk.	Tree	Regional		Reflora
Talisia esculenta (Cambess.) Radlk.	Tree	SA		NeoTrop
Toulicia tomentosa Radlk.	Shrub	Regional		Reflora
Tripterodendron filicifolium Radlk.	Tree	Regional		Reflora
SAPOTACEAE (Ericales)				
Chrysophyllum flexuosum Mart.	Tree	ESA		Reflora
Chrysophyllum gonocarpum (Mart. & Eichler ex Miq.) Engl.	Tree	SA		Reflora
Chrysophyllum imperiale (Linden ex K.Koch & Fintelm.) Benth. & Hook.	Tree	Neotrop.		Reflora
Chrysophyllum inornatum Mart.	Tree	Regional		Reflora

Family (Order) and species	Life form	Geog. distribut.	Threat status	Source
Chrysophyllum marginatum (Hook. & Arn.) Radlk.	Tree	ESA		Reflora
Chrysophyllum paranaense T.D.Penn.	Tree	Local		NeoTrop
Chrysophyllum viride Mart. & Eichler	Tree	ESA		Reflora
Diploon cuspidatum (Hoehne) Cronquist	Tree	SA		Reflora
Ecclinusa ramiflora Mart.	Tree	Neotrop.		Reflora
Manilkara salzmannii (A.DC.) H.J.Lam	Tree	ESA		NeoTrop
Manilkara subsericea (Mart.) Dubard	Tree	ESA		Reflora
Micropholis compta Pierre in Urb.	Tree	ESA		Reflora
Micropholis crassipedicellata (Mart. & Eichler) Pierre	Tree	ESA		Reflora
Micropholis gardneriana (A.DC.) Pierre	Tree	SA		NeoTrop
Micropholis venulosa (Mart. & Eichler) Pierre	Tree	Neotrop.		NeoTrop
Pouteria beaurepairei (Glaz. & Raunk.) Baehni	Tree	Regional		Reflora
Pouteria bullata (S.Moore) Baehni	Tree	ESA		Reflora
Pouteria caimito (Ruiz & Pav.) Radlk.	Tree	Neotrop.		Reflora
Pouteria gardneri (Mart. & Miq.) Baehni	Tree	SA		Reflora
Pouteria gardneriana (A.DC.) Radlk.	Tree	ESA		Reflora
Pouteria glomerata (Miq.) Radlk.	Tree	Neotrop.	EN	Reflora
Pouteria grandiflora (A.DC.) Baehni	Tree	ESA		Reflora
Pouteria oxypetala T.D.Penn.	Tree	Local	CR	Reflora
Pouteria psammophila (Mart.) Radlk.	Tree	ESA		Reflora
Pouteria ramiflora (Mart.) Radlk.	Tree	SA		Reflora
Pouteria reticulata (Engl.) Eyma	Tree	Neotrop.	EN	Reflora
Pouteria salicifolia (Spreng.) Radlk.	Tree	ESA		NeoTrop
Pouteria torta (Mart.) Radlk.	Tree	Neotrop.		Reflora
Pouteria venosa (Mart.) Baehni	Tree	ESA		Reflora
Pradosia lactescens (Vell.) Radlk.	Tree	ESA		Reflora
Sideroxylon obtusifolium (Roem. & Schult.) T.D.Penn.	Tree	Neotrop.		Reflora
SCHOEPFIACEAE (Santalales)				

Family (Order) and species	Life form	Geog. distribut.	Threat status	Source
Schoepfia brasiliensis A.DC.	Tree	SA		Reflora
SIMAROUBACEAE (Sapindales)				
Picrasma crenata (Vell.) Engl.	Tree	ESA	EN	Reflora
Simaba insignis A.St.-Hil. & Tul.	Shrub	Regional	VU	Reflora
SIPARUNACEAE (Laurales)				
Siparuna bifida (Poepp. & Endl.) A.DC.	Tree	SA	VU	Reflora
Siparuna brasiliensis (Spreng.) A.DC.	Tree	ESA		Reflora
Siparuna guianensis Aubl.	Tree	Neotrop.		Reflora
Siparuna reginae (Tul.) A.DC.	Tree	SA		Reflora
SOLANACEAE (Solanales)				
Acnistus arborescens (L.) Schltdl.	Tree	Neotrop.		Reflora
Athenaea pereirae Barboza & Hunz.	Shrub	Regional		Reflora
Athenaea pogogena (Moric.) Sendtn.	Shrub	Regional		Reflora
brasiliana (Hunz.) Barboza & Hunz.	Tree	Regional		Reflora
Aureliana cuspidata (Witasek) I.M.C.Rodrigues & Stehmann	Tree	Regional		Reflora
Aureliana fasciculata (Vell.) Sendtn.	Tree	SA		Reflora
Aureliana sellowiana (Sendtn.) Barboza & Stehmann	Tree	Local		Reflora
Aureliana tomentosa Sendtn.	Tree	Regional		Reflora
Aureliana wettsteiniana (Witasek) Hunz. & Barboza	Tree	Regional		Reflora
Brugmansia suaveolens (Willd.) Bercht. & J.Presl	Shrub	Pantrop.		Reflora
Brunfelsia brasiliensis (Spreng.) L.B.Sm. & Downs	Shrub	ESA		Reflora
Brunfelsia pauciflora (Cham. & Schltdl.) Benth.	Tree	Regional		Reflora
Brunfelsia uniflora (Pohl) D.Don	Tree	SA		Reflora
Capsicum flexuosum Sendtn.	Shrub	Regional		Reflora
Capsicum mirabile Mart.	Tree	Regional		NeoTrop
Cestrum axillare Vell.	Tree	ESA		Reflora
Cestrum bracteatum Link & Otto	Tree	ESA		Reflora

Family (Order) and species	Life form	Geog. distribut.	Threat status	Source
Cestrum intermedium Sendtn.	Tree	ESA		Reflora
Cestrum mariquitense Kunth	Tree	Neotrop.		Reflora
Cestrum montanum Miers	Tree	Regional		Reflora
Cestrum pedicellatum Sendtn.	Shrub	Regional		Reflora
Cestrum schlechtendalii G.Don	Tree	Neotrop.		Reflora
Cestrum strictum Schott ex Sendtn.	Shrub	Regional		Reflora
Cestrum strigilatum Ruiz & Pav.	Tree	Neotrop.		Reflora
Cestrum subpulverulentum Mart.	Shrub	Regional		Reflora
Cestrum velutinum Hiern	Tree	ESA		Reflora
Dyssochroma viridiflorum (Sims) Miers	Shrub	ESA		Reflora
Lycianthes pauciflora (Vahl) Bitter	Tree (L)	SA		Reflora
Sessea brasiliensis Toledo	Tree	Local		Reflora
Sessea regnellii Taub.	Tree	ESA		Reflora
Solanum argenteum Dunal	Tree	Neotrop.		Reflora
Solanum betaceum Cav. (*)	Tree	exotic		Reflora
Solanum bullatum Vell.	Tree	Regional		Reflora
Solanum caavurana Vell.	Tree	ESA		Reflora
Solanum campaniforme Roem. & Schult.	Tree	SA		Reflora
Solanum castaneum Carvalho	Shrub	Regional		Reflora
Solanum cernuum Vell.	Tree	ESA		Reflora
Solanum cinnamomeum Sendtn.	Tree	ESA		Reflora
Solanum cladotrichum Dunal	Shrub	Regional		Reflora
Solanum crinitum Lam.	Tree	SA		Reflora
Solanum decorum Sendtn.	Tree	Regional		Reflora
Solanum didymum Dunal	Shrub	SA		Reflora
Solanum diploconos (Mart.) Bohs	Tree	ESA		Reflora
Solanum echidnaeforme Dunal	Tree	Regional		Reflora
Solanum granulosoleprosum Dunal	Tree	ESA		Reflora
Solanum itatiaiae Dusén	Tree	Regional		Reflora
Solanum johannae Bitter	Shrub	Regional		Reflora

Family (Order) and species	Life form	Geog. distribut.	Threat status	Source
Solanum kleinii L.B.Sm. & Downs	Shrub	Regional		Reflora
Solanum lacerdae Dusén	Tree	Regional		Reflora
Solanum latiflorum Bohs	Shrub	Regional		Reflora
Solanum leptostachys Dunal	Tree	Regional		Reflora
Solanum lycocarpum A.St.-Hil.	Tree	ESA		Reflora
Solanum martii Sendtn.	Shrub	Regional		Reflora
Solanum mauritianum Scop.	Tree	ESA		Reflora
Solanum melissarum Bohs	Tree	ESA		Reflora
Solanum oocarpum Sendtn.	Shrub	ESA		Reflora
Solanum pabstii L.B.Sm. & Downs	Tree	Regional		Reflora
Solanum pinetorum (L.B.Sm. & Downs) Bohs	Tree	Regional		Reflora
Solanum pseudoquina A.St.-Hil.	Tree	ESA		Reflora
Solanum ramulosum Sendtn.	Shrub	Regional		Reflora
Solanum rufescens Sendtn.	Tree	ESA		Reflora
Solanum sanctae-catharinae Dunal	Tree	ESA		Reflora
Solanum sciadostylis (Sendtn.) Bohs	Tree	ESA		Reflora
Solanum scuticum M.Nee	Tree	SA		Reflora
Solanum sellowii Dunal	Tree	Regional		Reflora
Solanum swartzianum Roem. & Schult.	Tree	SA		Reflora
Solanum symmetricum Rusby	Tree	ESA		Reflora
Solanum variabile Mart.	Tree	Regional		Reflora
Solanum velleum Thunb.	Tree	ESA		Reflora
Solanum vellozianum Dunal	Tree	Regional		Reflora
Solanum verticillatum S. Knapp & Stehmann	Tree	Regional		Reflora
Vassobia breviflora (Sendtn.) Hunz.	Shrub	ESA		Reflora
STYRACACEAE (Ericales)				
Styrax acuminatus Pohl	Tree	ESA		Reflora
Styrax camporum Pohl	Tree	ESA		Reflora
Styrax ferrugineus Nees & Mart.	Tree	SA		Reflora
Styrax glabratus Schott	Tree	Neotrop.		Reflora

Family (Order) and species	Life form	Geog. distribut.	Threat status	Source
Styrax lancifolius Klotzsch ex Seub.	Shrub	Local		Reflora
Styrax latifolius Pohl	Tree	ESA		Reflora
Styrax leprosus Hook. & Arn.	Tree	ESA		Reflora
Styrax martii Seub.	Tree	ESA		Reflora
Styrax pohlii A.DC.	Tree	SA		Reflora
Styrax sieberi Perkins	Tree	SA		Reflora
SYMPLOCACEAE (Ericales)				
Symplocos atlantica Aranha	Shrub	Local		Reflora
Symplocos bidana Aranha	Tree	Regional		NeoTrop
Symplocos celastrinea Mart.	Tree	ESA		Reflora
Symplocos corymboclados Brand	Tree	Regional		NeoTrop
Symplocos estrellensis Casar.	Tree	ESA		Reflora
Symplocos falcata Brand	Tree	Regional		Reflora
Symplocos glandulosomarginata Hoehne	Tree	Regional		Reflora
Symplocos incrassata Aranha	Shrub	Local		NeoTrop
Symplocos insignis Brand	Tree	Regional		Reflora
Symplocos kleinii Bidá ex Aranha	Shrub	Local		NeoTrop
Symplocos laxiflora Benth.	Tree	Regional		Reflora
Symplocos nitens (Pohl) Benth.	Tree	SA		Reflora
Symplocos nitidiflora Brand	Tree	Regional		Reflora
Symplocos oblongifolia Casar.	Tree	ESA		Reflora
Symplocos pentandra (Mattos) Occhioni ex Aranha	Shrub	ESA		Reflora
Symplocos platyphylla (Pohl) Benth.	Tree	Regional		Reflora
Symplocos pubescens Klotzsch ex Benth.	Tree	ESA		Reflora
Symplocos pustulosa Aranha	Tree	Regional		Reflora
Symplocos revoluta Casar.	Tree	ESA		Reflora
Symplocos rizzinii Occhioni	Tree	Local		Reflora
Symplocos tenuifolia Brand	Tree	ESA		Reflora
Symplocos tetrandra Mart.	Tree	Regional		Reflora

Family (Order) and species	Life form	Geog. distribut.	Threat status	Source
Symplocos trachycarpos Brand	Tree	Regional		Reflora
Symplocos uniflora (Pohl) Benth.	Tree	ESA		Reflora
THEACEAE (Ericales)				
Laplacea fructicosa (Schrad.) Kobuski	Tree	Neotrop.		Reflora
THYMELAEACEAE (Malvales)				
Daphnopsis brasiliensis Mart.	Tree	Regional		Reflora
Daphnopsis coriacea Taub.	Tree	Regional		Reflora
Daphnopsis fasciculata (Meisn.) Nevling	Tree	ESA		Reflora
Daphnopsis martii Meisn.	Tree	Local		Reflora
Daphnopsis racemosa Griseb.	Tree	ESA		Reflora
Daphnopsis schwackeana Taub.	Tree	Local		Reflora
Daphnopsis sellowiana Taub.	Tree	ESA		Reflora
Daphnopsis utilis Warm.	Tree	ESA		Reflora
ULMACEAE (Rosales)				
Phyllostylon rhamnoides (Poiss.) Taub.	Tree	Neotrop.	VU	Reflora
URTICACEAE (Rosales)				
Boehmeria caudata Sw.	Tree	Neotrop.		Reflora
Boehmeria ulmifolia Wedd.	Tree	Neotrop.		Reflora
Cecropia glaziovii Snethl.	Tree	ESA		Reflora
Cecropia hololeuca Miq.	Tree	Regional		Reflora
Cecropia pachystachya Trécul	Tree	SA		Reflora
Coussapoa microcarpa (Schott) Rizzini	Tree (H)	ESA		Reflora
Myriocarpa stipitata Benth.	Tree	SA		Reflora
Pourouma guianensis Aubl.	Tree	SA		Reflora
Urera baccifera (L.) Gaudich. ex Wedd.	Tree	Neotrop.		Reflora
Urera caracasana (Jacq.) Griseb.	Tree	Neotrop.		Reflora
Urera nitida (Vell.) P.Brack	Shrub	ESA		NeoTrop
VERBENACEAE (Lamiales)				
Aloysia virgata (Ruiz & Pav.) Juss.	Tree	SA		Reflora
Citharexylum glaziovii Moldenke	Tree	ESA		Reflora

Family (Order) and species	Life form	Geog. distribut.	Threat status	Source
Citharexylum laetum Hiern	Tree	ESA		Reflora
Citharexylum montevidense (Spreng.) Moldenke	Tree	ESA		Reflora
Citharexylum myrianthum Cham.	Tree	ESA		Reflora
Citharexylum solanaceum Cham.	Tree	ESA		Reflora
Duranta vestita Cham.	Tree	ESA		Reflora
Lantana camara L.	Shrub	SA		Reflora
Petrea volubilis L.	Tree	SA		Reflora
VIOLACEAE (Malpighiales)				
Amphirrhox longifolia (A.St.-Hil.) Spreng.	Tree	Neotrop.		Reflora
Hybanthus atropurpureus (A.St.-Hil.) Taub.	Shrub	ESA		NeoTrop
Paypayrola blanchetiana Tul.	Tree	ESA		Reflora
VOCHYSIACEAE (Myrtales)				
Callisthene castellanosii H.F.Martins	Tree	Regional		Reflora
Callisthene fasciculata Mart.	Tree	SA		Reflora
Callisthene kuhlmannii H.F.Martins	Tree	Regional		NeoTrop
Callisthene major Mart. & Zucc.	Tree	ESA		NeoTrop
Callisthene minor Mart.	Tree	Regional		Reflora
Qualea cordata (Mart.) Spreng.	Tree	ESA		Reflora
Qualea cryptantha (Spreng.) Warm.	Tree	ESA		NeoTrop
Qualea densiflora Warm.	Tree	ESA		Reflora
Qualea dichotoma (Mart.) Warm.	Tree	ESA		Reflora
Qualea gestasiana A.St.-Hil.	Tree	Local		NeoTrop
Qualea glaziovii Warm.	Tree	Local		NeoTrop
Qualea grandiflora Mart.	Tree	SA		Reflora
Qualea multiflora Mart.	Tree	SA		Reflora
Qualea parviflora Mart.	Tree	SA		Reflora
Qualea selloi Warm.	Tree	Regional		Reflora
Salvertia convallariodora A.St.-Hil.	Tree	SA		Reflora
Vochysia acuminata Bong.	Tree	ESA		NeoTrop
Vochysia bifalcata Warm.	Tree	Regional		Reflora

Family (Order) and species	Life form	Geog. distribut.	Threat status	Source
Vochysia cinnamomea Pohl	Tree	ESA		Reflora
Vochysia glazioviana Warm.	Tree	Local		Reflora
Vochysia laurifolia Warm.	Tree	ESA		Reflora
Vochysia magnifica Warm.	Tree	ESA		Reflora
Vochysia oppugnata (Vell.) Warm.	Tree	Regional		Reflora
Vochysia rufa Mart.	Tree	SA		Reflora
Vochysia saldanhana Warm.	Tree	Regional		NeoTrop
Vochysia schwackeana Warm.	Tree	Regional		NeoTrop
Vochysia selloi Warm.	Tree	Local		Reflora
Vochysia thyrsoidea Pohl	Tree	ESA		Reflora
Vochysia tucanorum Mart.	Tree	ESA		Reflora
WINTERACEAE (Canellales)				
Drimys brasiliensis Miers	Tree	ESA		Reflora
