# Supplementary material for: Can plant DNA barcoding be implemented in species-rich tropical regions? A perspective from São Paulo State, Brazil
Source: Genet Mol Biol. 2018 Jul-Sep;41(3):661–70. doi: 10.1590/1678-4685-GMB-2017-0282 (PMC6136365; doi:10.1590/1678-4685-GMB-2017-0282)
Supplement: Supplementary file 5 [file 1415-4757-GMB-41-03-2017-0282-20180723-suppl5.pdf]

Supplementary Material to "Can plant DNA barcoding be implemented in species-rich tropical regions? A perspective from São Paulo State, Brazil"

Table S5 - List of GenBank accessions found for the São Paulo tree flora. For species with larger number of sequences, it is given the accession interval for each barcode. Note that the GenBank accessions for the sequences produced here are listed in the main text.

Family	Species	Accession	Barcode
Acanthaceae	*Avicennia germinans*	AB861082-AB861219	ITS
Acanthaceae	*Avicennia germinans*	AF365979	ITS
Acanthaceae	*Avicennia germinans*	AF531771	matK
Acanthaceae	*Avicennia germinans*	AY008819	trnL
Acanthaceae	*Avicennia germinans*	AY008830	rbcL
Acanthaceae	*Avicennia germinans*	DQ469834-DQ469863	ITS
Acanthaceae	*Avicennia germinans*	EF136920-EF136928	ITS
Acanthaceae	*Avicennia germinans*	EF540979-EF540990	ITS
Acanthaceae	*Avicennia germinans*	EU315869	trnL
Acanthaceae	*Avicennia germinans*	EU352145-EU352148	ITS
Acanthaceae	*Avicennia germinans*	EU529073	trnL
Acanthaceae	*Avicennia germinans*	HG963703	psbA-trnH
Acanthaceae	*Avicennia germinans*	JQ586379	matK
Acanthaceae	*Avicennia germinans*	JQ589991	matK
Acanthaceae	*Avicennia germinans*	JQ590028	rbcL
Acanthaceae	*Avicennia germinans*	JQ590029	rbcL
Acanthaceae	*Avicennia germinans*	JQ590030	rbcL
Acanthaceae	*Avicennia germinans*	JQ594363	rbcL
Acanthaceae	*Avicennia germinans*	JQ594364	rbcL
Acanthaceae	*Avicennia germinans*	JQ594977	rbcL
Acanthaceae	*Avicennia germinans*	JQ594978	rbcL
Acanthaceae	*Avicennia germinans*	JQ594979	rbcL
Acanthaceae	*Avicennia germinans*	JQ594980	rbcL
Acanthaceae	*Avicennia germinans*	JQ594981	rbcL

Family	Species	Accession	Barcode
Acanthaceae	*Avicennia germinans*	KC420634	psbA-trnH
Acanthaceae	*Avicennia germinans*	KJ082127	rbcL
Acanthaceae	*Avicennia germinans*	KJ426610	psbA-trnH
Acanthaceae	*Avicennia germinans*	U28868	rbcL
Acanthaceae	*Avicennia schaueriana*	AB861220-AB861412	ITS
Acanthaceae	*Avicennia schaueriana*	DQ469862	ITS
Acanthaceae	*Avicennia schaueriana*	EF540986	ITS
Acanthaceae	*Ruellia brevifolia*	AB162025	trnL
Acanthaceae	*Ruellia brevifolia*	EF214441	ITS
Acanthaceae	*Ruellia brevifolia*	EF214455	ITS
Acanthaceae	*Ruellia brevifolia*	EF214456	ITS
Acanthaceae	*Ruellia brevifolia*	EF214457	ITS
Acanthaceae	*Ruellia brevifolia*	JX443797	ITS
Acanthaceae	*Ruellia brevifolia*	KF945494	psbA-trnH
Acanthaceae	*Ruellia brevifolia*	KF945505	psbA-trnH
Acanthaceae	*Ruellia brevifolia*	KT004491	ITS
Acanthaceae	*Ruellia brevifolia*	KT075021	trnL
Acanthaceae	*Ruellia brevifolia*	KT161370	psbA-trnH
Acanthaceae	*Ruellia brevifolia*	L12595	rbcL
Achatocarpaceae	*Achatocarpus praecox*	AY042533	matK
Achatocarpaceae	*Achatocarpus praecox*	AY270142	rbcL
Achatocarpaceae	*Achatocarpus praecox*	AY491643	matK
Achatocarpaceae	*Achatocarpus praecox*	AY514845	matK
Adoxaceae	*Sambucus australis*	HQ714444	ITS
Adoxaceae	*Sambucus australis*	U88196	ITS
Anacardiaceae	*Anacardium occidentale*	AB071690	ITS
Anacardiaceae	*Anacardium occidentale*	AY462008	rbcL
Anacardiaceae	*Anacardium occidentale*	AY594459	matK
Anacardiaceae	*Anacardium occidentale*	AY594497	trnL
Anacardiaceae	*Anacardium occidentale*	DQ131556	trnL
Anacardiaceae	*Anacardium occidentale*	HG963848	psbA-trnH
Anacardiaceae	*Anacardium occidentale*	JQ769096	trnL
Anacardiaceae	*Anacardium occidentale*	KF664192	ITS
Anacardiaceae	*Anacardium occidentale*	KF664216	trnL
Anacardiaceae	*Anacardium occidentale*	KF664313	psbA-trnH

Family	Species	Accession	Barcode
Anacardiaceae	*Astronium graveolens*	AY594492	matK
Anacardiaceae	*Astronium graveolens*	GQ981674	rbcL
Anacardiaceae	*Astronium graveolens*	GQ982155	psbA-trnH
Anacardiaceae	*Astronium graveolens*	JQ586469	matK
Anacardiaceae	*Astronium graveolens*	JQ586470	matK
Anacardiaceae	*Astronium graveolens*	JQ586471	matK
Anacardiaceae	*Lithrea molleoides*	AY594470	matK
Anacardiaceae	*Lithrea molleoides*	AY594554	trnL
Anacardiaceae	*Lithrea molleoides*	AY640463	trnL
Anacardiaceae	*Lithrea molleoides*	AY641512	ITS
Anacardiaceae	*Lithrea molleoides*	FM179923	matK
Anacardiaceae	*Lithrea molleoides*	JX495755	matK
Anacardiaceae	*Lithrea molleoides*	JX517745	matK
Anacardiaceae	*Lithrea molleoides*	JX571890	rbcL
Anacardiaceae	*Lithrea molleoides*	JX572951	rbcL
Anacardiaceae	*Lithrea molleoides*	KF420989	ITS
Anacardiaceae	*Lithrea molleoides*	KF421075	psbA-trnH
Anacardiaceae	*Lithrea molleoides*	KF555405	matK
Anacardiaceae	*Lithrea molleoides*	KF561924	rbcL
Anacardiaceae	*Lithrea molleoides*	KF664329	psbA-trnH
Anacardiaceae	*Lithrea molleoides*	U39270	rbcL
Anacardiaceae	*Mangifera indica*	GQ435225	psbA-trnH
Anacardiaceae	*Mangifera indica*	HG963847	psbA-trnH
Anacardiaceae	*Mangifera indica*	JF739081	rbcL
Anacardiaceae	*Mangifera indica*	JF739088	rbcL
Anacardiaceae	*Mangifera indica*	JX856471	ITS
Anacardiaceae	*Mangifera indica*	JX856472	ITS
Anacardiaceae	*Mangifera indica*	KF664199	ITS
Anacardiaceae	*Mangifera indica*	KF664223	trnL
Anacardiaceae	*Mangifera indica*	KF664320	psbA-trnH
Anacardiaceae	*Mangifera indica*	KJ833758-KJ833767	ITS
Anacardiaceae	*Mangifera indica*	KR028419-KR071821	rpoC1
Anacardiaceae	*Mangifera indica*	KR071822-KR071839	psbA-trnH
Anacardiaceae	*Mangifera indica*	KR422626-KR422643	rpoB
Anacardiaceae	*Mangifera indica*	KR610374-KR610391	rbcL

Family	Species	Accession	Barcode
Anacardiaceae	*Myracrodruon urundeuva*	AY594560	trnL
Anacardiaceae	*Myracrodruon urundeuva*	DQ787397	ITS
Anacardiaceae	*Myracrodruon urundeuva*	EF513743	psbA-trnH
Anacardiaceae	*Myracrodruon urundeuva*	EF513744	psbA-trnH
Anacardiaceae	*Myracrodruon urundeuva*	EF513745	psbA-trnH
Anacardiaceae	*Schinus terebinthifolius*	AY864894	ITS
Anacardiaceae	*Schinus terebinthifolius*	GU134992	matK
Anacardiaceae	*Schinus terebinthifolius*	GU135156	rbcL
Anacardiaceae	*Schinus terebinthifolius*	GU135324	psbA-trnH
Anacardiaceae	*Schinus terebinthifolius*	JF804927	trnL
Anacardiaceae	*Schinus terebinthifolius*	JQ412287	matK
Anacardiaceae	*Schinus terebinthifolius*	JQ412419	rbcL
Anacardiaceae	*Schinus terebinthifolius*	JX518124	matK
Anacardiaceae	*Schinus terebinthifolius*	JX571891	rbcL
Anacardiaceae	*Schinus terebinthifolius*	JX572952	rbcL
Anacardiaceae	*Schinus terebinthifolius*	KF420939	ITS
Anacardiaceae	*Schinus terebinthifolius*	KF420940	ITS
Anacardiaceae	*Schinus terebinthifolius*	KF420941	ITS
Anacardiaceae	*Schinus terebinthifolius*	KF420942	ITS
Anacardiaceae	*Schinus terebinthifolius*	KF420943	ITS
Anacardiaceae	*Schinus terebinthifolius*	KF420944	ITS
Anacardiaceae	*Schinus terebinthifolius*	KF421076	psbA-trnH
Anacardiaceae	*Schinus terebinthifolius*	KF421077	psbA-trnH
Anacardiaceae	*Schinus terebinthifolius*	KF421078	psbA-trnH
Anacardiaceae	*Schinus terebinthifolius*	KF421079	psbA-trnH
Anacardiaceae	*Schinus terebinthifolius*	KF555445	matK
Anacardiaceae	*Schinus terebinthifolius*	KF555446	matK
Anacardiaceae	*Schinus terebinthifolius*	KF555447	matK
Anacardiaceae	*Schinus terebinthifolius*	KF561967	rbcL
Anacardiaceae	*Schinus terebinthifolius*	KF561968	rbcL
Anacardiaceae	*Schinus terebinthifolius*	KJ012760	matK
Anacardiaceae	*Schinus terebinthifolius*	KJ082554	rbcL
Anacardiaceae	*Schinus terebinthifolius*	KJ426922	psbA-trnH
Anacardiaceae	*Schinus terebinthifolius*	KP055563	trnL
Anacardiaceae	*Schinus terebinthifolius*	KP149521	matK

Family	Species	Accession	Barcode
Anacardiaceae	*Schinus terebinthifolius*	KP149551	rbcL
Anacardiaceae	*Schinus terebinthifolius*	KP398504	trnL
Anacardiaceae	*Schinus terebinthifolius*	KP398505	trnL
Anacardiaceae	*Schinus terebinthifolius*	KP398506	ITS
Anacardiaceae	*Schinus terebinthifolius*	KP398507	ITS
Anacardiaceae	*Spondias mombin*	AF080064	ITS
Anacardiaceae	*Spondias mombin*	AF445882	ITS
Anacardiaceae	*Spondias mombin*	AY594480	matK
Anacardiaceae	*Spondias mombin*	AY594577	trnL
Anacardiaceae	*Spondias mombin*	GQ981882	rbcL
Anacardiaceae	*Spondias mombin*	GQ982370	psbA-trnH
Anacardiaceae	*Spondias mombin*	JQ590139	rbcL
Anacardiaceae	*Spondias mombin*	JQ590140	rbcL
Anacardiaceae	*Spondias mombin*	KC283103	trnL
Anacardiaceae	*Spondias mombin*	KJ026805	psbA-trnH
Anacardiaceae	*Spondias mombin*	KJ026806	psbA-trnH
Anacardiaceae	*Spondias mombin*	KJ026807	psbA-trnH
Anacardiaceae	*Spondias mombin*	KJ026808	psbA-trnH
Anacardiaceae	*Spondias mombin*	KJ026809	psbA-trnH
Anacardiaceae	*Spondias mombin*	KJ026810	psbA-trnH
Anacardiaceae	*Spondias mombin*	KJ026811	psbA-trnH
Anacardiaceae	*Spondias mombin*	KJ026812	psbA-trnH
Anacardiaceae	*Spondias mombin*	KJ026813	psbA-trnH
Anacardiaceae	*Spondias mombin*	KJ026814	psbA-trnH
Anacardiaceae	*Spondias mombin*	KJ026815	psbA-trnH
Anacardiaceae	*Spondias mombin*	KJ026816	psbA-trnH
Anacardiaceae	*Spondias mombin*	KP055575	trnL
Anacardiaceae	*Spondias mombin*	KP774609	matK
Anacardiaceae	*Spondias mombin*	KP774611	matK
Anacardiaceae	*Spondias mombin*	KP774615	rbcL
Anacardiaceae	*Spondias mombin*	KP774617	rbcL
Anacardiaceae	*Spondias mombin*	KP774618	rbcL
Anacardiaceae	*Spondias mombin*	KP789466	psbA-trnH
Anacardiaceae	*Spondias mombin*	KP789469	psbA-trnH
Anacardiaceae	*Tapirira guianensis*	DQ787402	ITS

Family	Species	Accession	Barcode
Anacardiaceae	*Tapirira guianensis*	JQ626278	rbcL
Anacardiaceae	*Tapirira guianensis*	KP055579	trnL
Anacardiaceae	*Tapirira guianensis*	KP055580	trnL
Anacardiaceae	*Tapirira obtusa*	AY594482	matK
Anacardiaceae	*Tapirira obtusa*	AY594579	trnL
Anacardiaceae	*Tapirira obtusa*	GU935446	rbcL
Anacardiaceae	*Tapirira obtusa*	JQ625925	rbcL
Anacardiaceae	*Tapirira obtusa*	JQ626383	matK
Annonaceae	*Anaxagorea dolichocarpa*	AY578311	psbA-trnH
Annonaceae	*Anaxagorea dolichocarpa*	AY580027	trnL
Annonaceae	*Anaxagorea dolichocarpa*	AY580044	trnL
Annonaceae	*Annona cornifolia*	EU420855	rbcL
Annonaceae	*Annona glabra*	AY819068	trnL
Annonaceae	*Annona glabra*	AY841596	rbcL
Annonaceae	*Annona glabra*	AY841673	trnL
Annonaceae	*Annona glabra*	DQ125050	matK
Annonaceae	*Annona glabra*	DQ125116	psbA-trnH
Annonaceae	*Annona glabra*	GQ139717	matK
Annonaceae	*Annona glabra*	GQ139857	psbA-trnH
Annonaceae	*Annona glabra*	GQ139891	trnL
Annonaceae	*Annona glabra*	GU937365	ycf1
Annonaceae	*Annona glabra*	JQ586484	matK
Annonaceae	*Annona glabra*	JQ586485	matK
Annonaceae	*Annona glabra*	JQ586486	matK
Annonaceae	*Annona glabra*	JQ586487	matK
Annonaceae	*Annona glabra*	JQ590152	rbcL
Annonaceae	*Annona glabra*	JQ590153	rbcL
Annonaceae	*Annona glabra*	JQ590154	rbcL
Annonaceae	*Annona glabra*	JQ742022	trnL
Annonaceae	*Annona glabra*	KJ012462	matK
Annonaceae	*Annona glabra*	KJ082120	rbcL
Annonaceae	*Annona glabra*	KJ426605	psbA-trnH
Annonaceae	*Annona glabra*	KM068861	matK
Annonaceae	*Annona glabra*	KM068862	matK
Annonaceae	*Annona glabra*	KM068863	matK

**Family**	**Species**	**Accession**	**Barcode**
Annonaceae	*Annona glabra*	KM068882	rbcL
Annonaceae	*Annona glabra*	KM068883	rbcL
Annonaceae	*Annona glabra*	KM068884	rbcL
Annonaceae	*Annona montana*	AY819072	trnL
Annonaceae	*Annona montana*	EU420842	trnL
Annonaceae	*Annona montana*	EU420860	rbcL
Annonaceae	*Annona montana*	KJ012463	matK
Annonaceae	*Annona montana*	KJ082121	rbcL
Annonaceae	*Annona montana*	KJ082122	rbcL
Annonaceae	*Annona montana*	KJ426606	psbA-trnH
Annonaceae	*Annona montana*	KJ426607	psbA-trnH
Annonaceae	*Annona mucosa*	AY819065	trnL
Annonaceae	*Annona mucosa*	EU420852	trnL
Annonaceae	*Annona mucosa*	EU420870	rbcL
Annonaceae	*Annona mucosa*	GQ139705	matK
Annonaceae	*Annona mucosa*	GQ139845	psbA-trnH
Annonaceae	*Annona mucosa*	GQ139880	trnL
Annonaceae	*Annona mucosa*	GU937353	ycf1
Annonaceae	*Annona rugulosa*	JX880394	matK
Annonaceae	*Annona rugulosa*	JX880395	rbcL
Annonaceae	*Duguetia furfuracea*	KP859337	trnL
Annonaceae	*Duguetia salicifolia*	AY738175	rbcL
Annonaceae	*Duguetia salicifolia*	AY740555	matK
Annonaceae	*Duguetia salicifolia*	AY740587	trnL
Annonaceae	*Guatteria australis*	AY740915	matK
Annonaceae	*Guatteria australis*	AY740943	matK
Annonaceae	*Guatteria australis*	AY740964	rbcL
Annonaceae	*Guatteria australis*	AY740992	rbcL
Annonaceae	*Guatteria australis*	AY741013	trnL
Annonaceae	*Guatteria australis*	AY741041	trnL
Annonaceae	*Guatteria australis*	DQ124948	rbcL
Annonaceae	*Guatteria australis*	DQ125006	trnL
Annonaceae	*Guatteria australis*	DQ125072	matK
Annonaceae	*Guatteria australis*	DQ125155	psbA-trnH
Annonaceae	*Guatteria australis*	DQ125189	psbA-trnH

**Family**	**Species**	**Accession**	**Barcode**
Annonaceae	*Guatteria australis*	DQ861675	trnL
Annonaceae	*Guatteria australis*	DQ861710	matK
Annonaceae	*Guatteria australis*	DQ861716	matK
Annonaceae	*Guatteria australis*	DQ861756	psbA-trnH
Annonaceae	*Guatteria australis*	DQ861762	psbA-trnH
Annonaceae	*Guatteria australis*	DQ861806	rbcL
Annonaceae	*Guatteria australis*	DQ861858	trnL
Annonaceae	*Guatteria australis*	DQ861865	trnL
Annonaceae	*Guatteria latifolia*	AY740933	matK
Annonaceae	*Guatteria latifolia*	AY740982	rbcL
Annonaceae	*Guatteria latifolia*	AY741031	trnL
Annonaceae	*Guatteria latifolia*	DQ125170	psbA-trnH
Annonaceae	*Guatteria latifolia*	DQ861673	trnL
Annonaceae	*Guatteria pohliana*	DQ124964	rbcL
Annonaceae	*Guatteria pohliana*	DQ125022	trnL
Annonaceae	*Guatteria pohliana*	DQ125088	matK
Annonaceae	*Guatteria pohliana*	DQ125193	psbA-trnH
Annonaceae	*Guatteria sellowiana*	AY740954	matK
Annonaceae	*Guatteria sellowiana*	AY741003	rbcL
Annonaceae	*Guatteria sellowiana*	AY741052	trnL
Annonaceae	*Guatteria sellowiana*	AY841624	rbcL
Annonaceae	*Guatteria sellowiana*	AY841702	trnL
Annonaceae	*Guatteria sellowiana*	DQ125212	psbA-trnH
Annonaceae	*Guatteria sellowiana*	DQ861674	trnL
Annonaceae	*Xylopia aromatica*	KP052711	matK
Annonaceae	*Xylopia aromatica*	KP052727	psbA-trnH
Annonaceae	*Xylopia aromatica*	KP052735	trnL
Apocynaceae	*Aspidosperma australe*	AF214159	trnL
Apocynaceae	*Aspidosperma australe*	AF214313	trnL
Apocynaceae	*Aspidosperma australe*	DQ660502	matK
Apocynaceae	*Aspidosperma australe*	DQ660632	rbcL
Apocynaceae	*Aspidosperma cuspa*	AF214161	trnL
Apocynaceae	*Aspidosperma cuspa*	AF214315	trnL
Apocynaceae	*Aspidosperma cylindrocarpon*	DQ660503	matK
Apocynaceae	*Aspidosperma cylindrocarpon*	DQ660633	rbcL

Family	Species	Accession	Barcode
Apocynaceae	*Aspidosperma spruceanum*	FJ037792	ITS
Apocynaceae	*Aspidosperma spruceanum*	FJ037963	rbcL
Apocynaceae	*Aspidosperma spruceanum*	FJ038222	rpoB
Apocynaceae	*Aspidosperma spruceanum*	FJ038422	rpoC1
Apocynaceae	*Aspidosperma spruceanum*	FJ038856	psbA-trnH
Apocynaceae	*Aspidosperma spruceanum*	FJ039102	trnL
Apocynaceae	*Aspidosperma spruceanum*	FJ514713	matK
Apocynaceae	*Aspidosperma spruceanum*	GQ428619	rbcL
Apocynaceae	*Aspidosperma spruceanum*	GQ428620	rbcL
Apocynaceae	*Aspidosperma spruceanum*	GQ428748	psbA-trnH
Apocynaceae	*Aspidosperma spruceanum*	GQ981672	rbcL
Apocynaceae	*Aspidosperma spruceanum*	GQ981941	matK
Apocynaceae	*Aspidosperma spruceanum*	GQ982153	psbA-trnH
Apocynaceae	*Aspidosperma spruceanum*	JQ625998	rbcL
Apocynaceae	*Aspidosperma spruceanum*	JQ626066	rbcL
Apocynaceae	*Aspidosperma spruceanum*	JQ626435	matK
Apocynaceae	*Aspidosperma spruceanum*	JQ626476	matK
Apocynaceae	*Calotropis procera*	AF214170	trnL
Apocynaceae	*Calotropis procera*	AF214324	trnL
Apocynaceae	*Calotropis procera*	AJ419736	rbcL
Apocynaceae	*Calotropis procera*	AJ428795	trnL
Apocynaceae	*Calotropis procera*	AJ428796	trnL
Apocynaceae	*Calotropis procera*	AJ428797	trnL
Apocynaceae	*Calotropis procera*	AM396900	ITS
Apocynaceae	*Calotropis procera*	EU196255	rbcL
Apocynaceae	*Calotropis procera*	HE805509	trnL
Apocynaceae	*Calotropis procera*	HG963787	psbA-trnH
Apocynaceae	*Calotropis procera*	KF425762	rbcL
Apocynaceae	*Calotropis procera*	KT344854	matK
Apocynaceae	*Hancornia speciosa*	DQ660519	matK
Apocynaceae	*Hancornia speciosa*	DQ660646	rbcL
Apocynaceae	*Rauvolfia sellowii*	DQ660537	matK
Apocynaceae	*Rauvolfia sellowii*	DQ660662	rbcL
Apocynaceae	*Tabernaemontana catharinensis*	DQ660549	matK
Apocynaceae	*Tabernaemontana catharinensis*	DQ660672	rbcL

Family	Species	Accession	Barcode
Apocynaceae	*Tabernaemontana hystrix*	GU973942	matK
Apocynaceae	*Tabernaemontana laeta*	GU973944	matK
Apocynaceae	*Thevetia peruviana*	AF214174	trnL
Apocynaceae	*Thevetia peruviana*	AF214282	trnL
Apocynaceae	*Thevetia peruviana*	AF214328	trnL
Apocynaceae	*Thevetia peruviana*	AF214436	trnL
Apocynaceae	*Thevetia peruviana*	EF456088	trnL
Apocynaceae	*Thevetia peruviana*	EU916732	rbcL
Apocynaceae	*Thevetia peruviana*	GQ220741	rpoC1
Apocynaceae	*Thevetia peruviana*	HE805540	trnL
Apocynaceae	*Thevetia peruviana*	HG963821	psbA-trnH
Apocynaceae	*Thevetia peruviana*	JN228929	matK
Apocynaceae	*Thevetia peruviana*	JN245983	psbA-trnH
Apocynaceae	*Thevetia peruviana*	JN416982	matK
Apocynaceae	*Thevetia peruviana*	JX571904	rbcL
Apocynaceae	*Thevetia peruviana*	KJ436389	ITS
Apocynaceae	*Thevetia peruviana*	KJ436390	ITS
Apocynaceae	*Thevetia peruviana*	LN883891	rbcL
Apocynaceae	*Thevetia peruviana*	LN883900	psbA-trnH
Apocynaceae	*Thevetia peruviana*	X91773	rbcL
Apocynaceae	*Thevetia peruviana*	Z70188	matK
Aquifoliaceae	*Ilex affinis*	EU359340	psbA-trnH
Aquifoliaceae	*Ilex brasiliensis*	AJ492575	trnL
Aquifoliaceae	*Ilex brasiliensis*	AJ492661	ITS
Aquifoliaceae	*Ilex brasiliensis*	AJ786506	ITS
Aquifoliaceae	*Ilex brasiliensis*	AY183490	ITS
Aquifoliaceae	*Ilex brasiliensis*	EU359357	psbA-trnH
Aquifoliaceae	*Ilex brasiliensis*	X98735	rbcL
Aquifoliaceae	*Ilex brevicuspis*	AJ492576	trnL
Aquifoliaceae	*Ilex brevicuspis*	AJ492662	ITS
Aquifoliaceae	*Ilex brevicuspis*	AJ492663	ITS
Aquifoliaceae	*Ilex brevicuspis*	AJ786507	ITS
Aquifoliaceae	*Ilex brevicuspis*	AY183494	ITS
Aquifoliaceae	*Ilex brevicuspis*	AY183495	ITS
Aquifoliaceae	*Ilex brevicuspis*	AY183496	ITS

**Family**	**Species**	**Accession**	**Barcode**
Aquifoliaceae	*Ilex brevicuspis*	X98719	rbcL
Aquifoliaceae	*Ilex chamaedryfolia*	FJ394594	rbcL
Aquifoliaceae	*Ilex chamaedryfolia*	FJ394665	ITS
Aquifoliaceae	*Ilex chamaedryfolia*	FJ394736	trnL
Aquifoliaceae	*Ilex dumosa*	AJ492571	trnL
Aquifoliaceae	*Ilex dumosa*	AJ492656	ITS
Aquifoliaceae	*Ilex dumosa*	AJ492657	ITS
Aquifoliaceae	*Ilex dumosa*	AY183479	ITS
Aquifoliaceae	*Ilex dumosa*	AY183480	ITS
Aquifoliaceae	*Ilex dumosa*	AY183481	ITS
Aquifoliaceae	*Ilex dumosa*	AY183488	ITS
Aquifoliaceae	*Ilex dumosa*	AY183489	ITS
Aquifoliaceae	*Ilex dumosa*	EU359316	psbA-trnH
Aquifoliaceae	*Ilex dumosa*	EU359360	psbA-trnH
Aquifoliaceae	*Ilex dumosa*	FJ394588	rbcL
Aquifoliaceae	*Ilex dumosa*	FJ394657	ITS
Aquifoliaceae	*Ilex dumosa*	FJ394730	trnL
Aquifoliaceae	*Ilex dumosa*	X98725	rbcL
Aquifoliaceae	*Ilex integerrima*	AJ492577	trnL
Aquifoliaceae	*Ilex integerrima*	AJ492664	ITS
Aquifoliaceae	*Ilex integerrima*	AJ786508	ITS
Aquifoliaceae	*Ilex integerrima*	AY183477	ITS
Aquifoliaceae	*Ilex integerrima*	AY183478	ITS
Aquifoliaceae	*Ilex microdonta*	AJ492578	trnL
Aquifoliaceae	*Ilex microdonta*	AJ492665	ITS
Aquifoliaceae	*Ilex microdonta*	AJ492702	rbcL
Aquifoliaceae	*Ilex microdonta*	AY183484	ITS
Aquifoliaceae	*Ilex microdonta*	AY183485	ITS
Aquifoliaceae	*Ilex paraguariensis*	AY183491	ITS
Aquifoliaceae	*Ilex paraguariensis*	AY183492	ITS
Aquifoliaceae	*Ilex paraguariensis*	EF590404	matK
Aquifoliaceae	*Ilex paraguariensis*	EF590537	rbcL
Aquifoliaceae	*Ilex paraguariensis*	EF590624	rpoC1
Aquifoliaceae	*Ilex paraguariensis*	EF590705	psbA-trnH
Aquifoliaceae	*Ilex paraguariensis*	EF590778	ITS

**Family**	**Species**	**Accession**	**Barcode**
Aquifoliaceae	*Ilex paraguariensis*	EU359321	psbA-trnH
Aquifoliaceae	*Ilex paraguariensis*	EU359353	psbA-trnH
Aquifoliaceae	*Ilex paraguariensis*	EU796897	ITS
Aquifoliaceae	*Ilex paraguariensis*	FJ394634	rbcL
Aquifoliaceae	*Ilex paraguariensis*	FJ394705	ITS
Aquifoliaceae	*Ilex paraguariensis*	FJ394776	trnL
Aquifoliaceae	*Ilex paraguariensis*	GQ247982	atpF-atpH
Aquifoliaceae	*Ilex paraguariensis*	GQ248141	matK
Aquifoliaceae	*Ilex paraguariensis*	GQ248322	psbA-trnH
Aquifoliaceae	*Ilex paraguariensis*	GQ248477	psbK-psbI
Aquifoliaceae	*Ilex paraguariensis*	GQ248625	rbcL
Aquifoliaceae	*Ilex paraguariensis*	GQ248796	rpoB
Aquifoliaceae	*Ilex paraguariensis*	GQ248958	rpoC1
Aquifoliaceae	*Ilex pseudobuxus*	AJ492574	trnL
Aquifoliaceae	*Ilex pseudobuxus*	AJ492660	ITS
Aquifoliaceae	*Ilex pseudobuxus*	AY183486	ITS
Aquifoliaceae	*Ilex pseudobuxus*	FJ394707	ITS
Aquifoliaceae	*Ilex pseudobuxus*	KF981196	rbcL
Aquifoliaceae	*Ilex pseudobuxus*	KF981300	matK
Aquifoliaceae	*Ilex pseudobuxus*	X98736	rbcL
Aquifoliaceae	*Ilex taubertiana*	AY183487	ITS
Aquifoliaceae	*Ilex theezans*	AJ492579	trnL
Aquifoliaceae	*Ilex theezans*	AJ492666	ITS
Aquifoliaceae	*Ilex theezans*	AJ492703	rbcL
Aquifoliaceae	*Ilex theezans*	AY183493	ITS
Aquifoliaceae	*Ilex theezans*	AY183497	ITS
Aquifoliaceae	*Ilex theezans*	AY183498	ITS
Aquifoliaceae	*Ilex theezans*	EU359352	psbA-trnH
Aquifoliaceae	*Ilex theezans*	FJ394643	rbcL
Aquifoliaceae	*Ilex theezans*	FJ394716	ITS
Aquifoliaceae	*Ilex theezans*	FJ394785	trnL
Aquifoliaceae	*Ilex theezans*	KF420998	ITS
Aquifoliaceae	*Ilex theezans*	KF421106	psbA-trnH
Aquifoliaceae	*Ilex theezans*	KF421107	psbA-trnH
Aquifoliaceae	*Ilex theezans*	KF421108	psbA-trnH

Family	Species	Accession	Barcode
Aquifoliaceae	*Ilex theezans*	KF555394	matK
Aquifoliaceae	*Ilex theezans*	KF555395	matK
Aquifoliaceae	*Ilex theezans*	KF555396	matK
Aquifoliaceae	*Ilex theezans*	KF561914	rbcL
Aquifoliaceae	*Ilex theezans*	KF561915	rbcL
Araliaceae	*Dendropanax cuneatus*	GU054674	ITS
Araliaceae	*Dendropanax cuneatus*	GU054680	ITS
Araliaceae	*Dendropanax cuneatus*	GU054864	psbA-trnH
Araliaceae	*Dendropanax cuneatus*	GU054870	psbA-trnH
Araliaceae	*Dendropanax cuneatus*	GU055149	trnL
Araliaceae	*Dendropanax cuneatus*	GU055155	trnL
Araliaceae	*Oreopanax capitatus*	JQ586673	matK
Araliaceae	*Oreopanax capitatus*	JQ586674	matK
Araliaceae	*Oreopanax capitatus*	JQ590384	rbcL
Araliaceae	*Oreopanax capitatus*	JQ590385	rbcL
Araliaceae	*Schefflera actinophylla*	AF242245	ITS
Araliaceae	*Schefflera actinophylla*	AF382153	trnL
Araliaceae	*Schefflera actinophylla*	AY393766	trnL
Araliaceae	*Schefflera actinophylla*	GU135026	matK
Araliaceae	*Schefflera actinophylla*	GU135189	rbcL
Araliaceae	*Schefflera actinophylla*	GU135355	psbA-trnH
Araliaceae	*Schefflera actinophylla*	HQ220371	trnL
Araliaceae	*Schefflera actinophylla*	HQ220594	psbA-trnH
Araliaceae	*Schefflera actinophylla*	KC952108	trnL
Araliaceae	*Schefflera actinophylla*	KC952372	ITS
Araliaceae	*Schefflera actinophylla*	KC952548	psbA-trnH
Araliaceae	*Schefflera actinophylla*	KM894791	matK
Araliaceae	*Schefflera actinophylla*	U72471	rpoC1
Araliaceae	*Schefflera angustissima*	GU004028	ITS
Araliaceae	*Schefflera angustissima*	GU004033	trnL
Araliaceae	*Schefflera calva*	GU004071	ITS
Araliaceae	*Schefflera macrocarpa*	GU004048	trnL
Araliaceae	*Schefflera macrocarpa*	GU004090	ITS
Araliaceae	*Schefflera morototoni*	AY955416	trnL
Araliaceae	*Schefflera morototoni*	AY955463	ITS

Family	Species	Accession	Barcode
Araliaceae	*Schefflera morototoni*	GU004092	ITS
Araliaceae	*Schefflera morototoni*	GU054647	ITS
Araliaceae	*Schefflera morototoni*	GU054837	psbA-trnH
Araliaceae	*Schefflera morototoni*	GU055122	trnL
Araliaceae	*Schefflera morototoni*	HM446744	matK
Araliaceae	*Schefflera morototoni*	HM446870	rbcL
Araliaceae	*Schefflera morototoni*	HM447000	psbA-trnH
Araliaceae	*Schefflera morototoni*	JQ586675	matK
Araliaceae	*Schefflera morototoni*	JQ586676	matK
Araliaceae	*Schefflera morototoni*	JQ586677	matK
Araliaceae	*Schefflera morototoni*	JQ590386	rbcL
Araliaceae	*Schefflera morototoni*	JQ625796	rbcL
Araucariaceae	*Araucaria angustifolia*	AF479866	trnL
Araucariaceae	*Araucaria angustifolia*	AM919875	rpoC1
Araucariaceae	*Araucaria angustifolia*	AM920061	rpoB
Araucariaceae	*Araucaria angustifolia*	AM920224	rbcL
Araucariaceae	*Araucaria angustifolia*	AM921999	psbA-trnH
Araucariaceae	*Araucaria angustifolia*	EF451975	matK
Araucariaceae	*Araucaria angustifolia*	EU025973	matK
Araucariaceae	*Araucaria angustifolia*	EU164985	rbcL
Araucariaceae	*Araucaria angustifolia*	EU164998	matK
Araucariaceae	*Araucaria angustifolia*	EU165012	ITS
Araucariaceae	*Araucaria angustifolia*	JN564915	ITS
Araucariaceae	*Araucaria angustifolia*	JN564916	ITS
Araucariaceae	*Araucaria angustifolia*	JN564917	ITS
Araucariaceae	*Araucaria angustifolia*	JN564918	ITS
Araucariaceae	*Araucaria angustifolia*	JN564919	ITS
Araucariaceae	*Araucaria angustifolia*	KF421014	psbA-trnH
Araucariaceae	*Araucaria angustifolia*	KF421015	psbA-trnH
Araucariaceae	*Araucaria angustifolia*	KF421016	psbA-trnH
Araucariaceae	*Araucaria angustifolia*	KF561901	rbcL
Araucariaceae	*Araucaria angustifolia*	KM459937	ITS
Araucariaceae	*Araucaria angustifolia*	KM517207	atpF-atpH
Araucariaceae	*Araucaria angustifolia*	KM517222	matK
Araucariaceae	*Araucaria angustifolia*	U87750	rbcL

**Family**	**Species**	**Accession**	**Barcode**
Araucariaceae	*Araucaria angustifolia*	U96470	rbcL
Arecaceae	*Acrocomia aculeata*	AM110212	rbcL
Arecaceae	*Acrocomia aculeata*	AM113648	trnL
Arecaceae	*Acrocomia aculeata*	AM114639	matK
Arecaceae	*Acrocomia aculeata*	AY044625	rbcL
Arecaceae	*Acrocomia aculeata*	HQ265478	ITS
Arecaceae	*Acrocomia aculeata*	HQ265759	trnL
Arecaceae	*Acrocomia aculeata*	JQ586682	matK
Arecaceae	*Acrocomia aculeata*	JQ586683	matK
Arecaceae	*Acrocomia aculeata*	JQ586684	matK
Arecaceae	*Acrocomia aculeata*	JQ586685	matK
Arecaceae	*Acrocomia aculeata*	JQ590390	rbcL
Arecaceae	*Acrocomia aculeata*	JQ590391	rbcL
Arecaceae	*Acrocomia aculeata*	JQ590392	rbcL
Arecaceae	*Acrocomia aculeata*	JQ590393	rbcL
Arecaceae	*Acrocomia aculeata*	JQ590394	rbcL
Arecaceae	*Acrocomia aculeata*	JQ590395	rbcL
Arecaceae	*Acrocomia aculeata*	JQ590396	rbcL
Arecaceae	*Acrocomia aculeata*	JQ590397	rbcL
Arecaceae	*Acrocomia aculeata*	KJ012454	matK
Arecaceae	*Acrocomia aculeata*	KJ082107	rbcL
Arecaceae	*Archontophoenix cunninghamiana*	GQ892876	ITS
Arecaceae	*Archontophoenix cunninghamiana*	HG969673	rbcL
Arecaceae	*Archontophoenix cunninghamiana*	HG969708	rpoC1
Arecaceae	*Archontophoenix cunninghamiana*	HG969771	rpoB
Arecaceae	*Archontophoenix cunninghamiana*	HG969840	psbK-psbI
Arecaceae	*Archontophoenix cunninghamiana*	HG969874	atpF-atpH
Arecaceae	*Archontophoenix cunninghamiana*	HG969938	trnL
Arecaceae	*Archontophoenix cunninghamiana*	HG969972	matK
Arecaceae	*Archontophoenix cunninghamiana*	KJ634508	ITS
Arecaceae	*Archontophoenix cunninghamiana*	KM894505	matK
Arecaceae	*Archontophoenix cunninghamiana*	KM894651	matK
Arecaceae	*Archontophoenix cunninghamiana*	KM895593	rbcL
Arecaceae	*Archontophoenix cunninghamiana*	KM895776	rbcL
Arecaceae	*Attalea oleifera*	KT321504	psbK-psbI

**Family**	**Species**	**Accession**	**Barcode**
Arecaceae	*Attalea phalerata*	HQ265548	matK
Arecaceae	*Attalea phalerata*	HQ265783	trnL
Arecaceae	*Attalea phalerata*	KC924913	psbA-trnH
Arecaceae	*Butia eriospatha*	AY044632	rbcL
Arecaceae	*Mauritia flexuosa*	AJ241281	trnL
Arecaceae	*Mauritia flexuosa*	AJ242141-AJ242145	ITS
Arecaceae	*Mauritia flexuosa*	AJ404777	rbcL
Arecaceae	*Mauritia flexuosa*	AM114545	matK
Arecaceae	*Mauritia flexuosa*	AY012473	rbcL
Arecaceae	*Mauritia flexuosa*	KC528353-KC528609	psbA-trnH
Arecaceae	*Mauritia flexuosa*	KJ500038	psbA-trnH
Arecaceae	*Syagrus romanzoffiana*	GU135086	matK
Arecaceae	*Syagrus romanzoffiana*	GU135249	rbcL
Arecaceae	*Syagrus romanzoffiana*	GU135420	psbA-trnH
Asteraceae	*Austroeupatorium inulaefolium*	AB032052	ITS
Asteraceae	*Austroeupatorium inulaefolium*	KJ637186	matK
Asteraceae	*Austroeupatorium inulaefolium*	KP454311	ITS
Asteraceae	*Austroeupatorium inulaefolium*	KP454464	ITS
Asteraceae	*Baccharis dracunculifolia*	AF046958	ITS
Asteraceae	*Dasyphyllum brasiliense*	AF412845	ITS
Asteraceae	*Dasyphyllum brasiliense*	EU547646	trnL
Asteraceae	*Dasyphyllum brasiliense*	EU841077	trnL
Asteraceae	*Dasyphyllum brasiliense*	EU841118	rbcL
Asteraceae	*Dasyphyllum brasiliense*	EU841159	ITS
Asteraceae	*Dasyphyllum brasiliense*	EU841248	rpoC1
Asteraceae	*Dasyphyllum brasiliense*	EU841291	psbA-trnH
Asteraceae	*Dasyphyllum brasiliense*	EU841341	matK
Asteraceae	*Dasyphyllum spinescens*	AF412860	ITS
Asteraceae	*Dasyphyllum spinescens*	AF412928	trnL
Asteraceae	*Dasyphyllum spinescens*	EU547648	trnL
Asteraceae	*Dasyphyllum spinescens*	EU841079	trnL
Asteraceae	*Dasyphyllum spinescens*	EU841120	rbcL
Asteraceae	*Dasyphyllum spinescens*	EU841161	ITS
Asteraceae	*Dasyphyllum spinescens*	EU841250	rpoC1
Asteraceae	*Dasyphyllum spinescens*	EU841293	psbA-trnH

**Family**	**Species**	**Accession**	**Barcode**
Asteraceae	*Dasyphyllum spinescens*	EU841343	matK
Asteraceae	*Eremanthus elaeagnus*	KM278334	ITS
Asteraceae	*Eremanthus erythropappus*	EF155770	ITS
Asteraceae	*Eremanthus erythropappus*	EF155858	trnL
Asteraceae	*Eremanthus erythropappus*	EU384972	rbcL
Asteraceae	*Eremanthus erythropappus*	EU385066	trnL
Asteraceae	*Eremanthus erythropappus*	EU385351	matK
Asteraceae	*Eremanthus erythropappus*	EU385446	rpoB
Asteraceae	*Eremanthus erythropappus*	EU385541	rpoC1
Asteraceae	*Eremanthus erythropappus*	KM053303	ITS
Asteraceae	*Eremanthus mattogrossensis*	KM278338	ITS
Asteraceae	*Gochnatia barrosoae*	KF989549	ITS
Asteraceae	*Gochnatia barrosoae*	KF989657	trnL
Asteraceae	*Gochnatia barrosoae*	KF989968	psbA-trnH
Asteraceae	*Gochnatia floribunda*	KF989559	ITS
Asteraceae	*Gochnatia floribunda*	KF989667	trnL
Asteraceae	*Gochnatia floribunda*	KF989876	matK
Asteraceae	*Gochnatia floribunda*	KF989978	psbA-trnH
Asteraceae	*Gochnatia paniculata*	KF989572	ITS
Asteraceae	*Gochnatia paniculata*	KF989573	ITS
Asteraceae	*Gochnatia paniculata*	KF989680	trnL
Asteraceae	*Gochnatia paniculata*	KF989681	trnL
Asteraceae	*Gochnatia paniculata*	KF989889	matK
Asteraceae	*Gochnatia paniculata*	KF989890	matK
Asteraceae	*Gochnatia paniculata*	KF989991	psbA-trnH
Asteraceae	*Gochnatia paniculata*	KF989992	psbA-trnH
Asteraceae	*Gochnatia polymorpha*	KF989575	ITS
Asteraceae	*Gochnatia polymorpha*	KF989576	ITS
Asteraceae	*Gochnatia polymorpha*	KF989683	trnL
Asteraceae	*Gochnatia polymorpha*	KF989684	trnL
Asteraceae	*Gochnatia polymorpha*	KF989892	matK
Asteraceae	*Gochnatia polymorpha*	KF989893	matK
Asteraceae	*Gochnatia polymorpha*	KF989994	psbA-trnH
Asteraceae	*Gochnatia polymorpha*	KF989995	psbA-trnH
Asteraceae	*Gochnatia pulchra*	KF989577	ITS

Family	Species	Accession	Barcode
Asteraceae	*Gochnatia pulchra*	KF989685	trnL
Asteraceae	*Gochnatia pulchra*	KF989894	matK
Asteraceae	*Gochnatia pulchra*	KF989996	psbA-trnH
Asteraceae	*Kaunia rufescens*	KP454375	ITS
Asteraceae	*Kaunia rufescens*	KP454525	ITS
Asteraceae	*Lychnophora ericoides*	FJ031242-FJ031400	trnL
Asteraceae	*Lychnophora ericoides*	FJ031413-FJ031454	trnL
Asteraceae	*Lychnophora ericoides*	FJ031455-FJ031630	ITS
Asteraceae	*Lychnophora ericoides*	FJ031653-FJ031668	ITS
Asteraceae	*Lychnophora ericoides*	FJ031669-FJ031882	psbA-trnH
Asteraceae	*Lychnophora ericoides*	KM053316	ITS
Asteraceae	*Piptocarpha axillaris*	L13651	rbcL
Asteraceae	*Piptocarpha oblonga*	KM053324	ITS
Asteraceae	*Piptocarpha rotundifolia*	KM053325	ITS
Asteraceae	*Raulinoreitzia leptophlebia*	KP454415	ITS
Asteraceae	*Raulinoreitzia leptophlebia*	KP454563	ITS
Asteraceae	*Stifftia chrysantha*	EU385020	rbcL
Asteraceae	*Stifftia chrysantha*	EU385114	trnL
Asteraceae	*Stifftia chrysantha*	EU385399	matK
Asteraceae	*Stifftia chrysantha*	EU385495	rpoB
Asteraceae	*Stifftia chrysantha*	EU385592	rpoC1
Asteraceae	*Stifftia chrysantha*	JF920291	psbA-trnH
Asteraceae	*Stifftia chrysantha*	JF920296	trnL
Asteraceae	*Stifftia chrysantha*	KM191977	rpoC1
Asteraceae	*Stifftia fruticosa*	JF920292	psbA-trnH
Asteraceae	*Stifftia fruticosa*	JF920297	trnL
Asteraceae	*Stifftia parviflora*	JF920293	psbA-trnH
Asteraceae	*Stifftia parviflora*	JF920298	trnL
Asteraceae	*Symphyopappus itatiayensis*	KP454432	ITS
Asteraceae	*Symphyopappus itatiayensis*	KP454580	ITS
Asteraceae	*Wunderlichia mirabilis*	DQ414741	ITS
Asteraceae	*Wunderlichia mirabilis*	DQ414742	ITS
Asteraceae	*Wunderlichia mirabilis*	DQ414743	trnL
Asteraceae	*Wunderlichia mirabilis*	DQ414744	trnL
Asteraceae	*Wunderlichia mirabilis*	EU385028	rbcL

Family	Species	Accession	Barcode
Asteraceae	*Wunderlichia mirabilis*	EU385122	trnL
Asteraceae	*Wunderlichia mirabilis*	EU385408	matK
Asteraceae	*Wunderlichia mirabilis*	EU385504	rpoB
Asteraceae	*Wunderlichia mirabilis*	EU385601	rpoC1
Asteraceae	*Wunderlichia mirabilis*	KF989521	ITS
Asteraceae	*Wunderlichia mirabilis*	KF989626	trnL
Asteraceae	*Wunderlichia mirabilis*	KF989849	matK
Asteraceae	*Wunderlichia mirabilis*	KF989937	psbA-trnH
Asteraceae	*Wunderlichia mirabilis*	KM191981	rpoC1
Bignoniaceae	*Cybistax antisyphilitica*	EF105061	trnL
Bignoniaceae	*Cybistax antisyphilitica*	JX497390	psbA-trnH
Bignoniaceae	*Cybistax antisyphilitica*	JX497391	psbA-trnH
Bignoniaceae	*Cybistax antisyphilitica*	JX497392	psbA-trnH
Bignoniaceae	*Cybistax antisyphilitica*	JX497393	psbA-trnH
Bignoniaceae	*Cybistax antisyphilitica*	JX497394	psbA-trnH
Bignoniaceae	*Cybistax antisyphilitica*	JX497395	psbA-trnH
Bignoniaceae	*Cybistax antisyphilitica*	JX497396	psbA-trnH
Bignoniaceae	*Cybistax antisyphilitica*	JX497397	psbA-trnH
Bignoniaceae	*Cybistax antisyphilitica*	JX497398	psbA-trnH
Bignoniaceae	*Cybistax antisyphilitica*	JX497399	psbA-trnH
Bignoniaceae	*Cybistax antisyphilitica*	JX497400	psbA-trnH
Bignoniaceae	*Cybistax antisyphilitica*	JX497401	psbA-trnH
Bignoniaceae	*Cybistax antisyphilitica*	JX497402	psbA-trnH
Bignoniaceae	*Cybistax antisyphilitica*	JX497403	psbA-trnH
Bignoniaceae	*Cybistax antisyphilitica*	JX497404	psbA-trnH
Bignoniaceae	*Cybistax antisyphilitica*	JX497405	psbA-trnH
Bignoniaceae	*Cybistax antisyphilitica*	JX497406	psbA-trnH
Bignoniaceae	*Cybistax antisyphilitica*	JX497699	ITS
Bignoniaceae	*Cybistax antisyphilitica*	JX497700	ITS
Bignoniaceae	*Cybistax antisyphilitica*	JX497701	ITS
Bignoniaceae	*Cybistax antisyphilitica*	JX497702	ITS
Bignoniaceae	*Cybistax antisyphilitica*	JX497703	ITS
Bignoniaceae	*Cybistax antisyphilitica*	JX497704	ITS
Bignoniaceae	*Cybistax antisyphilitica*	JX497705	ITS
Bignoniaceae	*Cybistax antisyphilitica*	JX497706	ITS

**Family**	**Species**	**Accession**	**Barcode**
Bignoniaceae	*Cybistax antisyphilitica*	JX497707	ITS
Bignoniaceae	*Cybistax antisyphilitica*	JX497708	ITS
Bignoniaceae	*Cybistax antisyphilitica*	JX497709	ITS
Bignoniaceae	*Cybistax antisyphilitica*	JX497710	ITS
Bignoniaceae	*Cybistax antisyphilitica*	JX497711	ITS
Bignoniaceae	*Cybistax antisyphilitica*	JX497712	ITS
Bignoniaceae	*Cybistax antisyphilitica*	JX497713	ITS
Bignoniaceae	*Cybistax antisyphilitica*	JX497714	ITS
Bignoniaceae	*Cybistax antisyphilitica*	JX497715	ITS
Bignoniaceae	*Handroanthus chrysotrichus*	AF034881	trnL
Bignoniaceae	*Handroanthus chrysotrichus*	EF105092	trnL
Bignoniaceae	*Handroanthus chrysotrichus*	JX497407	psbA-trnH
Bignoniaceae	*Handroanthus chrysotrichus*	JX497408	psbA-trnH
Bignoniaceae	*Handroanthus chrysotrichus*	JX497409	psbA-trnH
Bignoniaceae	*Handroanthus chrysotrichus*	JX497410	psbA-trnH
Bignoniaceae	*Handroanthus chrysotrichus*	JX497411	psbA-trnH
Bignoniaceae	*Handroanthus chrysotrichus*	JX497412	psbA-trnH
Bignoniaceae	*Handroanthus chrysotrichus*	JX497413	psbA-trnH
Bignoniaceae	*Handroanthus chrysotrichus*	JX497414	psbA-trnH
Bignoniaceae	*Handroanthus chrysotrichus*	JX497415	psbA-trnH
Bignoniaceae	*Handroanthus chrysotrichus*	JX497416	psbA-trnH
Bignoniaceae	*Handroanthus chrysotrichus*	JX497417	psbA-trnH
Bignoniaceae	*Handroanthus chrysotrichus*	JX497418	psbA-trnH
Bignoniaceae	*Handroanthus chrysotrichus*	JX497419	psbA-trnH
Bignoniaceae	*Handroanthus chrysotrichus*	JX497420	psbA-trnH
Bignoniaceae	*Handroanthus chrysotrichus*	JX497716	ITS
Bignoniaceae	*Handroanthus chrysotrichus*	JX497717	ITS
Bignoniaceae	*Handroanthus chrysotrichus*	JX497718	ITS
Bignoniaceae	*Handroanthus chrysotrichus*	JX497719	ITS
Bignoniaceae	*Handroanthus chrysotrichus*	JX497720	ITS
Bignoniaceae	*Handroanthus chrysotrichus*	JX497721	ITS
Bignoniaceae	*Handroanthus chrysotrichus*	JX497722	ITS
Bignoniaceae	*Handroanthus chrysotrichus*	JX497723	ITS
Bignoniaceae	*Handroanthus chrysotrichus*	JX497724	ITS
Bignoniaceae	*Handroanthus chrysotrichus*	JX497725	ITS

Family	Species	Accession	Barcode
Bignoniaceae	*Handroanthus chrysotrichus*	JX497726	ITS
Bignoniaceae	*Handroanthus chrysotrichus*	JX497727	ITS
Bignoniaceae	*Handroanthus chrysotrichus*	JX497728	ITS
Bignoniaceae	*Handroanthus chrysotrichus*	JX497729	ITS
Bignoniaceae	*Handroanthus chrysotrichus*	JX856516	ITS
Bignoniaceae	*Handroanthus chrysotrichus*	JX856786	rbcL
Bignoniaceae	*Handroanthus chrysotrichus*	JX856787	rbcL
Bignoniaceae	*Handroanthus impetiginosus*	EF105097	trnL
Bignoniaceae	*Handroanthus impetiginosus*	JQ587040-JQ587045	matK
Bignoniaceae	*Handroanthus impetiginosus*	JQ590845-JQ590850	rbcL
Bignoniaceae	*Handroanthus impetiginosus*	JX497102-JX497389	psbA-trnH
Bignoniaceae	*Handroanthus impetiginosus*	JX497421-JX497698	ITS
Bignoniaceae	*Handroanthus impetiginosus*	JX856460	ITS
Bignoniaceae	*Handroanthus impetiginosus*	JX856961	psbA-trnH
Bignoniaceae	*Handroanthus impetiginosus*	KM219811	matK
Bignoniaceae	*Handroanthus ochraceus*	EF105100	trnL
Bignoniaceae	*Handroanthus ochraceus*	HG963652	psbA-trnH
Bignoniaceae	*Handroanthus ochraceus*	JQ587046	matK
Bignoniaceae	*Handroanthus ochraceus*	JQ587047	matK
Bignoniaceae	*Handroanthus ochraceus*	JQ587048	matK
Bignoniaceae	*Handroanthus ochraceus*	JQ587049	matK
Bignoniaceae	*Handroanthus ochraceus*	JQ587050	matK
Bignoniaceae	*Handroanthus ochraceus*	JQ587051	matK
Bignoniaceae	*Handroanthus ochraceus*	JQ590851	rbcL
Bignoniaceae	*Handroanthus ochraceus*	JQ590852	rbcL
Bignoniaceae	*Handroanthus ochraceus*	JQ590853	rbcL
Bignoniaceae	*Handroanthus ochraceus*	JQ590854	rbcL
Bignoniaceae	*Handroanthus ochraceus*	JQ590855	rbcL
Bignoniaceae	*Handroanthus ochraceus*	JQ590856	rbcL
Bignoniaceae	*Handroanthus serratifolius*	EF105105	trnL
Bignoniaceae	*Handroanthus serratifolius*	JQ626306	rbcL
Bignoniaceae	*Jacaranda cuspidifolia*	JX856717	rbcL
Bignoniaceae	*Jacaranda cuspidifolia*	JX856900	psbA-trnH
Bignoniaceae	*Jacaranda puberula*	KF420996	ITS
Bignoniaceae	*Jacaranda puberula*	KF420997	ITS

**Family**	**Species**	**Accession**	**Barcode**
Bignoniaceae	*Jacaranda puberula*	KF421097	psbA-trnH
Bignoniaceae	*Jacaranda puberula*	KF421098	psbA-trnH
Bignoniaceae	*Jacaranda puberula*	KF421099	psbA-trnH
Bignoniaceae	*Jacaranda puberula*	KF555398	matK
Bignoniaceae	*Jacaranda puberula*	KF555400	matK
Bignoniaceae	*Jacaranda puberula*	KF561917	rbcL
Bignoniaceae	*Jacaranda puberula*	KF561918	rbcL
Bignoniaceae	*Jacaranda puberula*	KF561919	rbcL
Bignoniaceae	*Sparattosperma leucanthum*	EF105082	trnL
Bignoniaceae	*Spathodea campanulata*	AY500408	trnL
Bignoniaceae	*Spathodea campanulata*	AY500428	trnL
Bignoniaceae	*Spathodea campanulata*	AY702555	ITS
Bignoniaceae	*Spathodea campanulata*	AY702562	rbcL
Bignoniaceae	*Spathodea campanulata*	EF105083	trnL
Bignoniaceae	*Spathodea campanulata*	FJ976171	rbcL
Bignoniaceae	*Spathodea campanulata*	HM446746	matK
Bignoniaceae	*Spathodea campanulata*	HM446873	rbcL
Bignoniaceae	*Spathodea campanulata*	HM447003	psbA-trnH
Bignoniaceae	*Spathodea campanulata*	JX495760	matK
Bignoniaceae	*Spathodea campanulata*	JX571895	rbcL
Bignoniaceae	*Tabebuia aurea*	EF105087	trnL
Bignoniaceae	*Tabebuia aurea*	JX856512-JX856515	ITS
Bignoniaceae	*Tabebuia aurea*	JX856784	rbcL
Bignoniaceae	*Tabebuia aurea*	JX856785	rbcL
Bignoniaceae	*Tabebuia aurea*	JX856958	psbA-trnH
Bignoniaceae	*Tabebuia aurea*	JX856959	psbA-trnH
Bignoniaceae	*Tabebuia aurea*	JX856960	psbA-trnH
Bignoniaceae	*Tabebuia aurea*	KF432023	rbcL
Bignoniaceae	*Tabebuia aurea*	KM054010-KM054214	ITS
Bignoniaceae	*Tabebuia aurea*	KM054215-KM054512	psbA-trnH
Bignoniaceae	*Tabebuia insignis*	EF105108	trnL
Bignoniaceae	*Tecoma stans*	AF034888	trnL
Bignoniaceae	*Tecoma stans*	AF102655	rbcL
Bignoniaceae	*Tecoma stans*	AY008826	trnL
Bignoniaceae	*Tecoma stans*	AY178636	ITS

Family	Species	Accession	Barcode
Bignoniaceae	*Tecoma stans*	FJ870059	trnL
Bignoniaceae	*Tecoma stans*	HG963507	psbA-trnH
Bignoniaceae	*Tecoma stans*	HQ384522	matK
Bignoniaceae	*Tecoma stans*	JX495765	matK
Bignoniaceae	*Tecoma stans*	JX517475	matK
Bignoniaceae	*Tecoma stans*	JX571902	rbcL
Bignoniaceae	*Tecoma stans*	JX573034	rbcL
Bignoniaceae	*Zeyheria montana*	EF105114	trnL
Bignoniaceae	*Zeyheria tuberculosa*	EF105115	trnL
Bixaceae	*Bixa orellana*	AF022128	rbcL
Bixaceae	*Bixa orellana*	FM179540	trnL
Bixaceae	*Bixa orellana*	FM179929	matK
Bixaceae	*Bixa orellana*	HG963562	psbA-trnH
Bixaceae	*Bixa orellana*	JQ587060	matK
Bixaceae	*Bixa orellana*	JQ587061	matK
Bixaceae	*Bixa orellana*	JQ587062	matK
Bixaceae	*Bixa orellana*	JQ590867	rbcL
Bixaceae	*Bixa orellana*	JQ590868	rbcL
Bixaceae	*Bixa orellana*	JQ590869	rbcL
Bixaceae	*Bixa orellana*	JQ590870	rbcL
Bixaceae	*Bixa orellana*	JQ590871	rbcL
Bixaceae	*Bixa orellana*	KF055235	ITS
Bixaceae	*Bixa orellana*	Y15139	rbcL
Boraginaceae	*Cordia americana*	AY176080	ITS
Boraginaceae	*Cordia americana*	KF158133	rbcL
Boraginaceae	*Cordia americana*	KF158212	trnL
Boraginaceae	*Cordia ecalyculata*	AY321600	ITS
Boraginaceae	*Cordia ecalyculata*	JF332057	ITS
Boraginaceae	*Cordia ecalyculata*	JF427918	psbA-trnH
Boraginaceae	*Cordia glabrata*	AY701578	ITS
Boraginaceae	*Cordia glabrata*	JF332097	ITS
Boraginaceae	*Cordia glabrata*	JF427958	psbA-trnH
Boraginaceae	*Cordia sellowiana*	JF332069	ITS
Boraginaceae	*Cordia sellowiana*	JF427930	psbA-trnH
Boraginaceae	*Cordia superba*	AY321615	ITS

Family	Species	Accession	Barcode
Boraginaceae	*Cordia superba*	JF332085	ITS
Boraginaceae	*Cordia superba*	JF427946	psbA-trnH
Boraginaceae	*Cordia taguahyensis*	AY321616	ITS
Boraginaceae	*Cordia taguahyensis*	JF332087	ITS
Boraginaceae	*Cordia taguahyensis*	JF427948	psbA-trnH
Boraginaceae	*Cordia trichoclada*	JF332077	ITS
Boraginaceae	*Cordia trichoclada*	JF427938	psbA-trnH
Boraginaceae	*Cordia trichotoma*	AF402580	ITS
Boraginaceae	*Cordia trichotoma*	AY701596	ITS
Boraginaceae	*Cordia trichotoma*	EU599651	matK
Boraginaceae	*Cordia trichotoma*	EU599827	rbcL
Boraginaceae	*Cordia trichotoma*	EU599915	trnL
Boraginaceae	*Cordia trichotoma*	EU600003	trnL
Boraginaceae	*Cordia trichotoma*	JF332099	ITS
Boraginaceae	*Cordia trichotoma*	JF427960	psbA-trnH
Boraginaceae	*Tournefortia bicolor*	JQ587127	matK
Boraginaceae	*Tournefortia bicolor*	JQ587128	matK
Boraginaceae	*Tournefortia bicolor*	JQ590929	rbcL
Boraginaceae	*Tournefortia bicolor*	JQ590930	rbcL
Burseraceae	*Protium brasiliense*	KJ503502	ITS
Burseraceae	*Protium brasiliense*	KJ503781	trnL
Burseraceae	*Protium heptaphyllum*	AY375508	ITS
Burseraceae	*Protium heptaphyllum*	KJ503445	ITS
Burseraceae	*Protium heptaphyllum*	KJ503446	ITS
Burseraceae	*Protium heptaphyllum*	KJ503492	ITS
Burseraceae	*Protium heptaphyllum*	KJ503500	ITS
Burseraceae	*Protium heptaphyllum*	KJ503723	trnL
Burseraceae	*Protium heptaphyllum*	KJ503724	trnL
Burseraceae	*Protium heptaphyllum*	KJ503771	trnL
Burseraceae	*Protium heptaphyllum*	KJ503779	trnL
Burseraceae	*Protium kleinii*	KJ503505	ITS
Burseraceae	*Protium kleinii*	KJ503784	trnL
Burseraceae	*Protium ovatum*	KJ503506	ITS
Burseraceae	*Protium ovatum*	KJ503785	trnL
Burseraceae	*Protium spruceanum*	KJ503451	ITS

Family	Species	Accession	Barcode
Burseraceae	*Protium spruceanum*	KJ503729	trnL
Burseraceae	*Protium warmingianum*	KJ503503	ITS
Burseraceae	*Protium warmingianum*	KJ503782	trnL
Burseraceae	*Protium widgrenii*	KJ503512	ITS
Burseraceae	*Protium widgrenii*	KJ503791	trnL
Cactaceae	*Brasiliopuntia brasiliensis*	AY875234	rbcL
Cactaceae	*Brasiliopuntia brasiliensis*	AY875343	psbA-trnH
Cactaceae	*Brasiliopuntia brasiliensis*	AY875370	matK
Cactaceae	*Brasiliopuntia brasiliensis*	FN997068	matK
Cactaceae	*Brasiliopuntia brasiliensis*	HQ872512	ITS
Cactaceae	*Brasiliopuntia brasiliensis*	JF712685	trnL
Cactaceae	*Brasiliopuntia brasiliensis*	JF786712	matK
Cactaceae	*Brasiliopuntia brasiliensis*	JF786876	ITS
Cactaceae	*Brasiliopuntia brasiliensis*	JN387143	ycf1
Cactaceae	*Cereus fernambucensis*	AY875240	rbcL
Cactaceae	*Cereus fernambucensis*	AY875328	psbA-trnH
Cactaceae	*Cereus hildmannianus*	AM502522	trnL
Cactaceae	*Cereus hildmannianus*	HM041240	trnL
Cactaceae	*Cereus hildmannianus*	HM041660	matK
Cactaceae	*Coleocephalocereus fluminensis*	AY015318	matK
Cactaceae	*Coleocephalocereus fluminensis*	AY015364	trnL
Cactaceae	*Coleocephalocereus fluminensis*	AY015405	trnL
Cactaceae	*Coleocephalocereus fluminensis*	AY064347	ITS
Cactaceae	*Opuntia monacantha*	JF712782	trnL
Cactaceae	*Opuntia monacantha*	JF786810	matK
Cactaceae	*Opuntia monacantha*	JF786967	ITS
Cactaceae	*Opuntia monacantha*	KJ914596	trnL
Cactaceae	*Pereskia aculeata*	AF206805	rbcL
Cactaceae	*Pereskia aculeata*	AF432938	trnL
Cactaceae	*Pereskia aculeata*	AJ583236	trnL
Cactaceae	*Pereskia aculeata*	AY042626	matK
Cactaceae	*Pereskia aculeata*	AY851563	psbA-trnH
Cactaceae	*Pereskia aculeata*	AY875229	rbcL
Cactaceae	*Pereskia aculeata*	AY875323	psbA-trnH
Cactaceae	*Pereskia aculeata*	AY875355	matK

Family	Species	Accession	Barcode
Cactaceae	*Pereskia aculeata*	DQ855863	matK
Cactaceae	*Pereskia aculeata*	EU926671	trnL
Cactaceae	*Pereskia aculeata*	EU926672	trnL
Cactaceae	*Pereskia aculeata*	EU926673	trnL
Cactaceae	*Pereskia aculeata*	EU926674	trnL
Cactaceae	*Pereskia aculeata*	EU926675	trnL
Cactaceae	*Pereskia aculeata*	EU926676	trnL
Cactaceae	*Pereskia aculeata*	EU926677	trnL
Cactaceae	*Pereskia aculeata*	EU926678	trnL
Cactaceae	*Pereskia aculeata*	EU926679	trnL
Cactaceae	*Pereskia aculeata*	EU926680	trnL
Cactaceae	*Pereskia aculeata*	EU926681	trnL
Cactaceae	*Pereskia aculeata*	EU926682	trnL
Cactaceae	*Pereskia aculeata*	EU926683	trnL
Cactaceae	*Pereskia aculeata*	EU926684	trnL
Cactaceae	*Pereskia aculeata*	EU926685	trnL
Cactaceae	*Pereskia aculeata*	EU926686	trnL
Cactaceae	*Pereskia aculeata*	EU926687	trnL
Cactaceae	*Pereskia aculeata*	EU926688	trnL
Cactaceae	*Pereskia aculeata*	EU926689	trnL
Cactaceae	*Pereskia aculeata*	EU926690	trnL
Cactaceae	*Pereskia aculeata*	EU926691	trnL
Cactaceae	*Pereskia aculeata*	EU926692	trnL
Cactaceae	*Pereskia aculeata*	EU926693	trnL
Cactaceae	*Pereskia aculeata*	EU926694	trnL
Cactaceae	*Pereskia aculeata*	EU926695	trnL
Cactaceae	*Pereskia aculeata*	EU926696	trnL
Cactaceae	*Pereskia aculeata*	EU926697	trnL
Cactaceae	*Pereskia aculeata*	EU926698	trnL
Cactaceae	*Pereskia aculeata*	EU926699	trnL
Cactaceae	*Pereskia aculeata*	EU926700	trnL
Cactaceae	*Pereskia aculeata*	EU926701	trnL
Cactaceae	*Pereskia aculeata*	EU926702	trnL
Cactaceae	*Pereskia aculeata*	EU926703	trnL
Cactaceae	*Pereskia aculeata*	EU926704	trnL

**Family**	**Species**	**Accession**	**Barcode**
Cactaceae	*Pereskia aculeata*	FN997387	matK
Cactaceae	*Pereskia aculeata*	HM041338	trnL
Cactaceae	*Pereskia aculeata*	HM041757	matK
Cactaceae	*Pereskia aculeata*	HQ872561	ITS
Cactaceae	*Pereskia aculeata*	JF508526	ITS
Cactaceae	*Pereskia aculeata*	JX905944	matK
Cactaceae	*Pereskia aculeata*	JX905965	rbcL
Cactaceae	*Pereskia aculeata*	L78035	ITS
Cactaceae	*Pereskia aculeata*	LN871112	rbcL
Cactaceae	*Pereskia aculeata*	LN871159	psbA-trnH
Cactaceae	*Pereskia aculeata*	M97888	rbcL
Cactaceae	*Pereskia grandifolia*	AY851577	psbA-trnH
Cactaceae	*Pereskia grandifolia*	AY851578	psbA-trnH
Cactaceae	*Pereskia grandifolia*	AY875228	rbcL
Cactaceae	*Pereskia grandifolia*	AY875325	psbA-trnH
Cactaceae	*Pereskia grandifolia*	AY875362	matK
Cactaceae	*Pereskia grandifolia*	HQ901364	ITS
Cactaceae	*Pereskia grandifolia*	HQ901365	trnL
Cactaceae	*Pereskia grandifolia*	KM261954	trnL
Cactaceae	*Pereskia grandifolia*	KM262028	trnL
Cactaceae	*Pilosocereus machrisii*	JN035466-JN035480	psbA-trnH
Cactaceae	*Pilosocereus machrisii*	JN035491-JN035503	psbA-trnH
Cactaceae	*Pilosocereus machrisii*	JN035515-JN035523	trnL
Cactaceae	*Pilosocereus machrisii*	JN035527-JN035532	trnL
Cactaceae	*Pilosocereus machrisii*	JN035539-JN035548	trnL
Cactaceae	*Pilosocereus machrisii*	JN035554-JN035560	trnL
Cactaceae	*Pilosocereus machrisii*	JN035570-JN035580	trnL
Cactaceae	*Pilosocereus machrisii*	JN035590-JN035604	trnL
Cactaceae	*Pilosocereus machrisii*	KC621138-KC621152	trnL
Cactaceae	*Pilosocereus machrisii*	KC621159-KC621163	trnL
Cactaceae	*Pilosocereus machrisii*	KC621189-KC621193	trnL
Cactaceae	*Pilosocereus machrisii*	KC621199-KC621208	trnL
Cactaceae	*Pilosocereus machrisii*	KC621213-KC621217	trnL
Cactaceae	*Pilosocereus machrisii*	KC621238-KC621245	trnL
Calophyllaceae	*Calophyllum brasiliense*	AY625643	ITS

**Family**	**Species**	**Accession**	**Barcode**
Calophyllaceae	*Calophyllum brasiliense*	HQ331550	matK
Calophyllaceae	*Calophyllum brasiliense*	JQ219659	trnL
Calophyllaceae	*Calophyllum brasiliense*	JQ591092	rbcL
Calophyllaceae	*Calophyllum brasiliense*	JQ591093	rbcL
Calophyllaceae	*Calophyllum brasiliense*	JQ591094	rbcL
Calophyllaceae	*Calophyllum brasiliense*	KC174558	ITS
Calophyllaceae	*Calophyllum brasiliense*	KC484699	ITS
Calophyllaceae	*Calophyllum brasiliense*	KC493365	ITS
Calophyllaceae	*Calophyllum brasiliense*	KC493366	rbcL
Calophyllaceae	*Calophyllum brasiliense*	KC493367	rbcL
Calophyllaceae	*Calophyllum brasiliense*	KC493368	rbcL
Calophyllaceae	*Calophyllum brasiliense*	KC570910	ITS
Calophyllaceae	*Calophyllum brasiliense*	KC570911	rbcL
Calophyllaceae	*Calophyllum brasiliense*	KF854307	trnL
Calophyllaceae	*Calophyllum brasiliense*	KF854308	trnL
Calophyllaceae	*Calophyllum brasiliense*	KF854309	trnL
Calophyllaceae	*Calophyllum brasiliense*	KJ155012	psbA-trnH
Calophyllaceae	*Calophyllum brasiliense*	KJ155013	psbA-trnH
Calophyllaceae	*Calophyllum brasiliense*	KR057764	psbA-trnH
Calophyllaceae	*Calophyllum brasiliense*	KR057765	psbA-trnH
Calophyllaceae	*Calophyllum brasiliense*	KR057766	psbA-trnH
Calophyllaceae	*Calophyllum brasiliense*	KR057767	psbA-trnH
Calophyllaceae	*Calophyllum brasiliense*	KR057768	psbA-trnH
Calophyllaceae	*Calophyllum brasiliense*	KR057769	psbA-trnH
Calophyllaceae	*Calophyllum brasiliense*	KR057770	psbA-trnH
Calophyllaceae	*Calophyllum brasiliense*	KR057771	psbA-trnH
Calophyllaceae	*Calophyllum brasiliense*	KR270511	matK
Calophyllaceae	*Kielmeyera lathrophyton*	AF518400	rbcL
Calophyllaceae	*Kielmeyera lathrophyton*	AY625015	rbcL
Calophyllaceae	*Kielmeyera lathrophyton*	AY625623	ITS
Calophyllaceae	*Kielmeyera lathrophyton*	HQ331641	matK
Calophyllaceae	*Kielmeyera rosea*	AY625014	rbcL
Calophyllaceae	*Kielmeyera rosea*	AY625622	ITS
Canellaceae	*Cinnamodendron dinisii*	AY004132	ITS
Canellaceae	*Cinnamodendron dinisii*	AY004149	trnL

**Family**	**Species**	**Accession**	**Barcode**
Canellaceae	*Cinnamodendron dinisii*	EU669477	matK
Canellaceae	*Cinnamodendron dinisii*	EU669489	matK
Canellaceae	*Cinnamodendron dinisii*	EU669491	ITS
Canellaceae	*Cinnamodendron dinisii*	EU669492	ITS
Canellaceae	*Cinnamodendron dinisii*	EU669509	rbcL
Canellaceae	*Cinnamodendron dinisii*	EU669546	trnL
Canellaceae	*Cinnamodendron dinisii*	EU669547	trnL
Canellaceae	*Cinnamodendron dinisii*	FJ539215	psbA-trnH
Canellaceae	*Cinnamodendron dinisii*	KP407470	matK
Canellaceae	*Cinnamodendron dinisii*	KP407471	matK
Canellaceae	*Cinnamodendron dinisii*	KP407472	matK
Canellaceae	*Cinnamodendron occhionianum*	KP407474	matK
Cannabaceae	*Celtis ehrenbergiana*	JN040365	trnL
Cannabaceae	*Celtis ehrenbergiana*	JN040406	rbcL
Cannabaceae	*Celtis iguanaea*	AY488672	trnL
Cannabaceae	*Celtis iguanaea*	AY488673	trnL
Cannabaceae	*Celtis iguanaea*	AY488719	ITS
Cannabaceae	*Celtis iguanaea*	HG963813	psbA-trnH
Cannabaceae	*Celtis iguanaea*	JQ589360	matK
Cannabaceae	*Celtis iguanaea*	JQ589361	matK
Cannabaceae	*Celtis iguanaea*	JQ589362	matK
Cannabaceae	*Celtis iguanaea*	JQ589363	matK
Cannabaceae	*Celtis iguanaea*	JQ589364	matK
Cannabaceae	*Celtis iguanaea*	JQ589365	matK
Cannabaceae	*Celtis iguanaea*	JQ589366	matK
Cannabaceae	*Celtis iguanaea*	JQ589367	matK
Cannabaceae	*Celtis iguanaea*	JQ589979	matK
Cannabaceae	*Celtis iguanaea*	JQ594286	rbcL
Cannabaceae	*Celtis iguanaea*	JQ594287	rbcL
Cannabaceae	*Celtis iguanaea*	JQ594288	rbcL
Cannabaceae	*Celtis iguanaea*	JQ594289	rbcL
Cannabaceae	*Celtis iguanaea*	JQ594290	rbcL
Cannabaceae	*Celtis iguanaea*	JQ594291	rbcL
Cannabaceae	*Celtis iguanaea*	JQ594292	rbcL
Cannabaceae	*Celtis iguanaea*	JQ594293	rbcL

**Family**	**Species**	**Accession**	**Barcode**
Cannabaceae	*Celtis iguanaea*	JQ594966	rbcL
Cannabaceae	*Celtis iguanaea*	JQ594967	rbcL
Cannabaceae	*Celtis iguanaea*	KJ593797	matK
Cannabaceae	*Celtis iguanaea*	KJ593798	matK
Cannabaceae	*Celtis iguanaea*	KJ594143	rbcL
Cannabaceae	*Celtis iguanaea*	KJ594144	rbcL
Cannabaceae	*Celtis iguanaea*	KJ594145	rbcL
Cannabaceae	*Trema micrantha*	AY488699	trnL
Cannabaceae	*Trema micrantha*	AY488700	trnL
Cannabaceae	*Trema micrantha*	AY488701	trnL
Cannabaceae	*Trema micrantha*	AY488702	trnL
Cannabaceae	*Trema micrantha*	AY488703	trnL
Cannabaceae	*Trema micrantha*	AY488704	trnL
Cannabaceae	*Trema micrantha*	AY488705	trnL
Cannabaceae	*Trema micrantha*	AY488706	trnL
Cannabaceae	*Trema micrantha*	AY488707	trnL
Cannabaceae	*Trema micrantha*	AY488708	trnL
Cannabaceae	*Trema micrantha*	AY488709	trnL
Cannabaceae	*Trema micrantha*	AY488710	trnL
Cannabaceae	*Trema micrantha*	AY488711	trnL
Cannabaceae	*Trema micrantha*	AY488712	trnL
Cannabaceae	*Trema micrantha*	AY488713	trnL
Cannabaceae	*Trema micrantha*	AY488714	trnL
Cannabaceae	*Trema micrantha*	AY488715	trnL
Cannabaceae	*Trema micrantha*	AY488736	ITS
Cannabaceae	*Trema micrantha*	AY488737	ITS
Cannabaceae	*Trema micrantha*	AY488738	ITS
Cannabaceae	*Trema micrantha*	AY488739	ITS
Cannabaceae	*Trema micrantha*	AY488740	ITS
Cannabaceae	*Trema micrantha*	AY488741	ITS
Cannabaceae	*Trema micrantha*	AY488742	ITS
Cannabaceae	*Trema micrantha*	AY488743	ITS
Cannabaceae	*Trema micrantha*	AY488744	ITS
Cannabaceae	*Trema micrantha*	AY488745	ITS
Cannabaceae	*Trema micrantha*	AY488746	ITS

Family	Species	Accession	Barcode
Cannabaceae	*Trema micrantha*	AY488747	ITS
Cannabaceae	*Trema micrantha*	AY488748	ITS
Cannabaceae	*Trema micrantha*	AY488749	ITS
Cannabaceae	*Trema micrantha*	AY488750	ITS
Cannabaceae	*Trema micrantha*	AY488751	ITS
Cannabaceae	*Trema micrantha*	AY488752	ITS
Cannabaceae	*Trema micrantha*	AY488753	ITS
Cannabaceae	*Trema micrantha*	AY488754	ITS
Cannabaceae	*Trema micrantha*	AY635566	ITS
Cannabaceae	*Trema micrantha*	AY635567	ITS
Cannabaceae	*Trema micrantha*	AY635568	ITS
Cannabaceae	*Trema micrantha*	AY635569	ITS
Cannabaceae	*Trema micrantha*	AY635570	ITS
Cannabaceae	*Trema micrantha*	AY635571	ITS
Cannabaceae	*Trema micrantha*	GQ981902	rbcL
Cannabaceae	*Trema micrantha*	GQ982115	matK
Cannabaceae	*Trema micrantha*	GQ982390	psbA-trnH
Cannabaceae	*Trema micrantha*	HG963656	psbA-trnH
Cannabaceae	*Trema micrantha*	JN040393	trnL
Cannabaceae	*Trema micrantha*	JN040430	rbcL
Cannabaceae	*Trema micrantha*	JQ589368	matK
Cannabaceae	*Trema micrantha*	JQ589369	matK
Cannabaceae	*Trema micrantha*	JQ589370	matK
Cannabaceae	*Trema micrantha*	JQ589371	matK
Cannabaceae	*Trema micrantha*	JQ589372	matK
Cannabaceae	*Trema micrantha*	JQ589373	matK
Cannabaceae	*Trema micrantha*	JQ589374	matK
Cannabaceae	*Trema micrantha*	JQ594294	rbcL
Cannabaceae	*Trema micrantha*	JQ594295	rbcL
Cannabaceae	*Trema micrantha*	JQ594296	rbcL
Cannabaceae	*Trema micrantha*	JQ594297	rbcL
Cannabaceae	*Trema micrantha*	JQ594298	rbcL
Cannabaceae	*Trema micrantha*	JQ594299	rbcL
Cannabaceae	*Trema micrantha*	JQ594300	rbcL
Cannabaceae	*Trema micrantha*	JX987559	ITS

Family	Species	Accession	Barcode
Cannabaceae	*Trema micrantha*	JX987560	ITS
Cannabaceae	*Trema micrantha*	JX987561	ITS
Cannabaceae	*Trema micrantha*	JX987562	ITS
Cannabaceae	*Trema micrantha*	JX987563	ITS
Cannabaceae	*Trema micrantha*	JX987564	ITS
Cannabaceae	*Trema micrantha*	JX987598	rbcL
Cannabaceae	*Trema micrantha*	JX987599	rbcL
Cannabaceae	*Trema micrantha*	JX987600	rbcL
Cannabaceae	*Trema micrantha*	JX987601	rbcL
Cannabaceae	*Trema micrantha*	JX997357	psbA-trnH
Cannabaceae	*Trema micrantha*	JX997358	psbA-trnH
Cannabaceae	*Trema micrantha*	JX997359	psbA-trnH
Cannabaceae	*Trema micrantha*	JX997360	psbA-trnH
Cannabaceae	*Trema micrantha*	KJ012809	matK
Cannabaceae	*Trema micrantha*	KJ082620	rbcL
Cannabaceae	*Trema micrantha*	U03844	rbcL
Capparaceae	*Capparidastrum frondosum*	AY122419	trnL
Capparaceae	*Capparidastrum frondosum*	GQ981684	rbcL
Capparaceae	*Capparidastrum frondosum*	GQ981949	matK
Capparaceae	*Capparidastrum frondosum*	GQ982165	psbA-trnH
Capparaceae	*Capparidastrum frondosum*	JQ587193	matK
Capparaceae	*Capparidastrum frondosum*	JQ587194	matK
Capparaceae	*Capparidastrum frondosum*	JQ587195	matK
Capparaceae	*Capparidastrum frondosum*	JQ587211	matK
Capparaceae	*Capparidastrum frondosum*	JQ587212	matK
Capparaceae	*Capparidastrum frondosum*	JQ587213	matK
Capparaceae	*Capparidastrum frondosum*	JQ591015	rbcL
Capparaceae	*Capparidastrum frondosum*	JQ591016	rbcL
Capparaceae	*Capparidastrum frondosum*	JQ591017	rbcL
Capparaceae	*Capparidastrum frondosum*	JQ591033	rbcL
Capparaceae	*Capparidastrum frondosum*	JQ591034	rbcL
Capparaceae	*Capparidastrum frondosum*	JQ591035	rbcL
Capparaceae	*Capparidastrum frondosum*	JQ591036	rbcL
Capparaceae	*Capparidastrum frondosum*	KJ012501	matK
Capparaceae	*Capparidastrum frondosum*	KJ082172	rbcL

Family	Species	Accession	Barcode
Capparaceae	*Capparidastrum frondosum*	KJ426646	psbA-trnH
Capparaceae	*Crateva tapia*	AJ876565	psbA-trnH
Capparaceae	*Crateva tapia*	AY122429	trnL
Capparaceae	*Crateva tapia*	DQ455783	ITS
Capparaceae	*Crateva tapia*	DQ455784	ITS
Capparaceae	*Crateva tapia*	EU371781	matK
Capparaceae	*Crateva tapia*	HG963730	psbA-trnH
Capparaceae	*Cynophalla flexuosa*	AY122418	trnL
Capparaceae	*Cynophalla flexuosa*	EU371760	matK
Capparaceae	*Cynophalla flexuosa*	KJ012556	matK
Capparaceae	*Cynophalla flexuosa*	KJ082258	rbcL
Capparaceae	*Cynophalla flexuosa*	KJ426691	psbA-trnH
Caricaceae	*Carica papaya*	KC984643	ITS
Caricaceae	*Jacaratia heptaphylla*	JX091833	trnL
Caricaceae	*Jacaratia heptaphylla*	JX092011	matK
Caricaceae	*Jacaratia heptaphylla*	JX092059	ITS
Caricaceae	*Jacaratia spinosa*	JX091835	trnL
Caricaceae	*Jacaratia spinosa*	JX091836	trnL
Caricaceae	*Jacaratia spinosa*	JX091924	rbcL
Caricaceae	*Jacaratia spinosa*	JX092014	matK
Caricaceae	*Jacaratia spinosa*	JX092015	matK
Caricaceae	*Jacaratia spinosa*	JX092061	ITS
Caricaceae	*Jacaratia spinosa*	JX092062	ITS
Caricaceae	*Vasconcellea monoica*	AY461537	ITS
Caricaceae	*Vasconcellea monoica*	AY461564	matK
Caricaceae	*Vasconcellea monoica*	AY847032	psbA-trnH
Caricaceae	*Vasconcellea monoica*	DQ061119	trnL
Caricaceae	*Vasconcellea monoica*	JX091859	trnL
Caricaceae	*Vasconcellea monoica*	JX091860	trnL
Caricaceae	*Vasconcellea monoica*	JX091949	rbcL
Caricaceae	*Vasconcellea monoica*	JX091950	rbcL
Caricaceae	*Vasconcellea monoica*	JX092038	matK
Caricaceae	*Vasconcellea monoica*	JX092039	matK
Caricaceae	*Vasconcellea monoica*	JX092081	ITS
Caricaceae	*Vasconcellea quercifolia*	AY461530	ITS

**Family**	**Species**	**Accession**	**Barcode**
Caricaceae	*Vasconcellea quercifolia*	AY461558	matK
Caricaceae	*Vasconcellea quercifolia*	AY847041	psbA-trnH
Caricaceae	*Vasconcellea quercifolia*	DQ061136	trnL
Caricaceae	*Vasconcellea quercifolia*	JX091867	trnL
Caricaceae	*Vasconcellea quercifolia*	JX091868	trnL
Caricaceae	*Vasconcellea quercifolia*	JX091869	trnL
Caricaceae	*Vasconcellea quercifolia*	JX091957	rbcL
Caricaceae	*Vasconcellea quercifolia*	JX091958	rbcL
Caricaceae	*Vasconcellea quercifolia*	JX092046	matK
Caricaceae	*Vasconcellea quercifolia*	JX092047	matK
Caricaceae	*Vasconcellea quercifolia*	JX092083	ITS
Caricaceae	*Vasconcellea quercifolia*	JX092084	ITS
Caryocaraceae	*Caryocar brasiliense*	EU339920	trnL
Caryocaraceae	*Caryocar brasiliense*	EU339921	trnL
Caryocaraceae	*Caryocar brasiliense*	EU339922	trnL
Caryocaraceae	*Caryocar brasiliense*	EU339923	trnL
Caryocaraceae	*Caryocar brasiliense*	EU339924	trnL
Caryocaraceae	*Caryocar brasiliense*	EU339925	trnL
Caryocaraceae	*Caryocar brasiliense*	EU339926	trnL
Caryocaraceae	*Caryocar brasiliense*	EU339927	trnL
Caryocaraceae	*Caryocar brasiliense*	EU339928	trnL
Caryocaraceae	*Caryocar brasiliense*	EU339929	trnL
Caryocaraceae	*Caryocar brasiliense*	EU350258	psbA-trnH
Caryocaraceae	*Caryocar brasiliense*	EU350259	psbA-trnH
Caryocaraceae	*Caryocar brasiliense*	EU350260	psbA-trnH
Caryocaraceae	*Caryocar brasiliense*	EU350261	psbA-trnH
Caryocaraceae	*Caryocar brasiliense*	EU350262	psbA-trnH
Caryocaraceae	*Caryocar brasiliense*	EU350263	psbA-trnH
Caryocaraceae	*Caryocar brasiliense*	EU350264	psbA-trnH
Caryocaraceae	*Caryocar brasiliense*	EU350265	psbA-trnH
Caryocaraceae	*Caryocar brasiliense*	EU350266	psbA-trnH
Caryocaraceae	*Caryocar brasiliense*	EU350267	psbA-trnH
Casuarinaceae	*Casuarina equisetifolia*	AB015462	matK
Casuarinaceae	*Casuarina equisetifolia*	AB817433	trnL
Casuarinaceae	*Casuarina equisetifolia*	AY033837	matK

Family	Species	Accession	Barcode
Casuarinaceae	*Casuarina equisetifolia*	AY033858	rbcL
Casuarinaceae	*Casuarina equisetifolia*	AY033859	rbcL
Casuarinaceae	*Casuarina equisetifolia*	AY033874	matK
Casuarinaceae	*Casuarina equisetifolia*	AY147090	trnL
Casuarinaceae	*Casuarina equisetifolia*	AY191700	matK
Casuarinaceae	*Casuarina equisetifolia*	AY191701	matK
Casuarinaceae	*Casuarina equisetifolia*	AY191702	matK
Casuarinaceae	*Casuarina equisetifolia*	AY191703	matK
Casuarinaceae	*Casuarina equisetifolia*	AY263930	rbcL
Casuarinaceae	*Casuarina equisetifolia*	AY864057	ITS
Casuarinaceae	*Casuarina equisetifolia*	GU135038	matK
Casuarinaceae	*Casuarina equisetifolia*	GU135200	rbcL
Casuarinaceae	*Casuarina equisetifolia*	GU135367	psbA-trnH
Casuarinaceae	*Casuarina equisetifolia*	JX856868	psbA-trnH
Casuarinaceae	*Casuarina equisetifolia*	KC878591	ITS
Casuarinaceae	*Casuarina equisetifolia*	KC878592	ITS
Casuarinaceae	*Casuarina equisetifolia*	KC878593	ITS
Casuarinaceae	*Casuarina equisetifolia*	L01893	rbcL
Celastraceae	*Cheiloclinium cognatum*	FJ705511	ITS
Celastraceae	*Cheiloclinium cognatum*	FJ705538	matK
Celastraceae	*Cheiloclinium cognatum*	FJ705566	trnL
Celastraceae	*Cheiloclinium cognatum*	FJ705592	trnL
Celastraceae	*Cheiloclinium cognatum*	GQ428749	psbA-trnH
Celastraceae	*Cheiloclinium cognatum*	GQ428750	psbA-trnH
Celastraceae	*Cheiloclinium cognatum*	JN661741	psbA-trnH
Celastraceae	*Cheiloclinium cognatum*	JN661754	psbA-trnH
Celastraceae	*Cheiloclinium cognatum*	JQ626275	rbcL
Celastraceae	*Cheiloclinium cognatum*	JQ626564	matK
Celastraceae	*Maytenus boaria*	EU328753	ITS
Celastraceae	*Maytenus boaria*	EU328778	trnL
Celastraceae	*Maytenus boaria*	EU328862	trnL
Celastraceae	*Maytenus boaria*	EU328948	matK
Celastraceae	*Maytenus evonymoides*	HQ267108	matK
Celastraceae	*Maytenus evonymoides*	HQ267129	trnL
Celastraceae	*Maytenus evonymoides*	HQ267154	trnL

**Family**	**Species**	**Accession**	**Barcode**
Celastraceae	*Maytenus evonymoides*	HQ267180	ITS
Celastraceae	*Maytenus floribunda*	EU328751	ITS
Celastraceae	*Maytenus floribunda*	EU328792	trnL
Celastraceae	*Maytenus floribunda*	EU328863	trnL
Celastraceae	*Maytenus floribunda*	EU328961	matK
Celastraceae	*Maytenus gonoclada*	HQ267115	matK
Celastraceae	*Maytenus gonoclada*	HQ267116	matK
Celastraceae	*Maytenus gonoclada*	HQ267139	trnL
Celastraceae	*Maytenus gonoclada*	HQ267140	trnL
Celastraceae	*Maytenus gonoclada*	HQ267162	trnL
Celastraceae	*Maytenus gonoclada*	HQ267163	trnL
Celastraceae	*Maytenus gonoclada*	HQ267191	ITS
Celastraceae	*Maytenus gonoclada*	HQ267192	ITS
Celastraceae	*Maytenus ilicifolia*	HQ267103	matK
Celastraceae	*Maytenus ilicifolia*	HQ267110	matK
Celastraceae	*Maytenus ilicifolia*	HQ267126	trnL
Celastraceae	*Maytenus ilicifolia*	HQ267131	trnL
Celastraceae	*Maytenus ilicifolia*	HQ267149	trnL
Celastraceae	*Maytenus ilicifolia*	HQ267156	trnL
Celastraceae	*Maytenus ilicifolia*	HQ267174	ITS
Celastraceae	*Maytenus ilicifolia*	HQ267183	ITS
Celastraceae	*Maytenus ilicifolia*	KJ751174	matK
Celastraceae	*Maytenus ilicifolia*	KJ751403	rbcL
Celastraceae	*Maytenus ilicifolia*	KM519582	ITS
Celastraceae	*Plenckia populnea*	JF409991	ITS
Celastraceae	*Plenckia populnea*	JF409992	ITS
Celastraceae	*Plenckia populnea*	JF409993	ITS
Celastraceae	*Plenckia populnea*	JF410054	trnL
Celastraceae	*Plenckia populnea*	JF410092	matK
Celastraceae	*Plenckia populnea*	JF410096	matK
Celastraceae	*Plenckia populnea*	JF410098	matK
Celastraceae	*Salacia arborea*	FJ705531	ITS
Celastraceae	*Salacia arborea*	FJ705557	matK
Celastraceae	*Salacia arborea*	FJ705584	trnL
Celastraceae	*Salacia arborea*	FJ705609	trnL

**Family**	**Species**	**Accession**	**Barcode**
Celastraceae	*Salacia crassifolia*	FJ705522	ITS
Celastraceae	*Salacia crassifolia*	FJ705548	matK
Celastraceae	*Salacia crassifolia*	FJ705576	trnL
Celastraceae	*Salacia crassifolia*	FJ705601	trnL
Celastraceae	*Salacia elliptica*	FJ705524	ITS
Celastraceae	*Salacia elliptica*	FJ705550	matK
Celastraceae	*Salacia elliptica*	FJ705578	trnL
Celastraceae	*Salacia elliptica*	FJ705603	trnL
Celastraceae	*Salacia grandifolia*	HM230111	ITS
Celastraceae	*Salacia grandifolia*	HM230158	matK
Celastraceae	*Salacia grandifolia*	HM230204	trnL
Celastraceae	*Salacia grandifolia*	HM230249	trnL
Celastraceae	*Tontelea micrantha*	FJ705535	ITS
Celastraceae	*Tontelea micrantha*	FJ705563	matK
Celastraceae	*Tontelea micrantha*	FJ705590	trnL
Celastraceae	*Tontelea micrantha*	FJ705613	trnL
Chloranthaceae	*Hedyosmum brasiliense*	EU302161	ITS
Chloranthaceae	*Hedyosmum brasiliense*	EU302201	rbcL
Chloranthaceae	*Hedyosmum brasiliense*	HQ336526	rbcL
Chloranthaceae	*Hedyosmum brasiliense*	HQ336605	trnL
Chrysobalanaceae	*Chrysobalanus icaco*	EF135519	matK
Chrysobalanaceae	*Chrysobalanus icaco*	GQ424456	ITS
Chrysobalanaceae	*Chrysobalanus icaco*	GQ424476	rbcL
Chrysobalanaceae	*Chrysobalanus icaco*	JQ898692	rbcL
Chrysobalanaceae	*Chrysobalanus icaco*	JQ898693	rbcL
Chrysobalanaceae	*Chrysobalanus icaco*	JQ898850	matK
Chrysobalanaceae	*Chrysobalanus icaco*	JQ898857	matK
Chrysobalanaceae	*Chrysobalanus icaco*	JQ898919	psbA-trnH
Chrysobalanaceae	*Chrysobalanus icaco*	JQ898923	psbA-trnH
Chrysobalanaceae	*Chrysobalanus icaco*	JQ898992	ITS
Chrysobalanaceae	*Chrysobalanus icaco*	JQ898993	ITS
Chrysobalanaceae	*Chrysobalanus icaco*	JX661935	matK
Chrysobalanaceae	*Chrysobalanus icaco*	JX664040	rbcL
Chrysobalanaceae	*Chrysobalanus icaco*	JX664687	ycf1
Chrysobalanaceae	*Chrysobalanus icaco*	JX664929	rpoC1

Family	Species	Accession	Barcode
Chrysobalanaceae	*Chrysobalanus icaco*	KC627502	matK
Chrysobalanaceae	*Chrysobalanus icaco*	KC627653	matK
Chrysobalanaceae	*Chrysobalanus icaco*	KC628076	rbcL
Chrysobalanaceae	*Chrysobalanus icaco*	KC628284	rbcL
Chrysobalanaceae	*Chrysobalanus icaco*	KC667665	psbA-trnH
Chrysobalanaceae	*Chrysobalanus icaco*	KC667830	psbA-trnH
Chrysobalanaceae	*Chrysobalanus icaco*	KC688789	psbA-trnH
Chrysobalanaceae	*Chrysobalanus icaco*	KJ012509	matK
Chrysobalanaceae	*Chrysobalanus icaco*	KJ082186	rbcL
Chrysobalanaceae	*Chrysobalanus icaco*	KJ414477	ITS
Chrysobalanaceae	*Chrysobalanus icaco*	L11178	rbcL
Chrysobalanaceae	*Hirtella glandulosa*	DQ787419	ITS
Chrysobalanaceae	*Hirtella glandulosa*	FJ037810	ITS
Chrysobalanaceae	*Hirtella glandulosa*	FJ038001	rbcL
Chrysobalanaceae	*Hirtella glandulosa*	FJ038244	rpoB
Chrysobalanaceae	*Hirtella glandulosa*	FJ038465	rpoC1
Chrysobalanaceae	*Hirtella glandulosa*	FJ038466	rpoC1
Chrysobalanaceae	*Hirtella glandulosa*	FJ038467	rpoC1
Chrysobalanaceae	*Hirtella glandulosa*	FJ038894	psbA-trnH
Chrysobalanaceae	*Hirtella glandulosa*	FJ039266	trnL
Chrysobalanaceae	*Hirtella glandulosa*	FJ039267	trnL
Chrysobalanaceae	*Hirtella glandulosa*	FJ817191	rpoB
Chrysobalanaceae	*Hirtella glandulosa*	JQ898733	rbcL
Chrysobalanaceae	*Hirtella glandulosa*	JQ898891	psbA-trnH
Chrysobalanaceae	*Hirtella glandulosa*	JQ898986	ITS
Chrysobalanaceae	*Hirtella hebeclada*	JQ898746	rbcL
Chrysobalanaceae	*Hirtella hebeclada*	JQ898843	matK
Chrysobalanaceae	*Hirtella hebeclada*	JQ898915	psbA-trnH
Chrysobalanaceae	*Hirtella hebeclada*	JQ899020	ITS
Chrysobalanaceae	*Hirtella racemosa*	GQ981763	rbcL
Chrysobalanaceae	*Hirtella racemosa*	GQ982247	psbA-trnH
Chrysobalanaceae	*Hirtella racemosa*	JQ591068	rbcL
Chrysobalanaceae	*Hirtella racemosa*	JQ591069	rbcL
Chrysobalanaceae	*Hirtella racemosa*	JQ591070	rbcL
Chrysobalanaceae	*Hirtella racemosa*	JQ594779	rbcL

Family	Species	Accession	Barcode
Chrysobalanaceae	*Hirtella racemosa*	KJ414472	ITS
Chrysobalanaceae	*Licania kunthiana*	JQ625860	rbcL
Chrysobalanaceae	*Licania kunthiana*	JQ898714	rbcL
Chrysobalanaceae	*Licania kunthiana*	JQ899007	ITS
Chrysobalanaceae	*Licania octandra*	JQ625798	rbcL
Chrysobalanaceae	*Licania octandra*	JQ898718	rbcL
Chrysobalanaceae	*Licania octandra*	JQ898855	matK
Chrysobalanaceae	*Licania octandra*	JQ899025	ITS
Chrysobalanaceae	*Licania tomentosa*	GQ424464	ITS
Chrysobalanaceae	*Licania tomentosa*	GQ424484	rbcL
Chrysobalanaceae	*Licania tomentosa*	L11193	rbcL
Chrysobalanaceae	*Parinari excelsa*	JQ625960	rbcL
Chrysobalanaceae	*Parinari excelsa*	JQ898741	rbcL
Chrysobalanaceae	*Parinari excelsa*	JQ898842	matK
Chrysobalanaceae	*Parinari excelsa*	JQ898907	psbA-trnH
Chrysobalanaceae	*Parinari excelsa*	JQ898977	ITS
Chrysobalanaceae	*Parinari excelsa*	JQ899019	ITS
Chrysobalanaceae	*Parinari excelsa*	KF147501	rbcL
Clethraceae	*Clethra scabra*	AY190559	ITS
Clethraceae	*Clethra scabra*	AY190580	trnL
Clusiaceae	*Clusia criuva*	AB450037	matK
Clusiaceae	*Clusia criuva*	AJ414713	ITS
Clusiaceae	*Clusia criuva*	AY145187	ITS
Clusiaceae	*Clusia grandiflora*	AY144057	trnL
Clusiaceae	*Clusia grandiflora*	AY144083	trnL
Clusiaceae	*Clusia grandiflora*	AY145218	ITS
Clusiaceae	*Clusia grandiflora*	FJ038013	rbcL
Clusiaceae	*Clusia grandiflora*	FJ038250	rpoB
Clusiaceae	*Clusia grandiflora*	FJ038498	rpoC1
Clusiaceae	*Clusia grandiflora*	FJ038905	psbA-trnH
Clusiaceae	*Clusia grandiflora*	FJ514669	matK
Clusiaceae	*Clusia grandiflora*	JQ626019	rbcL
Clusiaceae	*Clusia grandiflora*	JQ626447	matK
Clusiaceae	*Clusia lanceolata*	AJ414716	ITS
Clusiaceae	*Clusia lanceolata*	AY144059	trnL

Family	Species	Accession	Barcode
Clusiaceae	*Clusia lanceolata*	AY144085	trnL
Clusiaceae	*Clusia lanceolata*	AY145195	ITS
Clusiaceae	*Clusia lanceolata*	HQ331579	matK
Clusiaceae	*Clusia lanceolata*	HQ332039	rbcL
Clusiaceae	*Tovomitopsis saldanhae*	AY145240	ITS
Clusiaceae	*Tovomitopsis saldanhae*	HQ331687	matK
Clusiaceae	*Tovomitopsis saldanhae*	HQ332123	rbcL
Combretaceae	*Combretum laxum*	KJ593833	matK
Combretaceae	*Combretum laxum*	KJ593834	matK
Combretaceae	*Combretum laxum*	KJ594191	rbcL
Combretaceae	*Combretum laxum*	KJ594192	rbcL
Combretaceae	*Combretum laxum*	KJ594193	rbcL
Combretaceae	*Conocarpus erectus*	AY050562	ITS
Combretaceae	*Conocarpus erectus*	AY179339	trnL
Combretaceae	*Conocarpus erectus*	AY179341	trnL
Combretaceae	*Conocarpus erectus*	JQ587268	matK
Combretaceae	*Conocarpus erectus*	JQ587269	matK
Combretaceae	*Conocarpus erectus*	JQ587270	matK
Combretaceae	*Conocarpus erectus*	JQ589987	matK
Combretaceae	*Conocarpus erectus*	JQ589988	matK
Combretaceae	*Conocarpus erectus*	JQ589989	matK
Combretaceae	*Conocarpus erectus*	JQ589990	matK
Combretaceae	*Conocarpus erectus*	JQ591118	rbcL
Combretaceae	*Conocarpus erectus*	JQ591119	rbcL
Combretaceae	*Conocarpus erectus*	JQ591120	rbcL
Combretaceae	*Conocarpus erectus*	JQ591121	rbcL
Combretaceae	*Conocarpus erectus*	KJ012545	matK
Combretaceae	*Conocarpus erectus*	KJ082235	rbcL
Combretaceae	*Laguncularia racemosa*	AF425685	ITS
Combretaceae	*Laguncularia racemosa*	AF425686	ITS
Combretaceae	*Laguncularia racemosa*	AF425715	rbcL
Combretaceae	*Laguncularia racemosa*	AF425716	rbcL
Combretaceae	*Laguncularia racemosa*	FJ381787	ITS
Combretaceae	*Laguncularia racemosa*	FJ381825	rbcL
Combretaceae	*Laguncularia racemosa*	FJ381826	rbcL

**Family**	**Species**	**Accession**	**Barcode**
Combretaceae	*Laguncularia racemosa*	KJ012652	matK
Combretaceae	*Laguncularia racemosa*	KJ082379	rbcL
Combretaceae	*Laguncularia racemosa*	KJ772885	matK
Combretaceae	*Terminalia catappa*	AB817424	trnL
Combretaceae	*Terminalia catappa*	AB817635	trnL
Combretaceae	*Terminalia catappa*	FJ381811	rbcL
Combretaceae	*Terminalia catappa*	FJ381882	psbA-trnH
Combretaceae	*Terminalia catappa*	GU135057	matK
Combretaceae	*Terminalia catappa*	GU135220	rbcL
Combretaceae	*Terminalia catappa*	GU135388	psbA-trnH
Combretaceae	*Terminalia catappa*	JF747601	rbcL
Combretaceae	*Terminalia catappa*	JX518026	matK
Combretaceae	*Terminalia catappa*	JX573036	rbcL
Combretaceae	*Terminalia catappa*	JX856522	ITS
Combretaceae	*Terminalia catappa*	KF848301	ITS
Combretaceae	*Terminalia catappa*	KJ784585	ITS
Combretaceae	*Terminalia catappa*	KM030002	rbcL
Combretaceae	*Terminalia catappa*	KM030007	trnL
Combretaceae	*Terminalia catappa*	KP319001	psbA-trnH
Combretaceae	*Terminalia catappa*	KP319002	psbA-trnH
Combretaceae	*Terminalia catappa*	KP319003	psbA-trnH
Combretaceae	*Terminalia catappa*	KT235566	ITS
Combretaceae	*Terminalia catappa*	KT274011	rbcL
Combretaceae	*Terminalia catappa*	KT274012	rbcL
Combretaceae	*Terminalia catappa*	KT279736	ITS
Combretaceae	*Terminalia catappa*	KT279737	ITS
Combretaceae	*Terminalia catappa*	KT279741	rbcL
Combretaceae	*Terminalia catappa*	LC050568	ITS
Combretaceae	*Terminalia catappa*	U26338	rbcL
Cunoniaceae	*Lamanonia ternata*	JX236029	trnL
Cunoniaceae	*Lamanonia ternata*	JX236032	rbcL
Cunoniaceae	*Lamanonia ternata*	KF420990	ITS
Cunoniaceae	*Lamanonia ternata*	KF420991	ITS
Cunoniaceae	*Lamanonia ternata*	KF420992	ITS
Cunoniaceae	*Lamanonia ternata*	KF420993	ITS

Family	Species	Accession	Barcode
Cunoniaceae	*Lamanonia ternata*	KF420994	ITS
Cunoniaceae	*Lamanonia ternata*	KF421051	psbA-trnH
Cunoniaceae	*Lamanonia ternata*	KF421052	psbA-trnH
Cunoniaceae	*Lamanonia ternata*	KF421053	psbA-trnH
Cunoniaceae	*Lamanonia ternata*	KF421054	psbA-trnH
Cunoniaceae	*Lamanonia ternata*	KF421055	psbA-trnH
Cunoniaceae	*Lamanonia ternata*	KF421056	psbA-trnH
Cunoniaceae	*Lamanonia ternata*	KF555403	matK
Cunoniaceae	*Lamanonia ternata*	KF555404	matK
Cunoniaceae	*Lamanonia ternata*	KF561922	rbcL
Cunoniaceae	*Lamanonia ternata*	KF561923	rbcL
Cunoniaceae	*Lamanonia ternata*	KF667928	rbcL
Cyatheaceae	*Alsophila capensis*	AM177321	rbcL
Cyatheaceae	*Alsophila capensis*	AM410316	trnL
Cyatheaceae	*Cyathea microdonta*	EU554324	trnL
Cyatheaceae	*Cyathea microdonta*	KR082865	rbcL
Cyatheaceae	*Cyathea pungens*	KR082853	rbcL
Cyatheaceae	*Cyathea pungens*	KR082857	rbcL
Dicksoniaceae	*Dicksonia sellowiana*	AM177342	rbcL
Dicksoniaceae	*Dicksonia sellowiana*	AM410357	trnL
Dicksoniaceae	*Dicksonia sellowiana*	EU090940	ITS
Dilleniaceae	*Curatella americana*	AJ419729	rbcL
Dilleniaceae	*Curatella americana*	FJ860341	rbcL
Dilleniaceae	*Curatella americana*	JQ587382	matK
Dilleniaceae	*Curatella americana*	JQ587383	matK
Dilleniaceae	*Curatella americana*	JQ587384	matK
Dilleniaceae	*Curatella americana*	JQ587385	matK
Dilleniaceae	*Curatella americana*	JQ591308	rbcL
Dilleniaceae	*Curatella americana*	JQ591309	rbcL
Dilleniaceae	*Curatella americana*	JQ591310	rbcL
Dilleniaceae	*Curatella americana*	JQ591311	rbcL
Dilleniaceae	*Curatella americana*	JQ591312	rbcL
Dilleniaceae	*Curatella americana*	JQ591313	rbcL
Dilleniaceae	*Davilla elliptica*	FJ860342	rbcL
Elaeocarpaceae	*Sloanea garckeana*	JQ626000	rbcL

**Family**	**Species**	**Accession**	**Barcode**
Elaeocarpaceae	*Sloanea garckeana*	JQ626436	matK
Elaeocarpaceae	*Sloanea guianensis*	FJ038022	rbcL
Elaeocarpaceae	*Sloanea guianensis*	FJ038260	rpoB
Elaeocarpaceae	*Sloanea guianensis*	FJ038508	rpoC1
Elaeocarpaceae	*Sloanea guianensis*	FJ038914	psbA-trnH
Elaeocarpaceae	*Sloanea guianensis*	FJ514674	matK
Elaeocarpaceae	*Sloanea guianensis*	JQ626032	rbcL
Elaeocarpaceae	*Sloanea guianensis*	JQ626451	matK
Elaeocarpaceae	*Sloanea terniflora*	GQ981874	rbcL
Elaeocarpaceae	*Sloanea terniflora*	GQ982094	matK
Elaeocarpaceae	*Sloanea terniflora*	GQ982362	psbA-trnH
Elaeocarpaceae	*Sloanea terniflora*	JQ587411	matK
Elaeocarpaceae	*Sloanea terniflora*	JQ587412	matK
Elaeocarpaceae	*Sloanea terniflora*	JQ587413	matK
Elaeocarpaceae	*Sloanea terniflora*	JQ587414	matK
Elaeocarpaceae	*Sloanea terniflora*	JQ591340	rbcL
Elaeocarpaceae	*Sloanea terniflora*	JQ591341	rbcL
Ericaceae	*Gaultheria eriophylla*	AF358884	ITS
Ericaceae	*Gaultheria eriophylla*	JF801336	matK
Ericaceae	*Gaultheria eriophylla*	JF801337	matK
Ericaceae	*Gaultheria eriophylla*	JF801588	ITS
Ericaceae	*Gaultheria eriophylla*	JF801589	ITS
Ericaceae	*Gaultheria eriophylla*	JF801670	trnL
Ericaceae	*Gaultheria eriophylla*	JF801671	trnL
Ericaceae	*Gaultheria eriophylla*	L12618	rbcL
Ericaceae	*Gaultheria eriophylla*	U61317	matK
Ericaceae	*Gaylussacia brasiliensis*	AF271713	trnL
Ericaceae	*Gaylussacia brasiliensis*	AF273722	ITS
Erythroxylaceae	*Erythroxylum amplifolium*	DQ787423	ITS
Erythroxylaceae	*Erythroxylum argentinum*	AF127731	trnL
Erythroxylaceae	*Erythroxylum argentinum*	AF130316	ITS
Erythroxylaceae	*Erythroxylum deciduum*	KF420999	ITS
Erythroxylaceae	*Erythroxylum deciduum*	KF421000	ITS
Erythroxylaceae	*Erythroxylum deciduum*	KF421001	ITS
Erythroxylaceae	*Erythroxylum deciduum*	KF421049	psbA-trnH

Family	Species	Accession	Barcode
Erythroxylaceae	*Erythroxylum deciduum*	KF421050	psbA-trnH
Erythroxylaceae	*Erythroxylum deciduum*	KF561912	rbcL
Erythroxylaceae	*Erythroxylum deciduum*	KF561913	rbcL
Erythroxylaceae	*Erythroxylum suberosum*	JX661939	matK
Erythroxylaceae	*Erythroxylum suberosum*	JX663472	rpoB
Erythroxylaceae	*Erythroxylum suberosum*	JX664044	rbcL
Erythroxylaceae	*Erythroxylum suberosum*	JX664690	ycf1
Erythroxylaceae	*Erythroxylum suberosum*	JX664934	rpoC1
Erythroxylaceae	*Erythroxylum suberosum*	KJ012579	matK
Erythroxylaceae	*Erythroxylum suberosum*	KJ082286	rbcL
Erythroxylaceae	*Erythroxylum suberosum*	KJ082287	rbcL
Erythroxylaceae	*Erythroxylum suberosum*	KJ426715	psbA-trnH
Escalloniaceae	*Escallonia bifida*	KC355678	psbA-trnH
Escalloniaceae	*Escallonia bifida*	KC355679	psbA-trnH
Escalloniaceae	*Escallonia bifida*	KC355680	psbA-trnH
Escalloniaceae	*Escallonia megapotamica*	KC355706	psbA-trnH
Escalloniaceae	*Escallonia megapotamica*	KC355707	psbA-trnH
Escalloniaceae	*Escallonia megapotamica*	KC355708	psbA-trnH
Escalloniaceae	*Escallonia megapotamica*	KC355709	psbA-trnH
Euphorbiaceae	*Acalypha macrostachya*	GQ981654	rbcL
Euphorbiaceae	*Acalypha macrostachya*	GQ981926	matK
Euphorbiaceae	*Acalypha macrostachya*	GQ982135	psbA-trnH
Euphorbiaceae	*Acalypha macrostachya*	HG971790	trnL
Euphorbiaceae	*Acalypha macrostachya*	JQ587422	matK
Euphorbiaceae	*Acalypha macrostachya*	JQ591364	rbcL
Euphorbiaceae	*Acalypha macrostachya*	JQ591365	rbcL
Euphorbiaceae	*Acalypha macrostachya*	JQ591366	rbcL
Euphorbiaceae	*Acalypha macrostachya*	JQ591367	rbcL
Euphorbiaceae	*Acalypha macrostachya*	JQ591368	rbcL
Euphorbiaceae	*Acalypha macrostachya*	JQ591369	rbcL
Euphorbiaceae	*Actinostemon concolor*	AB233779	matK
Euphorbiaceae	*Actinostemon concolor*	AB233883	rbcL
Euphorbiaceae	*Adelia membranifolia*	DQ997786	ITS
Euphorbiaceae	*Adelia membranifolia*	DQ997803	rbcL
Euphorbiaceae	*Adelia membranifolia*	DQ997808	trnL

Family	Species	Accession	Barcode
Euphorbiaceae	*Aparisthmium cordatum*	AY794793	trnL
Euphorbiaceae	*Aparisthmium cordatum*	AY794955	rbcL
Euphorbiaceae	*Bernardia pulchella*	HG971826	trnL
Euphorbiaceae	*Croton alchorneicarpus*	HM044769	trnL
Euphorbiaceae	*Croton alchorneicarpus*	HM044788	ITS
Euphorbiaceae	*Croton alchorneicarpus*	HM044811	psbA-trnH
Euphorbiaceae	*Croton campanulatus*	HM044771	trnL
Euphorbiaceae	*Croton campanulatus*	HM044790	ITS
Euphorbiaceae	*Croton campanulatus*	HM044815	psbA-trnH
Euphorbiaceae	*Croton celtidifolius*	EU586920	ITS
Euphorbiaceae	*Croton celtidifolius*	EU586975	trnL
Euphorbiaceae	*Croton floribundus*	HM564080	ITS
Euphorbiaceae	*Croton gracilipes*	EU586909	ITS
Euphorbiaceae	*Croton gracilipes*	EU586962	trnL
Euphorbiaceae	*Croton gracilipes*	HM044823	psbA-trnH
Euphorbiaceae	*Croton hemiargyreus*	HM044774	trnL
Euphorbiaceae	*Croton hemiargyreus*	HM044793	ITS
Euphorbiaceae	*Croton hemiargyreus*	HM044824	psbA-trnH
Euphorbiaceae	*Croton macrobothrys*	EU586928	ITS
Euphorbiaceae	*Croton macrobothrys*	EU586984	trnL
Euphorbiaceae	*Croton organensis*	EU586914	ITS
Euphorbiaceae	*Croton organensis*	EU586969	trnL
Euphorbiaceae	*Croton organensis*	HM044832	psbA-trnH
Euphorbiaceae	*Croton piptocalyx*	EF405858	rbcL
Euphorbiaceae	*Croton piptocalyx*	EF408132	trnL
Euphorbiaceae	*Croton piptocalyx*	EF408148	trnL
Euphorbiaceae	*Croton piptocalyx*	EF421790	ITS
Euphorbiaceae	*Croton piptocalyx*	EF421791	ITS
Euphorbiaceae	*Croton piptocalyx*	HM044836	psbA-trnH
Euphorbiaceae	*Croton priscus*	EU586950	ITS
Euphorbiaceae	*Croton priscus*	EU587002	trnL
Euphorbiaceae	*Croton rottlerifolius*	HM044781	trnL
Euphorbiaceae	*Croton rottlerifolius*	HM044801	ITS
Euphorbiaceae	*Croton rottlerifolius*	HM044838	psbA-trnH
Euphorbiaceae	*Croton salutaris*	HM044783	trnL

Family	Species	Accession	Barcode
Euphorbiaceae	*Croton salutaris*	HM044803	ITS
Euphorbiaceae	*Croton salutaris*	HM044804	ITS
Euphorbiaceae	*Croton salutaris*	HM044840	psbA-trnH
Euphorbiaceae	*Croton salutaris*	HM071959	ITS
Euphorbiaceae	*Croton salutaris*	HM071977	trnL
Euphorbiaceae	*Croton sphaerogynus*	HM044784	trnL
Euphorbiaceae	*Croton sphaerogynus*	HM044805	ITS
Euphorbiaceae	*Croton sphaerogynus*	HM044843	psbA-trnH
Euphorbiaceae	*Croton urucurana*	EU586937	ITS
Euphorbiaceae	*Croton urucurana*	EU586991	trnL
Euphorbiaceae	*Euphorbia comosa*	AF537503	ITS
Euphorbiaceae	*Euphorbia comosa*	JN249619	trnL
Euphorbiaceae	*Euphorbia comosa*	JN250128	ITS
Euphorbiaceae	*Gymnanthes klotzschiana*	AY794640	trnL
Euphorbiaceae	*Gymnanthes klotzschiana*	AY794850	rbcL
Euphorbiaceae	*Jatropha curcas*	KC984645	ITS
Euphorbiaceae	*Jatropha curcas*	KP827667	rbcL
Euphorbiaceae	*Joannesia princeps*	AJ418808	rbcL
Euphorbiaceae	*Joannesia princeps*	AY794686	trnL
Euphorbiaceae	*Mabea piriri*	JQ625733	rbcL
Euphorbiaceae	*Mabea piriri*	JQ626343	matK
Euphorbiaceae	*Manihot anomala*	EU518898	trnL
Euphorbiaceae	*Manihot anomala*	FN551987	trnL
Euphorbiaceae	*Manihot caerulescens*	EU518900	trnL
Euphorbiaceae	*Manihot caerulescens*	FN551959	trnL
Euphorbiaceae	*Manihot grahamii*	AY794680	trnL
Euphorbiaceae	*Manihot grahamii*	AY794875	rbcL
Euphorbiaceae	*Manihot grahamii*	FJ670014	matK
Euphorbiaceae	*Manihot grahamii*	FN551958	trnL
Euphorbiaceae	*Manihot pilosa*	EU518919	trnL
Euphorbiaceae	*Manihot pilosa*	FN551951	trnL
Euphorbiaceae	*Manihot pilosa*	FN551979	trnL
Euphorbiaceae	*Manihot pilosa*	FN551993	trnL
Euphorbiaceae	*Maprounea guianensis*	AJ418810	rbcL
Euphorbiaceae	*Maprounea guianensis*	AY794658	trnL

Family	Species	Accession	Barcode
Euphorbiaceae	*Maprounea guianensis*	DQ787404	ITS
Euphorbiaceae	*Maprounea guianensis*	EF135563	matK
Euphorbiaceae	*Maprounea guianensis*	JN250096	ITS
Euphorbiaceae	*Maprounea guianensis*	JQ625792	rbcL
Euphorbiaceae	*Pachystroma longifolium*	AY794637	trnL
Euphorbiaceae	*Pachystroma longifolium*	AY794847	rbcL
Euphorbiaceae	*Philyra brasiliensis*	AB267927	rbcL
Euphorbiaceae	*Philyra brasiliensis*	AB268031	matK
Euphorbiaceae	*Philyra brasiliensis*	AY794740	trnL
Euphorbiaceae	*Philyra brasiliensis*	AY794920	rbcL
Euphorbiaceae	*Philyra brasiliensis*	DQ997800	ITS
Euphorbiaceae	*Philyra brasiliensis*	GU000028	ITS
Euphorbiaceae	*Philyra brasiliensis*	HG971914	trnL
Euphorbiaceae	*Sapium glandulosum*	AY794626	trnL
Euphorbiaceae	*Sapium glandulosum*	AY794841	rbcL
Euphorbiaceae	*Sapium glandulosum*	GQ981869	rbcL
Euphorbiaceae	*Sapium glandulosum*	GQ982089	matK
Euphorbiaceae	*Sapium glandulosum*	GQ982356	psbA-trnH
Euphorbiaceae	*Sapium glandulosum*	JQ589777	matK
Euphorbiaceae	*Sapium glandulosum*	JQ589778	matK
Euphorbiaceae	*Sapium glandulosum*	JQ589779	matK
Euphorbiaceae	*Sapium glandulosum*	JQ591513	rbcL
Euphorbiaceae	*Sapium glandulosum*	JQ591514	rbcL
Euphorbiaceae	*Sapium glandulosum*	JQ591515	rbcL
Euphorbiaceae	*Sapium glandulosum*	JQ591516	rbcL
Euphorbiaceae	*Sapium glandulosum*	JQ594785	rbcL
Euphorbiaceae	*Sapium glandulosum*	JQ594786	rbcL
Euphorbiaceae	*Sapium haematospermum*	AY794627	trnL
Euphorbiaceae	*Sebastiania brasiliensis*	AY794624	trnL
Euphorbiaceae	*Stillingia oppositifolia*	AY794632	trnL
Euphorbiaceae	*Tetrorchidium rubrivenium*	AB267952	rbcL
Euphorbiaceae	*Tetrorchidium rubrivenium*	AB268056	matK
Fabaceae	*Acacia mearnsii*	AF195675	trnL
Fabaceae	*Acacia mearnsii*	AF195694	trnL
Fabaceae	*Acacia mearnsii*	AF195707	psbA-trnH

Family	Species	Accession	Barcode
Fabaceae	*Acacia mearnsii*	AF360705	ITS
Fabaceae	*Acacia mearnsii*	AY864897	ITS
Fabaceae	*Acacia mearnsii*	DQ489976	ITS
Fabaceae	*Acacia mearnsii*	HM020723	matK
Fabaceae	*Acacia mearnsii*	JF420062	ITS
Fabaceae	*Acacia mearnsii*	JF420078	ITS
Fabaceae	*Acacia mearnsii*	JF420160	psbA-trnH
Fabaceae	*Acacia mearnsii*	JF420174	psbA-trnH
Fabaceae	*Acacia mearnsii*	JF420188	psbA-trnH
Fabaceae	*Acacia mearnsii*	JF420379	trnL
Fabaceae	*Acacia mearnsii*	JF420393	trnL
Fabaceae	*Acacia mearnsii*	JF420408	trnL
Fabaceae	*Acacia mearnsii*	JF420486	trnL
Fabaceae	*Acacia mearnsii*	JF420511	trnL
Fabaceae	*Acacia mearnsii*	JX517946	matK
Fabaceae	*Acacia mearnsii*	JX572209	rbcL
Fabaceae	*Acacia mearnsii*	KF048786	ITS
Fabaceae	*Acacia mearnsii*	KF531952	matK
Fabaceae	*Acacia mearnsii*	KF531967	matK
Fabaceae	*Acacia mearnsii*	KF531990	psbA-trnH
Fabaceae	*Acacia mearnsii*	KF532006	psbA-trnH
Fabaceae	*Acacia mearnsii*	KF532029	rbcL
Fabaceae	*Acacia mearnsii*	KF532045	rbcL
Fabaceae	*Acacia mearnsii*	KF532066	ITS
Fabaceae	*Acacia mearnsii*	KF532082	ITS
Fabaceae	*Albizia niopoides*	HG963561	psbA-trnH
Fabaceae	*Albizia niopoides*	JQ587505	matK
Fabaceae	*Albizia niopoides*	JQ587506	matK
Fabaceae	*Albizia niopoides*	JQ587507	matK
Fabaceae	*Albizia niopoides*	JQ591570	rbcL
Fabaceae	*Albizia niopoides*	JQ591571	rbcL
Fabaceae	*Albizia niopoides*	JQ591572	rbcL
Fabaceae	*Albizia pedicellaris*	JQ625907	rbcL
Fabaceae	*Albizia pedicellaris*	JX870657	ITS
Fabaceae	*Albizia pedicellaris*	JX870789	trnL

**Family**	**Species**	**Accession**	**Barcode**
Fabaceae	*Albizia polycephala*	JX870656	ITS
Fabaceae	*Albizia polycephala*	JX870788	trnL
Fabaceae	*Albizia polycephala*	KF921821	psbA-trnH
Fabaceae	*Albizia polycephala*	KF933275	ITS
Fabaceae	*Albizia polycephala*	KF933279	trnL
Fabaceae	*Anadenanthera colubrina*	AF278481	trnL
Fabaceae	*Anadenanthera colubrina*	AF521813	matK
Fabaceae	*Anadenanthera colubrina*	AF522947	trnL
Fabaceae	*Anadenanthera colubrina*	AF523114	matK
Fabaceae	*Anadenanthera colubrina*	AF524967	psbA-trnH
Fabaceae	*Anadenanthera colubrina*	DQ344571	trnL
Fabaceae	*Anadenanthera colubrina*	DQ344605	trnL
Fabaceae	*Anadenanthera colubrina*	DQ787408	ITS
Fabaceae	*Anadenanthera colubrina*	EU440041	trnL
Fabaceae	*Anadenanthera colubrina*	EU812000	psbA-trnH
Fabaceae	*Anadenanthera colubrina*	EU812064	matK
Fabaceae	*Anadenanthera colubrina*	HF564640	ITS
Fabaceae	*Anadenanthera colubrina*	HF564641	ITS
Fabaceae	*Anadenanthera colubrina*	HF564642	ITS
Fabaceae	*Anadenanthera colubrina*	JQ910930	ITS
Fabaceae	*Anadenanthera colubrina*	KF787090	trnL
Fabaceae	*Anadenanthera colubrina*	LN827633	ITS
Fabaceae	*Anadenanthera colubrina*	LN827634	ITS
Fabaceae	*Anadenanthera colubrina*	LN827635	ITS
Fabaceae	*Anadenanthera colubrina*	LN827636	ITS
Fabaceae	*Anadenanthera colubrina*	LN827637	ITS
Fabaceae	*Anadenanthera colubrina*	LN827638	ITS
Fabaceae	*Anadenanthera colubrina*	LN827639	ITS
Fabaceae	*Anadenanthera colubrina*	LN827640	ITS
Fabaceae	*Anadenanthera colubrina*	LN827641	ITS
Fabaceae	*Anadenanthera colubrina*	LN827642	ITS
Fabaceae	*Anadenanthera colubrina*	LN827643	ITS
Fabaceae	*Anadenanthera colubrina*	LN827644	ITS
Fabaceae	*Anadenanthera colubrina*	LN827645	ITS
Fabaceae	*Anadenanthera colubrina*	LN827646	ITS

Family	Species	Accession	Barcode
Fabaceae	*Anadenanthera colubrina*	LN827647	ITS
Fabaceae	*Anadenanthera colubrina*	LN827648	ITS
Fabaceae	*Anadenanthera colubrina*	LN827649	ITS
Fabaceae	*Anadenanthera colubrina*	LN827650	ITS
Fabaceae	*Anadenanthera colubrina*	LN827651	ITS
Fabaceae	*Anadenanthera colubrina*	LN827652	ITS
Fabaceae	*Anadenanthera colubrina*	LN827653	ITS
Fabaceae	*Anadenanthera colubrina*	LN827654	ITS
Fabaceae	*Anadenanthera colubrina*	LN827655	ITS
Fabaceae	*Anadenanthera colubrina*	LN827656	ITS
Fabaceae	*Anadenanthera peregrina*	AF278480	trnL
Fabaceae	*Anadenanthera peregrina*	AF521814	matK
Fabaceae	*Anadenanthera peregrina*	EU440022	trnL
Fabaceae	*Anadenanthera peregrina*	EU811982	psbA-trnH
Fabaceae	*Anadenanthera peregrina*	HF545437	trnL
Fabaceae	*Anadenanthera peregrina*	KJ082119	rbcL
Fabaceae	*Anadenanthera peregrina*	KJ426604	psbA-trnH
Fabaceae	*Andira anthelmia*	FJ542763	ITS
Fabaceae	*Andira anthelmia*	FJ542764	ITS
Fabaceae	*Andira anthelmia*	JX275930	trnL
Fabaceae	*Andira fraxinifolia*	FJ542769	ITS
Fabaceae	*Andira fraxinifolia*	FJ542770	ITS
Fabaceae	*Andira humilis*	FJ542773	ITS
Fabaceae	*Andira humilis*	FJ542774	ITS
Fabaceae	*Andira humilis*	FJ542775	ITS
Fabaceae	*Andira humilis*	FJ542776	ITS
Fabaceae	*Andira humilis*	JX275924	trnL
Fabaceae	*Andira humilis*	JX295960	matK
Fabaceae	*Andira humilis*	JX295961	matK
Fabaceae	*Andira inermis*	AF309853	trnL
Fabaceae	*Andira inermis*	AY635469	ITS
Fabaceae	*Andira inermis*	AY635470	ITS
Fabaceae	*Andira inermis*	AY635471	ITS
Fabaceae	*Andira inermis*	AY635472	ITS
Fabaceae	*Andira inermis*	AY635473	ITS

**Family**	**Species**	**Accession**	**Barcode**
Fabaceae	*Andira inermis*	AY635474	ITS
Fabaceae	*Andira inermis*	AY635475	ITS
Fabaceae	*Andira inermis*	AY635476	ITS
Fabaceae	*Andira inermis*	AY635477	ITS
Fabaceae	*Andira inermis*	AY635478	ITS
Fabaceae	*Andira inermis*	AY635479	ITS
Fabaceae	*Andira inermis*	AY635480	ITS
Fabaceae	*Andira inermis*	FJ542777	ITS
Fabaceae	*Andira inermis*	FJ542778	ITS
Fabaceae	*Andira inermis*	FJ542779	ITS
Fabaceae	*Andira inermis*	FJ542780	ITS
Fabaceae	*Andira inermis*	FJ542781	ITS
Fabaceae	*Andira inermis*	FJ542782	ITS
Fabaceae	*Andira inermis*	FJ542783	ITS
Fabaceae	*Andira inermis*	FJ542784	ITS
Fabaceae	*Andira inermis*	GQ429072	matK
Fabaceae	*Andira inermis*	GQ429099	rpoC1
Fabaceae	*Andira inermis*	GQ429134	psbA-trnH
Fabaceae	*Andira inermis*	GQ981663	rbcL
Fabaceae	*Andira inermis*	GQ982145	psbA-trnH
Fabaceae	*Andira inermis*	HG963872	psbA-trnH
Fabaceae	*Andira inermis*	HM446757	rbcL
Fabaceae	*Andira inermis*	HM446886	psbA-trnH
Fabaceae	*Andira inermis*	JF501102	matK
Fabaceae	*Andira inermis*	JQ591576	rbcL
Fabaceae	*Andira inermis*	JQ591577	rbcL
Fabaceae	*Andira inermis*	JQ625802	rbcL
Fabaceae	*Andira inermis*	JX987565	rbcL
Fabaceae	*Andira inermis*	JX987566	rbcL
Fabaceae	*Andira inermis*	JX987567	rbcL
Fabaceae	*Andira inermis*	JX997361	psbA-trnH
Fabaceae	*Andira inermis*	JX997362	psbA-trnH
Fabaceae	*Andira inermis*	JX997363	psbA-trnH
Fabaceae	*Andira inermis*	JX997364	psbA-trnH
Fabaceae	*Andira inermis*	JX997365	psbA-trnH

Family	Species	Accession	Barcode
Fabaceae	*Andira inermis*	JX997366	psbA-trnH
Fabaceae	*Andira inermis*	U74199	rbcL
Fabaceae	*Andira ormosioides*	FJ542798	ITS
Fabaceae	*Andira ormosioides*	JX275927	trnL
Fabaceae	*Andira ormosioides*	JX295962	matK
Fabaceae	*Andira ormosioides*	JX295963	matK
Fabaceae	*Andira vermifuga*	FJ542808	ITS
Fabaceae	*Apuleia leiocarpa*	EU361737	trnL
Fabaceae	*Apuleia leiocarpa*	EU361858	matK
Fabaceae	*Apuleia leiocarpa*	U74249	rbcL
Fabaceae	*Ateleia glazioveana*	EF466257	trnL
Fabaceae	*Ateleia glazioveana*	EF527478	trnL
Fabaceae	*Ateleia glazioveana*	GU219999	ITS
Fabaceae	*Ateleia glazioveana*	GU220020	matK
Fabaceae	*Bauhinia cheilantha*	DQ787410	ITS
Fabaceae	*Bauhinia forficata*	FJ801053	trnL
Fabaceae	*Bauhinia forficata*	FJ801094	trnL
Fabaceae	*Bauhinia forficata*	FJ801113	trnL
Fabaceae	*Bauhinia forficata*	JN881365	matK
Fabaceae	*Bauhinia pentandra*	FJ801067	trnL
Fabaceae	*Bauhinia pentandra*	FJ801106	trnL
Fabaceae	*Bauhinia pentandra*	JN881382	matK
Fabaceae	*Bauhinia rufa*	FJ801098	trnL
Fabaceae	*Bauhinia ungulata*	FJ009818	ITS
Fabaceae	*Bauhinia ungulata*	FJ009873	trnL
Fabaceae	*Bauhinia ungulata*	FJ801073	trnL
Fabaceae	*Bauhinia ungulata*	FJ801110	trnL
Fabaceae	*Bauhinia ungulata*	HG963854	psbA-trnH
Fabaceae	*Bauhinia ungulata*	JN881404	matK
Fabaceae	*Bauhinia ungulata*	JQ587516	matK
Fabaceae	*Bauhinia ungulata*	JQ587517	matK
Fabaceae	*Bauhinia ungulata*	JQ591585	rbcL
Fabaceae	*Bauhinia ungulata*	JQ591586	rbcL
Fabaceae	*Bauhinia ungulata*	JQ591587	rbcL
Fabaceae	*Bauhinia ungulata*	JQ591588	rbcL

Family	Species	Accession	Barcode
Fabaceae	*Bauhinia ungulata*	JQ591589	rbcL
Fabaceae	*Bauhinia ungulata*	JQ591590	rbcL
Fabaceae	*Bowdichia virgilioides*	AF309486	trnL
Fabaceae	*Bowdichia virgilioides*	AY386936	matK
Fabaceae	*Bowdichia virgilioides*	AY386937	matK
Fabaceae	*Bowdichia virgilioides*	EF457709	ITS
Fabaceae	*Bowdichia virgilioides*	JX124393	matK
Fabaceae	*Bowdichia virgilioides*	JX124429	trnL
Fabaceae	*Bowdichia virgilioides*	JX124430	trnL
Fabaceae	*Bowdichia virgilioides*	JX124475	ITS
Fabaceae	*Bowdichia virgilioides*	JX124476	ITS
Fabaceae	*Caesalpinia pulcherrima*	AF430733	trnL
Fabaceae	*Caesalpinia pulcherrima*	EU361774	trnL
Fabaceae	*Caesalpinia pulcherrima*	EU361906	matK
Fabaceae	*Caesalpinia pulcherrima*	HG963814	psbA-trnH
Fabaceae	*Caesalpinia pulcherrima*	JX856420	ITS
Fabaceae	*Caesalpinia pulcherrima*	JX856553	ITS
Fabaceae	*Caesalpinia pulcherrima*	JX856664	rbcL
Fabaceae	*Caesalpinia pulcherrima*	KF379227	ITS
Fabaceae	*Caesalpinia pulcherrima*	U74190	rbcL
Fabaceae	*Caesalpinia pulcherrima*	Z70153	rbcL
Fabaceae	*Calliandra brevipes*	JX870671	ITS
Fabaceae	*Calliandra brevipes*	JX870802	trnL
Fabaceae	*Calliandra foliolosa*	EF638181	ITS
Fabaceae	*Calliandra tweedii*	DQ344583	trnL
Fabaceae	*Calliandra tweedii*	DQ344617	trnL
Fabaceae	*Calliandra tweedii*	JX870752	ITS
Fabaceae	*Centrolobium robustum*	EU401414	matK
Fabaceae	*Centrolobium robustum*	EU401425	trnL
Fabaceae	*Centrolobium robustum*	EU401438	ITS
Fabaceae	*Centrolobium tomentosum*	EU401417	matK
Fabaceae	*Centrolobium tomentosum*	EU401418	matK
Fabaceae	*Centrolobium tomentosum*	EU401427	trnL
Fabaceae	*Centrolobium tomentosum*	EU401441	ITS
Fabaceae	*Chloroleucon tenuiflorum*	EF638184	ITS

**Family**	**Species**	**Accession**	**Barcode**
Fabaceae	*Chloroleucon tenuiflorum*	KF921691	ITS
Fabaceae	*Chloroleucon tenuiflorum*	KF921692	ITS
Fabaceae	*Chloroleucon tenuiflorum*	KF921769	trnL
Fabaceae	*Chloroleucon tenuiflorum*	KF921842	psbA-trnH
Fabaceae	*Chloroleucon tenuiflorum*	KF921843	psbA-trnH
Fabaceae	*Chloroleucon tortum*	KF921693	ITS
Fabaceae	*Chloroleucon tortum*	KF921694	ITS
Fabaceae	*Chloroleucon tortum*	KF921770	trnL
Fabaceae	*Chloroleucon tortum*	KF921771	trnL
Fabaceae	*Chloroleucon tortum*	KF921844	psbA-trnH
Fabaceae	*Chloroleucon tortum*	KF921845	psbA-trnH
Fabaceae	*Copaifera langsdorffii*	AY958464	trnL
Fabaceae	*Copaifera trapezifolia*	AY958463	trnL
Fabaceae	*Copaifera trapezifolia*	AY958496	trnL
Fabaceae	*Cyclolobium brasiliense*	AF287637	ITS
Fabaceae	*Cyclolobium brasiliense*	GQ246151	matK
Fabaceae	*Cyclolobium brasiliense*	GQ246152	matK
Fabaceae	*Cyclolobium brasiliense*	KJ028452	matK
Fabaceae	*Cyclolobium brasiliense*	KJ028461	ITS
Fabaceae	*Dahlstedtia pentaphylla*	KP230716	matK
Fabaceae	*Dahlstedtia pentaphylla*	KP230717	matK
Fabaceae	*Dahlstedtia pinnata*	AF467042	ITS
Fabaceae	*Dahlstedtia pinnata*	KJ411649	ITS
Fabaceae	*Dahlstedtia pinnata*	KP230718	matK
Fabaceae	*Dalbergia brasiliensis*	EF451076	ITS
Fabaceae	*Dalbergia brasiliensis*	EF451115	trnL
Fabaceae	*Dalbergia foliolosa*	AF189022	ITS
Fabaceae	*Dalbergia foliolosa*	EF451067	ITS
Fabaceae	*Dalbergia foliolosa*	EF451106	trnL
Fabaceae	*Dalbergia frutescens*	AB828631	ITS
Fabaceae	*Dalbergia frutescens*	AB828632	ITS
Fabaceae	*Dalbergia frutescens*	EF451078	ITS
Fabaceae	*Dalbergia frutescens*	EF451117	trnL
Fabaceae	*Dalbergia miscolobium*	AB828666	ITS
Fabaceae	*Dalbergia miscolobium*	DQ787405	ITS

**Family**	**Species**	**Accession**	**Barcode**
Fabaceae	*Dalbergia miscolobium*	EF451069	ITS
Fabaceae	*Dalbergia miscolobium*	EF451070	ITS
Fabaceae	*Dalbergia miscolobium*	EF451108	trnL
Fabaceae	*Dalbergia miscolobium*	EF451109	trnL
Fabaceae	*Dalbergia miscolobium*	JQ582850	ITS
Fabaceae	*Dalbergia miscolobium*	JQ582851	ITS
Fabaceae	*Dalbergia miscolobium*	JQ582852	ITS
Fabaceae	*Dalbergia miscolobium*	JQ582853	ITS
Fabaceae	*Dalbergia miscolobium*	JQ582854	ITS
Fabaceae	*Dalbergia miscolobium*	JQ582855	ITS
Fabaceae	*Dalbergia miscolobium*	JQ582856	ITS
Fabaceae	*Dalbergia miscolobium*	JQ582857	ITS
Fabaceae	*Dalbergia miscolobium*	JQ582858	ITS
Fabaceae	*Dalbergia miscolobium*	JQ582859	ITS
Fabaceae	*Dalbergia miscolobium*	JQ582860	ITS
Fabaceae	*Dalbergia miscolobium*	JQ582861	ITS
Fabaceae	*Dalbergia miscolobium*	JQ582862	ITS
Fabaceae	*Dalbergia miscolobium*	JQ582863	ITS
Fabaceae	*Dalbergia miscolobium*	JQ582864	ITS
Fabaceae	*Dalbergia miscolobium*	JQ582865	ITS
Fabaceae	*Dalbergia miscolobium*	JQ582866	ITS
Fabaceae	*Dalbergia miscolobium*	JQ582867	ITS
Fabaceae	*Dalbergia miscolobium*	JQ582868	ITS
Fabaceae	*Dalbergia miscolobium*	JQ582869	ITS
Fabaceae	*Dalbergia miscolobium*	JQ582870	ITS
Fabaceae	*Dalbergia miscolobium*	JQ582871	ITS
Fabaceae	*Dalbergia miscolobium*	JQ582872	ITS
Fabaceae	*Dalbergia miscolobium*	JQ582873	ITS
Fabaceae	*Dalbergia miscolobium*	JQ582874	ITS
Fabaceae	*Dalbergia miscolobium*	JQ582875	ITS
Fabaceae	*Dalbergia miscolobium*	JQ582876	ITS
Fabaceae	*Dalbergia miscolobium*	JQ612719	trnL
Fabaceae	*Dalbergia miscolobium*	JQ612720	trnL
Fabaceae	*Dalbergia miscolobium*	JQ612721	trnL
Fabaceae	*Dalbergia miscolobium*	JQ612722	trnL

Family	Species	Accession	Barcode
Fabaceae	*Dalbergia miscolobium*	JQ612723	trnL
Fabaceae	*Dalbergia miscolobium*	JQ612724	trnL
Fabaceae	*Dalbergia miscolobium*	JQ612725	trnL
Fabaceae	*Dalbergia miscolobium*	JQ612726	trnL
Fabaceae	*Dalbergia miscolobium*	JQ612727	trnL
Fabaceae	*Dalbergia miscolobium*	JQ612728	trnL
Fabaceae	*Dalbergia miscolobium*	JQ612729	trnL
Fabaceae	*Dalbergia miscolobium*	JQ612730	trnL
Fabaceae	*Dalbergia miscolobium*	JX850050	matK
Fabaceae	*Dalbergia miscolobium*	KM510273	rbcL
Fabaceae	*Dalbergia miscolobium*	KM521312	matK
Fabaceae	*Dalbergia miscolobium*	KM521383	ITS
Fabaceae	*Dalbergia nigra*	EF451074	ITS
Fabaceae	*Dalbergia nigra*	EF451075	ITS
Fabaceae	*Dalbergia nigra*	EF451113	trnL
Fabaceae	*Dalbergia nigra*	EF451114	trnL
Fabaceae	*Dalbergia nigra*	GU816025	trnL
Fabaceae	*Dalbergia nigra*	GU816026	trnL
Fabaceae	*Dalbergia nigra*	GU816027	trnL
Fabaceae	*Dalbergia nigra*	GU816028	trnL
Fabaceae	*Dalbergia villosa*	EF451068	ITS
Fabaceae	*Dalbergia villosa*	EF451107	trnL
Fabaceae	*Dimorphandra mollis*	AF309480	trnL
Fabaceae	*Dimorphandra mollis*	JX850052	matK
Fabaceae	*Dipteryx alata*	AF187090	ITS
Fabaceae	*Dipteryx alata*	AF208896	trnL
Fabaceae	*Dipteryx alata*	AF272092	matK
Fabaceae	*Dipteryx alata*	AF272093	matK
Fabaceae	*Dipteryx alata*	AH009911	matK
Fabaceae	*Dipteryx alata*	AY553717	matK
Fabaceae	*Dipteryx alata*	JF491250	ITS
Fabaceae	*Dipteryx alata*	JF491265	matK
Fabaceae	*Dipteryx alata*	JF491278	trnL
Fabaceae	*Dipteryx alata*	KJ813617	ITS
Fabaceae	*Dipteryx alata*	KJ813652	trnL

**Family**	**Species**	**Accession**	**Barcode**
Fabaceae	*Diptychandra aurantiaca*	AF309478	trnL
Fabaceae	*Diptychandra aurantiaca*	AF430774	trnL
Fabaceae	*Diptychandra aurantiaca*	EU361799	trnL
Fabaceae	*Diptychandra aurantiaca*	EU361935	matK
Fabaceae	*Enterolobium contortisiliquum*	AF274124	matK
Fabaceae	*Enterolobium contortisiliquum*	AF522952	trnL
Fabaceae	*Enterolobium contortisiliquum*	AF524971	psbA-trnH
Fabaceae	*Enterolobium contortisiliquum*	EF638190	ITS
Fabaceae	*Enterolobium contortisiliquum*	JX495708	matK
Fabaceae	*Enterolobium contortisiliquum*	JX571823	rbcL
Fabaceae	*Enterolobium gummiferum*	KF921696	ITS
Fabaceae	*Enterolobium gummiferum*	KF921773	trnL
Fabaceae	*Enterolobium gummiferum*	KF921848	psbA-trnH
Fabaceae	*Erythrina crista-galli*	AB441760	ITS
Fabaceae	*Erythrina crista-galli*	AY386869	matK
Fabaceae	*Erythrina crista-galli*	FN825780	ITS
Fabaceae	*Erythrina crista-galli*	KJ419280	ITS
Fabaceae	*Erythrina crista-galli*	KJ419281	ITS
Fabaceae	*Erythrina crista-galli*	Z70170	rbcL
Fabaceae	*Exostyles godoyensis*	AY438092	trnL
Fabaceae	*Exostyles godoyensis*	JX152589	matK
Fabaceae	*Exostyles godoyensis*	JX152662	ITS
Fabaceae	*Exostyles godoyensis*	JX187640	trnL
Fabaceae	*Exostyles godoyensis*	JX187716	trnL
Fabaceae	*Exostyles godoyensis*	JX187760	psbA-trnH
Fabaceae	*Exostyles godoyensis*	KC595366	ITS
Fabaceae	*Exostyles venusta*	AF309838	trnL
Fabaceae	*Exostyles venusta*	AF524882	trnL
Fabaceae	*Exostyles venusta*	JX152590	matK
Fabaceae	*Exostyles venusta*	JX152591	matK
Fabaceae	*Exostyles venusta*	JX152663	ITS
Fabaceae	*Exostyles venusta*	JX152664	ITS
Fabaceae	*Exostyles venusta*	JX187641	trnL
Fabaceae	*Exostyles venusta*	JX187642	trnL
Fabaceae	*Exostyles venusta*	JX187717	trnL

Family	Species	Accession	Barcode
Fabaceae	*Exostyles venusta*	JX187718	trnL
Fabaceae	*Exostyles venusta*	JX187761	psbA-trnH
Fabaceae	*Exostyles venusta*	JX187762	psbA-trnH
Fabaceae	*Holocalyx balansae*	AF310999	trnL
Fabaceae	*Holocalyx balansae*	AF524881	trnL
Fabaceae	*Holocalyx balansae*	AY553714	matK
Fabaceae	*Holocalyx balansae*	EF457718	ITS
Fabaceae	*Holocalyx balansae*	EF466269	trnL
Fabaceae	*Holocalyx balansae*	EF527490	trnL
Fabaceae	*Holocalyx balansae*	JX152593	matK
Fabaceae	*Holocalyx balansae*	JX152666	ITS
Fabaceae	*Holocalyx balansae*	JX187644	trnL
Fabaceae	*Holocalyx balansae*	JX187720	trnL
Fabaceae	*Holocalyx balansae*	JX187764	psbA-trnH
Fabaceae	*Holocalyx balansae*	U74244	rbcL
Fabaceae	*Hymenaea courbaril*	AF365160	trnL
Fabaceae	*Hymenaea courbaril*	AY386906	matK
Fabaceae	*Hymenaea courbaril*	AY955800	ITS
Fabaceae	*Hymenaea courbaril*	AY958514	trnL
Fabaceae	*Hymenaea courbaril*	EU361972	matK
Fabaceae	*Hymenaea courbaril*	FJ009817	ITS
Fabaceae	*Hymenaea courbaril*	FJ009872	trnL
Fabaceae	*Hymenaea courbaril*	FJ037908	matK
Fabaceae	*Hymenaea courbaril*	FJ038273	rpoB
Fabaceae	*Hymenaea courbaril*	FJ038550	rpoC1
Fabaceae	*Hymenaea courbaril*	HG963810	psbA-trnH
Fabaceae	*Hymenaea courbaril*	JQ587656	matK
Fabaceae	*Hymenaea courbaril*	JQ587657	matK
Fabaceae	*Hymenaea courbaril*	JQ587658	matK
Fabaceae	*Hymenaea courbaril*	JQ587659	matK
Fabaceae	*Hymenaea courbaril*	JQ587660	matK
Fabaceae	*Hymenaea courbaril*	JQ587661	matK
Fabaceae	*Hymenaea courbaril*	JQ591776	rbcL
Fabaceae	*Hymenaea courbaril*	JQ591777	rbcL
Fabaceae	*Hymenaea courbaril*	JQ591778	rbcL

Family	Species	Accession	Barcode
Fabaceae	*Hymenaea courbaril*	JQ591779	rbcL
Fabaceae	*Hymenaea courbaril*	JQ591780	rbcL
Fabaceae	*Hymenaea courbaril*	JQ591781	rbcL
Fabaceae	*Hymenaea courbaril*	JQ625969	rbcL
Fabaceae	*Hymenaea courbaril*	JQ626412	matK
Fabaceae	*Hymenaea courbaril*	KJ082365	rbcL
Fabaceae	*Hymenaea stigonocarpa*	AY958475	trnL
Fabaceae	*Hymenaea stigonocarpa*	AY958515	trnL
Fabaceae	*Hymenolobium janeirense*	JX275934	trnL
Fabaceae	*Hymenolobium janeirense*	JX275935	trnL
Fabaceae	*Hymenolobium janeirense*	JX295904	matK
Fabaceae	*Inga barbata*	AM919889	rpoC1
Fabaceae	*Inga barbata*	GU012922	ITS
Fabaceae	*Inga barbata*	GU012923	ITS
Fabaceae	*Inga barbata*	GU012924	ITS
Fabaceae	*Inga barbata*	GU012925	ITS
Fabaceae	*Inga capitata*	AM919891	rpoC1
Fabaceae	*Inga capitata*	GQ118719	trnL
Fabaceae	*Inga capitata*	GQ118797	rpoC1
Fabaceae	*Inga capitata*	GU012963	ITS
Fabaceae	*Inga capitata*	GU012964	ITS
Fabaceae	*Inga capitata*	GU012965	ITS
Fabaceae	*Inga capitata*	GU012966	ITS
Fabaceae	*Inga capitata*	GU012967	ITS
Fabaceae	*Inga capitata*	GU012968	ITS
Fabaceae	*Inga capitata*	GU012969	ITS
Fabaceae	*Inga capitata*	GU012970	ITS
Fabaceae	*Inga capitata*	GU012971	ITS
Fabaceae	*Inga capitata*	GU012972	ITS
Fabaceae	*Inga capitata*	GU012973	ITS
Fabaceae	*Inga capitata*	GU012974	ITS
Fabaceae	*Inga capitata*	GU012975	ITS
Fabaceae	*Inga capitata*	GU012976	ITS
Fabaceae	*Inga capitata*	GU012977	ITS
Fabaceae	*Inga capitata*	GU012978	ITS

**Family**	**Species**	**Accession**	**Barcode**
Fabaceae	*Inga capitata*	GU012979	ITS
Fabaceae	*Inga capitata*	GU012980	ITS
Fabaceae	*Inga capitata*	GU012981	ITS
Fabaceae	*Inga capitata*	GU012982	ITS
Fabaceae	*Inga capitata*	GU012983	ITS
Fabaceae	*Inga capitata*	GU012984	ITS
Fabaceae	*Inga capitata*	GU012985	ITS
Fabaceae	*Inga capitata*	GU012986	ITS
Fabaceae	*Inga capitata*	GU012987	ITS
Fabaceae	*Inga capitata*	GU012988	ITS
Fabaceae	*Inga capitata*	GU012989	ITS
Fabaceae	*Inga capitata*	JQ625753	rbcL
Fabaceae	*Inga edulis*	AF522957	trnL
Fabaceae	*Inga edulis*	AF523078	matK
Fabaceae	*Inga edulis*	AF524976	psbA-trnH
Fabaceae	*Inga edulis*	AM919897	rpoC1
Fabaceae	*Inga edulis*	AM919898	rpoC1
Fabaceae	*Inga edulis*	AM920095	rpoB
Fabaceae	*Inga edulis*	AM920096	rpoB
Fabaceae	*Inga edulis*	AM920181	matK
Fabaceae	*Inga edulis*	AM920183	matK
Fabaceae	*Inga edulis*	AM920256	rbcL
Fabaceae	*Inga edulis*	AM922019	psbA-trnH
Fabaceae	*Inga edulis*	AM922020	psbA-trnH
Fabaceae	*Inga edulis*	AM922021	psbA-trnH
Fabaceae	*Inga edulis*	DQ499104	ITS
Fabaceae	*Inga edulis*	FJ173504	atpF-atpH
Fabaceae	*Inga edulis*	FJ173636	psbK-psbI
Fabaceae	*Inga edulis*	FJ173637	psbK-psbI
Fabaceae	*Inga edulis*	FJ173737	rbcL
Fabaceae	*Inga edulis*	GQ118722	trnL
Fabaceae	*Inga edulis*	GQ118800	rpoC1
Fabaceae	*Inga edulis*	GQ118870	psbA-trnH
Fabaceae	*Inga edulis*	GU013045	ITS
Fabaceae	*Inga edulis*	GU013046	ITS

Family	Species	Accession	Barcode
Fabaceae	*Inga edulis*	GU013047	ITS
Fabaceae	*Inga edulis*	GU013048	ITS
Fabaceae	*Inga edulis*	GU013049	ITS
Fabaceae	*Inga edulis*	GU013050	ITS
Fabaceae	*Inga edulis*	GU013051	ITS
Fabaceae	*Inga edulis*	GU013052	ITS
Fabaceae	*Inga edulis*	GU013053	ITS
Fabaceae	*Inga edulis*	GU013054	ITS
Fabaceae	*Inga edulis*	GU013055	ITS
Fabaceae	*Inga edulis*	GU013056	ITS
Fabaceae	*Inga edulis*	GU013057	ITS
Fabaceae	*Inga edulis*	GU013058	ITS
Fabaceae	*Inga edulis*	GU013059	ITS
Fabaceae	*Inga edulis*	GU013060	ITS
Fabaceae	*Inga edulis*	GU013061	ITS
Fabaceae	*Inga edulis*	GU013062	ITS
Fabaceae	*Inga edulis*	GU013063	ITS
Fabaceae	*Inga edulis*	GU013064	ITS
Fabaceae	*Inga edulis*	GU013065	ITS
Fabaceae	*Inga edulis*	GU013066	ITS
Fabaceae	*Inga edulis*	HM020738	matK
Fabaceae	*Inga edulis*	HM020836	trnL
Fabaceae	*Inga edulis*	JQ591797	rbcL
Fabaceae	*Inga edulis*	JQ591798	rbcL
Fabaceae	*Inga edulis*	JQ591799	rbcL
Fabaceae	*Inga edulis*	JX870764	ITS
Fabaceae	*Inga edulis*	JX870880	trnL
Fabaceae	*Inga edulis*	KF921853	psbA-trnH
Fabaceae	*Inga edulis*	KT722474	ITS
Fabaceae	*Inga edulis*	KT722508	psbA-trnH
Fabaceae	*Inga edulis*	KT722556	trnL
Fabaceae	*Inga ingoides*	GU013078	ITS
Fabaceae	*Inga ingoides*	GU013079	ITS
Fabaceae	*Inga ingoides*	GU013080	ITS
Fabaceae	*Inga laurina*	AM919899	rpoC1

**Family**	**Species**	**Accession**	**Barcode**
Fabaceae	*Inga laurina*	AM919947	rpoC1
Fabaceae	*Inga laurina*	AM919949	rpoC1
Fabaceae	*Inga laurina*	AM922044	psbA-trnH
Fabaceae	*Inga laurina*	AM922045	psbA-trnH
Fabaceae	*Inga laurina*	AM922046	psbA-trnH
Fabaceae	*Inga laurina*	GQ118732	trnL
Fabaceae	*Inga laurina*	GQ118811	rpoC1
Fabaceae	*Inga laurina*	GQ118877	psbA-trnH
Fabaceae	*Inga laurina*	GQ981770	rbcL
Fabaceae	*Inga laurina*	GQ982132	matK
Fabaceae	*Inga laurina*	GQ982254	psbA-trnH
Fabaceae	*Inga laurina*	GU013082	ITS
Fabaceae	*Inga laurina*	GU013083	ITS
Fabaceae	*Inga laurina*	GU013084	ITS
Fabaceae	*Inga laurina*	GU013085	ITS
Fabaceae	*Inga laurina*	GU013086	ITS
Fabaceae	*Inga laurina*	GU013087	ITS
Fabaceae	*Inga laurina*	GU013088	ITS
Fabaceae	*Inga laurina*	GU013089	ITS
Fabaceae	*Inga laurina*	GU013090	ITS
Fabaceae	*Inga laurina*	GU013091	ITS
Fabaceae	*Inga laurina*	GU013092	ITS
Fabaceae	*Inga laurina*	GU013093	ITS
Fabaceae	*Inga laurina*	GU013094	ITS
Fabaceae	*Inga laurina*	GU013095	ITS
Fabaceae	*Inga laurina*	GU013096	ITS
Fabaceae	*Inga laurina*	GU013097	ITS
Fabaceae	*Inga laurina*	GU013098	ITS
Fabaceae	*Inga laurina*	GU013099	ITS
Fabaceae	*Inga laurina*	GU013100	ITS
Fabaceae	*Inga laurina*	GU013101	ITS
Fabaceae	*Inga laurina*	GU013102	ITS
Fabaceae	*Inga laurina*	GU013103	ITS
Fabaceae	*Inga laurina*	GU013104	ITS
Fabaceae	*Inga laurina*	GU013105	ITS

Family	Species	Accession	Barcode
Fabaceae	*Inga laurina*	GU013106	ITS
Fabaceae	*Inga laurina*	GU013107	ITS
Fabaceae	*Inga laurina*	GU013108	ITS
Fabaceae	*Inga laurina*	GU013109	ITS
Fabaceae	*Inga laurina*	HM446701	matK
Fabaceae	*Inga laurina*	HM446814	rbcL
Fabaceae	*Inga laurina*	HM446946	psbA-trnH
Fabaceae	*Inga laurina*	KT722476	ITS
Fabaceae	*Inga laurina*	KT722511	psbA-trnH
Fabaceae	*Inga laurina*	KT722523	rpoC1
Fabaceae	*Inga laurina*	KT722559	trnL
Fabaceae	*Inga marginata*	AM919915	rpoC1
Fabaceae	*Inga marginata*	AM919916	rpoC1
Fabaceae	*Inga marginata*	AM919917	rpoC1
Fabaceae	*Inga marginata*	AM919918	rpoC1
Fabaceae	*Inga marginata*	AM919919	rpoC1
Fabaceae	*Inga marginata*	AM919920	rpoC1
Fabaceae	*Inga marginata*	AM919921	rpoC1
Fabaceae	*Inga marginata*	AM920104	rpoB
Fabaceae	*Inga marginata*	AM920105	rpoB
Fabaceae	*Inga marginata*	AM920106	rpoB
Fabaceae	*Inga marginata*	AM920107	rpoB
Fabaceae	*Inga marginata*	AM920180	matK
Fabaceae	*Inga marginata*	AM920186	matK
Fabaceae	*Inga marginata*	AM920188	matK
Fabaceae	*Inga marginata*	AM920215	matK
Fabaceae	*Inga marginata*	AM922030	psbA-trnH
Fabaceae	*Inga marginata*	AM922031	psbA-trnH
Fabaceae	*Inga marginata*	AM922032	psbA-trnH
Fabaceae	*Inga marginata*	AM922033	psbA-trnH
Fabaceae	*Inga marginata*	EF638197	ITS
Fabaceae	*Inga marginata*	FJ173642	psbK-psbI
Fabaceae	*Inga marginata*	FJ173643	psbK-psbI
Fabaceae	*Inga marginata*	FJ173644	psbK-psbI
Fabaceae	*Inga marginata*	FJ173741	rbcL

**Family**	**Species**	**Accession**	**Barcode**
Fabaceae	*Inga marginata*	FJ173742	rbcL
Fabaceae	*Inga marginata*	FJ173743	rbcL
Fabaceae	*Inga marginata*	FJ173744	rbcL
Fabaceae	*Inga marginata*	GQ118728	trnL
Fabaceae	*Inga marginata*	GQ118806	rpoC1
Fabaceae	*Inga marginata*	GQ118873	psbA-trnH
Fabaceae	*Inga marginata*	GQ981771	rbcL
Fabaceae	*Inga marginata*	GQ982018	matK
Fabaceae	*Inga marginata*	GQ982255	psbA-trnH
Fabaceae	*Inga marginata*	GU013143	ITS
Fabaceae	*Inga marginata*	GU013144	ITS
Fabaceae	*Inga marginata*	GU013145	ITS
Fabaceae	*Inga marginata*	GU013146	ITS
Fabaceae	*Inga marginata*	GU013147	ITS
Fabaceae	*Inga marginata*	GU013148	ITS
Fabaceae	*Inga marginata*	GU013149	ITS
Fabaceae	*Inga marginata*	GU013150	ITS
Fabaceae	*Inga marginata*	GU013151	ITS
Fabaceae	*Inga marginata*	GU013152	ITS
Fabaceae	*Inga marginata*	GU013153	ITS
Fabaceae	*Inga marginata*	GU013154	ITS
Fabaceae	*Inga marginata*	GU013155	ITS
Fabaceae	*Inga marginata*	GU013156	ITS
Fabaceae	*Inga marginata*	GU013157	ITS
Fabaceae	*Inga marginata*	GU013158	ITS
Fabaceae	*Inga marginata*	GU013159	ITS
Fabaceae	*Inga marginata*	GU013160	ITS
Fabaceae	*Inga marginata*	GU013161	ITS
Fabaceae	*Inga marginata*	GU013162	ITS
Fabaceae	*Inga marginata*	GU013163	ITS
Fabaceae	*Inga marginata*	GU013164	ITS
Fabaceae	*Inga marginata*	GU013165	ITS
Fabaceae	*Inga marginata*	GU013166	ITS
Fabaceae	*Inga marginata*	GU013167	ITS
Fabaceae	*Inga marginata*	GU013168	ITS

**Family**	**Species**	**Accession**	**Barcode**
Fabaceae	*Inga marginata*	GU013169	ITS
Fabaceae	*Inga marginata*	GU013170	ITS
Fabaceae	*Inga marginata*	GU013171	ITS
Fabaceae	*Inga marginata*	GU013172	ITS
Fabaceae	*Inga marginata*	GU013173	ITS
Fabaceae	*Inga marginata*	KF421057	psbA-trnH
Fabaceae	*Inga marginata*	KF555397	matK
Fabaceae	*Inga marginata*	KF561916	rbcL
Fabaceae	*Inga marginata*	KT722478	ITS
Fabaceae	*Inga marginata*	KT722514	psbA-trnH
Fabaceae	*Inga marginata*	KT722562	trnL
Fabaceae	*Inga sellowiana*	AM919968	rpoC1
Fabaceae	*Inga striata*	AM919975	rpoC1
Fabaceae	*Inga tenuis*	AM919967	rpoC1
Fabaceae	*Inga tenuis*	AM920125	rpoB
Fabaceae	*Inga tenuis*	AM920199	matK
Fabaceae	*Inga tenuis*	AM922051	psbA-trnH
Fabaceae	*Inga tenuis*	FJ173657	psbK-psbI
Fabaceae	*Inga tenuis*	FJ173753	rbcL
Fabaceae	*Inga thibaudiana*	AM919977	rpoC1
Fabaceae	*Inga thibaudiana*	AM919978	rpoC1
Fabaceae	*Inga thibaudiana*	GQ118743	trnL
Fabaceae	*Inga thibaudiana*	GQ118821	rpoC1
Fabaceae	*Inga thibaudiana*	GQ118886	psbA-trnH
Fabaceae	*Inga thibaudiana*	GQ981779	rbcL
Fabaceae	*Inga thibaudiana*	GQ982025	matK
Fabaceae	*Inga thibaudiana*	GQ982263	psbA-trnH
Fabaceae	*Inga thibaudiana*	GU013329	ITS
Fabaceae	*Inga thibaudiana*	GU013330	ITS
Fabaceae	*Inga thibaudiana*	GU013331	ITS
Fabaceae	*Inga thibaudiana*	GU013332	ITS
Fabaceae	*Inga thibaudiana*	GU013333	ITS
Fabaceae	*Inga thibaudiana*	GU013334	ITS
Fabaceae	*Inga thibaudiana*	GU013335	ITS
Fabaceae	*Inga thibaudiana*	GU013336	ITS

Family	Species	Accession	Barcode
Fabaceae	*Inga thibaudiana*	GU013337	ITS
Fabaceae	*Inga thibaudiana*	GU013338	ITS
Fabaceae	*Inga thibaudiana*	GU013339	ITS
Fabaceae	*Inga thibaudiana*	GU013340	ITS
Fabaceae	*Inga thibaudiana*	GU013341	ITS
Fabaceae	*Inga thibaudiana*	GU013342	ITS
Fabaceae	*Inga thibaudiana*	GU013343	ITS
Fabaceae	*Inga thibaudiana*	GU013344	ITS
Fabaceae	*Inga thibaudiana*	GU013345	ITS
Fabaceae	*Inga thibaudiana*	GU013346	ITS
Fabaceae	*Inga thibaudiana*	GU013347	ITS
Fabaceae	*Inga thibaudiana*	GU013348	ITS
Fabaceae	*Inga thibaudiana*	GU013349	ITS
Fabaceae	*Inga thibaudiana*	GU013350	ITS
Fabaceae	*Inga thibaudiana*	GU013351	ITS
Fabaceae	*Inga thibaudiana*	GU013352	ITS
Fabaceae	*Inga thibaudiana*	GU013353	ITS
Fabaceae	*Inga thibaudiana*	GU013354	ITS
Fabaceae	*Inga thibaudiana*	GU013355	ITS
Fabaceae	*Inga thibaudiana*	GU013356	ITS
Fabaceae	*Inga thibaudiana*	GU013357	ITS
Fabaceae	*Inga thibaudiana*	GU013358	ITS
Fabaceae	*Inga thibaudiana*	GU013359	ITS
Fabaceae	*Inga thibaudiana*	GU013360	ITS
Fabaceae	*Inga thibaudiana*	GU013361	ITS
Fabaceae	*Inga thibaudiana*	GU013362	ITS
Fabaceae	*Inga thibaudiana*	GU013363	ITS
Fabaceae	*Inga thibaudiana*	JX870765	ITS
Fabaceae	*Inga thibaudiana*	JX870881	trnL
Fabaceae	*Inga thibaudiana*	KF921854	psbA-trnH
Fabaceae	*Inga thibaudiana*	KT722483	ITS
Fabaceae	*Inga thibaudiana*	KT722520	psbA-trnH
Fabaceae	*Inga thibaudiana*	KT722528	rpoC1
Fabaceae	*Inga thibaudiana*	KT722568	trnL
Fabaceae	*Inga vera*	AM919984	rpoC1

Family	Species	Accession	Barcode
Fabaceae	*Inga vera*	AM919985	rpoC1
Fabaceae	*Inga vera*	GQ118747	trnL
Fabaceae	*Inga vera*	GQ118825	rpoC1
Fabaceae	*Inga vera*	GQ118890	psbA-trnH
Fabaceae	*Inga vera*	GU013408	ITS
Fabaceae	*Inga vera*	HG963539	psbA-trnH
Fabaceae	*Inga vera*	HM446702	matK
Fabaceae	*Inga vera*	HM446815	rbcL
Fabaceae	*Inga vera*	HM446947	psbA-trnH
Fabaceae	*Inga vera*	JQ587686	matK
Fabaceae	*Inga vera*	JQ587687	matK
Fabaceae	*Inga vera*	JQ587688	matK
Fabaceae	*Inga vera*	JQ591826	rbcL
Fabaceae	*Inga vera*	JQ591827	rbcL
Fabaceae	*Inga vera*	JQ591828	rbcL
Fabaceae	*Leptolobium dasycarpum*	JX124408	matK
Fabaceae	*Leptolobium dasycarpum*	JX124450	trnL
Fabaceae	*Leptolobium dasycarpum*	JX124496	ITS
Fabaceae	*Leptolobium dasycarpum*	JX124504	ITS
Fabaceae	*Leptolobium dasycarpum*	JX124512	ITS
Fabaceae	*Leptolobium dasycarpum*	JX124514	ITS
Fabaceae	*Leptolobium dasycarpum*	JX124515	ITS
Fabaceae	*Leptolobium dasycarpum*	JX124516	ITS
Fabaceae	*Leptolobium dasycarpum*	JX124517	ITS
Fabaceae	*Leptolobium dasycarpum*	JX124518	ITS
Fabaceae	*Leptolobium dasycarpum*	JX124519	ITS
Fabaceae	*Leptolobium dasycarpum*	U74255	rbcL
Fabaceae	*Leptolobium elegans*	AF309826	trnL
Fabaceae	*Leptolobium elegans*	EF457703	ITS
Fabaceae	*Leptolobium elegans*	JX124410	matK
Fabaceae	*Leptolobium elegans*	JX124505	ITS
Fabaceae	*Leptolobium elegans*	JX124509	ITS
Fabaceae	*Leptolobium elegans*	JX124510	ITS
Fabaceae	*Leucaena leucocephala*	AB817392	trnL
Fabaceae	*Leucaena leucocephala*	AB817443	trnL

**Family**	**Species**	**Accession**	**Barcode**
Fabaceae	*Leucaena leucocephala*	GU135371	psbA-trnH
Fabaceae	*Leucaena leucocephala*	JF339937	ITS
Fabaceae	*Leucaena leucocephala*	JF339938	ITS
Fabaceae	*Leucaena leucocephala*	JF339960	ITS
Fabaceae	*Leucaena leucocephala*	JF339961	ITS
Fabaceae	*Leucaena leucocephala*	JF339966	ITS
Fabaceae	*Leucaena leucocephala*	JF500794	psbA-trnH
Fabaceae	*Leucaena leucocephala*	JF500799	psbA-trnH
Fabaceae	*Leucaena leucocephala*	JF500800	psbA-trnH
Fabaceae	*Leucaena leucocephala*	JF804912	trnL
Fabaceae	*Leucaena leucocephala*	JX495731	matK
Fabaceae	*Leucaena leucocephala*	JX517864	matK
Fabaceae	*Leucaena leucocephala*	JX571861	rbcL
Fabaceae	*Leucaena leucocephala*	JX572724	rbcL
Fabaceae	*Leucaena leucocephala*	JX856903	psbA-trnH
Fabaceae	*Leucaena leucocephala*	KF381139	rbcL
Fabaceae	*Leucaena leucocephala*	KJ012654	matK
Fabaceae	*Leucaena leucocephala*	KJ082381	rbcL
Fabaceae	*Leucaena leucocephala*	KJ426793	psbA-trnH
Fabaceae	*Leucaena leucocephala*	KM030000	rbcL
Fabaceae	*Leucaena leucocephala*	KM030003	trnL
Fabaceae	*Leucaena leucocephala*	KM030010	matK
Fabaceae	*Leucaena leucocephala*	KP093586	matK
Fabaceae	*Leucaena leucocephala*	KP093587	matK
Fabaceae	*Leucaena leucocephala*	KP094514	rbcL
Fabaceae	*Leucaena leucocephala*	KP094515	rbcL
Fabaceae	*Leucaena leucocephala*	KP095362	psbA-trnH
Fabaceae	*Leucaena leucocephala*	KP095363	psbA-trnH
Fabaceae	*Leucaena leucocephala*	KP096012	ITS
Fabaceae	*Leucochloron incuriale*	KF921701	ITS
Fabaceae	*Leucochloron incuriale*	KF921778	trnL
Fabaceae	*Leucochloron incuriale*	KF921857	psbA-trnH
Fabaceae	*Lonchocarpus campestris*	KC779549	matK
Fabaceae	*Lonchocarpus campestris*	KC779550	matK
Fabaceae	*Lonchocarpus campestris*	KP177929	matK

Family	Species	Accession	Barcode
Fabaceae	*Lonchocarpus latifolius*	GQ981789	rbcL
Fabaceae	*Lonchocarpus latifolius*	GQ982035	matK
Fabaceae	*Lonchocarpus latifolius*	GQ982274	psbA-trnH
Fabaceae	*Lonchocarpus latifolius*	HM446705	matK
Fabaceae	*Lonchocarpus latifolius*	HM446818	rbcL
Fabaceae	*Lonchocarpus latifolius*	HM446950	psbA-trnH
Fabaceae	*Lonchocarpus latifolius*	KJ012657	matK
Fabaceae	*Lonchocarpus latifolius*	KJ082389	rbcL
Fabaceae	*Lonchocarpus latifolius*	KJ411672	ITS
Fabaceae	*Lonchocarpus latifolius*	KJ426799	psbA-trnH
Fabaceae	*Lonchocarpus muehlbergianus*	AF467059	ITS
Fabaceae	*Lonchocarpus muehlbergianus*	JX506502	trnL
Fabaceae	*Lonchocarpus muehlbergianus*	JX506558	psbA-trnH
Fabaceae	*Lonchocarpus muehlbergianus*	JX506615	matK
Fabaceae	*Lonchocarpus subglaucescens*	AF467066	ITS
Fabaceae	*Lonchocarpus subglaucescens*	JX506501	trnL
Fabaceae	*Lonchocarpus subglaucescens*	JX506557	psbA-trnH
Fabaceae	*Lonchocarpus subglaucescens*	JX506614	matK
Fabaceae	*Lonchocarpus subglaucescens*	KJ411647	ITS
Fabaceae	*Luetzelburgia guaissara*	AF524876	trnL
Fabaceae	*Luetzelburgia guaissara*	JX152636	matK
Fabaceae	*Luetzelburgia guaissara*	JX152637	matK
Fabaceae	*Luetzelburgia guaissara*	JX152713	ITS
Fabaceae	*Luetzelburgia guaissara*	JX152714	ITS
Fabaceae	*Luetzelburgia guaissara*	JX152715	ITS
Fabaceae	*Luetzelburgia guaissara*	JX187691	trnL
Fabaceae	*Luetzelburgia guaissara*	JX187692	trnL
Fabaceae	*Luetzelburgia guaissara*	JX187693	trnL
Fabaceae	*Luetzelburgia guaissara*	JX187811	psbA-trnH
Fabaceae	*Luetzelburgia guaissara*	JX187812	psbA-trnH
Fabaceae	*Luetzelburgia guaissara*	JX187813	psbA-trnH
Fabaceae	*Luetzelburgia guaissara*	KC595477	ITS
Fabaceae	*Luetzelburgia guaissara*	KC595478	ITS
Fabaceae	*Luetzelburgia guaissara*	KC595479	ITS
Fabaceae	*Luetzelburgia guaissara*	KC595480	ITS

**Family**	**Species**	**Accession**	**Barcode**
Fabaceae	*Luetzelburgia guaissara*	KC595481	ITS
Fabaceae	*Luetzelburgia guaissara*	KC595482	ITS
Fabaceae	*Luetzelburgia guaissara*	KC595483	ITS
Fabaceae	*Luetzelburgia guaissara*	KC595484	ITS
Fabaceae	*Machaerium acutifolium*	AF208926	trnL
Fabaceae	*Machaerium acutifolium*	EF451090	ITS
Fabaceae	*Machaerium acutifolium*	EF451129	trnL
Fabaceae	*Machaerium brasiliense*	EF451134	trnL
Fabaceae	*Machaerium hirtum*	EF451081	ITS
Fabaceae	*Machaerium hirtum*	EF451120	trnL
Fabaceae	*Machaerium nyctitans*	EF451082	ITS
Fabaceae	*Machaerium nyctitans*	EF451083	ITS
Fabaceae	*Machaerium nyctitans*	EF451121	trnL
Fabaceae	*Machaerium nyctitans*	EF451122	trnL
Fabaceae	*Machaerium scleroxylon*	EF451084	ITS
Fabaceae	*Machaerium scleroxylon*	EF451085	ITS
Fabaceae	*Machaerium scleroxylon*	EF451123	trnL
Fabaceae	*Machaerium scleroxylon*	EF451124	trnL
Fabaceae	*Machaerium stipitatum*	EF451091	ITS
Fabaceae	*Machaerium stipitatum*	EF451092	ITS
Fabaceae	*Machaerium stipitatum*	EF451130	trnL
Fabaceae	*Machaerium stipitatum*	EF451131	trnL
Fabaceae	*Machaerium villosum*	EF451093	ITS
Fabaceae	*Machaerium villosum*	EF451094	ITS
Fabaceae	*Machaerium villosum*	EF451132	trnL
Fabaceae	*Machaerium villosum*	EF451133	trnL
Fabaceae	*Melanoxylon brauna*	AY899700	trnL
Fabaceae	*Melanoxylon brauna*	AY904388	rbcL
Fabaceae	*Melanoxylon brauna*	EU361822	trnL
Fabaceae	*Melanoxylon brauna*	EU362000	matK
Fabaceae	*Mimosa caesalpiniifolia*	JX850058	matK
Fabaceae	*Mimosa pilulifera*	DQ344573	trnL
Fabaceae	*Mimosa pilulifera*	DQ344607	trnL
Fabaceae	*Mimosa scabrella*	KF420980	ITS
Fabaceae	*Mimosa scabrella*	KF420981	ITS

Family	Species	Accession	Barcode
Fabaceae	*Mimosa scabrella*	KF420982	ITS
Fabaceae	*Mimosa scabrella*	KF420983	ITS
Fabaceae	*Mimosa scabrella*	KF421058	psbA-trnH
Fabaceae	*Mimosa scabrella*	KF421059	psbA-trnH
Fabaceae	*Mimosa scabrella*	KF421060	psbA-trnH
Fabaceae	*Mimosa scabrella*	KF421061	psbA-trnH
Fabaceae	*Mimosa scabrella*	KF421062	psbA-trnH
Fabaceae	*Mimosa scabrella*	KF421063	psbA-trnH
Fabaceae	*Mimosa scabrella*	KF555411	matK
Fabaceae	*Mimosa scabrella*	KF555412	matK
Fabaceae	*Mimosa scabrella*	KF555413	matK
Fabaceae	*Mimosa scabrella*	KF555415	matK
Fabaceae	*Mimosa scabrella*	KF561930	rbcL
Fabaceae	*Mimosa scabrella*	KF561931	rbcL
Fabaceae	*Mimosa scabrella*	KF561932	rbcL
Fabaceae	*Mimosa tenuiflora*	AF274120	matK
Fabaceae	*Mimosa tenuiflora*	AF522943	trnL
Fabaceae	*Mimosa tenuiflora*	AF524963	psbA-trnH
Fabaceae	*Mimosa tenuiflora*	JX850057	matK
Fabaceae	*Myrocarpus frondosus*	AF311002	trnL
Fabaceae	*Myrocarpus frondosus*	AY386925	matK
Fabaceae	*Myroxylon peruiferum*	JX275949	trnL
Fabaceae	*Myroxylon peruiferum*	JX295911	matK
Fabaceae	*Ormosia arborea*	JX295939	matK
Fabaceae	*Ormosia fastigiata*	JX275915	trnL
Fabaceae	*Ormosia fastigiata*	JX295885	matK
Fabaceae	*Ormosia fastigiata*	JX295940	matK
Fabaceae	*Ormosia fastigiata*	JX295941	matK
Fabaceae	*Ormosia fastigiata*	JX295942	matK
Fabaceae	*Ormosia minor*	JX124470	trnL
Fabaceae	*Ormosia minor*	JX124471	trnL
Fabaceae	*Ormosia minor*	JX275920	trnL
Fabaceae	*Ormosia minor*	JX275921	trnL
Fabaceae	*Ormosia minor*	JX295945	matK
Fabaceae	*Parapiptadenia rigida*	AF278505	trnL

**Family**	**Species**	**Accession**	**Barcode**
Fabaceae	*Parapiptadenia rigida*	AF521849	matK
Fabaceae	*Parapiptadenia rigida*	DQ784652	trnL
Fabaceae	*Parkinsonia aculeata*	AF365072	trnL
Fabaceae	*Parkinsonia aculeata*	AF430784	trnL
Fabaceae	*Parkinsonia aculeata*	AY386917	matK
Fabaceae	*Parkinsonia aculeata*	AY899715	trnL
Fabaceae	*Parkinsonia aculeata*	AY904403	rbcL
Fabaceae	*Parkinsonia aculeata*	EF101287	rbcL
Fabaceae	*Parkinsonia aculeata*	EF101288	rbcL
Fabaceae	*Parkinsonia aculeata*	EF101289	rbcL
Fabaceae	*Parkinsonia aculeata*	EF101290	rbcL
Fabaceae	*Parkinsonia aculeata*	EF101291	rbcL
Fabaceae	*Parkinsonia aculeata*	EF101292	rbcL
Fabaceae	*Parkinsonia aculeata*	EF101293	trnL
Fabaceae	*Parkinsonia aculeata*	EF101294	trnL
Fabaceae	*Parkinsonia aculeata*	EF101295	trnL
Fabaceae	*Parkinsonia aculeata*	EF101296	trnL
Fabaceae	*Parkinsonia aculeata*	EF101297	trnL
Fabaceae	*Parkinsonia aculeata*	EF101298	trnL
Fabaceae	*Parkinsonia aculeata*	EU362019	matK
Fabaceae	*Parkinsonia aculeata*	JX495738	matK
Fabaceae	*Parkinsonia aculeata*	JX571869	rbcL
Fabaceae	*Parkinsonia aculeata*	KF379226	ITS
Fabaceae	*Parkinsonia aculeata*	Z70157	rbcL
Fabaceae	*Peltogyne confertiflora*	AF308718	rbcL
Fabaceae	*Peltogyne confertiflora*	AF365163	trnL
Fabaceae	*Peltogyne confertiflora*	AF549274	trnL
Fabaceae	*Peltogyne confertiflora*	AY955798	ITS
Fabaceae	*Peltogyne confertiflora*	EU362021	matK
Fabaceae	*Peltophorum dubium*	AF430785	trnL
Fabaceae	*Peltophorum dubium*	AY386846	matK
Fabaceae	*Peltophorum dubium*	AY904400	rbcL
Fabaceae	*Peltophorum dubium*	AY904432	trnL
Fabaceae	*Peltophorum dubium*	EU361828	trnL
Fabaceae	*Piptadenia gonoacantha*	DQ784663	trnL

**Family**	**Species**	**Accession**	**Barcode**
Fabaceae	*Piptadenia gonoacantha*	DQ790620	matK
Fabaceae	*Piptadenia gonoacantha*	HF545438	trnL
Fabaceae	*Piptadenia paniculata*	DQ784668	trnL
Fabaceae	*Piptadenia paniculata*	DQ784669	trnL
Fabaceae	*Piptadenia paniculata*	HF545441	trnL
Fabaceae	*Pithecellobium dulce*	EF638206	ITS
Fabaceae	*Pithecellobium dulce*	EF638207	ITS
Fabaceae	*Pithecellobium dulce*	HG963628	psbA-trnH
Fabaceae	*Pithecellobium dulce*	HM020740	matK
Fabaceae	*Pithecellobium dulce*	JX856482	ITS
Fabaceae	*Pithecellobium dulce*	JX856483	ITS
Fabaceae	*Pithecellobium dulce*	JX856734	rbcL
Fabaceae	*Pithecellobium dulce*	JX856735	rbcL
Fabaceae	*Pithecellobium dulce*	JX856736	rbcL
Fabaceae	*Pithecellobium dulce*	JX856915	psbA-trnH
Fabaceae	*Pithecellobium dulce*	KC479268	trnL
Fabaceae	*Pithecellobium dulce*	KJ012723	matK
Fabaceae	*Pithecellobium dulce*	KJ082505	rbcL
Fabaceae	*Pithecellobium dulce*	KJ426883	psbA-trnH
Fabaceae	*Plathymenia reticulata*	AF278509	trnL
Fabaceae	*Plathymenia reticulata*	AF521858	matK
Fabaceae	*Plathymenia reticulata*	GQ141980	trnL
Fabaceae	*Plathymenia reticulata*	GQ141981	trnL
Fabaceae	*Plathymenia reticulata*	GQ141982	trnL
Fabaceae	*Plathymenia reticulata*	GQ141983	trnL
Fabaceae	*Plathymenia reticulata*	JX850054	matK
Fabaceae	*Platycyamus regnellii*	AB045817	rbcL
Fabaceae	*Platycyamus regnellii*	AF142709	matK
Fabaceae	*Platycyamus regnellii*	AF311378	trnL
Fabaceae	*Platycyamus regnellii*	AF467491	ITS
Fabaceae	*Platymiscium floribundum*	EU401429	ITS
Fabaceae	*Platymiscium floribundum*	EU735880	ITS
Fabaceae	*Platymiscium floribundum*	EU735881	ITS
Fabaceae	*Platymiscium floribundum*	EU735882	ITS
Fabaceae	*Platymiscium floribundum*	EU735883	ITS

**Family**	**Species**	**Accession**	**Barcode**
Fabaceae	*Platymiscium floribundum*	EU735884	ITS
Fabaceae	*Platymiscium floribundum*	EU735885	ITS
Fabaceae	*Platymiscium floribundum*	EU735941	matK
Fabaceae	*Platymiscium floribundum*	EU735942	matK
Fabaceae	*Platymiscium floribundum*	EU735943	matK
Fabaceae	*Platymiscium floribundum*	EU735944	matK
Fabaceae	*Platymiscium floribundum*	EU735945	matK
Fabaceae	*Platymiscium floribundum*	EU735946	matK
Fabaceae	*Platymiscium floribundum*	EU735997	trnL
Fabaceae	*Platymiscium floribundum*	EU735998	trnL
Fabaceae	*Platymiscium floribundum*	EU735999	trnL
Fabaceae	*Platymiscium floribundum*	EU736000	trnL
Fabaceae	*Platymiscium floribundum*	EU736001	trnL
Fabaceae	*Platymiscium floribundum*	EU736002	trnL
Fabaceae	*Platymiscium floribundum*	EU736054	trnL
Fabaceae	*Platymiscium floribundum*	EU736055	trnL
Fabaceae	*Platymiscium floribundum*	EU736056	trnL
Fabaceae	*Platymiscium floribundum*	EU736057	trnL
Fabaceae	*Platymiscium floribundum*	EU736058	trnL
Fabaceae	*Platymiscium floribundum*	EU736059	trnL
Fabaceae	*Platypodium elegans*	AF208961	trnL
Fabaceae	*Platypodium elegans*	AF270877	matK
Fabaceae	*Platypodium elegans*	GQ981836	rbcL
Fabaceae	*Platypodium elegans*	GQ982065	matK
Fabaceae	*Platypodium elegans*	GQ982322	psbA-trnH
Fabaceae	*Poecilanthe parviflora*	AF142687	matK
Fabaceae	*Poecilanthe parviflora*	AF187089	ITS
Fabaceae	*Poecilanthe parviflora*	AF208897	trnL
Fabaceae	*Poecilanthe parviflora*	KJ028459	matK
Fabaceae	*Poecilanthe parviflora*	KJ028463	ITS
Fabaceae	*Pseudopiptadenia contorta*	DQ784676	trnL
Fabaceae	*Pterocarpus rohrii*	EF451061	ITS
Fabaceae	*Pterocarpus rohrii*	EF451101	trnL
Fabaceae	*Pterocarpus rohrii*	GQ981862	rbcL
Fabaceae	*Pterocarpus rohrii*	GQ982083	matK

Family	Species	Accession	Barcode
Fabaceae	*Pterocarpus rohrii*	GQ982349	psbA-trnH
Fabaceae	*Pterocarpus rohrii*	JN083499	ITS
Fabaceae	*Pterocarpus rohrii*	JN083500	ITS
Fabaceae	*Pterocarpus rohrii*	JN083501	ITS
Fabaceae	*Pterocarpus rohrii*	JN083502	ITS
Fabaceae	*Pterocarpus rohrii*	JN083503	ITS
Fabaceae	*Pterocarpus rohrii*	JN083504	ITS
Fabaceae	*Pterocarpus rohrii*	JN083505	ITS
Fabaceae	*Pterocarpus rohrii*	JN083506	ITS
Fabaceae	*Pterocarpus rohrii*	JN083559	matK
Fabaceae	*Pterocarpus rohrii*	JN083560	matK
Fabaceae	*Pterocarpus rohrii*	JN083561	matK
Fabaceae	*Pterocarpus rohrii*	JN083562	matK
Fabaceae	*Pterocarpus rohrii*	JN083563	matK
Fabaceae	*Pterocarpus rohrii*	JN083564	matK
Fabaceae	*Pterocarpus rohrii*	JN083669	trnL
Fabaceae	*Pterocarpus rohrii*	JN083670	trnL
Fabaceae	*Pterocarpus rohrii*	JN083671	trnL
Fabaceae	*Pterocarpus rohrii*	JN083672	trnL
Fabaceae	*Pterocarpus rohrii*	JN083673	trnL
Fabaceae	*Pterocarpus rohrii*	JN083674	trnL
Fabaceae	*Pterocarpus rohrii*	JN083675	trnL
Fabaceae	*Pterocarpus rohrii*	JN083676	trnL
Fabaceae	*Pterocarpus rohrii*	JN083740	rbcL
Fabaceae	*Pterocarpus rohrii*	JN083741	rbcL
Fabaceae	*Pterocarpus rohrii*	JN083742	rbcL
Fabaceae	*Pterocarpus rohrii*	JN083743	rbcL
Fabaceae	*Pterocarpus rohrii*	JN083744	rbcL
Fabaceae	*Pterocarpus rohrii*	JN083745	rbcL
Fabaceae	*Pterocarpus rohrii*	JN083746	rbcL
Fabaceae	*Pterocarpus rohrii*	JN083747	rbcL
Fabaceae	*Pterocarpus rohrii*	KF436433	ITS
Fabaceae	*Pterocarpus rohrii*	KF436434	ITS
Fabaceae	*Pterocarpus rohrii*	KF436492	trnL
Fabaceae	*Pterodon emarginatus*	JF491258	ITS

Family	Species	Accession	Barcode
Fabaceae	*Pterodon emarginatus*	JF491272	matK
Fabaceae	*Pterodon emarginatus*	JF491286	trnL
Fabaceae	*Pterodon emarginatus*	JX275904	trnL
Fabaceae	*Pterodon emarginatus*	JX295874	matK
Fabaceae	*Pterodon pubescens*	AF187091	ITS
Fabaceae	*Pterodon pubescens*	AF208895	trnL
Fabaceae	*Pterodon pubescens*	AF272094	matK
Fabaceae	*Pterodon pubescens*	AF272095	matK
Fabaceae	*Pterodon pubescens*	AH009912	matK
Fabaceae	*Pterodon pubescens*	JF491259	ITS
Fabaceae	*Pterodon pubescens*	JF491273	matK
Fabaceae	*Pterodon pubescens*	JF491287	trnL
Fabaceae	*Pterogyne nitens*	AF365074	trnL
Fabaceae	*Pterogyne nitens*	AM234247	rbcL
Fabaceae	*Pterogyne nitens*	AY232772	trnL
Fabaceae	*Pterogyne nitens*	AY899689	trnL
Fabaceae	*Pterogyne nitens*	AY904377	rbcL
Fabaceae	*Pterogyne nitens*	EU362031	matK
Fabaceae	*Riedeliella graciliflora*	AF208949	trnL
Fabaceae	*Riedeliella graciliflora*	AF272090	matK
Fabaceae	*Riedeliella graciliflora*	AF272091	matK
Fabaceae	*Riedeliella graciliflora*	AH009910	matK
Fabaceae	*Schizolobium parahyba*	AF365108	trnL
Fabaceae	*Schizolobium parahyba*	AF524985	psbA-trnH
Fabaceae	*Schizolobium parahyba*	AY899710	trnL
Fabaceae	*Schizolobium parahyba*	AY899711	trnL
Fabaceae	*Schizolobium parahyba*	AY904398	rbcL
Fabaceae	*Schizolobium parahyba*	EU362036	matK
Fabaceae	*Schizolobium parahyba*	FJ668606	psbA-trnH
Fabaceae	*Schizolobium parahyba*	FJ668607	psbA-trnH
Fabaceae	*Schizolobium parahyba*	FJ668610	psbA-trnH
Fabaceae	*Schizolobium parahyba*	FJ668611	psbA-trnH
Fabaceae	*Schizolobium parahyba*	FJ668612	psbA-trnH
Fabaceae	*Schizolobium parahyba*	FJ668613	psbA-trnH
Fabaceae	*Schizolobium parahyba*	FJ668616	rpoB

Family	Species	Accession	Barcode
Fabaceae	*Schizolobium parahyba*	FJ668618	trnL
Fabaceae	*Schizolobium parahyba*	FJ668626	matK
Fabaceae	*Schizolobium parahyba*	FJ668627	matK
Fabaceae	*Schizolobium parahyba*	FJ668628	matK
Fabaceae	*Schizolobium parahyba*	FJ668629	matK
Fabaceae	*Schizolobium parahyba*	FJ668630	matK
Fabaceae	*Schizolobium parahyba*	FJ668631	matK
Fabaceae	*Schizolobium parahyba*	GQ167769	psbA-trnH
Fabaceae	*Schizolobium parahyba*	GQ167771	ITS
Fabaceae	*Schizolobium parahyba*	GQ167772	ITS
Fabaceae	*Schizolobium parahyba*	GQ981870	rbcL
Fabaceae	*Schizolobium parahyba*	GQ982090	matK
Fabaceae	*Schizolobium parahyba*	GQ982357	psbA-trnH
Fabaceae	*Senegalia polyphylla*	AF274147	matK
Fabaceae	*Senegalia polyphylla*	AF522980	trnL
Fabaceae	*Senegalia polyphylla*	AF525000	psbA-trnH
Fabaceae	*Senegalia tenuifolia*	FJ037903	matK
Fabaceae	*Senegalia tenuifolia*	FJ038029	rbcL
Fabaceae	*Senegalia tenuifolia*	FJ038262	rpoB
Fabaceae	*Senegalia tenuifolia*	FJ038513	rpoC1
Fabaceae	*Senegalia tenuifolia*	FJ038932	psbA-trnH
Fabaceae	*Senegalia tenuifolia*	JQ587485	matK
Fabaceae	*Senegalia tenuifolia*	JQ587486	matK
Fabaceae	*Senegalia tenuifolia*	JQ587487	matK
Fabaceae	*Senegalia tenuifolia*	JQ587884	matK
Fabaceae	*Senegalia tenuifolia*	JQ587885	matK
Fabaceae	*Senegalia tenuifolia*	JQ587886	matK
Fabaceae	*Senegalia tenuifolia*	JQ587887	matK
Fabaceae	*Senegalia tenuifolia*	JQ587888	matK
Fabaceae	*Senegalia tenuifolia*	JQ587901	matK
Fabaceae	*Senegalia tenuifolia*	JQ587902	matK
Fabaceae	*Senegalia tenuifolia*	JQ587903	matK
Fabaceae	*Senegalia tenuifolia*	JQ589982	matK
Fabaceae	*Senegalia tenuifolia*	JQ591535	rbcL
Fabaceae	*Senegalia tenuifolia*	JQ591536	rbcL

**Family**	**Species**	**Accession**	**Barcode**
Fabaceae	*Senegalia tenuifolia*	JQ591537	rbcL
Fabaceae	*Senegalia tenuifolia*	JQ591538	rbcL
Fabaceae	*Senegalia tenuifolia*	JQ592070	rbcL
Fabaceae	*Senegalia tenuifolia*	JQ592071	rbcL
Fabaceae	*Senegalia tenuifolia*	JQ592072	rbcL
Fabaceae	*Senegalia tenuifolia*	JQ592073	rbcL
Fabaceae	*Senegalia tenuifolia*	JQ592074	rbcL
Fabaceae	*Senegalia tenuifolia*	JQ592085	rbcL
Fabaceae	*Senegalia tenuifolia*	JQ592086	rbcL
Fabaceae	*Senegalia tenuifolia*	JQ592087	rbcL
Fabaceae	*Senegalia tenuifolia*	JQ594971	rbcL
Fabaceae	*Senegalia tenuifolia*	KJ593761	matK
Fabaceae	*Senegalia tenuifolia*	KJ594092	rbcL
Fabaceae	*Senna alata*	AF365091	trnL
Fabaceae	*Senna alata*	AM086839	matK
Fabaceae	*Senna alata*	AY232769	trnL
Fabaceae	*Senna alata*	EU362042	matK
Fabaceae	*Senna alata*	FJ980412	ITS
Fabaceae	*Senna alata*	GQ434748	ITS
Fabaceae	*Senna alata*	GQ435366	psbA-trnH
Fabaceae	*Senna alata*	GQ436678	rbcL
Fabaceae	*Senna alata*	GU396790	psbA-trnH
Fabaceae	*Senna alata*	GU942495	matK
Fabaceae	*Senna alata*	GU969278	psbA-trnH
Fabaceae	*Senna alata*	HG963646	psbA-trnH
Fabaceae	*Senna alata*	HQ161753	psbA-trnH
Fabaceae	*Senna alata*	HQ833041	ITS
Fabaceae	*Senna alata*	JF975364	rbcL
Fabaceae	*Senna alata*	JQ301828	ITS
Fabaceae	*Senna alata*	JQ301848	rbcL
Fabaceae	*Senna alata*	JQ301868	matK
Fabaceae	*Senna alata*	KJ638412	ITS
Fabaceae	*Senna alata*	KJ638413	ITS
Fabaceae	*Senna alata*	KJ638414	ITS
Fabaceae	*Senna alata*	KJ638432	matK

**Family**	**Species**	**Accession**	**Barcode**
Fabaceae	*Senna alata*	KJ638433	matK
Fabaceae	*Senna alata*	KJ638434	matK
Fabaceae	*Senna alata*	KJ638460	psbA-trnH
Fabaceae	*Senna alata*	KJ638461	psbA-trnH
Fabaceae	*Senna alata*	KT308089	ITS
Fabaceae	*Senna alata*	U74250	rbcL
Fabaceae	*Senna cana*	AM086608	matK
Fabaceae	*Senna corymbosa*	AM086856	matK
Fabaceae	*Senna corymbosa*	HQ833047	ITS
Fabaceae	*Senna macranthera*	AM086873	matK
Fabaceae	*Senna macranthera*	JX856680	rbcL
Fabaceae	*Senna multijuga*	AM086611	matK
Fabaceae	*Senna multijuga*	AM086879	matK
Fabaceae	*Senna pendula*	AM086887	matK
Fabaceae	*Senna pendula*	GU135008	matK
Fabaceae	*Senna pendula*	GU135101	matK
Fabaceae	*Senna pendula*	GU135268	rbcL
Fabaceae	*Senna pendula*	GU135439	psbA-trnH
Fabaceae	*Senna polyphylla*	KJ012766	matK
Fabaceae	*Senna polyphylla*	KJ082563	rbcL
Fabaceae	*Senna polyphylla*	KJ426930	psbA-trnH
Fabaceae	*Senna polyphylla*	KJ605890	psbA-trnH
Fabaceae	*Senna polyphylla*	KJ605891	psbA-trnH
Fabaceae	*Senna polyphylla*	KJ605899	ITS
Fabaceae	*Senna polyphylla*	KJ605900	ITS
Fabaceae	*Senna polyphylla*	KJ605901	ITS
Fabaceae	*Senna polyphylla*	KJ638449	matK
Fabaceae	*Senna polyphylla*	KJ638450	matK
Fabaceae	*Senna polyphylla*	KJ638451	matK
Fabaceae	*Senna polyphylla*	KJ638472	psbA-trnH
Fabaceae	*Senna rugosa*	AM086616	matK
Fabaceae	*Senna siamea*	AM086897	matK
Fabaceae	*Senna siamea*	GU969279	psbA-trnH
Fabaceae	*Senna siamea*	HG963781	psbA-trnH
Fabaceae	*Senna siamea*	HQ161767	psbA-trnH

**Family**	**Species**	**Accession**	**Barcode**
Fabaceae	*Senna siamea*	JQ301842	ITS
Fabaceae	*Senna siamea*	JQ301862	rbcL
Fabaceae	*Senna siamea*	JQ301882	matK
Fabaceae	*Senna siamea*	KC984644	ITS
Fabaceae	*Senna siamea*	KJ012767	matK
Fabaceae	*Senna siamea*	KJ082564	rbcL
Fabaceae	*Senna siamea*	KJ426931	psbA-trnH
Fabaceae	*Senna siamea*	KJ638421	ITS
Fabaceae	*Senna siamea*	KJ638422	ITS
Fabaceae	*Senna siamea*	KJ638423	ITS
Fabaceae	*Senna siamea*	KJ638438	matK
Fabaceae	*Senna siamea*	KJ638467	psbA-trnH
Fabaceae	*Senna siamea*	KJ638468	psbA-trnH
Fabaceae	*Senna siamea*	KJ638469	psbA-trnH
Fabaceae	*Senna siamea*	KT308091	ITS
Fabaceae	*Senna silvestris*	AM086898	matK
Fabaceae	*Senna spectabilis*	AM086900	matK
Fabaceae	*Senna spectabilis*	HQ161761	psbA-trnH
Fabaceae	*Senna spectabilis*	JF838360	psbA-trnH
Fabaceae	*Senna spectabilis*	JQ301836	ITS
Fabaceae	*Senna spectabilis*	JQ301856	rbcL
Fabaceae	*Senna spectabilis*	JQ301876	matK
Fabaceae	*Senna spectabilis*	KC414137	psbA-trnH
Fabaceae	*Senna spectabilis*	KJ082565	rbcL
Fabaceae	*Senna spectabilis*	KJ426932	psbA-trnH
Fabaceae	*Senna splendida*	AM086617	matK
Fabaceae	*Senna velutina*	HM236875	matK
Fabaceae	*Sesbania sesban*	HM468272	psbA-trnH
Fabaceae	*Sesbania sesban*	HM468338	ITS
Fabaceae	*Sesbania sesban*	HQ730422	matK
Fabaceae	*Sesbania sesban*	JQ669578	matK
Fabaceae	*Sesbania sesban*	JX453702	ITS
Fabaceae	*Sesbania sesban*	JX453703	ITS
Fabaceae	*Sesbania sesban*	JX453704	ITS
Fabaceae	*Sesbania sesban*	JX453705	ITS

**Family**	**Species**	**Accession**	**Barcode**
Fabaceae	*Sesbania sesban*	Z95541	rbcL
Fabaceae	*Sophora tomentosa*	AB127030	trnL
Fabaceae	*Sophora tomentosa*	AB127038	rbcL
Fabaceae	*Sophora tomentosa*	AB817493	trnL
Fabaceae	*Sophora tomentosa*	AY725481	rbcL
Fabaceae	*Sophora tomentosa*	AY725482	ITS
Fabaceae	*Sophora tomentosa*	DQ499085	ITS
Fabaceae	*Sophora tomentosa*	HQ207666	ITS
Fabaceae	*Sophora tomentosa*	JF279450	ITS
Fabaceae	*Sophora tomentosa*	JX495451	ITS
Fabaceae	*Sophora tomentosa*	JX495463	psbA-trnH
Fabaceae	*Stryphnodendron adstringens*	DQ784678	trnL
Fabaceae	*Stryphnodendron adstringens*	HF545439	trnL
Fabaceae	*Stryphnodendron rotundifolium*	DQ784685	trnL
Fabaceae	*Stryphnodendron rotundifolium*	DQ790643	matK
Fabaceae	*Swartzia flaemingii*	AY386941	matK
Fabaceae	*Swartzia langsdorffii*	EF466307	trnL
Fabaceae	*Swartzia langsdorffii*	EF527528	trnL
Fabaceae	*Swartzia langsdorffii*	EF560858	ITS
Fabaceae	*Swartzia myrtifolia*	EF466318	trnL
Fabaceae	*Swartzia myrtifolia*	EF527539	trnL
Fabaceae	*Swartzia myrtifolia*	EF560877	ITS
Fabaceae	*Swartzia simplex*	AF142678	matK
Fabaceae	*Swartzia simplex*	AF309843	trnL
Fabaceae	*Swartzia simplex*	AJ581415	matK
Fabaceae	*Swartzia simplex*	EF466345	trnL
Fabaceae	*Swartzia simplex*	EF466346	trnL
Fabaceae	*Swartzia simplex*	EF466347	trnL
Fabaceae	*Swartzia simplex*	EF527566	trnL
Fabaceae	*Swartzia simplex*	EF527567	trnL
Fabaceae	*Swartzia simplex*	EF527568	trnL
Fabaceae	*Swartzia simplex*	EF560918	ITS
Fabaceae	*Swartzia simplex*	EF560919	ITS
Fabaceae	*Swartzia simplex*	EF560920	ITS
Fabaceae	*Swartzia simplex*	GQ981887	rbcL

Family	Species	Accession	Barcode
Fabaceae	*Swartzia simplex*	GQ981888	rbcL
Fabaceae	*Swartzia simplex*	GQ982105	matK
Fabaceae	*Swartzia simplex*	GQ982106	matK
Fabaceae	*Swartzia simplex*	GQ982375	psbA-trnH
Fabaceae	*Swartzia simplex*	JQ587870	matK
Fabaceae	*Swartzia simplex*	JQ587871	matK
Fabaceae	*Swartzia simplex*	JQ587872	matK
Fabaceae	*Swartzia simplex*	JQ587873	matK
Fabaceae	*Swartzia simplex*	JQ587874	matK
Fabaceae	*Swartzia simplex*	JQ592055	rbcL
Fabaceae	*Swartzia simplex*	JQ592056	rbcL
Fabaceae	*Swartzia simplex*	JQ592057	rbcL
Fabaceae	*Swartzia simplex*	JQ592058	rbcL
Fabaceae	*Swartzia simplex*	JQ592059	rbcL
Fabaceae	*Swartzia simplex*	JQ592060	rbcL
Fabaceae	*Swartzia submarginata*	EF466272	trnL
Fabaceae	*Swartzia submarginata*	EF527493	trnL
Fabaceae	*Swartzia submarginata*	EF560807	ITS
Fabaceae	*Sweetia fruticosa*	AF524883	trnL
Fabaceae	*Sweetia fruticosa*	AY386911	matK
Fabaceae	*Sweetia fruticosa*	EF457730	ITS
Fabaceae	*Sweetia fruticosa*	JX152619	matK
Fabaceae	*Sweetia fruticosa*	JX152620	matK
Fabaceae	*Sweetia fruticosa*	JX152621	matK
Fabaceae	*Sweetia fruticosa*	JX152669	ITS
Fabaceae	*Sweetia fruticosa*	JX152693	ITS
Fabaceae	*Sweetia fruticosa*	JX152694	ITS
Fabaceae	*Sweetia fruticosa*	JX152695	ITS
Fabaceae	*Sweetia fruticosa*	JX187671	trnL
Fabaceae	*Sweetia fruticosa*	JX187672	trnL
Fabaceae	*Sweetia fruticosa*	JX187673	trnL
Fabaceae	*Sweetia fruticosa*	JX187747	trnL
Fabaceae	*Sweetia fruticosa*	JX187748	trnL
Fabaceae	*Sweetia fruticosa*	JX187791	psbA-trnH
Fabaceae	*Sweetia fruticosa*	JX187792	psbA-trnH

**Family**	**Species**	**Accession**	**Barcode**
Fabaceae	*Sweetia fruticosa*	JX187793	psbA-trnH
Fabaceae	*Sweetia fruticosa*	JX187839	psbA-trnH
Fabaceae	*Sweetia fruticosa*	KC595406	ITS
Fabaceae	*Sweetia fruticosa*	KC595407	ITS
Fabaceae	*Sweetia fruticosa*	KC595408	ITS
Fabaceae	*Sweetia fruticosa*	KC595409	ITS
Fabaceae	*Sweetia fruticosa*	KC595410	ITS
Fabaceae	*Sweetia fruticosa*	KC595411	ITS
Fabaceae	*Tachigali aurea*	KR872710	matK
Fabaceae	*Tachigali denudata*	KR492616	ITS
Fabaceae	*Tachigali denudata*	KR872668	psbA-trnH
Fabaceae	*Tachigali denudata*	KR872692	trnL
Fabaceae	*Tachigali denudata*	KR872715	matK
Fabaceae	*Tachigali paratyensis*	KR872680	psbA-trnH
Fabaceae	*Tachigali paratyensis*	KR872728	matK
Fabaceae	*Tachigali vulgaris*	AF309477	trnL
Fabaceae	*Tachigali vulgaris*	KR492633	ITS
Fabaceae	*Tachigali vulgaris*	KR872677	psbA-trnH
Fabaceae	*Tachigali vulgaris*	KR872678	psbA-trnH
Fabaceae	*Tachigali vulgaris*	KR872688	psbA-trnH
Fabaceae	*Tachigali vulgaris*	KR872700	trnL
Fabaceae	*Tachigali vulgaris*	KR872701	trnL
Fabaceae	*Tachigali vulgaris*	KR872708	trnL
Fabaceae	*Tachigali vulgaris*	KR872725	matK
Fabaceae	*Tachigali vulgaris*	KR872726	matK
Fabaceae	*Tachigali vulgaris*	KR872738	matK
Fabaceae	*Tipuana tipu*	AF189056	ITS
Fabaceae	*Tipuana tipu*	AF208956	trnL
Fabaceae	*Tipuana tipu*	AF270882	matK
Fabaceae	*Tipuana tipu*	EU735869	trnL
Fabaceae	*Tipuana tipu*	JN083777	rbcL
Fabaceae	*Tipuana tipu*	JX495767	matK
Fabaceae	*Tipuana tipu*	JX571906	rbcL
Fabaceae	*Tipuana tipu*	KF436436	ITS
Fabaceae	*Tipuana tipu*	KF436446	matK

**Family**	**Species**	**Accession**	**Barcode**
Fabaceae	*Tipuana tipu*	KF436476	rbcL
Fabaceae	*Vatairea heteroptera*	JX152603	matK
Fabaceae	*Vatairea heteroptera*	JX152604	matK
Fabaceae	*Vatairea heteroptera*	JX152677	ITS
Fabaceae	*Vatairea heteroptera*	JX152678	ITS
Fabaceae	*Vatairea heteroptera*	JX187655	trnL
Fabaceae	*Vatairea heteroptera*	JX187656	trnL
Fabaceae	*Vatairea heteroptera*	JX187731	trnL
Fabaceae	*Vatairea heteroptera*	JX187775	psbA-trnH
Fabaceae	*Vatairea heteroptera*	JX187776	psbA-trnH
Fabaceae	*Vatairea heteroptera*	KC595379	ITS
Fabaceae	*Vatairea heteroptera*	KC595380	ITS
Fabaceae	*Vatairea heteroptera*	KC595381	ITS
Fabaceae	*Vatairea heteroptera*	KC595382	ITS
Fabaceae	*Vatairea macrocarpa*	AF265560	trnL
Fabaceae	*Vatairea macrocarpa*	AF309835	trnL
Fabaceae	*Vatairea macrocarpa*	AY293848	ITS
Fabaceae	*Vatairea macrocarpa*	AY293849	ITS
Fabaceae	*Vatairea macrocarpa*	AY386927	matK
Fabaceae	*Vatairea macrocarpa*	EF457732	ITS
Fabaceae	*Vatairea macrocarpa*	JX152608	matK
Fabaceae	*Vatairea macrocarpa*	JX152609	matK
Fabaceae	*Vatairea macrocarpa*	JX152610	matK
Fabaceae	*Vatairea macrocarpa*	JX152682	ITS
Fabaceae	*Vatairea macrocarpa*	JX152683	ITS
Fabaceae	*Vatairea macrocarpa*	JX152684	ITS
Fabaceae	*Vatairea macrocarpa*	JX187660	trnL
Fabaceae	*Vatairea macrocarpa*	JX187661	trnL
Fabaceae	*Vatairea macrocarpa*	JX187662	trnL
Fabaceae	*Vatairea macrocarpa*	JX187736	trnL
Fabaceae	*Vatairea macrocarpa*	JX187738	trnL
Fabaceae	*Vatairea macrocarpa*	JX187780	psbA-trnH
Fabaceae	*Vatairea macrocarpa*	JX187781	psbA-trnH
Fabaceae	*Vatairea macrocarpa*	JX187782	psbA-trnH
Fabaceae	*Vatairea macrocarpa*	KC595387	ITS

Family	Species	Accession	Barcode
Fabaceae	*Vatairea macrocarpa*	KC595388	ITS
Fabaceae	*Vatairea macrocarpa*	KC595389	ITS
Fabaceae	*Vatairea macrocarpa*	KC595390	ITS
Fabaceae	*Vatairea macrocarpa*	KC595391	ITS
Fabaceae	*Vatairea macrocarpa*	KC595392	ITS
Fabaceae	*Vatairea macrocarpa*	KC595393	ITS
Fabaceae	*Vatairea macrocarpa*	KC595394	ITS
Fabaceae	*Vatairea macrocarpa*	KC595395	ITS
Fabaceae	*Vatairea macrocarpa*	KC595396	ITS
Fabaceae	*Vatairea macrocarpa*	KC595397	ITS
Fabaceae	*Vatairea macrocarpa*	KC595398	ITS
Fabaceae	*Zollernia glabra*	JX187723	trnL
Fabaceae	*Zollernia glabra*	JX275942	trnL
Fabaceae	*Zollernia glabra*	JX275947	trnL
Fabaceae	*Zollernia glabra*	JX295915	matK
Fabaceae	*Zollernia glabra*	JX295916	matK
Fabaceae	*Zollernia ilicifolia*	JX152655	matK
Fabaceae	*Zollernia ilicifolia*	JX152670	ITS
Fabaceae	*Zollernia ilicifolia*	JX187648	trnL
Fabaceae	*Zollernia ilicifolia*	JX187724	trnL
Fabaceae	*Zollernia ilicifolia*	JX187768	psbA-trnH
Griseliniaceae	*Griselinia ruscifolia*	AJ536584	ITS
Griseliniaceae	*Griselinia ruscifolia*	AJ536585	ITS
Griseliniaceae	*Griselinia ruscifolia*	AJ537459	trnL
Griseliniaceae	*Griselinia ruscifolia*	AJ537460	trnL
Lacistemataceae	*Lacistema aggregatum*	AB233790	matK
Lacistemataceae	*Lacistema aggregatum*	AB233894	rbcL
Lacistemataceae	*Lacistema aggregatum*	AY935746	rbcL
Lacistemataceae	*Lacistema aggregatum*	AY935787	trnL
Lacistemataceae	*Lacistema aggregatum*	AY935933	matK
Lacistemataceae	*Lacistema aggregatum*	FJ670025	matK
Lacistemataceae	*Lacistema aggregatum*	GQ982027	matK
Lamiaceae	*Aegiphila brachiata*	FJ952019	trnL
Lamiaceae	*Aegiphila brachiata*	FJ952075	trnL
Lamiaceae	*Aegiphila vitelliniflora*	FJ952016	trnL

Family	Species	Accession	Barcode
Lamiaceae	*Aegiphila vitelliniflora*	FJ952072	trnL
Lamiaceae	*Hyptidendron canum*	JF301556	ITS
Lamiaceae	*Hyptidendron canum*	JF357803	trnL
Lamiaceae	*Hyptidendron canum*	JF357869	matK
Lamiaceae	*Vitex agnus-castus*	AB284182	matK
Lamiaceae	*Vitex agnus-castus*	DQ070731	ITS
Lamiaceae	*Vitex agnus-castus*	EU785943	ITS
Lamiaceae	*Vitex agnus-castus*	FM200111	ITS
Lamiaceae	*Vitex agnus-castus*	HQ384496	matK
Lamiaceae	*Vitex agnus-castus*	HQ412926	trnL
Lamiaceae	*Vitex agnus-castus*	JN408586	trnL
Lamiaceae	*Vitex agnus-castus*	JN575350	ITS
Lamiaceae	*Vitex agnus-castus*	U78716	rbcL
Lamiaceae	*Vitex megapotamica*	KF420935	ITS
Lamiaceae	*Vitex megapotamica*	KF420936	ITS
Lamiaceae	*Vitex megapotamica*	KF420937	ITS
Lamiaceae	*Vitex megapotamica*	KF420938	ITS
Lamiaceae	*Vitex megapotamica*	KF421100	psbA-trnH
Lamiaceae	*Vitex megapotamica*	KF421101	psbA-trnH
Lamiaceae	*Vitex megapotamica*	KF421102	psbA-trnH
Lamiaceae	*Vitex megapotamica*	KF421103	psbA-trnH
Lamiaceae	*Vitex megapotamica*	KF421104	psbA-trnH
Lamiaceae	*Vitex megapotamica*	KF421105	psbA-trnH
Lamiaceae	*Vitex megapotamica*	KF561969	rbcL
Lamiaceae	*Vitex megapotamica*	KF561970	rbcL
Lauraceae	*Aiouea trinervis*	GQ480363	ITS
Lauraceae	*Aniba heringeri*	GQ480364	ITS
Lauraceae	*Beilschmiedia emarginata*	HG314958	matK
Lauraceae	*Beilschmiedia emarginata*	HG315534	ITS
Lauraceae	*Cinnamomum triplinerve*	EU153832	matK
Lauraceae	*Cinnamomum triplinerve*	EU153833	matK
Lauraceae	*Cinnamomum triplinerve*	EU153834	matK
Lauraceae	*Cinnamomum triplinerve*	EU153892	rpoB
Lauraceae	*Cinnamomum triplinerve*	EU153893	rpoB
Lauraceae	*Cinnamomum triplinerve*	EU153894	rpoB

**Family**	**Species**	**Accession**	**Barcode**
Lauraceae	*Cinnamomum triplinerve*	EU153950	psbA-trnH
Lauraceae	*Cinnamomum triplinerve*	EU153951	psbA-trnH
Lauraceae	*Cinnamomum triplinerve*	EU153952	psbA-trnH
Lauraceae	*Cinnamomum triplinerve*	EU154006	rpoC1
Lauraceae	*Cinnamomum triplinerve*	EU154007	rpoC1
Lauraceae	*Cinnamomum triplinerve*	EU154008	rpoC1
Lauraceae	*Cinnamomum triplinerve*	GQ981703	rbcL
Lauraceae	*Cinnamomum triplinerve*	GQ981967	matK
Lauraceae	*Cinnamomum triplinerve*	GQ982187	psbA-trnH
Lauraceae	*Cinnamomum triplinerve*	JQ589851	matK
Lauraceae	*Cinnamomum triplinerve*	JQ594840	rbcL
Lauraceae	*Cryptocarya aschersoniana*	KF421006	ITS
Lauraceae	*Cryptocarya aschersoniana*	KF421007	ITS
Lauraceae	*Cryptocarya aschersoniana*	KF421020	psbA-trnH
Lauraceae	*Cryptocarya aschersoniana*	KF421021	psbA-trnH
Lauraceae	*Cryptocarya aschersoniana*	KF555389	matK
Lauraceae	*Cryptocarya aschersoniana*	KF561908	rbcL
Lauraceae	*Cryptocarya aschersoniana*	KF561909	rbcL
Lauraceae	*Cryptocarya botelhensis*	HG314981	matK
Lauraceae	*Cryptocarya botelhensis*	HG314982	matK
Lauraceae	*Cryptocarya botelhensis*	HG315560	ITS
Lauraceae	*Cryptocarya botelhensis*	HG315561	ITS
Lauraceae	*Cryptocarya mandioccana*	HG314989	matK
Lauraceae	*Cryptocarya mandioccana*	HG315568	ITS
Lauraceae	*Cryptocarya mandioccana*	HG315569	ITS
Lauraceae	*Cryptocarya mandioccana*	HG315570	ITS
Lauraceae	*Cryptocarya moschata*	HG314991	matK
Lauraceae	*Cryptocarya moschata*	HG315578	ITS
Lauraceae	*Cryptocarya moschata*	HG315579	ITS
Lauraceae	*Cryptocarya saligna*	HG314995	matK
Lauraceae	*Cryptocarya saligna*	HG314996	matK
Lauraceae	*Cryptocarya saligna*	HG315583	ITS
Lauraceae	*Cryptocarya saligna*	HG315584	ITS
Lauraceae	*Endlicheria paniculata*	AF363378	ITS
Lauraceae	*Endlicheria paniculata*	GQ480367	ITS

**Family**	**Species**	**Accession**	**Barcode**
Lauraceae	*Nectandra cissiflora*	EU153846	matK
Lauraceae	*Nectandra cissiflora*	EU153847	matK
Lauraceae	*Nectandra cissiflora*	EU153848	matK
Lauraceae	*Nectandra cissiflora*	EU153849	matK
Lauraceae	*Nectandra cissiflora*	EU153906	rpoB
Lauraceae	*Nectandra cissiflora*	EU153907	rpoB
Lauraceae	*Nectandra cissiflora*	EU153908	rpoB
Lauraceae	*Nectandra cissiflora*	EU153909	rpoB
Lauraceae	*Nectandra cissiflora*	EU153962	psbA-trnH
Lauraceae	*Nectandra cissiflora*	EU153963	psbA-trnH
Lauraceae	*Nectandra cissiflora*	EU153964	psbA-trnH
Lauraceae	*Nectandra cissiflora*	EU153965	psbA-trnH
Lauraceae	*Nectandra cissiflora*	EU154020	rpoC1
Lauraceae	*Nectandra cissiflora*	EU154021	rpoC1
Lauraceae	*Nectandra cissiflora*	EU154022	rpoC1
Lauraceae	*Nectandra cissiflora*	EU154023	rpoC1
Lauraceae	*Nectandra cissiflora*	GQ981810	rbcL
Lauraceae	*Nectandra cissiflora*	GQ982048	matK
Lauraceae	*Nectandra cissiflora*	GQ982296	psbA-trnH
Lauraceae	*Nectandra cuspidata*	AF272291	ITS
Lauraceae	*Nectandra cuspidata*	EU153850	matK
Lauraceae	*Nectandra cuspidata*	EU153851	matK
Lauraceae	*Nectandra cuspidata*	EU153910	rpoB
Lauraceae	*Nectandra cuspidata*	EU153911	rpoB
Lauraceae	*Nectandra cuspidata*	EU153966	psbA-trnH
Lauraceae	*Nectandra cuspidata*	EU153967	psbA-trnH
Lauraceae	*Nectandra cuspidata*	EU154024	rpoC1
Lauraceae	*Nectandra cuspidata*	EU154025	rpoC1
Lauraceae	*Nectandra cuspidata*	GQ480369	ITS
Lauraceae	*Nectandra grandiflora*	KF420968	ITS
Lauraceae	*Nectandra grandiflora*	KF420969	ITS
Lauraceae	*Nectandra grandiflora*	KF420970	ITS
Lauraceae	*Nectandra grandiflora*	KF420971	ITS
Lauraceae	*Nectandra grandiflora*	KF420972	ITS
Lauraceae	*Nectandra grandiflora*	KF420973	ITS

Family	Species	Accession	Barcode
Lauraceae	*Nectandra grandiflora*	KF421022	psbA-trnH
Lauraceae	*Nectandra grandiflora*	KF421023	psbA-trnH
Lauraceae	*Nectandra grandiflora*	KF421024	psbA-trnH
Lauraceae	*Nectandra grandiflora*	KF421025	psbA-trnH
Lauraceae	*Nectandra grandiflora*	KF555420	matK
Lauraceae	*Nectandra grandiflora*	KF555421	matK
Lauraceae	*Nectandra grandiflora*	KF561939	rbcL
Lauraceae	*Nectandra grandiflora*	KF561940	rbcL
Lauraceae	*Nectandra grandiflora*	KF561941	rbcL
Lauraceae	*Nectandra hihua*	HM446971	psbA-trnH
Lauraceae	*Nectandra hihua*	JQ588098	matK
Lauraceae	*Nectandra hihua*	JQ592325	rbcL
Lauraceae	*Nectandra hihua*	JQ592326	rbcL
Lauraceae	*Nectandra hihua*	JQ592327	rbcL
Lauraceae	*Nectandra hihua*	JQ592328	rbcL
Lauraceae	*Nectandra hihua*	JQ592329	rbcL
Lauraceae	*Nectandra hihua*	JQ592330	rbcL
Lauraceae	*Nectandra hihua*	JQ592331	rbcL
Lauraceae	*Nectandra hihua*	JQ592332	rbcL
Lauraceae	*Nectandra hihua*	JQ592333	rbcL
Lauraceae	*Nectandra hihua*	KJ082455	rbcL
Lauraceae	*Nectandra hihua*	KJ426843	psbA-trnH
Lauraceae	*Nectandra lanceolata*	GQ480370	ITS
Lauraceae	*Nectandra lanceolata*	KF420966	ITS
Lauraceae	*Nectandra lanceolata*	KF420967	ITS
Lauraceae	*Nectandra lanceolata*	KF421026	psbA-trnH
Lauraceae	*Nectandra lanceolata*	KF421027	psbA-trnH
Lauraceae	*Nectandra lanceolata*	KF421028	psbA-trnH
Lauraceae	*Nectandra lanceolata*	KF555422	matK
Lauraceae	*Nectandra lanceolata*	KF555423	matK
Lauraceae	*Nectandra lanceolata*	KF555424	matK
Lauraceae	*Nectandra lanceolata*	KF561942	rbcL
Lauraceae	*Nectandra lanceolata*	KF561943	rbcL
Lauraceae	*Nectandra lanceolata*	KF561944	rbcL
Lauraceae	*Nectandra megapotamica*	GQ480371	ITS

**Family**	**Species**	**Accession**	**Barcode**
Lauraceae	*Nectandra megapotamica*	KF420964	ITS
Lauraceae	*Nectandra megapotamica*	KF420965	ITS
Lauraceae	*Nectandra megapotamica*	KF421029	psbA-trnH
Lauraceae	*Nectandra megapotamica*	KF421030	psbA-trnH
Lauraceae	*Nectandra megapotamica*	KF421031	psbA-trnH
Lauraceae	*Nectandra megapotamica*	KF555425	matK
Lauraceae	*Nectandra megapotamica*	KF555426	matK
Lauraceae	*Nectandra megapotamica*	KF555427	matK
Lauraceae	*Nectandra megapotamica*	KF561945	rbcL
Lauraceae	*Nectandra megapotamica*	KF561946	rbcL
Lauraceae	*Nectandra megapotamica*	KF561947	rbcL
Lauraceae	*Nectandra membranacea*	AF268730	trnL
Lauraceae	*Nectandra membranacea*	AF268767	psbA-trnH
Lauraceae	*Nectandra membranacea*	AF268825	trnL
Lauraceae	*Nectandra membranacea*	JQ589850	matK
Lauraceae	*Nectandra membranacea*	JQ594839	rbcL
Lauraceae	*Nectandra membranacea*	KJ012695	matK
Lauraceae	*Nectandra membranacea*	KJ082457	rbcL
Lauraceae	*Nectandra nitidula*	GQ480372	ITS
Lauraceae	*Nectandra psammophila*	AF272292	ITS
Lauraceae	*Nectandra reticulata*	GQ480373	ITS
Lauraceae	*Ocotea aciphylla*	AF268766	psbA-trnH
Lauraceae	*Ocotea aciphylla*	AF272314	ITS
Lauraceae	*Ocotea aciphylla*	DQ787422	ITS
Lauraceae	*Ocotea aciphylla*	GQ480374	ITS
Lauraceae	*Ocotea bicolor*	GQ480375	ITS
Lauraceae	*Ocotea brachybotrya*	GQ480376	ITS
Lauraceae	*Ocotea catharinensis*	KF420963	ITS
Lauraceae	*Ocotea catharinensis*	KF421032	psbA-trnH
Lauraceae	*Ocotea catharinensis*	KF421033	psbA-trnH
Lauraceae	*Ocotea catharinensis*	KF421034	psbA-trnH
Lauraceae	*Ocotea catharinensis*	KF555428	matK
Lauraceae	*Ocotea catharinensis*	KF555429	matK
Lauraceae	*Ocotea catharinensis*	KF555430	matK
Lauraceae	*Ocotea catharinensis*	KF561948	rbcL

**Family**	**Species**	**Accession**	**Barcode**
Lauraceae	*Ocotea catharinensis*	KF561949	rbcL
Lauraceae	*Ocotea catharinensis*	KF561950	rbcL
Lauraceae	*Ocotea corymbosa*	GQ480377	ITS
Lauraceae	*Ocotea daphnifolia*	GQ480378	ITS
Lauraceae	*Ocotea diospyrifolia*	GQ480379	ITS
Lauraceae	*Ocotea indecora*	GQ480382	ITS
Lauraceae	*Ocotea lancifolia*	GQ480383	ITS
Lauraceae	*Ocotea laxa*	GQ480384	ITS
Lauraceae	*Ocotea minarum*	GQ480386	ITS
Lauraceae	*Ocotea odorifera*	AF268738	trnL
Lauraceae	*Ocotea odorifera*	AF268762	psbA-trnH
Lauraceae	*Ocotea odorifera*	AF272309	ITS
Lauraceae	*Ocotea odorifera*	GQ480388	ITS
Lauraceae	*Ocotea odorifera*	KF420960	ITS
Lauraceae	*Ocotea odorifera*	KF420961	ITS
Lauraceae	*Ocotea odorifera*	KF420962	ITS
Lauraceae	*Ocotea odorifera*	KF421035	psbA-trnH
Lauraceae	*Ocotea odorifera*	KF421036	psbA-trnH
Lauraceae	*Ocotea odorifera*	KF421037	psbA-trnH
Lauraceae	*Ocotea odorifera*	KF421038	psbA-trnH
Lauraceae	*Ocotea odorifera*	KF555431	matK
Lauraceae	*Ocotea odorifera*	KF555432	matK
Lauraceae	*Ocotea odorifera*	KF555433	matK
Lauraceae	*Ocotea odorifera*	KF561951	rbcL
Lauraceae	*Ocotea odorifera*	KF561952	rbcL
Lauraceae	*Ocotea odorifera*	KF561953	rbcL
Lauraceae	*Ocotea porosa*	KF420956	ITS
Lauraceae	*Ocotea porosa*	KF420957	ITS
Lauraceae	*Ocotea porosa*	KF420958	ITS
Lauraceae	*Ocotea porosa*	KF420959	ITS
Lauraceae	*Ocotea porosa*	KF421039	psbA-trnH
Lauraceae	*Ocotea porosa*	KF421040	psbA-trnH
Lauraceae	*Ocotea porosa*	KF421041	psbA-trnH
Lauraceae	*Ocotea porosa*	KF555434	matK
Lauraceae	*Ocotea porosa*	KF555435	matK

**Family**	**Species**	**Accession**	**Barcode**
Lauraceae	*Ocotea porosa*	KF555436	matK
Lauraceae	*Ocotea porosa*	KF561954	rbcL
Lauraceae	*Ocotea porosa*	KF561955	rbcL
Lauraceae	*Ocotea porosa*	KF561956	rbcL
Lauraceae	*Ocotea puberula*	EU153870	matK
Lauraceae	*Ocotea puberula*	EU153871	matK
Lauraceae	*Ocotea puberula*	EU153872	matK
Lauraceae	*Ocotea puberula*	EU153929	rpoB
Lauraceae	*Ocotea puberula*	EU153930	rpoB
Lauraceae	*Ocotea puberula*	EU153931	rpoB
Lauraceae	*Ocotea puberula*	EU153985	psbA-trnH
Lauraceae	*Ocotea puberula*	EU153986	psbA-trnH
Lauraceae	*Ocotea puberula*	EU154044	rpoC1
Lauraceae	*Ocotea puberula*	EU154045	rpoC1
Lauraceae	*Ocotea puberula*	EU154046	rpoC1
Lauraceae	*Ocotea puberula*	GQ480391	ITS
Lauraceae	*Ocotea puberula*	GQ480392	ITS
Lauraceae	*Ocotea puberula*	GQ981818	rbcL
Lauraceae	*Ocotea puberula*	GQ982054	matK
Lauraceae	*Ocotea puberula*	GQ982304	psbA-trnH
Lauraceae	*Ocotea puberula*	KF420951	ITS
Lauraceae	*Ocotea puberula*	KF420952	ITS
Lauraceae	*Ocotea puberula*	KF420953	ITS
Lauraceae	*Ocotea puberula*	KF420954	ITS
Lauraceae	*Ocotea puberula*	KF420955	ITS
Lauraceae	*Ocotea puberula*	KF421042	psbA-trnH
Lauraceae	*Ocotea puberula*	KF421043	psbA-trnH
Lauraceae	*Ocotea puberula*	KF421044	psbA-trnH
Lauraceae	*Ocotea puberula*	KF421045	psbA-trnH
Lauraceae	*Ocotea puberula*	KF555437	matK
Lauraceae	*Ocotea puberula*	KF555438	matK
Lauraceae	*Ocotea puberula*	KF555439	matK
Lauraceae	*Ocotea puberula*	KF555440	matK
Lauraceae	*Ocotea puberula*	KF561957	rbcL
Lauraceae	*Ocotea puberula*	KF561958	rbcL

Family	Species	Accession	Barcode
Lauraceae	*Ocotea puberula*	KF561959	rbcL
Lauraceae	*Ocotea pulchella*	AF268740	trnL
Lauraceae	*Ocotea pulchella*	AF272312	ITS
Lauraceae	*Ocotea pulchella*	KF420948	ITS
Lauraceae	*Ocotea pulchella*	KF420949	ITS
Lauraceae	*Ocotea pulchella*	KF420950	ITS
Lauraceae	*Ocotea pulchella*	KF421046	psbA-trnH
Lauraceae	*Ocotea pulchella*	KF421047	psbA-trnH
Lauraceae	*Ocotea pulchella*	KF555441	matK
Lauraceae	*Ocotea pulchella*	KF555442	matK
Lauraceae	*Ocotea pulchella*	KF561960	rbcL
Lauraceae	*Ocotea pulchella*	KF561961	rbcL
Lauraceae	*Ocotea silvestris*	GQ480394	ITS
Lauraceae	*Ocotea tristis*	AF268742	trnL
Lauraceae	*Ocotea tristis*	AF272318	ITS
Lauraceae	*Ocotea velloziana*	GQ480395	ITS
Lauraceae	*Persea alba*	FM957820	ITS
Lauraceae	*Persea alba*	HQ697192	ITS
Lauraceae	*Persea americana*	AF268744	trnL
Lauraceae	*Persea americana*	AF268794	psbA-trnH
Lauraceae	*Persea americana*	AJ247179	matK
Lauraceae	*Persea americana*	AY337727	rbcL
Lauraceae	*Persea americana*	AY841592	rbcL
Lauraceae	*Persea americana*	AY841669	trnL
Lauraceae	*Persea americana*	HG963716	psbA-trnH
Lauraceae	*Persea americana*	HM019367	matK
Lauraceae	*Persea americana*	HM019437	psbA-trnH
Lauraceae	*Persea americana*	HM019507	rbcL
Lauraceae	*Persea americana*	JF966395-JF966417	psbA-trnH
Lauraceae	*Persea americana*	JF966437-JF966464	matK
Lauraceae	*Persea americana*	JF966471-JF966498	rpoC1
Lauraceae	*Persea americana*	JF966607-JF966634	rbcL
Lauraceae	*Persea americana*	JQ513882	psbA-trnH
Lauraceae	*Persea americana*	JQ588140-JQ588149	matK
Lauraceae	*Persea americana*	JQ592386-JQ592394	rbcL

**Family**	**Species**	**Accession**	**Barcode**
Lauraceae	*Persea americana*	JQ742021	trnL
Lauraceae	*Persea americana*	KF155327	psbA-trnH
Lauraceae	*Persea americana*	KF155401	trnL
Lauraceae	*Persea americana*	KF586677	trnL
Lauraceae	*Persea americana*	KF586695	trnL
Lauraceae	*Persea americana*	L14620	rbcL
Lauraceae	*Persea major*	GQ480396	ITS
Lauraceae	*Persea major*	HQ697197	ITS
Lauraceae	*Persea venosa*	FM957840	ITS
Lauraceae	*Persea willdenovii*	FM957843	ITS
Lauraceae	*Urbanodendron bahiense*	AF272338	ITS
Lecythidaceae	*Cariniana estrellensis*	AF077647	trnL
Lecythidaceae	*Cariniana estrellensis*	DQ417937	trnL
Lecythidaceae	*Cariniana legalis*	JN222194	ITS
Lecythidaceae	*Cariniana legalis*	Z80179	rbcL
Lecythidaceae	*Lecythis lanceolata*	DQ418020	trnL
Lecythidaceae	*Lecythis lanceolata*	JN221672	psbA-trnH
Lecythidaceae	*Lecythis lanceolata*	JN222191	ITS
Lecythidaceae	*Lecythis lanceolata*	JN222243	ITS
Lecythidaceae	*Lecythis pisonis*	DQ427110	trnL
Lecythidaceae	*Lecythis pisonis*	JN221670	psbA-trnH
Lecythidaceae	*Lecythis pisonis*	JN221682	psbA-trnH
Lecythidaceae	*Lecythis pisonis*	JN221697	psbA-trnH
Lecythidaceae	*Lecythis pisonis*	JN221709	psbA-trnH
Lecythidaceae	*Lecythis pisonis*	JN221723	psbA-trnH
Lecythidaceae	*Lecythis pisonis*	JN221942	trnL
Lecythidaceae	*Lecythis pisonis*	JN221943	trnL
Lecythidaceae	*Lecythis pisonis*	JN221944	trnL
Lecythidaceae	*Lecythis pisonis*	JN221955	trnL
Lecythidaceae	*Lecythis pisonis*	JN221969	trnL
Lecythidaceae	*Lecythis pisonis*	JN221970	trnL
Lecythidaceae	*Lecythis pisonis*	JN221971	trnL
Lecythidaceae	*Lecythis pisonis*	JN222116	ITS
Lecythidaceae	*Lecythis pisonis*	JN222119	ITS
Lecythidaceae	*Lecythis pisonis*	JN222120	ITS

**Family**	**Species**	**Accession**	**Barcode**
Lecythidaceae	*Lecythis pisonis*	JN222121	ITS
Lecythidaceae	*Lecythis pisonis*	JN222122	ITS
Lecythidaceae	*Lecythis pisonis*	JN222130	ITS
Lecythidaceae	*Lecythis pisonis*	JN222131	ITS
Lecythidaceae	*Lecythis pisonis*	JN222132	ITS
Lecythidaceae	*Lecythis pisonis*	JN222133	ITS
Lecythidaceae	*Lecythis pisonis*	JN222136	ITS
Lecythidaceae	*Lecythis pisonis*	JN222137	ITS
Lecythidaceae	*Lecythis pisonis*	JN222184	ITS
Lecythidaceae	*Lecythis pisonis*	JN222196	ITS
Lecythidaceae	*Lecythis pisonis*	JN222197	ITS
Lecythidaceae	*Lecythis pisonis*	JN222199	ITS
Lecythidaceae	*Lecythis pisonis*	JN222342	ITS
Loganiaceae	*Strychnos brasiliensis*	JF937956	ITS
Loganiaceae	*Strychnos pseudoquina*	JF938028	ITS
Loganiaceae	*Strychnos pseudoquina*	KF667924	rbcL
Loganiaceae	*Strychnos pseudoquina*	KF667926	rbcL
Loganiaceae	*Strychnos pseudoquina*	KF667931	rbcL
Loganiaceae	*Strychnos pseudoquina*	KF667934	rbcL
Loganiaceae	*Strychnos pseudoquina*	KF667938	rbcL
Loganiaceae	*Strychnos pseudoquina*	KF667943	rbcL
Loganiaceae	*Strychnos pseudoquina*	KF667947	rbcL
Loganiaceae	*Strychnos pseudoquina*	KF667964	matK
Loganiaceae	*Strychnos pseudoquina*	KF683522	matK
Loganiaceae	*Strychnos pseudoquina*	KF683523	matK
Loganiaceae	*Strychnos pseudoquina*	KF683524	matK
Loganiaceae	*Strychnos pseudoquina*	KF683529	rbcL
Loganiaceae	*Strychnos pseudoquina*	KF683530	rbcL
Loganiaceae	*Strychnos pseudoquina*	KF683531	rbcL
Lythraceae	*Diplusodon virgatus*	JN701285	ITS
Lythraceae	*Diplusodon virgatus*	JN701301	ITS
Lythraceae	*Diplusodon virgatus*	JN701363	psbA-trnH
Lythraceae	*Lafoensia pacari*	JN701292	ITS
Lythraceae	*Lafoensia pacari*	JN701314	psbA-trnH
Lythraceae	*Lafoensia pacari*	KF420995	ITS

**Family**	**Species**	**Accession**	**Barcode**
Lythraceae	*Lafoensia pacari*	KF421068	psbA-trnH
Lythraceae	*Lafoensia pacari*	KF421069	psbA-trnH
Lythraceae	*Lafoensia pacari*	KF555401	matK
Lythraceae	*Lafoensia pacari*	KF555402	matK
Lythraceae	*Lafoensia pacari*	KF561920	rbcL
Lythraceae	*Lafoensia pacari*	KF561921	rbcL
Magnoliaceae	*Magnolia ovata*	AB055537	matK
Magnoliaceae	*Magnolia ovata*	AB055563	psbA-trnH
Magnoliaceae	*Magnolia ovata*	L12666	rbcL
Malpighiaceae	*Banisteriopsis latifolia*	HQ247206	matK
Malpighiaceae	*Barnebya dispar*	AF351006	trnL
Malpighiaceae	*Barnebya dispar*	AJ402924	rbcL
Malpighiaceae	*Barnebya dispar*	AY137290	trnL
Malpighiaceae	*Barnebya dispar*	AY137314	ITS
Malpighiaceae	*Bunchosia pallescens*	KM197248	matK
Malpighiaceae	*Byrsonima basiloba*	HQ247236	matK
Malpighiaceae	*Byrsonima basiloba*	HQ247459	rbcL
Malpighiaceae	*Byrsonima coccolobifolia*	HQ247237	matK
Malpighiaceae	*Byrsonima coccolobifolia*	HQ247460	rbcL
Malpighiaceae	*Byrsonima crassifolia*	AB233794	matK
Malpighiaceae	*Byrsonima crassifolia*	AB233898	rbcL
Malpighiaceae	*Byrsonima crassifolia*	AF344535	matK
Malpighiaceae	*Byrsonima crassifolia*	AF350945	trnL
Malpighiaceae	*Byrsonima crassifolia*	AJ581433	matK
Malpighiaceae	*Byrsonima crassifolia*	DQ787393	ITS
Malpighiaceae	*Byrsonima crassifolia*	GQ429064	matK
Malpighiaceae	*Byrsonima crassifolia*	GQ429092	rpoC1
Malpighiaceae	*Byrsonima crassifolia*	GQ429122	psbA-trnH
Malpighiaceae	*Byrsonima crassifolia*	JQ588183	matK
Malpighiaceae	*Byrsonima crassifolia*	JQ588184	matK
Malpighiaceae	*Byrsonima crassifolia*	JQ588185	matK
Malpighiaceae	*Byrsonima crassifolia*	JQ588186	matK
Malpighiaceae	*Byrsonima crassifolia*	JQ588187	matK
Malpighiaceae	*Byrsonima crassifolia*	JQ588188	matK
Malpighiaceae	*Byrsonima crassifolia*	JQ588189	matK

Family	Species	Accession	Barcode
Malpighiaceae	*Byrsonima crassifolia*	JQ588190	matK
Malpighiaceae	*Byrsonima crassifolia*	JQ588191	matK
Malpighiaceae	*Byrsonima crassifolia*	JQ588192	matK
Malpighiaceae	*Byrsonima crassifolia*	JQ592443	rbcL
Malpighiaceae	*Byrsonima crassifolia*	JQ592444	rbcL
Malpighiaceae	*Byrsonima crassifolia*	JQ592445	rbcL
Malpighiaceae	*Byrsonima crassifolia*	JQ592446	rbcL
Malpighiaceae	*Byrsonima crassifolia*	JQ592447	rbcL
Malpighiaceae	*Byrsonima crassifolia*	JQ592448	rbcL
Malpighiaceae	*Byrsonima crassifolia*	JQ592449	rbcL
Malpighiaceae	*Byrsonima crassifolia*	JQ592450	rbcL
Malpighiaceae	*Byrsonima crassifolia*	JQ592451	rbcL
Malpighiaceae	*Byrsonima crassifolia*	JQ592452	rbcL
Malpighiaceae	*Byrsonima crassifolia*	JQ592453	rbcL
Malpighiaceae	*Byrsonima crassifolia*	JQ592454	rbcL
Malpighiaceae	*Byrsonima crassifolia*	JQ592455	rbcL
Malpighiaceae	*Byrsonima crassifolia*	JQ592456	rbcL
Malpighiaceae	*Byrsonima crassifolia*	JQ592457	rbcL
Malpighiaceae	*Byrsonima crassifolia*	JX661932	matK
Malpighiaceae	*Byrsonima crassifolia*	JX663464	rpoB
Malpighiaceae	*Byrsonima crassifolia*	JX664036	rbcL
Malpighiaceae	*Byrsonima crassifolia*	JX664925	rpoC1
Malpighiaceae	*Byrsonima crassifolia*	KJ751236	rbcL
Malpighiaceae	*Byrsonima crassifolia*	KR270510	matK
Malpighiaceae	*Byrsonima crassifolia*	L01892	rbcL
Malpighiaceae	*Byrsonima ligustrifolia*	KF981237	rbcL
Malpighiaceae	*Byrsonima ligustrifolia*	KF981328	matK
Malpighiaceae	*Heteropterys byrsonimifolia*	HQ247286	matK
Malpighiaceae	*Heteropterys byrsonimifolia*	HQ247497	rbcL
Malvaceae	*Apeiba tibourbou*	AJ233145	rbcL
Malvaceae	*Apeiba tibourbou*	GQ981667	rbcL
Malvaceae	*Apeiba tibourbou*	GQ981936	matK
Malvaceae	*Apeiba tibourbou*	GQ982148	psbA-trnH
Malvaceae	*Apeiba tibourbou*	HG963880	psbA-trnH
Malvaceae	*Apeiba tibourbou*	JQ589325	matK

**Family**	**Species**	**Accession**	**Barcode**
Malvaceae	*Apeiba tibourbou*	JQ589326	matK
Malvaceae	*Apeiba tibourbou*	JQ589327	matK
Malvaceae	*Bastardiopsis densiflora*	AY591815	ITS
Malvaceae	*Ceiba speciosa*	AF460191	ITS
Malvaceae	*Ceiba speciosa*	AJ233116	rbcL
Malvaceae	*Ceiba speciosa*	AY328148	trnL
Malvaceae	*Ceiba speciosa*	AY328182	rbcL
Malvaceae	*Ceiba speciosa*	HQ658388	ITS
Malvaceae	*Ceiba speciosa*	HQ696702	matK
Malvaceae	*Ceiba speciosa*	HQ696755	trnL
Malvaceae	*Eriotheca candolleana*	HQ658394	ITS
Malvaceae	*Eriotheca candolleana*	HQ696718	matK
Malvaceae	*Eriotheca candolleana*	HQ696772	trnL
Malvaceae	*Eriotheca gracilipes*	HQ696708	matK
Malvaceae	*Eriotheca gracilipes*	HQ696762	trnL
Malvaceae	*Eriotheca pentaphylla*	HQ696714	matK
Malvaceae	*Eriotheca pentaphylla*	HQ696768	trnL
Malvaceae	*Eriotheca pubescens*	HQ658397	ITS
Malvaceae	*Eriotheca pubescens*	HQ696709	matK
Malvaceae	*Eriotheca pubescens*	HQ696763	trnL
Malvaceae	*Eriotheca pubescens*	JX850031	matK
Malvaceae	*Guazuma crinita*	GU981727	rbcL
Malvaceae	*Guazuma ulmifolia*	AY083658	ITS
Malvaceae	*Guazuma ulmifolia*	GQ981753	rbcL
Malvaceae	*Guazuma ulmifolia*	GQ982003	matK
Malvaceae	*Guazuma ulmifolia*	GQ982236	psbA-trnH
Malvaceae	*Guazuma ulmifolia*	HG963541	psbA-trnH
Malvaceae	*Guazuma ulmifolia*	HM446807	rbcL
Malvaceae	*Guazuma ulmifolia*	HM446939	psbA-trnH
Malvaceae	*Guazuma ulmifolia*	HQ656780	trnL
Malvaceae	*Guazuma ulmifolia*	JQ589293	matK
Malvaceae	*Guazuma ulmifolia*	JQ589294	matK
Malvaceae	*Guazuma ulmifolia*	JQ589295	matK
Malvaceae	*Guazuma ulmifolia*	JQ589296	matK
Malvaceae	*Guazuma ulmifolia*	JQ589297	matK

Family	Species	Accession	Barcode
Malvaceae	*Guazuma ulmifolia*	JQ589298	matK
Malvaceae	*Guazuma ulmifolia*	JQ589299	matK
Malvaceae	*Guazuma ulmifolia*	JQ589300	matK
Malvaceae	*Guazuma ulmifolia*	JQ594204	rbcL
Malvaceae	*Guazuma ulmifolia*	JQ594205	rbcL
Malvaceae	*Guazuma ulmifolia*	JQ594206	rbcL
Malvaceae	*Guazuma ulmifolia*	JQ594207	rbcL
Malvaceae	*Guazuma ulmifolia*	JQ594208	rbcL
Malvaceae	*Guazuma ulmifolia*	JQ594209	rbcL
Malvaceae	*Guazuma ulmifolia*	JQ594210	rbcL
Malvaceae	*Guazuma ulmifolia*	JQ594211	rbcL
Malvaceae	*Guazuma ulmifolia*	KF724295	rbcL
Malvaceae	*Heliocarpus popayanensis*	AJ233153	rbcL
Malvaceae	*Heliocarpus popayanensis*	JQ589794	matK
Malvaceae	*Heliocarpus popayanensis*	JQ589795	matK
Malvaceae	*Heliocarpus popayanensis*	JQ589796	matK
Malvaceae	*Heliocarpus popayanensis*	JQ589797	matK
Malvaceae	*Heliocarpus popayanensis*	JQ589798	matK
Malvaceae	*Heliocarpus popayanensis*	JQ594802	rbcL
Malvaceae	*Heliocarpus popayanensis*	JQ594803	rbcL
Malvaceae	*Heliocarpus popayanensis*	JQ594804	rbcL
Malvaceae	*Heliocarpus popayanensis*	JQ594805	rbcL
Malvaceae	*Heliocarpus popayanensis*	JQ594806	rbcL
Malvaceae	*Luehea divaricata*	GU981728	rbcL
Malvaceae	*Luehea divaricata*	KF420987	ITS
Malvaceae	*Luehea divaricata*	KF420988	ITS
Malvaceae	*Luehea divaricata*	KF421092	psbA-trnH
Malvaceae	*Luehea divaricata*	KF421093	psbA-trnH
Malvaceae	*Luehea divaricata*	KF555406	matK
Malvaceae	*Luehea divaricata*	KF555407	matK
Malvaceae	*Luehea divaricata*	KF561925	rbcL
Malvaceae	*Luehea divaricata*	KF561926	rbcL
Malvaceae	*Pachira glabra*	HQ658393	ITS
Malvaceae	*Pachira glabra*	HQ696706	matK
Malvaceae	*Pachira glabra*	HQ696761	trnL

**Family**	**Species**	**Accession**	**Barcode**
Malvaceae	*Pseudobombax grandiflorum*	HQ658383	ITS
Malvaceae	*Pseudobombax grandiflorum*	HQ696698	matK
Malvaceae	*Pseudobombax grandiflorum*	HQ696750	trnL
Malvaceae	*Pseudobombax marginatum*	AF028521	ITS
Malvaceae	*Pseudobombax marginatum*	HQ658381	ITS
Malvaceae	*Pseudobombax marginatum*	HQ696696	matK
Malvaceae	*Pseudobombax marginatum*	HQ696748	trnL
Malvaceae	*Quararibea turbinata*	HM446740	matK
Malvaceae	*Quararibea turbinata*	HM446864	rbcL
Malvaceae	*Quararibea turbinata*	HM446994	psbA-trnH
Malvaceae	*Quararibea turbinata*	KJ012743	matK
Malvaceae	*Quararibea turbinata*	KJ082533	rbcL
Melastomataceae	*Leandra acutiflora*	EF418813	ITS
Melastomataceae	*Leandra acutiflora*	EU055689	ITS
Melastomataceae	*Leandra acutiflora*	GQ139280	atpF-atpH
Melastomataceae	*Leandra acutiflora*	GQ139337	psbK-psbI
Melastomataceae	*Leandra acutiflora*	KR062213	atpF-atpH
Melastomataceae	*Leandra acutiflora*	KR062482	ITS
Melastomataceae	*Leandra acutiflora*	KR062590	psbK-psbI
Melastomataceae	*Leandra amplexicaulis*	EU055685	ITS
Melastomataceae	*Leandra amplexicaulis*	GQ139281	atpF-atpH
Melastomataceae	*Leandra amplexicaulis*	GQ139338	psbK-psbI
Melastomataceae	*Leandra amplexicaulis*	KR062217	atpF-atpH
Melastomataceae	*Leandra amplexicaulis*	KR062218	atpF-atpH
Melastomataceae	*Leandra amplexicaulis*	KR062485	ITS
Melastomataceae	*Leandra amplexicaulis*	KR062486	ITS
Melastomataceae	*Leandra aurea*	KR062220	atpF-atpH
Melastomataceae	*Leandra aurea*	KR062221	atpF-atpH
Melastomataceae	*Leandra aurea*	KR062488	ITS
Melastomataceae	*Leandra aurea*	KR062489	ITS
Melastomataceae	*Leandra aurea*	KR062592	psbK-psbI
Melastomataceae	*Leandra barbinervis*	EF418817	ITS
Melastomataceae	*Leandra barbinervis*	KR062223	atpF-atpH
Melastomataceae	*Leandra barbinervis*	KR062224	atpF-atpH
Melastomataceae	*Leandra brackenridgei*	KR062226	atpF-atpH

Family	Species	Accession	Barcode
Melastomataceae	*Leandra brackenridgei*	KR062490	ITS
Melastomataceae	*Leandra brackenridgei*	KR062593	psbK-psbI
Melastomataceae	*Leandra carassana*	EU055688	ITS
Melastomataceae	*Leandra carassana*	KR062231	atpF-atpH
Melastomataceae	*Leandra carassana*	KR062232	atpF-atpH
Melastomataceae	*Leandra carassana*	KR062233	atpF-atpH
Melastomataceae	*Leandra carassana*	KR062234	atpF-atpH
Melastomataceae	*Leandra carassana*	KR062494	ITS
Melastomataceae	*Leandra carassana*	KR062495	ITS
Melastomataceae	*Leandra carassana*	KR062597	psbK-psbI
Melastomataceae	*Leandra carassana*	KR062598	psbK-psbI
Melastomataceae	*Leandra clidemioides*	EF418820	ITS
Melastomataceae	*Leandra clidemioides*	KR062237	atpF-atpH
Melastomataceae	*Leandra fallax*	KR062251	atpF-atpH
Melastomataceae	*Leandra fallax*	KR062252	atpF-atpH
Melastomataceae	*Leandra fallax*	KR062511	ITS
Melastomataceae	*Leandra fallax*	KR062512	ITS
Melastomataceae	*Leandra fallax*	KR062607	psbK-psbI
Melastomataceae	*Leandra fragilis*	EF418830	ITS
Melastomataceae	*Leandra lancifolia*	KR062273	atpF-atpH
Melastomataceae	*Leandra lancifolia*	KR062524	ITS
Melastomataceae	*Leandra lancifolia*	KR062617	psbK-psbI
Melastomataceae	*Leandra melastomoides*	EF418841	ITS
Melastomataceae	*Leandra melastomoides*	KR062277	atpF-atpH
Melastomataceae	*Leandra melastomoides*	KR062278	atpF-atpH
Melastomataceae	*Leandra melastomoides*	KR062279	atpF-atpH
Melastomataceae	*Leandra melastomoides*	KR062280	atpF-atpH
Melastomataceae	*Leandra melastomoides*	KR062281	atpF-atpH
Melastomataceae	*Leandra melastomoides*	KR062282	atpF-atpH
Melastomataceae	*Leandra melastomoides*	KR062528	ITS
Melastomataceae	*Leandra melastomoides*	KR062529	ITS
Melastomataceae	*Leandra melastomoides*	KR062530	ITS
Melastomataceae	*Leandra melastomoides*	KR062531	ITS
Melastomataceae	*Leandra melastomoides*	KR062620	psbK-psbI
Melastomataceae	*Leandra melastomoides*	KR062621	psbK-psbI

Family	Species	Accession	Barcode
Melastomataceae	*Leandra multiplinervis*	KR062287	atpF-atpH
Melastomataceae	*Leandra multiplinervis*	KR062288	atpF-atpH
Melastomataceae	*Leandra multiplinervis*	KR062535	ITS
Melastomataceae	*Leandra multiplinervis*	KR062624	psbK-psbI
Melastomataceae	*Leandra purpureovillosa*	KR062302	atpF-atpH
Melastomataceae	*Leandra purpureovillosa*	KR062303	atpF-atpH
Melastomataceae	*Leandra purpureovillosa*	KR062546	ITS
Melastomataceae	*Leandra purpureovillosa*	KR062630	psbK-psbI
Melastomataceae	*Leandra quinquedentata*	EF418867	ITS
Melastomataceae	*Leandra quinquedentata*	GQ139286	atpF-atpH
Melastomataceae	*Leandra quinquedentata*	GQ139344	psbK-psbI
Melastomataceae	*Leandra quinquedentata*	KR062304	atpF-atpH
Melastomataceae	*Leandra quinquedentata*	KR062547	ITS
Melastomataceae	*Leandra quinquedentata*	KR062631	psbK-psbI
Melastomataceae	*Leandra quinquenodis*	KR062305	atpF-atpH
Melastomataceae	*Leandra regnellii*	EF418851	ITS
Melastomataceae	*Leandra regnellii*	KR062308	atpF-atpH
Melastomataceae	*Leandra regnellii*	KR062309	atpF-atpH
Melastomataceae	*Leandra regnellii*	KR062310	atpF-atpH
Melastomataceae	*Leandra regnellii*	KR062549	ITS
Melastomataceae	*Leandra regnellii*	KR062550	ITS
Melastomataceae	*Leandra regnellii*	KR062633	psbK-psbI
Melastomataceae	*Leandra regnellii*	KR062634	psbK-psbI
Melastomataceae	*Leandra tetraquetra*	EF418864	ITS
Melastomataceae	*Leandra tetraquetra*	KR062324	atpF-atpH
Melastomataceae	*Leandra tetraquetra*	KR062325	atpF-atpH
Melastomataceae	*Leandra tetraquetra*	KR062561	ITS
Melastomataceae	*Leandra umbellata*	KR062332	atpF-atpH
Melastomataceae	*Leandra umbellata*	KR062333	atpF-atpH
Melastomataceae	*Leandra umbellata*	KR062565	ITS
Melastomataceae	*Leandra umbellata*	KR062566	ITS
Melastomataceae	*Leandra umbellata*	KR062644	psbK-psbI
Melastomataceae	*Leandra variabilis*	EF418824	ITS
Melastomataceae	*Leandra variabilis*	KR062334	atpF-atpH
Melastomataceae	*Leandra variabilis*	KR062335	atpF-atpH

Family	Species	Accession	Barcode
Melastomataceae	*Leandra variabilis*	KR062567	ITS
Melastomataceae	*Leandra vesiculosa*	KR062336	atpF-atpH
Melastomataceae	*Leandra vesiculosa*	KR062568	ITS
Melastomataceae	*Leandra vesiculosa*	KR062645	psbK-psbI
Melastomataceae	*Macairea radula*	EU711394	rbcL
Melastomataceae	*Macairea radula*	JQ730095	ITS
Melastomataceae	*Macairea radula*	JQ730514	psbK-psbI
Melastomataceae	*Miconia affinis*	EF418879	ITS
Melastomataceae	*Miconia affinis*	GQ981798	rbcL
Melastomataceae	*Miconia affinis*	GQ982042	matK
Melastomataceae	*Miconia affinis*	GQ982284	psbA-trnH
Melastomataceae	*Miconia albicans*	EF418880	ITS
Melastomataceae	*Miconia brasiliensis*	EU055727	ITS
Melastomataceae	*Miconia brasiliensis*	GQ139292	atpF-atpH
Melastomataceae	*Miconia brasiliensis*	GQ139307	ITS
Melastomataceae	*Miconia brasiliensis*	GQ139350	psbK-psbI
Melastomataceae	*Miconia brunnea*	EU055730	ITS
Melastomataceae	*Miconia budlejoides*	EU055732	ITS
Melastomataceae	*Miconia cabucu*	EU055734	ITS
Melastomataceae	*Miconia calvescens*	EU055736	ITS
Melastomataceae	*Miconia chamissois*	EU055748	ITS
Melastomataceae	*Miconia chartacea*	EU055749	ITS
Melastomataceae	*Miconia cinerascens*	EU055751	ITS
Melastomataceae	*Miconia cinerascens*	EU055752	ITS
Melastomataceae	*Miconia cinnamomifolia*	EU055753	ITS
Melastomataceae	*Miconia collatata*	EU055754	ITS
Melastomataceae	*Miconia cubatanensis*	EU055761	ITS
Melastomataceae	*Miconia discolor*	EU055767	ITS
Melastomataceae	*Miconia dodecandra*	AY460506	ITS
Melastomataceae	*Miconia dodecandra*	EU055769	ITS
Melastomataceae	*Miconia dodecandra*	EU055770	ITS
Melastomataceae	*Miconia dodecandra*	EU711396	rbcL
Melastomataceae	*Miconia dodecandra*	FJ358429	ITS
Melastomataceae	*Miconia dodecandra*	JQ730527	psbK-psbI
Melastomataceae	*Miconia dodecandra*	KM495208	ITS

Family	Species	Accession	Barcode
Melastomataceae	*Miconia fasciculata*	EU055774	ITS
Melastomataceae	*Miconia hyemalis*	EU055782	ITS
Melastomataceae	*Miconia ibaguensis*	EU055785	ITS
Melastomataceae	*Miconia inconspicua*	EU055786	ITS
Melastomataceae	*Miconia jucunda*	EU055789	ITS
Melastomataceae	*Miconia latecrenata*	EU055790	ITS
Melastomataceae	*Miconia lepidota*	EU055792	ITS
Melastomataceae	*Miconia ligustroides*	EU055794	ITS
Melastomataceae	*Miconia longicuspis*	EU055796	ITS
Melastomataceae	*Miconia lymanii*	EU055800	ITS
Melastomataceae	*Miconia minutiflora*	AY460517	ITS
Melastomataceae	*Miconia minutiflora*	EU055805	ITS
Melastomataceae	*Miconia nervosa*	GQ981804	rbcL
Melastomataceae	*Miconia nervosa*	GQ982290	psbA-trnH
Melastomataceae	*Miconia paniculata*	EU055771	ITS
Melastomataceae	*Miconia pepericarpa*	EU055814	ITS
Melastomataceae	*Miconia petropolitana*	EU055815	ITS
Melastomataceae	*Miconia polyandra*	EU055819	ITS
Melastomataceae	*Miconia prasina*	AY460520	ITS
Melastomataceae	*Miconia prasina*	HM446830	rbcL
Melastomataceae	*Miconia prasina*	HM446962	psbA-trnH
Melastomataceae	*Miconia prasina*	KF724280	rbcL
Melastomataceae	*Miconia prasina*	KJ082427	rbcL
Melastomataceae	*Miconia prasina*	KJ426826	psbA-trnH
Melastomataceae	*Miconia pusilliflora*	EU055783	ITS
Melastomataceae	*Miconia pusilliflora*	EU055822	ITS
Melastomataceae	*Miconia pusilliflora*	EU055826	ITS
Melastomataceae	*Miconia rubiginosa*	AY460525	ITS
Melastomataceae	*Miconia sclerophylla*	EU055835	ITS
Melastomataceae	*Miconia sellowiana*	EU055836	ITS
Melastomataceae	*Miconia serrulata*	AY460535	ITS
Melastomataceae	*Miconia serrulata*	KJ082429	rbcL
Melastomataceae	*Miconia serrulata*	KJ082430	rbcL
Melastomataceae	*Miconia serrulata*	KJ426828	psbA-trnH
Melastomataceae	*Miconia serrulata*	KJ426829	psbA-trnH

Family	Species	Accession	Barcode
Melastomataceae	*Miconia stenostachya*	EU055843	ITS
Melastomataceae	*Miconia theizans*	AY460533	ITS
Melastomataceae	*Miconia theizans*	EU055849	ITS
Melastomataceae	*Miconia trianae*	EU055851	ITS
Melastomataceae	*Miconia tristis*	EU055855	ITS
Melastomataceae	*Miconia valtheri*	EU055857	ITS
Melastomataceae	*Miconia willdenowii*	EU055858	ITS
Melastomataceae	*Mouriri myrtilloides*	GQ981807	rbcL
Melastomataceae	*Mouriri myrtilloides*	GQ982293	psbA-trnH
Melastomataceae	*Mouriri myrtilloides*	JQ588330	matK
Melastomataceae	*Mouriri myrtilloides*	JQ588331	matK
Melastomataceae	*Mouriri myrtilloides*	JQ592687	rbcL
Melastomataceae	*Mouriri myrtilloides*	JQ592688	rbcL
Melastomataceae	*Mouriri myrtilloides*	JQ592689	rbcL
Melastomataceae	*Mouriri myrtilloides*	JQ592690	rbcL
Melastomataceae	*Mouriri myrtilloides*	JQ592691	rbcL
Melastomataceae	*Ossaea angustifolia*	GQ139294	atpF-atpH
Melastomataceae	*Ossaea angustifolia*	GQ139309	ITS
Melastomataceae	*Ossaea angustifolia*	GQ139352	psbK-psbI
Melastomataceae	*Ossaea angustifolia*	KR062342	atpF-atpH
Melastomataceae	*Ossaea angustifolia*	KR062343	atpF-atpH
Melastomataceae	*Ossaea angustifolia*	KR062572	ITS
Melastomataceae	*Ossaea angustifolia*	KR062573	ITS
Melastomataceae	*Ossaea angustifolia*	KR062650	psbK-psbI
Melastomataceae	*Ossaea marginata*	KR062354	atpF-atpH
Melastomataceae	*Ossaea marginata*	KR062582	ITS
Melastomataceae	*Ossaea sanguinea*	EU055865	ITS
Melastomataceae	*Ossaea sanguinea*	GQ139353	psbK-psbI
Melastomataceae	*Ossaea sanguinea*	KR062355	atpF-atpH
Melastomataceae	*Tibouchina arborea*	JQ730152	ITS
Melastomataceae	*Tibouchina arborea*	JQ730575	psbK-psbI
Melastomataceae	*Tibouchina candolleana*	JQ730164	ITS
Melastomataceae	*Tibouchina candolleana*	JQ730585	psbK-psbI
Melastomataceae	*Tibouchina clavata*	JQ730172	ITS
Melastomataceae	*Tibouchina clavata*	JQ730593	psbK-psbI

Family	Species	Accession	Barcode
Melastomataceae	*Tibouchina estrellensis*	JQ730180	ITS
Melastomataceae	*Tibouchina estrellensis*	JQ730600	psbK-psbI
Melastomataceae	*Tibouchina fothergillae*	JQ730182	ITS
Melastomataceae	*Tibouchina fothergillae*	JQ730602	psbK-psbI
Melastomataceae	*Tibouchina granulosa*	FJ628144	ITS
Melastomataceae	*Tibouchina granulosa*	FJ628148	trnL
Melastomataceae	*Tibouchina granulosa*	GQ465909	trnL
Melastomataceae	*Tibouchina granulosa*	GQ465910	trnL
Melastomataceae	*Tibouchina granulosa*	GQ465915	ITS
Melastomataceae	*Tibouchina granulosa*	GQ465916	ITS
Melastomataceae	*Tibouchina granulosa*	JQ730191	ITS
Melastomataceae	*Tibouchina granulosa*	JQ730611	psbK-psbI
Melastomataceae	*Tibouchina mutabilis*	JQ730213	ITS
Melastomataceae	*Tibouchina mutabilis*	JQ730633	psbK-psbI
Melastomataceae	*Tibouchina pulchra*	JQ730222	ITS
Melastomataceae	*Tibouchina pulchra*	JQ730642	psbK-psbI
Melastomataceae	*Tibouchina sellowiana*	JQ730227	ITS
Melastomataceae	*Tibouchina sellowiana*	JQ730647	psbK-psbI
Melastomataceae	*Tibouchina stenocarpa*	JQ730233	ITS
Melastomataceae	*Tibouchina stenocarpa*	JQ730653	psbK-psbI
Melastomataceae	*Tibouchina trichopoda*	JQ730235	ITS
Melastomataceae	*Tibouchina trichopoda*	JQ730655	psbK-psbI
Melastomataceae	*Tococa guianensis*	AM235650	rbcL
Melastomataceae	*Tococa guianensis*	AY460554	ITS
Melastomataceae	*Tococa guianensis*	EU055895	ITS
Melastomataceae	*Trembleya parviflora*	AY553746	ITS
Melastomataceae	*Trembleya parviflora*	JQ730242	ITS
Melastomataceae	*Trembleya parviflora*	JQ730663	psbK-psbI
Meliaceae	*Cabralea canjerana*	DQ238055	rbcL
Meliaceae	*Cabralea canjerana*	DQ861617	ITS
Meliaceae	*Cabralea canjerana*	KF421012	ITS
Meliaceae	*Cabralea canjerana*	KF421013	ITS
Meliaceae	*Cabralea canjerana*	KF421080	psbA-trnH
Meliaceae	*Cabralea canjerana*	KF421081	psbA-trnH
Meliaceae	*Cabralea canjerana*	KF555384	matK

**Family**	**Species**	**Accession**	**Barcode**
Meliaceae	*Cabralea canjerana*	KF555385	matK
Meliaceae	*Cabralea canjerana*	KF561902	rbcL
Meliaceae	*Cabralea canjerana*	KF561903	rbcL
Meliaceae	*Cabralea canjerana*	KR364500	ycf1
Meliaceae	*Cedrela fissilis*	EU853783	trnL
Meliaceae	*Cedrela fissilis*	FJ462475	ITS
Meliaceae	*Cedrela fissilis*	FJ518893	ITS
Meliaceae	*Cedrela fissilis*	GU295824	rpoC1
Meliaceae	*Cedrela fissilis*	HM368493	rpoB
Meliaceae	*Cedrela fissilis*	HM368496	rpoB
Meliaceae	*Cedrela fissilis*	HM368497	rpoB
Meliaceae	*Cedrela fissilis*	HQ291769	rpoC1
Meliaceae	*Cedrela fissilis*	JF922130-JF922156	trnL
Meliaceae	*Cedrela fissilis*	JF922184-JF922260	ITS
Meliaceae	*Cedrela fissilis*	KC155960	ITS
Meliaceae	*Cedrela fissilis*	KC155980	psbA-trnH
Meliaceae	*Cedrela fissilis*	KF421008	ITS
Meliaceae	*Cedrela fissilis*	KF421082	psbA-trnH
Meliaceae	*Cedrela fissilis*	KF555388	matK
Meliaceae	*Cedrela fissilis*	KF561907	rbcL
Meliaceae	*Cedrela fissilis*	KF840443-KF840449	ITS
Meliaceae	*Cedrela fissilis*	KM408379-KM408386	psbA-trnH
Meliaceae	*Cedrela odorata*	DQ861606	ITS
Meliaceae	*Cedrela odorata*	FJ462463	ITS
Meliaceae	*Cedrela odorata*	FJ462464	ITS
Meliaceae	*Cedrela odorata*	FJ462467	ITS
Meliaceae	*Cedrela odorata*	FJ462468	ITS
Meliaceae	*Cedrela odorata*	FJ462471	ITS
Meliaceae	*Cedrela odorata*	FJ462477	ITS
Meliaceae	*Cedrela odorata*	GU338247	ITS
Meliaceae	*Cedrela odorata*	JN112854-JN112874	ITS
Meliaceae	*Cedrela odorata*	JN112875	ITS
Meliaceae	*Cedrela odorata*	KF840436-KF840442	ITS
Meliaceae	*Cedrela odorata*	AY128182	matK
Meliaceae	*Cedrela odorata*	GQ981959	matK

Family	Species	Accession	Barcode
Meliaceae	*Cedrela odorata*	JQ588333	matK
Meliaceae	*Cedrela odorata*	JQ588334	matK
Meliaceae	*Cedrela odorata*	JQ588335	matK
Meliaceae	*Cedrela odorata*	AB057482	psbA-trnH
Meliaceae	*Cedrela odorata*	GQ982176	psbA-trnH
Meliaceae	*Cedrela odorata*	HG963851	psbA-trnH
Meliaceae	*Cedrela odorata*	JN112886-JN112897	psbA-trnH
Meliaceae	*Cedrela odorata*	KC155976	psbA-trnH
Meliaceae	*Cedrela odorata*	KC155977	psbA-trnH
Meliaceae	*Cedrela odorata*	KM408359-KM408366	psbA-trnH
Meliaceae	*Cedrela odorata*	AJ402938	rbcL
Meliaceae	*Cedrela odorata*	AY128220	rbcL
Meliaceae	*Cedrela odorata*	GQ981695	rbcL
Meliaceae	*Cedrela odorata*	JQ592710-JQ592715	rbcL
Meliaceae	*Cedrela odorata*	HM368484	rpoB
Meliaceae	*Cedrela odorata*	HM368488	rpoB
Meliaceae	*Cedrela odorata*	HM368489	rpoB
Meliaceae	*Cedrela odorata*	HM368490	rpoB
Meliaceae	*Cedrela odorata*	GU295816	rpoC1
Meliaceae	*Cedrela odorata*	GU295818	rpoC1
Meliaceae	*Cedrela odorata*	GU295819	rpoC1
Meliaceae	*Cedrela odorata*	GU295821	rpoC1
Meliaceae	*Cedrela odorata*	HQ291774	rpoC1
Meliaceae	*Cedrela odorata*	AB057455	trnL
Meliaceae	*Cedrela odorata*	AB057509	trnL
Meliaceae	*Guarea guidonia*	HE653741	ITS
Meliaceae	*Guarea guidonia*	HM446695	matK
Meliaceae	*Guarea guidonia*	AB057506	psbA-trnH
Meliaceae	*Guarea guidonia*	GQ982234	psbA-trnH
Meliaceae	*Guarea guidonia*	HM446937	psbA-trnH
Meliaceae	*Guarea guidonia*	GQ981751	rbcL
Meliaceae	*Guarea guidonia*	HM446805	rbcL
Meliaceae	*Guarea guidonia*	JQ626153	rbcL
Meliaceae	*Guarea guidonia*	AB057479	trnL
Meliaceae	*Guarea guidonia*	AB057532	trnL

Family	Species	Accession	Barcode
Meliaceae	*Guarea kunthiana*	HE653760	ITS
Meliaceae	*Guarea macrophylla*	HE653729	ITS
Meliaceae	*Guarea macrophylla*	AB057507	psbA-trnH
Meliaceae	*Guarea macrophylla*	AB057480	trnL
Meliaceae	*Guarea macrophylla*	AB057534	trnL
Meliaceae	*Guarea macrophylla*	EU853797	trnL
Meliaceae	*Melia azedarach*	KP675797-KP675815	atpF-atpH
Meliaceae	*Melia azedarach*	AY695595	ITS
Meliaceae	*Melia azedarach*	FJ518900	ITS
Meliaceae	*Melia azedarach*	GQ434557	ITS
Meliaceae	*Melia azedarach*	JF421516	ITS
Meliaceae	*Melia azedarach*	JF421517	ITS
Meliaceae	*Melia azedarach*	JX856475	ITS
Meliaceae	*Melia azedarach*	JX856578	ITS
Meliaceae	*Melia azedarach*	KP675777-KP675795	ITS
Meliaceae	*Melia azedarach*	AY128193	matK
Meliaceae	*Melia azedarach*	EF489117	matK
Meliaceae	*Melia azedarach*	EU042834	matK
Meliaceae	*Melia azedarach*	FM179921	matK
Meliaceae	*Melia azedarach*	GU134981	matK
Meliaceae	*Melia azedarach*	JX517878	matK
Meliaceae	*Melia azedarach*	KP089161	matK
Meliaceae	*Melia azedarach*	KP675817-KP675835	matK
Meliaceae	*Melia azedarach*	AB057508	psbA-trnH
Meliaceae	*Melia azedarach*	GQ435159	psbA-trnH
Meliaceae	*Melia azedarach*	GQ435160	psbA-trnH
Meliaceae	*Melia azedarach*	GU135311	psbA-trnH
Meliaceae	*Melia azedarach*	JX856906	psbA-trnH
Meliaceae	*Melia azedarach*	JX856907	psbA-trnH
Meliaceae	*Melia azedarach*	JX856908	psbA-trnH
Meliaceae	*Melia azedarach*	KP675857-KP675875	psbA-trnH
Meliaceae	*Melia azedarach*	KP675837-KP675855	psbK-psbI
Meliaceae	*Melia azedarach*	AY128234	rbcL
Meliaceae	*Melia azedarach*	EU042973	rbcL
Meliaceae	*Melia azedarach*	FN599453	rbcL

**Family**	**Species**	**Accession**	**Barcode**
Meliaceae	*Melia azedarach*	GU135144	rbcL
Meliaceae	*Melia azedarach*	JX571865	rbcL
Meliaceae	*Melia azedarach*	JX856725	rbcL
Meliaceae	*Melia azedarach*	JX856726	rbcL
Meliaceae	*Melia azedarach*	JX905969	rbcL
Meliaceae	*Melia azedarach*	KF496754	rbcL
Meliaceae	*Melia azedarach*	KM895647	rbcL
Meliaceae	*Melia azedarach*	KP675877-KP675895	rbcL
Meliaceae	*Melia azedarach*	AB057481	trnL
Meliaceae	*Melia azedarach*	AB057535	trnL
Meliaceae	*Melia azedarach*	AB817682	trnL
Meliaceae	*Melia azedarach*	EF489265	trnL
Meliaceae	*Melia azedarach*	FM179536	trnL
Meliaceae	*Melia azedarach*	FN599481	trnL
Meliaceae	*Melia azedarach*	KP088396	ycf1
Meliaceae	*Melia azedarach*	KR364524	ycf1
Meliaceae	*Trichilia elegans*	KP055584	trnL
Meliaceae	*Trichilia hirta*	KJ012810	matK
Meliaceae	*Trichilia hirta*	KJ426979	psbA-trnH
Meliaceae	*Trichilia hirta*	KJ082621	rbcL
Meliaceae	*Trichilia martiana*	JQ588363	matK
Meliaceae	*Trichilia martiana*	JQ588364	matK
Meliaceae	*Trichilia martiana*	JQ588365	matK
Meliaceae	*Trichilia martiana*	JQ588366	matK
Meliaceae	*Trichilia martiana*	JQ588367	matK
Meliaceae	*Trichilia martiana*	JQ592429	rbcL
Meliaceae	*Trichilia martiana*	JQ592749	rbcL
Meliaceae	*Trichilia martiana*	JQ592750	rbcL
Meliaceae	*Trichilia martiana*	JQ592751	rbcL
Meliaceae	*Trichilia martiana*	JQ592752	rbcL
Meliaceae	*Trichilia martiana*	JQ592753	rbcL
Meliaceae	*Trichilia martiana*	JQ592754	rbcL
Meliaceae	*Trichilia pallida*	FJ037840	ITS
Meliaceae	*Trichilia pallida*	FJ514739	matK
Meliaceae	*Trichilia pallida*	GQ982117	matK

Family	Species	Accession	Barcode
Meliaceae	*Trichilia pallida*	HM446750	matK
Meliaceae	*Trichilia pallida*	JQ626491	matK
Meliaceae	*Trichilia pallida*	FJ039005	psbA-trnH
Meliaceae	*Trichilia pallida*	GQ428732	psbA-trnH
Meliaceae	*Trichilia pallida*	GQ982392	psbA-trnH
Meliaceae	*Trichilia pallida*	HM447009	psbA-trnH
Meliaceae	*Trichilia pallida*	FJ038116	rbcL
Meliaceae	*Trichilia pallida*	FJ038117	rbcL
Meliaceae	*Trichilia pallida*	GQ981904	rbcL
Meliaceae	*Trichilia pallida*	HM446879	rbcL
Meliaceae	*Trichilia pallida*	JQ626046	rbcL
Meliaceae	*Trichilia pallida*	FJ038338	rpoB
Meliaceae	*Trichilia pallida*	FJ038339	rpoB
Meliaceae	*Trichilia pallida*	FJ038715	rpoC1
Meliaceae	*Trichilia pallida*	FJ038716	rpoC1
Meliaceae	*Trichilia pallida*	FJ039159	trnL
Monimiaceae	*Hennecartia omphalandra*	GU177653	ITS
Monimiaceae	*Hennecartia omphalandra*	AF022950	rbcL
Monimiaceae	*Hennecartia omphalandra*	AF040682	trnL
Monimiaceae	*Hennecartia omphalandra*	KF586697	trnL
Monimiaceae	*Macrotorus utriculatus*	GU177655	ITS
Monimiaceae	*Macrotorus utriculatus*	GU177702	trnL
Monimiaceae	*Mollinedia ovata*	GU177657	ITS
Monimiaceae	*Mollinedia ovata*	AF050218	rbcL
Monimiaceae	*Mollinedia ovata*	AF040686	trnL
Monimiaceae	*Mollinedia schottiana*	GU177658	ITS
Monimiaceae	*Mollinedia schottiana*	GU177704	trnL
Monimiaceae	*Mollinedia widgrenii*	GU177659	ITS
Monimiaceae	*Mollinedia widgrenii*	GU177705	trnL
Moraceae	*Artocarpus altilis*	JQ774147	atpF-atpH
Moraceae	*Artocarpus altilis*	FJ917023	ITS
Moraceae	*Artocarpus altilis*	FJ917055	ITS
Moraceae	*Artocarpus altilis*	FJ917056	ITS
Moraceae	*Artocarpus altilis*	FJ917057	ITS
Moraceae	*Artocarpus altilis*	KM234120	ITS

**Family**	**Species**	**Accession**	**Barcode**
Moraceae	*Artocarpus altilis*	HM446658	matK
Moraceae	*Artocarpus altilis*	KJ767843	matK
Moraceae	*Artocarpus altilis*	KJ767844	matK
Moraceae	*Artocarpus altilis*	KJ767845	matK
Moraceae	*Artocarpus altilis*	KJ767846	matK
Moraceae	*Artocarpus altilis*	HM446889	psbA-trnH
Moraceae	*Artocarpus altilis*	AF500345	rbcL
Moraceae	*Artocarpus altilis*	HM446760	rbcL
Moraceae	*Artocarpus altilis*	JF738376	rbcL
Moraceae	*Artocarpus altilis*	JQ592778	rbcL
Moraceae	*Artocarpus altilis*	JQ592779	rbcL
Moraceae	*Artocarpus altilis*	JQ592780	rbcL
Moraceae	*Artocarpus altilis*	JQ592781	rbcL
Moraceae	*Artocarpus altilis*	KJ767821	rbcL
Moraceae	*Artocarpus altilis*	KJ767822	rbcL
Moraceae	*Artocarpus altilis*	KJ767823	rbcL
Moraceae	*Artocarpus altilis*	KJ767824	rbcL
Moraceae	*Artocarpus altilis*	KJ767825	rbcL
Moraceae	*Artocarpus altilis*	AF501600	trnL
Moraceae	*Artocarpus altilis*	FJ917087	trnL
Moraceae	*Artocarpus altilis*	FJ917116	trnL
Moraceae	*Artocarpus altilis*	FJ917117	trnL
Moraceae	*Artocarpus altilis*	FJ917118	trnL
Moraceae	*Artocarpus altilis*	KT032208	trnL
Moraceae	*Artocarpus heterophyllus*	FJ917039	ITS
Moraceae	*Artocarpus heterophyllus*	FJ917052	ITS
Moraceae	*Artocarpus heterophyllus*	JX856537	ITS
Moraceae	*Artocarpus heterophyllus*	KT002551	ITS
Moraceae	*Artocarpus heterophyllus*	AB981765	rbcL
Moraceae	*Artocarpus heterophyllus*	JX856635	rbcL
Moraceae	*Artocarpus heterophyllus*	KF724291	rbcL
Moraceae	*Artocarpus heterophyllus*	AJ390376	trnL
Moraceae	*Artocarpus heterophyllus*	FJ917103	trnL
Moraceae	*Artocarpus heterophyllus*	FJ917113	trnL
Moraceae	*Artocarpus heterophyllus*	KT032196	trnL

**Family**	**Species**	**Accession**	**Barcode**
Moraceae	*Brosimum guianense*	AY635481	ITS
Moraceae	*Brosimum guianense*	AY635482	ITS
Moraceae	*Brosimum guianense*	AY635483	ITS
Moraceae	*Brosimum guianense*	AY635484	ITS
Moraceae	*Brosimum guianense*	AY635485	ITS
Moraceae	*Brosimum guianense*	AY635486	ITS
Moraceae	*Brosimum guianense*	AY635487	ITS
Moraceae	*Brosimum guianense*	AY635488	ITS
Moraceae	*Brosimum guianense*	AY635489	ITS
Moraceae	*Brosimum guianense*	AY635490	ITS
Moraceae	*Brosimum guianense*	AY635491	ITS
Moraceae	*Brosimum guianense*	AY635492	ITS
Moraceae	*Brosimum guianense*	FJ037845	ITS
Moraceae	*Brosimum guianense*	GQ981948	matK
Moraceae	*Brosimum guianense*	JQ626530	matK
Moraceae	*Brosimum guianense*	FJ039011	psbA-trnH
Moraceae	*Brosimum guianense*	GQ982163	psbA-trnH
Moraceae	*Brosimum guianense*	JX997334	psbA-trnH
Moraceae	*Brosimum guianense*	JX997335	psbA-trnH
Moraceae	*Brosimum guianense*	JX997336	psbA-trnH
Moraceae	*Brosimum guianense*	JX997337	psbA-trnH
Moraceae	*Brosimum guianense*	FJ038120	rbcL
Moraceae	*Brosimum guianense*	GQ428589	rbcL
Moraceae	*Brosimum guianense*	GQ981682	rbcL
Moraceae	*Brosimum guianense*	JQ626188	rbcL
Moraceae	*Brosimum guianense*	JX987568	rbcL
Moraceae	*Brosimum guianense*	JX987569	rbcL
Moraceae	*Brosimum guianense*	JX987570	rbcL
Moraceae	*Brosimum guianense*	FJ038719	rpoC1
Moraceae	*Brosimum guianense*	FJ039163	trnL
Moraceae	*Brosimum lactescens*	FJ916996	ITS
Moraceae	*Brosimum lactescens*	JQ588391	matK
Moraceae	*Brosimum lactescens*	JQ588392	matK
Moraceae	*Brosimum lactescens*	JQ588393	matK
Moraceae	*Brosimum lactescens*	JQ592790	rbcL

**Family**	**Species**	**Accession**	**Barcode**
Moraceae	*Brosimum lactescens*	JQ592791	rbcL
Moraceae	*Brosimum lactescens*	JQ592792	rbcL
Moraceae	*Brosimum lactescens*	FJ917061	trnL
Moraceae	*Ficus adhatodifolia*	EU091563	ITS
Moraceae	*Ficus adhatodifolia*	KM186256	ITS
Moraceae	*Ficus adhatodifolia*	KM186257	ITS
Moraceae	*Ficus adhatodifolia*	KM186258	ITS
Moraceae	*Ficus catappifolia*	GQ504301	ITS
Moraceae	*Ficus catappifolia*	GQ504615	rpoC1
Moraceae	*Ficus catappifolia*	GQ504468	trnL
Moraceae	*Ficus cestrifolia*	AY730076	ITS
Moraceae	*Ficus citrifolia*	AY730077	ITS
Moraceae	*Ficus citrifolia*	AY730079	ITS
Moraceae	*Ficus citrifolia*	EU081759	ITS
Moraceae	*Ficus citrifolia*	GQ981992	matK
Moraceae	*Ficus citrifolia*	HM446689	matK
Moraceae	*Ficus citrifolia*	GQ982219	psbA-trnH
Moraceae	*Ficus citrifolia*	HM446931	psbA-trnH
Moraceae	*Ficus citrifolia*	GQ981736	rbcL
Moraceae	*Ficus citrifolia*	HM446799	rbcL
Moraceae	*Ficus citrifolia*	JQ592817	rbcL
Moraceae	*Ficus citrifolia*	JQ592818	rbcL
Moraceae	*Ficus citrifolia*	JQ592819	rbcL
Moraceae	*Ficus citrifolia*	JQ592820	rbcL
Moraceae	*Ficus citrifolia*	JQ592821	rbcL
Moraceae	*Ficus citrifolia*	JQ592822	rbcL
Moraceae	*Ficus citrifolia*	KF724292	rbcL
Moraceae	*Ficus clusiifolia*	EU091600	ITS
Moraceae	*Ficus enormis*	AY730082	ITS
Moraceae	*Ficus gomelleira*	AY730081	ITS
Moraceae	*Ficus gomelleira*	GQ504613	rpoC1
Moraceae	*Ficus gomelleira*	GQ504466	trnL
Moraceae	*Ficus luschnathiana*	AY730083	ITS
Moraceae	*Ficus obtusifolia*	AY730084	ITS
Moraceae	*Ficus obtusifolia*	EU081766	ITS

**Family**	**Species**	**Accession**	**Barcode**
Moraceae	*Ficus obtusifolia*	JX137113	ITS
Moraceae	*Ficus obtusifolia*	GQ981996	matK
Moraceae	*Ficus obtusifolia*	GQ982223	psbA-trnH
Moraceae	*Ficus obtusifolia*	HG963743	psbA-trnH
Moraceae	*Ficus obtusifolia*	GQ981740	rbcL
Moraceae	*Ficus obtusifolia*	JQ592844	rbcL
Moraceae	*Ficus obtusifolia*	JQ592845	rbcL
Moraceae	*Ficus obtusifolia*	JQ592846	rbcL
Moraceae	*Ficus obtusifolia*	JQ592847	rbcL
Moraceae	*Ficus obtusiuscula*	KM186239	ITS
Moraceae	*Ficus pertusa*	AF165400	ITS
Moraceae	*Ficus pertusa*	JQ588411	matK
Moraceae	*Ficus pertusa*	JQ588412	matK
Moraceae	*Ficus pertusa*	JQ588413	matK
Moraceae	*Ficus pertusa*	JQ588414	matK
Moraceae	*Ficus pertusa*	JQ592852	rbcL
Moraceae	*Ficus pertusa*	JQ592853	rbcL
Moraceae	*Ficus pertusa*	JQ592854	rbcL
Moraceae	*Ficus pertusa*	JQ592855	rbcL
Moraceae	*Ficus pertusa*	JQ592856	rbcL
Moraceae	*Ficus pertusa*	JQ592857	rbcL
Moraceae	*Ficus pertusa*	JQ592858	rbcL
Moraceae	*Ficus trigona*	DQ455669	ITS
Moraceae	*Ficus trigona*	GU935026	ITS
Moraceae	*Ficus trigona*	GU935027	ITS
Moraceae	*Ficus trigona*	HM368210	ITS
Moraceae	*Ficus trigona*	HM368211	ITS
Moraceae	*Ficus trigona*	GU935114	psbA-trnH
Moraceae	*Ficus trigona*	GU935115	psbA-trnH
Moraceae	*Ficus trigona*	GU935083	rbcL
Moraceae	*Ficus trigona*	GU935084	rbcL
Moraceae	*Ficus trigona*	GQ504609	rpoC1
Moraceae	*Ficus trigona*	GQ504462	trnL
Moraceae	*Ficus trigonata*	EU081762	ITS
Moraceae	*Ficus trigonata*	EU091607	ITS

Family	Species	Accession	Barcode
Moraceae	*Ficus trigonata*	JX495719	matK
Moraceae	*Ficus trigonata*	KJ012605	matK
Moraceae	*Ficus trigonata*	GQ982226	psbA-trnH
Moraceae	*Ficus trigonata*	KJ426743	psbA-trnH
Moraceae	*Ficus trigonata*	GQ981743	rbcL
Moraceae	*Ficus trigonata*	JX571842	rbcL
Moraceae	*Ficus trigonata*	KJ082318	rbcL
Moraceae	*Ficus trigonata*	KJ082319	rbcL
Moraceae	*Maclura tinctoria*	JQ588419	matK
Moraceae	*Maclura tinctoria*	JQ588420	matK
Moraceae	*Maclura tinctoria*	JQ588421	matK
Moraceae	*Maclura tinctoria*	JQ588422	matK
Moraceae	*Maclura tinctoria*	JQ588423	matK
Moraceae	*Maclura tinctoria*	KJ012662	matK
Moraceae	*Maclura tinctoria*	GQ982277	psbA-trnH
Moraceae	*Maclura tinctoria*	HG963650	psbA-trnH
Moraceae	*Maclura tinctoria*	KJ426804	psbA-trnH
Moraceae	*Maclura tinctoria*	JQ592863	rbcL
Moraceae	*Maclura tinctoria*	JQ592864	rbcL
Moraceae	*Maclura tinctoria*	JQ592865	rbcL
Moraceae	*Maclura tinctoria*	JQ592866	rbcL
Moraceae	*Maclura tinctoria*	JQ592867	rbcL
Moraceae	*Maclura tinctoria*	KJ082396	rbcL
Moraceae	*Morus alba*	AJ554225	ITS
Moraceae	*Morus alba*	AM042003	ITS
Moraceae	*Morus alba*	AY345145	ITS
Moraceae	*Morus alba*	AY345153	ITS
Moraceae	*Morus alba*	HM747164	ITS
Moraceae	*Morus alba*	KC573840-KC573848	ITS
Moraceae	*Morus alba*	KF454437	ITS
Moraceae	*Morus alba*	KF454439	ITS
Moraceae	*Morus alba*	KF454442	ITS
Moraceae	*Morus alba*	KF454443	ITS
Moraceae	*Morus alba*	KF672604	ITS
Moraceae	*Morus alba*	KF784881-KF784897	ITS

Family	Species	Accession	Barcode
Moraceae	*Morus alba*	KF986150	ITS
Moraceae	*Morus alba*	KF986154	ITS
Moraceae	*Morus alba*	KJ606350	ITS
Moraceae	*Morus alba*	KJ606352	ITS
Moraceae	*Morus alba*	KJ606354	ITS
Moraceae	*Morus alba*	KJ606356	ITS
Moraceae	*Morus alba*	KJ606358	ITS
Moraceae	*Morus alba*	KJ606359	ITS
Moraceae	*Morus alba*	GU145562	matK
Moraceae	*Morus alba*	JQ412268	matK
Moraceae	*Morus alba*	JX495736	matK
Moraceae	*Morus alba*	KM030011	matK
Moraceae	*Morus alba*	KP089164	matK
Moraceae	*Morus alba*	KP149516	matK
Moraceae	*Morus alba*	GQ435324	psbA-trnH
Moraceae	*Morus alba*	JN407006-JN407008	psbA-trnH
Moraceae	*Morus alba*	KC584956	psbA-trnH
Moraceae	*Morus alba*	KJ606374	psbA-trnH
Moraceae	*Morus alba*	GU145576	rbcL
Moraceae	*Morus alba*	JQ412393	rbcL
Moraceae	*Morus alba*	JX571867	rbcL
Moraceae	*Morus alba*	KC584883	rbcL
Moraceae	*Morus alba*	KF031063	rbcL
Moraceae	*Morus alba*	KF986157	rbcL
Moraceae	*Morus alba*	KJ606366	rbcL
Moraceae	*Morus alba*	KM030001	rbcL
Moraceae	*Morus alba*	KP149546	rbcL
Moraceae	*Morus alba*	KC584908	rpoC1
Moraceae	*Morus alba*	AF406985	trnL
Moraceae	*Morus alba*	AY271270	trnL
Moraceae	*Morus alba*	AY271280	trnL
Moraceae	*Morus alba*	AY271293	trnL
Moraceae	*Morus alba*	GU145590	trnL
Moraceae	*Morus alba*	HM747180	trnL
Moraceae	*Morus alba*	KM030004	trnL

Family	Species	Accession	Barcode
Moraceae	*Morus alba*	KP088319	ycf1
Moraceae	*Morus nigra*	AB604291	ITS
Moraceae	*Morus nigra*	AJ554226	ITS
Moraceae	*Morus nigra*	AM042002	ITS
Moraceae	*Morus nigra*	HM747174	ITS
Moraceae	*Morus nigra*	KF454438	ITS
Moraceae	*Morus nigra*	KF454440	ITS
Moraceae	*Morus nigra*	KF454441	ITS
Moraceae	*Morus nigra*	KF784875	ITS
Moraceae	*Morus nigra*	KF784876	ITS
Moraceae	*Morus nigra*	GU145558	matK
Moraceae	*Morus nigra*	JX495737	matK
Moraceae	*Morus nigra*	GU145572	rbcL
Moraceae	*Morus nigra*	JX571868	rbcL
Moraceae	*Morus nigra*	AY267539	trnL
Moraceae	*Morus nigra*	EF195632-EF195643	trnL
Moraceae	*Morus nigra*	GU145586	trnL
Moraceae	*Morus nigra*	HM747190	trnL
Moraceae	*Morus nigra*	JN006374	trnL
Moraceae	*Morus nigra*	KT032212	trnL
Myristicaceae	*Virola sebifera*	EU090507	matK
Myristicaceae	*Virola sebifera*	EU090661	psbA-trnH
Myristicaceae	*Virola sebifera*	EU090543	rbcL
Myristicaceae	*Virola sebifera*	GQ981915	rbcL
Myristicaceae	*Virola sebifera*	EU090581	rpoB
Myristicaceae	*Virola sebifera*	EU090621	rpoC1
Myrtaceae	*Acca sellowiana*	AM234067	ITS
Myrtaceae	*Acca sellowiana*	KM064796	ITS
Myrtaceae	*Acca sellowiana*	KM064869	ITS
Myrtaceae	*Acca sellowiana*	KM064915	ITS
Myrtaceae	*Acca sellowiana*	AM489973	matK
Myrtaceae	*Acca sellowiana*	AY525128	matK
Myrtaceae	*Acca sellowiana*	KM065351	matK
Myrtaceae	*Acca sellowiana*	KM065381	matK
Myrtaceae	*Acca sellowiana*	AM489807	psbA-trnH

Family	Species	Accession	Barcode
Myrtaceae	*Acca sellowiana*	JX571773	rbcL
Myrtaceae	*Blepharocalyx salicifolius*	AM234084	ITS
Myrtaceae	*Blepharocalyx salicifolius*	JN660935	ITS
Myrtaceae	*Blepharocalyx salicifolius*	JN660936	ITS
Myrtaceae	*Blepharocalyx salicifolius*	AM489979	matK
Myrtaceae	*Blepharocalyx salicifolius*	AY521531	matK
Myrtaceae	*Blepharocalyx salicifolius*	JN661034	matK
Myrtaceae	*Blepharocalyx salicifolius*	JN661035	matK
Myrtaceae	*Blepharocalyx salicifolius*	AM489815	psbA-trnH
Myrtaceae	*Calyptranthes clusiifolia*	JN091200	ITS
Myrtaceae	*Calyptranthes clusiifolia*	JN091391	psbA-trnH
Myrtaceae	*Calyptranthes concinna*	AM234103	ITS
Myrtaceae	*Calyptranthes concinna*	KP722378	ITS
Myrtaceae	*Calyptranthes concinna*	AM489980	matK
Myrtaceae	*Calyptranthes concinna*	AM489817	psbA-trnH
Myrtaceae	*Calyptranthes concinna*	JN091321	trnL
Myrtaceae	*Calyptranthes grandifolia*	JN091201	ITS
Myrtaceae	*Calyptranthes grandifolia*	JN091302	matK
Myrtaceae	*Calyptranthes grandifolia*	JN091392	psbA-trnH
Myrtaceae	*Calyptranthes grandifolia*	JN091322	trnL
Myrtaceae	*Calyptranthes lanceolata*	AM234104	ITS
Myrtaceae	*Calyptranthes lanceolata*	AM489818	psbA-trnH
Myrtaceae	*Calyptranthes lanceolata*	JN091324	trnL
Myrtaceae	*Campomanesia guazumifolia*	AM234076	ITS
Myrtaceae	*Campomanesia guazumifolia*	AM489982	matK
Myrtaceae	*Campomanesia guazumifolia*	AY521532	matK
Myrtaceae	*Campomanesia guazumifolia*	AM489821	psbA-trnH
Myrtaceae	*Campomanesia pubescens*	AM234077	ITS
Myrtaceae	*Campomanesia pubescens*	AM489822	psbA-trnH
Myrtaceae	*Campomanesia xanthocarpa*	KF421009	ITS
Myrtaceae	*Campomanesia xanthocarpa*	KF421010	ITS
Myrtaceae	*Campomanesia xanthocarpa*	KF421011	ITS
Myrtaceae	*Campomanesia xanthocarpa*	KF555386	matK
Myrtaceae	*Campomanesia xanthocarpa*	KF555387	matK
Myrtaceae	*Campomanesia xanthocarpa*	KF421070	psbA-trnH

**Family**	**Species**	**Accession**	**Barcode**
Myrtaceae	*Campomanesia xanthocarpa*	KF421071	psbA-trnH
Myrtaceae	*Campomanesia xanthocarpa*	KF561904	rbcL
Myrtaceae	*Campomanesia xanthocarpa*	KF561905	rbcL
Myrtaceae	*Campomanesia xanthocarpa*	KF561906	rbcL
Myrtaceae	*Eugenia astringens*	KJ187606	ITS
Myrtaceae	*Eugenia astringens*	KJ469655	psbA-trnH
Myrtaceae	*Eugenia bacopari*	JQ033301	ITS
Myrtaceae	*Eugenia bacopari*	KJ187608	ITS
Myrtaceae	*Eugenia bacopari*	JQ033330	psbA-trnH
Myrtaceae	*Eugenia bacopari*	KJ469657	psbA-trnH
Myrtaceae	*Eugenia bacopari*	JQ033269	rpoB
Myrtaceae	*Eugenia bacopari*	JQ033362	rpoC1
Myrtaceae	*Eugenia beaurepairiana*	JQ033310	ITS
Myrtaceae	*Eugenia beaurepairiana*	KJ187609	ITS
Myrtaceae	*Eugenia beaurepairiana*	JQ033343	psbA-trnH
Myrtaceae	*Eugenia beaurepairiana*	KJ469658	psbA-trnH
Myrtaceae	*Eugenia beaurepairiana*	JQ033281	rpoB
Myrtaceae	*Eugenia beaurepairiana*	JQ033373	rpoC1
Myrtaceae	*Eugenia bocainensis*	KJ187612	ITS
Myrtaceae	*Eugenia bocainensis*	KJ469661	psbA-trnH
Myrtaceae	*Eugenia brasiliensis*	JQ033300	ITS
Myrtaceae	*Eugenia brasiliensis*	KJ187613	ITS
Myrtaceae	*Eugenia brasiliensis*	JQ033332	psbA-trnH
Myrtaceae	*Eugenia brasiliensis*	KJ469662	psbA-trnH
Myrtaceae	*Eugenia brasiliensis*	JQ033271	rpoB
Myrtaceae	*Eugenia brasiliensis*	JQ033364	rpoC1
Myrtaceae	*Eugenia brevistyla*	JQ033307	ITS
Myrtaceae	*Eugenia brevistyla*	KJ187614	ITS
Myrtaceae	*Eugenia brevistyla*	JQ033338	psbA-trnH
Myrtaceae	*Eugenia brevistyla*	KJ469663	psbA-trnH
Myrtaceae	*Eugenia brevistyla*	JQ033282	rpoB
Myrtaceae	*Eugenia brevistyla*	JQ033374	rpoC1
Myrtaceae	*Eugenia burkartiana*	JQ033305	ITS
Myrtaceae	*Eugenia burkartiana*	JQ033334	psbA-trnH
Myrtaceae	*Eugenia burkartiana*	JQ033273	rpoB

**Family**	**Species**	**Accession**	**Barcode**
Myrtaceae	*Eugenia burkartiana*	JQ033366	rpoC1
Myrtaceae	*Eugenia cerasiflora*	AY487289	ITS
Myrtaceae	*Eugenia cerasiflora*	AY487296	ITS
Myrtaceae	*Eugenia cerasiflora*	JF804904	trnL
Myrtaceae	*Eugenia convexinervia*	KJ187618	ITS
Myrtaceae	*Eugenia convexinervia*	KJ469667	psbA-trnH
Myrtaceae	*Eugenia cuprea*	KJ187619	ITS
Myrtaceae	*Eugenia cuprea*	KJ469668	psbA-trnH
Myrtaceae	*Eugenia dysenterica*	KJ187620	ITS
Myrtaceae	*Eugenia dysenterica*	JX850043	matK
Myrtaceae	*Eugenia dysenterica*	KJ469669	psbA-trnH
Myrtaceae	*Eugenia excelsa*	KJ187621	ITS
Myrtaceae	*Eugenia excelsa*	KJ469670	psbA-trnH
Myrtaceae	*Eugenia florida*	AM234090	ITS
Myrtaceae	*Eugenia florida*	JQ033313	ITS
Myrtaceae	*Eugenia florida*	KJ187622	ITS
Myrtaceae	*Eugenia florida*	AM489830	psbA-trnH
Myrtaceae	*Eugenia florida*	JQ033340	psbA-trnH
Myrtaceae	*Eugenia florida*	KJ469671	psbA-trnH
Myrtaceae	*Eugenia florida*	JQ033278	rpoB
Myrtaceae	*Eugenia florida*	JQ033371	rpoC1
Myrtaceae	*Eugenia hiemalis*	JQ033298	ITS
Myrtaceae	*Eugenia hiemalis*	KJ187623	ITS
Myrtaceae	*Eugenia hiemalis*	JQ033328	psbA-trnH
Myrtaceae	*Eugenia hiemalis*	KJ469672	psbA-trnH
Myrtaceae	*Eugenia hiemalis*	JQ033267	rpoB
Myrtaceae	*Eugenia hiemalis*	JQ033360	rpoC1
Myrtaceae	*Eugenia involucrata*	AY487294	ITS
Myrtaceae	*Eugenia involucrata*	JQ033302	ITS
Myrtaceae	*Eugenia involucrata*	JQ033333	psbA-trnH
Myrtaceae	*Eugenia involucrata*	JQ033272	rpoB
Myrtaceae	*Eugenia involucrata*	JQ033365	rpoC1
Myrtaceae	*Eugenia ligustrina*	KJ012589	matK
Myrtaceae	*Eugenia ligustrina*	KJ012590	matK
Myrtaceae	*Eugenia ligustrina*	KJ426728	psbA-trnH

Family	Species	Accession	Barcode
Myrtaceae	*Eugenia ligustrina*	KJ426729	psbA-trnH
Myrtaceae	*Eugenia ligustrina*	KJ082303	rbcL
Myrtaceae	*Eugenia ligustrina*	KJ082304	rbcL
Myrtaceae	*Eugenia melanogyna*	KJ187624	ITS
Myrtaceae	*Eugenia melanogyna*	KJ469673	psbA-trnH
Myrtaceae	*Eugenia modesta*	KJ187625	ITS
Myrtaceae	*Eugenia multicostata*	JQ033312	ITS
Myrtaceae	*Eugenia multicostata*	JQ033336	psbA-trnH
Myrtaceae	*Eugenia multicostata*	JQ033275	rpoB
Myrtaceae	*Eugenia multicostata*	JQ033368	rpoC1
Myrtaceae	*Eugenia myrcianthes*	JQ033291	ITS
Myrtaceae	*Eugenia myrcianthes*	KJ187652	ITS
Myrtaceae	*Eugenia myrcianthes*	AY525131	matK
Myrtaceae	*Eugenia myrcianthes*	JQ033323	psbA-trnH
Myrtaceae	*Eugenia myrcianthes*	KJ469702	psbA-trnH
Myrtaceae	*Eugenia myrcianthes*	JQ033259	rpoB
Myrtaceae	*Eugenia myrcianthes*	JQ033352	rpoC1
Myrtaceae	*Eugenia neoglomerata*	KJ187626	ITS
Myrtaceae	*Eugenia neoglomerata*	KJ469674	psbA-trnH
Myrtaceae	*Eugenia neomyrtifolia*	KJ187627	ITS
Myrtaceae	*Eugenia neomyrtifolia*	KJ469675	psbA-trnH
Myrtaceae	*Eugenia neoverrucosa*	KJ187628	ITS
Myrtaceae	*Eugenia neoverrucosa*	KJ469676	psbA-trnH
Myrtaceae	*Eugenia nutans*	KJ187629	ITS
Myrtaceae	*Eugenia nutans*	KJ469677	psbA-trnH
Myrtaceae	*Eugenia oblongata*	KJ187630	ITS
Myrtaceae	*Eugenia oblongata*	KJ469678	psbA-trnH
Myrtaceae	*Eugenia pisiformis*	KJ187634	ITS
Myrtaceae	*Eugenia pisiformis*	KJ469682	psbA-trnH
Myrtaceae	*Eugenia pluriflora*	KJ187636	ITS
Myrtaceae	*Eugenia pluriflora*	KJ469684	psbA-trnH
Myrtaceae	*Eugenia prasina*	AM234086	ITS
Myrtaceae	*Eugenia prasina*	KJ187637	ITS
Myrtaceae	*Eugenia prasina*	AM489826	psbA-trnH
Myrtaceae	*Eugenia prasina*	KJ469685	psbA-trnH

**Family**	**Species**	**Accession**	**Barcode**
Myrtaceae	*Eugenia punicifolia*	AM234087	ITS
Myrtaceae	*Eugenia punicifolia*	KJ187638	ITS
Myrtaceae	*Eugenia punicifolia*	AM489827	psbA-trnH
Myrtaceae	*Eugenia punicifolia*	KJ469686	psbA-trnH
Myrtaceae	*Eugenia pyriformis*	JQ033304	ITS
Myrtaceae	*Eugenia pyriformis*	KJ187639	ITS
Myrtaceae	*Eugenia pyriformis*	AM489832	psbA-trnH
Myrtaceae	*Eugenia pyriformis*	JQ033331	psbA-trnH
Myrtaceae	*Eugenia pyriformis*	KJ469687	psbA-trnH
Myrtaceae	*Eugenia pyriformis*	JQ033270	rpoB
Myrtaceae	*Eugenia pyriformis*	JQ033363	rpoC1
Myrtaceae	*Eugenia ramboi*	JQ033315	ITS
Myrtaceae	*Eugenia ramboi*	JQ033342	psbA-trnH
Myrtaceae	*Eugenia ramboi*	JQ033280	rpoB
Myrtaceae	*Eugenia ramboi*	JQ033375	rpoC1
Myrtaceae	*Eugenia repanda*	KJ187640	ITS
Myrtaceae	*Eugenia repanda*	KJ469688	psbA-trnH
Myrtaceae	*Eugenia speciosa*	JQ033309	ITS
Myrtaceae	*Eugenia speciosa*	JQ033339	psbA-trnH
Myrtaceae	*Eugenia speciosa*	JQ033277	rpoB
Myrtaceae	*Eugenia speciosa*	JQ033370	rpoC1
Myrtaceae	*Eugenia sphenophylla*	KJ187644	ITS
Myrtaceae	*Eugenia sphenophylla*	KJ469693	psbA-trnH
Myrtaceae	*Eugenia subavenia*	KJ187646	ITS
Myrtaceae	*Eugenia subavenia*	KJ469695	psbA-trnH
Myrtaceae	*Eugenia subterminalis*	KJ469696	psbA-trnH
Myrtaceae	*Eugenia sulcata*	AM234089	ITS
Myrtaceae	*Eugenia sulcata*	KJ187647	ITS
Myrtaceae	*Eugenia sulcata*	AM489987	matK
Myrtaceae	*Eugenia sulcata*	AM489829	psbA-trnH
Myrtaceae	*Eugenia sulcata*	KJ469697	psbA-trnH
Myrtaceae	*Eugenia uniflora*	AM234088	ITS
Myrtaceae	*Eugenia uniflora*	AY487284	ITS
Myrtaceae	*Eugenia uniflora*	JQ033303	ITS
Myrtaceae	*Eugenia uniflora*	KM064791	ITS

Family	Species	Accession	Barcode
Myrtaceae	*Eugenia uniflora*	KM064828	ITS
Myrtaceae	*Eugenia uniflora*	KM064994	ITS
Myrtaceae	*Eugenia uniflora*	AF368207	matK
Myrtaceae	*Eugenia uniflora*	AM489986	matK
Myrtaceae	*Eugenia uniflora*	GU135006	matK
Myrtaceae	*Eugenia uniflora*	KM065110	matK
Myrtaceae	*Eugenia uniflora*	KM065113	matK
Myrtaceae	*Eugenia uniflora*	KM065173	matK
Myrtaceae	*Eugenia uniflora*	KM065203	matK
Myrtaceae	*Eugenia uniflora*	AM489828	psbA-trnH
Myrtaceae	*Eugenia uniflora*	GU135338	psbA-trnH
Myrtaceae	*Eugenia uniflora*	JQ033327	psbA-trnH
Myrtaceae	*Eugenia uniflora*	AF294255	rbcL
Myrtaceae	*Eugenia uniflora*	AM235654	rbcL
Myrtaceae	*Eugenia uniflora*	GU135170	rbcL
Myrtaceae	*Eugenia uniflora*	JQ033266	rpoB
Myrtaceae	*Eugenia uniflora*	JQ033359	rpoC1
Myrtaceae	*Eugenia uniflora*	JF804906	trnL
Myrtaceae	*Eugenia uruguayensis*	JQ033299	ITS
Myrtaceae	*Eugenia uruguayensis*	JQ033329	psbA-trnH
Myrtaceae	*Eugenia uruguayensis*	JQ033268	rpoB
Myrtaceae	*Eugenia uruguayensis*	JQ033361	rpoC1
Myrtaceae	*Eugenia verticillata*	JQ033311	ITS
Myrtaceae	*Eugenia verticillata*	KJ187650	ITS
Myrtaceae	*Eugenia verticillata*	JQ033335	psbA-trnH
Myrtaceae	*Eugenia verticillata*	KJ469700	psbA-trnH
Myrtaceae	*Eugenia verticillata*	JQ033274	rpoB
Myrtaceae	*Eugenia verticillata*	JQ033367	rpoC1
Myrtaceae	*Marlierea angustifolia*	JN091230	ITS
Myrtaceae	*Marlierea angustifolia*	JN091312	matK
Myrtaceae	*Marlierea angustifolia*	JN091421	psbA-trnH
Myrtaceae	*Marlierea angustifolia*	JN091358	trnL
Myrtaceae	*Marlierea clausseniana*	JN091202	ITS
Myrtaceae	*Marlierea clausseniana*	JN091393	psbA-trnH
Myrtaceae	*Marlierea clausseniana*	JN091326	trnL

Family	Species	Accession	Barcode
Myrtaceae	*Marlierea eugeniopsoides*	AM234107	ITS
Myrtaceae	*Marlierea eugeniopsoides*	AM489996	matK
Myrtaceae	*Marlierea eugeniopsoides*	AM489845	psbA-trnH
Myrtaceae	*Marlierea eugeniopsoides*	JN091327	trnL
Myrtaceae	*Marlierea excoriata*	JN091203	ITS
Myrtaceae	*Marlierea excoriata*	JN091394	psbA-trnH
Myrtaceae	*Marlierea excoriata*	JN091328	trnL
Myrtaceae	*Marlierea glazioviana*	JN091204	ITS
Myrtaceae	*Marlierea glazioviana*	JN091395	psbA-trnH
Myrtaceae	*Marlierea glazioviana*	JN091329	trnL
Myrtaceae	*Marlierea obscura*	AM234109	ITS
Myrtaceae	*Marlierea obscura*	JN091205	ITS
Myrtaceae	*Marlierea obscura*	AM489997	matK
Myrtaceae	*Marlierea obscura*	AM489847	psbA-trnH
Myrtaceae	*Marlierea obscura*	JN091396	psbA-trnH
Myrtaceae	*Marlierea obscura*	JN091330	trnL
Myrtaceae	*Marlierea racemosa*	JN091207	ITS
Myrtaceae	*Marlierea racemosa*	JN091303	matK
Myrtaceae	*Marlierea racemosa*	JN091398	psbA-trnH
Myrtaceae	*Marlierea racemosa*	JN091332	trnL
Myrtaceae	*Marlierea regeliana*	JN091208	ITS
Myrtaceae	*Marlierea regeliana*	JN091399	psbA-trnH
Myrtaceae	*Marlierea regeliana*	JN091333	trnL
Myrtaceae	*Marlierea suaveolens*	AM234108	ITS
Myrtaceae	*Marlierea suaveolens*	AM489846	psbA-trnH
Myrtaceae	*Marlierea suaveolens*	JN091334	trnL
Myrtaceae	*Marlierea tomentosa*	JN091210	ITS
Myrtaceae	*Marlierea tomentosa*	JN091401	psbA-trnH
Myrtaceae	*Marlierea tomentosa*	JN091336	trnL
Myrtaceae	*Myrceugenia alpigena*	AM234098	ITS
Myrtaceae	*Myrceugenia alpigena*	JN660891	ITS
Myrtaceae	*Myrceugenia alpigena*	JN660892	ITS
Myrtaceae	*Myrceugenia alpigena*	JN660893	ITS
Myrtaceae	*Myrceugenia alpigena*	JN660990	matK
Myrtaceae	*Myrceugenia alpigena*	JN660991	matK

Family	Species	Accession	Barcode
Myrtaceae	*Myrceugenia alpigena*	JN660992	matK
Myrtaceae	*Myrceugenia alpigena*	AM489854	psbA-trnH
Myrtaceae	*Myrceugenia brevipedicellata*	JN660894	ITS
Myrtaceae	*Myrceugenia brevipedicellata*	JN660993	matK
Myrtaceae	*Myrceugenia campestris*	AY616475	ITS
Myrtaceae	*Myrceugenia campestris*	AY616515	ITS
Myrtaceae	*Myrceugenia campestris*	JN660895	ITS
Myrtaceae	*Myrceugenia campestris*	JN660994	matK
Myrtaceae	*Myrceugenia cucullata*	JN660898	ITS
Myrtaceae	*Myrceugenia cucullata*	JN660997	matK
Myrtaceae	*Myrceugenia euosma*	JN660899	ITS
Myrtaceae	*Myrceugenia euosma*	JN660998	matK
Myrtaceae	*Myrceugenia franciscensis*	JN660902	ITS
Myrtaceae	*Myrceugenia franciscensis*	JN661001	matK
Myrtaceae	*Myrceugenia gertii*	JN660904	ITS
Myrtaceae	*Myrceugenia gertii*	JN661003	matK
Myrtaceae	*Myrceugenia glaucescens*	JN660905	ITS
Myrtaceae	*Myrceugenia glaucescens*	JN661004	matK
Myrtaceae	*Myrceugenia kleinii*	JN660906	ITS
Myrtaceae	*Myrceugenia kleinii*	JN661005	matK
Myrtaceae	*Myrceugenia miersiana*	JN660912	ITS
Myrtaceae	*Myrceugenia miersiana*	JN661011	matK
Myrtaceae	*Myrceugenia myrcioides*	AM234097	ITS
Myrtaceae	*Myrceugenia myrcioides*	JN660913	ITS
Myrtaceae	*Myrceugenia myrcioides*	JN660915	ITS
Myrtaceae	*Myrceugenia myrcioides*	AM490000	matK
Myrtaceae	*Myrceugenia myrcioides*	JN661012	matK
Myrtaceae	*Myrceugenia myrcioides*	JN661014	matK
Myrtaceae	*Myrceugenia myrcioides*	AM489853	psbA-trnH
Myrtaceae	*Myrceugenia ovalifolia*	JN660917	ITS
Myrtaceae	*Myrceugenia ovalifolia*	JN661016	matK
Myrtaceae	*Myrceugenia ovata*	AM234096	ITS
Myrtaceae	*Myrceugenia ovata*	JN660918	ITS
Myrtaceae	*Myrceugenia ovata*	JN660920	ITS
Myrtaceae	*Myrceugenia ovata*	JN660921	ITS

Family	Species	Accession	Barcode
Myrtaceae	*Myrceugenia ovata*	JN660922	ITS
Myrtaceae	*Myrceugenia ovata*	JN661017	matK
Myrtaceae	*Myrceugenia ovata*	JN661019	matK
Myrtaceae	*Myrceugenia ovata*	JN661020	matK
Myrtaceae	*Myrceugenia ovata*	JN661021	matK
Myrtaceae	*Myrceugenia ovata*	JN661037	matK
Myrtaceae	*Myrceugenia ovata*	AM489852	psbA-trnH
Myrtaceae	*Myrceugenia oxysepala*	JN660923	ITS
Myrtaceae	*Myrceugenia oxysepala*	JN661022	matK
Myrtaceae	*Myrceugenia pilotantha*	JN660925	ITS
Myrtaceae	*Myrceugenia pilotantha*	JN660926	ITS
Myrtaceae	*Myrceugenia pilotantha*	JN661024	matK
Myrtaceae	*Myrceugenia pilotantha*	JN661025	matK
Myrtaceae	*Myrceugenia reitzii*	JN660937	ITS
Myrtaceae	*Myrceugenia reitzii*	JN661036	matK
Myrtaceae	*Myrceugenia rufescens*	JN660930	ITS
Myrtaceae	*Myrceugenia rufescens*	JN661029	matK
Myrtaceae	*Myrceugenia seriatoramosa*	JN660932	ITS
Myrtaceae	*Myrceugenia seriatoramosa*	JN661031	matK
Myrtaceae	*Myrcia amazonica*	JN091212	ITS
Myrtaceae	*Myrcia amazonica*	JN091213	ITS
Myrtaceae	*Myrcia amazonica*	JN091214	ITS
Myrtaceae	*Myrcia amazonica*	JN091215	ITS
Myrtaceae	*Myrcia amazonica*	HM446717	matK
Myrtaceae	*Myrcia amazonica*	JN091304	matK
Myrtaceae	*Myrcia amazonica*	JN091305	matK
Myrtaceae	*Myrcia amazonica*	JN091306	matK
Myrtaceae	*Myrcia amazonica*	HM446969	psbA-trnH
Myrtaceae	*Myrcia amazonica*	JN091403	psbA-trnH
Myrtaceae	*Myrcia amazonica*	JN091404	psbA-trnH
Myrtaceae	*Myrcia amazonica*	JN091405	psbA-trnH
Myrtaceae	*Myrcia amazonica*	JN091406	psbA-trnH
Myrtaceae	*Myrcia amazonica*	HM446837	rbcL
Myrtaceae	*Myrcia amazonica*	JN091337	trnL
Myrtaceae	*Myrcia amazonica*	JN091338	trnL

Family	Species	Accession	Barcode
Myrtaceae	*Myrcia amazonica*	JN091339	trnL
Myrtaceae	*Myrcia amazonica*	JN091340	trnL
Myrtaceae	*Myrcia anacardiifolia*	JN091216	ITS
Myrtaceae	*Myrcia anacardiifolia*	JN091407	psbA-trnH
Myrtaceae	*Myrcia anacardiifolia*	JN091341	trnL
Myrtaceae	*Myrcia anceps*	JN091217	ITS
Myrtaceae	*Myrcia anceps*	JN091408	psbA-trnH
Myrtaceae	*Myrcia anceps*	JN091342	trnL
Myrtaceae	*Myrcia brasiliensis*	AM234112	ITS
Myrtaceae	*Myrcia brasiliensis*	JQ033318	ITS
Myrtaceae	*Myrcia brasiliensis*	AM489988	matK
Myrtaceae	*Myrcia brasiliensis*	AM489835	psbA-trnH
Myrtaceae	*Myrcia brasiliensis*	JQ033347	psbA-trnH
Myrtaceae	*Myrcia brasiliensis*	JQ033286	rpoB
Myrtaceae	*Myrcia brasiliensis*	JQ033379	rpoC1
Myrtaceae	*Myrcia brasiliensis*	JN091345	trnL
Myrtaceae	*Myrcia eriopus*	JN091222	ITS
Myrtaceae	*Myrcia eriopus*	JN091413	psbA-trnH
Myrtaceae	*Myrcia eriopus*	JN091348	trnL
Myrtaceae	*Myrcia eumecephylla*	JN091223	ITS
Myrtaceae	*Myrcia eumecephylla*	JN091414	psbA-trnH
Myrtaceae	*Myrcia eumecephylla*	JN091349	trnL
Myrtaceae	*Myrcia fenzliana*	KJ012612	matK
Myrtaceae	*Myrcia fenzliana*	KJ426752	psbA-trnH
Myrtaceae	*Myrcia fenzliana*	KJ082330	rbcL
Myrtaceae	*Myrcia flagellaris*	AM234113	ITS
Myrtaceae	*Myrcia flagellaris*	AM489989	matK
Myrtaceae	*Myrcia flagellaris*	AM489836	psbA-trnH
Myrtaceae	*Myrcia flagellaris*	JN091350	trnL
Myrtaceae	*Myrcia glabra*	JQ033317	ITS
Myrtaceae	*Myrcia glabra*	JQ033346	psbA-trnH
Myrtaceae	*Myrcia glabra*	JQ033285	rpoB
Myrtaceae	*Myrcia glabra*	JQ033378	rpoC1
Myrtaceae	*Myrcia guianensis*	JN091224	ITS
Myrtaceae	*Myrcia guianensis*	JN091225	ITS

**Family**	**Species**	**Accession**	**Barcode**
Myrtaceae	*Myrcia guianensis*	JN091238	ITS
Myrtaceae	*Myrcia guianensis*	KJ012690	matK
Myrtaceae	*Myrcia guianensis*	JN091415	psbA-trnH
Myrtaceae	*Myrcia guianensis*	JN091416	psbA-trnH
Myrtaceae	*Myrcia guianensis*	JN091429	psbA-trnH
Myrtaceae	*Myrcia guianensis*	KJ082447	rbcL
Myrtaceae	*Myrcia guianensis*	KJ082448	rbcL
Myrtaceae	*Myrcia guianensis*	JN091351	trnL
Myrtaceae	*Myrcia guianensis*	JN091368	trnL
Myrtaceae	*Myrcia hartwegiana*	JN091226	ITS
Myrtaceae	*Myrcia hartwegiana*	JN091417	psbA-trnH
Myrtaceae	*Myrcia hartwegiana*	JN091352	trnL
Myrtaceae	*Myrcia hebepetala*	AM234111	ITS
Myrtaceae	*Myrcia hebepetala*	AM489834	psbA-trnH
Myrtaceae	*Myrcia hebepetala*	JN091353	trnL
Myrtaceae	*Myrcia hexasticha*	JN091227	ITS
Myrtaceae	*Myrcia hexasticha*	JN091309	matK
Myrtaceae	*Myrcia hexasticha*	JN091418	psbA-trnH
Myrtaceae	*Myrcia hexasticha*	JN091354	trnL
Myrtaceae	*Myrcia isaiana*	JN091229	ITS
Myrtaceae	*Myrcia isaiana*	JN091311	matK
Myrtaceae	*Myrcia isaiana*	JN091420	psbA-trnH
Myrtaceae	*Myrcia isaiana*	JN091356	trnL
Myrtaceae	*Myrcia laruotteana*	AM234115	ITS
Myrtaceae	*Myrcia laruotteana*	AM490002	matK
Myrtaceae	*Myrcia laruotteana*	AM489856	psbA-trnH
Myrtaceae	*Myrcia laruotteana*	JN091357	trnL
Myrtaceae	*Myrcia laxiflora*	KP722403	ITS
Myrtaceae	*Myrcia laxiflora*	KP722311	psbA-trnH
Myrtaceae	*Myrcia multiflora*	AM234117	ITS
Myrtaceae	*Myrcia multiflora*	KP722379	ITS
Myrtaceae	*Myrcia multiflora*	KP722386	ITS
Myrtaceae	*Myrcia multiflora*	KP722387	ITS
Myrtaceae	*Myrcia multiflora*	AM490003	matK
Myrtaceae	*Myrcia multiflora*	AM489858	psbA-trnH

Family	Species	Accession	Barcode
Myrtaceae	*Myrcia multiflora*	KP722286	psbA-trnH
Myrtaceae	*Myrcia multiflora*	KP722293	psbA-trnH
Myrtaceae	*Myrcia multiflora*	KP722294	psbA-trnH
Myrtaceae	*Myrcia multiflora*	JN091360	trnL
Myrtaceae	*Myrcia palustris*	JQ033319	ITS
Myrtaceae	*Myrcia palustris*	JQ033348	psbA-trnH
Myrtaceae	*Myrcia palustris*	JQ033287	rpoB
Myrtaceae	*Myrcia palustris*	JQ033380	rpoC1
Myrtaceae	*Myrcia pubescens*	JN091234	ITS
Myrtaceae	*Myrcia pubescens*	JN091425	psbA-trnH
Myrtaceae	*Myrcia pubescens*	JN091363	trnL
Myrtaceae	*Myrcia pubipetala*	AM234114	ITS
Myrtaceae	*Myrcia pubipetala*	AM490001	matK
Myrtaceae	*Myrcia pubipetala*	AM489855	psbA-trnH
Myrtaceae	*Myrcia pubipetala*	JN091364	trnL
Myrtaceae	*Myrcia pulchra*	AM234121	ITS
Myrtaceae	*Myrcia pulchra*	JN091235	ITS
Myrtaceae	*Myrcia pulchra*	JN091314	matK
Myrtaceae	*Myrcia pulchra*	AM489862	psbA-trnH
Myrtaceae	*Myrcia pulchra*	JN091426	psbA-trnH
Myrtaceae	*Myrcia pulchra*	JN091343	trnL
Myrtaceae	*Myrcia pulchra*	JN091365	trnL
Myrtaceae	*Myrcia racemosa*	KP722380	ITS
Myrtaceae	*Myrcia racemosa*	AM490005	matK
Myrtaceae	*Myrcia racemosa*	AM489861	psbA-trnH
Myrtaceae	*Myrcia racemosa*	KP722287	psbA-trnH
Myrtaceae	*Myrcia racemosa*	JN091366	trnL
Myrtaceae	*Myrcia retorta*	JN091237	ITS
Myrtaceae	*Myrcia retorta*	JN091428	psbA-trnH
Myrtaceae	*Myrcia rufipes*	JN091239	ITS
Myrtaceae	*Myrcia rufipes*	JN091430	psbA-trnH
Myrtaceae	*Myrcia rufipes*	JN091369	trnL
Myrtaceae	*Myrcia selloi*	JN091240	ITS
Myrtaceae	*Myrcia selloi*	JN091315	matK
Myrtaceae	*Myrcia selloi*	JN091431	psbA-trnH

Family	Species	Accession	Barcode
Myrtaceae	*Myrcia selloi*	JN091371	trnL
Myrtaceae	*Myrcia spectabilis*	JN091241	ITS
Myrtaceae	*Myrcia spectabilis*	JN091432	psbA-trnH
Myrtaceae	*Myrcia spectabilis*	JN091372	trnL
Myrtaceae	*Myrcia splendens*	GQ248009	atpF-atpH
Myrtaceae	*Myrcia splendens*	AM234122	ITS
Myrtaceae	*Myrcia splendens*	AM234124	ITS
Myrtaceae	*Myrcia splendens*	JN091242	ITS
Myrtaceae	*Myrcia splendens*	JN091243	ITS
Myrtaceae	*Myrcia splendens*	KF420977	ITS
Myrtaceae	*Myrcia splendens*	KF420978	ITS
Myrtaceae	*Myrcia splendens*	KF420979	ITS
Myrtaceae	*Myrcia splendens*	AM490006	matK
Myrtaceae	*Myrcia splendens*	AM889732	matK
Myrtaceae	*Myrcia splendens*	GQ248164	matK
Myrtaceae	*Myrcia splendens*	HM446716	matK
Myrtaceae	*Myrcia splendens*	HM446718	matK
Myrtaceae	*Myrcia splendens*	JQ588489	matK
Myrtaceae	*Myrcia splendens*	JQ588490	matK
Myrtaceae	*Myrcia splendens*	JQ588491	matK
Myrtaceae	*Myrcia splendens*	JQ588492	matK
Myrtaceae	*Myrcia splendens*	JQ588493	matK
Myrtaceae	*Myrcia splendens*	JQ588494	matK
Myrtaceae	*Myrcia splendens*	JQ588495	matK
Myrtaceae	*Myrcia splendens*	JQ588496	matK
Myrtaceae	*Myrcia splendens*	JQ588497	matK
Myrtaceae	*Myrcia splendens*	JQ588498	matK
Myrtaceae	*Myrcia splendens*	JQ588499	matK
Myrtaceae	*Myrcia splendens*	KF555416	matK
Myrtaceae	*Myrcia splendens*	AM489863	psbA-trnH
Myrtaceae	*Myrcia splendens*	AM489865	psbA-trnH
Myrtaceae	*Myrcia splendens*	GQ248349	psbA-trnH
Myrtaceae	*Myrcia splendens*	HM446968	psbA-trnH
Myrtaceae	*Myrcia splendens*	HM446970	psbA-trnH
Myrtaceae	*Myrcia splendens*	JN091433	psbA-trnH

**Family**	**Species**	**Accession**	**Barcode**
Myrtaceae	*Myrcia splendens*	JN091434	psbA-trnH
Myrtaceae	*Myrcia splendens*	KF421072	psbA-trnH
Myrtaceae	*Myrcia splendens*	KF421073	psbA-trnH
Myrtaceae	*Myrcia splendens*	KF421074	psbA-trnH
Myrtaceae	*Myrcia splendens*	GQ248498	psbK-psbI
Myrtaceae	*Myrcia splendens*	GQ248652	rbcL
Myrtaceae	*Myrcia splendens*	HM446836	rbcL
Myrtaceae	*Myrcia splendens*	HM446838	rbcL
Myrtaceae	*Myrcia splendens*	JQ592963	rbcL
Myrtaceae	*Myrcia splendens*	JQ592964	rbcL
Myrtaceae	*Myrcia splendens*	JQ592965	rbcL
Myrtaceae	*Myrcia splendens*	JQ592966	rbcL
Myrtaceae	*Myrcia splendens*	JQ592967	rbcL
Myrtaceae	*Myrcia splendens*	JQ592968	rbcL
Myrtaceae	*Myrcia splendens*	JQ592969	rbcL
Myrtaceae	*Myrcia splendens*	JQ625851	rbcL
Myrtaceae	*Myrcia splendens*	KF561933	rbcL
Myrtaceae	*Myrcia splendens*	KF561934	rbcL
Myrtaceae	*Myrcia splendens*	KF561935	rbcL
Myrtaceae	*Myrcia splendens*	AM889825	rpoB
Myrtaceae	*Myrcia splendens*	GQ248823	rpoB
Myrtaceae	*Myrcia splendens*	AM889916	rpoC1
Myrtaceae	*Myrcia splendens*	GQ248984	rpoC1
Myrtaceae	*Myrcia splendens*	JN091373	trnL
Myrtaceae	*Myrcia splendens*	JN091374	trnL
Myrtaceae	*Myrcia splendens*	JN091375	trnL
Myrtaceae	*Myrcia splendens*	JN091376	trnL
Myrtaceae	*Myrcia tenuivenosa*	JN091246	ITS
Myrtaceae	*Myrcia tenuivenosa*	JN091317	matK
Myrtaceae	*Myrcia tenuivenosa*	JN091437	psbA-trnH
Myrtaceae	*Myrcia tenuivenosa*	JN091378	trnL
Myrtaceae	*Myrcia tijucensis*	AM234110	ITS
Myrtaceae	*Myrcia tijucensis*	AM489833	psbA-trnH
Myrtaceae	*Myrcia tijucensis*	JN091379	trnL
Myrtaceae	*Myrcia tomentosa*	AM234116	ITS

**Family**	**Species**	**Accession**	**Barcode**
Myrtaceae	*Myrcia tomentosa*	AM489857	psbA-trnH
Myrtaceae	*Myrcia tomentosa*	JN091380	trnL
Myrtaceae	*Myrcia variabilis*	JN091248	ITS
Myrtaceae	*Myrcia variabilis*	JN091319	matK
Myrtaceae	*Myrcia variabilis*	JN091439	psbA-trnH
Myrtaceae	*Myrcia variabilis*	JN091382	trnL
Myrtaceae	*Myrcia venulosa*	AM234125	ITS
Myrtaceae	*Myrcia venulosa*	AM489866	psbA-trnH
Myrtaceae	*Myrcia venulosa*	JN091383	trnL
Myrtaceae	*Myrcia vestita*	JN091249	ITS
Myrtaceae	*Myrcia vestita*	JN091440	psbA-trnH
Myrtaceae	*Myrcia vestita*	JN091384	trnL
Myrtaceae	*Myrcia vittoriana*	JN091250	ITS
Myrtaceae	*Myrcia vittoriana*	JN091441	psbA-trnH
Myrtaceae	*Myrcia vittoriana*	JN091385	trnL
Myrtaceae	*Myrcianthes gigantea*	JQ033321	ITS
Myrtaceae	*Myrcianthes gigantea*	JQ033350	psbA-trnH
Myrtaceae	*Myrcianthes gigantea*	JQ033289	rpoB
Myrtaceae	*Myrcianthes gigantea*	JQ033382	rpoC1
Myrtaceae	*Myrcianthes pungens*	AM234099	ITS
Myrtaceae	*Myrcianthes pungens*	KJ187656	ITS
Myrtaceae	*Myrcianthes pungens*	AM489867	psbA-trnH
Myrtaceae	*Myrcianthes pungens*	KJ469706	psbA-trnH
Myrtaceae	*Myrciaria cuspidata*	JQ033316	ITS
Myrtaceae	*Myrciaria cuspidata*	JQ033345	psbA-trnH
Myrtaceae	*Myrciaria cuspidata*	JQ033284	rpoB
Myrtaceae	*Myrciaria cuspidata*	JQ033377	rpoC1
Myrtaceae	*Myrciaria floribunda*	AM234094	ITS
Myrtaceae	*Myrciaria floribunda*	KJ426839	psbA-trnH
Myrtaceae	*Myrciaria floribunda*	JQ626319	rbcL
Myrtaceae	*Pimenta pseudocaryophyllus*	AM234083	ITS
Myrtaceae	*Pimenta pseudocaryophyllus*	AM490013	matK
Myrtaceae	*Pimenta pseudocaryophyllus*	AM489876	psbA-trnH
Myrtaceae	*Plinia cauliflora*	AM234093	ITS
Myrtaceae	*Plinia cauliflora*	KM064870	ITS

Family	Species	Accession	Barcode
Myrtaceae	*Plinia cauliflora*	KM064982	ITS
Myrtaceae	*Plinia cauliflora*	AM490007	matK
Myrtaceae	*Plinia cauliflora*	KM065264	matK
Myrtaceae	*Plinia cauliflora*	KM065319	matK
Myrtaceae	*Plinia cauliflora*	AM489869	psbA-trnH
Myrtaceae	*Plinia cauliflora*	JN091387	trnL
Myrtaceae	*Plinia pseudodichasiantha*	AM489411	ITS
Myrtaceae	*Plinia pseudodichasiantha*	JN091388	trnL
Myrtaceae	*Plinia rivularis*	JQ626311	rbcL
Myrtaceae	*Psidium cattleianum*	AB354960	ITS
Myrtaceae	*Psidium cattleianum*	AM234080	ITS
Myrtaceae	*Psidium cattleianum*	KM064916	ITS
Myrtaceae	*Psidium cattleianum*	KM064972	ITS
Myrtaceae	*Psidium cattleianum*	KM065002	ITS
Myrtaceae	*Psidium cattleianum*	AB354959	matK
Myrtaceae	*Psidium cattleianum*	AM490014	matK
Myrtaceae	*Psidium cattleianum*	GU135031	matK
Myrtaceae	*Psidium cattleianum*	HM851054	matK
Myrtaceae	*Psidium cattleianum*	KM065073	matK
Myrtaceae	*Psidium cattleianum*	KM065132	matK
Myrtaceae	*Psidium cattleianum*	AM489878	psbA-trnH
Myrtaceae	*Psidium cattleianum*	GU135360	psbA-trnH
Myrtaceae	*Psidium cattleianum*	GU135194	rbcL
Myrtaceae	*Psidium cattleianum*	HM850290	rbcL
Myrtaceae	*Psidium cattleianum*	AB817363	trnL
Myrtaceae	*Psidium cattleianum*	AB817414	trnL
Myrtaceae	*Psidium cattleianum*	KC428589	trnL
Myrtaceae	*Psidium grandifolium*	AM234079	ITS
Myrtaceae	*Psidium grandifolium*	AM489877	psbA-trnH
Myrtaceae	*Psidium guajava*	AB354956	ITS
Myrtaceae	*Psidium guajava*	AY487283-AY781099	ITS
Myrtaceae	*Psidium guajava*	AY864898	ITS
Myrtaceae	*Psidium guajava*	AB354958	matK
Myrtaceae	*Psidium guajava*	GU135087	matK
Myrtaceae	*Psidium guajava*	JQ024986	matK

Family	Species	Accession	Barcode
Myrtaceae	*Psidium guajava*	JQ024987	matK
Myrtaceae	*Psidium guajava*	JQ588508-JQ588512	matK
Myrtaceae	*Psidium guajava*	GQ434986	psbA-trnH
Myrtaceae	*Psidium guajava*	GU135421	psbA-trnH
Myrtaceae	*Psidium guajava*	HG963647	psbA-trnH
Myrtaceae	*Psidium guajava*	GU135250	rbcL
Myrtaceae	*Psidium guajava*	JQ025077	rbcL
Myrtaceae	*Psidium guajava*	JQ025078	rbcL
Myrtaceae	*Psidium guajava*	JQ592981	rbcL
Myrtaceae	*Psidium guajava*	JQ592982	rbcL
Myrtaceae	*Psidium guajava*	JQ592983	rbcL
Myrtaceae	*Psidium guajava*	JQ592984	rbcL
Myrtaceae	*Psidium guajava*	KP231663	rpoB
Myrtaceae	*Psidium guajava*	KP231664	rpoB
Myrtaceae	*Psidium guajava*	AB817344	trnL
Myrtaceae	*Psidium guajava*	AB817418	trnL
Myrtaceae	*Psidium guajava*	JF804924	trnL
Myrtaceae	*Psidium guajava*	KC428590	trnL
Myrtaceae	*Psidium guineense*	JQ588513	matK
Myrtaceae	*Psidium guineense*	JQ588514	matK
Myrtaceae	*Psidium guineense*	JQ588515	matK
Myrtaceae	*Psidium guineense*	HG963888	psbA-trnH
Myrtaceae	*Psidium guineense*	JQ592985	rbcL
Myrtaceae	*Siphoneugena densiflora*	AM489412	ITS
Myrtaceae	*Siphoneugena densiflora*	AM489571	ITS
Myrtaceae	*Siphoneugena densiflora*	JN091389	trnL
Myrtaceae	*Siphoneugena guilfoyleiana*	AM234085	ITS
Myrtaceae	*Siphoneugena guilfoyleiana*	AM490016	matK
Myrtaceae	*Siphoneugena guilfoyleiana*	AM490638	psbA-trnH
Myrtaceae	*Siphoneugena guilfoyleiana*	JN091390	trnL
Myrtaceae	*Syzygium cumini*	FM887016	ITS
Myrtaceae	*Syzygium cumini*	JF682812	ITS
Myrtaceae	*Syzygium cumini*	JN115051	ITS
Myrtaceae	*Syzygium cumini*	JX856510	ITS
Myrtaceae	*Syzygium cumini*	JX856605	ITS

Family	Species	Accession	Barcode
Myrtaceae	*Syzygium cumini*	KF186456	ITS
Myrtaceae	*Syzygium cumini*	KR532622	ITS
Myrtaceae	*Syzygium cumini*	KR532623	ITS
Myrtaceae	*Syzygium cumini*	AB924857	matK
Myrtaceae	*Syzygium cumini*	AB924961	matK
Myrtaceae	*Syzygium cumini*	AB925007	matK
Myrtaceae	*Syzygium cumini*	AB925017	matK
Myrtaceae	*Syzygium cumini*	AB925038	matK
Myrtaceae	*Syzygium cumini*	AB925174	matK
Myrtaceae	*Syzygium cumini*	AB925241	matK
Myrtaceae	*Syzygium cumini*	AY525140	matK
Myrtaceae	*Syzygium cumini*	DQ088575	matK
Myrtaceae	*Syzygium cumini*	GU134997	matK
Myrtaceae	*Syzygium cumini*	GU135062	matK
Myrtaceae	*Syzygium cumini*	JN114772	matK
Myrtaceae	*Syzygium cumini*	JX495762	matK
Myrtaceae	*Syzygium cumini*	KR531524	matK
Myrtaceae	*Syzygium cumini*	KR531525	matK
Myrtaceae	*Syzygium cumini*	KR531526	matK
Myrtaceae	*Syzygium cumini*	KR531527	matK
Myrtaceae	*Syzygium cumini*	KR531528	matK
Myrtaceae	*Syzygium cumini*	GU135329	psbA-trnH
Myrtaceae	*Syzygium cumini*	GU135395	psbA-trnH
Myrtaceae	*Syzygium cumini*	JX856956	psbA-trnH
Myrtaceae	*Syzygium cumini*	JX856957	psbA-trnH
Myrtaceae	*Syzygium cumini*	KR532983	psbA-trnH
Myrtaceae	*Syzygium cumini*	KR532990	psbA-trnH
Myrtaceae	*Syzygium cumini*	KR532998	psbA-trnH
Myrtaceae	*Syzygium cumini*	AB925471	rbcL
Myrtaceae	*Syzygium cumini*	AB925586	rbcL
Myrtaceae	*Syzygium cumini*	AB925635	rbcL
Myrtaceae	*Syzygium cumini*	AB925645	rbcL
Myrtaceae	*Syzygium cumini*	AB925666	rbcL
Myrtaceae	*Syzygium cumini*	AB925805	rbcL
Myrtaceae	*Syzygium cumini*	AB925872	rbcL

Family	Species	Accession	Barcode
Myrtaceae	*Syzygium cumini*	GU135161	rbcL
Myrtaceae	*Syzygium cumini*	GU135224	rbcL
Myrtaceae	*Syzygium cumini*	JN114842	rbcL
Myrtaceae	*Syzygium cumini*	JX571897	rbcL
Myrtaceae	*Syzygium cumini*	JX856782	rbcL
Myrtaceae	*Syzygium cumini*	KF381145	rbcL
Myrtaceae	*Syzygium cumini*	KR530085	rbcL
Myrtaceae	*Syzygium cumini*	KR530086	rbcL
Myrtaceae	*Syzygium cumini*	KR530087	rbcL
Myrtaceae	*Syzygium cumini*	KR530088	rbcL
Myrtaceae	*Syzygium cumini*	KR530089	rbcL
Myrtaceae	*Syzygium cumini*	KT026222	rbcL
Myrtaceae	*Syzygium cumini*	JN114920	rpoB
Myrtaceae	*Syzygium cumini*	JN114999	rpoC1
Myrtaceae	*Syzygium cumini*	JF804935	trnL
Myrtaceae	*Syzygium jambos*	AM234135	ITS
Myrtaceae	*Syzygium jambos*	EF026629	ITS
Myrtaceae	*Syzygium jambos*	JF682816	ITS
Myrtaceae	*Syzygium jambos*	JX856511	ITS
Myrtaceae	*Syzygium jambos*	KC815991	ITS
Myrtaceae	*Syzygium jambos*	KP093050	ITS
Myrtaceae	*Syzygium jambos*	KP093051	ITS
Myrtaceae	*Syzygium jambos*	AM490017	matK
Myrtaceae	*Syzygium jambos*	DQ088583	matK
Myrtaceae	*Syzygium jambos*	GU135056	matK
Myrtaceae	*Syzygium jambos*	HM446748	matK
Myrtaceae	*Syzygium jambos*	HQ415317	matK
Myrtaceae	*Syzygium jambos*	JQ588516	matK
Myrtaceae	*Syzygium jambos*	KP093262	matK
Myrtaceae	*Syzygium jambos*	KP093263	matK
Myrtaceae	*Syzygium jambos*	AM489882	psbA-trnH
Myrtaceae	*Syzygium jambos*	GU135387	psbA-trnH
Myrtaceae	*Syzygium jambos*	HM447006	psbA-trnH
Myrtaceae	*Syzygium jambos*	HQ415491	psbA-trnH
Myrtaceae	*Syzygium jambos*	KP095755	psbA-trnH

**Family**	**Species**	**Accession**	**Barcode**
Myrtaceae	*Syzygium jambos*	KP095756	psbA-trnH
Myrtaceae	*Syzygium jambos*	GU135219	rbcL
Myrtaceae	*Syzygium jambos*	HM446876	rbcL
Myrtaceae	*Syzygium jambos*	HQ415141	rbcL
Myrtaceae	*Syzygium jambos*	JQ592986	rbcL
Myrtaceae	*Syzygium jambos*	JX856783	rbcL
Myrtaceae	*Syzygium jambos*	KP094169	rbcL
Myrtaceae	*Syzygium jambos*	KP094170	rbcL
Nyctaginaceae	*Bougainvillea glabra*	EF079463	ITS
Nyctaginaceae	*Bougainvillea glabra*	AY042560	matK
Nyctaginaceae	*Bougainvillea glabra*	JQ412215	matK
Nyctaginaceae	*Bougainvillea glabra*	JQ844141	matK
Nyctaginaceae	*Bougainvillea glabra*	JX495674	matK
Nyctaginaceae	*Bougainvillea glabra*	KP299261	matK
Nyctaginaceae	*Bougainvillea glabra*	JQ412331	rbcL
Nyctaginaceae	*Bougainvillea glabra*	JX571786	rbcL
Nyctaginaceae	*Bougainvillea glabra*	M88340	rbcL
Nyctaginaceae	*Bougainvillea spectabilis*	KJ161169	ITS
Nyctaginaceae	*Bougainvillea spectabilis*	KJ161170	ITS
Nyctaginaceae	*Bougainvillea spectabilis*	L78087	ITS
Nyctaginaceae	*Bougainvillea spectabilis*	JN114741	matK
Nyctaginaceae	*Bougainvillea spectabilis*	KP088978	matK
Nyctaginaceae	*Bougainvillea spectabilis*	JN114788	rbcL
Nyctaginaceae	*Bougainvillea spectabilis*	KF381106	rbcL
Nyctaginaceae	*Bougainvillea spectabilis*	JN114861	rpoB
Nyctaginaceae	*Bougainvillea spectabilis*	JN114944	rpoC1
Nyctaginaceae	*Bougainvillea spectabilis*	AJ558036	trnL
Nyctaginaceae	*Bougainvillea spectabilis*	KM262024	trnL
Nyctaginaceae	*Bougainvillea spectabilis*	KP088431	ycf1
Nyctaginaceae	*Guapira obtusata*	KJ082335	rbcL
Nyctaginaceae	*Neea hermaphrodita*	EF079489	ITS
Nyctaginaceae	*Pisonia aculeata*	DQ317077	ITS
Nyctaginaceae	*Pisonia aculeata*	JX844235	ITS
Nyctaginaceae	*Pisonia aculeata*	JQ588520	matK
Nyctaginaceae	*Pisonia aculeata*	JQ588521	matK

**Family**	**Species**	**Accession**	**Barcode**
Nyctaginaceae	*Pisonia aculeata*	JQ588522	matK
Nyctaginaceae	*Pisonia aculeata*	JQ588523	matK
Nyctaginaceae	*Pisonia aculeata*	JQ589905	matK
Nyctaginaceae	*Pisonia aculeata*	JX844247	matK
Nyctaginaceae	*Pisonia aculeata*	KJ593992	matK
Nyctaginaceae	*Pisonia aculeata*	KJ593993	matK
Nyctaginaceae	*Pisonia aculeata*	HG963546	psbA-trnH
Nyctaginaceae	*Pisonia aculeata*	JQ592990	rbcL
Nyctaginaceae	*Pisonia aculeata*	JQ592991	rbcL
Nyctaginaceae	*Pisonia aculeata*	JQ592992	rbcL
Nyctaginaceae	*Pisonia aculeata*	JQ592993	rbcL
Nyctaginaceae	*Pisonia aculeata*	JQ592994	rbcL
Nyctaginaceae	*Pisonia aculeata*	JQ592995	rbcL
Nyctaginaceae	*Pisonia aculeata*	JQ592996	rbcL
Nyctaginaceae	*Pisonia aculeata*	JQ594885	rbcL
Nyctaginaceae	*Pisonia aculeata*	JX844262	rbcL
Nyctaginaceae	*Pisonia aculeata*	KJ594427	rbcL
Nyctaginaceae	*Pisonia aculeata*	KJ594428	rbcL
Nyctaginaceae	*Pisonia aculeata*	JX844280	trnL
Ochnaceae	*Ouratea castaneifolia*	KP196873	rbcL
Ochnaceae	*Ouratea castaneifolia*	KP196911	trnL
Ochnaceae	*Ouratea floribunda*	KP196874	rbcL
Ochnaceae	*Ouratea floribunda*	KP196912	trnL
Ochnaceae	*Ouratea vaccinioides*	KF263220	ITS
Ochnaceae	*Ouratea vaccinioides*	KF263285	matK
Ochnaceae	*Ouratea vaccinioides*	KF263477	trnL
Ochnaceae	*Quiina glazovii*	JX664069	rbcL
Ochnaceae	*Quiina glazovii*	JX663497	rpoB
Ochnaceae	*Quiina glazovii*	JX664960	rpoC1
Ochnaceae	*Quiina glazovii*	JX664704	ycf1
Olacaceae	*Ximenia americana*	DQ333869	ITS
Olacaceae	*Ximenia americana*	DQ790186	matK
Olacaceae	*Ximenia americana*	GQ997871	matK
Olacaceae	*Ximenia americana*	JF270999	matK
Olacaceae	*Ximenia americana*	JQ588540	matK

Family	Species	Accession	Barcode
Olacaceae	*Ximenia americana*	JQ588541	matK
Olacaceae	*Ximenia americana*	JQ588542	matK
Olacaceae	*Ximenia americana*	JX517654	matK
Olacaceae	*Ximenia americana*	KJ012821	matK
Olacaceae	*Ximenia americana*	DQ790149	rbcL
Olacaceae	*Ximenia americana*	GQ997898	rbcL
Olacaceae	*Ximenia americana*	JF265658	rbcL
Olacaceae	*Ximenia americana*	JQ593025	rbcL
Olacaceae	*Ximenia americana*	JQ593026	rbcL
Olacaceae	*Ximenia americana*	JQ593027	rbcL
Olacaceae	*Ximenia americana*	JX573094	rbcL
Olacaceae	*Ximenia americana*	KF496540	rbcL
Olacaceae	*Ximenia americana*	KJ082636	rbcL
Olacaceae	*Ximenia americana*	GQ997909	rpoB
Olacaceae	*Ximenia americana*	GQ997910	rpoC1
Olacaceae	*Ximenia americana*	DQ340620	trnL
Olacaceae	*Ximenia americana*	GQ997928	ycf1
Oleaceae	*Chionanthus filiformis*	LN515400	ITS
Oleaceae	*Chionanthus filiformis*	AF231809	trnL
Oleaceae	*Chionanthus filiformis*	LN515460	trnL
Oleaceae	*Chionanthus filiformis*	LN515518	trnL
Oleaceae	*Ligustrum vulgare*	FJ395354	atpF-atpH
Oleaceae	*Ligustrum vulgare*	AF361298	ITS
Oleaceae	*Ligustrum vulgare*	AM933476	ITS
Oleaceae	*Ligustrum vulgare*	EU314901	ITS
Oleaceae	*Ligustrum vulgare*	KR134837	ITS
Oleaceae	*Ligustrum vulgare*	KT695286	ITS
Oleaceae	*Ligustrum vulgare*	FJ395441	matK
Oleaceae	*Ligustrum vulgare*	FN668807	matK
Oleaceae	*Ligustrum vulgare*	HE966946	matK
Oleaceae	*Ligustrum vulgare*	HQ384543	matK
Oleaceae	*Ligustrum vulgare*	HQ619820	matK
Oleaceae	*Ligustrum vulgare*	JN893855	matK
Oleaceae	*Ligustrum vulgare*	JN894788	matK
Oleaceae	*Ligustrum vulgare*	JN895660	matK

Family	Species	Accession	Barcode
Oleaceae	*Ligustrum vulgare*	KJ204499	matK
Oleaceae	*Ligustrum vulgare*	KP643122	matK
Oleaceae	*Ligustrum vulgare*	FJ395550	psbA-trnH
Oleaceae	*Ligustrum vulgare*	FJ493288	psbA-trnH
Oleaceae	*Ligustrum vulgare*	FN675794	psbA-trnH
Oleaceae	*Ligustrum vulgare*	GU120319	psbA-trnH
Oleaceae	*Ligustrum vulgare*	HE966688	psbA-trnH
Oleaceae	*Ligustrum vulgare*	KR134859	psbA-trnH
Oleaceae	*Ligustrum vulgare*	DQ673302	rbcL
Oleaceae	*Ligustrum vulgare*	FJ395605	rbcL
Oleaceae	*Ligustrum vulgare*	FJ862059	rbcL
Oleaceae	*Ligustrum vulgare*	HE963539	rbcL
Oleaceae	*Ligustrum vulgare*	HQ384901	rbcL
Oleaceae	*Ligustrum vulgare*	HQ619759	rbcL
Oleaceae	*Ligustrum vulgare*	JN890556	rbcL
Oleaceae	*Ligustrum vulgare*	JN890610	rbcL
Oleaceae	*Ligustrum vulgare*	JN891799	rbcL
Oleaceae	*Ligustrum vulgare*	JN893020	rbcL
Oleaceae	*Ligustrum vulgare*	KJ204365	rbcL
Oleaceae	*Ligustrum vulgare*	KP644045	rbcL
Oleaceae	*Ligustrum vulgare*	KT695578	rbcL
Oleaceae	*Ligustrum vulgare*	L11686	rbcL
Oleaceae	*Ligustrum vulgare*	FJ395724	rpoB
Oleaceae	*Ligustrum vulgare*	FN668856	rpoB
Oleaceae	*Ligustrum vulgare*	FJ395854	rpoC1
Oleaceae	*Ligustrum vulgare*	FN689634	rpoC1
Oleaceae	*Ligustrum vulgare*	AF231848	trnL
Oleaceae	*Ligustrum vulgare*	AF380876	trnL
Oleaceae	*Ligustrum vulgare*	AM931524	trnL
Oleaceae	*Ligustrum vulgare*	AM933081	trnL
Oleaceae	*Ligustrum vulgare*	FJ490797	trnL
Oleaceae	*Ligustrum vulgare*	HQ323957	trnL
Onagraceae	*Fuchsia regia*	AY357801	ITS
Onagraceae	*Fuchsia regia*	AY357842	trnL
Onagraceae	*Ludwigia nervosa*	KP026979	ITS

Family	Species	Accession	Barcode
Onagraceae	*Ludwigia nervosa*	KP026980	ITS
Onagraceae	*Ludwigia nervosa*	JQ593031	rbcL
Onagraceae	*Ludwigia nervosa*	JQ593032	rbcL
Onagraceae	*Ludwigia nervosa*	JQ593033	rbcL
Peraceae	*Pera glabrata*	DQ787417	ITS
Peraceae	*Pera heteranthera*	AB233765	matK
Peraceae	*Pera heteranthera*	AB233869	rbcL
Phyllanthaceae	*Gonatogyne brasiliensis*	AY552429	matK
Phyllanthaceae	*Gonatogyne brasiliensis*	AJ418815	rbcL
Phyllanthaceae	*Hyeronima alchorneoides*	FJ037822	ITS
Phyllanthaceae	*Hyeronima alchorneoides*	FJ037824	ITS
Phyllanthaceae	*Hyeronima alchorneoides*	FJ514743	matK
Phyllanthaceae	*Hyeronima alchorneoides*	FJ514744	matK
Phyllanthaceae	*Hyeronima alchorneoides*	GQ982012	matK
Phyllanthaceae	*Hyeronima alchorneoides*	JQ626494	matK
Phyllanthaceae	*Hyeronima alchorneoides*	FJ039032	psbA-trnH
Phyllanthaceae	*Hyeronima alchorneoides*	GQ428769	psbA-trnH
Phyllanthaceae	*Hyeronima alchorneoides*	GQ982246	psbA-trnH
Phyllanthaceae	*Hyeronima alchorneoides*	FJ038027	rbcL
Phyllanthaceae	*Hyeronima alchorneoides*	FJ038028	rbcL
Phyllanthaceae	*Hyeronima alchorneoides*	JQ626093	rbcL
Phyllanthaceae	*Hyeronima alchorneoides*	FJ817163	rpoB
Phyllanthaceae	*Hyeronima alchorneoides*	FJ817218	rpoB
Phyllanthaceae	*Hyeronima alchorneoides*	FJ039322	trnL
Phyllanthaceae	*Hyeronima alchorneoides*	FJ039323	trnL
Phyllanthaceae	*Hyeronima oblonga*	AY830268	matK
Phyllanthaceae	*Hyeronima oblonga*	AY663588	rbcL
Phyllanthaceae	*Hyeronima oblonga*	AY830387	rbcL
Phyllanthaceae	*Margaritaria nobilis*	GQ982040	matK
Phyllanthaceae	*Margaritaria nobilis*	HM446709	matK
Phyllanthaceae	*Margaritaria nobilis*	JQ587467	matK
Phyllanthaceae	*Margaritaria nobilis*	JQ587468	matK
Phyllanthaceae	*Margaritaria nobilis*	JQ588585	matK
Phyllanthaceae	*Margaritaria nobilis*	GQ982281	psbA-trnH
Phyllanthaceae	*Margaritaria nobilis*	HG963933	psbA-trnH

Family	Species	Accession	Barcode
Phyllanthaceae	*Margaritaria nobilis*	HM446955	psbA-trnH
Phyllanthaceae	*Margaritaria nobilis*	GQ981795	rbcL
Phyllanthaceae	*Margaritaria nobilis*	HM446823	rbcL
Phyllanthaceae	*Margaritaria nobilis*	JQ591498	rbcL
Phyllanthaceae	*Margaritaria nobilis*	JQ591499	rbcL
Phyllanthaceae	*Margaritaria nobilis*	JQ593115	rbcL
Phyllanthaceae	*Margaritaria nobilis*	JQ593116	rbcL
Phyllanthaceae	*Phyllanthus acuminatus*	AY936667	ITS
Phyllanthaceae	*Phyllanthus acuminatus*	AY936573	matK
Phyllanthaceae	*Phyllanthus juglandifolius*	AY936698	ITS
Phyllanthaceae	*Phyllanthus juglandifolius*	AY936699	ITS
Phyllanthaceae	*Phyllanthus juglandifolius*	AY830277	matK
Phyllanthaceae	*Phyllanthus juglandifolius*	AY936602	matK
Phyllanthaceae	*Phyllanthus juglandifolius*	KJ426869	psbA-trnH
Phyllanthaceae	*Phyllanthus juglandifolius*	AY663607	rbcL
Phyllanthaceae	*Richeria grandis*	AY830281	matK
Phyllanthaceae	*Richeria grandis*	AY663616	rbcL
Phyllanthaceae	*Savia dictyocarpa*	AY552449	matK
Phyllanthaceae	*Savia dictyocarpa*	AY663619	rbcL
Phytolaccaceae	*Gallesia integrifolia*	JX232590	ITS
Phytolaccaceae	*Gallesia integrifolia*	AY042590	matK
Phytolaccaceae	*Phytolacca dioica*	AJ532595	ITS
Phytolaccaceae	*Phytolacca dioica*	AJ577757	ITS
Phytolaccaceae	*Phytolacca dioica*	JX232571	ITS
Phytolaccaceae	*Phytolacca dioica*	AY042631	matK
Phytolaccaceae	*Phytolacca dioica*	JX495745	matK
Phytolaccaceae	*Phytolacca dioica*	JX517912	matK
Phytolaccaceae	*Phytolacca dioica*	JX571876	rbcL
Phytolaccaceae	*Phytolacca dioica*	JX572856	rbcL
Phytolaccaceae	*Phytolacca dioica*	AJ558037	trnL
Phytolaccaceae	*Phytolacca dioica*	KM261955	trnL
Phytolaccaceae	*Phytolacca dioica*	KM262029	trnL
Pinaceae	*Pinus caribaea*	AB063498	matK
Pinaceae	*Pinus caribaea*	AB063500	matK
Pinaceae	*Pinus caribaea*	AB063517	matK

Family	Species	Accession	Barcode
Pinaceae	*Pinus caribaea*	AB080940	matK
Pinaceae	*Pinus caribaea*	AB080941	matK
Pinaceae	*Pinus caribaea*	AB080942	matK
Pinaceae	*Pinus caribaea*	AY497280	matK
Pinaceae	*Pinus caribaea*	KC156990	matK
Pinaceae	*Pinus caribaea*	KC157001	matK
Pinaceae	*Pinus caribaea*	KC157011	matK
Pinaceae	*Pinus caribaea*	KC157327	psbA-trnH
Pinaceae	*Pinus caribaea*	KC157346	psbA-trnH
Pinaceae	*Pinus caribaea*	KC157363	psbA-trnH
Pinaceae	*Pinus caribaea*	AB063366	rbcL
Pinaceae	*Pinus caribaea*	AB063368	rbcL
Pinaceae	*Pinus caribaea*	AB063385	rbcL
Pinaceae	*Pinus caribaea*	AY497244	rbcL
Pinaceae	*Pinus caribaea*	KC156830	rbcL
Pinaceae	*Pinus caribaea*	KC156849	rbcL
Pinaceae	*Pinus caribaea*	KC156866	rbcL
Pinaceae	*Pinus caribaea*	KJ082496	rbcL
Pinaceae	*Pinus caribaea*	AM883744	rpoB
Pinaceae	*Pinus caribaea*	AM883766	rpoB
Pinaceae	*Pinus caribaea*	AM883426	rpoC1
Pinaceae	*Pinus caribaea*	AM883701	rpoC1
Pinaceae	*Pinus caribaea*	AB081137	trnL
Pinaceae	*Pinus caribaea*	AB081138	trnL
Pinaceae	*Pinus caribaea*	AB081139	trnL
Pinaceae	*Pinus caribaea*	AF528522	trnL
Pinaceae	*Pinus caribaea*	KC157162	ycf1
Pinaceae	*Pinus caribaea*	KC157173	ycf1
Pinaceae	*Pinus caribaea*	KC157184	ycf1
Pinaceae	*Pinus elliottii*	AB080931	matK
Pinaceae	*Pinus elliottii*	AY724747	matK
Pinaceae	*Pinus elliottii*	FM955321	matK
Pinaceae	*Pinus elliottii*	KC156933	matK
Pinaceae	*Pinus elliottii*	KC156952	matK
Pinaceae	*Pinus elliottii*	KC157015	matK

Family	Species	Accession	Barcode
Pinaceae	*Pinus elliottii*	KC157241	psbA-trnH
Pinaceae	*Pinus elliottii*	KC157267	psbA-trnH
Pinaceae	*Pinus elliottii*	KC157299	psbA-trnH
Pinaceae	*Pinus elliottii*	KC157367	psbA-trnH
Pinaceae	*Pinus elliottii*	AB081075	rbcL
Pinaceae	*Pinus elliottii*	AY724755	rbcL
Pinaceae	*Pinus elliottii*	KC156744	rbcL
Pinaceae	*Pinus elliottii*	KC156770	rbcL
Pinaceae	*Pinus elliottii*	KC156802	rbcL
Pinaceae	*Pinus elliottii*	KC156870	rbcL
Pinaceae	*Pinus elliottii*	AM883738	rpoB
Pinaceae	*Pinus elliottii*	AM883417	rpoC1
Pinaceae	*Pinus elliottii*	AB081144	trnL
Pinaceae	*Pinus elliottii*	AF343578	trnL
Pinaceae	*Pinus elliottii*	AF528523	trnL
Pinaceae	*Pinus elliottii*	KC157104	ycf1
Pinaceae	*Pinus elliottii*	KC157123	ycf1
Pinaceae	*Pinus elliottii*	KC157144	ycf1
Pinaceae	*Pinus elliottii*	KC157188	ycf1
Pinaceae	*Pinus taeda*	KJ661363	atpF-atpH
Pinaceae	*Pinus taeda*	KC156911	matK
Pinaceae	*Pinus taeda*	KC157023	matK
Pinaceae	*Pinus taeda*	KC157213	psbA-trnH
Pinaceae	*Pinus taeda*	KC157383	psbA-trnH
Pinaceae	*Pinus taeda*	KJ661381	psbA-trnH
Pinaceae	*Pinus taeda*	KC156716	rbcL
Pinaceae	*Pinus taeda*	KC156886	rbcL
Pinaceae	*Pinus taeda*	KC157082	ycf1
Pinaceae	*Pinus taeda*	KC157197	ycf1
Piperaceae	*Piper aduncum*	AF275157	ITS
Piperaceae	*Piper aduncum*	AF275158	ITS
Piperaceae	*Piper aduncum*	AF275159	ITS
Piperaceae	*Piper aduncum*	AM901426	ITS
Piperaceae	*Piper aduncum*	DQ868701	ITS
Piperaceae	*Piper aduncum*	EF060061	ITS

**Family**	**Species**	**Accession**	**Barcode**
Piperaceae	*Piper aduncum*	KF924116	ITS
Piperaceae	*Piper aduncum*	DQ882201	matK
Piperaceae	*Piper aduncum*	DQ882202	matK
Piperaceae	*Piper aduncum*	JQ588613	matK
Piperaceae	*Piper aduncum*	JQ588614	matK
Piperaceae	*Piper aduncum*	JQ588615	matK
Piperaceae	*Piper aduncum*	HG963908	psbA-trnH
Piperaceae	*Piper aduncum*	HM446980	psbA-trnH
Piperaceae	*Piper aduncum*	AY572252	rbcL
Piperaceae	*Piper aduncum*	FJ976157	rbcL
Piperaceae	*Piper aduncum*	HM446849	rbcL
Piperaceae	*Piper aduncum*	JQ593170	rbcL
Piperaceae	*Piper aduncum*	JQ593171	rbcL
Piperaceae	*Piper aduncum*	JQ593172	rbcL
Piperaceae	*Piper aduncum*	EU519629	trnL
Piperaceae	*Piper aduncum*	EU519630	trnL
Piperaceae	*Piper aduncum*	EU519809	trnL
Piperaceae	*Piper aduncum*	EU519810	trnL
Piperaceae	*Piper amalago*	AF275186	ITS
Piperaceae	*Piper amalago*	DQ868703	ITS
Piperaceae	*Piper amalago*	JQ588620	matK
Piperaceae	*Piper amalago*	JQ588621	matK
Piperaceae	*Piper amalago*	JQ588622	matK
Piperaceae	*Piper amalago*	JQ588623	matK
Piperaceae	*Piper amalago*	JQ588624	matK
Piperaceae	*Piper amalago*	JQ588625	matK
Piperaceae	*Piper amalago*	KJ012718	matK
Piperaceae	*Piper amalago*	HG963748	psbA-trnH
Piperaceae	*Piper amalago*	KJ426879	psbA-trnH
Piperaceae	*Piper amalago*	JQ593177	rbcL
Piperaceae	*Piper amalago*	JQ593178	rbcL
Piperaceae	*Piper amalago*	JQ593179	rbcL
Piperaceae	*Piper amalago*	JQ593180	rbcL
Piperaceae	*Piper amalago*	JQ593181	rbcL
Piperaceae	*Piper amalago*	JQ593182	rbcL

Family	Species	Accession	Barcode
Piperaceae	*Piper amalago*	JQ593183	rbcL
Piperaceae	*Piper amalago*	JQ593184	rbcL
Piperaceae	*Piper amalago*	JQ593185	rbcL
Piperaceae	*Piper amalago*	JQ593186	rbcL
Piperaceae	*Piper amalago*	JQ593187	rbcL
Piperaceae	*Piper amalago*	JQ593188	rbcL
Piperaceae	*Piper amalago*	JQ593189	rbcL
Piperaceae	*Piper amalago*	JQ593190	rbcL
Piperaceae	*Piper amalago*	KJ082498	rbcL
Piperaceae	*Piper amalago*	EU519633	trnL
Piperaceae	*Piper amalago*	EU519813	trnL
Piperaceae	*Piper arboreum*	AF275180	ITS
Piperaceae	*Piper arboreum*	AM901427	ITS
Piperaceae	*Piper arboreum*	EF056221	ITS
Piperaceae	*Piper arboreum*	EF056222	ITS
Piperaceae	*Piper arboreum*	EF056223	ITS
Piperaceae	*Piper arboreum*	EF056224	ITS
Piperaceae	*Piper arboreum*	GQ982316	psbA-trnH
Piperaceae	*Piper arboreum*	HG963563	psbA-trnH
Piperaceae	*Piper arboreum*	GQ981830	rbcL
Piperaceae	*Piper arboreum*	EU519614	trnL
Piperaceae	*Piper arboreum*	EU519615	trnL
Piperaceae	*Piper arboreum*	EU519794	trnL
Piperaceae	*Piper arboreum*	EU519795	trnL
Piperaceae	*Piper cernuum*	EF056242	ITS
Piperaceae	*Piper cernuum*	EF056259	ITS
Piperaceae	*Piper corcovadensis*	KJ930391	matK
Piperaceae	*Piper hispidum*	AF275156	ITS
Piperaceae	*Piper hispidum*	AM901423	ITS
Piperaceae	*Piper hispidum*	DQ868721	ITS
Piperaceae	*Piper hispidum*	EF060071	ITS
Piperaceae	*Piper hispidum*	EF060072	ITS
Piperaceae	*Piper hispidum*	DQ882219	matK
Piperaceae	*Piper hispidum*	HM446983	psbA-trnH
Piperaceae	*Piper hispidum*	AY572249	rbcL

**Family**	**Species**	**Accession**	**Barcode**
Piperaceae	*Piper hispidum*	HM446852	rbcL
Piperaceae	*Piper hispidum*	EU519631	trnL
Piperaceae	*Piper hispidum*	EU519811	trnL
Piperaceae	*Piper obliquum*	EF056272	ITS
Piperaceae	*Piper obliquum*	EF056273	ITS
Piperaceae	*Piper obliquum*	EF056274	ITS
Piperaceae	*Piper obliquum*	HG963688	psbA-trnH
Piperaceae	*Piper obliquum*	EU519627	trnL
Piperaceae	*Piper obliquum*	EU519807	trnL
Piperaceae	*Piper tuberculatum*	AM901422	ITS
Piperaceae	*Piper tuberculatum*	AY326223	ITS
Piperaceae	*Piper tuberculatum*	EF056292	ITS
Piperaceae	*Piper tuberculatum*	EF056293	ITS
Piperaceae	*Piper tuberculatum*	HG963555	psbA-trnH
Piperaceae	*Piper tuberculatum*	HG963639	psbA-trnH
Piperaceae	*Piper tuberculatum*	JQ593251	rbcL
Piperaceae	*Piper tuberculatum*	JQ593252	rbcL
Pittosporaceae	*Pittosporum undulatum*	AF302014	ITS
Pittosporaceae	*Pittosporum undulatum*	HM116994	ITS
Pittosporaceae	*Pittosporum undulatum*	AJ429374	matK
Pittosporaceae	*Pittosporum undulatum*	DQ133794	matK
Pittosporaceae	*Pittosporum undulatum*	HM850707	matK
Pittosporaceae	*Pittosporum undulatum*	KM894453	matK
Pittosporaceae	*Pittosporum undulatum*	KM894821	matK
Pittosporaceae	*Pittosporum undulatum*	KM895297	psbA-trnH
Pittosporaceae	*Pittosporum undulatum*	HM850262	rbcL
Pittosporaceae	*Pittosporum undulatum*	KM895533	rbcL
Pittosporaceae	*Pittosporum undulatum*	KM895988	rbcL
Pittosporaceae	*Pittosporum undulatum*	AJ430960	trnL
Poaceae	*Actinocladum verticillatum*	JQ408586	trnL
Poaceae	*Apoclada simplex*	JQ408589	trnL
Poaceae	*Apoclada simplex*	KC020545	trnL
Poaceae	*Aulonemia amplissima*	JQ408598	trnL
Poaceae	*Bambusa multiplex*	AY839710	ITS
Poaceae	*Bambusa multiplex*	DQ270126	ITS

Family	Species	Accession	Barcode
Poaceae	*Bambusa multiplex*	EF450229	ITS
Poaceae	*Bambusa multiplex*	FJ410317	ITS
Poaceae	*Bambusa multiplex*	GQ464807	ITS
Poaceae	*Bambusa multiplex*	EF125166	matK
Poaceae	*Bambusa multiplex*	GU063085	psbA-trnH
Poaceae	*Bambusa multiplex*	GU390999	psbA-trnH
Poaceae	*Bambusa multiplex*	AJ746170	rbcL
Poaceae	*Bambusa multiplex*	M91626	rbcL
Poaceae	*Bambusa multiplex*	DQ137347	trnL
Poaceae	*Bambusa tulda*	EF540854	ITS
Poaceae	*Bambusa tulda*	EU434248	matK
Poaceae	*Bambusa tulda*	JX966239	matK
Poaceae	*Bambusa tulda*	GU063086	psbA-trnH
Poaceae	*Bambusa tulda*	KC150893	psbA-trnH
Poaceae	*Bambusa tulda*	KC123349	rbcL
Poaceae	*Bambusa tulda*	EU434056	trnL
Poaceae	*Bambusa tulda*	JX507132	trnL
Poaceae	*Bambusa tuldoides*	AY839708	ITS
Poaceae	*Bambusa tuldoides*	HM448937	matK
Poaceae	*Bambusa tuldoides*	GU063083	psbA-trnH
Poaceae	*Bambusa tuldoides*	GU391001	psbA-trnH
Poaceae	*Bambusa tuldoides*	KC150898	psbA-trnH
Poaceae	*Bambusa tuldoides*	KM437945	psbA-trnH
Poaceae	*Bambusa tuldoides*	KM437946	psbA-trnH
Poaceae	*Bambusa tuldoides*	KM437947	psbA-trnH
Poaceae	*Bambusa tuldoides*	KM437948	psbA-trnH
Poaceae	*Bambusa tuldoides*	KM437949	psbA-trnH
Poaceae	*Bambusa tuldoides*	KM437950	psbA-trnH
Poaceae	*Bambusa tuldoides*	KM437951	psbA-trnH
Poaceae	*Bambusa tuldoides*	HM448967	trnL
Poaceae	*Bambusa tuldoides*	KC013288	trnL
Poaceae	*Bambusa ventricosa*	JX428395	matK
Poaceae	*Bambusa ventricosa*	JX428396	matK
Poaceae	*Bambusa ventricosa*	JX428397	matK
Poaceae	*Bambusa ventricosa*	JX428398	matK

Family	Species	Accession	Barcode
Poaceae	*Bambusa ventricosa*	JX428399	matK
Poaceae	*Bambusa ventricosa*	JX428400	matK
Poaceae	*Bambusa ventricosa*	JX428401	matK
Poaceae	*Bambusa ventricosa*	JX428402	matK
Poaceae	*Bambusa ventricosa*	GU063074	psbA-trnH
Poaceae	*Bambusa ventricosa*	JX428414	psbA-trnH
Poaceae	*Bambusa ventricosa*	JX428415	psbA-trnH
Poaceae	*Bambusa ventricosa*	JX428416	psbA-trnH
Poaceae	*Bambusa ventricosa*	JX428417	psbA-trnH
Poaceae	*Bambusa ventricosa*	JX428418	psbA-trnH
Poaceae	*Bambusa ventricosa*	JX428419	psbA-trnH
Poaceae	*Bambusa ventricosa*	JX428420	psbA-trnH
Poaceae	*Bambusa ventricosa*	JX428421	psbA-trnH
Poaceae	*Bambusa ventricosa*	KF796694	rbcL
Poaceae	*Bambusa ventricosa*	GU354998	trnL
Poaceae	*Bambusa ventricosa*	JX428433	trnL
Poaceae	*Bambusa ventricosa*	JX428434	trnL
Poaceae	*Bambusa ventricosa*	JX428435	trnL
Poaceae	*Bambusa ventricosa*	JX428436	trnL
Poaceae	*Bambusa ventricosa*	JX428437	trnL
Poaceae	*Bambusa ventricosa*	JX428438	trnL
Poaceae	*Bambusa ventricosa*	JX428439	trnL
Poaceae	*Bambusa ventricosa*	JX428440	trnL
Poaceae	*Bambusa vulgaris*	AY839705	ITS
Poaceae	*Bambusa vulgaris*	FJ410314	ITS
Poaceae	*Bambusa vulgaris*	HE574450	matK
Poaceae	*Bambusa vulgaris*	JX428387-JX428394	matK
Poaceae	*Bambusa vulgaris*	JX428403	matK
Poaceae	*Bambusa vulgaris*	JX428404	matK
Poaceae	*Bambusa vulgaris*	JX428405	matK
Poaceae	*Bambusa vulgaris*	JX966235	matK
Poaceae	*Bambusa vulgaris*	JX966238	matK
Poaceae	*Bambusa vulgaris*	KF364961	matK
Poaceae	*Bambusa vulgaris*	KP093727	matK
Poaceae	*Bambusa vulgaris*	KP093728	matK

Family	Species	Accession	Barcode
Poaceae	*Bambusa vulgaris*	EF589631	psbA-trnH
Poaceae	*Bambusa vulgaris*	FJ644251	psbA-trnH
Poaceae	*Bambusa vulgaris*	GU063097	psbA-trnH
Poaceae	*Bambusa vulgaris*	JX428406-JX428413	psbA-trnH
Poaceae	*Bambusa vulgaris*	JX428422-JX428424	psbA-trnH
Poaceae	*Bambusa vulgaris*	KC150892	psbA-trnH
Poaceae	*Bambusa vulgaris*	KC150894	psbA-trnH
Poaceae	*Bambusa vulgaris*	KP095787	psbA-trnH
Poaceae	*Bambusa vulgaris*	KP095788	psbA-trnH
Poaceae	*Bambusa vulgaris*	HE573326	rbcL
Poaceae	*Bambusa vulgaris*	JQ734486	rbcL
Poaceae	*Bambusa vulgaris*	JX571783	rbcL
Poaceae	*Bambusa vulgaris*	KF796691	rbcL
Poaceae	*Bambusa vulgaris*	KP094664	rbcL
Poaceae	*Bambusa vulgaris*	KP094665	rbcL
Poaceae	*Bambusa vulgaris*	EF137524	trnL
Poaceae	*Bambusa vulgaris*	FJ644133	trnL
Poaceae	*Bambusa vulgaris*	JX428425-JX428432	trnL
Poaceae	*Bambusa vulgaris*	JX428441-JX428443	trnL
Poaceae	*Bambusa vulgaris*	JX564906	trnL
Poaceae	*Bambusa vulgaris*	KF797196	trnL
Poaceae	*Chusquea capitata*	KR061462	ITS
Poaceae	*Chusquea capitata*	KR061463	ITS
Poaceae	*Chusquea capituliflora*	KR061472	ITS
Poaceae	*Chusquea capituliflora*	KR061473	ITS
Poaceae	*Chusquea capituliflora*	KR061474	ITS
Poaceae	*Chusquea capituliflora*	KR061475	ITS
Poaceae	*Chusquea capituliflora*	KR061476	ITS
Poaceae	*Chusquea capituliflora*	KR061480	ITS
Poaceae	*Chusquea juergensii*	EU503054	atpF-atpH
Poaceae	*Chusquea juergensii*	KF945235	ITS
Poaceae	*Chusquea juergensii*	KF945342	trnL
Poaceae	*Chusquea urelytra*	KR061477	ITS
Poaceae	*Chusquea urelytra*	KR061483	ITS
Poaceae	*Chusquea urelytra*	KF945382	trnL

Family	Species	Accession	Barcode
Poaceae	*Guadua chacoensis*	KF796737	rbcL
Poaceae	*Guadua chacoensis*	KF797232	trnL
Poaceae	*Guadua paniculata*	HQ847211	matK
Poaceae	*Guadua paniculata*	JQ588718	matK
Poaceae	*Guadua paniculata*	JQ588719	matK
Poaceae	*Guadua paniculata*	EF589639	psbA-trnH
Poaceae	*Guadua paniculata*	HQ847344	psbK-psbI
Poaceae	*Guadua paniculata*	HQ847278	rbcL
Poaceae	*Guadua paniculata*	JQ593324	rbcL
Poaceae	*Guadua paniculata*	JQ593325	rbcL
Poaceae	*Guadua paniculata*	JQ594900	rbcL
Poaceae	*Guadua velutina*	EF589640	psbA-trnH
Poaceae	*Merostachys ternata*	JQ408610	trnL
Poaceae	*Phyllostachys edulis*	EU434242	matK
Poaceae	*Phyllostachys edulis*	JN247141	matK
Poaceae	*Phyllostachys edulis*	JN247142	matK
Poaceae	*Phyllostachys edulis*	JN247143	matK
Poaceae	*Phyllostachys edulis*	JN247331	psbA-trnH
Poaceae	*Phyllostachys edulis*	JN247332	psbA-trnH
Poaceae	*Phyllostachys edulis*	JN247333	psbA-trnH
Poaceae	*Phyllostachys edulis*	JN247236	rbcL
Poaceae	*Phyllostachys edulis*	JN247237	rbcL
Poaceae	*Phyllostachys edulis*	JN247238	rbcL
Poaceae	*Phyllostachys edulis*	EU434050	trnL
Poaceae	*Phyllostachys edulis*	GU354974	trnL
Podocarpaceae	*Podocarpus lambertii*	JF969579	ITS
Podocarpaceae	*Podocarpus lambertii*	JF969580	ITS
Podocarpaceae	*Podocarpus lambertii*	KF713936	ITS
Podocarpaceae	*Podocarpus lambertii*	KF713937	ITS
Podocarpaceae	*Podocarpus lambertii*	KF713938	ITS
Podocarpaceae	*Podocarpus lambertii*	HM593753	matK
Podocarpaceae	*Podocarpus lambertii*	KF713712	matK
Podocarpaceae	*Podocarpus lambertii*	KF713713	matK
Podocarpaceae	*Podocarpus lambertii*	KF713714	matK
Podocarpaceae	*Podocarpus lambertii*	KF421017	psbA-trnH

**Family**	**Species**	**Accession**	**Barcode**
Podocarpaceae	*Podocarpus lambertii*	KF421018	psbA-trnH
Podocarpaceae	*Podocarpus lambertii*	KF421019	psbA-trnH
Podocarpaceae	*Podocarpus lambertii*	HM593650	rbcL
Podocarpaceae	*Podocarpus lambertii*	JF969700	rbcL
Podocarpaceae	*Podocarpus lambertii*	KF561962	rbcL
Podocarpaceae	*Podocarpus lambertii*	KF561963	rbcL
Podocarpaceae	*Podocarpus lambertii*	KF714137	rbcL
Podocarpaceae	*Podocarpus lambertii*	KF714138	rbcL
Podocarpaceae	*Podocarpus lambertii*	KF714139	rbcL
Podocarpaceae	*Podocarpus lambertii*	JN001374	trnL
Podocarpaceae	*Podocarpus lambertii*	JN001450	trnL
Podocarpaceae	*Podocarpus sellowii*	JF969586	ITS
Podocarpaceae	*Podocarpus sellowii*	KF713994	ITS
Podocarpaceae	*Podocarpus sellowii*	KF713995	ITS
Podocarpaceae	*Podocarpus sellowii*	KF713996	ITS
Podocarpaceae	*Podocarpus sellowii*	HM593778	matK
Podocarpaceae	*Podocarpus sellowii*	KF713775	matK
Podocarpaceae	*Podocarpus sellowii*	KF713776	matK
Podocarpaceae	*Podocarpus sellowii*	KF713777	matK
Podocarpaceae	*Podocarpus sellowii*	HM593674	rbcL
Podocarpaceae	*Podocarpus sellowii*	JF969730	rbcL
Podocarpaceae	*Podocarpus sellowii*	KF714191	rbcL
Podocarpaceae	*Podocarpus sellowii*	KF714192	rbcL
Podocarpaceae	*Podocarpus sellowii*	KF714193	rbcL
Podocarpaceae	*Podocarpus sellowii*	JN001395	trnL
Podocarpaceae	*Podocarpus sellowii*	JN001471	trnL
Polygalaceae	*Acanthocladus brasiliensis*	GQ888974	ITS
Polygalaceae	*Acanthocladus brasiliensis*	AM234209	rbcL
Polygalaceae	*Acanthocladus brasiliensis*	AF366973	trnL
Polygalaceae	*Acanthocladus brasiliensis*	GQ889153	trnL
Polygalaceae	*Asemeia acuminata*	GQ888918	ITS
Polygalaceae	*Asemeia acuminata*	GQ889097	trnL
Polygalaceae	*Bredemeyera floribunda*	GQ888883	ITS
Polygalaceae	*Bredemeyera floribunda*	EU596520	matK
Polygalaceae	*Bredemeyera floribunda*	AM234172	rbcL

Family	Species	Accession	Barcode
Polygalaceae	*Bredemeyera floribunda*	EU644699	rbcL
Polygalaceae	*Bredemeyera floribunda*	AF366945	trnL
Polygalaceae	*Bredemeyera floribunda*	GQ888773	trnL
Polygalaceae	*Bredemeyera floribunda*	GQ889062	trnL
Polygonaceae	*Coccoloba mollis*	JQ626541	matK
Polygonaceae	*Coccoloba mollis*	JQ626225	rbcL
Polygonaceae	*Ruprechtia laxiflora*	AY256535	ITS
Polygonaceae	*Ruprechtia laxiflora*	FJ154484	ITS
Polygonaceae	*Ruprechtia laxiflora*	HM137444	ITS
Polygonaceae	*Ruprechtia laxiflora*	EF437987	rbcL
Polygonaceae	*Triplaris americana*	FJ154486	ITS
Polygonaceae	*Triplaris americana*	KP271195	ITS
Polygonaceae	*Triplaris americana*	KP271196	ITS
Polygonaceae	*Triplaris americana*	KP271198	ITS
Polygonaceae	*Triplaris americana*	KP271199	ITS
Polygonaceae	*Triplaris americana*	KP271200	ITS
Polygonaceae	*Triplaris americana*	KP271204	ITS
Polygonaceae	*Triplaris americana*	KP271210	ITS
Polygonaceae	*Triplaris americana*	KP271213	ITS
Polygonaceae	*Triplaris americana*	AY042668	matK
Polygonaceae	*Triplaris americana*	KP236817	psbA-trnH
Polygonaceae	*Triplaris americana*	KP236819	psbA-trnH
Polygonaceae	*Triplaris americana*	KP236820	psbA-trnH
Polygonaceae	*Triplaris americana*	KP236823	psbA-trnH
Polygonaceae	*Triplaris americana*	KP236827	psbA-trnH
Polygonaceae	*Triplaris americana*	KP236832	psbA-trnH
Polygonaceae	*Triplaris americana*	KP236834	psbA-trnH
Polygonaceae	*Triplaris americana*	KP236838	psbA-trnH
Polygonaceae	*Triplaris americana*	Y16910	rbcL
Polygonaceae	*Triplaris americana*	AJ312251	trnL
Polygonaceae	*Triplaris gardneriana*	AY256520	ITS
Polygonaceae	*Triplaris gardneriana*	KP271192	ITS
Polygonaceae	*Triplaris gardneriana*	KP236828	psbA-trnH
Primulaceae	*Clavija spinosa*	AF402450	trnL
Primulaceae	*Myrsine coriacea*	JQ588471	matK

**Family**	**Species**	**Accession**	**Barcode**
Primulaceae	*Myrsine coriacea*	JQ588472	matK
Primulaceae	*Myrsine coriacea*	JQ588473	matK
Primulaceae	*Myrsine coriacea*	JQ589927	matK
Primulaceae	*Myrsine coriacea*	KJ012692	matK
Primulaceae	*Myrsine coriacea*	HG963840	psbA-trnH
Primulaceae	*Myrsine coriacea*	KJ426840	psbA-trnH
Primulaceae	*Myrsine coriacea*	HM446839	rbcL
Primulaceae	*Myrsine coriacea*	JQ592926	rbcL
Primulaceae	*Myrsine coriacea*	JQ592927	rbcL
Primulaceae	*Myrsine coriacea*	JQ592928	rbcL
Primulaceae	*Myrsine coriacea*	JQ592929	rbcL
Primulaceae	*Myrsine coriacea*	JQ592930	rbcL
Primulaceae	*Myrsine coriacea*	JQ594922	rbcL
Primulaceae	*Myrsine coriacea*	JQ594923	rbcL
Primulaceae	*Myrsine coriacea*	KJ082452	rbcL
Primulaceae	*Myrsine coriacea*	Z80204	rbcL
Primulaceae	*Myrsine guianensis*	JF416237	ITS
Primulaceae	*Myrsine guianensis*	JF416243	ITS
Primulaceae	*Myrsine guianensis*	GQ981939	matK
Primulaceae	*Myrsine guianensis*	JF416269	matK
Primulaceae	*Myrsine guianensis*	JF416280	matK
Primulaceae	*Myrsine guianensis*	GQ981670	rbcL
Primulaceae	*Myrsine guianensis*	JF416258	trnL
Primulaceae	*Myrsine guianensis*	JF416263	trnL
Primulaceae	*Myrsine umbellata*	KF420974	ITS
Primulaceae	*Myrsine umbellata*	KF420975	ITS
Primulaceae	*Myrsine umbellata*	KF420976	ITS
Primulaceae	*Myrsine umbellata*	KF555417	matK
Primulaceae	*Myrsine umbellata*	KF555418	matK
Primulaceae	*Myrsine umbellata*	KF555419	matK
Primulaceae	*Myrsine umbellata*	KF421094	psbA-trnH
Primulaceae	*Myrsine umbellata*	KF421095	psbA-trnH
Primulaceae	*Myrsine umbellata*	KF421096	psbA-trnH
Primulaceae	*Myrsine umbellata*	KF561936	rbcL
Primulaceae	*Myrsine umbellata*	KF561937	rbcL

Family	Species	Accession	Barcode
Primulaceae	*Myrsine umbellata*	KF561938	rbcL
Proteaceae	*Grevillea robusta*	AY864893	ITS
Proteaceae	*Grevillea robusta*	DQ499132	ITS
Proteaceae	*Grevillea robusta*	EU169631	matK
Proteaceae	*Grevillea robusta*	FJ626529	matK
Proteaceae	*Grevillea robusta*	JX495722	matK
Proteaceae	*Grevillea robusta*	KM894579	matK
Proteaceae	*Grevillea robusta*	KP089098	matK
Proteaceae	*Grevillea robusta*	KT454609	matK
Proteaceae	*Grevillea robusta*	KM895122	psbA-trnH
Proteaceae	*Grevillea robusta*	AF193973	rbcL
Proteaceae	*Grevillea robusta*	AF197589	rbcL
Proteaceae	*Grevillea robusta*	JQ734508	rbcL
Proteaceae	*Grevillea robusta*	JQ734509	rbcL
Proteaceae	*Grevillea robusta*	JX571846	rbcL
Proteaceae	*Grevillea robusta*	KM895690	rbcL
Proteaceae	*Grevillea robusta*	FJ626569	trnL
Proteaceae	*Grevillea robusta*	KP088415	ycf1
Proteaceae	*Roupala montana*	EU676097	ITS
Proteaceae	*Roupala montana*	KF420945	ITS
Proteaceae	*Roupala montana*	KM519463	ITS
Proteaceae	*Roupala montana*	EU169661	matK
Proteaceae	*Roupala montana*	EU642684	matK
Proteaceae	*Roupala montana*	JQ588871	matK
Proteaceae	*Roupala montana*	JQ588872	matK
Proteaceae	*Roupala montana*	JQ588873	matK
Proteaceae	*Roupala montana*	JQ588874	matK
Proteaceae	*Roupala montana*	JQ588875	matK
Proteaceae	*Roupala montana*	JQ588876	matK
Proteaceae	*Roupala montana*	JQ588877	matK
Proteaceae	*Roupala montana*	JQ588878	matK
Proteaceae	*Roupala montana*	JQ588879	matK
Proteaceae	*Roupala montana*	JQ588880	matK
Proteaceae	*Roupala montana*	JQ588881	matK
Proteaceae	*Roupala montana*	JQ589707	matK

Family	Species	Accession	Barcode
Proteaceae	*Roupala montana*	JQ589708	matK
Proteaceae	*Roupala montana*	JQ589709	matK
Proteaceae	*Roupala montana*	KF555444	matK
Proteaceae	*Roupala montana*	KF421048	psbA-trnH
Proteaceae	*Roupala montana*	JQ593558	rbcL
Proteaceae	*Roupala montana*	JQ593559	rbcL
Proteaceae	*Roupala montana*	JQ593560	rbcL
Proteaceae	*Roupala montana*	JQ593561	rbcL
Proteaceae	*Roupala montana*	JQ593562	rbcL
Proteaceae	*Roupala montana*	JQ593563	rbcL
Proteaceae	*Roupala montana*	JQ593564	rbcL
Proteaceae	*Roupala montana*	JQ593565	rbcL
Proteaceae	*Roupala montana*	JQ593566	rbcL
Proteaceae	*Roupala montana*	JQ593567	rbcL
Proteaceae	*Roupala montana*	JQ593568	rbcL
Proteaceae	*Roupala montana*	JQ593569	rbcL
Proteaceae	*Roupala montana*	JQ593570	rbcL
Proteaceae	*Roupala montana*	JQ593571	rbcL
Proteaceae	*Roupala montana*	JQ594703	rbcL
Proteaceae	*Roupala montana*	JQ594704	rbcL
Proteaceae	*Roupala montana*	JQ594705	rbcL
Proteaceae	*Roupala montana*	JQ594706	rbcL
Proteaceae	*Roupala montana*	JQ594707	rbcL
Proteaceae	*Roupala montana*	JQ594708	rbcL
Proteaceae	*Roupala montana*	KF561966	rbcL
Proteaceae	*Roupala montana*	AF482144	trnL
Proteaceae	*Roupala montana*	AF482189	trnL
Rhamnaceae	*Colubrina glandulosa*	JQ593574	rbcL
Rhamnaceae	*Colubrina glandulosa*	JQ593575	rbcL
Rhamnaceae	*Colubrina glandulosa*	JQ593576	rbcL
Rhamnaceae	*Hovenia dulcis*	DQ146607	ITS
Rhamnaceae	*Hovenia dulcis*	KT159700	ITS
Rhamnaceae	*Hovenia dulcis*	JX495724	matK
Rhamnaceae	*Hovenia dulcis*	AJ390039	rbcL
Rhamnaceae	*Hovenia dulcis*	JX571848	rbcL

Family	Species	Accession	Barcode
Rhamnaceae	*Hovenia dulcis*	AJ390343	trnL
Rhamnaceae	*Hovenia dulcis*	DQ146563	trnL
Rhamnaceae	*Hovenia dulcis*	DQ146564	trnL
Rhamnaceae	*Hovenia dulcis*	KP299394	trnL
Rhamnaceae	*Rhamnidium elaeocarpum*	AY626452	ITS
Rhamnaceae	*Rhamnidium elaeocarpum*	AJ390030	rbcL
Rhamnaceae	*Rhamnidium elaeocarpum*	AJ390332	trnL
Rhamnaceae	*Rhamnidium glabrum*	JN900286	ITS
Rhamnaceae	*Rhamnidium glabrum*	JN900324	trnL
Rhamnaceae	*Rhamnus sphaerosperma*	KJ012751	matK
Rhamnaceae	*Rhamnus sphaerosperma*	KJ426915	psbA-trnH
Rhamnaceae	*Rhamnus sphaerosperma*	KP299593	psbA-trnH
Rhamnaceae	*Rhamnus sphaerosperma*	KJ082543	rbcL
Rhamnaceae	*Rhamnus sphaerosperma*	KP299468	trnL
Rhizophoraceae	*Rhizophora mangle*	AF130332	ITS
Rhizophoraceae	*Rhizophora mangle*	HQ337954	ITS
Rhizophoraceae	*Rhizophora mangle*	HQ337955	ITS
Rhizophoraceae	*Rhizophora mangle*	HQ337956	ITS
Rhizophoraceae	*Rhizophora mangle*	HQ337957	ITS
Rhizophoraceae	*Rhizophora mangle*	HQ337958	ITS
Rhizophoraceae	*Rhizophora mangle*	KJ194264	ITS
Rhizophoraceae	*Rhizophora mangle*	KJ194265	ITS
Rhizophoraceae	*Rhizophora mangle*	KJ194266	ITS
Rhizophoraceae	*Rhizophora mangle*	KJ194267	ITS
Rhizophoraceae	*Rhizophora mangle*	KJ194268	ITS
Rhizophoraceae	*Rhizophora mangle*	KJ194269	ITS
Rhizophoraceae	*Rhizophora mangle*	KJ194270	ITS
Rhizophoraceae	*Rhizophora mangle*	JX661962	matK
Rhizophoraceae	*Rhizophora mangle*	KJ012752	matK
Rhizophoraceae	*Rhizophora mangle*	AB668356	rbcL
Rhizophoraceae	*Rhizophora mangle*	AB668357	rbcL
Rhizophoraceae	*Rhizophora mangle*	AB668358	rbcL
Rhizophoraceae	*Rhizophora mangle*	AB668359	rbcL
Rhizophoraceae	*Rhizophora mangle*	AF127688	rbcL
Rhizophoraceae	*Rhizophora mangle*	AF127689	rbcL

Family	Species	Accession	Barcode
Rhizophoraceae	*Rhizophora mangle*	JQ593598	rbcL
Rhizophoraceae	*Rhizophora mangle*	JQ593599	rbcL
Rhizophoraceae	*Rhizophora mangle*	JQ593600	rbcL
Rhizophoraceae	*Rhizophora mangle*	JX664070	rbcL
Rhizophoraceae	*Rhizophora mangle*	U26335.2	rbcL
Rhizophoraceae	*Rhizophora mangle*	JX663498	rpoB
Rhizophoraceae	*Rhizophora mangle*	JX664961	rpoC1
Rhizophoraceae	*Rhizophora mangle*	AF127753	trnL
Rhizophoraceae	*Rhizophora mangle*	AF127754	trnL
Rhizophoraceae	*Rhizophora mangle*	AY947404	trnL
Rhizophoraceae	*Rhizophora mangle*	JX664705	ycf1
Rosaceae	*Eriobotrya japonica*	AB636342	ITS
Rosaceae	*Eriobotrya japonica*	FJ449731-FJ449737	ITS
Rosaceae	*Eriobotrya japonica*	JQ392431	ITS
Rosaceae	*Eriobotrya japonica*	JQ392432	ITS
Rosaceae	*Eriobotrya japonica*	JQ392433	ITS
Rosaceae	*Eriobotrya japonica*	KC004046	ITS
Rosaceae	*Eriobotrya japonica*	KF022253	ITS
Rosaceae	*Eriobotrya japonica*	KJ170761-KJ170775	ITS
Rosaceae	*Eriobotrya japonica*	DQ860462	matK
Rosaceae	*Eriobotrya japonica*	GQ434196	matK
Rosaceae	*Eriobotrya japonica*	JQ391005	matK
Rosaceae	*Eriobotrya japonica*	JQ391006	matK
Rosaceae	*Eriobotrya japonica*	JX517887	matK
Rosaceae	*Eriobotrya japonica*	KJ170735-KJ170750	matK
Rosaceae	*Eriobotrya japonica*	KJ510949	matK
Rosaceae	*Eriobotrya japonica*	GQ305326	psbA-trnH
Rosaceae	*Eriobotrya japonica*	KF022254	psbA-trnH
Rosaceae	*Eriobotrya japonica*	KF022255	psbA-trnH
Rosaceae	*Eriobotrya japonica*	KJ139964-KJ139977	psbA-trnH
Rosaceae	*Eriobotrya japonica*	JQ391268	rbcL
Rosaceae	*Eriobotrya japonica*	JQ391271	rbcL
Rosaceae	*Eriobotrya japonica*	JQ391272	rbcL
Rosaceae	*Eriobotrya japonica*	JX572562	rbcL
Rosaceae	*Eriobotrya japonica*	KJ170709-KJ170724	rbcL

**Family**	**Species**	**Accession**	**Barcode**
Rosaceae	*Eriobotrya japonica*	KJ440049	rbcL
Rosaceae	*Eriobotrya japonica*	U06800	rbcL
Rosaceae	*Eriobotrya japonica*	JQ392275	trnL
Rosaceae	*Prunus myrtifolia*	JQ776841	ITS
Rosaceae	*Prunus myrtifolia*	JQ926611	ITS
Rosaceae	*Prunus myrtifolia*	KF420946	ITS
Rosaceae	*Prunus myrtifolia*	KF420947	ITS
Rosaceae	*Prunus myrtifolia*	HQ235202	matK
Rosaceae	*Prunus myrtifolia*	KF555443	matK
Rosaceae	*Prunus myrtifolia*	HQ188813	psbA-trnH
Rosaceae	*Prunus myrtifolia*	KF421064	psbA-trnH
Rosaceae	*Prunus myrtifolia*	KF421065	psbA-trnH
Rosaceae	*Prunus myrtifolia*	KF421066	psbA-trnH
Rosaceae	*Prunus myrtifolia*	KF421067	psbA-trnH
Rosaceae	*Prunus myrtifolia*	KJ426893	psbA-trnH
Rosaceae	*Prunus myrtifolia*	KJ426894	psbA-trnH
Rosaceae	*Prunus myrtifolia*	KJ426895	psbA-trnH
Rosaceae	*Prunus myrtifolia*	HQ235486	rbcL
Rosaceae	*Prunus myrtifolia*	KF561964	rbcL
Rosaceae	*Prunus myrtifolia*	KF561965	rbcL
Rosaceae	*Prunus myrtifolia*	KJ082518	rbcL
Rosaceae	*Prunus myrtifolia*	KJ082519	rbcL
Rosaceae	*Prunus myrtifolia*	HQ243834	trnL
Rosaceae	*Prunus myrtifolia*	HQ244071	trnL
Rubiaceae	*Alibertia edulis*	AF183740	ITS
Rubiaceae	*Alibertia edulis*	AF183741	ITS
Rubiaceae	*Alibertia edulis*	GQ981930	matK
Rubiaceae	*Alibertia edulis*	JQ588901	matK
Rubiaceae	*Alibertia edulis*	JQ588902	matK
Rubiaceae	*Alibertia edulis*	JQ588903	matK
Rubiaceae	*Alibertia edulis*	JQ589634	matK
Rubiaceae	*Alibertia edulis*	JQ589637	matK
Rubiaceae	*Alibertia edulis*	GQ982139	psbA-trnH
Rubiaceae	*Alibertia edulis*	GQ981657	rbcL
Rubiaceae	*Alibertia edulis*	JQ593605	rbcL

Family	Species	Accession	Barcode
Rubiaceae	*Alibertia edulis*	JQ593606	rbcL
Rubiaceae	*Alibertia edulis*	JQ593607	rbcL
Rubiaceae	*Alibertia edulis*	JQ594617	rbcL
Rubiaceae	*Alibertia edulis*	KF964808	rbcL
Rubiaceae	*Alibertia edulis*	Z68843	rbcL
Rubiaceae	*Alibertia edulis*	AF201029	trnL
Rubiaceae	*Alibertia edulis*	KF965155	trnL
Rubiaceae	*Alibertia edulis*	KF965156	trnL
Rubiaceae	*Alseis floribunda*	FJ984954	ITS
Rubiaceae	*Alseis floribunda*	FJ905330	matK
Rubiaceae	*Alseis floribunda*	FJ860148	psbA-trnH
Rubiaceae	*Alseis floribunda*	FJ948363	trnL
Rubiaceae	*Amaioua guianensis*	AF183774	ITS
Rubiaceae	*Amaioua guianensis*	AF183775	ITS
Rubiaceae	*Amaioua guianensis*	JQ626364	matK
Rubiaceae	*Amaioua guianensis*	GQ428646	psbA-trnH
Rubiaceae	*Amaioua guianensis*	GQ428647	psbA-trnH
Rubiaceae	*Amaioua guianensis*	AM117202	rbcL
Rubiaceae	*Amaioua guianensis*	GQ428615	rbcL
Rubiaceae	*Amaioua guianensis*	JQ625864	rbcL
Rubiaceae	*Amaioua guianensis*	AF201030	trnL
Rubiaceae	*Amaioua guianensis*	KF965157	trnL
Rubiaceae	*Bathysa australis*	FJ984957	ITS
Rubiaceae	*Bathysa gymnocarpa*	FJ984960	ITS
Rubiaceae	*Bathysa gymnocarpa*	FJ860153	psbA-trnH
Rubiaceae	*Bathysa gymnocarpa*	FJ948368	trnL
Rubiaceae	*Bathysa stipulata*	FJ984963	ITS
Rubiaceae	*Bathysa stipulata*	FJ905337	matK
Rubiaceae	*Bathysa stipulata*	FJ860156	psbA-trnH
Rubiaceae	*Bathysa stipulata*	HM164156	rbcL
Rubiaceae	*Bathysa stipulata*	FJ948371	trnL
Rubiaceae	*Coffea liberica*	AM412412	atpF-atpH
Rubiaceae	*Coffea liberica*	AF542985	ITS
Rubiaceae	*Coffea liberica*	AF542992	ITS
Rubiaceae	*Coffea liberica*	AF542999	ITS

Family	Species	Accession	Barcode
Rubiaceae	*Coffea liberica*	AF543000-AF543007	ITS
Rubiaceae	*Coffea liberica*	DQ153603	ITS
Rubiaceae	*Coffea liberica*	DQ153610	ITS
Rubiaceae	*Coffea liberica*	U64335	ITS
Rubiaceae	*Coffea liberica*	U64336	ITS
Rubiaceae	*Coffea liberica*	U64337	ITS
Rubiaceae	*Coffea liberica*	AM412465	matK
Rubiaceae	*Coffea liberica*	KC758274-KC758281	matK
Rubiaceae	*Coffea liberica*	KC758286-KC758293	rbcL
Rubiaceae	*Coffea liberica*	AF543018	trnL
Rubiaceae	*Coffea liberica*	AF543019	trnL
Rubiaceae	*Coffea liberica*	AF543041	trnL
Rubiaceae	*Coffea liberica*	AY555081	trnL
Rubiaceae	*Coffea liberica*	DQ153839	trnL
Rubiaceae	*Coffea liberica*	DQ153846	trnL
Rubiaceae	*Coffea liberica*	FJ493330	trnL
Rubiaceae	*Coffea liberica*	FJ493331	trnL
Rubiaceae	*Coffea liberica*	FJ493384	trnL
Rubiaceae	*Coffea liberica*	FJ493385	trnL
Rubiaceae	*Coffea liberica*	U93394	trnL
Rubiaceae	*Coffea liberica*	U93397	trnL
Rubiaceae	*Cordiera concolor*	AF183736	ITS
Rubiaceae	*Cordiera concolor*	AF183737	ITS
Rubiaceae	*Cordiera concolor*	AF183746	ITS
Rubiaceae	*Cordiera concolor*	AF183747	ITS
Rubiaceae	*Cordiera elliptica*	AY321602	ITS
Rubiaceae	*Cordiera macrophylla*	AF183752	ITS
Rubiaceae	*Cordiera macrophylla*	AF183753	ITS
Rubiaceae	*Cordiera myrciifolia*	AF183754	ITS
Rubiaceae	*Cordiera myrciifolia*	AF183755	ITS
Rubiaceae	*Cordiera sessilis*	AF183760	ITS
Rubiaceae	*Cordiera sessilis*	AF183761	ITS
Rubiaceae	*Coussarea hydrangeifolia*	EU145360	ITS
Rubiaceae	*Coussarea hydrangeifolia*	JQ729739	psbA-trnH
Rubiaceae	*Coussarea hydrangeifolia*	EU145460	rbcL

**Family**	**Species**	**Accession**	**Barcode**
Rubiaceae	*Coussarea hydrangeifolia*	EU145549	trnL
Rubiaceae	*Coutarea hexandra*	AY763890	ITS
Rubiaceae	*Coutarea hexandra*	Z95534	ITS
Rubiaceae	*Coutarea hexandra*	GQ981975	matK
Rubiaceae	*Coutarea hexandra*	JQ588930	matK
Rubiaceae	*Coutarea hexandra*	JQ588931	matK
Rubiaceae	*Coutarea hexandra*	JQ588932	matK
Rubiaceae	*Coutarea hexandra*	JQ588933	matK
Rubiaceae	*Coutarea hexandra*	JQ588934	matK
Rubiaceae	*Coutarea hexandra*	GQ982200	psbA-trnH
Rubiaceae	*Coutarea hexandra*	AM117221	rbcL
Rubiaceae	*Coutarea hexandra*	GQ981716	rbcL
Rubiaceae	*Coutarea hexandra*	JQ593649	rbcL
Rubiaceae	*Coutarea hexandra*	JQ593650	rbcL
Rubiaceae	*Coutarea hexandra*	JQ593651	rbcL
Rubiaceae	*Coutarea hexandra*	JQ593652	rbcL
Rubiaceae	*Coutarea hexandra*	JQ593653	rbcL
Rubiaceae	*Coutarea hexandra*	JQ593654	rbcL
Rubiaceae	*Coutarea hexandra*	JQ593655	rbcL
Rubiaceae	*Coutarea hexandra*	KF667925	rbcL
Rubiaceae	*Coutarea hexandra*	KF667930	rbcL
Rubiaceae	*Coutarea hexandra*	AF152699	trnL
Rubiaceae	*Coutarea hexandra*	AY763821	trnL
Rubiaceae	*Coutarea hexandra*	GQ852487	trnL
Rubiaceae	*Coutarea hexandra*	KJ906569	trnL
Rubiaceae	*Faramea hyacinthina*	KM215410	psbA-trnH
Rubiaceae	*Faramea multiflora*	EU145363	ITS
Rubiaceae	*Faramea multiflora*	JQ588942	matK
Rubiaceae	*Faramea multiflora*	JQ588943	matK
Rubiaceae	*Faramea multiflora*	JQ588944	matK
Rubiaceae	*Faramea multiflora*	FJ209071	rbcL
Rubiaceae	*Faramea multiflora*	JQ593665	rbcL
Rubiaceae	*Faramea multiflora*	JQ593666	rbcL
Rubiaceae	*Faramea multiflora*	JQ593667	rbcL
Rubiaceae	*Faramea multiflora*	Z68796	rbcL

**Family**	**Species**	**Accession**	**Barcode**
Rubiaceae	*Faramea multiflora*	AF102422	trnL
Rubiaceae	*Genipa americana*	AY538388	matK
Rubiaceae	*Genipa americana*	GQ982000	matK
Rubiaceae	*Genipa americana*	HM119538	matK
Rubiaceae	*Genipa americana*	HM446692	matK
Rubiaceae	*Genipa americana*	JQ588948	matK
Rubiaceae	*Genipa americana*	JQ588949	matK
Rubiaceae	*Genipa americana*	JQ588950	matK
Rubiaceae	*Genipa americana*	JQ588951	matK
Rubiaceae	*Genipa americana*	JQ588952	matK
Rubiaceae	*Genipa americana*	KJ136882	matK
Rubiaceae	*Genipa americana*	GQ982230	psbA-trnH
Rubiaceae	*Genipa americana*	HG963852	psbA-trnH
Rubiaceae	*Genipa americana*	HM446934	psbA-trnH
Rubiaceae	*Genipa americana*	GQ981747	rbcL
Rubiaceae	*Genipa americana*	HM446802	rbcL
Rubiaceae	*Genipa americana*	JQ593671	rbcL
Rubiaceae	*Genipa americana*	JQ593672	rbcL
Rubiaceae	*Genipa americana*	JQ593673	rbcL
Rubiaceae	*Genipa americana*	JQ593674	rbcL
Rubiaceae	*Genipa americana*	JQ593675	rbcL
Rubiaceae	*Genipa americana*	JQ593676	rbcL
Rubiaceae	*Genipa americana*	Z68839	rbcL
Rubiaceae	*Genipa americana*	AF201045	trnL
Rubiaceae	*Genipa americana*	HM164322	trnL
Rubiaceae	*Genipa americana*	KF965205	trnL
Rubiaceae	*Genipa americana*	KT218917	trnL
Rubiaceae	*Guettarda pohliana*	DQ063684	ITS
Rubiaceae	*Guettarda uruguensis*	AY730294	ITS
Rubiaceae	*Guettarda uruguensis*	DQ063692	ITS
Rubiaceae	*Guettarda uruguensis*	X83638	rbcL
Rubiaceae	*Guettarda uruguensis*	EU145533	trnL
Rubiaceae	*Hamelia patens*	GQ852135	ITS
Rubiaceae	*Hamelia patens*	JX468353	ITS
Rubiaceae	*Hamelia patens*	GQ982007	matK

**Family**	**Species**	**Accession**	**Barcode**
Rubiaceae	*Hamelia patens*	JQ588960	matK
Rubiaceae	*Hamelia patens*	JQ588961	matK
Rubiaceae	*Hamelia patens*	JQ588962	matK
Rubiaceae	*Hamelia patens*	JQ589616	matK
Rubiaceae	*Hamelia patens*	JQ589617	matK
Rubiaceae	*Hamelia patens*	JQ589618	matK
Rubiaceae	*Hamelia patens*	JQ589625	matK
Rubiaceae	*Hamelia patens*	JQ589725	matK
Rubiaceae	*Hamelia patens*	JQ589726	matK
Rubiaceae	*Hamelia patens*	JQ589727	matK
Rubiaceae	*Hamelia patens*	JQ589728	matK
Rubiaceae	*Hamelia patens*	KJ012631	matK
Rubiaceae	*Hamelia patens*	GQ982240	psbA-trnH
Rubiaceae	*Hamelia patens*	HG963617	psbA-trnH
Rubiaceae	*Hamelia patens*	KJ426770	psbA-trnH
Rubiaceae	*Hamelia patens*	GQ852333	rbcL
Rubiaceae	*Hamelia patens*	GQ981757	rbcL
Rubiaceae	*Hamelia patens*	JQ593683	rbcL
Rubiaceae	*Hamelia patens*	JQ593684	rbcL
Rubiaceae	*Hamelia patens*	JQ593685	rbcL
Rubiaceae	*Hamelia patens*	JQ593686	rbcL
Rubiaceae	*Hamelia patens*	JQ593687	rbcL
Rubiaceae	*Hamelia patens*	JQ593688	rbcL
Rubiaceae	*Hamelia patens*	JQ593689	rbcL
Rubiaceae	*Hamelia patens*	JQ593690	rbcL
Rubiaceae	*Hamelia patens*	JQ593691	rbcL
Rubiaceae	*Hamelia patens*	JQ593692	rbcL
Rubiaceae	*Hamelia patens*	JQ593693	rbcL
Rubiaceae	*Hamelia patens*	JQ594594	rbcL
Rubiaceae	*Hamelia patens*	JQ594595	rbcL
Rubiaceae	*Hamelia patens*	JQ594596	rbcL
Rubiaceae	*Hamelia patens*	JQ594605	rbcL
Rubiaceae	*Hamelia patens*	JQ594724	rbcL
Rubiaceae	*Hamelia patens*	JQ594725	rbcL
Rubiaceae	*Hamelia patens*	JQ594726	rbcL

Family	Species	Accession	Barcode
Rubiaceae	*Hamelia patens*	JQ594727	rbcL
Rubiaceae	*Hamelia patens*	JQ734510	rbcL
Rubiaceae	*Hamelia patens*	JQ734511	rbcL
Rubiaceae	*Hamelia patens*	KJ082355	rbcL
Rubiaceae	*Hamelia patens*	JX474907	trnL
Rubiaceae	*Hamelia patens*	KT218920	trnL
Rubiaceae	*Ixora brevifolia*	HG315453	ITS
Rubiaceae	*Ixora brevifolia*	HG315189	trnL
Rubiaceae	*Margaritopsis astrellantha*	AF149362	ITS
Rubiaceae	*Margaritopsis astrellantha*	JX155096	ITS
Rubiaceae	*Margaritopsis astrellantha*	JX155225	psbA-trnH
Rubiaceae	*Margaritopsis astrellantha*	JX155047	trnL
Rubiaceae	*Margaritopsis astrellantha*	JX155048	trnL
Rubiaceae	*Palicourea macrobotrys*	AF149335	ITS
Rubiaceae	*Palicourea rigida*	AF149342	ITS
Rubiaceae	*Posoqueria latifolia*	AJ224828	ITS
Rubiaceae	*Posoqueria latifolia*	DQ787409	ITS
Rubiaceae	*Posoqueria latifolia*	KC535862	ITS
Rubiaceae	*Posoqueria latifolia*	KM048212	ITS
Rubiaceae	*Posoqueria latifolia*	AY538412	matK
Rubiaceae	*Posoqueria latifolia*	FJ905325	matK
Rubiaceae	*Posoqueria latifolia*	GQ982066	matK
Rubiaceae	*Posoqueria latifolia*	JQ626556	matK
Rubiaceae	*Posoqueria latifolia*	GQ982323	psbA-trnH
Rubiaceae	*Posoqueria latifolia*	GQ428616	rbcL
Rubiaceae	*Posoqueria latifolia*	GQ981837	rbcL
Rubiaceae	*Posoqueria latifolia*	JQ626258	rbcL
Rubiaceae	*Posoqueria latifolia*	Z68850	rbcL
Rubiaceae	*Posoqueria latifolia*	AF152680	trnL
Rubiaceae	*Posoqueria latifolia*	FM207135	trnL
Rubiaceae	*Posoqueria latifolia*	KM048230	trnL
Rubiaceae	*Posoqueria latifolia*	KT218937	trnL
Rubiaceae	*Psychotria anceps*	AF149361	ITS
Rubiaceae	*Psychotria anceps*	JN053650	trnL
Rubiaceae	*Psychotria anceps*	JN643406	trnL

Family	Species	Accession	Barcode
Rubiaceae	*Psychotria capitata*	AF072005	ITS
Rubiaceae	*Psychotria capitata*	AF149375	ITS
Rubiaceae	*Psychotria carthagenensis*	KC480533	ITS
Rubiaceae	*Psychotria carthagenensis*	JN643433	trnL
Rubiaceae	*Psychotria cupularis*	JN643440	trnL
Rubiaceae	*Psychotria deflexa*	AF072006	ITS
Rubiaceae	*Psychotria deflexa*	AF149377	ITS
Rubiaceae	*Psychotria deflexa*	GQ982076	matK
Rubiaceae	*Psychotria deflexa*	HM446737	matK
Rubiaceae	*Psychotria deflexa*	GQ982339	psbA-trnH
Rubiaceae	*Psychotria deflexa*	HM446991	psbA-trnH
Rubiaceae	*Psychotria deflexa*	GQ981852	rbcL
Rubiaceae	*Psychotria deflexa*	HM446861	rbcL
Rubiaceae	*Psychotria hoffmannseggiana*	AF072035	ITS
Rubiaceae	*Psychotria hoffmannseggiana*	AF149365	ITS
Rubiaceae	*Psychotria hoffmannseggiana*	AF149387	ITS
Rubiaceae	*Psychotria hoffmannseggiana*	EF667970	ITS
Rubiaceae	*Psychotria hoffmannseggiana*	FJ980387	ITS
Rubiaceae	*Psychotria hoffmannseggiana*	GQ434644	ITS
Rubiaceae	*Psychotria hoffmannseggiana*	JN407051	psbA-trnH
Rubiaceae	*Psychotria hoffmannseggiana*	JN407052	psbA-trnH
Rubiaceae	*Psychotria hoffmannseggiana*	JN407053	psbA-trnH
Rubiaceae	*Psychotria hoffmannseggiana*	JN407054	psbA-trnH
Rubiaceae	*Psychotria hoffmannseggiana*	KJ687118	psbA-trnH
Rubiaceae	*Psychotria hoffmannseggiana*	KJ687119	psbA-trnH
Rubiaceae	*Psychotria hoffmannseggiana*	KJ687120	psbA-trnH
Rubiaceae	*Psychotria hoffmannseggiana*	KJ687121	psbA-trnH
Rubiaceae	*Psychotria hoffmannseggiana*	KJ687411	psbA-trnH
Rubiaceae	*Psychotria hoffmannseggiana*	KJ687412	psbA-trnH
Rubiaceae	*Psychotria hoffmannseggiana*	KJ687413	psbA-trnH
Rubiaceae	*Psychotria hoffmannseggiana*	KJ687414	psbA-trnH
Rubiaceae	*Psychotria hoffmannseggiana*	GQ436561	rbcL
Rubiaceae	*Psychotria hoffmannseggiana*	JN407371	rbcL
Rubiaceae	*Psychotria hoffmannseggiana*	JN407372	rbcL
Rubiaceae	*Psychotria hoffmannseggiana*	JN407373	rbcL

Family	Species	Accession	Barcode
Rubiaceae	*Psychotria hoffmannseggiana*	JN407374	rbcL
Rubiaceae	*Psychotria hoffmannseggiana*	KJ688510	rbcL
Rubiaceae	*Psychotria hoffmannseggiana*	KJ688511	rbcL
Rubiaceae	*Psychotria hoffmannseggiana*	KJ688512	rbcL
Rubiaceae	*Psychotria hoffmannseggiana*	KJ688513	rbcL
Rubiaceae	*Psychotria hoffmannseggiana*	KJ688825	rbcL
Rubiaceae	*Psychotria hoffmannseggiana*	KJ688826	rbcL
Rubiaceae	*Psychotria hoffmannseggiana*	KJ688827	rbcL
Rubiaceae	*Psychotria hoffmannseggiana*	KJ688828	rbcL
Rubiaceae	*Psychotria hoffmannseggiana*	JN643524	trnL
Rubiaceae	*Psychotria mapourioides*	AF072040	ITS
Rubiaceae	*Psychotria mapourioides*	AF149393	ITS
Rubiaceae	*Psychotria mapourioides*	KF675948	ITS
Rubiaceae	*Psychotria mapourioides*	FJ514725	matK
Rubiaceae	*Psychotria mapourioides*	JQ626482	matK
Rubiaceae	*Psychotria mapourioides*	KF676303	psbA-trnH
Rubiaceae	*Psychotria mapourioides*	FJ038151	rbcL
Rubiaceae	*Psychotria mapourioides*	JQ626079	rbcL
Rubiaceae	*Psychotria mapourioides*	FJ038364	rpoB
Rubiaceae	*Psychotria mapourioides*	FJ038767	rpoC1
Rubiaceae	*Psychotria mapourioides*	JN643498	trnL
Rubiaceae	*Psychotria mapourioides*	KF676212	trnL
Rubiaceae	*Psychotria nuda*	AF072053	ITS
Rubiaceae	*Randia armata*	AF493466-AF493469	ITS
Rubiaceae	*Randia armata*	AJ846856	ITS
Rubiaceae	*Randia armata*	JF977116-JF977127	ITS
Rubiaceae	*Randia armata*	KC339508	ITS
Rubiaceae	*Randia armata*	KM005489-KM005501	ITS
Rubiaceae	*Randia armata*	GQ982084	matK
Rubiaceae	*Randia armata*	JF954668-JF954680	matK
Rubiaceae	*Randia armata*	JQ589020-JQ589023	matK
Rubiaceae	*Randia armata*	JQ589559	matK
Rubiaceae	*Randia armata*	JQ589689	matK
Rubiaceae	*Randia armata*	KJ136894	matK
Rubiaceae	*Randia armata*	KM005394-KM005406	matK

Family	Species	Accession	Barcode
Rubiaceae	*Randia armata*	GQ982352	psbA-trnH
Rubiaceae	*Randia armata*	HG963812	psbA-trnH
Rubiaceae	*Randia armata*	JN045520-JN045532	psbA-trnH
Rubiaceae	*Randia armata*	KC339518	psbA-trnH
Rubiaceae	*Randia armata*	KM005580-KM005592	psbA-trnH
Rubiaceae	*Randia armata*	GQ981864	rbcL
Rubiaceae	*Randia armata*	JF942576-JF942588	rbcL
Rubiaceae	*Randia armata*	JQ593841-JQ593844	rbcL
Rubiaceae	*Randia armata*	JQ594679	rbcL
Rubiaceae	*Randia armata*	KM005301-KM005313	rbcL
Rubiaceae	*Randia armata*	AJ847370	trnL
Rubiaceae	*Randia armata*	AY555089	trnL
Rubiaceae	*Randia armata*	AY555093	trnL
Rubiaceae	*Randia armata*	KJ136960	trnL
Rubiaceae	*Randia armata*	KM384704	trnL
Rubiaceae	*Randia armata*	KM384705	trnL
Rubiaceae	*Randia armata*	KM384709	trnL
Rubiaceae	*Randia armata*	KM384710	trnL
Rubiaceae	*Randia armata*	KT218927	trnL
Rubiaceae	*Rudgea recurva*	AM117267	rbcL
Rubiaceae	*Rudgea viburnoides*	KJ804983	ITS
Rubiaceae	*Rudgea viburnoides*	KJ805779	rbcL
Rubiaceae	*Rustia formosa*	FJ905382	matK
Rubiaceae	*Rustia formosa*	FJ860202	psbA-trnH
Rubiaceae	*Rustia formosa*	FJ948414	trnL
Rubiaceae	*Schizocalyx cuspidatus*	FJ905334	matK
Rubiaceae	*Simira corumbensis*	FJ985009	ITS
Rubiaceae	*Simira corumbensis*	FJ905389	matK
Rubiaceae	*Simira corumbensis*	FJ860209	psbA-trnH
Rubiaceae	*Simira corumbensis*	FJ948420	trnL
Rubiaceae	*Simira viridiflora*	Y18718	rbcL
Rubiaceae	*Tocoyena formosa*	KF964891	rbcL
Rubiaceae	*Tocoyena formosa*	KF965272	trnL
Rutaceae	*Almeidea lilacina*	KP866622	ITS
Rutaceae	*Almeidea lilacina*	KP866645	ITS

Family	Species	Accession	Barcode
Rutaceae	*Almeidea lilacina*	KP866581	trnL
Rutaceae	*Balfourodendron riedelianum*	KC502921	ITS
Rutaceae	*Balfourodendron riedelianum*	FJ716747	matK
Rutaceae	*Balfourodendron riedelianum*	EU853779	trnL
Rutaceae	*Balfourodendron riedelianum*	FJ716791	trnL
Rutaceae	*Citrus reticulata*	GQ247928	atpF-atpH
Rutaceae	*Citrus reticulata*	AB456090	ITS
Rutaceae	*Citrus reticulata*	AB456091	ITS
Rutaceae	*Citrus reticulata*	AB456092	ITS
Rutaceae	*Citrus reticulata*	AB456115	ITS
Rutaceae	*Citrus reticulata*	AB571153	ITS
Rutaceae	*Citrus reticulata*	AM398230	ITS
Rutaceae	*Citrus reticulata*	EF590764	ITS
Rutaceae	*Citrus reticulata*	FJ641915-FJ641917	ITS
Rutaceae	*Citrus reticulata*	FJ641926	ITS
Rutaceae	*Citrus reticulata*	FJ641928-FJ641939	ITS
Rutaceae	*Citrus reticulata*	GQ225853	ITS
Rutaceae	*Citrus reticulata*	GQ231966	ITS
Rutaceae	*Citrus reticulata*	GQ231967	ITS
Rutaceae	*Citrus reticulata*	GQ434822	ITS
Rutaceae	*Citrus reticulata*	HQ893877	ITS
Rutaceae	*Citrus reticulata*	JN661209	ITS
Rutaceae	*Citrus reticulata*	AB626762	matK
Rutaceae	*Citrus reticulata*	AB626773	matK
Rutaceae	*Citrus reticulata*	FJ716729	matK
Rutaceae	*Citrus reticulata*	GQ248100	matK
Rutaceae	*Citrus reticulata*	HM163958	matK
Rutaceae	*Citrus reticulata*	JQ589068-JQ589071	matK
Rutaceae	*Citrus reticulata*	EF590680	psbA-trnH
Rutaceae	*Citrus reticulata*	GQ248268	psbA-trnH
Rutaceae	*Citrus reticulata*	GQ267062	psbA-trnH
Rutaceae	*Citrus reticulata*	GQ267063	psbA-trnH
Rutaceae	*Citrus reticulata*	GQ435438	psbA-trnH
Rutaceae	*Citrus reticulata*	GQ248434	psbK-psbI
Rutaceae	*Citrus reticulata*	KJ920728	psbK-psbI

Family	Species	Accession	Barcode
Rutaceae	*Citrus reticulata*	AB505952	rbcL
Rutaceae	*Citrus reticulata*	EF590512	rbcL
Rutaceae	*Citrus reticulata*	GQ248571	rbcL
Rutaceae	*Citrus reticulata*	GQ436736	rbcL
Rutaceae	*Citrus reticulata*	JQ593910-JQ593913	rbcL
Rutaceae	*Citrus reticulata*	GQ248747	rpoB
Rutaceae	*Citrus reticulata*	EF590602	rpoC1
Rutaceae	*Citrus reticulata*	GQ248908	rpoC1
Rutaceae	*Citrus reticulata*	GQ436252	rpoC1
Rutaceae	*Citrus reticulata*	AY115634	trnL
Rutaceae	*Citrus reticulata*	AY115657	trnL
Rutaceae	*Citrus reticulata*	AY116527	trnL
Rutaceae	*Citrus reticulata*	EF126670	trnL
Rutaceae	*Citrus reticulata*	EF126671	trnL
Rutaceae	*Citrus reticulata*	EU178123	trnL
Rutaceae	*Citrus reticulata*	EU369533-EU369576	trnL
Rutaceae	*Citrus reticulata*	FJ716773	trnL
Rutaceae	*Citrus reticulata*	HM163757	trnL
Rutaceae	*Citrus reticulata*	KJ667704	trnL
Rutaceae	*Citrus reticulata*	KJ667705	trnL
Rutaceae	*Citrus reticulata*	KJ667706	trnL
Rutaceae	*Citrus reticulata*	KJ667713	trnL
Rutaceae	*Citrus reticulata*	KJ667715	trnL
Rutaceae	*Citrus reticulata*	KJ667726	trnL
Rutaceae	*Citrus X limon*	AB456060	ITS
Rutaceae	*Citrus X limon*	AB456061	ITS
Rutaceae	*Citrus X limon*	AB456128	ITS
Rutaceae	*Citrus X limon*	FJ641963	ITS
Rutaceae	*Citrus X limon*	FJ980439	ITS
Rutaceae	*Citrus X limon*	GQ225862	ITS
Rutaceae	*Citrus X limon*	GQ434832	ITS
Rutaceae	*Citrus X limon*	HQ660683-HQ660688	ITS
Rutaceae	*Citrus X limon*	JQ990175	ITS
Rutaceae	*Citrus X limon*	AB626784	matK
Rutaceae	*Citrus X limon*	AB762352	matK

**Family**	**Species**	**Accession**	**Barcode**
Rutaceae	*Citrus X limon*	AB762353	matK
Rutaceae	*Citrus X limon*	JN315357	matK
Rutaceae	*Citrus X limon*	JN315359	matK
Rutaceae	*Citrus X limon*	KJ813750	matK
Rutaceae	*Citrus X limon*	GQ435450	psbA-trnH
Rutaceae	*Citrus X limon*	JN315362	psbA-trnH
Rutaceae	*Citrus X limon*	JN315363	psbA-trnH
Rutaceae	*Citrus X limon*	KJ920729	psbK-psbI
Rutaceae	*Citrus X limon*	AB505956	rbcL
Rutaceae	*Citrus X limon*	AY198378	rbcL
Rutaceae	*Citrus X limon*	GQ436744	rbcL
Rutaceae	*Citrus X limon*	AB817419	trnL
Rutaceae	*Citrus X limon*	AF025512	trnL
Rutaceae	*Citrus X limon*	AY115627	trnL
Rutaceae	*Citrus X limon*	AY115650	trnL
Rutaceae	*Citrus X limon*	AY116520	trnL
Rutaceae	*Citrus X limon*	EF126659	trnL
Rutaceae	*Citrus X limon*	EU178137	trnL
Rutaceae	*Citrus X limon*	EU369569	trnL
Rutaceae	*Citrus X limon*	JX390725	trnL
Rutaceae	*Citrus X limon*	KJ667682	trnL
Rutaceae	*Citrus X limon*	KJ667683	trnL
Rutaceae	*Citrus X limon*	KJ667684	trnL
Rutaceae	*Citrus X limon*	KJ667685	trnL
Rutaceae	*Citrus X limon*	KJ667714	trnL
Rutaceae	*Citrus X limon*	KJ667717	trnL
Rutaceae	*Citrus X limon*	KJ667722	trnL
Rutaceae	*Citrus X limon*	KJ667729	trnL
Rutaceae	*Conchocarpus pentandrus*	KP866633	ITS
Rutaceae	*Conchocarpus pentandrus*	KP866652	ITS
Rutaceae	*Dictyoloma vandellianum*	FR747983	psbA-trnH
Rutaceae	*Dictyoloma vandellianum*	FR747984	psbA-trnH
Rutaceae	*Dictyoloma vandellianum*	AF066823	rbcL
Rutaceae	*Dictyoloma vandellianum*	FR747845	rbcL
Rutaceae	*Dictyoloma vandellianum*	FR747846	rbcL

Family	Species	Accession	Barcode
Rutaceae	*Dictyoloma vandellianum*	AF025514	trnL
Rutaceae	*Dictyoloma vandellianum*	EU853793	trnL
Rutaceae	*Dictyoloma vandellianum*	FR747911	trnL
Rutaceae	*Dictyoloma vandellianum*	FR747912	trnL
Rutaceae	*Dictyoloma vandellianum*	KP055585	trnL
Rutaceae	*Esenbeckia febrifuga*	KP866634	ITS
Rutaceae	*Esenbeckia febrifuga*	KP866657	ITS
Rutaceae	*Esenbeckia febrifuga*	KP866594	trnL
Rutaceae	*Esenbeckia grandiflora*	KP866635	ITS
Rutaceae	*Esenbeckia grandiflora*	KP866656	ITS
Rutaceae	*Esenbeckia grandiflora*	EU853795	trnL
Rutaceae	*Galipea jasminiflora*	KP866636	ITS
Rutaceae	*Galipea jasminiflora*	KP866658	ITS
Rutaceae	*Galipea jasminiflora*	KP866595	trnL
Rutaceae	*Hortia brasiliana*	EU853800	trnL
Rutaceae	*Metrodorea nigra*	KC502928	ITS
Rutaceae	*Metrodorea nigra*	EU853809	trnL
Rutaceae	*Metrodorea stipularis*	KC502930	ITS
Rutaceae	*Murraya paniculata*	AB456044	ITS
Rutaceae	*Murraya paniculata*	AJ879085	ITS
Rutaceae	*Murraya paniculata*	FJ434153	ITS
Rutaceae	*Murraya paniculata*	FJ641970	ITS
Rutaceae	*Murraya paniculata*	FJ980438	ITS
Rutaceae	*Murraya paniculata*	FN293002	ITS
Rutaceae	*Murraya paniculata*	FN293003	ITS
Rutaceae	*Murraya paniculata*	FN293004	ITS
Rutaceae	*Murraya paniculata*	FN293005	ITS
Rutaceae	*Murraya paniculata*	FN293006	ITS
Rutaceae	*Murraya paniculata*	GQ434828	ITS
Rutaceae	*Murraya paniculata*	GQ434829	ITS
Rutaceae	*Murraya paniculata*	GQ434830	ITS
Rutaceae	*Murraya paniculata*	GQ434831	ITS
Rutaceae	*Murraya paniculata*	JX144211	ITS
Rutaceae	*Murraya paniculata*	JX144218	ITS
Rutaceae	*Murraya paniculata*	JX144219	ITS

Family	Species	Accession	Barcode
Rutaceae	*Murraya paniculata*	KM514676	ITS
Rutaceae	*Murraya paniculata*	KM887368	ITS
Rutaceae	*Murraya paniculata*	KP093213	ITS
Rutaceae	*Murraya paniculata*	KP093214	ITS
Rutaceae	*Murraya paniculata*	AB762389	matK
Rutaceae	*Murraya paniculata*	EF138906	matK
Rutaceae	*Murraya paniculata*	GQ434284	matK
Rutaceae	*Murraya paniculata*	GQ434285	matK
Rutaceae	*Murraya paniculata*	GU135010	matK
Rutaceae	*Murraya paniculata*	KP093688	matK
Rutaceae	*Murraya paniculata*	KP093689	matK
Rutaceae	*Murraya paniculata*	GQ435447	psbA-trnH
Rutaceae	*Murraya paniculata*	GQ435448	psbA-trnH
Rutaceae	*Murraya paniculata*	GQ435449	psbA-trnH
Rutaceae	*Murraya paniculata*	GU135341	psbA-trnH
Rutaceae	*Murraya paniculata*	KP095944	psbA-trnH
Rutaceae	*Murraya paniculata*	KP095945	psbA-trnH
Rutaceae	*Murraya paniculata*	AB505906	rbcL
Rutaceae	*Murraya paniculata*	AF320868	rbcL
Rutaceae	*Murraya paniculata*	AF320870	rbcL
Rutaceae	*Murraya paniculata*	GQ436741	rbcL
Rutaceae	*Murraya paniculata*	GQ436742	rbcL
Rutaceae	*Murraya paniculata*	GQ436743	rbcL
Rutaceae	*Murraya paniculata*	GU135173	rbcL
Rutaceae	*Murraya paniculata*	KF496528	rbcL
Rutaceae	*Murraya paniculata*	KJ667644	rbcL
Rutaceae	*Murraya paniculata*	KP094625	rbcL
Rutaceae	*Murraya paniculata*	KP094626	rbcL
Rutaceae	*Murraya paniculata*	U38860	rbcL
Rutaceae	*Murraya paniculata*	GQ436258	rpoC1
Rutaceae	*Murraya paniculata*	GQ436259	rpoC1
Rutaceae	*Murraya paniculata*	KJ749906	rpoC1
Rutaceae	*Murraya paniculata*	AB985680	trnL
Rutaceae	*Murraya paniculata*	AF025521	trnL
Rutaceae	*Murraya paniculata*	AY115632	trnL

**Family**	**Species**	**Accession**	**Barcode**
Rutaceae	*Murraya paniculata*	AY115655	trnL
Rutaceae	*Murraya paniculata*	AY116525	trnL
Rutaceae	*Murraya paniculata*	AY295280	trnL
Rutaceae	*Murraya paniculata*	EU853810	trnL
Rutaceae	*Murraya paniculata*	JF804918	trnL
Rutaceae	*Murraya paniculata*	JX144256	trnL
Rutaceae	*Murraya paniculata*	JX144263	trnL
Rutaceae	*Murraya paniculata*	JX144264	trnL
Rutaceae	*Murraya paniculata*	JX144265	trnL
Rutaceae	*Murraya paniculata*	KJ641529	trnL
Rutaceae	*Pilocarpus pennatifolius*	AF066809	rbcL
Rutaceae	*Pilocarpus spicatus*	KP866639	ITS
Rutaceae	*Pilocarpus spicatus*	KP866661	ITS
Rutaceae	*Pilocarpus spicatus*	EU853811	trnL
Rutaceae	*Pilocarpus spicatus*	KP866597	trnL
Rutaceae	*Zanthoxylum acuminatum*	HM851472	ITS
Rutaceae	*Zanthoxylum acuminatum*	JQ589091	matK
Rutaceae	*Zanthoxylum acuminatum*	JQ589550	matK
Rutaceae	*Zanthoxylum acuminatum*	GQ982408	psbA-trnH
Rutaceae	*Zanthoxylum acuminatum*	GQ982409	psbA-trnH
Rutaceae	*Zanthoxylum acuminatum*	GQ981921	rbcL
Rutaceae	*Zanthoxylum acuminatum*	JQ594535	rbcL
Rutaceae	*Zanthoxylum acuminatum*	HM851508	trnL
Rutaceae	*Zanthoxylum caribaeum*	KJ012825	matK
Rutaceae	*Zanthoxylum caribaeum*	KJ426988	psbA-trnH
Rutaceae	*Zanthoxylum caribaeum*	JQ593944	rbcL
Rutaceae	*Zanthoxylum caribaeum*	JQ593945	rbcL
Rutaceae	*Zanthoxylum caribaeum*	KJ082644	rbcL
Rutaceae	*Zanthoxylum fagara*	HM851473	ITS
Rutaceae	*Zanthoxylum fagara*	JQ589092	matK
Rutaceae	*Zanthoxylum fagara*	JQ589093	matK
Rutaceae	*Zanthoxylum fagara*	JQ589094	matK
Rutaceae	*Zanthoxylum fagara*	KJ773241	matK
Rutaceae	*Zanthoxylum fagara*	KM219823	matK
Rutaceae	*Zanthoxylum fagara*	HG963931	psbA-trnH

Family	Species	Accession	Barcode
Rutaceae	*Zanthoxylum fagara*	JQ593946	rbcL
Rutaceae	*Zanthoxylum fagara*	JQ593947	rbcL
Rutaceae	*Zanthoxylum fagara*	JQ593948	rbcL
Rutaceae	*Zanthoxylum fagara*	JQ593949	rbcL
Rutaceae	*Zanthoxylum fagara*	KJ773993	rbcL
Rutaceae	*Zanthoxylum fagara*	HM851509	trnL
Rutaceae	*Zanthoxylum rhoifolium*	HM851477	ITS
Rutaceae	*Zanthoxylum rhoifolium*	HM851478	ITS
Rutaceae	*Zanthoxylum rhoifolium*	KC502933	ITS
Rutaceae	*Zanthoxylum rhoifolium*	KF420934	ITS
Rutaceae	*Zanthoxylum rhoifolium*	KP866642	ITS
Rutaceae	*Zanthoxylum rhoifolium*	KP866663	ITS
Rutaceae	*Zanthoxylum rhoifolium*	KF555448	matK
Rutaceae	*Zanthoxylum rhoifolium*	KF421083	psbA-trnH
Rutaceae	*Zanthoxylum rhoifolium*	KF561971	rbcL
Rutaceae	*Zanthoxylum rhoifolium*	EU853773	trnL
Rutaceae	*Zanthoxylum rhoifolium*	HM851513	trnL
Rutaceae	*Zanthoxylum rhoifolium*	HM851514	trnL
Rutaceae	*Zanthoxylum riedelianum*	HM851491	ITS
Rutaceae	*Zanthoxylum riedelianum*	HM851527	trnL
Rutaceae	*Zanthoxylum tingoassuiba*	HM851469	ITS
Rutaceae	*Zanthoxylum tingoassuiba*	JX393913	ITS
Rutaceae	*Zanthoxylum tingoassuiba*	JX393915	ITS
Rutaceae	*Zanthoxylum tingoassuiba*	JX393916	ITS
Rutaceae	*Zanthoxylum tingoassuiba*	JX393918	ITS
Rutaceae	*Zanthoxylum tingoassuiba*	JX393919	ITS
Rutaceae	*Zanthoxylum tingoassuiba*	JX393922	ITS
Rutaceae	*Zanthoxylum tingoassuiba*	JX393923	ITS
Rutaceae	*Zanthoxylum tingoassuiba*	JX393924	ITS
Rutaceae	*Zanthoxylum tingoassuiba*	JX393926	ITS
Rutaceae	*Zanthoxylum tingoassuiba*	JX393928	ITS
Rutaceae	*Zanthoxylum tingoassuiba*	JX393929	ITS
Rutaceae	*Zanthoxylum tingoassuiba*	JX393930	ITS
Rutaceae	*Zanthoxylum tingoassuiba*	JX393933	ITS
Rutaceae	*Zanthoxylum tingoassuiba*	JX393934	ITS

**Family**	**Species**	**Accession**	**Barcode**
Rutaceae	*Zanthoxylum tingoassuiba*	JX393935	ITS
Rutaceae	*Zanthoxylum tingoassuiba*	KF240663	ITS
Rutaceae	*Zanthoxylum tingoassuiba*	KF240664	ITS
Rutaceae	*Zanthoxylum tingoassuiba*	KF240665	ITS
Rutaceae	*Zanthoxylum tingoassuiba*	KF240667	ITS
Rutaceae	*Zanthoxylum tingoassuiba*	KF240668	ITS
Rutaceae	*Zanthoxylum tingoassuiba*	KF240669	ITS
Rutaceae	*Zanthoxylum tingoassuiba*	KF240670	ITS
Rutaceae	*Zanthoxylum tingoassuiba*	KF240671	ITS
Rutaceae	*Zanthoxylum tingoassuiba*	KF240672	ITS
Rutaceae	*Zanthoxylum tingoassuiba*	KF240675	ITS
Rutaceae	*Zanthoxylum tingoassuiba*	KF240676	ITS
Rutaceae	*Zanthoxylum tingoassuiba*	KF240678	ITS
Rutaceae	*Zanthoxylum tingoassuiba*	KF240679	ITS
Rutaceae	*Zanthoxylum tingoassuiba*	KF240681	ITS
Rutaceae	*Zanthoxylum tingoassuiba*	KF240682	ITS
Rutaceae	*Zanthoxylum tingoassuiba*	KP093221	ITS
Rutaceae	*Zanthoxylum tingoassuiba*	KP093222	ITS
Rutaceae	*Zanthoxylum tingoassuiba*	AB925028	matK
Rutaceae	*Zanthoxylum tingoassuiba*	KP093339	matK
Rutaceae	*Zanthoxylum tingoassuiba*	KP093340	matK
Rutaceae	*Zanthoxylum tingoassuiba*	KF240691	psbA-trnH
Rutaceae	*Zanthoxylum tingoassuiba*	KF240695	psbA-trnH
Rutaceae	*Zanthoxylum tingoassuiba*	KF240696	psbA-trnH
Rutaceae	*Zanthoxylum tingoassuiba*	KF240697	psbA-trnH
Rutaceae	*Zanthoxylum tingoassuiba*	KF240698	psbA-trnH
Rutaceae	*Zanthoxylum tingoassuiba*	KF240700	psbA-trnH
Rutaceae	*Zanthoxylum tingoassuiba*	KF240701	psbA-trnH
Rutaceae	*Zanthoxylum tingoassuiba*	KF240703	psbA-trnH
Rutaceae	*Zanthoxylum tingoassuiba*	KF240704	psbA-trnH
Rutaceae	*Zanthoxylum tingoassuiba*	KF240705	psbA-trnH
Rutaceae	*Zanthoxylum tingoassuiba*	KF240706	psbA-trnH
Rutaceae	*Zanthoxylum tingoassuiba*	KF240707	psbA-trnH
Rutaceae	*Zanthoxylum tingoassuiba*	KF240708	psbA-trnH
Rutaceae	*Zanthoxylum tingoassuiba*	KF240709	psbA-trnH

**Family**	**Species**	**Accession**	**Barcode**
Rutaceae	*Zanthoxylum tingoassuiba*	KF240710	psbA-trnH
Rutaceae	*Zanthoxylum tingoassuiba*	KP095952	psbA-trnH
Rutaceae	*Zanthoxylum tingoassuiba*	KP095953	psbA-trnH
Rutaceae	*Zanthoxylum tingoassuiba*	AB925656	rbcL
Rutaceae	*Zanthoxylum tingoassuiba*	FN599471	rbcL
Rutaceae	*Zanthoxylum tingoassuiba*	KF240621	rbcL
Rutaceae	*Zanthoxylum tingoassuiba*	KF240626	rbcL
Rutaceae	*Zanthoxylum tingoassuiba*	KF240627	rbcL
Rutaceae	*Zanthoxylum tingoassuiba*	KF240628	rbcL
Rutaceae	*Zanthoxylum tingoassuiba*	KF240629	rbcL
Rutaceae	*Zanthoxylum tingoassuiba*	KF240632	rbcL
Rutaceae	*Zanthoxylum tingoassuiba*	KF240633	rbcL
Rutaceae	*Zanthoxylum tingoassuiba*	KF240634	rbcL
Rutaceae	*Zanthoxylum tingoassuiba*	KF240635	rbcL
Rutaceae	*Zanthoxylum tingoassuiba*	KF240636	rbcL
Rutaceae	*Zanthoxylum tingoassuiba*	KF240637	rbcL
Rutaceae	*Zanthoxylum tingoassuiba*	KF240638	rbcL
Rutaceae	*Zanthoxylum tingoassuiba*	KF240647	rbcL
Rutaceae	*Zanthoxylum tingoassuiba*	KF240648	rbcL
Rutaceae	*Zanthoxylum tingoassuiba*	KF240649	rbcL
Rutaceae	*Zanthoxylum tingoassuiba*	KP094251	rbcL
Rutaceae	*Zanthoxylum tingoassuiba*	KP094252	rbcL
Rutaceae	*Zanthoxylum tingoassuiba*	GQ436260	rpoC1
Rutaceae	*Zanthoxylum tingoassuiba*	DQ225915	trnL
Rutaceae	*Zanthoxylum tingoassuiba*	DQ226009	trnL
Rutaceae	*Zanthoxylum tingoassuiba*	FN599490	trnL
Rutaceae	*Zanthoxylum tingoassuiba*	HM851505	trnL
Sabiaceae	*Meliosma chartacea*	KP900365	ITS
Sabiaceae	*Meliosma chartacea*	KP900556	psbA-trnH
Salicaceae	*Banara tomentosa*	AY756940	trnL
Salicaceae	*Banara tomentosa*	AY756998	trnL
Salicaceae	*Casearia aculeata*	GQ981950	matK
Salicaceae	*Casearia aculeata*	GQ982166	psbA-trnH
Salicaceae	*Casearia aculeata*	GQ981685	rbcL
Salicaceae	*Casearia arborea*	GQ981951	matK

Family	Species	Accession	Barcode
Salicaceae	*Casearia arborea*	HM446663	matK
Salicaceae	*Casearia arborea*	JQ587935	matK
Salicaceae	*Casearia arborea*	JQ589106	matK
Salicaceae	*Casearia arborea*	JQ589107	matK
Salicaceae	*Casearia arborea*	JQ589108	matK
Salicaceae	*Casearia arborea*	GQ982167	psbA-trnH
Salicaceae	*Casearia arborea*	HM446896	psbA-trnH
Salicaceae	*Casearia arborea*	GQ981686	rbcL
Salicaceae	*Casearia arborea*	JQ593966	rbcL
Salicaceae	*Casearia arborea*	JQ593967	rbcL
Salicaceae	*Casearia decandra*	EF135516	matK
Salicaceae	*Casearia decandra*	JQ589111	matK
Salicaceae	*Casearia decandra*	JQ589112	matK
Salicaceae	*Casearia decandra*	JX661934	matK
Salicaceae	*Casearia decandra*	KJ426647	psbA-trnH
Salicaceae	*Casearia decandra*	JQ593971	rbcL
Salicaceae	*Casearia decandra*	JQ593972	rbcL
Salicaceae	*Casearia decandra*	JQ593973	rbcL
Salicaceae	*Casearia decandra*	JQ593974	rbcL
Salicaceae	*Casearia decandra*	JQ593975	rbcL
Salicaceae	*Casearia decandra*	JQ593976	rbcL
Salicaceae	*Casearia decandra*	JQ593977	rbcL
Salicaceae	*Casearia decandra*	JQ593978	rbcL
Salicaceae	*Casearia decandra*	JQ593979	rbcL
Salicaceae	*Casearia decandra*	JQ593980	rbcL
Salicaceae	*Casearia decandra*	JQ626222	rbcL
Salicaceae	*Casearia decandra*	JX664038	rbcL
Salicaceae	*Casearia decandra*	KJ082173	rbcL
Salicaceae	*Casearia decandra*	JX663466	rpoB
Salicaceae	*Casearia decandra*	JX664927	rpoC1
Salicaceae	*Casearia decandra*	AY756895	trnL
Salicaceae	*Casearia decandra*	AY757010	trnL
Salicaceae	*Casearia decandra*	JX664686	ycf1
Salicaceae	*Casearia grandiflora*	AY756894	trnL
Salicaceae	*Casearia grandiflora*	AY757009	trnL

**Family**	**Species**	**Accession**	**Barcode**
Salicaceae	*Casearia sylvestris*	GQ981954	matK
Salicaceae	*Casearia sylvestris*	HM446664	matK
Salicaceae	*Casearia sylvestris*	JQ589114	matK
Salicaceae	*Casearia sylvestris*	JQ589115	matK
Salicaceae	*Casearia sylvestris*	JQ589116	matK
Salicaceae	*Casearia sylvestris*	JQ589639	matK
Salicaceae	*Casearia sylvestris*	GQ982170	psbA-trnH
Salicaceae	*Casearia sylvestris*	HM446898	psbA-trnH
Salicaceae	*Casearia sylvestris*	AF206746	rbcL
Salicaceae	*Casearia sylvestris*	GQ428609	rbcL
Salicaceae	*Casearia sylvestris*	GQ428610	rbcL
Salicaceae	*Casearia sylvestris*	GQ981689	rbcL
Salicaceae	*Casearia sylvestris*	HM446768	rbcL
Salicaceae	*Casearia sylvestris*	JQ592122	rbcL
Salicaceae	*Casearia sylvestris*	JQ592123	rbcL
Salicaceae	*Casearia sylvestris*	JQ592124	rbcL
Salicaceae	*Casearia sylvestris*	JQ592125	rbcL
Salicaceae	*Casearia sylvestris*	JQ593984	rbcL
Salicaceae	*Casearia sylvestris*	JQ593985	rbcL
Salicaceae	*Casearia sylvestris*	JQ593986	rbcL
Salicaceae	*Casearia sylvestris*	JQ594619	rbcL
Salicaceae	*Casearia sylvestris*	JQ625967	rbcL
Salicaceae	*Casearia sylvestris*	AY756897	trnL
Salicaceae	*Casearia sylvestris*	AY757012	trnL
Salicaceae	*Prockia crucis*	EF135588	matK
Salicaceae	*Prockia crucis*	AJ418831	rbcL
Salicaceae	*Prockia crucis*	JQ592131	rbcL
Salicaceae	*Prockia crucis*	JQ592132	rbcL
Salicaceae	*Prockia crucis*	JQ592133	rbcL
Salicaceae	*Prockia crucis*	AY756961	trnL
Salicaceae	*Prockia crucis*	AY757057	trnL
Salicaceae	*Salix humboldtiana*	EF060372	ITS
Salicaceae	*Salix humboldtiana*	KC415516	ITS
Salicaceae	*Salix humboldtiana*	EU790679	matK
Salicaceae	*Xylosma venosa*	AY756972	trnL

**Family**	**Species**	**Accession**	**Barcode**
Salicaceae	*Xylosma venosa*	AY757082	trnL
Sapindaceae	*Allophylus edulis*	EU853777	trnL
Sapindaceae	*Allophylus racemosus*	JQ589127	matK
Sapindaceae	*Allophylus racemosus*	JQ589128	matK
Sapindaceae	*Allophylus racemosus*	JQ589129	matK
Sapindaceae	*Allophylus racemosus*	KJ012459	matK
Sapindaceae	*Allophylus racemosus*	KJ426599	psbA-trnH
Sapindaceae	*Allophylus racemosus*	JQ593996	rbcL
Sapindaceae	*Allophylus racemosus*	JQ593997	rbcL
Sapindaceae	*Allophylus racemosus*	JQ593998	rbcL
Sapindaceae	*Allophylus racemosus*	KJ082114	rbcL
Sapindaceae	*Cupania vernalis*	KF421002	ITS
Sapindaceae	*Cupania vernalis*	KF421003	ITS
Sapindaceae	*Cupania vernalis*	KF421004	ITS
Sapindaceae	*Cupania vernalis*	KF421005	ITS
Sapindaceae	*Cupania vernalis*	KF555390	matK
Sapindaceae	*Cupania vernalis*	KF555391	matK
Sapindaceae	*Cupania vernalis*	KF555392	matK
Sapindaceae	*Cupania vernalis*	KF555393	matK
Sapindaceae	*Cupania vernalis*	KF421084	psbA-trnH
Sapindaceae	*Cupania vernalis*	KF421085	psbA-trnH
Sapindaceae	*Cupania vernalis*	KF421086	psbA-trnH
Sapindaceae	*Cupania vernalis*	KF421087	psbA-trnH
Sapindaceae	*Cupania vernalis*	KF421088	psbA-trnH
Sapindaceae	*Cupania vernalis*	KF561910	rbcL
Sapindaceae	*Cupania vernalis*	KF561911	rbcL
Sapindaceae	*Cupania vernalis*	EU853791	trnL
Sapindaceae	*Diatenopteryx sorbifolia*	EU720534	ITS
Sapindaceae	*Diatenopteryx sorbifolia*	EU720682	matK
Sapindaceae	*Diatenopteryx sorbifolia*	EU720810	rpoB
Sapindaceae	*Diatenopteryx sorbifolia*	EU720882	rpoB
Sapindaceae	*Diatenopteryx sorbifolia*	EU721246	trnL
Sapindaceae	*Diatenopteryx sorbifolia*	EU721303	trnL
Sapindaceae	*Diatenopteryx sorbifolia*	EU721434	trnL
Sapindaceae	*Diatenopteryx sorbifolia*	EU721491	trnL

**Family**	**Species**	**Accession**	**Barcode**
Sapindaceae	*Dilodendron bipinnatum*	EU720677	matK
Sapindaceae	*Dilodendron bipinnatum*	GU935449	rbcL
Sapindaceae	*Dilodendron bipinnatum*	EU720874	rpoB
Sapindaceae	*Dilodendron bipinnatum*	AY594510	trnL
Sapindaceae	*Dilodendron bipinnatum*	EU721296	trnL
Sapindaceae	*Dilodendron bipinnatum*	EU721484	trnL
Sapindaceae	*Dodonaea viscosa*	AY864896	ITS
Sapindaceae	*Dodonaea viscosa*	DQ499142	ITS
Sapindaceae	*Dodonaea viscosa*	DQ499143	ITS
Sapindaceae	*Dodonaea viscosa*	EU720406	ITS
Sapindaceae	*Dodonaea viscosa*	EU720519	ITS
Sapindaceae	*Dodonaea viscosa*	EU720536	ITS
Sapindaceae	*Dodonaea viscosa*	FJ375187	ITS
Sapindaceae	*Dodonaea viscosa*	FJ546933	ITS
Sapindaceae	*Dodonaea viscosa*	FJ546934	ITS
Sapindaceae	*Dodonaea viscosa*	FJ546935	ITS
Sapindaceae	*Dodonaea viscosa*	FJ546936	ITS
Sapindaceae	*Dodonaea viscosa*	FJ546937	ITS
Sapindaceae	*Dodonaea viscosa*	FJ546938	ITS
Sapindaceae	*Dodonaea viscosa*	FJ546939	ITS
Sapindaceae	*Dodonaea viscosa*	FJ546940	ITS
Sapindaceae	*Dodonaea viscosa*	FJ546941	ITS
Sapindaceae	*Dodonaea viscosa*	FJ546942	ITS
Sapindaceae	*Dodonaea viscosa*	FJ546943	ITS
Sapindaceae	*Dodonaea viscosa*	FJ546944	ITS
Sapindaceae	*Dodonaea viscosa*	FJ546945	ITS
Sapindaceae	*Dodonaea viscosa*	FJ546946	ITS
Sapindaceae	*Dodonaea viscosa*	FJ546947	ITS
Sapindaceae	*Dodonaea viscosa*	FJ546948	ITS
Sapindaceae	*Dodonaea viscosa*	FJ546949	ITS
Sapindaceae	*Dodonaea viscosa*	FJ546950	ITS
Sapindaceae	*Dodonaea viscosa*	FJ546951	ITS
Sapindaceae	*Dodonaea viscosa*	FJ546952	ITS
Sapindaceae	*Dodonaea viscosa*	FJ546953	ITS
Sapindaceae	*Dodonaea viscosa*	FJ546954	ITS

**Family**	**Species**	**Accession**	**Barcode**
Sapindaceae	*Dodonaea viscosa*	FJ546955	ITS
Sapindaceae	*Dodonaea viscosa*	FJ546956	ITS
Sapindaceae	*Dodonaea viscosa*	FJ546957	ITS
Sapindaceae	*Dodonaea viscosa*	FJ546958	ITS
Sapindaceae	*Dodonaea viscosa*	FJ546959	ITS
Sapindaceae	*Dodonaea viscosa*	FJ546960	ITS
Sapindaceae	*Dodonaea viscosa*	FJ546961	ITS
Sapindaceae	*Dodonaea viscosa*	FJ546962	ITS
Sapindaceae	*Dodonaea viscosa*	FJ546963	ITS
Sapindaceae	*Dodonaea viscosa*	FJ546964	ITS
Sapindaceae	*Dodonaea viscosa*	FJ546965	ITS
Sapindaceae	*Dodonaea viscosa*	FJ546966	ITS
Sapindaceae	*Dodonaea viscosa*	FJ546967	ITS
Sapindaceae	*Dodonaea viscosa*	FJ546968	ITS
Sapindaceae	*Dodonaea viscosa*	FJ546969	ITS
Sapindaceae	*Dodonaea viscosa*	FJ546970	ITS
Sapindaceae	*Dodonaea viscosa*	FJ546971	ITS
Sapindaceae	*Dodonaea viscosa*	FJ546972	ITS
Sapindaceae	*Dodonaea viscosa*	FJ546973	ITS
Sapindaceae	*Dodonaea viscosa*	FJ546974	ITS
Sapindaceae	*Dodonaea viscosa*	FJ546975	ITS
Sapindaceae	*Dodonaea viscosa*	HE586149	ITS
Sapindaceae	*Dodonaea viscosa*	HE586150	ITS
Sapindaceae	*Dodonaea viscosa*	HE586151	ITS
Sapindaceae	*Dodonaea viscosa*	JN190991	ITS
Sapindaceae	*Dodonaea viscosa*	JN681314	ITS
Sapindaceae	*Dodonaea viscosa*	AF314803	matK
Sapindaceae	*Dodonaea viscosa*	EU720567	matK
Sapindaceae	*Dodonaea viscosa*	EU720666	matK
Sapindaceae	*Dodonaea viscosa*	EU720684	matK
Sapindaceae	*Dodonaea viscosa*	JN191118	matK
Sapindaceae	*Dodonaea viscosa*	JX495703	matK
Sapindaceae	*Dodonaea viscosa*	JX517889	matK
Sapindaceae	*Dodonaea viscosa*	KJ012569	matK
Sapindaceae	*Dodonaea viscosa*	KJ426704	psbA-trnH

Family	Species	Accession	Barcode
Sapindaceae	*Dodonaea viscosa*	DQ978445	rbcL
Sapindaceae	*Dodonaea viscosa*	FN599447	rbcL
Sapindaceae	*Dodonaea viscosa*	GU935451	rbcL
Sapindaceae	*Dodonaea viscosa*	JX571819	rbcL
Sapindaceae	*Dodonaea viscosa*	JX572528	rbcL
Sapindaceae	*Dodonaea viscosa*	KF432049	rbcL
Sapindaceae	*Dodonaea viscosa*	KJ082273	rbcL
Sapindaceae	*Dodonaea viscosa*	EU720721	rpoB
Sapindaceae	*Dodonaea viscosa*	EU720863	rpoB
Sapindaceae	*Dodonaea viscosa*	EU720884	rpoB
Sapindaceae	*Dodonaea viscosa*	AB817434	trnL
Sapindaceae	*Dodonaea viscosa*	AY594513	trnL
Sapindaceae	*Dodonaea viscosa*	DQ978578	trnL
Sapindaceae	*Dodonaea viscosa*	EU721158	trnL
Sapindaceae	*Dodonaea viscosa*	EU721285	trnL
Sapindaceae	*Dodonaea viscosa*	EU721305	trnL
Sapindaceae	*Dodonaea viscosa*	EU721347	trnL
Sapindaceae	*Dodonaea viscosa*	EU721473	trnL
Sapindaceae	*Dodonaea viscosa*	EU721493	trnL
Sapindaceae	*Dodonaea viscosa*	FN599503	trnL
Sapindaceae	*Dodonaea viscosa*	JF804897	trnL
Sapindaceae	*Dodonaea viscosa*	JN191034	trnL
Sapindaceae	*Dodonaea viscosa*	JN191076	trnL
Sapindaceae	*Dodonaea viscosa*	JN681487	trnL
Sapindaceae	*Dodonaea viscosa*	JN681553	trnL
Sapindaceae	*Dodonaea viscosa*	KP398502	trnL
Sapindaceae	*Magonia pubescens*	EU720483	ITS
Sapindaceae	*Magonia pubescens*	EU720819	rpoB
Sapindaceae	*Matayba elaeagnoides*	EU720553	ITS
Sapindaceae	*Matayba elaeagnoides*	KF420984	ITS
Sapindaceae	*Matayba elaeagnoides*	KF420985	ITS
Sapindaceae	*Matayba elaeagnoides*	KF420986	ITS
Sapindaceae	*Matayba elaeagnoides*	EU720699	matK
Sapindaceae	*Matayba elaeagnoides*	KF555408	matK
Sapindaceae	*Matayba elaeagnoides*	KF555409	matK

Family	Species	Accession	Barcode
Sapindaceae	*Matayba elaeagnoides*	KF555410	matK
Sapindaceae	*Matayba elaeagnoides*	KF421089	psbA-trnH
Sapindaceae	*Matayba elaeagnoides*	KF421090	psbA-trnH
Sapindaceae	*Matayba elaeagnoides*	KF421091	psbA-trnH
Sapindaceae	*Matayba elaeagnoides*	KF561927	rbcL
Sapindaceae	*Matayba elaeagnoides*	KF561928	rbcL
Sapindaceae	*Matayba elaeagnoides*	KF561929	rbcL
Sapindaceae	*Matayba elaeagnoides*	EU720901	rpoB
Sapindaceae	*Matayba elaeagnoides*	EU721323	trnL
Sapindaceae	*Matayba elaeagnoides*	EU721511	trnL
Sapindaceae	*Matayba guianensis*	EU720527	ITS
Sapindaceae	*Matayba guianensis*	EU720675	matK
Sapindaceae	*Matayba guianensis*	EU720872	rpoB
Sapindaceae	*Matayba guianensis*	EU721294	trnL
Sapindaceae	*Matayba guianensis*	EU721482	trnL
Sapindaceae	*Sapindus saponaria*	AY491661	matK
Sapindaceae	*Sapindus saponaria*	AY724324	matK
Sapindaceae	*Sapindus saponaria*	EU720639	matK
Sapindaceae	*Sapindus saponaria*	JQ589158	matK
Sapindaceae	*Sapindus saponaria*	JQ589159	matK
Sapindaceae	*Sapindus saponaria*	HG963928	psbA-trnH
Sapindaceae	*Sapindus saponaria*	AY724366	rbcL
Sapindaceae	*Sapindus saponaria*	GU935453	rbcL
Sapindaceae	*Sapindus saponaria*	JQ594032	rbcL
Sapindaceae	*Sapindus saponaria*	JQ594033	rbcL
Sapindaceae	*Sapindus saponaria*	KJ082549	rbcL
Sapindaceae	*Sapindus saponaria*	EU720804	rpoB
Sapindaceae	*Sapindus saponaria*	AJ413040	trnL
Sapindaceae	*Sapindus saponaria*	AY594534	trnL
Sapindaceae	*Sapindus saponaria*	EU721240	trnL
Sapindaceae	*Sapindus saponaria*	EU721428	trnL
Sapindaceae	*Talisia angustifolia*	EU720558	ITS
Sapindaceae	*Talisia angustifolia*	EU720705	matK
Sapindaceae	*Talisia angustifolia*	EU720907	rpoB
Sapindaceae	*Talisia angustifolia*	EU721328	trnL

Family	Species	Accession	Barcode
Sapindaceae	*Talisia angustifolia*	EU721516	trnL
Sapotaceae	*Chrysophyllum flexuosum*	KP092671	ITS
Sapotaceae	*Chrysophyllum flexuosum*	HQ415295	matK
Sapotaceae	*Chrysophyllum flexuosum*	KJ708868	matK
Sapotaceae	*Chrysophyllum flexuosum*	KJ708869	matK
Sapotaceae	*Chrysophyllum flexuosum*	KP094145	matK
Sapotaceae	*Chrysophyllum flexuosum*	HQ415467	psbA-trnH
Sapotaceae	*Chrysophyllum flexuosum*	KP095281	psbA-trnH
Sapotaceae	*Chrysophyllum flexuosum*	HQ415117	rbcL
Sapotaceae	*Chrysophyllum flexuosum*	KJ594653	rbcL
Sapotaceae	*Chrysophyllum flexuosum*	KP095113	rbcL
Sapotaceae	*Chrysophyllum gonocarpum*	JQ413927	matK
Sapotaceae	*Chrysophyllum gonocarpum*	JQ434255	psbA-trnH
Sapotaceae	*Chrysophyllum gonocarpum*	JQ413811	rbcL
Sapotaceae	*Chrysophyllum imperiale*	EF558615	ITS
Sapotaceae	*Chrysophyllum imperiale*	EF558600	psbA-trnH
Sapotaceae	*Chrysophyllum imperiale*	EF558603	trnL
Sapotaceae	*Diploon cuspidatum*	DQ246676	ITS
Sapotaceae	*Diploon cuspidatum*	FJ037870	ITS
Sapotaceae	*Diploon cuspidatum*	JQ434169	ITS
Sapotaceae	*Diploon cuspidatum*	JQ434170	ITS
Sapotaceae	*Diploon cuspidatum*	JQ434171	ITS
Sapotaceae	*Diploon cuspidatum*	JQ434172	ITS
Sapotaceae	*Diploon cuspidatum*	KF943855	ITS
Sapotaceae	*Diploon cuspidatum*	FJ037936	matK
Sapotaceae	*Diploon cuspidatum*	FJ514691	matK
Sapotaceae	*Diploon cuspidatum*	FJ514729	matK
Sapotaceae	*Diploon cuspidatum*	JQ413916	matK
Sapotaceae	*Diploon cuspidatum*	JQ413924	matK
Sapotaceae	*Diploon cuspidatum*	JQ413928	matK
Sapotaceae	*Diploon cuspidatum*	JQ626461	matK
Sapotaceae	*Diploon cuspidatum*	KF943848	matK
Sapotaceae	*Diploon cuspidatum*	DQ344105	psbA-trnH
Sapotaceae	*Diploon cuspidatum*	FJ039058	psbA-trnH
Sapotaceae	*Diploon cuspidatum*	JQ434241	psbA-trnH

**Family**	**Species**	**Accession**	**Barcode**
Sapotaceae	*Diploon cuspidatum*	JQ434242	psbA-trnH
Sapotaceae	*Diploon cuspidatum*	JQ434243	psbA-trnH
Sapotaceae	*Diploon cuspidatum*	JQ434244	psbA-trnH
Sapotaceae	*Diploon cuspidatum*	KF943863	psbA-trnH
Sapotaceae	*Diploon cuspidatum*	FJ038165	rbcL
Sapotaceae	*Diploon cuspidatum*	FJ038166	rbcL
Sapotaceae	*Diploon cuspidatum*	JQ413812	rbcL
Sapotaceae	*Diploon cuspidatum*	JQ413813	rbcL
Sapotaceae	*Diploon cuspidatum*	JQ413869	rbcL
Sapotaceae	*Diploon cuspidatum*	JQ413877	rbcL
Sapotaceae	*Diploon cuspidatum*	JQ626045	rbcL
Sapotaceae	*Diploon cuspidatum*	KF943834	rbcL
Sapotaceae	*Diploon cuspidatum*	FJ038369	rpoB
Sapotaceae	*Diploon cuspidatum*	FJ817182	rpoB
Sapotaceae	*Diploon cuspidatum*	FJ038779	rpoC1
Sapotaceae	*Diploon cuspidatum*	DQ344293	trnL
Sapotaceae	*Diploon cuspidatum*	FJ039182	trnL
Sapotaceae	*Diploon cuspidatum*	FJ039183	trnL
Sapotaceae	*Ecclinusa ramiflora*	DQ246678	ITS
Sapotaceae	*Ecclinusa ramiflora*	JQ434154	ITS
Sapotaceae	*Ecclinusa ramiflora*	JQ434155	ITS
Sapotaceae	*Ecclinusa ramiflora*	JQ434194	ITS
Sapotaceae	*Ecclinusa ramiflora*	JQ434195	ITS
Sapotaceae	*Ecclinusa ramiflora*	KF943854	ITS
Sapotaceae	*Ecclinusa ramiflora*	JQ413884	matK
Sapotaceae	*Ecclinusa ramiflora*	JQ413885	matK
Sapotaceae	*Ecclinusa ramiflora*	JQ413886	matK
Sapotaceae	*Ecclinusa ramiflora*	JQ413887	matK
Sapotaceae	*Ecclinusa ramiflora*	KF943846	matK
Sapotaceae	*Ecclinusa ramiflora*	DQ344108	psbA-trnH
Sapotaceae	*Ecclinusa ramiflora*	GQ428718	psbA-trnH
Sapotaceae	*Ecclinusa ramiflora*	GQ428719	psbA-trnH
Sapotaceae	*Ecclinusa ramiflora*	JQ434236	psbA-trnH
Sapotaceae	*Ecclinusa ramiflora*	JQ434237	psbA-trnH
Sapotaceae	*Ecclinusa ramiflora*	JQ434238	psbA-trnH

**Family**	**Species**	**Accession**	**Barcode**
Sapotaceae	*Ecclinusa ramiflora*	JQ434239	psbA-trnH
Sapotaceae	*Ecclinusa ramiflora*	KF943864	psbA-trnH
Sapotaceae	*Ecclinusa ramiflora*	JQ413837	rbcL
Sapotaceae	*Ecclinusa ramiflora*	JQ413838	rbcL
Sapotaceae	*Ecclinusa ramiflora*	JQ413839	rbcL
Sapotaceae	*Ecclinusa ramiflora*	JQ413840	rbcL
Sapotaceae	*Ecclinusa ramiflora*	JQ626076	rbcL
Sapotaceae	*Ecclinusa ramiflora*	KF943832	rbcL
Sapotaceae	*Ecclinusa ramiflora*	FJ038780	rpoC1
Sapotaceae	*Ecclinusa ramiflora*	DQ344296	trnL
Sapotaceae	*Manilkara salzmannii*	JQ434146	ITS
Sapotaceae	*Manilkara salzmannii*	JQ434147	ITS
Sapotaceae	*Manilkara salzmannii*	JQ434148	ITS
Sapotaceae	*Manilkara salzmannii*	JQ434149	ITS
Sapotaceae	*Manilkara salzmannii*	JQ434191	ITS
Sapotaceae	*Manilkara salzmannii*	JQ434192	ITS
Sapotaceae	*Manilkara salzmannii*	KM036005	ITS
Sapotaceae	*Manilkara salzmannii*	KM370949	ITS
Sapotaceae	*Manilkara salzmannii*	JQ413903	matK
Sapotaceae	*Manilkara salzmannii*	JQ413904	matK
Sapotaceae	*Manilkara salzmannii*	JQ413905	matK
Sapotaceae	*Manilkara salzmannii*	JQ413906	matK
Sapotaceae	*Manilkara salzmannii*	JQ413907	matK
Sapotaceae	*Manilkara salzmannii*	JQ413908	matK
Sapotaceae	*Manilkara salzmannii*	KM036003	matK
Sapotaceae	*Manilkara salzmannii*	JQ434211	psbA-trnH
Sapotaceae	*Manilkara salzmannii*	JQ434212	psbA-trnH
Sapotaceae	*Manilkara salzmannii*	JQ434213	psbA-trnH
Sapotaceae	*Manilkara salzmannii*	JQ434214	psbA-trnH
Sapotaceae	*Manilkara salzmannii*	JQ434215	psbA-trnH
Sapotaceae	*Manilkara salzmannii*	JQ434216	psbA-trnH
Sapotaceae	*Manilkara salzmannii*	KM036006	psbA-trnH
Sapotaceae	*Manilkara salzmannii*	JQ413856	rbcL
Sapotaceae	*Manilkara salzmannii*	JQ413857	rbcL
Sapotaceae	*Manilkara salzmannii*	JQ413858	rbcL

Family	Species	Accession	Barcode
Sapotaceae	*Manilkara salzmannii*	JQ413859	rbcL
Sapotaceae	*Manilkara salzmannii*	JQ413860	rbcL
Sapotaceae	*Manilkara salzmannii*	JQ413861	rbcL
Sapotaceae	*Manilkara salzmannii*	KM036004	rbcL
Sapotaceae	*Manilkara subsericea*	KM370957	ITS
Sapotaceae	*Micropholis compta*	JQ413914	matK
Sapotaceae	*Micropholis compta*	JQ434226	psbA-trnH
Sapotaceae	*Micropholis compta*	JQ413867	rbcL
Sapotaceae	*Micropholis crassipedicellata*	JQ434163	ITS
Sapotaceae	*Micropholis crassipedicellata*	JQ434164	ITS
Sapotaceae	*Micropholis crassipedicellata*	JQ434165	ITS
Sapotaceae	*Micropholis crassipedicellata*	JQ413911	matK
Sapotaceae	*Micropholis crassipedicellata*	JQ413912	matK
Sapotaceae	*Micropholis crassipedicellata*	JQ413913	matK
Sapotaceae	*Micropholis crassipedicellata*	JQ413923	matK
Sapotaceae	*Micropholis crassipedicellata*	JQ413925	matK
Sapotaceae	*Micropholis crassipedicellata*	JQ434222	psbA-trnH
Sapotaceae	*Micropholis crassipedicellata*	JQ434223	psbA-trnH
Sapotaceae	*Micropholis crassipedicellata*	JQ434224	psbA-trnH
Sapotaceae	*Micropholis crassipedicellata*	JQ434225	psbA-trnH
Sapotaceae	*Micropholis crassipedicellata*	JQ413864	rbcL
Sapotaceae	*Micropholis crassipedicellata*	JQ413865	rbcL
Sapotaceae	*Micropholis crassipedicellata*	JQ413866	rbcL
Sapotaceae	*Micropholis crassipedicellata*	JQ413876	rbcL
Sapotaceae	*Micropholis crassipedicellata*	JQ413878	rbcL
Sapotaceae	*Micropholis gardneriana*	JQ434158	ITS
Sapotaceae	*Micropholis gardneriana*	JQ434159	ITS
Sapotaceae	*Micropholis gardneriana*	JQ434160	ITS
Sapotaceae	*Micropholis gardneriana*	JQ434161	ITS
Sapotaceae	*Micropholis gardneriana*	JQ413909	matK
Sapotaceae	*Micropholis gardneriana*	JQ413910	matK
Sapotaceae	*Micropholis gardneriana*	JQ413915	matK
Sapotaceae	*Micropholis gardneriana*	JQ413917	matK
Sapotaceae	*Micropholis gardneriana*	JQ434217	psbA-trnH
Sapotaceae	*Micropholis gardneriana*	JQ434218	psbA-trnH

**Family**	**Species**	**Accession**	**Barcode**
Sapotaceae	*Micropholis gardneriana*	JQ434219	psbA-trnH
Sapotaceae	*Micropholis gardneriana*	JQ434220	psbA-trnH
Sapotaceae	*Micropholis gardneriana*	JQ413862	rbcL
Sapotaceae	*Micropholis gardneriana*	JQ413863	rbcL
Sapotaceae	*Micropholis gardneriana*	JQ413868	rbcL
Sapotaceae	*Micropholis gardneriana*	JQ413870	rbcL
Sapotaceae	*Micropholis venulosa*	DQ246683	ITS
Sapotaceae	*Micropholis venulosa*	FJ037884	ITS
Sapotaceae	*Micropholis venulosa*	FJ037885	ITS
Sapotaceae	*Micropholis venulosa*	FJ037938	matK
Sapotaceae	*Micropholis venulosa*	FJ514727	matK
Sapotaceae	*Micropholis venulosa*	FJ514736	matK
Sapotaceae	*Micropholis venulosa*	JQ626490	matK
Sapotaceae	*Micropholis venulosa*	DQ344117	psbA-trnH
Sapotaceae	*Micropholis venulosa*	FJ039067	psbA-trnH
Sapotaceae	*Micropholis venulosa*	FJ039068	psbA-trnH
Sapotaceae	*Micropholis venulosa*	FJ038175	rbcL
Sapotaceae	*Micropholis venulosa*	FJ038176	rbcL
Sapotaceae	*Micropholis venulosa*	GQ428628	rbcL
Sapotaceae	*Micropholis venulosa*	JQ626105	rbcL
Sapotaceae	*Micropholis venulosa*	FJ038372	rpoB
Sapotaceae	*Micropholis venulosa*	FJ038373	rpoB
Sapotaceae	*Micropholis venulosa*	FJ038784	rpoC1
Sapotaceae	*Micropholis venulosa*	FJ038786	rpoC1
Sapotaceae	*Micropholis venulosa*	FJ038787	rpoC1
Sapotaceae	*Micropholis venulosa*	DQ344305	trnL
Sapotaceae	*Micropholis venulosa*	FJ039195	trnL
Sapotaceae	*Micropholis venulosa*	FJ039196	trnL
Sapotaceae	*Pouteria beaurepairei*	KJ399375	ITS
Sapotaceae	*Pouteria caimito*	JQ434181	ITS
Sapotaceae	*Pouteria caimito*	KF943861	ITS
Sapotaceae	*Pouteria caimito*	KJ399378	ITS
Sapotaceae	*Pouteria caimito*	KJ399379	ITS
Sapotaceae	*Pouteria caimito*	KJ399380	ITS
Sapotaceae	*Pouteria caimito*	KJ399381	ITS

**Family**	**Species**	**Accession**	**Barcode**
Sapotaceae	*Pouteria caimito*	KJ399439	ITS
Sapotaceae	*Pouteria caimito*	JQ413932	matK
Sapotaceae	*Pouteria caimito*	KF943850	matK
Sapotaceae	*Pouteria caimito*	JQ434245	psbA-trnH
Sapotaceae	*Pouteria caimito*	KF943871	psbA-trnH
Sapotaceae	*Pouteria caimito*	JQ413818	rbcL
Sapotaceae	*Pouteria caimito*	KF943837	rbcL
Sapotaceae	*Pouteria gardneri*	JQ434178	ITS
Sapotaceae	*Pouteria gardneri*	KJ399437	ITS
Sapotaceae	*Pouteria gardneri*	JQ413936	matK
Sapotaceae	*Pouteria gardneri*	JQ434260	psbA-trnH
Sapotaceae	*Pouteria gardneri*	JQ413822	rbcL
Sapotaceae	*Pouteria gardneriana*	DQ246689	ITS
Sapotaceae	*Pouteria gardneriana*	DQ344133	psbA-trnH
Sapotaceae	*Pouteria gardneriana*	DQ344321	trnL
Sapotaceae	*Pouteria glomerata*	JQ625786	rbcL
Sapotaceae	*Pouteria grandiflora*	KF943862	ITS
Sapotaceae	*Pouteria grandiflora*	KF943843	matK
Sapotaceae	*Pouteria grandiflora*	KF943844	matK
Sapotaceae	*Pouteria grandiflora*	KF943845	matK
Sapotaceae	*Pouteria grandiflora*	KF943868	psbA-trnH
Sapotaceae	*Pouteria grandiflora*	KF943829	rbcL
Sapotaceae	*Pouteria grandiflora*	KF943830	rbcL
Sapotaceae	*Pouteria grandiflora*	KF943831	rbcL
Sapotaceae	*Pouteria oxypetala*	KJ399417	ITS
Sapotaceae	*Pouteria ramiflora*	KJ399432	ITS
Sapotaceae	*Pouteria reticulata*	JQ434184	ITS
Sapotaceae	*Pouteria reticulata*	JQ434185	ITS
Sapotaceae	*Pouteria reticulata*	JQ434186	ITS
Sapotaceae	*Pouteria reticulata*	KJ399434	ITS
Sapotaceae	*Pouteria reticulata*	JQ413940	matK
Sapotaceae	*Pouteria reticulata*	JQ413941	matK
Sapotaceae	*Pouteria reticulata*	JQ413942	matK
Sapotaceae	*Pouteria reticulata*	JQ589187	matK
Sapotaceae	*Pouteria reticulata*	JQ589188	matK

Family	Species	Accession	Barcode
Sapotaceae	*Pouteria reticulata*	JQ589189	matK
Sapotaceae	*Pouteria reticulata*	JQ589190	matK
Sapotaceae	*Pouteria reticulata*	GQ982327	psbA-trnH
Sapotaceae	*Pouteria reticulata*	JQ434247	psbA-trnH
Sapotaceae	*Pouteria reticulata*	JQ434248	psbA-trnH
Sapotaceae	*Pouteria reticulata*	JQ434249	psbA-trnH
Sapotaceae	*Pouteria reticulata*	GQ428632	rbcL
Sapotaceae	*Pouteria reticulata*	GQ981841	rbcL
Sapotaceae	*Pouteria reticulata*	JQ413826	rbcL
Sapotaceae	*Pouteria reticulata*	JQ413827	rbcL
Sapotaceae	*Pouteria reticulata*	JQ413828	rbcL
Sapotaceae	*Pouteria reticulata*	JQ594074	rbcL
Sapotaceae	*Pouteria reticulata*	JQ594075	rbcL
Sapotaceae	*Pouteria reticulata*	JQ594076	rbcL
Sapotaceae	*Pouteria reticulata*	JQ594077	rbcL
Sapotaceae	*Pouteria reticulata*	JQ594078	rbcL
Sapotaceae	*Pouteria reticulata*	JQ625962	rbcL
Sapotaceae	*Pouteria torta*	DQ787391	ITS
Sapotaceae	*Pouteria torta*	FJ037896	ITS
Sapotaceae	*Pouteria torta*	KJ399438	ITS
Sapotaceae	*Pouteria torta*	KJ399443	ITS
Sapotaceae	*Pouteria torta*	KJ399444	ITS
Sapotaceae	*Pouteria torta*	FJ037947	matK
Sapotaceae	*Pouteria torta*	FJ514643	matK
Sapotaceae	*Pouteria torta*	JQ626418	matK
Sapotaceae	*Pouteria torta*	FJ039081	psbA-trnH
Sapotaceae	*Pouteria torta*	FJ039082	psbA-trnH
Sapotaceae	*Pouteria torta*	FJ038192	rbcL
Sapotaceae	*Pouteria torta*	FJ038193	rbcL
Sapotaceae	*Pouteria torta*	GQ428636	rbcL
Sapotaceae	*Pouteria torta*	GQ428637	rbcL
Sapotaceae	*Pouteria torta*	JQ594079	rbcL
Sapotaceae	*Pouteria torta*	JQ625931	rbcL
Sapotaceae	*Pouteria torta*	FJ038387	rpoB
Sapotaceae	*Pouteria torta*	FJ038817	rpoC1

**Family**	**Species**	**Accession**	**Barcode**
Sapotaceae	*Pouteria torta*	FJ038818	rpoC1
Sapotaceae	*Pouteria torta*	FJ038819	rpoC1
Sapotaceae	*Pouteria torta*	FJ039208	trnL
Sapotaceae	*Pouteria torta*	FJ039209	trnL
Sapotaceae	*Pouteria venosa*	JQ434188	ITS
Sapotaceae	*Pouteria venosa*	KJ399445	ITS
Sapotaceae	*Pouteria venosa*	JQ413944	matK
Sapotaceae	*Pouteria venosa*	JQ413945	matK
Sapotaceae	*Pouteria venosa*	JQ434251	psbA-trnH
Sapotaceae	*Pouteria venosa*	JQ434252	psbA-trnH
Sapotaceae	*Pouteria venosa*	JQ413830	rbcL
Sapotaceae	*Pouteria venosa*	JQ413831	rbcL
Sapotaceae	*Pradosia lactescens*	JQ434156	ITS
Sapotaceae	*Pradosia lactescens*	JQ434157	ITS
Sapotaceae	*Pradosia lactescens*	JQ434196	ITS
Sapotaceae	*Pradosia lactescens*	JQ434197	ITS
Sapotaceae	*Pradosia lactescens*	KF943857	ITS
Sapotaceae	*Pradosia lactescens*	KM042317	ITS
Sapotaceae	*Pradosia lactescens*	KM042318	ITS
Sapotaceae	*Pradosia lactescens*	KM042319	ITS
Sapotaceae	*Pradosia lactescens*	JQ413888	matK
Sapotaceae	*Pradosia lactescens*	JQ413889	matK
Sapotaceae	*Pradosia lactescens*	JQ413890	matK
Sapotaceae	*Pradosia lactescens*	JQ413891	matK
Sapotaceae	*Pradosia lactescens*	KF943842	matK
Sapotaceae	*Pradosia lactescens*	JQ434240	psbA-trnH
Sapotaceae	*Pradosia lactescens*	JQ413841	rbcL
Sapotaceae	*Pradosia lactescens*	JQ413842	rbcL
Sapotaceae	*Pradosia lactescens*	JQ413843	rbcL
Sapotaceae	*Pradosia lactescens*	JQ413844	rbcL
Sapotaceae	*Pradosia lactescens*	KF943836	rbcL
Sapotaceae	*Sideroxylon obtusifolium*	AM408087	ITS
Sapotaceae	*Sideroxylon obtusifolium*	AM179763	psbA-trnH
Sapotaceae	*Sideroxylon obtusifolium*	HG963822	psbA-trnH
Simaroubaceae	*Picrasma crenata*	EU042872	matK

**Family**	**Species**	**Accession**	**Barcode**
Simaroubaceae	*Picrasma crenata*	EU043010	rbcL
Simaroubaceae	*Simaba insignis*	EU042893	matK
Simaroubaceae	*Simaba insignis*	EU043031	rbcL
Siparunaceae	*Siparuna bifida*	AF289832	ITS
Siparunaceae	*Siparuna bifida*	AF289853	trnL
Siparunaceae	*Siparuna brasiliensis*	DQ401375	matK
Siparunaceae	*Siparuna brasiliensis*	AF013246	rbcL
Siparunaceae	*Siparuna brasiliensis*	AF012408	trnL
Siparunaceae	*Siparuna guianensis*	AF289829	ITS
Siparunaceae	*Siparuna guianensis*	GQ982092	matK
Siparunaceae	*Siparuna guianensis*	GQ982360	psbA-trnH
Siparunaceae	*Siparuna guianensis*	GQ981872	rbcL
Siparunaceae	*Siparuna guianensis*	AF040697	trnL
Siparunaceae	*Siparuna guianensis*	AF289850	trnL
Siparunaceae	*Siparuna reginae*	AF289831	ITS
Siparunaceae	*Siparuna reginae*	AF289852	trnL
Solanaceae	*Acnistus arborescens*	DQ314173	ITS
Solanaceae	*Acnistus arborescens*	DQ314181	ITS
Solanaceae	*Acnistus arborescens*	DQ314183	ITS
Solanaceae	*Acnistus arborescens*	KP756806	matK
Solanaceae	*Acnistus arborescens*	KJ426590	psbA-trnH
Solanaceae	*Acnistus arborescens*	KJ082106	rbcL
Solanaceae	*Acnistus arborescens*	EU580954	trnL
Solanaceae	*Acnistus arborescens*	KP756700	trnL
Solanaceae	*Athenaea micrantha*	KC832780	ITS
Solanaceae	*Athenaea micrantha*	KC549612	trnL
Solanaceae	*Athenaea micrantha*	KC549631	trnL
Solanaceae	*Athenaea pereirae*	KC832788	ITS
Solanaceae	*Athenaea pereirae*	KC549620	trnL
Solanaceae	*Athenaea pereirae*	KC549639	trnL
Solanaceae	*Athenaea pogogena*	KC832790	ITS
Solanaceae	*Athenaea pogogena*	KF720738	ITS
Solanaceae	*Athenaea pogogena*	KC549622	trnL
Solanaceae	*Athenaea pogogena*	KC549641	trnL
Solanaceae	*Athenaea pogogena*	KF720760	trnL

Family	Species	Accession	Barcode
Solanaceae	*Aureliana brasiliana*	KC832783	ITS
Solanaceae	*Aureliana brasiliana*	KC549615	trnL
Solanaceae	*Aureliana brasiliana*	KC549634	trnL
Solanaceae	*Aureliana cuspidata*	KC832784	ITS
Solanaceae	*Aureliana cuspidata*	KC549616	trnL
Solanaceae	*Aureliana cuspidata*	KC549635	trnL
Solanaceae	*Aureliana darcyi*	KC832785	ITS
Solanaceae	*Aureliana darcyi*	KC549617	trnL
Solanaceae	*Aureliana darcyi*	KC549636	trnL
Solanaceae	*Aureliana fasciculata*	KC832786	ITS
Solanaceae	*Aureliana fasciculata*	KC832791	ITS
Solanaceae	*Aureliana fasciculata*	KC832798	ITS
Solanaceae	*Aureliana fasciculata*	EF537319	matK
Solanaceae	*Aureliana fasciculata*	EF537252	psbA-trnH
Solanaceae	*Aureliana fasciculata*	JQ744337-JQ744448	psbA-trnH
Solanaceae	*Aureliana fasciculata*	EU580961	trnL
Solanaceae	*Aureliana fasciculata*	KC549618	trnL
Solanaceae	*Aureliana fasciculata*	KC549623	trnL
Solanaceae	*Aureliana fasciculata*	KC549630	trnL
Solanaceae	*Aureliana fasciculata*	KC549637	trnL
Solanaceae	*Aureliana fasciculata*	KC549642	trnL
Solanaceae	*Aureliana fasciculata*	KC549649	trnL
Solanaceae	*Aureliana sellowiana*	KC832781	ITS
Solanaceae	*Aureliana sellowiana*	KC549613	trnL
Solanaceae	*Aureliana sellowiana*	KC549632	trnL
Solanaceae	*Aureliana wettsteiniana*	KC832793	ITS
Solanaceae	*Aureliana wettsteiniana*	EF537253	psbA-trnH
Solanaceae	*Aureliana wettsteiniana*	KC549625	trnL
Solanaceae	*Aureliana wettsteiniana*	KC549644	trnL
Solanaceae	*Brugmansia suaveolens*	HG738853	ITS
Solanaceae	*Brugmansia suaveolens*	JX467597	ITS
Solanaceae	*Brugmansia suaveolens*	LC076493	ITS
Solanaceae	*Brugmansia suaveolens*	HM851090	matK
Solanaceae	*Brugmansia suaveolens*	KC146589	matK
Solanaceae	*Brugmansia suaveolens*	KP756824	matK

Family	Species	Accession	Barcode
Solanaceae	*Brugmansia suaveolens*	KC146624	psbA-trnH
Solanaceae	*Brugmansia suaveolens*	HM849829	rbcL
Solanaceae	*Brugmansia suaveolens*	JX467580	trnL
Solanaceae	*Brugmansia suaveolens*	KC146642	trnL
Solanaceae	*Brugmansia suaveolens*	KP756710	trnL
Solanaceae	*Brunfelsia brasiliensis*	JQ081183	ITS
Solanaceae	*Brunfelsia brasiliensis*	JQ081184	ITS
Solanaceae	*Brunfelsia brasiliensis*	JQ081077	trnL
Solanaceae	*Brunfelsia brasiliensis*	JQ081078	trnL
Solanaceae	*Brunfelsia pauciflora*	JQ081213	ITS
Solanaceae	*Brunfelsia pauciflora*	JQ081108	trnL
Solanaceae	*Brunfelsia uniflora*	JQ081224	ITS
Solanaceae	*Brunfelsia uniflora*	JQ081225	ITS
Solanaceae	*Brunfelsia uniflora*	LC076496	ITS
Solanaceae	*Brunfelsia uniflora*	KP756838	matK
Solanaceae	*Brunfelsia uniflora*	JX856326	psbA-trnH
Solanaceae	*Brunfelsia uniflora*	JX856305	rbcL
Solanaceae	*Brunfelsia uniflora*	JX856284	rpoB
Solanaceae	*Brunfelsia uniflora*	EU580967	trnL
Solanaceae	*Brunfelsia uniflora*	JQ081119	trnL
Solanaceae	*Brunfelsia uniflora*	JQ081120	trnL
Solanaceae	*Capsicum flexuosum*	EF537280	matK
Solanaceae	*Capsicum flexuosum*	EF537281	matK
Solanaceae	*Capsicum flexuosum*	EF537282	matK
Solanaceae	*Capsicum flexuosum*	EF537283	matK
Solanaceae	*Capsicum flexuosum*	EF537229	psbA-trnH
Solanaceae	*Capsicum flexuosum*	EF537230	psbA-trnH
Solanaceae	*Capsicum flexuosum*	EF537231	psbA-trnH
Solanaceae	*Capsicum flexuosum*	EF537232	psbA-trnH
Solanaceae	*Capsicum flexuosum*	AY348962	trnL
Solanaceae	*Capsicum flexuosum*	AY349008	trnL
Solanaceae	*Capsicum flexuosum*	DQ077643	trnL
Solanaceae	*Capsicum recurvatum*	EF537286	matK
Solanaceae	*Capsicum recurvatum*	EF537287	matK
Solanaceae	*Capsicum recurvatum*	EF537288	matK

**Family**	**Species**	**Accession**	**Barcode**
Solanaceae	*Capsicum recurvatum*	EF537318	matK
Solanaceae	*Capsicum recurvatum*	EF537240	psbA-trnH
Solanaceae	*Capsicum recurvatum*	EF537241	psbA-trnH
Solanaceae	*Capsicum recurvatum*	EF537242	psbA-trnH
Solanaceae	*Capsicum recurvatum*	EF537243	psbA-trnH
Solanaceae	*Cestrum axillare*	JX517961	matK
Solanaceae	*Cestrum axillare*	JX572398	rbcL
Solanaceae	*Cestrum intermedium*	KP100277	ITS
Solanaceae	*Cestrum intermedium*	JQ589215-JQ589227	matK
Solanaceae	*Cestrum intermedium*	JQ589573	matK
Solanaceae	*Cestrum intermedium*	JQ589574	matK
Solanaceae	*Cestrum intermedium*	JQ589575	matK
Solanaceae	*Cestrum intermedium*	GQ982180	psbA-trnH
Solanaceae	*Cestrum intermedium*	HG963906	psbA-trnH
Solanaceae	*Cestrum intermedium*	JQ594112-JQ594123	rbcL
Solanaceae	*Cestrum intermedium*	JQ594559	rbcL
Solanaceae	*Cestrum intermedium*	JQ594560	rbcL
Solanaceae	*Cestrum intermedium*	JQ594561	rbcL
Solanaceae	*Cestrum intermedium*	EU580973	trnL
Solanaceae	*Cestrum strigilatum*	KP756810	matK
Solanaceae	*Cestrum strigilatum*	EU580976	trnL
Solanaceae	*Dyssochroma viridiflorum*	EU580989	trnL
Solanaceae	*Lycianthes pauciflora*	JQ589252	matK
Solanaceae	*Lycianthes pauciflora*	JQ589253	matK
Solanaceae	*Lycianthes pauciflora*	JQ589254	matK
Solanaceae	*Lycianthes pauciflora*	JQ594154	rbcL
Solanaceae	*Lycianthes pauciflora*	JQ594155	rbcL
Solanaceae	*Lycianthes pauciflora*	JQ594156	rbcL
Solanaceae	*Solanum betaceum*	AF244713	ITS
Solanaceae	*Solanum betaceum*	AY523871	ITS
Solanaceae	*Solanum betaceum*	AY523872	ITS
Solanaceae	*Solanum betaceum*	AY523873	ITS
Solanaceae	*Solanum betaceum*	AY523874	ITS
Solanaceae	*Solanum betaceum*	AY523875	ITS
Solanaceae	*Solanum betaceum*	AY523876	ITS

Family	Species	Accession	Barcode
Solanaceae	*Solanum betaceum*	EF438983	matK
Solanaceae	*Solanum betaceum*	DQ180426	trnL
Solanaceae	*Solanum betaceum*	DQ855055	trnL
Solanaceae	*Solanum betaceum*	HM006830	trnL
Solanaceae	*Solanum cernuum*	DQ837371	ITS
Solanaceae	*Solanum crinitum*	AY996501	ITS
Solanaceae	*Solanum crinitum*	GQ143651	ITS
Solanaceae	*Solanum crinitum*	GQ143652	ITS
Solanaceae	*Solanum crinitum*	DQ180482	trnL
Solanaceae	*Solanum crinitum*	GQ149736	trnL
Solanaceae	*Solanum crinitum*	GQ149737	trnL
Solanaceae	*Solanum decorum*	HQ457396	ITS
Solanaceae	*Solanum decorum*	HQ457405	trnL
Solanaceae	*Solanum diploconos*	AY523890	ITS
Solanaceae	*Solanum diploconos*	AY875751	ITS
Solanaceae	*Solanum diploconos*	DQ180429	trnL
Solanaceae	*Solanum latiflorum*	AY523900	ITS
Solanaceae	*Solanum lycocarpum*	AY996525	ITS
Solanaceae	*Solanum lycocarpum*	DQ812107	trnL
Solanaceae	*Solanum mauritianum*	EF439031	matK
Solanaceae	*Solanum mauritianum*	HM851104	matK
Solanaceae	*Solanum mauritianum*	JX517446	matK
Solanaceae	*Solanum mauritianum*	HM850366	rbcL
Solanaceae	*Solanum mauritianum*	JX572987	rbcL
Solanaceae	*Solanum mauritianum*	DQ180487	trnL
Solanaceae	*Solanum mauritianum*	HM006828	trnL
Solanaceae	*Solanum mauritianum*	JF804931	trnL
Solanaceae	*Solanum melissarum*	AY523926	ITS
Solanaceae	*Solanum pinetorum*	AY523912	ITS
Solanaceae	*Solanum sciadostylis*	AY523917	ITS
Solanaceae	*Solanum scuticum*	HQ457400	ITS
Solanaceae	*Solanum scuticum*	HQ457410	trnL
Solanaceae	*Vassobia breviflora*	DQ314190	ITS
Solanaceae	*Vassobia breviflora*	EF439077	matK
Solanaceae	*Vassobia breviflora*	EF439078	matK

**Family**	**Species**	**Accession**	**Barcode**
Solanaceae	*Vassobia breviflora*	AF212022	trnL
Solanaceae	*Vassobia breviflora*	KC549627	trnL
Solanaceae	*Vassobia breviflora*	KC549646	trnL
Styracaceae	*Styrax camporum*	AF327504	ITS
Styracaceae	*Styrax ferrugineus*	AF327503	ITS
Styracaceae	*Styrax latifolius*	AY143585	ITS
Styracaceae	*Styrax leprosus*	AY143586	ITS
Styracaceae	*Styrax martii*	AF327508	ITS
Styracaceae	*Styrax martii*	AY143584	ITS
Styracaceae	*Styrax pohlii*	AY143581	ITS
Styracaceae	*Styrax sieberi*	AY143587	ITS
Styracaceae	*Styrax sieberi*	AY143588	ITS
Symplocaceae	*Symplocos bidana*	KF002837	ITS
Symplocaceae	*Symplocos bidana*	KF002882	matK
Symplocaceae	*Symplocos bidana*	KF002937	trnL
Symplocaceae	*Symplocos celastrinea*	AY336266	ITS
Symplocaceae	*Symplocos celastrinea*	DQ086266	ITS
Symplocaceae	*Symplocos celastrinea*	DQ086280	matK
Symplocaceae	*Symplocos celastrinea*	DQ086401	trnL
Symplocaceae	*Symplocos corymboclados*	KF002841	ITS
Symplocaceae	*Symplocos corymboclados*	KF002886	matK
Symplocaceae	*Symplocos corymboclados*	KF002941	trnL
Symplocaceae	*Symplocos estrellensis*	DQ086272	ITS
Symplocaceae	*Symplocos estrellensis*	DQ086286	matK
Symplocaceae	*Symplocos estrellensis*	DQ086407	trnL
Symplocaceae	*Symplocos falcata*	DQ086276	ITS
Symplocaceae	*Symplocos falcata*	DQ086277	ITS
Symplocaceae	*Symplocos falcata*	DQ086290	matK
Symplocaceae	*Symplocos falcata*	DQ086291	matK
Symplocaceae	*Symplocos falcata*	DQ086411	trnL
Symplocaceae	*Symplocos falcata*	DQ086412	trnL
Symplocaceae	*Symplocos glandulosomarginata*	KF002846	ITS
Symplocaceae	*Symplocos glandulosomarginata*	KF002892	matK
Symplocaceae	*Symplocos glandulosomarginata*	KF002946	trnL
Symplocaceae	*Symplocos incrassata*	KF002847	ITS

Family	Species	Accession	Barcode
Symplocaceae	*Symplocos incrassata*	KF002893	matK
Symplocaceae	*Symplocos incrassata*	KF002947	trnL
Symplocaceae	*Symplocos itatiaiae*	DQ086268	ITS
Symplocaceae	*Symplocos itatiaiae*	DQ086282	matK
Symplocaceae	*Symplocos itatiaiae*	DQ086403	trnL
Symplocaceae	*Symplocos kleinii*	AY336267	ITS
Symplocaceae	*Symplocos kleinii*	KF002853	ITS
Symplocaceae	*Symplocos kleinii*	KF002895	matK
Symplocaceae	*Symplocos kleinii*	AY336432	trnL
Symplocaceae	*Symplocos kleinii*	KF002949	trnL
Symplocaceae	*Symplocos nitens*	DQ086273	ITS
Symplocaceae	*Symplocos nitens*	DQ086287	matK
Symplocaceae	*Symplocos nitens*	DQ086408	trnL
Symplocaceae	*Symplocos nitidiflora*	KF002867	ITS
Symplocaceae	*Symplocos nitidiflora*	KF002907	matK
Symplocaceae	*Symplocos nitidiflora*	KF002955	trnL
Symplocaceae	*Symplocos pentandra*	DQ086270	ITS
Symplocaceae	*Symplocos pentandra*	DQ086271	ITS
Symplocaceae	*Symplocos pentandra*	DQ086284	matK
Symplocaceae	*Symplocos pentandra*	DQ086285	matK
Symplocaceae	*Symplocos pentandra*	DQ086405	trnL
Symplocaceae	*Symplocos pentandra*	DQ086406	trnL
Symplocaceae	*Symplocos platyphylla*	KF002870	ITS
Symplocaceae	*Symplocos platyphylla*	KF002910	matK
Symplocaceae	*Symplocos platyphylla*	KF002958	trnL
Symplocaceae	*Symplocos pubescens*	KF002871	ITS
Symplocaceae	*Symplocos pubescens*	KF002911	matK
Symplocaceae	*Symplocos pubescens*	KF002959	trnL
Symplocaceae	*Symplocos tenuifolia*	AY336290	ITS
Symplocaceae	*Symplocos tenuifolia*	KF002879	ITS
Symplocaceae	*Symplocos tenuifolia*	AY336356	matK
Symplocaceae	*Symplocos tenuifolia*	KF002920	matK
Symplocaceae	*Symplocos tetrandra*	KF002859	ITS
Symplocaceae	*Symplocos tetrandra*	KF002905	matK
Symplocaceae	*Symplocos tetrandra*	KF002963	trnL

Family	Species	Accession	Barcode
Symplocaceae	*Symplocos uniflora*	DQ086274	ITS
Symplocaceae	*Symplocos uniflora*	DQ086288	matK
Symplocaceae	*Symplocos uniflora*	DQ086409	trnL
Ulmaceae	*Phyllostylon rhamnoides*	KC539585	ITS
Ulmaceae	*Phyllostylon rhamnoides*	KC539591	ITS
Ulmaceae	*Phyllostylon rhamnoides*	KC539592	ITS
Ulmaceae	*Phyllostylon rhamnoides*	KC539594	ITS
Ulmaceae	*Phyllostylon rhamnoides*	KC539596	ITS
Ulmaceae	*Phyllostylon rhamnoides*	KC539603	ITS
Ulmaceae	*Phyllostylon rhamnoides*	KC539619	matK
Ulmaceae	*Phyllostylon rhamnoides*	KC539625	matK
Ulmaceae	*Phyllostylon rhamnoides*	KC539626	matK
Ulmaceae	*Phyllostylon rhamnoides*	KC539628	matK
Ulmaceae	*Phyllostylon rhamnoides*	KC539630	matK
Ulmaceae	*Phyllostylon rhamnoides*	KC539637	matK
Ulmaceae	*Phyllostylon rhamnoides*	KC539689	rbcL
Ulmaceae	*Phyllostylon rhamnoides*	KC539695	rbcL
Ulmaceae	*Phyllostylon rhamnoides*	KC539696	rbcL
Ulmaceae	*Phyllostylon rhamnoides*	KC539697	rbcL
Ulmaceae	*Phyllostylon rhamnoides*	KC539699	rbcL
Ulmaceae	*Phyllostylon rhamnoides*	KC539706	rbcL
Ulmaceae	*Phyllostylon rhamnoides*	KC539723	trnL
Ulmaceae	*Phyllostylon rhamnoides*	KC539728	trnL
Ulmaceae	*Phyllostylon rhamnoides*	KC539729	trnL
Ulmaceae	*Phyllostylon rhamnoides*	KC539731	trnL
Ulmaceae	*Phyllostylon rhamnoides*	KC539733	trnL
Ulmaceae	*Phyllostylon rhamnoides*	KC539740	trnL
Urticaceae	*Boehmeria caudata*	HG963861	psbA-trnH
Urticaceae	*Urera baccifera*	KF137928	ITS
Urticaceae	*Urera baccifera*	KM586468	ITS
Urticaceae	*Urera baccifera*	KM586469	ITS
Urticaceae	*Urera baccifera*	GQ982123	matK
Urticaceae	*Urera baccifera*	HM446752	matK
Urticaceae	*Urera baccifera*	JQ589402	matK
Urticaceae	*Urera baccifera*	JQ589403	matK

**Family**	**Species**	**Accession**	**Barcode**
Urticaceae	*Urera baccifera*	JQ589404	matK
Urticaceae	*Urera baccifera*	JQ589405	matK
Urticaceae	*Urera baccifera*	JQ589406	matK
Urticaceae	*Urera baccifera*	JQ589407	matK
Urticaceae	*Urera baccifera*	KF138065	matK
Urticaceae	*Urera baccifera*	GQ982399	psbA-trnH
Urticaceae	*Urera baccifera*	HM447011	psbA-trnH
Urticaceae	*Urera baccifera*	GQ981911	rbcL
Urticaceae	*Urera baccifera*	HM446881	rbcL
Urticaceae	*Urera baccifera*	JQ594341	rbcL
Urticaceae	*Urera baccifera*	JQ594342	rbcL
Urticaceae	*Urera baccifera*	JQ594343	rbcL
Urticaceae	*Urera baccifera*	JQ594344	rbcL
Urticaceae	*Urera baccifera*	JQ594345	rbcL
Urticaceae	*Urera baccifera*	KF138249	rbcL
Urticaceae	*Urera baccifera*	KM586554	rbcL
Urticaceae	*Urera baccifera*	KM586555	rbcL
Urticaceae	*Urera baccifera*	KF138414	trnL
Urticaceae	*Urera baccifera*	KM586640	trnL
Urticaceae	*Urera baccifera*	KM586641	trnL
Urticaceae	*Urera caracasana*	KF137929	ITS
Urticaceae	*Urera caracasana*	KM586467	ITS
Urticaceae	*Urera caracasana*	JQ589408	matK
Urticaceae	*Urera caracasana*	KF138064	matK
Urticaceae	*Urera caracasana*	JQ594346	rbcL
Urticaceae	*Urera caracasana*	JQ594347	rbcL
Urticaceae	*Urera caracasana*	KF138248	rbcL
Urticaceae	*Urera caracasana*	KF138250	rbcL
Urticaceae	*Urera caracasana*	KM586553	rbcL
Urticaceae	*Urera caracasana*	KF138413	trnL
Urticaceae	*Urera caracasana*	KF138415	trnL
Urticaceae	*Urera caracasana*	KM586639	trnL
Verbenaceae	*Aloysia virgata*	FJ867398	ITS
Verbenaceae	*Aloysia virgata*	HM853863	matK
Verbenaceae	*Aloysia virgata*	EF571570	trnL

Family	Species	Accession	Barcode
Verbenaceae	*Aloysia virgata*	JX966960	trnL
Verbenaceae	*Aloysia virgata*	KF688774	trnL
Verbenaceae	*Citharexylum montevidense*	HM853879	matK
Verbenaceae	*Citharexylum montevidense*	HM216616	trnL
Verbenaceae	*Citharexylum montevidense*	JX966962	trnL
Verbenaceae	*Lantana camara*	AF437853-AF437873	ITS
Verbenaceae	*Lantana camara*	AF477784	ITS
Verbenaceae	*Lantana camara*	GQ478094	ITS
Verbenaceae	*Lantana camara*	AF315303	matK
Verbenaceae	*Lantana camara*	GQ429057	matK
Verbenaceae	*Lantana camara*	GU134977	matK
Verbenaceae	*Lantana camara*	HM850972	matK
Verbenaceae	*Lantana camara*	HM853859	matK
Verbenaceae	*Lantana camara*	JF270846	matK
Verbenaceae	*Lantana camara*	JQ589434-JQ589442	matK
Verbenaceae	*Lantana camara*	JQ589773	matK
Verbenaceae	*Lantana camara*	JX495729	matK
Verbenaceae	*Lantana camara*	GQ429115	psbA-trnH
Verbenaceae	*Lantana camara*	GU135307	psbA-trnH
Verbenaceae	*Lantana camara*	AF156736	rbcL
Verbenaceae	*Lantana camara*	GU135140	rbcL
Verbenaceae	*Lantana camara*	HM850104	rbcL
Verbenaceae	*Lantana camara*	JF265499	rbcL
Verbenaceae	*Lantana camara*	JQ594382-JQ594389	rbcL
Verbenaceae	*Lantana camara*	JQ618495	rbcL
Verbenaceae	*Lantana camara*	JQ618496	rbcL
Verbenaceae	*Lantana camara*	JQ618497	rbcL
Verbenaceae	*Lantana camara*	JQ618498	rbcL
Verbenaceae	*Lantana camara*	JX571858	rbcL
Verbenaceae	*Lantana camara*	KF425765	rbcL
Verbenaceae	*Lantana camara*	GQ429086	rpoC1
Verbenaceae	*Lantana camara*	AB817352	trnL
Verbenaceae	*Lantana camara*	AB817420	trnL
Verbenaceae	*Lantana camara*	AB817542	trnL
Verbenaceae	*Lantana camara*	AF231884	trnL

**Family**	**Species**	**Accession**	**Barcode**
Verbenaceae	*Lantana camara*	AF380872	trnL
Verbenaceae	*Lantana camara*	AY008824	trnL
Verbenaceae	*Lantana camara*	HM216633	trnL
Verbenaceae	*Lantana camara*	JF804911	trnL
Verbenaceae	*Lantana camara*	JX966966	trnL
Verbenaceae	*Lantana camara*	KC428545	trnL
Verbenaceae	*Petrea volubilis*	DQ070732	ITS
Verbenaceae	*Petrea volubilis*	DQ070733	ITS
Verbenaceae	*Petrea volubilis*	FJ514600	matK
Verbenaceae	*Petrea volubilis*	FN773552	matK
Verbenaceae	*Petrea volubilis*	HM853889	matK
Verbenaceae	*Petrea volubilis*	HQ384509	matK
Verbenaceae	*Petrea volubilis*	JQ589449	matK
Verbenaceae	*Petrea volubilis*	JQ589450	matK
Verbenaceae	*Petrea volubilis*	JQ589451	matK
Verbenaceae	*Petrea volubilis*	JQ589452	matK
Verbenaceae	*Petrea volubilis*	JQ589453	matK
Verbenaceae	*Petrea volubilis*	JQ589454	matK
Verbenaceae	*Petrea volubilis*	JQ589455	matK
Verbenaceae	*Petrea volubilis*	JQ589456	matK
Verbenaceae	*Petrea volubilis*	JQ589457	matK
Verbenaceae	*Petrea volubilis*	JQ589458	matK
Verbenaceae	*Petrea volubilis*	KJ593988	matK
Verbenaceae	*Petrea volubilis*	KJ593989	matK
Verbenaceae	*Petrea volubilis*	KJ593990	matK
Verbenaceae	*Petrea volubilis*	FJ039088	psbA-trnH
Verbenaceae	*Petrea volubilis*	HG963675	psbA-trnH
Verbenaceae	*Petrea volubilis*	FJ038204	rbcL
Verbenaceae	*Petrea volubilis*	HQ384877	rbcL
Verbenaceae	*Petrea volubilis*	JQ594396	rbcL
Verbenaceae	*Petrea volubilis*	JQ594397	rbcL
Verbenaceae	*Petrea volubilis*	JQ594398	rbcL
Verbenaceae	*Petrea volubilis*	JQ594399	rbcL
Verbenaceae	*Petrea volubilis*	JQ594400	rbcL
Verbenaceae	*Petrea volubilis*	JQ594401	rbcL

Family	Species	Accession	Barcode
Verbenaceae	*Petrea volubilis*	JQ594402	rbcL
Verbenaceae	*Petrea volubilis*	JQ594403	rbcL
Verbenaceae	*Petrea volubilis*	KJ594419	rbcL
Verbenaceae	*Petrea volubilis*	KJ594420	rbcL
Verbenaceae	*Petrea volubilis*	KJ594421	rbcL
Verbenaceae	*Petrea volubilis*	U28879	rbcL
Verbenaceae	*Petrea volubilis*	U28880	rbcL
Verbenaceae	*Petrea volubilis*	FJ038391	rpoB
Verbenaceae	*Petrea volubilis*	FJ038829	rpoC1
Verbenaceae	*Petrea volubilis*	FJ870052	trnL
Verbenaceae	*Petrea volubilis*	FN794069	trnL
Verbenaceae	*Petrea volubilis*	HM216654	trnL
Verbenaceae	*Petrea volubilis*	HM216655	trnL
Verbenaceae	*Petrea volubilis*	HQ412934	trnL
Violaceae	*Amphirrhox longifolia*	AB354476	matK
Violaceae	*Amphirrhox longifolia*	FJ514690	matK
Violaceae	*Amphirrhox longifolia*	FJ514708	matK
Violaceae	*Amphirrhox longifolia*	JQ626496	matK
Violaceae	*Amphirrhox longifolia*	FJ039089	psbA-trnH
Violaceae	*Amphirrhox longifolia*	AB354404	rbcL
Violaceae	*Amphirrhox longifolia*	FJ038205	rbcL
Violaceae	*Amphirrhox longifolia*	FJ038206	rbcL
Violaceae	*Amphirrhox longifolia*	GQ428608	rbcL
Violaceae	*Amphirrhox longifolia*	JQ626095	rbcL
Violaceae	*Amphirrhox longifolia*	KC699564	rbcL
Violaceae	*Amphirrhox longifolia*	FJ038392	rpoB
Violaceae	*Amphirrhox longifolia*	FJ038393	rpoB
Violaceae	*Amphirrhox longifolia*	FJ038830	rpoC1
Violaceae	*Amphirrhox longifolia*	FJ038831	rpoC1
Violaceae	*Amphirrhox longifolia*	FJ038832	rpoC1
Violaceae	*Amphirrhox longifolia*	FJ039213	trnL
Violaceae	*Amphirrhox longifolia*	FJ039214	trnL
Violaceae	*Amphirrhox longifolia*	KC699636	trnL
Violaceae	*Amphirrhox longifolia*	KC699637	trnL
Violaceae	*Hybanthus atropurpureus*	KC699574	rbcL

**Family**	**Species**	**Accession**	**Barcode**
Violaceae	*Hybanthus atropurpureus*	KC699650	trnL
Violaceae	*Paypayrola blanchetiana*	KC699606	rbcL
Violaceae	*Paypayrola blanchetiana*	KC699697	trnL
Vochysiaceae	*Callisthene fasciculata*	AH013846	matK
Vochysiaceae	*Qualea grandiflora*	AF368216	matK
Vochysiaceae	*Salvertia convallariodora*	AH013850	matK
Vochysiaceae	*Vochysia tucanorum*	DQ787415	ITS
Vochysiaceae	*Vochysia tucanorum*	AM235665	rbcL
Winteraceae	*Drimys brasiliensis*	FJ539226	ITS
Winteraceae	*Drimys brasiliensis*	FJ539227	ITS
Winteraceae	*Drimys brasiliensis*	FJ539228	ITS
Winteraceae	*Drimys brasiliensis*	FJ539229	ITS
Winteraceae	*Drimys brasiliensis*	FJ539230	ITS
Winteraceae	*Drimys brasiliensis*	FJ539231	ITS
Winteraceae	*Drimys brasiliensis*	FJ539204	psbA-trnH
Winteraceae	*Drimys brasiliensis*	FJ539205	psbA-trnH
Winteraceae	*Drimys brasiliensis*	FJ539206	psbA-trnH
Winteraceae	*Drimys brasiliensis*	FJ539207	psbA-trnH
