# Supplementary material for: Can plant DNA barcoding be implemented in species-rich tropical regions? A perspective from São Paulo State, Brazil
Source: Genet Mol Biol. 2018 Jul-Sep;41(3):661–70. doi: 10.1590/1678-4685-GMB-2017-0282 (PMC6136365; doi:10.1590/1678-4685-GMB-2017-0282)
Supplement: Supplementary file 6 [file 1415-4757-GMB-41-03-2017-0282-20180723-suppl6.pdf]

Supplementary Material to “Can plant DNA barcoding be implemented in species-rich tropical regions? A perspective from São Paulo State, Brazil”

Table S6 - Differences between the proportion of taxa in the SP tree flora and the proportion of taxa with barcoding sequences with DNA barcodes per family and genus. Positive difference values mean that the taxon is currently underrepresented, while negative values mean the opposite (overrepresentation). The *p*-value was obtained based on a Cauchy distribution fitted to the distribution of differences between the proportions mentioned above. The position of each taxon in respect to the 95% and 90% confidence intervals of the fitted distribution was used here as a test of how strong this taxon was under or overrepresented. Because this test was performed separately at genus and family level, monogeneric families may not be significantly under or overepresented even if the corresponding genus actually is.

Family and genera	Number of taxa	Proportion of taxa	Proportion of barcoded taxa	Diff.	Sampling (*p*-value)
ACANTHACEAE	3	0.14	0.25	-0.11	
Avicennia	2	0.09	0.16	-0.07	
Ruellia	1	0.05	0.08	-0.03	
ACHATOCARPACEAE	1	0.05	0.08	-0.03	
Achatocarpus	1	0.05	0.08	-0.03	
ADOXACEAE	1	0.05	0.08	-0.03	
Sambucus	1	0.05	0.08	-0.03	
ANACARDIACEAE	14	0.66	0.74	-0.08	
Anacardium	2	0.09	0.08	0.01	
Astronium	1	0.05	0.08	-0.03	
Lithrea	2	0.09	0.08	0.01	
Mangifera	1	0.05	0.08	-0.03	
Myracrodruon	1	0.05	0.08	-0.03	
Schinus	3	0.14	0.08	0.11	under (p<0.05)
Spondias	1	0.05	0.08	-0.03	
Tapirira	2	0.09	0.16	-0.07	
ANNONACEAE	35	1.66	1.97	-0.31	
Anaxagorea	1	0.05	0.08	-0.03	
Annona	18	0.85	0.9	0	
Duguetia	3	0.14	0.25	-0.11	

**Family and genera**	**Number of taxa**	**Proportion of taxa**	**Proportion of barcoded taxa**	**Diff.**	**Sampling (*p*-value)**
Guatteria	4	0.19	0.33	-0.14	
Porcelia	1	0.05	0	0.05	
Trigynaea	1	0.05	0	0.05	
Unonopsis	1	0.05	0.08	-0.03	
Xylopia	5	0.24	0.33	-0.09	
APOCYNACEAE	29	1.37	1.39	-0.02	
Aspidosperma	18	0.85	0.65	0.25	under (p<0.05)
Calotropis	1	0.05	0.08	-0.03	
Hancornia	1	0.05	0.08	-0.03	
Himatanthus	2	0.09	0.08	0.01	
Malouetia	1	0.05	0.08	-0.03	
Rauvolfia	1	0.05	0.08	-0.03	
Tabernaemontana	3	0.14	0.25	-0.11	
Thevetia	1	0.05	0.08	-0.03	
AQUIFOLIACEAE	13	0.61	0.9	-0.29	
Ilex	13	0.61	0.9	-0.29	over (p<0.05)
ARALIACEAE	16	0.76	0.9	-0.14	
Aralia	1	0.05	0.08	-0.03	
Dendropanax	6	0.28	0.25	0.03	
Oreopanax	2	0.09	0.08	0.01	
Schefflera	7	0.33	0.49	-0.16	
ARAUCARIACEAE	1	0.05	0.08	-0.03	
Araucaria	1	0.05	0.08	-0.03	
ARECACEAE	22	1.04	1.15	-0.11	
Acrocomia	1	0.05	0.08	-0.03	
Archontophoenix	1	0.05	0.08	-0.03	
Astrocaryum	1	0.05	0.08	-0.03	
Attalea	4	0.19	0.16	0.03	
Bactris	3	0.14	0.16	-0.02	
Butia	2	0.09	0.08	0.01	
Euterpe	1	0.05	0.08	-0.03	
Geonoma	3	0.14	0.16	-0.02	
Lytocaryum	1	0.05	0	0.05	
Mauritia	1	0.05	0.08	-0.03	

**Family and genera**	**Number of taxa**	**Proportion of taxa**	**Proportion of barcoded taxa**	**Diff.**	**Sampling (*p*-value)**
Syagrus	4	0.19	0.16	0.03	
ASTERACEAE	63	2.98	2.38	0.6	under (p<0.05)
Austrocritonia	2	0.09	0.16	-0.07	
Austroeupatorium	1	0.05	0.08	-0.03	
Baccharis	18	0.85	0.08	0.77	under (p<0.01)
Critoniopsis	2	0.09	0	0.09	
Dasyphyllum	2	0.09	0.16	-0.07	
Dendrophorbium	1	0.05	0	0.05	
Eremanthus	3	0.14	0.25	-0.11	
Gochnatia	7	0.33	0.41	-0.08	
Idiothamnus	1	0.05	0	0.05	
Kaunia	1	0.05	0.08	-0.03	
Lychnophora	1	0.05	0.08	-0.03	
Piptocarpha	9	0.43	0.33	0.1	
Raulinoreitzia	1	0.05	0.08	-0.03	
Stifftia	3	0.14	0.25	-0.11	
Symphyopappus	2	0.09	0.08	0.01	
Trixis	1	0.05	0	0.05	
Verbesina	1	0.05	0.08	-0.03	
Vernonanthura	6	0.28	0.16	0.12	under (p<0.05)
Wunderlichia	1	0.05	0.08	-0.03	
Moquiniastrum	0	0	0.08	-0.08	
BERBERIDACEAE	1	0.05	0.08	-0.03	
Berberis	1	0.05	0.08	-0.03	
BIGNONIACEAE	31	1.47	1.72	-0.25	
Cybistax	1	0.05	0.08	-0.03	
Handroanthus	12	0.57	0.65	-0.08	
Jacaranda	8	0.38	0.25	0.13	under (p<0.05)
Sparattosperma	1	0.05	0.08	-0.03	
Spathodea	1	0.05	0.08	-0.03	
Tabebuia	5	0.24	0.33	-0.09	
Tecoma	1	0.05	0.08	-0.03	
Zeyheria	2	0.09	0.16	-0.07	
BIXACEAE	2	0.09	0.08	0.01	

**Family and genera**	**Number of taxa**	**Proportion of taxa**	**Proportion of barcoded taxa**	**Diff.**	**Sampling (*p*-value)**
Bixa	2	0.09	0.08	0.01	
BORAGINACEAE	13	0.61	0.82	-0.21	
Cordia	12	0.57	0.74	-0.17	
Tournefortia	1	0.05	0.08	-0.03	
BURSERACEAE	7	0.33	0.57	-0.24	
Protium	7	0.33	0.57	-0.24	over (p<0.05)
CACTACEAE	10	0.47	0.66	-0.19	
Arthrocereus	1	0.05	0	0.05	
Brasiliopuntia	1	0.05	0.08	-0.03	
Cereus	3	0.14	0.16	-0.02	
Coleocephalocereus	1	0.05	0.08	-0.03	
Opuntia	1	0.05	0.08	-0.03	
Pereskia	2	0.09	0.16	-0.07	
Pilosocereus	1	0.05	0.08	-0.03	
CALOPHYLLACEAE	9	0.43	0.25	0.18	
Calophyllum	1	0.05	0.08	-0.03	
Kielmeyera	8	0.38	0.16	0.22	under (p<0.05)
CANELLACEAE	2	0.09	0.16	-0.07	
Cinnamodendron	2	0.09	0.16	-0.07	
CANNABACEAE	6	0.28	0.41	-0.13	
Celtis	5	0.24	0.33	-0.09	
Trema	1	0.05	0.08	-0.03	
CAPPARACEAE	3	0.14	0.25	-0.11	
Capparidastrum	1	0.05	0.08	-0.03	
Crateva	1	0.05	0.08	-0.03	
Cynophalla	1	0.05	0.08	-0.03	
CARDIOPTERIDACEAE	2	0.09	0.08	0.01	
Citronella	2	0.09	0.08	0.01	
CARICACEAE	5	0.24	0.41	-0.17	
Carica	1	0.05	0.08	-0.03	
Jacaratia	2	0.09	0.16	-0.07	
Vasconcellea	2	0.09	0.16	-0.07	
CARYOCARACEAE	1	0.05	0.08	-0.03	
Caryocar	1	0.05	0.08	-0.03	

Family and genera	Number of taxa	Proportion of taxa	Proportion of barcoded taxa	Diff.	Sampling (*p*-value)
CASUARINACEAE	1	0.05	0.08	-0.03	
Casuarina	1	0.05	0.08	-0.03	
CELASTRACEAE	29	1.37	1.31	0.06	
Cheiloclinium	1	0.05	0.08	-0.03	
Maytenus	17	0.8	0.65	0.15	under (p<0.05)
Peritassa	1	0.05	0.08	-0.03	
Plenckia	1	0.05	0.08	-0.03	
Salacia	4	0.19	0.33	-0.14	
Tontelea	4	0.19	0.08	0.16	under (p<0.05)
CHLORANTHACEAE	1	0.05	0.08	-0.03	
Hedyosmum	1	0.05	0.08	-0.03	
CHRYSOBALANACEAE	24	1.14	0.9	0.24	
Chrysobalanus	1	0.05	0.08	-0.03	
Couepia	6	0.28	0.08	0.2	under (p<0.05)
Hirtella	6	0.28	0.33	-0.05	
Licania	9	0.43	0.33	0.1	
Parinari	2	0.09	0.08	0.01	
CLETHRACEAE	1	0.05	0.08	-0.03	
Clethra	1	0.05	0.08	-0.03	
CLUSIACEAE	7	0.33	0.49	-0.16	
Clusia	4	0.19	0.25	-0.06	
Garcinia	1	0.05	0.08	-0.03	
Tovomitopsis	2	0.09	0.16	-0.07	
COMBRETACEAE	15	0.71	0.49	0.22	
Buchenavia	4	0.19	0.08	0.11	under (p<0.05)
Combretum	2	0.09	0.08	0.01	
Conocarpus	1	0.05	0.08	-0.03	
Laguncularia	1	0.05	0.08	-0.03	
Terminalia	7	0.33	0.16	0.17	under (p<0.05)
CONNARACEAE	6	0.28	0.33	-0.05	
Bernardinia	1	0.05	0.08	-0.03	
Connarus	3	0.14	0.16	-0.02	
Rourea	2	0.09	0.08	0.01	
CUNONIACEAE	9	0.43	0.16	0.27	

**Family and genera**	**Number of taxa**	**Proportion of taxa**	**Proportion of barcoded taxa**	**Diff.**	**Sampling (*p*-value)**
Lamanonia	4	0.19	0.08	0.11	under (p<0.05)
Weinmannia	5	0.24	0.08	0.16	under (p<0.05)
CYATHEACEAE	17	0.8	0.49	0.31	
Alsophila	3	0.14	0.16	-0.02	
Cyathea	14	0.66	0.33	0.33	under (p<0.01)
DICHAPETALACEAE	4	0.19	0.08	0.11	
Stephanopodium	4	0.19	0.08	0.11	under (p<0.05)
DICKSONIACEAE	1	0.05	0.08	-0.03	
Dicksonia	1	0.05	0.08	-0.03	
DILLENIACEAE	3	0.14	0.16	-0.02	
Curatella	1	0.05	0.08	-0.03	
Davilla	2	0.09	0.08	0.01	
EBENACEAE	3	0.14	0.16	-0.02	
Diospyros	3	0.14	0.16	-0.02	
ELAEOCARPACEAE	7	0.33	0.33	0	
Sloanea	7	0.33	0.33	0	
ERICACEAE	9	0.43	0.16	0.27	
Agarista	5	0.24	0	0.24	under (p<0.05)
Gaultheria	1	0.05	0.08	-0.03	
Gaylussacia	3	0.14	0.08	0.06	
ERYTHROXYLACEAE	21	0.99	0.57	0.42	
Erythroxylum	21	0.99	0.57	0.42	under (p<0.01)
ESCALLONIACEAE	4	0.19	0.33	-0.14	
Escallonia	4	0.19	0.33	-0.14	
EUPHORBIACEAE	64	3.03	3.36	-0.33	
Acalypha	2	0.09	0.08	0.01	
Actinostemon	6	0.28	0.16	0.12	under (p<0.05)
Adelia	1	0.05	0.08	-0.03	
Alchornea	3	0.14	0.16	-0.02	
Algernonia	3	0.14	0	0.14	under (p<0.05)
Aparisthmium	1	0.05	0.08	-0.03	
Bernardia	1	0.05	0.08	-0.03	
Caryodendron	1	0.05	0	0.05	
Croton	16	0.76	1.14	-0.38	over (p<0.05)

**Family and genera**	**Number of taxa**	**Proportion of taxa**	**Proportion of barcoded taxa**	**Diff.**	**Sampling (*p*-value)**
Euphorbia	1	0.05	0.08	-0.03	
Gymnanthes	3	0.14	0	0.14	under (p<0.05)
Jatropha	1	0.05	0.08	-0.03	
Joannesia	1	0.05	0.08	-0.03	
Mabea	2	0.09	0.16	-0.07	
Manihot	5	0.24	0.33	-0.09	
Maprounea	2	0.09	0.08	0.01	
Micrandra	1	0.05	0	0.05	
Ophthalmoblapton	1	0.05	0	0.05	
Pachystroma	1	0.05	0.08	-0.03	
Pausandra	1	0.05	0	0.05	
Philyra	1	0.05	0.08	-0.03	
Pleradenophora	1	0.05	0	0.05	
Sapium	3	0.14	0.16	-0.02	
Sebastiania	1	0.05	0.16	-0.11	
Senefeldera	1	0.05	0	0.05	
Stillingia	2	0.09	0.08	0.01	
Tetrorchidium	2	0.09	0.08	0.01	
FABACEAE	211	9.98	12.62	-2.64	over (p<0.01)
Abarema	3	0.14	0.16	-0.02	
Acacia	1	0.05	0.08	-0.03	
Albizia	5	0.24	0.25	-0.01	
Anadenanthera	2	0.09	0.16	-0.07	
Andira	6	0.28	0.49	-0.21	over (p<0.05)
Apuleia	1	0.05	0.08	-0.03	
Ateleia	1	0.05	0.08	-0.03	
Bauhinia	9	0.43	0.57	-0.14	
Bowdichia	1	0.05	0.08	-0.03	
Caesalpinia	1	0.05	0.08	-0.03	
Calliandra	3	0.14	0.25	-0.11	
Cassia	2	0.09	0.08	0.01	
Centrolobium	2	0.09	0.16	-0.07	
Chamaecrista	3	0.14	0	0.14	under (p<0.05)
Chloroleucon	2	0.09	0.16	-0.07	

**Family and genera**	**Number of taxa**	**Proportion of taxa**	**Proportion of barcoded taxa**	**Diff.**	**Sampling (*p*-value)**
Clitoria	1	0.05	0	0.05	
Copaifera	3	0.14	0.16	-0.02	
Cyclolobium	1	0.05	0.08	-0.03	
Dahlstedtia	2	0.09	0.25	-0.16	
Dalbergia	8	0.38	0.49	-0.11	
Dimorphandra	2	0.09	0.08	0.01	
Dipteryx	1	0.05	0.08	-0.03	
Diptychandra	1	0.05	0.08	-0.03	
Enterolobium	3	0.14	0.16	-0.02	
Erythrina	5	0.24	0.16	0.08	
Exostyles	2	0.09	0.16	-0.07	
Holocalyx	1	0.05	0.08	-0.03	
Hymenaea	3	0.14	0.16	-0.02	
Hymenolobium	1	0.05	0.08	-0.03	
Inga	24	1.14	1.06	0.08	
Leptolobium	2	0.09	0.16	-0.07	
Leucaena	1	0.05	0.08	-0.03	
Leucochloron	1	0.05	0.08	-0.03	
Lonchocarpus	6	0.28	0.41	-0.13	
Luetzelburgia	1	0.05	0.08	-0.03	
Machaerium	12	0.57	0.57	0	
Melanoxylon	1	0.05	0.08	-0.03	
Mimosa	10	0.47	0.33	0.14	under (p<0.05)
Myrocarpus	2	0.09	0.08	0.01	
Myroxylon	1	0.05	0.08	-0.03	
Ormosia	3	0.14	0.25	-0.11	
Parapiptadenia	1	0.05	0.08	-0.03	
Parkinsonia	1	0.05	0.08	-0.03	
Peltogyne	1	0.05	0.08	-0.03	
Peltophorum	1	0.05	0.08	-0.03	
Piptadenia	3	0.14	0.16	-0.02	
Pithecellobium	1	0.05	0.08	-0.03	
Plathymenia	1	0.05	0.08	-0.03	
Platycyamus	1	0.05	0.08	-0.03	

**Family and genera**	**Number of taxa**	**Proportion of taxa**	**Proportion of barcoded taxa**	**Diff.**	**Sampling (*p*-value)**
Platymiscium	1	0.05	0.08	-0.03	
Platypodium	1	0.05	0.08	-0.03	
Poecilanthe	1	0.05	0.08	-0.03	
Pseudopiptadenia	3	0.14	0.16	-0.02	
Pterocarpus	1	0.05	0.08	-0.03	
Pterodon	2	0.09	0.16	-0.07	
Pterogyne	1	0.05	0.08	-0.03	
Riedeliella	1	0.05	0.08	-0.03	
Schizolobium	1	0.05	0.08	-0.03	
Senegalia	4	0.19	0.16	0.03	
Senna	17	0.8	1.06	-0.26	over (p<0.05)
Sesbania	1	0.05	0.16	-0.11	
Sophora	1	0.05	0.08	-0.03	
Stryphnodendron	3	0.14	0.16	-0.02	
Swartzia	6	0.28	0.49	-0.21	over (p<0.05)
Sweetia	1	0.05	0.08	-0.03	
Tachigali	9	0.43	0.33	0.1	
Tipuana	1	0.05	0.08	-0.03	
Vatairea	2	0.09	0.16	-0.07	
Zollernia	2	0.09	0.16	-0.07	
Zygia	2	0.09	0.08	0.01	
Muellera	0	0	0.08	-0.08	
GRISELINIACEAE	1	0.05	0.08	-0.03	
Griselinia	1	0.05	0.08	-0.03	
HUMIRIACEAE	4	0.19	0.16	0.03	
Humiriastrum	2	0.09	0.08	0.01	
Sacoglottis	1	0.05	0	0.05	
Vantanea	1	0.05	0.08	-0.03	
HYPERICACEAE	3	0.14	0.08	0.06	
Vismia	3	0.14	0.08	0.06	
LACISTEMATACEAE	5	0.24	0.16	0.08	
Lacistema	5	0.24	0.16	0.08	
LAMIACEAE	18	0.85	0.57	0.28	
Aegiphila	9	0.43	0.25	0.18	under (p<0.05)

**Family and genera**	**Number of taxa**	**Proportion of taxa**	**Proportion of barcoded taxa**	**Diff.**	**Sampling (*p*-value)**
Cyanocephalus	1	0.05	0	0.05	
Hyptidendron	1	0.05	0.08	-0.03	
Vitex	7	0.33	0.25	0.08	
LAURACEAE	105	4.97	4.51	0.46	
Aiouea	5	0.24	0.16	0.08	
Aniba	3	0.14	0.16	-0.02	
Beilschmiedia	2	0.09	0.08	0.01	
Cinnamomum	6	0.28	0.08	0.2	under (p<0.05)
Cryptocarya	7	0.33	0.41	-0.08	
Endlicheria	1	0.05	0.08	-0.03	
Licaria	1	0.05	0.08	-0.03	
Nectandra	17	0.8	0.9	-0.1	
Ocotea	52	2.46	2.04	0.42	under (p<0.01)
Persea	9	0.43	0.41	0.02	
Rhodostemonodaphne	1	0.05	0	0.05	
Urbanodendron	1	0.05	0.08	-0.03	
LAXMANNIACEAE	1	0.05	0.08	-0.03	
Cordyline	1	0.05	0.08	-0.03	
LECYTHIDACEAE	4	0.19	0.33	-0.14	
Cariniana	2	0.09	0.16	-0.07	
Lecythis	2	0.09	0.16	-0.07	
LOGANIACEAE	3	0.14	0.25	-0.11	
Strychnos	3	0.14	0.25	-0.11	
LYTHRACEAE	3	0.14	0.16	-0.02	
Diplusodon	1	0.05	0.08	-0.03	
Lafoensia	2	0.09	0.08	0.01	
MAGNOLIACEAE	1	0.05	0.08	-0.03	
Magnolia	1	0.05	0.08	-0.03	
MALPIGHIACEAE	21	0.99	0.9	0.09	
Banisteriopsis	1	0.05	0.08	-0.03	
Barnebya	1	0.05	0.08	-0.03	
Bunchosia	2	0.09	0.16	-0.07	
Byrsonima	16	0.76	0.49	0.27	under (p<0.05)
Heteropterys	1	0.05	0.08	-0.03	

**Family and genera**	**Number of taxa**	**Proportion of taxa**	**Proportion of barcoded taxa**	**Diff.**	**Sampling (*p*-value)**
MALVACEAE	37	1.75	1.56	0.19	
Akrosida	1	0.05	0	0.05	
Apeiba	1	0.05	0.08	-0.03	
Bastardiopsis	1	0.05	0.08	-0.03	
Callianthe	1	0.05	0	0.05	
Ceiba	2	0.09	0.08	0.01	
Christiana	1	0.05	0	0.05	
Eriotheca	4	0.19	0.33	-0.14	
Guazuma	2	0.09	0.16	-0.07	
Helicteres	4	0.19	0	0.19	under (p<0.05)
Heliocarpus	1	0.05	0.08	-0.03	
Luehea	5	0.24	0.16	0.08	
Pachira	2	0.09	0.08	0.01	
Pavonia	1	0.05	0	0.05	
Pseudabutilon	1	0.05	0	0.05	
Pseudobombax	4	0.19	0.25	-0.06	
Quararibea	1	0.05	0.08	-0.03	
Spirotheca	1	0.05	0.08	-0.03	
Sterculia	2	0.09	0.08	0.01	
Talipariti	1	0.05	0	0.05	
Urena	1	0.05	0	0.05	
MELASTOMATACEAE	121	5.72	6.97	-1.25	over (p<0.01)
Behuria	1	0.05	0.08	-0.03	
Graffenrieda	1	0.05	0	0.05	
Henriettea	2	0.09	0.08	0.01	
Huberia	4	0.19	0	0.19	under (p<0.05)
Leandra	22	1.04	1.64	-0.6	over (p<0.01)
Macairea	1	0.05	0.08	-0.03	
Meriania	3	0.14	0	0.14	under (p<0.05)
Miconia	62	2.93	3.6	-0.67	over (p<0.01)
Mouriri	3	0.14	0.16	-0.02	
Ossaea	2	0.09	0.25	-0.16	
Pleroma	1	0.05	0	0.05	
Tibouchina	17	0.8	0.9	-0.1	

Family and genera	Number of taxa	Proportion of taxa	Proportion of barcoded taxa	Diff.	Sampling (*p*-value)
Tococa	1	0.05	0.08	-0.03	
Trembleya	1	0.05	0.08	-0.03	
MELIACEAE	20	0.95	1.31	-0.36	
Cabralea	1	0.05	0.08	-0.03	
Cedrela	2	0.09	0.16	-0.07	
Guarea	3	0.14	0.25	-0.11	
Melia	1	0.05	0.08	-0.03	
Trichilia	13	0.61	0.74	-0.13	
MONIMIACEAE	26	1.23	0.74	0.49	
Hennecartia	1	0.05	0.08	-0.03	
Macropeplus	2	0.09	0	0.09	
Macrotorus	1	0.05	0.08	-0.03	
Mollinedia	22	1.04	0.57	0.47	under (p<0.01)
MORACEAE	40	1.89	2.46	-0.57	over (p<0.05)
Artocarpus	2	0.09	0.16	-0.07	
Brosimum	4	0.19	0.25	-0.06	
Clarisia	1	0.05	0	0.05	
Ficus	23	1.09	1.47	-0.38	over (p<0.05)
Maclura	1	0.05	0.08	-0.03	
Morus	2	0.09	0.16	-0.07	
Pseudolmedia	2	0.09	0.08	0.01	
Sorocea	5	0.24	0.25	-0.01	
MYRISTICACEAE	3	0.14	0.16	-0.02	
Virola	3	0.14	0.16	-0.02	
MYRTACEAE	324	15.33	12.62	2.71	under (p<0.01)
Acca	1	0.05	0.08	-0.03	
Blepharocalyx	1	0.05	0.08	-0.03	
Calyptranthes	23	1.09	0.33	0.76	under (p<0.01)
Campomanesia	14	0.66	0.41	0.25	under (p<0.05)
Eugenia	116	5.53	4.58	0.95	under (p<0.01)
Marlierea	20	0.95	0.82	0.13	under (p<0.05)
Myrceugenia	25	1.18	1.47	-0.29	over (p<0.05)
Myrcia	68	3.24	3.11	0.15	under (p<0.05)
Myrcianthes	3	0.14	0.16	-0.02	

**Family and genera**	**Number of taxa**	**Proportion of taxa**	**Proportion of barcoded taxa**	**Diff.**	**Sampling (*p*-value)**
Myrciaria	5	0.24	0.16	0.08	
Myrrhinium	1	0.05	0	0.05	
Neomitranthes	8	0.38	0.08	0.3	under (p<0.05)
Pimenta	1	0.05	0.08	-0.03	
Plinia	10	0.47	0.41	0.06	
Psidium	18	0.85	0.41	0.44	under (p<0.01)
Siphoneugena	6	0.28	0.25	0.03	
Syzygium	2	0.09	0.16	-0.07	
NYCTAGINACEAE	16	0.76	0.82	-0.06	
Bougainvillea	2	0.09	0.16	-0.07	
Guapira	8	0.38	0.41	-0.03	
Neea	4	0.19	0.08	0.11	under (p<0.05)
Pisonia	2	0.09	0.16	-0.07	
OCHNACEAE	12	0.57	0.57	0	
Ouratea	12	0.57	0.49	0.08	
OLACACEAE	4	0.19	0.25	-0.06	
Heisteria	2	0.09	0.08	0.01	
Tetrastylidium	1	0.05	0.08	-0.03	
Ximenia	1	0.05	0.08	-0.03	
OLEACEAE	5	0.24	0.16	0.08	
Chionanthus	4	0.19	0.08	0.11	under (p<0.05)
Ligustrum	1	0.05	0.08	-0.03	
ONAGRACEAE	3	0.14	0.16	-0.02	
Fuchsia	1	0.05	0.08	-0.03	
Ludwigia	2	0.09	0.08	0.01	
OPILIACEAE	2	0.09	0.08	0.01	
Agonandra	2	0.09	0.08	0.01	
PENTAPHYLACACEAE	2	0.09	0.08	0.01	
Ternstroemia	2	0.09	0.08	0.01	
PERACEAE	3	0.14	0.16	-0.02	
Chaetocarpus	1	0.05	0	0.05	
Pera	2	0.09	0.16	-0.07	
PHYLLANTHACEAE	11	0.52	0.66	-0.14	
Gonatogyne	1	0.05	0.08	-0.03	

**Family and genera**	**Number of taxa**	**Proportion of taxa**	**Proportion of barcoded taxa**	**Diff.**	**Sampling (*p*-value)**
Hyeronima	2	0.09	0.16	-0.07	
Margaritaria	1	0.05	0.08	-0.03	
Phyllanthus	5	0.24	0.16	0.08	
Richeria	1	0.05	0.08	-0.03	
Savia	1	0.05	0.08	-0.03	
PHYTOLACCACEAE	4	0.19	0.25	-0.06	
Gallesia	1	0.05	0.08	-0.03	
Phytolacca	1	0.05	0.08	-0.03	
Seguieria	2	0.09	0.08	0.01	
PICRAMNIACEAE	6	0.28	0.25	0.03	
Picramnia	6	0.28	0.25	0.03	
PINACEAE	3	0.14	0.25	-0.11	
Pinus	3	0.14	0.25	-0.11	
PIPERACEAE	18	0.85	0.9	-0.05	
Piper	18	0.85	0.9	-0.05	
PITTOSPORACEAE	1	0.05	0.08	-0.03	
Pittosporum	1	0.05	0.08	-0.03	
POACEAE	45	2.13	1.39	0.74	under (p<0.05)
Actinocladum	1	0.05	0.08	-0.03	
Apoclada	1	0.05	0.08	-0.03	
Aulonemia	3	0.14	0.08	0.11	
Bambusa	6	0.28	0.41	-0.13	
Chusquea	7	0.33	0.33	0	
Eremocaulon	1	0.05	0	0.05	
Guadua	6	0.28	0.25	0.03	
Merostachys	16	0.76	0.08	0.68	under (p<0.01)
Phyllostachys	3	0.14	0.08	0.06	
PODOCARPACEAE	2	0.09	0.16	-0.07	
Podocarpus	2	0.09	0.16	-0.07	
POLYGALACEAE	5	0.24	0.25	-0.01	
Acanthocladus	1	0.05	0.08	-0.03	
Asemeia	1	0.05	0.08	-0.03	
Bredemeyera	3	0.14	0.08	0.06	
POLYGONACEAE	14	0.66	0.49	0.17	

**Family and genera**	**Number of taxa**	**Proportion of taxa**	**Proportion of barcoded taxa**	**Diff.**	**Sampling (*p*-value)**
Coccoloba	10	0.47	0.16	0.31	under (p<0.01)
Ruprechtia	2	0.09	0.16	-0.07	
Triplaris	2	0.09	0.16	-0.07	
PRIMULACEAE	33	1.56	0.98	0.58	under (p<0.05)
Ardisia	1	0.05	0	0.05	
Clavija	2	0.09	0.16	-0.07	
Cybianthus	6	0.28	0.08	0.2	under (p<0.05)
Geissanthus	1	0.05	0	0.05	
Myrsine	19	0.9	0.57	0.33	under (p<0.01)
Stylogyne	4	0.19	0.16	0.03	
PROTEACEAE	12	0.57	0.16	0.41	
Euplassa	6	0.28	0	0.28	under (p<0.05)
Grevillea	1	0.05	0.08	-0.03	
Panopsis	1	0.05	0	0.05	
Roupala	4	0.19	0.08	0.11	under (p<0.05)
QUIINACEAE	2	0.09	0	0.09	
Quiina	2	0.09	0.08	0.01	
RHAMNACEAE	8	0.38	0.41	-0.03	
Colubrina	2	0.09	0.08	0.01	
Condalia	1	0.05	0	0.05	
Hovenia	1	0.05	0.08	-0.03	
Rhamnidium	2	0.09	0.16	-0.07	
Rhamnus	1	0.05	0.08	-0.03	
Scutia	1	0.05	0	0.05	
RHIZOPHORACEAE	1	0.05	0.08	-0.03	
Rhizophora	1	0.05	0.08	-0.03	
ROSACEAE	3	0.14	0.16	-0.02	
Eriobotrya	1	0.05	0.08	-0.03	
Prunus	2	0.09	0.08	0.01	
RUBIACEAE	126	5.96	5.08	0.88	under (p<0.05)
Alibertia	1	0.05	0.08	-0.03	
Alseis	2	0.09	0.08	0.01	
Amaioua	2	0.09	0.16	-0.07	
Bathysa	4	0.19	0.25	-0.06	

**Family and genera**	**Number of taxa**	**Proportion of taxa**	**Proportion of barcoded taxa**	**Diff.**	**Sampling (*p*-value)**
Chomelia	8	0.38	0.16	0.22	under (p<0.05)
Coffea	1	0.05	0.08	-0.03	
Cordiera	6	0.28	0.41	-0.13	
Coussarea	8	0.38	0.33	0.05	
Coutarea	1	0.05	0.08	-0.03	
Faramea	12	0.57	0.25	0.32	under (p<0.01)
Genipa	2	0.09	0.08	0.01	
Guettarda	4	0.19	0.25	-0.06	
Hamelia	1	0.05	0.08	-0.03	
Ixora	6	0.28	0.25	0.03	
Ladenbergia	1	0.05	0	0.05	
Machaonia	1	0.05	0	0.05	
Margaritopsis	4	0.19	0.08	0.11	under (p<0.05)
Palicourea	3	0.14	0.25	-0.11	
Posoqueria	3	0.14	0.08	0.06	
Psychotria	27	1.28	1.14	0.18	under (p<0.05)
Randia	3	0.14	0.25	-0.11	
Rudgea	14	0.66	0.33	0.33	under (p<0.01)
Rustia	2	0.09	0.08	0.01	
Schizocalyx	1	0.05	0.08	-0.03	
Simira	5	0.24	0.16	0.08	
Tocoyena	3	0.14	0.08	0.06	
RUTACEAE	32	1.51	2.05	-0.54	over (p<0.05)
Almeidea	1	0.05	0.08	-0.03	
Balfourodendron	1	0.05	0.08	-0.03	
Citrus	2	0.09	0.16	-0.07	
Conchocarpus	2	0.09	0.16	-0.07	
Dictyoloma	1	0.05	0.08	-0.03	
Esenbeckia	5	0.24	0.25	-0.01	
Galipea	1	0.05	0.08	-0.03	
Helietta	1	0.05	0.08	-0.03	
Hortia	1	0.05	0.08	-0.03	
Metrodorea	2	0.09	0.16	-0.07	
Murraya	1	0.05	0.08	-0.03	

Family and genera	Number of taxa	Proportion of taxa	Proportion of barcoded taxa	Diff.	Sampling (*p*-value)
Neoraputia	1	0.05	0	0.05	
Pilocarpus	4	0.19	0.25	-0.06	
Zanthoxylum	9	0.43	0.49	-0.06	
SABIACEAE	3	0.14	0.16	-0.02	
Meliosma	3	0.14	0.16	-0.02	
SALICACEAE	25	1.18	1.07	0.11	
Azara	1	0.05	0	0.05	
Banara	3	0.14	0.08	0.06	
Casearia	13	0.61	0.57	0.04	
Prockia	1	0.05	0.08	-0.03	
Salix	1	0.05	0.08	-0.03	
Xylosma	6	0.28	0.25	0.03	
SAPINDACEAE	33	1.56	1.31	0.25	
Allophylus	7	0.33	0.25	0.13	under ($p<0.05$)
Cupania	9	0.43	0.25	0.18	under ($p<0.05$)
Diatenopteryx	1	0.05	0.08	-0.03	
Dilodendron	1	0.05	0.08	-0.03	
Dodonaea	1	0.05	0.08	-0.03	
Magonia	1	0.05	0.08	-0.03	
Matayba	7	0.33	0.33	0	
Sapindus	1	0.05	0.08	-0.03	
Talisia	2	0.09	0.08	0.01	
Toulicia	1	0.05	0	0.05	
Tripterodendron	1	0.05	0	0.05	
SAPOTACEAE	31	1.47	2.21	-0.74	over ($p<0.05$)
Chrysophyllum	7	0.33	0.41	-0.08	
Diploon	1	0.05	0.08	-0.03	
Ecclinusa	1	0.05	0.08	-0.03	
Manilkara	2	0.09	0.16	-0.07	
Micropholis	4	0.19	0.33	-0.14	
Pouteria	14	0.66	0.98	-0.32	over ($p<0.05$)
Pradosia	1	0.05	0.08	-0.03	
Sideroxylon	1	0.05	0.08	-0.03	
SCHOEPFIACEAE	1	0.05	0	0.05	

**Family and genera**	**Number of taxa**	**Proportion of taxa**	**Proportion of barcoded taxa**	**Diff.**	**Sampling (*p*-value)**
Schoepfia	1	0.05	0	0.05	
SIMAROUBACEAE	2	0.09	0.16	-0.07	
Picrasma	1	0.05	0.08	-0.03	
Simaba	1	0.05	0.08	-0.03	
SIPARUNACEAE	4	0.19	0.33	-0.14	
Siparuna	4	0.19	0.33	-0.14	
SOLANACEAE	77	3.64	3.28	0.36	
Acnistus	1	0.05	0.08	-0.03	
Athenaea	1	0.05	0.25	-0.11	
Aureliana	6	0.28	0.49	-0.11	
Brugmansia	1	0.05	0.08	-0.03	
Brunfelsia	3	0.14	0.25	-0.11	
Capsicum	3	0.14	0.16	-0.02	
Cestrum	11	0.52	0.33	0.19	under (p<0.05)
Dyssochroma	1	0.05	0.08	-0.03	
Lycianthes	1	0.05	0.08	-0.03	
Sessea	2	0.09	0	0.09	
Solanum	41	1.96	1.39	0.6	under (p<0.01)
Vassobia	1	0.05	0.08	-0.03	
STYRACACEAE	10	0.47	0.66	-0.19	
Styrax	10	0.47	0.65	-0.18	
SYMPLOCACEAE	25	1.18	1.56	-0.38	
Symplocos	24	1.14	1.55	-0.37	over (p<0.05)
THEACEAE	1	0.05	0.08	-0.03	
Laplacea	1	0.05	0.08	-0.03	
THYMELAEACEAE	8	0.38	0.25	0.13	
Daphnopsis	8	0.38	0.25	0.13	under (p<0.05)
ULMACEAE	1	0.05	0.08	-0.03	
Phyllostylon	1	0.05	0.08	-0.03	
URTICACEAE	11	0.52	0.66	-0.14	
Boehmeria	2	0.09	0.08	0.01	
Cecropia	3	0.14	0.16	-0.02	
Coussapoa	1	0.05	0.08	-0.03	
Myriocarpa	1	0.05	0	0.05	

**Family and genera**	**Number of taxa**	**Proportion of taxa**	**Proportion of barcoded taxa**	**Diff.**	**Sampling (*p*-value)**
Pourouma	1	0.05	0.08	-0.03	
Urera	3	0.14	0.25	-0.11	
VERBENACEAE	9	0.43	0.41	0.02	
Aloysia	1	0.05	0.08	-0.03	
Citharexylum	5	0.24	0.16	0.08	
Duranta	1	0.05	0	0.05	
Lantana	1	0.05	0.08	-0.03	
Petrea	1	0.05	0.08	-0.03	
VIOLACEAE	3	0.14	0.25	-0.11	
Amphirrhox	1	0.05	0.08	-0.03	
Hybanthus	1	0.05	0.08	-0.03	
Paypayrola	1	0.05	0.08	-0.03	
Pombalia	0	0	0.08	-0.08	
VOCHYSIACEAE	29	1.37	0.57	0.8	under ($p<0.05$)
Callisthene	5	0.24	0.08	0.16	under ($p<0.05$)
Qualea	10	0.47	0.33	0.14	under ($p<0.05$)
Salvertia	1	0.05	0.08	-0.03	
Vochysia	13	0.61	0.08	0.53	under ($p<0.01$)
WINTERACEAE	1	0.05	0.08	-0.03	
Drimys	1	0.05	0.08	-0.03	
